# Total Synthesis of Benthol A: Evidence for a Structure Revision

**DOI:** 10.1021/jacs.6c05580

**Published:** 2026-07-16

**Authors:** Guanghao Huang, Andrea Tomio, Thomas Varlet, Conny Wirtz, Alois Fürstner

**Affiliations:** 28314 Max-Planck-Institut für Kohlenforschung, 45470 Mülheim/Ruhr, Germany

## Abstract

Benthol A, a dinoflagellate-derived polyol/polyether marine natural product endowed with potent antimalaria and appreciable antiviral activity, consists of a 72 C atom linear backbone featuring 35 stereogenic centers and four stereogenic alkenes. The constitution and configuration of this intriguing “super-carbon-chain compound” had been assigned by the isolation team by a combination of spectroscopic, chemical, and computational means. Outlined in this and the accompanying paper is the first total synthesis of this challenging target, which came along with a subtle structure revision. During the synthesis of the building blocks, a spectral irregularity was noticed in that the ^13^C NMR chemical shifts as well as some of the ^3^ *J*_H,H_ coupling constants of the synthetic samples deviated notably from the data reported for the entire C31–C41 region of benthol A corresponding to the tetrahydropyran E-ring and its vicinity. A systematic approach made it possible to narrow down the likely site of error to a single, incorrectly assigned stereocenter, i.e., the C40 position; interestingly, the configuration of this site had been determined by the isolation team solely by computational means. In chemical terms, our studies showed how the proper choice of a propargylic protecting group allows the regiochemical course of a gold-catalyzed spiroacetalization reaction to be steered. Moreover, carbonyl homologation by the addition of a highly functionalized but entirely unstabilized diazo derivative generated in situ to an equally highly functionalized aldehyde proved adequate for the coupling of elaborate building blocks (Buchner–Curtius–Schlotterbeck reaction).

## Graphical abstract

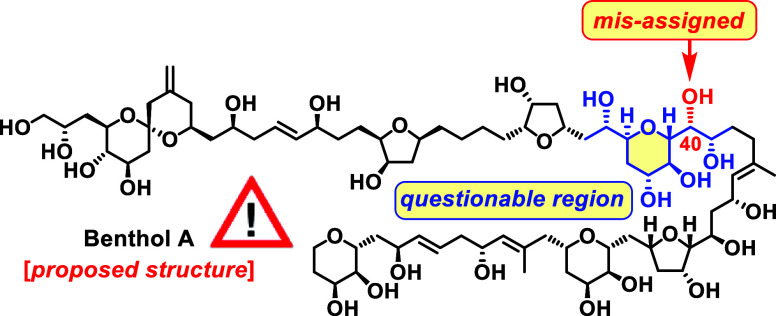

## Introduction

Apart from the eponymous flagella, the morphology of most marine dinoflagellates is rather inconspicuous. The simple macroscopic appearance of these unicellular organisms must not hide the fact that their genome is often disproportionately large.^1^ As a result, numerous members of this taxon exhibit extraordinary biosynthetic capabilities, producing a vast array of structurally highly complex secondary metabolites.^2,3^ Even within this quite spectacular panopticon, the so-called “super-carbon-chain compounds” (SCCCs) of molecular weights well exceeding 1000 Da stand out for their unusually long, polyoxygenated carbon backbones featuring numerous stereocenters;^4–6^ polyol derivatives such as the amphidinols or symbiodinolide on the one hand and iconic ladder-polyethers exemplified by brevetoxin, maitotoxin, and ciguoatoxin on the other hand are representative.^2,7–10^ In terms of molecular size and complexity, the SCCCs differ from virtually all secondary metabolites derived from terrestrial organisms known to date;^11^ at the meta level, they fall into the otherwise largely void chemical space in between the domain of small “drug-like” compounds and the realm of the biopolymers. Although their exact physiological role in the marine ecosystem is unknown in practically all cases, many SCCCs are endowed with exceptional biological activities; as such, they demonstrate that “Lipinski’s rule of five” is irrelevant for nature when it comes to evolving compounds of utmost potency.^12,13^

In 2021, Wu and co-workers disclosed benthol A as a new member of this exclusive series (Scheme 1).^14^ The compound was isolated from a previously unknown benthic dinoflagellate (strain MDRC-02) collected in the South China Sea; actually, it is not clear if this organism belongs to a distinct new clade of dinoflagellates or is perhaps just a new member of the *Amphidinium* genus. Benthol A has a net formula of C_75_H_126_O_30_ as confirmed by the molecular mass peak of *m*/*z* 1529.8234 [M + Na^+^]; it features a linear backbone comprised of 72 carbon atoms, which is adorned with three one-carbon branches at C10, C44, and C60, four double bonds (in addition to a branching *exo*-methylene substituent), 22 hydroxy groups, and eight fully saturated oxygenated rings distributed along the chain. These structural attributes result in 35 stereogenic centers in addition to the four stereogenic alkene moieties.^14^ Unlike in most other polyol/polyene SCCCs, however, it is not possible to separate hydrophilic from hydrophobic regions as both the polar and apolar functionalities are scattered over the entire framework; in this regard, the compound is reminiscent of palytoxin, an emblematic and even larger SCCC (2.680 Da, 41 −OH groups), which is derived, however, from an aquatic invertebrate rather than a dinoflagellate.^15^ While the latter is extremely toxic, benthol A was found to exhibit potent antimalaria (EC_50_ 60.8 nM) and appreciable HIV-inhibitory activity, whereas significant cytotoxicity against human cancer cell lines was observed only at concentrations of 100 μM.^14^

**Scheme 1:**
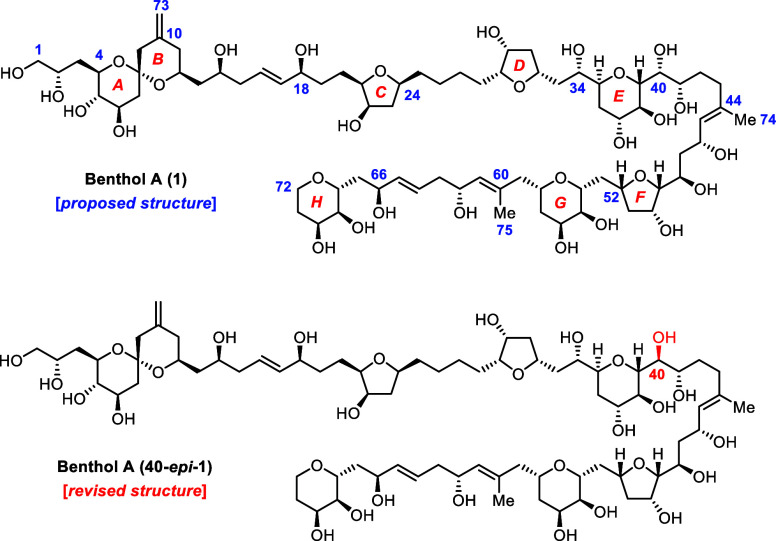
Nominal and Actual Benthol A

The scarcity of SCCCs in general and their noncrystalline nature are major obstacles for structure elucidation, while the vast conformational space populated by such compounds renders spectroscopic studies exceptionally demanding. Despite the massive advances in the analytical toolset during the last decades and a large body of comparative data available to date,^16^ the challenges persist and must not be underestimated. This notion is manifested, for example, in the fact that 20 years of hard work had to pass between the initial disclosure of the structure of amphidinol 3 (1.338 Da; 21 −OH groups) and the firm establishment of the absolute and relative configuration of this compound by total synthesis.^6,17,18^ Along this way, the configuration of no less than eight of the totally 25 stereogenic centers had to be corrected. Similarly, symbiodiolide (2.859 Da, 43 −OH groups) was first published in 2007, but the absolute configuration of two fragments on the backbone (C53–C61; C75–C79) remains unknown to date, despite considerable efforts.^19–21^

When seen against this backdrop, the structure elucidation of benthol A by the Wu group within a fairly short time frame is a remarkable achievement. These authors complemented their extensive spectroscopic efforts, as well as chemical derivatization and degradation experiments, by advanced computational methods. Specifically, DFT-NMR chemical shift calculations were followed by a DP4+ probabilistic analysis in order to assess the match between the computed and experimental spectroscopic data more accurately.^22,23^ This approach was mandatory for the assignment of the configuration of the C40 stereocenter, since severe signal overlap in the NMR spectra prevented the use of any *J*-coupling-based method; attempted Mosher ester formation at this site had also failed. In addition to the shift data, the ^3^ *J* coupling constants in the pertinent region were computed and compared with the experimental values. On the basis of these two lines of evidence, a statistical probability score of 99.93% was reached, which let the authors confidently assign this center as 40*R*-configured (Scheme 1).^14^

Described below and in the accompanying paper is the first total synthesis of benthol A. During the course of this project as part of our long-standing commitment to natural products derived from dinoflagellates,^24^ the analysis of several building blocks forecasted that the structure assigned to this remarkable marine natural product might not be entirely correct and some revision was needed. While the data initially pointed to a possible mistake at or close to the tetrahydropyran E-ring, it took considerable effort to narrow down the site of misassignment. In the end, the C40 position was identified as the most likely cause of error despite the apparently overwhelming evidence presented by the isolation team. On the basis of this anticipation, we embarked on the total synthesis of *both* diastereomeric C40-OH derivatives **1** and 40-*epi*-**1** of this sizable target compound. The effort was rewarded in that it allowed us to prove beyond any doubt that actual benthol A is 40*S*- rather than 40*R*-configured, as had originally been proposed.

## Results and Discussion

### Retrosynthetic Analysis and Strategic Considerations

Given the size and complexity of the target compound, it was mandatory to ensure high convergency while retaining flexibility during the assembly stage. In addition, the choice of the protecting groups for the no fewer than 22 −OH substituents on the backbone had to be carefully considered and their variance kept as small as possible because the sheer number of events necessary for the global deprotection of the target has a massive impact on the overall efficiency of the chosen route. Furthermore, fragment unions at or near the sites of unsaturation were deemed more favorable than those involving the formation of a stereogenic center, not least because the latter entail greater analytical effort to prove the correct stereochemical course of a given coupling event.

With these general considerations in mind, the retrosynthetic cuts at the C17–C18 and the C43–C44 bonds are fairly obvious, not least because the resulting three major building blocks **A**, **B**, and **C** are of fairly similar size and complexity (Scheme 2). Their further simplification, however, was less evident. As far as fragment **A** is concerned, we chose to unfold the conspicuous [6,6]-spiroacetal moiety to a dihydroxylated alkyne derivative **A1**: chemoselective activation by catalytic amounts of a carbophilic Lewis acid such as Au­(+1) or Pt­(+2) renders the triple bond susceptible to nucleophilic attack by the appropriately placed −OH groups,^25^ which eventually results in spiroacetal formation.^26–30^ At the same time, an alkyne greatly facilitates the assembly of the building block from two readily accessible fragments of type **A2** and **A3**.

**Scheme 2:**
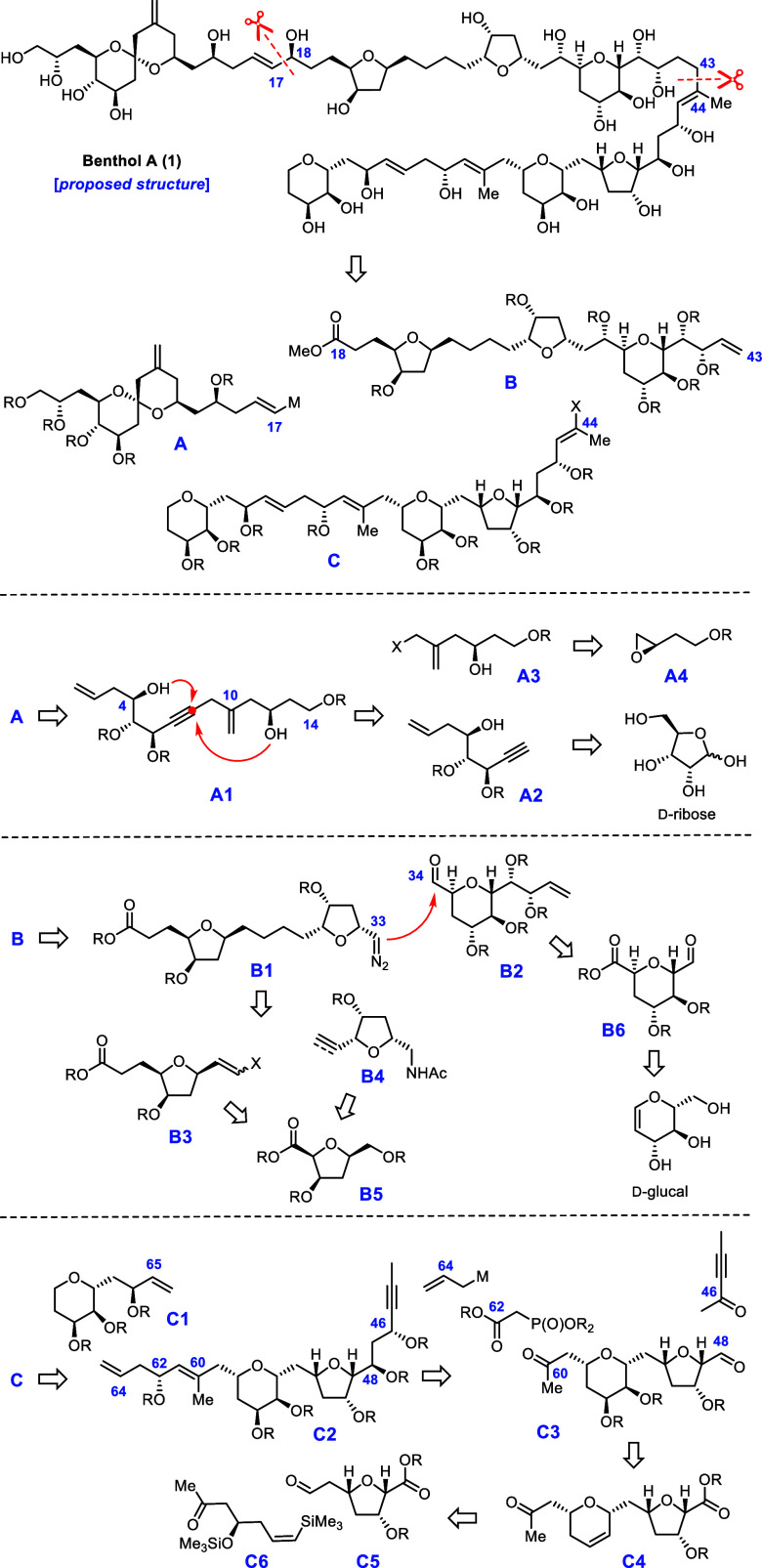
Retrosynthetic Analysis of Nominal Benthol A (**1**)

We had already pursued similar strategies in previous total syntheses,^31,32^ not least those of the dinoflagellate-derived compounds limaol^33,34^ and prorocentin;^35,36^ the envisaged application to benthol A, however, poses additional challenges. Specifically, the gold-catalyzed spiroacetalization reactions leading to the limaol and the prorocentin core structures were accompanied by the swift and quantitative migration of an *exo*-methylene group into an endocyclic position of the newly formed rings (Scheme 3).^35^ In the case of benthol A, however, such a rearrangement must be strictly avoided, and the *exo*-methylene group at the C10 position of the spiroacetal B-ring must be faithfully retained. At the same time, control over the site of attack of the lateral −OH groups and, hence, the size of the newly formed acetal rings is arguably more difficult to exert in the projected than in the documented cases: for limaol as well as prorocentin, the triple bond to be activated had no polar substituents in the vicinity; in the conversion **A1** → **A**, however, the (protected) propargylic hydroxy substituent polarizes the π-bond; in addition, it could engage the attacking −OH groups in hydrogen bonding and/or might precoordinate the incoming catalyst; each of these effects could bias the ketalization step in a way that is difficult to predict.^37^ This uncertainty notwithstanding, we could not help but try to push π-acid catalysis to a new level by this advanced application as part of the benthol A campaign.

**Scheme 3:**
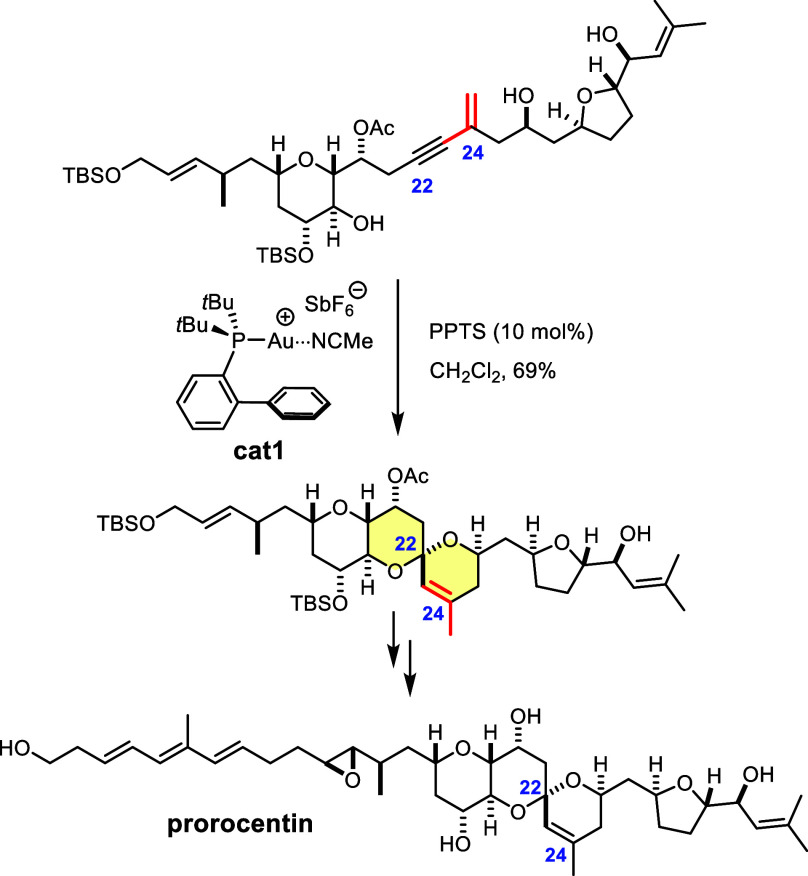
Gold-Catalyzed Spiroacetalization with Concomitant Migration of an *Exo*-Methylene Group into an *Endocyclic* Position as a Key Step of the Total Synthesis of Prorocentin

The central fragment **B** was further broken down based on symmetry considerations. Formal retrosynthetic cleavage at the stereogenic C33–C34 bond unravels building block **B1**, which can be thought of as consisting of two almost identical THF entities differing in their termini. They can be traced back to a common intermediate **B5**, which will be used twice, thus reducing the synthetic burden as well as the total step count. The second required component **B2** should be accessible by *syn*-selective oxy-allylation from aldehyde **B6**, which in turn may derive from d-glucal as a convenient starting material.

Less obvious, perhaps, is the way the fragments **B1** and **B2** can be joined without opening one or the other cyclic ether. We suspected that a terminal diazo derivative as a “masked carbene” might qualify for this purpose, even though the stability and reactivity of a compound of type **B1** could not be predicted with any degree of certainty. The conversion of simple aldehydes to homologated ketones on reaction with diazoalkanes is long known (the “Buchner–Curtius–Schlotterbeck reaction”), though not overly widely used since the reaction is often plagued by side product formation, most notably epoxides or compounds with rearranged carbon skeletons.^38–41^ The course is usually more reliable when (semi)­stabilized diazo derivatives such as diazoesters, trimethylsilyldiazomethane, or aryldiazomethane derivatives are used in the presence of a proper promoter.^42–44^ The intended application to fragment union has, hence, hardly any precedent at the projected level of complexity.^45^ Under the premises, however, that (i) the tetrahydropyran ring of **B2** (essentially) adopts a chair conformation, (ii) the endocyclic oxygen atom α to the aldehyde entails a polar Felkin–Anh-type scenario,^46,47^ and (iii) the diazo derivative **B1** attacks in an ylide-like manner known to favor an “eclipsed” orientation of the zwitterionic/polar groups,^48^ one can envisage that an adduct of type **D** is preferentially formed in the first place (Scheme 4). In such an intermediate, the H atom of the aldehyde, which has to migrate, is antiperiplanar to the leaving group; the favorable orbital overlap should entail the migratory aptitude it takes to generate the homologated ketone corresponding to the targeted building block **B** with high selectivity.

**Scheme 4:**
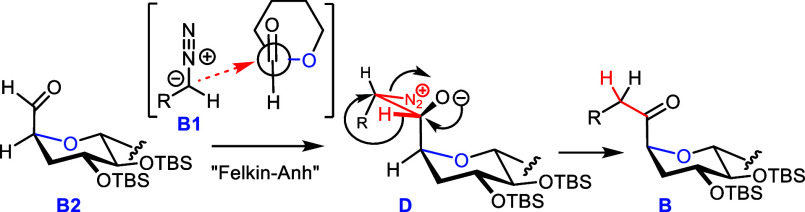
Forecast Why the Projected Subfragment Coupling by a Buchner–Curtius–Schlotterbeck Reaction Might Take the Desired Course

Fragment **C,** as the third large building block, was thought to derive from alkene **C1** by cross-metathesis with **C2** and chemo-orthogonal carbometalation/iodination of the methyl-capped alkyne, forming the other terminus. Fragment **C2**, in turn, leads back to a precursor of type **C3** by standard olefination/allylation and aldol chemistry, although the exact order of events had to be defined during implementation. We conjectured that the core could be assembled by linking the pyran G-ring in one step to an aldehyde **C5** via an intramolecular silyl-modified Sakurai (ISMS) reaction with the functionalized alkenylsilane **C6** as a reaction partner,^49–51^ followed by dihydroxylation of the double bond in fragment **C4** thus formed (for details, see the accompanying paper).

This blueprint was thought to meet the criteria of flexibility and multiple convergence. As such, it should also allow accommodation of structural amendments if constitutional and/or stereochemical revisions were necessary en route to the target compound, which in the end proved to be the case.

### The Spiroacetal Fragment

Even though d-ribose does not exactly map onto a building block of type **A2**, this cheap starting material proved to be a convenient entry point. The reason for this is that simple isopropylidene acetal formation renders the *cis*-orientation of the two branches on the five-membered acetal ring of **2** thermodynamically unfavorable and hence allows the stereogenic center α to the aldehyde (masked as hemiketal) to be epimerized with ease.^52^ When the Bestmann–Ohira reagent **6** ^53^ is used for chain extension, which mandates basic yet protic conditions, quantitative epimerization precedes alkyne formation (Scheme 5). The structure of the resulting compound **3** ^54^ in the solid state proves that the triple bond branching off C6 (benthol numbering scheme) is indeed *trans* to the hydroxylated substituent at the vicinal C5 position on the isopropylidene acetal ring (Figure 1); compound **3**, hence, shows the exact configuration needed for the projected synthesis of the benthol A spiroacetal building block.

**Scheme 5:**
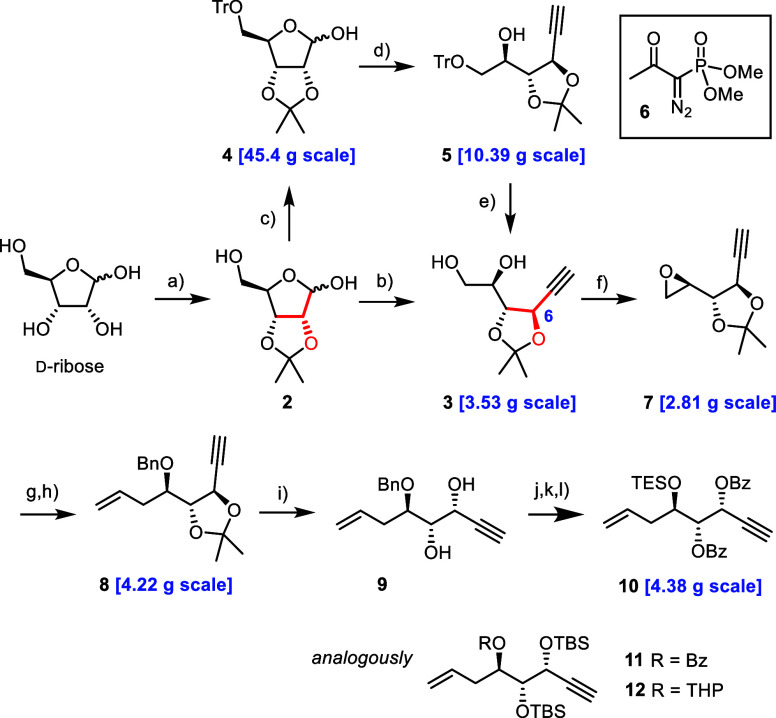
Reagents and Conditions: (a) Acetone, H_2_SO_4_ (8 mol %), RT, Quant.; (b) **6**, K_2_CO_3_, MeOH, Reflux, 47%; (c) Trityl Chloride, DMAO (10 mol %), Et_3_N, DMF, RT, 77%; (d) **6** (Slow Addition), K_2_CO_3_, MeOH, Reflux, 83%; (e) NaHSO_4_/Silica (10% *w*/*w*), CH_2_Cl_2_/MeOH (9:1), RT, 82%; (f) (i) TsCl, Bu_2_SnO (10 mol %), *i*Pr_2_NEt, CH_2_Cl_2_, RT; (ii) DBU, RT, 88%; (g) Vinylmagnesium Bromide, CuI (20 mol %), THF, −78 °C, 98% [3.24 g Scale]; (h) BnBr, NaH, DMF, 0 °C to RT, 89%; (i) HOAc/H_2_O (4:1), 65 °C; (j) Benzoyl Chloride, Pyridine, 0 °C to RT, 93% [6.20 g Scale] (Over Two Steps); (k) DDQ, hν (Blue LED), CH_2_Cl_2_/H_2_O (99:1), RT, 76% [3.79 g Scale]; (l) TESOTf, 2,6-Lutidine, CH_2_Cl_2_, 0 °C, 88%; the Scales Indicated in This and the Following Schemes Refer to the Amount of Product in the Single Largest Batch

**Figure 1:**
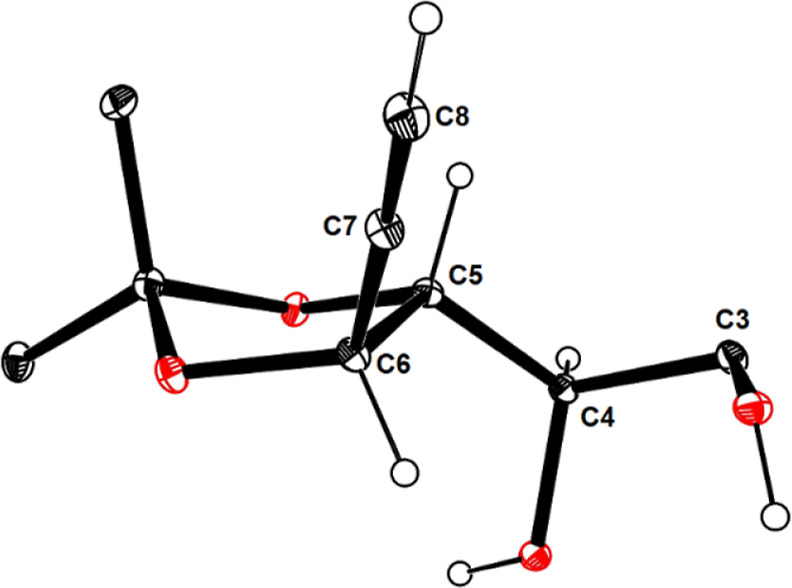
Structure of alkyne **3** in the solid state (H atoms partly removed for clarity); benthol numbering scheme.

The high water-solubility of this compound, however, makes the direct conversion of **2** into **3** cumbersome. As a consequence, this is a case in which a detour pays valuable dividends in practical terms; thus, it proved advantageous to protect the primary −OH group of **2** as the lipophilic trityl ether **4** prior to alkyne formation and then cleave this group off on contact of **5** with NaHSO_4_ adsorbed on silica.^55,56^ This apparent detour worked well on a (multi)­decagram scale. The resulting diol **3** was converted into epoxide **7** upon selective tosylation of the primary −OH group in the presence of catalytic amounts of Bu_2_SnO^57^ followed by ring closure on treatment with DBU. As expected, the opening of **7** by vinylmagnesium bromide in the presence of catalytic CuI at low temperature proceeded regioselectively in an almost quantitative yield. The derived product **8** was then diverted into a few differently protected enynes (**10**–**12**), as it was not clear at the outset if and how the substitution pattern impacts the projected π-acid-catalyzed spiroacetalization reaction; key to success was the photochemically driven oxidative debenzylation, which is chemo-orthogonal to the π-bonds of these building blocks.^58^

The required coupling partner was derived from l-aspartic acid, which was first converted by a literature route into epoxide **14** (Scheme 6).^59^ Once again, a copper-catalyzed ring opening with the functionalized Grignard reagent **15** proceeded cleanly.^60^ After some experimentation, we found that the resulting product **16** could be directly converted on a multigram scale into the desired allyl bromide **17** on treatment with NBS in THF at low temperature without the need to temporarily protect the –OH group or scavenge the released TMSBr.^61^ Copper-catalyzed coupling with the terminal alkynes **10**–**12**, promoted by a weak inorganic base in combination with catalytic DBU, gave an appropriate set of substrates for the envisaged spiroacetalization.^62^

**Scheme 6:**
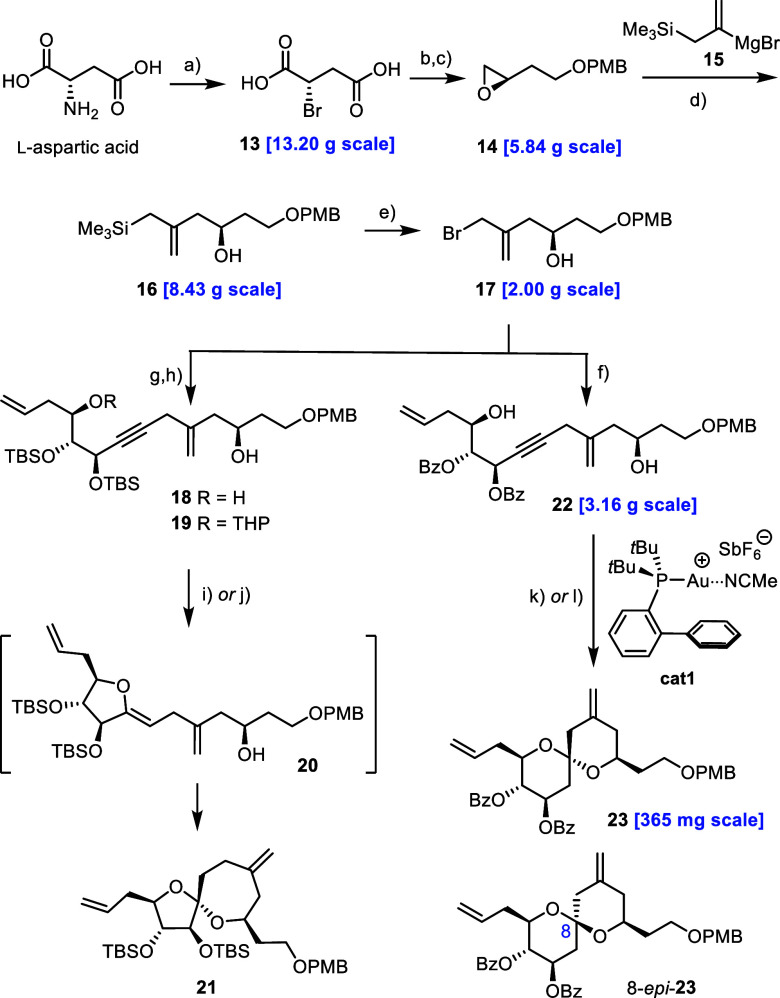
Reagents and Conditions: (a) NaNO_2_, KBr, H_2_SO_4_ Cat., H_2_O, 0–10 °C, 88%; (b) BH_3_·THF, THF, 0 °C, 94% [5.28 g Scale]; (c) NaH, THF, −5 °C, Then PMBBr, 89%; (d) **15**, CuI (15 mol %), THF, −50 °C, 93%; (e) NBS, THF, −78 °C, 85%; (f) (i) **10**, CuI (25 mol %), DBU (10 mol %), Na_2_SO_3_, K_2_CO_3_, DMF, RT; (ii) CSA (10 mol %), CH_2_Cl_2_/MeOH (4:1), RT, 82%; (g) **11**, CuI (10 mol %), K_2_CO_3_, Na_2_SO_3_, DBU (30 mol %), DMF, RT, 94%; (h) Dibal-H, CH_2_Cl_2_, −78 °C, THF, 97% (**18**); (i) **cat1** (10 mol %), CSA (20 mol %), CH_2_Cl_2_, −78 °C, Then RT, 80% (from **18** (R = H)); (j) **cat1** (20 mol %), PPTS (20 mol %), CH_2_Cl_2_, −50 °C to RT, 70% (from **19** (R = THP)); (k) **cat1** (20 mol %), CSA (10 mol %), CH_2_Cl_2_, −10 °C, 50% (**23** + 8-*epi*-**23**, dr ≈ 1.5:1); (l) **cat1** (2 mol %), CSA (10 mol %), CH_2_Cl_2_, −78 °C, Then RT, 76% (**23**) + 9% (8-*epi*-**23**)

It was not entirely unexpected that this first key step required considerable attention. First, we noticed that substrate **18** endowed with TBS-ethers, on activation with the gold complex **cat1** (20 mol %)^63^ and cocatalytic Brønsted acid (PPTS, camphorsulfonic acid), invariably and almost exclusively furnished the undesired [5,7]-spiroacetal **21**. As suggested by our previous results,^33–35^ the reaction likely proceeds in two distinct steps: the gold catalyst activates the π-bond for initial attack by one of the –OH groups to first form an enol ether, which is then activated by the protic acid to give a transient oxocarbenium ion that intercepts the second –OH group to yield the final acetal product.^64^ In the present case, the C4-OH group (benthol numbering scheme) is primarily accountable for the initial attack onto the triple bond in a kinetically favorable 5-*exo-dig* manner to give compound **20** as the primary product; this triggering event, however, determines the wrong regiochemical course.

In an attempt to rectify the outcome, the C4-OH group was protected as THP acetal. Cleavage of this group in the acidic medium might be sufficiently slow to give the C12-OH substituent the chance to react with the alkyne in the first place.^65^ This hope did not come true, because substrate **19** also gave the [5,7]-spiroacetal **21** as virtually the only detectable product. A switch of the carbophilic catalyst from Au­(+1) to Hg­(+2) or Pt­(+2) was to no avail either as complex mixtures were attained,^66,67^ but the change of the protecting group was rewarded with success. This move was based on the perception that a more electron-withdrawing substituent might favorably polarize the π-system, imposing a slightly higher positive charge onto the C atom distal to this propargylic substituent. At the same time, weak coordination of the ester carbonyl onto the incoming [LAu]^+^ fragment could steer auration at the C atom next to the benzoate; under this premise, the lateral −OH groups attack the C atom distal to the propargylic substituent, which ultimately delivers the desired [6,6]-spiroacetal.^68^ Indeed, a first hit was observed when the reaction was performed with compound **22** bearing benzoate groups at −10 °C in THF using a high loading of the gold catalyst; under these conditions, the [6,6]-spiroacetal was obtained as a 1.5:1 mixture of the anomers **23** and 8-*epi*-**23**. Further optimization allowed this result to be notably improved: the best outcome was obtained by lowering the loading of **cat1** to 2 mol % and applying a temperature ramp from −78 °C to ambient temperature.^69^ Under these conditions, the desired product **23** was obtained in a well-reproducible 76% yield, together with 9% of 8-*epi*-**23**, which could be separated by flash chromatography.

A subsequent platinum-catalyzed asymmetric diborylation/oxidation sequence allowed us to convert the terminal double bond of **23** into the required *vic*-diol **24** with appreciable diastereoselectivity (dr ≥ 10:1) while leaving the *exo*-methylene group untouched (Scheme 7).^70–73^ Following conventional protecting group and oxidation state management, the derived aldehyde **25** was reacted with the α-stannylallylborane reagent **27** generated in situ from allenyl­(tributyl)­stannane (**26**)^74^ and (−)-Ipc_2_BH (derived from (+)-α-pinene)^75,76^ to furnish the targeted stannylated homoallylalcohol derivative **28** virtually as a single diastereomer.^77^ This compound represents an adequate incarnation of building block **A** in readiness for fragment coupling.^78^

**Scheme 7:**
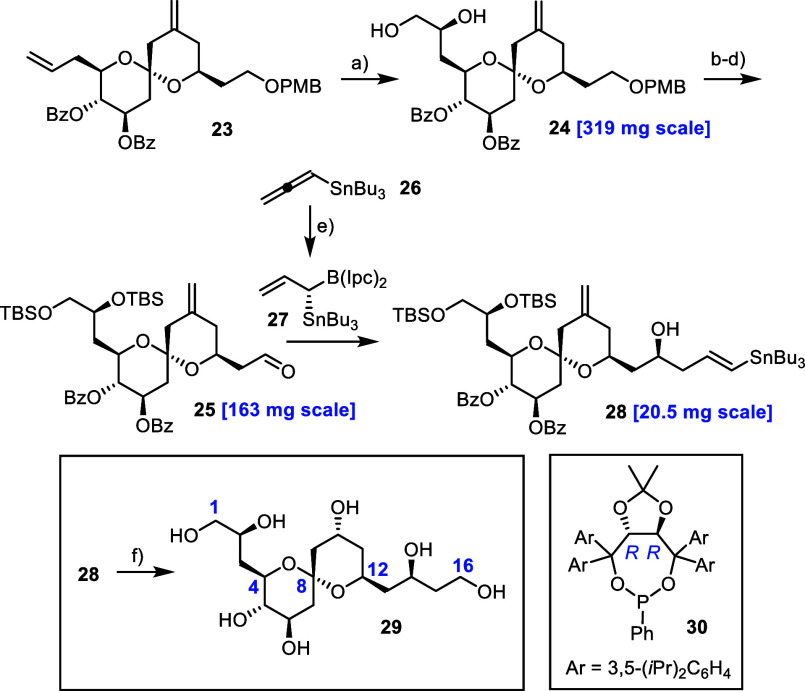
Reagents and Conditions: (a) Pt­(dba)_3_ (5 mol %), (*R*,*R*)-**30** (7.5 mol %), B_2_(pin)_2_, THF, Reflux, Then NaBO_3_·H_2_O, H_2_O, 81% (dr ≥ 10:1); (b) TBSOTf, 2,6-Lutidine, CH_2_Cl_2_, 0 °C; (c) DDQ, CH_2_Cl_2_/H_2_O (20:1), RT, 84% (over Two Steps); (d) Dess-Martin Periodinane, NaHCO_3_, CH_2_Cl_2_, RT, 90%; (e) **26**, (−)-Ipc_2_BH, Et_2_O, Then **25**, −78 °C, 71%; (f) (i) K_2_CO_3_, MeOH, RT; (ii) TBAF, THF, 0 °C to RT, Then DOWEX, 68% (over Two Steps); (iii) O_3_, CH_2_Cl_2_/MeOH (1:1), −78 °C, Then NaBH_4_

### First Substructure Verification

Owing to heavy signal overlap in the NMR spectra, the isolation team had tried to simplify their task by subjecting benthol A to ozonolysis followed by a reductive workup with NaBH_4_; this afforded five degradation products, which were then analyzed separately.^14^ As one of them could be reached starting from fragment **28**, we saw a valuable opportunity for substructure verification.

This goal was attained upon global deprotection of fragment **28**, followed by ozonolytic cleavage of both double bonds and reductive workup according to the literature conditions. Interestingly, the borohydride reduction of the ketone derived from the *exo*-methylene group branching off C10 proceeded stereoselectively within the limits of detection. In any case, the ^13^C NMR spectrum of the resulting product **29** perfectly matched the reported data (Δδ_C_ ≤ 0.05 ppm for all 16 C atoms; for details, see the Supporting Information), thus proving identity between the spiroacetal unit of our synthetic material and the westernmost C1–C16 spiroacetal sector of the natural product.

### The Central Segment

As mentioned above, the central fragment **B** was envisaged to derive from two subsegments **B1** and **B2** to be joined by an arguably challenging Buchner–Curtius–Schlotterbeck reaction. The larger of the two should be accessible by using a common building block of the type **B5** twice. This compound had, hence, to be made in substantial quantities, requiring a robust route.

To this end, the commercially available epoxide **31** was chosen as the point of departure, which was opened with vinylmagnesium bromide under copper catalysis (Scheme 8); the resulting crude product **32** was pure enough for use in the subsequent cross-metathesis (CM) with methyl acrylate,^79,80^ which furnished the desired *E*-configured product almost exclusively (98% over both steps, *E*/*Z* = 28:1). After converting the remaining hydroxy group into the corresponding mesylate, compound **33** was subjected to asymmetric dihydroxylation^81^ immediately followed by treatment of the diol **34** thus formed with 2,6-lutidine to cause ether formation by an intramolecular S_N_2 reaction.^82^ This one-pot operation gave decagram quantities of the desired common building block **35** after silylation of the remaining hydroxy group; small amounts of diastereomers originating from the imperfect selectivity at the CM and the dihydroxylation stages were separated at this point. The structure of compound **35** was confirmed by crystallographic means after debenzylation (for details, see the Supporting Information).

**Scheme 8:**
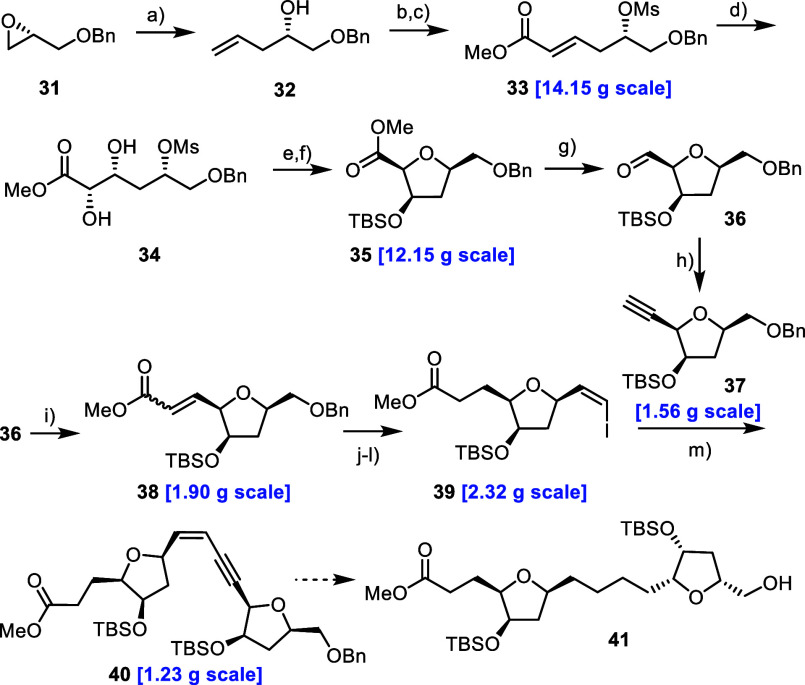
Reagents and Conditions: (a) Vinylmagnesium Bromide, CuI (20 mol %), THF, −78–0 °C; (b) Methyl Acrylate, Grubbs II Catalyst (2 × 1 mol %), CH_2_Cl_2_, Reflux, 98% (over Two steps; *E*/*Z* = 28:1 [10.90 g Scale]); (c) MsCl, Et_3_N, CH_2_Cl_2_, 0 °C, 99%; (d) K_2_OsO_2_(OH)_4_ (2 mol %), (DHQD)_2_PHAL (4 mol %), K_3_Fe­(CN)_6_, K_2_CO_3_, MeSO_2_NH_2_, *t*BuOH, H_2_O, CH_2_Cl_2_, 0 °C to RT; (e) 2,6-Lutidine, 120 °C, 80% [8.48 g Scale] (over Two Steps; +10% of Isomers); (f) TBSCl, DMAP Cat., Imidazole, DMF, RT, Quant.; (g) Dibal-H, CH_2_Cl_2_, −78 °C; (h) **6**, K_2_CO_3_, MeOH, 0 °C to RT, 90% (over Two Steps); (i) Ph_3_P = CHCOOMe, CH_2_Cl_2_, 0 °C to RT, 94% (over Two Steps); (j) H_2_ (1 atm), Pd­(OH)_2_/C, EtOAc, RT, 94%; (k) DMSO, (CO)_2_Cl_2_, CH_2_Cl_2_, Then Et_3_N, −78–0 °C; (l) [Ph_3_PCH_2_I]­I, KHMDS, HMPA, THF, −78–0 °C, 85% (over Two Steps); (m) [(Ph_3_P)_2_PdCl_2_] (10 mol %), CuI (20 mol %), Et_3_N, RT, 93%

Aldehyde **36**, derived from ester **35** by Dibal-H reduction, was diverted into alkyne **37** and enoate **38**; the latter was hydrogenated using Pd­(OH)_2_/C^83^ to saturate the double bond and, concomitantly, cleave the benzyl ether. Subsequent Swern oxidation^84^ followed by a Stork/Zhao olefination^85^ provided alkenyl iodide **39** with excellent isomeric purity. A high-yielding Sonogashira reaction mandating Et_3_N as the solvent allowed these two fragments to be coupled.^86^ With enyne **40** in hand, a simple hydrogenation/hydrogenolysis was expected to furnish the desired building block, representing **B1**. This seemingly trivial step, however, proved to be surprisingly troublesome and unreliable. After considerable but largely unsuccessful experimentation, we finally opted to skip the enyne altogether; rather, we resorted to a B-alkyl Suzuki coupling of alkenyl iodide **39** with alkene **42**,^87,88^ which also derives from the common building block **36** (Scheme 9). Strikingly, even the hydrogenation of the resulting product **43** over either Pd­(OH)_2_/C^83^ or commercial Pd/C was not straightforward either, facing significant reproducibility issues. An unexpected byproduct formed by the opening of one of the THF rings was isolated in an appreciable but somewhat variable quantity; its structure was unambiguously assigned as **44** after per-silylation. Although no further mechanistic study was performed, this compound suggested that an active palladium species inserts into the allyl ether bond of **43**; the resulting allylpalladium intermediate then gets reduced, even though the literature knows of very little precedent for this.^89,90^ We conjectured that the use of a catalyst other than Pd(0) might allow this issue to be circumvented. As expected, Raney-Ni furnished **45** without incident but failed to remove the benzyl ether. This saturation of the allylic double bond, however, should preclude ether cleavage; as expected, hydrogenolysis of **45** over Pd­(OH)_2_/C cleanly afforded the desired product **41**. Although the seemingly trivial conversion of **43** into **41** hence mandates two separate steps, the high overall yield and procedural robustness justify this maneuvering.^91^ Compound **41** was then elaborated via azide **46** into the *N*-nitrosamide derivative **47** as a storable surrogate of the highly unstable diazo derivative needed for the projected coupling with fragment **B2**.

**Scheme 9:**
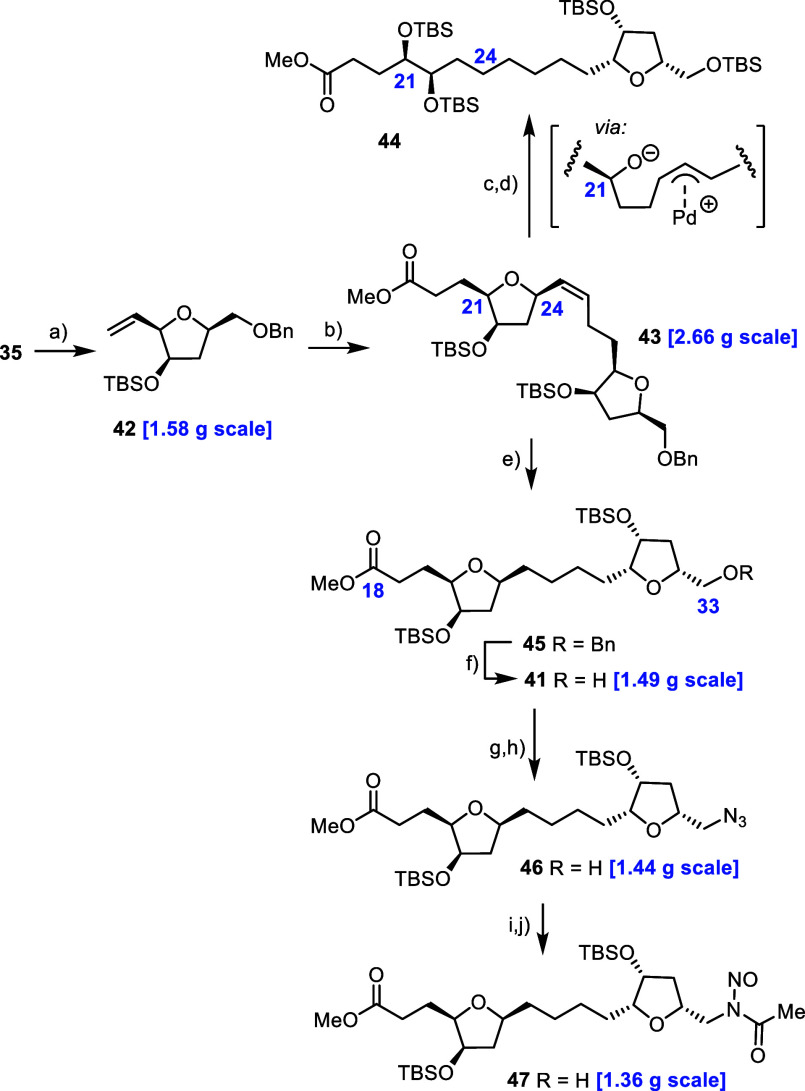
Reagents and Conditions: (a) (i) Dibal-H, CH_2_Cl_2_, −78 °C; (ii) Ph_3_P = CH_2_, THF, 0 °C, 91% (over Two Steps); (b) (9-H-9-BBN)_2_, THF, Then **39**, [(dppf)­PdCl_2_] (10 mol %), Ph_3_As (40 mol %), Cs_2_CO_3_, DMF/THF/H_2_O, RT, 96%; (c) H_2_ (1 atm), Pd­(OH)_2_/C, EtOAc, RT, ; (d) TBSCl, DMAP Cat., Imidazole, DMF, RT, up to 40% (See Text); (e) H_2_ (1 atm), Raney-Ni, EtOAc, RT; (f) H_2_ (1 atm), Pd­(OH)_2_/C, EtOAc, RT, 84–94% (over Two Steps); (g) MsCl, Et_3_N, CH_2_Cl_2_, 0 °C; (h) NaN_3_, DMF, 100 °C, 79% (over Two Steps); (i) H_2_ (1 atm), Pd/C, Ac_2_O, MeOH, 0 °C to RT, 90% [1.27 g Scale]; (j) NaNO_2_, Ac_2_O, HOAc, 0 °C, 84%

The preparation of the **B2**-segment started off by the replacement of the ester groups in commercial tri-*O*-acetyl-d-glucal by TBS-ethers (Scheme 10).^92^ Addition of anhydrous HCl in dioxane to product **48** thus formed furnished the corresponding glycosyl chloride **49**, which is very sensitive and must be dried and handled with great care.^93^ The subsequent metal/halogen exchange with lithium naphthalenide at low temperature, followed by trapping of the anomeric organolithium species with Mander’s reagent and selective cleavage of the primary –OTBS ether, furnished the corresponding primary alcohol **50** in an appreciable 55% yield over three steps on a 1.2 g scale. The derived crude aldehyde was subjected to a *syn*-selective oxy-allylation reaction.^94^ To this end, the MOM-ether **51** was deprotonated with *sec*-BuLi, and the resulting organolithium species was trapped with Ipc_2_BOMe to give a presumably (*Z*)-configured allylboron intermediate, which adds via the usual six-membered transition state to afford the *syn*-configured, monoprotected 1,2-diol **52** with excellent diastereoselectivity (dr > 20:1). Surprisingly, this addition turned out to be under strict substrate-rather than reagent control as (+)-Ipc_2_BOMe and (−)-Ipc_2_OMe led to the exact same stereochemical outcome, with the latter being higher yielding (for details, see the Supporting Information).

**Scheme 10:**
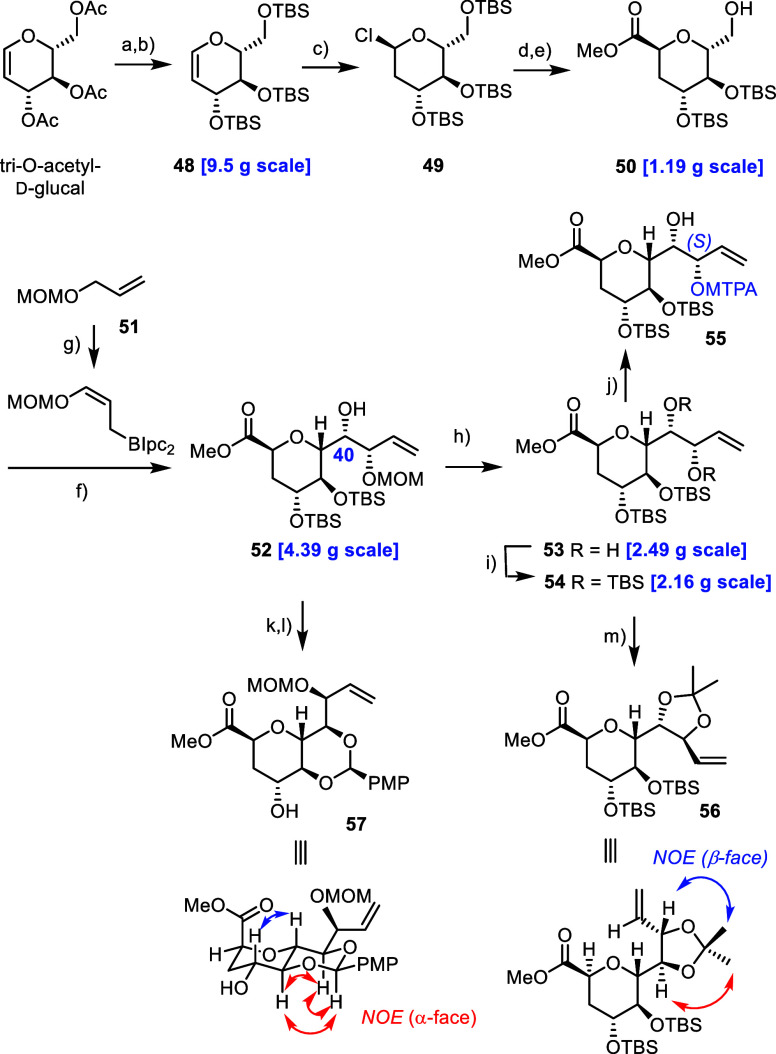
Reagents and Conditions: (a) K_2_CO_3_, MeOH, RT; (b) TBSCl, Imidazole, DMF, 0 °C to RT, 97% (over Two Steps); (c) HCl in 1,4-Dioxane, THF, 0 °C to RT; (d) Lithium Naphthalenide, THF, −95 °C, Then MeOC­(O)­CN, −95 °C to RT; (e) PTSA·H_2_O Cat., MeOH, 0 °C, 55% (over Three Steps); (f) Dess-Martin Periodinane, NaHCO_3_, CH_2_Cl_2_, 0 °C to RT; (g) **51**, *sec*-BuLi, (−)-Ipc_2_BOMe (See Text), BF_3_·OEt_2_, THF, −95 °C to RT, Then aq. H_2_O_2_, NaHCO_3_, 0 °C to RT, 82% (over Two Steps); (h) Me_3_SiBr, CH_2_Cl_2_, −78 °C to −10 °C, 62%; (i) TBSOTf, 2,6-Lutidine, CH_2_Cl_2_, 0 °C to RT, Quant.; (j) (*S*)-(+)- or (*R*)-(−)-α-Methoxy-α-(trifluoromethyl)­phenylacetyl Chloride, Pyridine, DMAP Cat., CH_2_Cl_2_, RT, 69–72%; (k) TBAF, THF, RT, 84%; (l) MeOC_6_H_4_CH­(OMe)_2_, PTSA·H_2_O Cat., THF, RT, Quant.; (m) 2,2-Dimethoxypropane, PPTS Cat., RT, 80%

In an attempt to confirm the absolute configuration of the newly formed stereogenic center, we tried to convert the secondary –OH group into the corresponding Mosher ester derivatives but failed. This fact is reminiscent of the isolation team’s inability to derivatize benthol A or its degradation products at the corresponding C40 position.^14^ Therefore, we had to resort to another chain of evidence: to this end, the MOM-ether was cleaved, and the configuration of the sterically less encumbered C41-OH group in diol **53** was determined by the modified Mosher method (**55**).^95^ In parallel, compounds **52** and **53** were converted into the cyclic acetals **57** and **56**, respectively; the recorded NOE data provided firm proof of the relative configuration between the −OR substituents at the C38, C39, C40, and C41 positions. Taken together, these data show beyond doubt that compound **54** perfectly corresponds to the proposed configuration about the pyran E-ring of benthol A.^14^

The aldehyde **58** derived from ester **54** by Dibal-H reduction is, hence, a fully functional and properly configured building block for the assembly of the central fragment (Scheme 11). We were pleased to find that the projected Buchner–Curtius–Schlotterbeck reaction between the diazo derivative **59** generated in situ from nitrosamine **47** on treatment with aq. KOH and this aldehyde worked remarkably well, despite all the challenges and issues that one might conceive in a reaction involving such an unstabilized yet densely functionalized masked “carbene” intermediate. Therefore, we trust that the rationale sketched in Scheme 4 captures the essence. In any event, the desired ketone **60** was isolated in 65% yield in a reaction furnishing no less than 1.46 g of product. The subsequent carbonyl reduction to complete the preparation of an adequate fragment **B** equivalent was best performed with L-selectride at low temperature; although the dr in favor of the required (*S*)-configured C34-OH derivative 34*S*-**61** was only 2.9:1, various other reducing agents gave even less favorable ratios; moreover, the undesired isomer 34*R*-**61** can be readily separated at this stage. For both diastereomers, a full Mosher ester analysis was carried out to exclude any doubt as to the configuration of the newly set secondary –OH group.

**Scheme 11:**
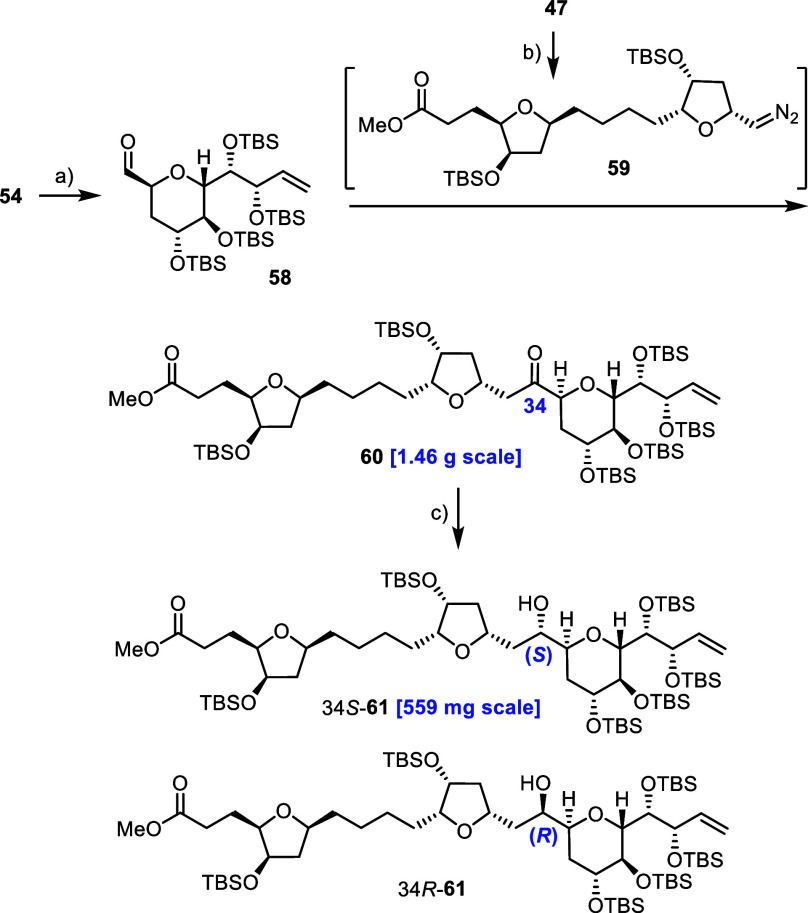
Reagents and Conditions: (a) Dibal-H, CH_2_Cl_2_, −78 °C; (b) aq. KOH, Toluene/MeOH, 0 °C, Then MgSO_4_; Then **58**, Toluene, 0 °C to RT, 65%; (c) l-Selectride, THF, −78 °C, 65% (34*S*-**61**), 23% (34*R*-**61**)

### A Massive Alert

From a substructure verification point of view, the availability of both C34 epimers with rigorously defined configurations was actually deemed advantageous. Therefore, both compounds were reduced with Dibal-H, and the resulting crude products were globally deprotected to give the polyol derivatives 34*S*-**62** and 34*R*-**62**, respectively (Scheme 12).

**Scheme 12:**
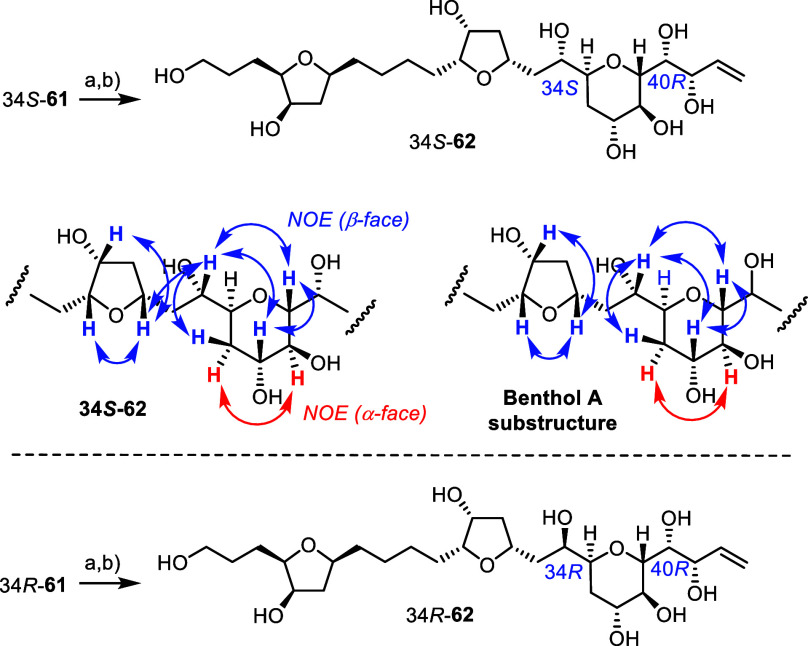
Reagents and Conditions: (a) Dibal-H, CH_2_Cl_2_, −78 °C to RT; (b) TBAF, THF; RT, 77% (34*S*-**62**), 93% (34*R*-**62**); Comparison of the NOEs Observed for 34*S*-**62** and the Corresponding Subsection of Authentic Benthol A

Very much to our surprise, the ^13^C NMR spectra of neither of the two mapped well onto the shifts recorded for this section of benthol A (Figure 2).^14^ The termini apart, which are necessarily off, notable differences of up to several ppm were observed for virtually all positions from C31 to C40. For the scatter, one cannot even decide with any degree of confidence whether the 34*R* or the 34*S* isomer is better fitting. It was hence totally unclear whether or not one or even several centers in this sector might have been misassigned by the isolation team and, if so, which one(s).

**Figure 2:**
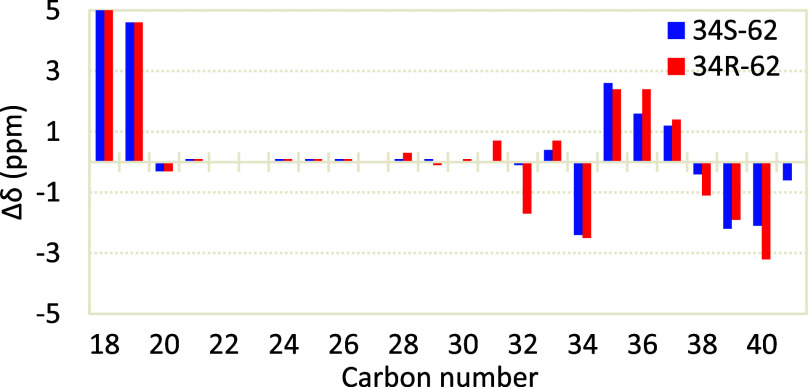
Graphical representation of the ^13^C NMR chemical shift differences (Δδ_C_, ppm; [D_4_]-MeOH) between both epimers of compound **62** and authentic benthol A as reported in the literature.

Given the excellent match observed for the spiroacetal fragment (see above), these significant deviations were worrisome, mandating closer inspection. First, a look at the sizable number of NOEs recorded for 34*S*-**62** showed them to be fully consistent with the pattern observed by the isolation team for the natural product;^14^ in contrast, massive signal overlap in the spectra prevented any such analysis for 34*R*-**62**. Although this clearly different fingerprint cannot be taken as rigorous proof, it suggests that the absolute and relative configurations assigned to the C34 stereogenic center and its vicinity might be correct.

A close look at the coupling constants provided additional hints. Thus, the pertinent ^3^ *J* values along the periphery of the E-ring of a degradation product derived from natural benthol A are all notably different from those of compound 34*S*-**61**, representing fragment **B**. Arguably most striking is the mismatch of ^3^ *J*_H39,H40_, which is small in benthol A (1.2 Hz)^14^ but large in the synthetic segment (7.8 Hz). These latter data points also cast serious doubts on whether the calculated coupling constants used by the isolation team as a supporting argument for assigning the 40*R* configuration to benthol A are accurate and meaningful: in any case, the fairly small computed ^3^ *J*_H39,H40_ = 3.41 Hz is significantly off the 7.8 Hz observed for 34*S*-**61**, which is undoubtedly 40*R* configured.

For this mismatch, a comparison with an appropriate 40*S*-configured fragment was deemed critically important. To this end, the small subsegment **52** was subjected to a standard Mitsunobu reaction, which, however, did not result in any conversion;^96^ this lack of reactivity mirrors the failed attempt at forming a Mosher ester at this site.^14^ The massive steric hindrance also surfaced in a number of attempts at stereoselective reduction of the derived ketone **63**: while bulky L-Selectride left the carbonyl group untouched, Zn­(BH_4_)_2_ as well as a CBS reduction solely regenerated the 40*R* isomer **52**. Only NaBH_4_ in EtOH furnished a small amount (ca. 19%) of the inverted, 40*S*-configured isomer **64** (in addition to **52** as the major product), which sufficed for our purpose at this point (Scheme 13). Compounds **52** and **64** were then separately transformed into the reduced and fully deprotected polyol **65** and the analogous 40*S*-configured product **66**, respectively. Gratifyingly, the data of these relatively small fragments were quite informative. Thus, the ^13^C NMR chemical shifts of the “inverted” 40*S*-isomer **66** were much closer to those of the C34–C41 sector of benthol A than those of 40*R*-configured **65** (Figure 3). Arguably even more convincing is the *J*-coupling pattern along the ring and the branching point: for the 40*S*-isomer **66**, it is almost spot-on to that of authentic benthol A (Scheme 13). This includes the conspicuously small ^3^ *J*_H39,H40_ = 1.8 Hz, which compares well to the 1.2 Hz in the natural product,^14^ whereas 40*R*-configured product **65** features a distinctively larger ^3^ *J*_H39,H40_ of 6.9 Hz.

**Scheme 13:**
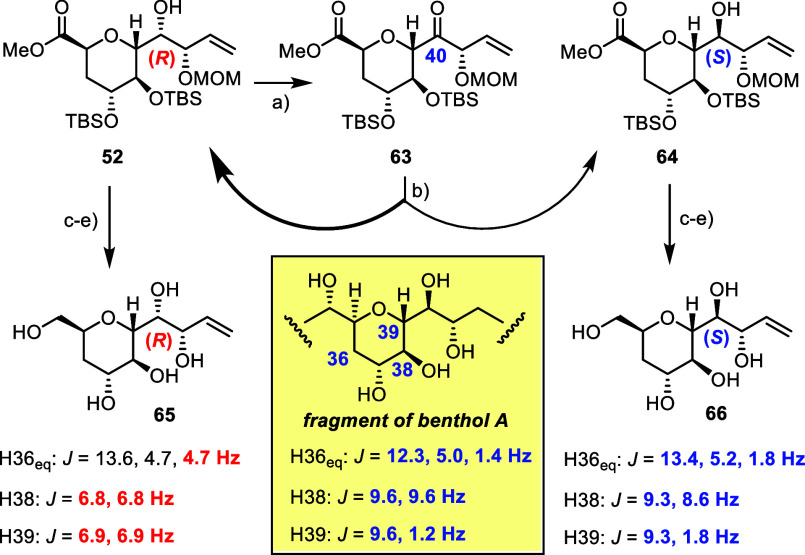
Reagents and Conditions: (a) Dess-Martin Periodinane, NaHCO_3_, CH_2_Cl_2_, 0 °C to RT, 86%; (b) NaBH_4_, EtOH, 0 °C, 48% (**52**) + 19% (**64**); (c) Me_3_SiBr, CH_2_Cl_2_, −78 °C to −10 °C, 94% (40*S* Series), 62% (40*R* Series); (d) Dibal-H, CH_2_Cl_2_, −78 °C to RT; (e) TBAF, THF, RT; 81% (**66**), 66% (**65**)

**Figure 3:**
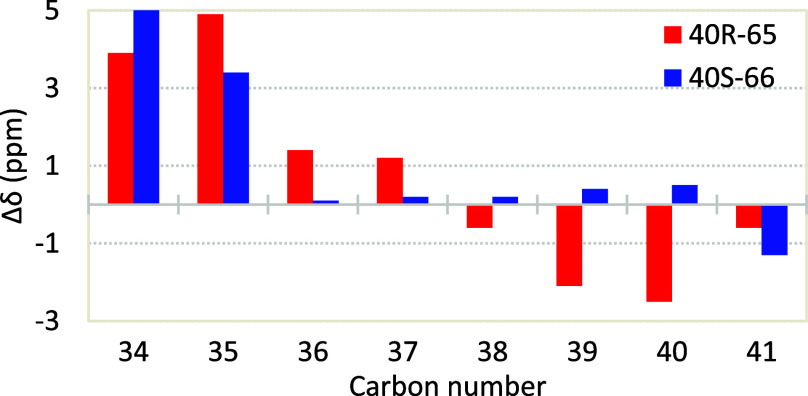
Graphical representation of the ^13^C NMR chemical shift differences (Δδ_C_, ppm; [D_4_]-MeOH) between fragments **65** and **66** and the pertinent region of the natural product.

All evidence suggested that the configuration of the C40 stereocenter of benthol A is opposite to that originally proposed by the isolation team, despite the apparently firm computational evidence on which their assignment had been based. At the meta level, this raises interesting questions as to potential limitations of computer-assisted structure assignment exercises in general.^97,98^ The final proof, however, has to be postponed until both epimers, that is, **1** and 40-*epi*-**1**, are reached by total synthesis and their spectral data have been compared in detail with those of the authentic natural product.

### The Revised Central Fragment

To this end, the preparation of the central building block had to be adjusted such that a C40,C41 *anti*-diol derivative was formed, since simple inversion of *syn*-configured compound **52** had proven to be unproductive. Methods allowing for reagent-controlled *anti*-oxyallylation of carbonyl compounds are rare;^99,100^ in view of the strong stereochemical bias observed in the reduction of ketone **63** as well as in the oxy-allylation leading to product **52** (see above), however, it seemed reasonable to assume that substrate control would be reigning anyway and a nonstereogenic oxyallyl anion equivalent might hence lead to a satisfactory outcome.^101^ Inspiration was drawn from an early report which had shown that allyl methyl ether can be deprotonated with *sec*-BuLi; after transmetalation with ZnCl_2_, the resulting intermediate engaged cyclohexanone in a clean oxyallylation, although the stereochemical course of the resulting *vic*-diol derivative had not been specified.^102^ Regardless of this aspect, we translated this reactivity from the allyl methyl ether to the corresponding MOM acetal **51** to ensure facile cleavage of the O-substituent after C–C-bond formation (Scheme 14). Specifically, the crude aldehyde derived from alcohol **50** was reacted in dilute THF solution (0.04 M)^103^ at low temperature with an organozinc species generated from **51** on treatment with *sec*-BuLi and subsequent transmetalation with excess ZnCl_2_ to give the mono-MOM-protected *anti*-diol **64** in a well-reproducible 78% yield with a dr ≥28:1 (NMR); gratifyingly, the reaction scaled well, furnishing >400 mg of product in the single largest batch. The scope of this new *anti*-oxyallylation reaction is currently under investigation. In any case, product **64** thus formed was identical in all respects to the tiny sample of the compound obtained by borohydride reduction of ketone **63** (see above).

**Scheme 14:**
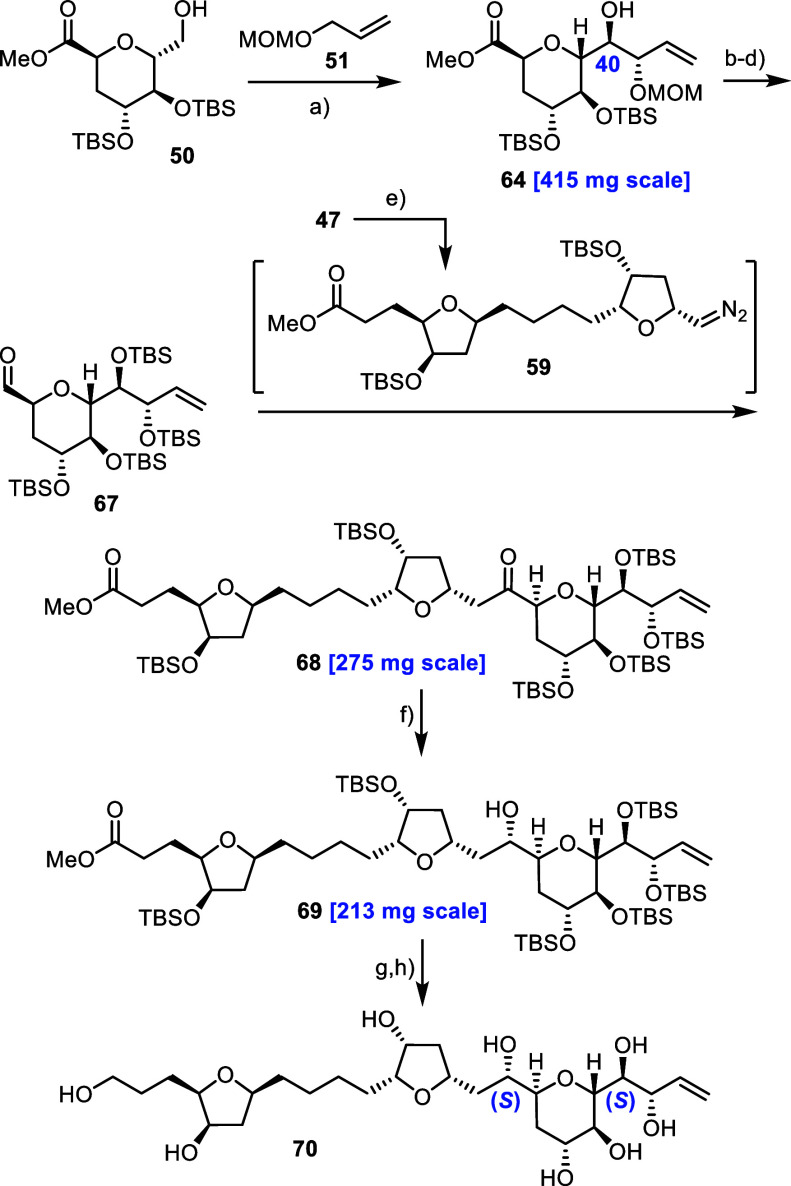
Reagents and Conditions: (a) (i) Dess-Martin Periodinane, NaHCO_3_, CH_2_Cl_2_, 0 °C to RT; (ii) **51**, *sec*-BuLi, ZnCl_2_, −78–0 °C, 78% (over Two Steps; dr = 28:1); (b) Me_3_SiBr, CH_2_Cl_2_, −78 °C to −10 °C, 81%; (c) TBSOTf, 2,6-Lutidine, CH_2_Cl_2_, 0 °C to RT, 97%; (d) Dibal-H, CH_2_Cl_2_, −78 °C; (e) aq. KOH, Toluene/MeOH, 0 °C, Then MgSO_4_; Then **67**, Toluene, 0 °C to RT, 74%; (f) L-Selectride, THF, −78 °C, 77% (dr = 10.4:1); (g) Dibal-H, CH_2_Cl_2_, −78 °C to RT; (h) TBAF, THF, RT, Quant. (over Two Steps)

The further elaboration into the revised building block **B′** followed the route outlined above for the *syn*-diol isomer. Thus, adjustment of the protecting group and oxidation state led to aldehyde **67**, which also succumbed to fragment coupling with the freshly prepared, unstabilized diazo derivative **59** with remarkable ease. The altered configuration at C40, though remote from the ketone group at C34 thus formed, proved beneficial in the subsequent reduction of product **68** with L-Selectide, which proceeded with notably higher diastereoselectivity in favor of the desired 34*S*-isomer **69** (dr = 10.4:1) than the reduction of its sibling **60** (dr = 2.9:1); once again, the epimers could be separated by flash chromatography.

### A Critical Substructure Authentication

Compound **69** represents the fully functional revised central building block **B′** comprising a 40*S*-configured center at the substituent branching off the pyran E-ring for use in the projected total synthesis of benthol A. Before that, however, we wanted to carry out yet another and arguably decisive test. To this end, **69** was elaborated into the globally deprotected polyol **70**: a match between its spectral data and those of the C18–C43 sector of benthol A—except for the termini—would lend credence to the anticipated structure revision of this marine natural product. This turned out to be the case, in that all shift differences were marginal (Δδ_C_ < 0.5 ppm) for the entire range from C20 to C38 (Figure 4).

**Figure 4:**
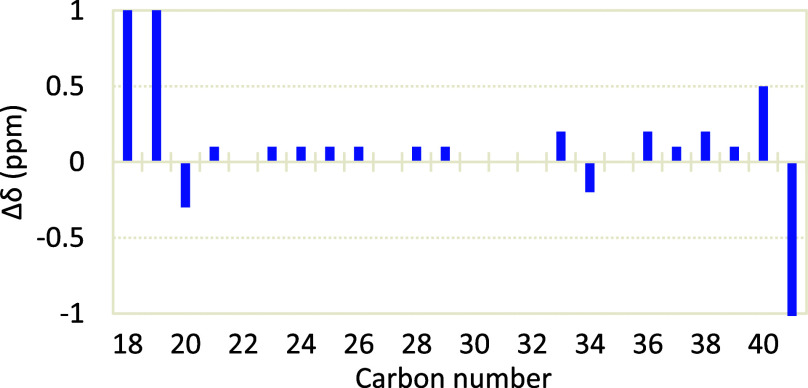
Graphical representation of the ^13^C NMR chemical shift differences (Δδ_C_, ppm; [D_4_]-MeOH) of the C18–C41 segment of benthol A reported in the literature and the 34*S*,40*S*-configured polyol derivative **70**.

## Conclusions

Since the still missing southern segment **C** did not bring any further ambiguity in structural terms (see the accompanying paper), we concluded that the proposed structure of benthol A requires only subtle revision. Actually, all information at this point suggested that a single stereochemical “site mutation” at C40 is mandatory from *R*, as originally proposed, to *S*, as deduced from our data. Interestingly, this is the only one of a total of 35 stereogenic centers decorating the 72 C atom long backbone of this marine “super-carbon-chain compound” (SCCC), for which the isolation team had neither been able to apply *J*-based configuration analysis nor secure Mosher ester derivatives; rather, the assignment had been solely based on computed coupling constants and shift data, in combination with a DP4+ probabilistic analysis. The impressively high score of 99.93% reached by this algorithm, however, seems to have been misleading. This may well raise questions as to the applicability of and the limitations inherent to this kind of computational structure assignment tools, in particular when applied to conformationally fairly unrestricted compounds. Another probable source of error is the fact that solvent effects had been ignored, which can be detrimental with polyhydroxylated compounds in which intramolecular hydrogen bonds compete with numerous hydrogen bonding interactions with the protic solvent used to record the NMR spectra; even implicit solvent models for MeOH have previously been recognized as being potentially misleading in such cases.^23,104,105^ The ultimate proof for the conclusion forecasted by the synthesis of two key building blocks described herein was reached by the total synthesis of nominal as well as revised benthol A outlined in the accompanying paper.

## Supplementary Material



## Notes

Experimental section including characterization data, copies of spectra of new compounds, and supporting crystallographic information (PDF)
