# Supplementary material for: Total Synthesis of Benthol A: Evidence for a Structure Revision
Source: J Am Chem Soc. 2026 Jul 16;148(29):30717–30. doi: 10.1021/jacs.6c05580 (PMC13426270; doi:10.1021/jacs.6c05580)
Supplement: Supplementary file 1 [file ja6c05580_si_001.pdf]

SUPPORTING INFORMATION

Total Synthesis of Benthol A: Evidence for a Structure Revision

Guanghao Huang,[+] Andrea Tomio,[+] Thomas Varlet, Conny Wirtz, and Alois Fürstner*

Max-Planck-Institut für Kohlenforschung, 45470 Mülheim/Ruhr, Germany

E-Mail: fuerstner@kofo.mpg.de

[+] These authors contributed equally

Table of Contents

Crystallographic Information	S2
General Information	S10
The Spiroketal Fragment A	S11
Sub-Structure Verification: Fragment A	S30
The Central Fragment B	S32
Sub-Fragment B1	S32
Alternative Route to Compound 41	S44
Sub-Fragment B2	S46
B1/B2 Sub-Fragment Coupling and Substructure Verification	S53
Revised Fragment B2 and Second Substructure Verification	S66
The Revised Central Fragment B	S72
Copies of Spectra of New Compounds	S77
References	S224

Crystallographic Information

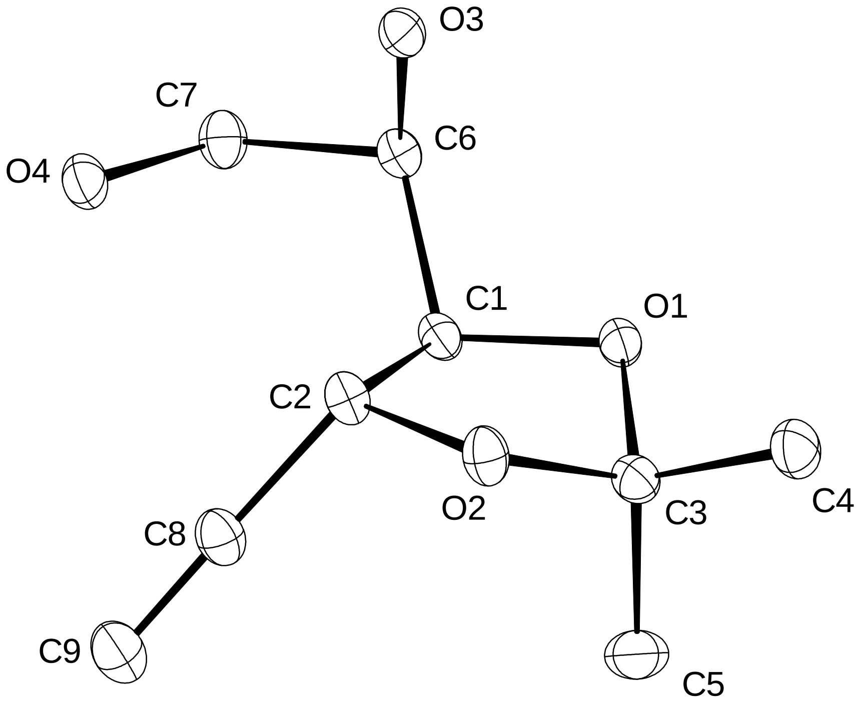

Figure S1. Structure of alkyne **3** in the solid state; H-atoms have been removed for clarity.

$C_9H_{14}O_4$, M_r = 186.20 g·mol^{-1}, colorless block, crystal size 0.122 x 0.101 x 0.034 mm^3, monoclinic, space group *C*2 [5], a = 19.451(3) Å, b = 6.5478(11) Å, c = 7.4579(12) Å, β = 95.279(8)°, V = 945.8(3) Å^3, T = 100(2) K, Z = 4, D_{calc} = 1.308 g·cm^3, λ = 0.71073 Å, $\mu(Mo\text{-}K_\alpha)$ = 0.102 mm^{-1}, Numerical absorption correction (T_{min} = 0, T_{max} = 0), Bruker AXS D8-Venture diffractometer with IµS Diamond Mo-anode X-ray source and PHOTON III detector, 2.103 < θ < 30.512°, 64262 measured reflections, 2890 independent reflections, 2729reflections with $I > 2\sigma(I)$, R_{int} = 0.0715. The structure was solved by *SHELXT* and refined by full-matrix least-squares (*SHELXL*) against F^2 to R_1 = 0.0290 [$I > 2\sigma(I)$], wR_2 = 0.0725, Absolute structure parameter = 0.1(3), 122 parameters. **CCDC-2532644**

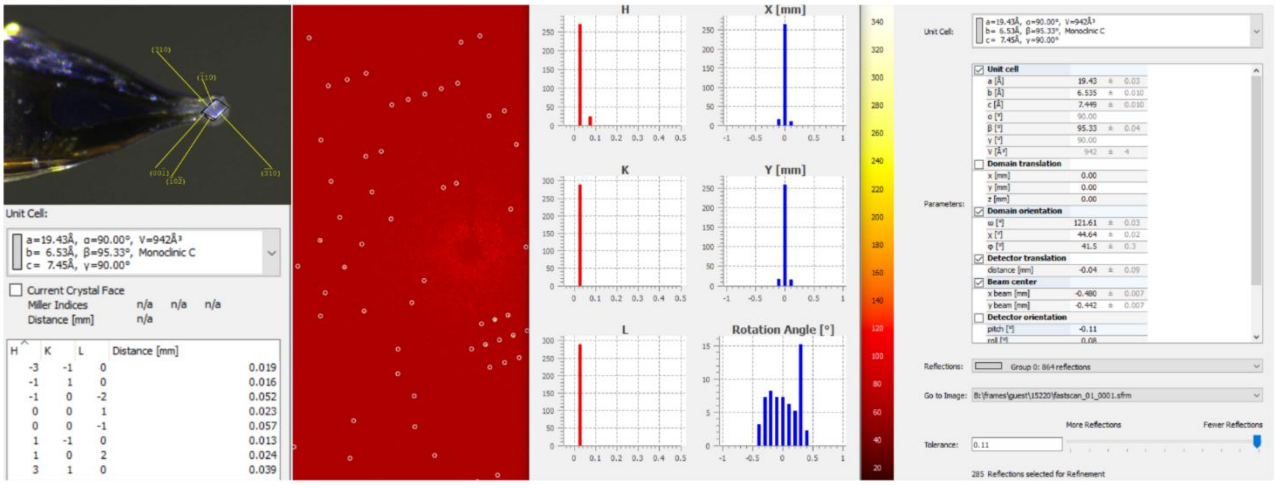

Figure S2. Crystal faces and unit cell determination/refinement

INTENSITY STATISTICS FOR DATASET

Resolution	#Data	#Theory	%Complete	Redundancy	Mean I	Mean I/s	Rmerge	Rsigma
Inf - 2.89	45	45	100.0	16.36	297.42	71.54	0.0280	0.0315
2.89 - 1.90	101	101	100.0	29.25	129.55	91.65	0.0317	0.0156
1.90 - 1.50	143	143	100.0	31.31	56.43	77.43	0.0414	0.0099
1.50 - 1.31	154	154	100.0	31.78	45.11	68.92	0.0519	0.0115
1.31 - 1.20	140	140	100.0	32.61	35.15	58.77	0.0617	0.0131
1.20 - 1.11	139	139	100.0	30.65	36.65	59.29	0.0644	0.0140
1.11 - 1.04	169	169	100.0	30.51	26.81	44.63	0.0754	0.0167
1.04 - 0.99	131	131	100.0	27.69	23.28	41.30	0.0838	0.0194
0.99 - 0.94	158	158	100.0	24.58	15.08	30.83	0.1135	0.0270
0.94 - 0.91	131	131	100.0	21.15	10.85	23.03	0.1296	0.0349
0.91 - 0.88	155	155	100.0	20.74	9.63	20.01	0.1373	0.0386
0.88 - 0.85	152	152	100.0	20.35	11.50	21.56	0.1314	0.0352
0.85 - 0.82	168	168	100.0	19.38	8.04	16.59	0.1584	0.0467
0.82 - 0.80	153	153	100.0	18.27	9.51	17.71	0.1476	0.0442
0.80 - 0.79	83	83	100.0	18.61	5.63	13.28	0.2121	0.0650
0.79 - 0.77	149	149	100.0	17.68	6.19	13.30	0.2046	0.0611
0.77 - 0.75	175	175	100.0	17.53	8.61	16.15	0.1741	0.0515
0.75 - 0.74	103	103	100.0	16.82	8.38	15.47	0.1897	0.0555
0.74 - 0.72	206	206	100.0	12.83	7.40	11.06	0.2064	0.0723
0.71 - 0.70	106	111	95.5	11.26	6.90	9.73	0.2126	0.0914
0.80 - 0.70	952	957	99.5	15.26	7.30	12.86	0.1964	0.0657
Inf - 0.70	2891	2896	99.8	22.20	26.25	33.06	0.0711	0.0247

The final structure refinement was carried out with using aspherical scattering factors with NoSpherA2.[1] DFT-calculated with ORCA using a B3LYP functional and def2-TZVPP basis set, whereby the H atom positions were refined using anisotropic atomic displacement parameters.

NoSpherA2 implementation of HAR makes use of tailor-made aspherical atomic form factors calculated on-the-fly from a Hirshfeld-partitioned electron density (ED) - not from spherical-atom form factors. The ED is calculated from a gaussian basis set single determinant SCF wave function - either Hartree-Fock or DFT using selected functional - for a fragment of the crystal. This fragment can be embedded in an electrostatic crystal field by employing cluster charges or modelled using implicit

solvation models, depending on the software used. The following options were used:

SOFTWARE:	ORCA 5.0
PARTITIONING:	NoSpherA2
INT ACCURACY:	Max
METHOD:	B3LYP
BASIS SET:	def2-TZVPP
CHARGE:	0
MULTIPLICITY:	1
DATE:	2024-06-10_11-00-59

Table S1. Crystal data and structure refinement of alkyne **3**

Empirical formula	$C_9 H_{14} O_4$	
Color	colourless	
Formula weight	186.20 g·mol^{-1}	
Temperature	100(2) K	
Wavelength	0.71073 Å	
Crystal system	Monoclinic	
Space group	**C2, (no. 5)**	
Unit cell dimensions	a = 19.451(3) Å	α= 90°.
	b = 6.5478(11) Å	β= 95.279(8)°.
	c = 7.4579(12) Å	γ = 90°.
Volume	945.8(3) Å^3	
Z	4	
Density (calculated)	1.308 Mg·m^{-3}	
Absorption coefficient	0.102 mm^{-1}	
F(000)	400 e	
Crystal size	0.122 x 0.101 x 0.034 mm^3	
θ range for data collection	2.103 to 30.512°.	
Index ranges	-27 ≤ h ≤ 27, -9≤ k ≤ 9, -10 ≤ l ≤ 10	
Reflections collected	64262	
Independent reflections	2890 [R_{int} = 0.0715]	
Reflections with I>2σ(I)	2729	
Completeness to θ = 25.242°	99.9 %	
Absorption correction	Numerical	
Max. and min. transmission	0 and 0	
Refinement method	Full-matrix least-squares on F^2	
Data / restraints / parameters	2890 / 1 / 122	
Goodness-of-fit on F^2	1.055	
Final R indices [I>2σ(I)]	R_1 = 0.0290	wR^2 = 0.0698
R indices (all data)	R_1 = 0.0322	wR^2 = 0.0725
Absolute structure parameter	0.1(3)	
Largest diff. peak and hole	0.275 and -0.180 e·Å^{-3}	

Table S2. Bond lengths [Å] and angles [°] of alkyne **3**

O(1)-C(1)	1.4388(15)	O(1)-C(3)	1.4384(16)
O(2)-C(2)	1.4412(16)	O(2)-C(3)	1.4289(16)
O(3)-H(3)	0.8400	O(3)-C(6)	1.4281(16)
O(4)-H(4)	0.8400	O(4)-C(7)	1.4220(16)
C(9)-H(9)	0.9500	C(9)-C(8)	1.193(2)
C(1)-H(1)	1.0000	C(1)-C(2)	1.5617(18)
C(1)-C(6)	1.5240(19)	C(2)-H(2)	1.0000
C(2)-C(8)	1.4702(18)	C(3)-C(4)	1.5125(19)
C(3)-C(5)	1.522(2)	C(4)-H(4A)	0.9800
C(4)-H(4B)	0.9800	C(4)-H(4C)	0.9800
C(5)-H(5A)	0.9800	C(5)-H(5B)	0.9800
C(5)-H(5C)	0.9800	C(6)-H(6)	1.0000
C(6)-C(7)	1.5200(17)	C(7)-H(7A)	0.9900
C(7)-H(7B)	0.9900		
C(3)-O(1)-C(1)	106.11(10)	C(3)-O(2)-C(2)	107.44(10)
C(6)-O(3)-H(3)	109.5	C(7)-O(4)-H(4)	109.5
C(8)-C(9)-H(9)	180.0	O(1)-C(1)-H(1)	109.8
O(1)-C(1)-C(2)	102.72(10)	O(1)-C(1)-C(6)	108.08(10)
C(2)-C(1)-H(1)	109.8	C(6)-C(1)-H(1)	109.8
C(6)-C(1)-C(2)	116.42(11)	O(2)-C(2)-C(1)	104.38(10)
O(2)-C(2)-H(2)	109.1	O(2)-C(2)-C(8)	110.99(11)
C(1)-C(2)-H(2)	109.1	C(8)-C(2)-C(1)	114.01(11)
C(8)-C(2)-H(2)	109.1	O(1)-C(3)-C(4)	109.30(11)
O(1)-C(3)-C(5)	110.59(11)	O(2)-C(3)-O(1)	103.12(10)
O(2)-C(3)-C(4)	108.85(11)	O(2)-C(3)-C(5)	111.95(11)
C(4)-C(3)-C(5)	112.60(12)	C(3)-C(4)-H(4A)	109.5
C(3)-C(4)-H(4B)	109.5	C(3)-C(4)-H(4C)	109.5
H(4A)-C(4)-H(4B)	109.5	H(4A)-C(4)-H(4C)	109.5
H(4B)-C(4)-H(4C)	109.5	C(3)-C(5)-H(5A)	109.5
C(3)-C(5)-H(5B)	109.5	C(3)-C(5)-H(5C)	109.5
H(5A)-C(5)-H(5B)	109.5	H(5A)-C(5)-H(5C)	109.5
H(5B)-C(5)-H(5C)	109.5	O(3)-C(6)-C(1)	111.66(11)
O(3)-C(6)-H(6)	108.3	O(3)-C(6)-C(7)	107.52(10)
C(1)-C(6)-H(6)	108.3	C(7)-C(6)-C(1)	112.74(10)
C(7)-C(6)-H(6)	108.3	C(9)-C(8)-C(2)	177.49(16)
O(4)-C(7)-C(6)	112.57(11)	O(4)-C(7)-H(7A)	109.1
O(4)-C(7)-H(7B)	109.1	C(6)-C(7)-H(7A)	109.1
C(6)-C(7)-H(7B)	109.1	H(7A)-C(7)-H(7B)	107.8

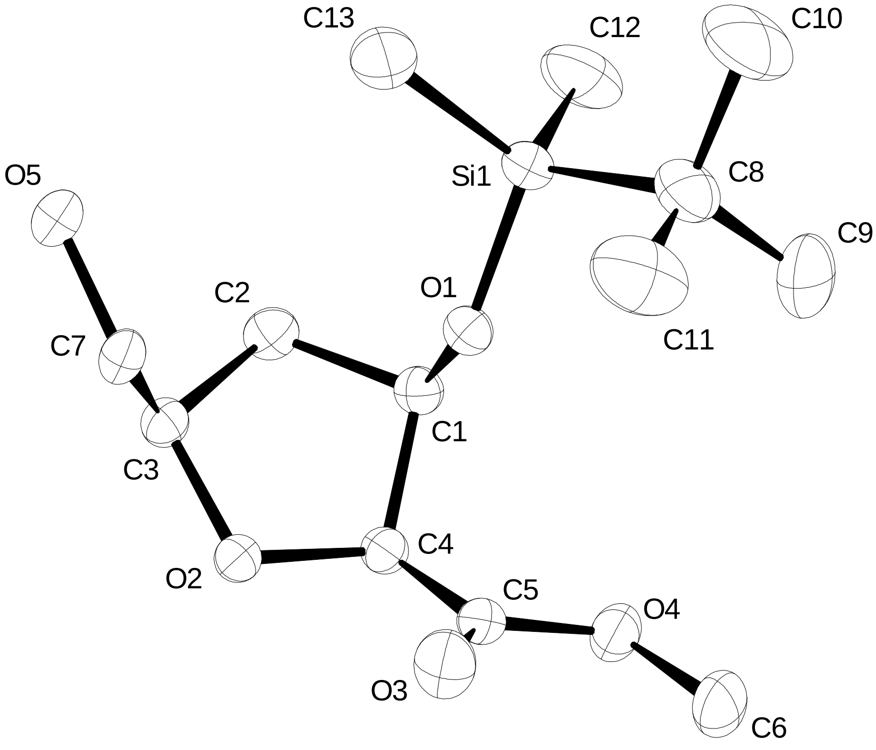

Figure S3. Structure of compound **S13** in the solid state; H-atoms have been removed for clarity.

$C_{13}H_{26}O_5Si$, M_r = 290.43 g·mol^{-1}, colourless plate, crystal size 0.46 x 0.14 x 0.121 mm^3, monoclinic, space group $P2_1$ [4], a = 6.7365(3) Å, b = 7.7995(4) Å, c = 15.6159(8) Å, β = 91.708(2)°, V = 820.12(7) Å^3, T = 150(2) K, Z = 2, D_{calc} = 1.176 g·cm^3, λ = 1.54178 Å, $\mu(Cu\text{-}K_\alpha)$ = 1.384 mm^{-1}, Gaussian absorption correction ($T_{\min}$ = 0.72, $T_{\max}$ = 0.92), Bruker-AXS Kappa Mach3 diffractometer with focus rotating Cu-anode X-ray source, 5.669 < θ < 72.195°, 29386 measured reflections, 3106 independent reflections, 3051 reflections with $I > 2\sigma(I)$, R_{int} = 0.0330. The structure was solved by *SHELXT* and refined by full-matrix least-squares (*SHELXL*) against F^2 to R_1 = 0.0324 [$I > 2\sigma(I)$], wR_2 = 0.0830, Absolute structure parameter = 0.001(12), 194 parameters. **CCDC-2532643**

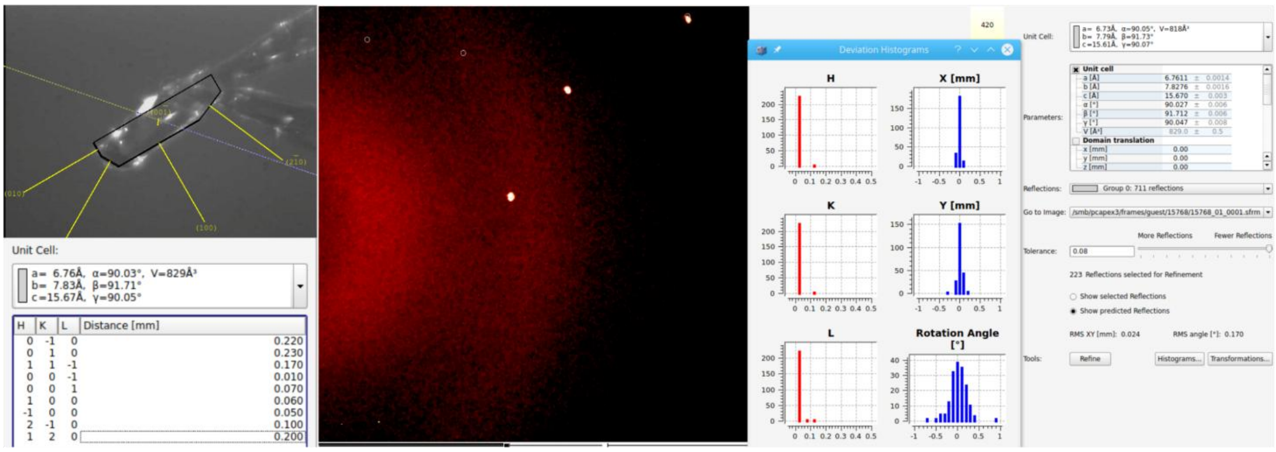

Figure S4. Crystal faces and unit cell determination/refinement.

INTENSITY STATISTICS FOR DATASET

Resolution	#Data	#Theory	%Complete	Redundancy	Mean I	Mean I/s	Rmerge	Rsigma
Inf - 3.13	48	54	88.9	7.78	184.96	70.03	0.0304	0.0154
3.13 - 2.20	108	108	100.0	8.96	122.66	71.00	0.0296	0.0134
2.20 - 1.76	155	155	100.0	10.45	78.34	74.55	0.0334	0.0130
1.76 - 1.53	168	168	100.0	9.81	45.12	65.97	0.0306	0.0137
1.53 - 1.40	155	155	100.0	8.57	44.03	60.80	0.0296	0.0150
1.40 - 1.29	155	155	100.0	7.10	34.33	53.33	0.0296	0.0172
1.29 - 1.22	158	158	100.0	11.45	35.29	66.51	0.0375	0.0159
1.22 - 1.16	149	149	100.0	15.05	28.12	80.02	0.0358	0.0126
1.16 - 1.11	148	148	100.0	14.06	20.29	72.48	0.0324	0.0130
1.11 - 1.07	173	173	100.0	14.28	20.10	72.84	0.0312	0.0130
1.07 - 1.03	160	160	100.0	13.39	14.96	62.81	0.0339	0.0146
1.03 - 1.00	139	139	100.0	11.84	16.86	63.73	0.0334	0.0144
1.00 - 0.97	148	153	96.7	10.29	13.12	58.33	0.0339	0.0163
0.97 - 0.94	178	190	93.7	10.78	9.76	56.68	0.0385	0.0153
0.94 - 0.92	155	163	95.1	9.82	9.18	54.20	0.0391	0.0170
0.92 - 0.90	152	154	98.7	10.15	8.00	52.22	0.0389	0.0167
0.90 - 0.88	149	150	99.3	9.71	7.03	50.35	0.0438	0.0179
0.88 - 0.86	159	165	96.4	4.78	6.96	28.26	0.0646	0.0467
0.86 - 0.84	196	208	94.2	2.01	6.24	12.02	0.0647	0.0841
0.84 - 0.83	106	111	95.5	1.87	4.72	9.95	0.0641	0.0966
0.83 - 0.81	156	244	63.9	1.15	4.70	10.50	0.0661	0.0919
0.91 - 0.81	852	965	88.3	4.19	6.27	25.49	0.0489	0.0563
Inf - 0.81	3115	3260	95.6	9.02	27.58	53.97	0.0330	0.0169

Table S3. Crystal data and structure refinement of compound **S13**

Empirical formula	$C_{13} H_{26} O_5 Si$	
Color	colorless	
Formula weight	290.43 g · mol^{-1}	
Temperature	150(2) K	
Wavelength	1.54178 Å	
Crystal system	MONOCLINIC	
Space group	**$P2_1$, (no. 4)**	
Unit cell dimensions	a = 6.7365(3) Å	α= 90°.
	b = 7.7995(4) Å	β= 91.708(2)°.
	c = 15.6159(8) Å	γ = 90°.
Volume	820.12(7) $Å^3$	
Z	2	
Density (calculated)	1.176 Mg · m^{-3}	
Absorption coefficient	1.384 mm^{-1}	
F(000)	316 e	
Crystal size	0.46 x 0.14 x 0.121 mm^3	
θ range for data collection	5.669 to 72.195°.	
Index ranges	$-7 \leq h \leq 8, -9\leq k \leq 9, -19 \leq l \leq 19$	
Reflections collected	29386	
Independent reflections	3106 [R_{int} = 0.0330]	
Reflections with I>2σ(I)	3051	
Completeness to θ = 67.679°	99.8 %	
Absorption correction	Gaussian	
Max. and min. transmission	0.92 and 0.72	
Refinement method	Full-matrix least-squares on F^2	
Data / restraints / parameters	3106 / 1 / 194	
Goodness-of-fit on F^2	1.127	
Final R indices [I>2σ(I)]	R_1 = 0.0324	wR^2 = 0.0825
R indices (all data)	R_1 = 0.0330	wR^2 = 0.0830
Absolute structure parameter	0.001(12)	
Largest diff. peak and hole	0.2 and -0.3 e · $Å^{-3}$	

Table S4. Bond lengths [Å] and angles [°] of compound **S13**

Si(1)-O(1)	1.6465(17)	Si(1)-C(8)	1.883(3)
Si(1)-C(12)	1.859(3)	Si(1)-C(13)	1.861(3)
O(1)-C(1)	1.426(3)	O(2)-C(3)	1.451(3)
O(2)-C(4)	1.426(3)	O(3)-C(5)	1.199(3)
O(4)-C(5)	1.340(3)	O(4)-C(6)	1.446(4)
O(5)-H(5)	0.96(4)	O(5)-C(7)	1.423(3)
C(1)-H(1)	1.00(3)	C(1)-C(2)	1.521(3)
C(1)-C(4)	1.526(3)	C(2)-C(3)	1.522(3)
C(3)-H(3)	1.04(3)	C(3)-C(7)	1.521(3)
C(4)-H(4)	0.93(3)	C(4)-C(5)	1.507(3)
C(8)-C(9)	1.534(5)	C(8)-C(10)	1.543(4)
C(8)-C(11)	1.531(5)		
O(1)-Si(1)-C(8)	104.66(12)	O(1)-Si(1)-C(12)	110.46(13)
O(1)-Si(1)-C(13)	109.22(12)	C(12)-Si(1)-C(8)	111.70(16)
C(12)-Si(1)-C(13)	110.07(19)	C(13)-Si(1)-C(8)	110.58(18)
C(1)-O(1)-Si(1)	128.22(15)	C(4)-O(2)-C(3)	109.80(17)
C(5)-O(4)-C(6)	115.6(2)	C(7)-O(5)-H(5)	107(2)
O(1)-C(1)-H(1)	112.3(15)	O(1)-C(1)-C(2)	112.97(18)
O(1)-C(1)-C(4)	108.3(2)	C(2)-C(1)-H(1)	112.4(16)
C(2)-C(1)-C(4)	101.07(17)	C(4)-C(1)-H(1)	109.1(15)
C(1)-C(2)-C(3)	103.95(19)	O(2)-C(3)-C(2)	105.58(18)
O(2)-C(3)-H(3)	107.9(17)	O(2)-C(3)-C(7)	107.58(19)
C(2)-C(3)-H(3)	110.4(17)	C(7)-C(3)-C(2)	115.1(2)
C(7)-C(3)-H(3)	109.9(16)	O(2)-C(4)-C(1)	107.05(18)
O(2)-C(4)-H(4)	110.4(17)	O(2)-C(4)-C(5)	109.44(19)
C(1)-C(4)-H(4)	107.9(18)	C(5)-C(4)-C(1)	113.46(19)
C(5)-C(4)-H(4)	108.5(18)	O(3)-C(5)-O(4)	124.6(2)
O(3)-C(5)-C(4)	125.8(2)	O(4)-C(5)-C(4)	109.5(2)
O(5)-C(7)-C(3)	111.6(2)	C(9)-C(8)-Si(1)	109.4(3)
C(9)-C(8)-C(10)	109.4(3)	C(10)-C(8)-Si(1)	110.4(3)
C(11)-C(8)-Si(1)	109.4(2)	C(11)-C(8)-C(9)	109.3(3)
C(11)-C(8)-C(10)	109.0(3)		

General Information

Unless stated otherwise, all reactions were carried out in flame-dried glassware using anhydrous solvents under argon atmosphere.

The solvents were purified by distillation over the indicated drying agents and were transferred under argon: THF, Et_2O (Mg/anthracene); acetonitrile, 2,6-lutidine, CH_2Cl_2, 1,2-DCE, nitromethane (CaH_2); toluene (Na/K alloy); methanol (Mg, stored over MS 3 Å). DMSO, DMF, Et_3N, pentane, and pyridine were dried by an adsorption solvent purification system based on molecular sieves.

Thin layer chromatography (TLC): Macherey-Nagel pre-coated plates (normal phase: POLYGRAM®SIL/UV254; reversed phase: ALUGRAM®SILCN/UV254); detection was achieved under UV-light (254 nm) and by staining with either acidic *para*-anisaldehyde, cerium ammonium molybdate, or basic $KMnO_4$ solution. Flash chromatography: Merck silica 60 (40–63 µm) (normal phase) or Carl Roth silica gel 60 cyano (45-70 µm) (reversed phase) with pre-distilled or HPLC grade solvents.

NMR: Spectra were recorded on Bruker AV 400, AV 500, AVIII 600, or AVneo 600 spectrometers in the indicated solvents; the 600 MHz spectrometers were equipped with Bruker cryoprobes; chemical shifts (δ) are given in ppm relative to TMS, coupling constants (J) in Hz. All spectra were recorded at 25 °C, unless stated otherwise. The solvent signals were used as references and the chemical shifts converted to the TMS scale ($CDCl_3$: δ_C = 77.16 ppm; residual $CHCl_3$: δ_H = 7.26 ppm; [D_4]-MeOH: δ_C = 49.00 ppm, residual [D_3]-MeOH: δ_H = 3.31 ppm; CD_2Cl_2: δ_C = 53.84 ppm, residual $CHDCl_2$: δ_H = 5.32 ppm; [D_6]-DMSO: δ_C = 39.52 ppm, residual [D_5]-DMSO: δ_H = 2.50 ppm). Multiplicities are indicated by the following abbreviations: s: singlet, d: doublet, t: triplet, q: quartet, hept: heptet, m: multiplet, br. s: broad singlet. ^{13}C NMR spectra were recorded in ^{1}H-decoupled manner and the values of the chemical shifts are rounded to one decimal point. Signal assignments were established using HSQC, HMBC, COSY, NOESY and other 2D experiments.

IR: Spectra were recorded on an Alpha Platinum ATR instrument (Bruker), wave numbers ($\tilde{\nu}$) in cm^{-1}.

MS (ESI-MS): Finnigan MAT 8200 (70 eV), ESI-MS: ESQ3000 (Bruker), accurate mass determinations: Bruker APEX III FTMS (7 T magnet) or Mat 95 (Finnigan).

Optical rotations ($[\alpha]_D^{20}$) were measured with an A-Krüss Optronic Model P8000-t polarimeter at a wavelength of 589 nm.

Molecular sieves were activated at 150 °C for 24 h under high vacuum (1 × 10^{-3} mbar) and stored under argon.

Unless stated otherwise, commercially available compounds (ABCR, Alfa Aesar, Aldrich, BLDPharm, TCI, Strem Chemicals, ChemPUR) were used as received.

The Spiroketal Fragment A

Compound 4. In a flame-dried two-necked round-bottom flask, 2,3-*O*-isopropylidene-D-ribofuranose

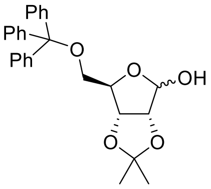

(**2**) (25.7 g, 135.12 mmol) was suspended in dry DMF (340 mL). 4-(Dimethyl-amino)pyridine (3.30 g, 27.03 mmol, 20 mol%) was added, followed by trityl chloride (56.5 g, 202.69 mmol) and triethylamine (47 mL, 337.81 mmol). The reaction was stirred under Ar for 5 d. The solution was then poured into ice-cold water (0.5 L) and the mixture extracted with *tert*-butyl methyl ether (3 × 300 mL). The combined organic layers were washed with sat. aq. NH_4Cl before being dried over anhydrous Na_2SO_4, filtered and concentrated under reduced pressure. The residue was purified by flash chromatography on silica (hexanes/EtOAc, 95:5 to 70:30) to afford the title product as white dry foam (45.37 g, 77%). *Mixture of diastereomers*: $[\alpha]_D^{20}$ = –6.2° (c = 1.4, MeOH); ^{1}H NMR (400 MHz, $CDCl_3$): δ = 7.43 – 7.19 (m, 19.5H), 5.72 (ddd, *J* = 11.3, 4.1, 1.4 Hz, 0.3H), 5.31 (dd, *J* = 9.1, 1.4 Hz, 1H), 4.76 (dt, *J* = 5.9, 1.4 Hz, 1H), 4.72 (ddd, *J* = 5.8, 4.0, 1.4 Hz, 0.3H), 4.63 (dd, *J* = 5.9, 1.4 Hz, 1H), 4.56 (dt, *J* = 6.3, 1.3 Hz, 0.3H), 4.33 (td, *J* = 3.6, 1.7 Hz, 1H), 4.19 – 4.15 (m, 0.3H), 3.97 (dd, *J* = 11.3, 1.5 Hz, 0.3H), 3.88 (dd, *J* = 9.0, 1.5 Hz, 1H), 3.47 – 3.28 (m, 2.3H), 3.00 (ddd, *J* = 10.2, 2.9, 1.4 Hz, 0.3H), 1.53 (d, *J* = 1.4 Hz, 0.9H), 1.46 (d, *J* = 1.4 Hz, 3H), 1.34 (d, *J* = 1.3 Hz, 0.9H), 1.32 (d, *J* = 1.3 Hz, 3H) ppm; ^{13}C NMR (101 MHz, $CDCl_3$): δ = 143.6 (3C, *minor isomer*), 142.9 (3C, *major isomer*), 128.8 (6C, *major isomer*), 128.7 (6C, *minor isomer*), 128.2 (6C, *major isomer*), 128.1 (6C, *minor isomer*), 127.6 (3C, *major isomer*), 127.3 (3C, *minor isomer*), 113.2 (*minor isomer*), 112.4 (*major isomer*), 103.7 (*major isomer*), 98.1 (*minor isomer*), 88.3 (*major isomer*), 87.6 (*minor isomer*), 87.2 (*major isomer*), 86.2 (*major isomer*), 82.3 (*minor isomer*), 82.1 (*major isomer*), 80.2 (*minor isomer*), 79.6 (*minor isomer*), 65.6 (*minor isomer*), 65.2 (*major isomer*), 26.7 (*major isomer*), 26.3 (*minor isomer*), 25.2 (*major isomer*), 24.9 (*minor isomer*) ppm; IR (film) $\tilde{\nu}$ = 3435, 3059, 3032, 2987, 2940, 2874, 1597, 1491, 1448, 1374, 1211, 1071, 705 cm^{-1}; HRMS (ESI): *m/z:* calcd. for $C_{27}H_{28}O_5Na$ $[M+Na]^+$: 455.18268, found: 455.18289.

Compound 5. In a flame-dried three-necked round bottom flask equipped with a condenser and a

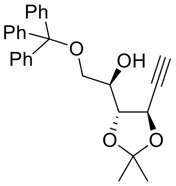

Teflon-coated stir bar, compound **4** (12.7 g, 29.35 mmol) was suspended in dry MeOH (300 mL). K_2CO_3 (12.17 g, 88 mmol) was added and the suspension was stirred at 55 °C (bath temperature). After 10 min, a solution of dimethyl (1-diazo-2-oxopropyl)phosphonate (**6**) (14.1 g, 73.4 mmol) in dry MeOH (56 mL) was added dropwise over 8 h using a syringe-pump. Once the addition was complete, the solution was allowed to reach room temperature before sat. aq. NH_4Cl (140 mL) was added. The volatile materials were removed under reduced pressure and the residue was extracted with EtOAc (3 × 150 mL). The combined organic layers were washed with brine (150 mL), dried over anhydrous Na_2SO_4, filtered and concentrated under reduced pressure to afford an orange oil. This residue was purified by flash chromatography on silica (hexanes/EtOAc, 95:5 to 80:20) to afford the title compound as a colorless

viscous oil (10.39 g, 83%). $[\alpha]_D^{20}$ = +5.9° (c = 1.0, $CHCl_3$); ^1H NMR (400 MHz, $CDCl_3$): δ = 7.47 – 7.44 (m, 6H), 7.34 – 7.28 (m, 6H), 7.28 – 7.22 (m, 3H), 4.69 (dd, *J* = 6.3, 2.1 Hz, 1H), 4.20 (t, *J* = 6.1 Hz, 1H), 3.92 – 3.82 (m, 1H), 3.32 (d, *J* = 5.1 Hz, 2H), 2.46 (d, *J* = 2.1 Hz, 1H), 2.43 (d, *J* = 4.4 Hz, 1H), 1.50 (d, *J* = 0.8 Hz, 3H), 1.37 (d, *J* = 0.8 Hz, 3H) ppm; ^{13}C NMR (101 MHz, $CDCl_3$): δ = 143.8 (3C), 128.8 (6C), 128.0 (6C), 127.3 (3C), 111.0, 87.2, 82.1, 82.0, 74.5, 71.1, 67.1, 64.5, 27.0, 26.2 ppm; IR (film) $\tilde{\nu}$ = 3482, 3292, 3086, 3059, 2989, 2934, 2883, 1597, 1491, 1448, 1373, 1213, 1059, 704 cm^{-1}; HRMS (ESI): *m/z:* calcd. for $C_{28}H_{28}O_4Na$ $[M+Na]^+$: 451.18812, found: 451.18798.

Compound 3. Trityl ether **5** (9.88 g, 23 mmol) was solubilized in dry CH_2Cl_2 (138 mL) in a flame-dried two-necked round bottom flask. Dry MeOH (15 mL) was added, followed by $NaHSO_4$ supported on silica (10 wt%, 1 g).[1] The suspension was stirred under Ar at room temperature and the reaction monitored by TLC. When complete consumption of the substrate was noticed (ca. 3 h), powdered $NaHCO_3$ (1 g) was added and, after 5 min, the solution was filtered through a plug of Celite, which was carefully rinsed with *tert*-butyl methyl ether (3 × 50 mL). The combined filtrates were evaporated under reduced pressure and the residue was purified by flash chromatography on silica (hexanes/EtOAc 80:20 to 1:1) to afford the title compound as a colorless crystalline solid (3.53 g, 82%). $[\alpha]_D^{20}$ = +11.4° (c = 1.4, $CHCl_3$); ^1H NMR (400 MHz, [D4]-MeOH): δ = 4.69 (dd, *J* = 6.1, 2.1 Hz, 1H), 4.14 – 4.05 (m, 1H), 3.72 – 3.62 (m, 2H), 3.60 – 3.49 (m, 1H), 2.96 (d, *J* = 2.1 Hz, 1H), 1.46 (s, 3H), 1.39 (s, 3H) ppm; ^{13}C NMR (101 MHz, [D4]-MeOH): δ = 111.9, 83.5, 83.4, 75.7, 73.6, 68.5, 64.3, 27.3, 26.4 ppm; IR (film) $\tilde{\nu}$ = 3397, 3287, 2990, 2938, 1457, 1375, 1214, 1063, 859 cm^{-1}; HRMS (ESI): *m/z:* calcd. for $C_9H_{14}O_4Na$ $[M+Na]^+$: 209.07837, found: 209.07843.

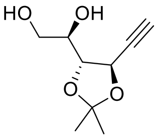

Crystals suitable for X-ray diffraction were grown by vapor diffusion of pentane into a saturated solution of the compound in CH_2Cl_2.

Compound 7. In a flame-dried two-necked round bottom flask, diol **3** (3.53 g, 18.96 mmol) was dissolved in CH_2Cl_2 (190 mL). Dibutyltin oxide (472 mg, 1.89 mmol, 10 mol%) was added and the resulting suspension was sonicated for 10 min before being vigorously stirred for 1 h at room temperature under Ar. *N*,*N*-Diisopropylethylamine (3.63 mL, 20.85 mmol) was then added, followed by tosyl chloride (3.72 g, 19.53 mmol). The mixture was stirred overnight (16 h). 1,8-Diazabicyclo[5.4.0]undec-7-ene (7.1 mL, 47.39 mmol) was introduced and stirring continued for another 2.5 h at room temperature. The mixture was then filtered through a plug of Celite, which was carefully rinsed with CH_2Cl_2 (3 × 100 mL). The combined filtrates were transferred into a separatory funnel and washed with aq. HCl (2 M, 50 mL) and brine (50 mL) before being dried over Na_2SO_4, filtered and carefully concentrated under reduced pressure (***Note***: *the product is volatile*; bath temperature: 40 °C, > 650 mbar). The residue was purified by flash chromatography on silica (*n*-pentane/Et_2O, 98:2 to 8:2) to afford the title compound as a colorless oil (2.81 g, 88%). $[\alpha]_D^{20}$ = +7.3° (c = 2.3, $CHCl_3$); ^1H

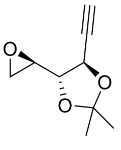

NMR (400 MHz, CD_2Cl_2): δ = 4.50 (dd, *J* = 6.9, 2.1 Hz, 1H), 4.05 (dd, *J* = 6.9, 4.4 Hz, 1H), 3.13 (td, *J* = 4.2, 2.6 Hz, 1H), 2.82 (dd, *J* = 4.8, 4.1 Hz, 1H), 2.67 (dd, *J* = 4.8, 2.6 Hz, 1H), 2.61 (d, *J* = 2.1 Hz, 1H), 1.46 (q, *J* = 0.7 Hz, 3H), 1.41 (q, *J* = 0.7 Hz, 3H) ppm; ^{13}C NMR (101 MHz, CD_2Cl_2): δ = 111.5, 81.7, 81.1, 75.1, 67.2, 51.0, 45.0, 26.9, 26.2 ppm; IR (film) $\tilde{\nu}$ = 3274, 2990, 2937, 1456, 1375, 1241, 1213, 1055, 863 cm^{-1}; HRMS (ESI): *m/z:* calcd. for $C_9H_{12}O_3Na$ $[M+Na]^+$: 191.06806, found: 191.06786.

Compound S1. In a flame-dried flask, CuI (636 mg, 3.34 mmol, 20 mol%) was suspended in dry THF

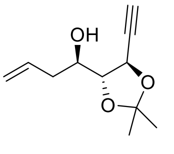

(110 mL). The suspension was cooled to −78 °C (dry ice/acetone bath) and vinylmagnesium bromide (1 M in THF, 41.7 mL, 41.7 mmol) was added dropwise over 10 min. The bright orange solution was stirred for 10 min at −78 °C before a solution of epoxide **7** (2.81 g, 16.71 mmol) in THF (10 mL) was added dropwise. The resulting slurry was allowed to reach −50 °C over the course of 1 h before being directly cannulated into a cooled solution of sat. aq. NH_4Cl (100 mL) (0 °C, ice bath). The mixture was vigorously stirred at room temperature until a clear organic layer and a clear blue aqueous layer had formed. The aqueous layer was extracted with Et_2O (3 × 50 mL), the combined organic phases were washed with brine and dried over Na_2SO_4 before being filtered and concentrated under reduced pressure. The residue was purified by flash chromatography on silica (*n*-pentane/Et_2O, 95:5 to 8:2) to afford the title product as a colorless oil (3.24 g, 98%). $[\alpha]_D^{20}$ = +19.3° (c = 0.63, $CHCl_3$); ^1H NMR (400 MHz, $CDCl_3$): δ = 5.87 (ddt, *J* = 17.2, 10.2, 7.1 Hz, 1H), 5.26 – 5.12 (m, 2H), 4.70 (dd, *J* = 7.0, 2.1 Hz, 1H), 4.13 (dd, *J* = 7.0, 4.0 Hz, 1H), 3.94 – 3.85 (m, 1H), 2.56 (d, *J* = 2.1 Hz, 1H), 2.38 (dddt, *J* = 13.1, 6.4, 4.9, 1.3 Hz, 1H), 2.28 (dddt, *J* = 14.3, 8.5, 7.3, 1.3 Hz, 1H), 2.11 (q, *J* = 2.3 Hz, 1H), 1.51 (d, *J* = 0.7 Hz, 3H), 1.45 (d, *J* = 0.7 Hz, 3H) ppm; ^{13}C NMR (101 MHz, $CDCl_3$): δ = 133.8, 118.7, 110.7, 83.7, 82.1, 74.8, 70.0, 65.6, 37.2, 26.9, 25.9 ppm; IR (film) $\tilde{\nu}$ = 3475, 3295, 2989, 2936, 1643, 1375, 1240, 1211, 1052 cm^{-1}; HRMS (ESI): *m/z:* calcd. for $C_{11}H_{16}O_3Na$ $[M+Na]^+$: 219.09946, found: 219.09916.

Compound 8. In a flame-dried flask, degreased NaH (790 mg, 32.97 mmol) was suspended in DMF (60

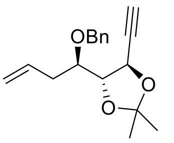

mL). The suspension was cooled to 0 °C (ice bath) before a solution of alcohol **S1** (3.23 g, 16.48 mmol) in DMF (20 mL) was added dropwise. The resulting mixture was stirred for 30 min at 0 °C before benzyl bromide (2.16 mL, 18.13 mmol) was introduced. After stirring for 2 h at room temperature, the mixture was cooled to 0 °C (ice bath) and the reaction carefully quenched with sat. aq. NH_4Cl (30 mL). The mixture was extracted with *tert*-butyl methyl ether (3 × 50 mL), the combined organic phases were washed with brine (3 × 100 mL) and dried over Na_2SO_4 before being filtered and concentrated under reduced pressure. The residue was purified by flash chromatography on silica (*n*-pentane/*tert*-butyl methyl ether, 100:0 to 97:3) to afford the title compound as a pale-yellow oil (4.22 g, 89%). $[\alpha]_D^{20}$ = +23.7 (c = 1.1, $CHCl_3$); ^1H NMR (400 MHz, $CDCl_3$): δ = 7.38 – 7.31 (m, 4H), 7.31 – 7.26 (m, 1H), 5.87 (ddt, *J* = 17.2, 10.2, 7.1 Hz, 1H), 5.16 (dq, *J* = 17.1, 1.6

Hz, 1H), 5.11 (ddt, J = 10.2, 2.1, 1.1 Hz, 1H), 4.73 – 4.60 (m, 3H), 4.22 (dd, J = 6.9, 4.3 Hz, 1H), 3.70 (td, J = 6.2, 4.3 Hz, 1H), 2.54 (d, J = 2.1 Hz, 1H), 2.40 (ddt, J = 7.4, 6.2, 1.3 Hz, 2H), 1.50 (d, J = 0.8 Hz, 3H), 1.44 (d, J = 0.7 Hz, 3H) ppm; ^{13}C NMR (101 MHz, $CDCl_3$): δ = 138.4, 134.2, 128.5 (2C), 127.9 (2C), 127.8, 118.0, 110.6, 83.4, 82.2, 78.1, 74.6, 73.2, 66.6, 36.0, 26.9, 26.0 ppm; IR (film) $\tilde{\nu}$ = 3286, 3067, 3033, 2988, 2936, 1723, 1642, 1497, 1454, 1381, 1211, 1064, 698 cm^{-1}; HRMS (ESI): *m/z:* calcd. for $C_{18}H_{22}O_3Na$ [M+Na]$^+$: 309.14611, found: 309.14625.

Compound S2. In a flask, acetonide **8** (4.2 g, 14.67 mmol) was dissolved in aq. acetic acid (80% *w/w*, 75 mL) and the solution was stirred at 65 °C (bath temperature) overnight. The mixture was diluted with water (50 mL) and extracted with CH_2Cl_2 (3 × 100 mL). The combined organic phases were washed with sat. aq. $NaHCO_3$ (200 mL) before being dried over Na_2SO_4, filtered and concentrated under reduced pressure.

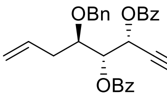

The residue was dissolved in pyridine (40 mL) and the resulting solution cooled to 0 °C (ice bath) before benzoyl chloride (4.22 mL, 36.67 mmol) was added dropwise. The mixture was allowed to reach room temperature overnight. For work up, the mixture was cooled to 0 °C (ice bath) before water (100 mL) followed by aq. HCl (2 M, 100 mL) were added. The mixture was extracted with *tert*-butyl methyl ether (3 × 50 mL), the combined organic phases were consecutively washed with aq. HCl (2 M, 200 mL), sat. aq. $NaHCO_3$ (200 mL) and brine (200 mL) before being dried over anhydrous Na_2SO_4, filtered and concentrated under reduced pressure. The residue was purified by flash chromatography on silica (hexanes/EtOAc, 100:0 to 8:2) to afford the title product as a colorless oil (6.2 g, 93%). $[\alpha]_D^{20}$ = +10.8 (c = 2.0, $CHCl_3$); ^{1}H NMR (400 MHz, $CDCl_3$): δ = 8.10 – 8.00 (m, 4H), 7.58 – 7.53 (m, 2H), 7.46 – 7.39 (m, 4H), 7.35 – 7.17 (m, 5H), 6.02 (dd, J = 5.6, 2.2 Hz, 1H), 5.93 (ddt, J = 17.2, 10.1, 7.0 Hz, 1H), 5.83 (t, J = 5.7 Hz, 1H), 5.20 – 5.10 (m, 2H), 4.60 (s, 2H), 4.00 (ddd, J = 6.7, 5.7, 4.6 Hz, 1H), 2.64 – 2.44 (m, 3H) ppm; ^{13}C NMR (101 MHz, $CDCl_3$): δ = 165.6, 165.2, 137.6, 133.9, 133.5, 133.4, 130.0 (4C), 129.7, 129.4, 128.59 (2C), 128.56 (2C), 128.5 (2C), 128.3 (2C), 127.9, 118.2, 78.0, 76.4, 76.1, 73.5, 72.0, 63.3, 34.9 ppm; IR (film) $\tilde{\nu}$ = 3291, 3065, 3033, 2945, 1722, 1601, 1494, 1452, 1315, 1245, 1091, 1067, 1026, 708 cm^{-1}; HRMS (ESI): *m/z:* calcd. for $C_{29}H_{26}O_5Na$ [M+Na]$^+$: 477.16724, found: 477.16765.

Compound S3. A stock solution of benzyl ether **S2** (6.2 g, 13.64 mmol) in dry CH_2Cl_2 (5.0 mL) was prepared. A Schlenk flask (diameter: *ca*. 6 cm) was charged with 2,3-dichloro-5,6-dicyano-1,4-benzoquinone (1.16 g, 5.11 mmol) under Ar and Ar was flushed through the solid for 5 min before CH_2Cl_2 (80 mL) was added. Next, a quarter of the above mentioned stock solution of the benzyl ether **S2** (corresponding to 1.55 g, 3.41 mmol) and degassed water (1.5 mL) were added. The resulting mixture was sparged with Ar for 10 min at 0 °C (ice bath). The Schlenk tube was then placed in a photoreactor equipped with blue LED-strips (at a distance of *ca*. 5 cm) and a fan and the reaction mixture stirred for 2.5 h under Ar while being irradiated. An orange

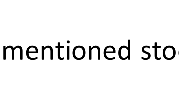

precipitate was formed during the course of the reaction. Sat. aq. $NaHCO_3$ (50 mL) was added and the mixture was vigorously stirred under air for 30 min before it was transferred into a separatory funnel and extracted with CH_2Cl_2 (3 × 100 mL). The combined organic phases were washed with brine (200 mL), dried over anhydrous Na_2SO_4, filtered and concentrated under reduced pressure. The process was repeated three times to engage all the starting material. The combined residues were purified by flash chromatography on silica (hexanes/EtOAc, 100:0 to 8:2) to afford the title compound as a pale-yellow oil (3.79 g, 76%). $[\alpha]^{20}_{D}$ = +29.6 (c = 2.0, $CHCl_3$); ^{1}H NMR (400 MHz, $CDCl_3$): δ = 8.09 (dt, *J* = 8.4, 1.3 Hz, 4H), 7.65 – 7.54 (m, 2H), 7.46 (t, *J* = 7.6 Hz, 4H), 6.12 (dd, *J* = 4.3, 2.3 Hz, 1H), 5.87 (ddt, *J* = 19.3, 9.6, 7.1 Hz, 1H), 5.55 (dd, *J* = 7.1, 4.3 Hz, 1H), 5.22 – 5.12 (m, 2H), 4.09 – 4.00 (m, 1H), 2.57 – 2.41 (m, 2H), 2.40 – 2.29 (m, 1H) ppm; ^{13}C NMR (101 MHz, $CDCl_3$): δ = 165.9, 165.7, 133.8, 133.7, 133.6, 130.14 (2C), 130.1 (2C), 129.5, 129.2, 128.70 (2C), 128.65 (2C), 119.4, 77.9, 76.1, 76.0, 68.8, 63.4, 37.5 ppm; IR (film) $\tilde{\nu}$ = 3501, 3294, 3073, 2960, 1721, 1601, 1452, 1316, 1246, 1092, 1068, 707 cm^{-1}; HRMS (ESI): *m/z:* calcd. for $C_{22}H_{20}O_5Na$ $[M+Na]^+$: 387. 12029, found: 387, 12054.

Compound 10. In a flame-dried flask, a solution of alcohol **S3** (3.8 g, 10.40 mmol) in CH_2Cl_2 (110 mL)

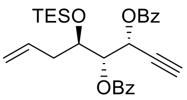

was cooled to 0 °C (ice bath) before 2,6-lutidine (1.82 mL, 15.6 mmol) and TESOTf (2.82 mL, 12.47 mmol) were added. The solution was stirred for 2 h at 0 °C before the reaction was quenched with sat. aq. NH_4Cl (40 mL). The mixture was extracted with *tert*-butyl methyl ether (3 × 50 mL). The combined organic phases were washed with brine (100 mL), dried over Na_2SO_4, filtered and concentrated under reduced pressure. The residue was purified by flash chromatography on silica (*n*-pentane/*tert*-butyl methyl ether, 100:0 to 9:1) to afford the title product as a colorless oil (4.38 g, 88%). $[\alpha]^{20}_{D}$ = –6.0 (c = 1.1, $CHCl_3$); ^{1}H NMR (400 MHz, $CDCl_3$): δ = 8.04 – 8.00 (m, 4H), 7.58 – 7.49 (m, 2H), 7.46 – 7.35 (m, 4H), 5.99 – 5.84 (m, 2H), 5.64 (dd, *J* = 6.5, 4.7 Hz, 1H), 5.19 – 5.08 (m, 2H), 4.30 (dt, *J* = 6.4, 5.1 Hz, 1H), 2.62 – 2.51 (m, 2H), 2.51 – 2.42 (m, 1H), 0.92 (t, *J* = 7.9 Hz, 9H), 0.59 (q, *J* = 7.8 Hz, 6H) ppm; ^{13}C NMR (101 MHz, $CDCl_3$): δ = 165.7, 165.3, 134.1, 133.5, 133.2, 130.0 (2C), 129.9 (2C), 129.5 (2C), 128.53 (2C), 128.50 (2C), 118.2, 78.3, 76.1, 76.0, 71.0, 63.2, 38.2, 6.9 (3C), 5.1 (3C) ppm; IR (film) $\tilde{\nu}$ = 3302, 3073, 2955, 2912, 2877, 1728, 1602, 1452, 1316, 1270, 1246, 193, 709 cm^{-1}; HRMS (ESI): *m/z:* calcd. for $C_{28}H_{34}O_5SiNa$ $[M+Na]^+$: 501.20677, found: 501.20658.

Compound S4. In a flame-dried flask, alcohol **S1** (450 mg, 2.29 mmol) was dissolved in pyridine (23

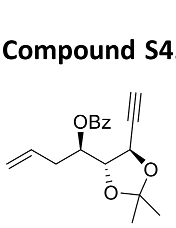

mL). The solution was cooled to 0 °C (ice bath) and benzoyl chloride (665 µL, 5.73 mmol) was added, and the resulting mixture stirred at room temperature overnight. The solution was cooled to 0 °C using an ice bath and distilled water (5 mL) was added followed by aq. HCl (2 M, 5 mL). The mixture was extracted with *tert*-butyl methyl ether (3 × 10 mL). The combined organic phases were washed with aq. HCl (2 M, 10 mL), sat. aq. $NaHCO_3$ (10 mL) and brine, dried over Na_2SO_4, filtered and concentrated under reduced pressure. The residue was purified

by flash chromatography on silica (hexanes/EtOAc, 98:2 to 96:4) to obtain the title compound as a colorless oil (653 mg, 95%). $[\alpha]_D^{20}$ = +16.8° (c = 0.76, $CHCl_3$); ^{1}H NMR (400 MHz, $CDCl_3$): δ = 8.06 – 8.02 (m, 2H), 7.61 – 7.52 (m, 1H), 7.51 – 7.40 (m, 2H), 5.92 – 5.77 (m, 1H), 5.39 (dt, *J* = 7.5, 5.2 Hz, 1H), 5.22 – 5.07 (m, 2H), 4.73 (dd, *J* = 6.9, 2.1 Hz, 1H), 4.32 (dd, *J* = 6.9, 5.4 Hz, 1H), 2.67 – 2.51 (m, 2H), 2.50 (d, *J* = 2.1 Hz, 1H), 1.50 (d, *J* = 0.7 Hz, 3H), 1.35 (d, *J* = 0.7 Hz, 3H) ppm; ^{13}C NMR (101 MHz, $CDCl_3$): δ = 165.9, 133.3, 132.6, 130.1, 129.9 (2C), 128.5 (2C), 119.0, 111.2, 82.0, 81.3, 75.0, 72.5, 67.3, 35.7, 26.9, 26.2 ppm; IR (film) $\tilde{\nu}$ = 3293, 2989, 2937, 1720, 1644, 1602, 1267, 1110, 1069, 711 cm^{-1}; HRMS (ESI): *m/z:* calcd. for $C_{18}H_{20}O_4Na$ $[M+Na]^+$: 323.12528, found: 323.12538.

Compound S5. In a flame-dried flask, benzoate **S4** (645 mg, 2.15 mmol) was dissolved in THF (8.5 mL).

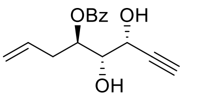

Water (2 mL) was added and the resulting mixture cooled to 0 °C (ice bath). Trifluoroacetic acid (9 mL) was added and stirring continued at room temperature for 24 h. The mixture was diluted with water (10 mL) and extracted with CH_2Cl_2 (3 × 10 mL). The combined organic phases were washed with sat. aq. $NaHCO_3$ (10 mL), dried over Na_2SO_4, filtered and concentrated under reduced pressure. The residue was purified by flash chromatography on silica (hexanes/EtOAc, 8:2 to 6:4) to afford the title compound as a colorless oil (527 mg, 94%). $[\alpha]_D^{20}$ = +11.1 (c = 1.87, $CHCl_3$); ^{1}H NMR (400 MHz, $CDCl_3$): δ = 8.05 – 8.01 (m, 2H), 7.62 – 7.54 (m, 1H), 7.48 – 7.40 (m, 2H), 5.86 (dddd, *J* = 16.8, 10.1, 7.7, 6.4 Hz, 1H), 5.25 (td, *J* = 7.5, 3.9 Hz, 1H), 5.21 – 5.03 (m, 2H), 4.43 (ddd, *J* = 5.7, 3.4, 2.2 Hz, 1H), 3.86 (ddd, *J* = 7.4, 6.3, 3.4 Hz, 1H), 3.13 (d, *J* = 6.0 Hz, 1H), 2.87 (d, *J* = 6.5 Hz, 1H), 2.73 (dddt, *J* = 14.7, 6.6, 3.9, 1.4 Hz, 1H), 2.66 – 2.56 (m, 1H), 2.50 (d, *J* = 2.2 Hz, 1H) ppm; ^{13}C NMR (101 MHz, $CDCl_3$): δ = 166.8, 133.6, 133.2, 129.9 (2C), 129.7, 128.6 (2C), 118.7, 81.8, 74.8, 74.7, 73.2, 62.2, 35.2 ppm; IR (film) $\tilde{\nu}$ = 3429, 3294, 3075, 2913, 1702, 1643, 1601, 1451, 1316, 1268, 1112, 711 cm^{-1}; HRMS (ESI): *m/z:* calcd. for $C_{15}H_{16}O_4Na$ $[M+Na]^+$: 283.09390, found: 283.09408.

Compound 11. In a flame-dried flask, diol **S5** (500 mg, 1.92 mmol) was dissolved in CH_2Cl_2 (20 mL) and

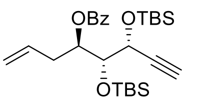

the solution cooled to 0 °C (ice bath). 2,6-Lutidine (670 µL, 5.76 mmol) was added, followed by dropwise addition of TBSOTf (1.06 mL, 4.61 mmol). And the resulting mixture stirred at room temperature 1h before the reaction was quenched with sat. aq. NH_4Cl (10 mL). The organic layer was separated and the aqueous phase was extracted with CH_2Cl_2 (3 × 10 mL). The combined organic layers were washed with brine and dried over Na_2SO_4 before being filtered and concentrated under reduced pressure. The residue was purified by flash chromatography on silica (hexanes/EtOAc, 1:0 to 96:4) to afford the title compound as a colorless oil (874 mg, 93%). $[\alpha]_D^{20}$ = +1.8 (c = 1.43, $CHCl_3$); ^{1}H NMR (400 MHz, $CDCl_3$): δ = 8.08 – 8.01 (m, 2H), 7.59 – 7.50 (m, 1H), 7.47 – 7.38 (m, 2H), 5.84 (dddd, *J* = 16.7, 10.1, 7.6, 6.4 Hz, 1H), 5.53 (dt, *J* = 9.6, 3.0 Hz, 1H), 5.10 (dq, *J* = 17.1, 1.6 Hz, 1H), 4.99 (ddt, *J* = 10.2, 2.3, 1.2 Hz, 1H), 4.47 (d, *J* = 2.3 Hz, 1H), 4.01 (dd, *J* = 5.4, 2.9 Hz, 1H), 2.78 (dddt, *J* = 15.0, 6.3, 3.1, 1.5 Hz, 1H), 2.63 (dddt, *J* = 15.0, 9.8, 7.5, 1.2 Hz, 1H), 2.46 (d, *J* = 2.3

Hz, 1H), 0.93 (s, 9H), 0.92 (s, 9H), 0.14 (s, 3H), 0.11 (s, 3H), 0.07 (s, 3H), 0.05 (s, 3H) ppm; ^{13}C NMR (101 MHz, $CDCl_3$): δ = 165.9, 134.9, 132.9, 130.7, 129.8 (2C), 128.4 (2C), 117.3, 83.2, 76.0, 75.0, 74.8, 65.3, 34.0, 26.01 (3C), 25.98 (3C), 18.39, 18.35, −4.2, −4.3, −4.5, −4.7 ppm; IR (film) $\tilde{\nu}$ = 3310, 2954, 2929, 2887, 2857, 1720, 1472, 1253, 1095, 835, 777, 710 cm^{-1}; HRMS (ESI): *m/z:* calcd. for $C_{27}H_{44}O_4Si_2Na$ $[M+Na]^+$: 511.26716, found: 511.26704.

Compound 13. A two-necked round bottomed flask equipped with a Teflon-coated stirbar and an internal thermometer was charged with L-aspartic acid (10.0 g, 75 mmol) and KBr (40.2 g, 338 mmol). Aq. H_2SO_4 (3 M, 150 mL) was added at ambient temperature and the mixture was stirred until a homogeneous solution had formed (ca. 15 min). This solution was cooled to ca. 5 °C (ice/NaCl bath at −10 °C). A solution of $NaNO_2$ in H_2O (2 M, 65 mL) was added dropwise via an addition funnel over 30 min (the internal temperature should not exceed 10 °C). The resulting solution was stirred with cooling for 3 h (evolution of NO_x-gasses). The mixture was carefully transferred into a separatory funnel and extracted with EtOAc (5 × 100 mL). The combined organic layers were washed with brine (50 mL) and dried over anhydrous $MgSO_4$, filtered, and concentrated under reduced pressure. The title compound was obtained as a white powder (13.2 g, 88%) after drying under high vacuum overnight. ^1H NMR (400 MHz, [D4]-MeOH): δ = 4.56 (dd, J = 8.7, 6.3 Hz, 1H), 3.19 (dd, J = 17.1, 8.7 Hz, 1H), 2.95 ppm (dd, J = 17.2, 6.3 Hz, 1H); ^{13}C NMR (101 MHz, [D4]-MeOH): δ = 173.2, 172.4, 40.8, 40.1 ppm. The spectral data was in agreement with the literature.[2]

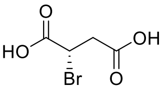

Compound S7. A flame-dried 2-necked round-bottomed flask equipped with a Teflon-coated stirbar was charged with acid **13** (6.50 g, 33.0 mmol) and THF (33 mL). The flask was immersed into an ice/NaCl bath before BH_3·THF (1.0 M in THF, 100 mL) was added slowly by a dropping funnel, maintaining the internal temperature below 0 °C (***Note***: *strong gas evolution*). After complete addition, the mixture was stirred for another 3 h while slowly reaching ambient temperature (***Note***: *in some cases, the formation of a gel was observed during the course of the reaction*). After 3 h, the mixture was cooled to 0 °C (ice bath) and the reaction was carefully quenched by addition of MeOH (50 mL) (***Note***: *strong gas evolution*). The ice bath was removed and the mixture stirred for 30 min. The volatile materials were removed under reduced pressure and the residue was dissolved in MeOH and all volatile materials evaporated to remove $B(OMe)_3$ together with the solvent (2 × 100 mL). Purification of the residue by flash chromatography (hexanes/EtOAc/MeOH, 50:50:0 to 45:45:10) afforded the title compound as a pale-yellow oil (5.28 g, 94% yield). ^1H NMR (400 MHz, $CDCl_3$): δ = 4.36 (m, 1H), 3.94 – 3.78 (m, 4H), 2.21– 2.07 ppm (m, 2H); ^{13}C NMR (101 MHz, $CDCl_3$): δ = 67.3, 60.3, 55.6, 37.8. The spectral data was in agreement with the literature.[2]

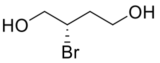

4-Methoxybenzyl Bromide. 4-Methoxybenzyl alcohol (6.2 g, 45 mmol) was dissolved in CH_2Cl_2 (80 mL)

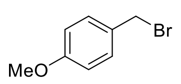

in a flame-dried round-bottomed flask. The mixture was cooled to <0 °C (bath temperature) before PBr_3 (4.2 mL, 45 mmol) was added drop-wise. The mixture was stirred for 20 min at 0 °C before the reaction was quenched by addition of crushed ice. The resulting slurry was partitioned between CH_2Cl_2 and sat. aq. $NaHCO_3$ (***Note***: *strong effervescence*). The cloudy organic layer was separated, the aqueous layer was extracted with CH_2Cl_2 (2 × 50 mL). The combined organic phases were washed with sat. aq. $NaHCO_3$ (2 × 50 mL), dried over $MgSO_4$, filtered and concentrated under reduced pressure (30 °C water bath), yielding the title compound as a pale yellow oil (9.78 g, quant.), which was used immediately without further purification. ^{1}H NMR (400 MHz, $CDCl_3$): δ = 7.35 – 7.30 (m, 2H), 6.89 – 6.84 (m, 2H), 4.51 (s, 2H), 3.81 ppm (s, 3H).[2]

Compound 14. To a suspension of NaH (2.25 g, 93.7 mmol) in THF (35 mL) was added drop-wise a

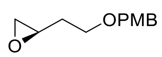

solution of diol **S7** (5.28 g, 31.2 mmol) in THF (30 mL) over 20 min at −30 °C (bath temperture). The mixture was stirred at −30 °C for 15 min until gas-evolution had ceased, before stirring was continued at −5 °C for 1.5 h. A solution of crude PMBBr (8.79 g, 43.7 mmol) in DMF (35 mL) was added dropwise and the resulting mixture was stirred at –5°C for 10 min and for another 1.5 h at ambient temperature. The mixture was cooled to <5 °C and diluted with *tert*-butyl methyl ether (50 mL) before the reaction was quenched by careful addition of semi-sat. aq. NH_4Cl (20 mL). The biphasic mixture was stirred until clear phase separation was reached. The aqueous phase was extracted with *tert*-butyl methyl ether (2 × 100 mL), the combined organic layers were washed with brine (2 × 100 mL), dried over $MgSO_4$, filtered, and concentrated. The resulting crude amber oil was submitted to flash chromatography on silica (hexanes/*tert*-butyl methyl ether, 90:10 to 60:40) to give the title compound as a colorless oil (5.84 g, 89%). $[\alpha]_D^{20}$ = +15.5° (c = 1.39 in $CHCl_3$); ^{1}H NMR (400 MHz, $CDCl_3$): δ = 7.29 – 7.24 (m, 2H), 6.90 – 6.85 (m, 2H), 4.46 (s, 2H), 3.81 (s, 3H), 3.64 – 3.55 (m, 2H), 3.08 – 3.03 (m, 1H), 2.78 (dd, J = 5.0, 4.0 Hz, 1H), 2.52 (dd, J = 5.0, 2.7 Hz, 1H), 1.90 (dddd, J = 14.4, 7.2, 6.2, 4.7 Hz, 1H), 1.77 ppm (dq, J = 14.4, 6.0 Hz, 1H); ^{13}C NMR (101 MHz, $CDCl_3$): δ = 159.4, 130.5, 129.4, 114.0, 72.9, 66.9, 55.4, 50.2, 47.3, 33.1 ppm; IR (film) $\tilde{\nu}$ = 2997, 2928, 2859, 1612, 1586, 1512, 1464, 1442, 1421, 1361, 1302, 1246, 1174, 1090, 1033, 907, 821, 757, 572, 517 cm^{-1}; HRMS (EI): *m/z*: calcd. for $C_{12}H_{16}O_3$ $[M]^{+\bullet}$: 208.10940; found: 208.10918.[2]

(2-Bromoallyl)trimethylsilane (S8). A flame-dried three-necked round-bottomed flask (500 mL)

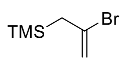

equipped with a reflux condenser, an addition funnel and a big Teflon-coated stirring bar was charged with CuCl (619 mg, 6.25 mmol, 5 mol%), Et_2O (50 mL), and Et_3N (17.0 mL, 125 mmol). The addition funnel was charged with Et_2O (12 mL), 2,3-dibromo-1-propene (25.0 g, 125 mmol), and Cl_3SiH (13.9 mL, 137 mmol). The mixture was sparged with argon for 10 s, before it was added dropwise to the flask at such a rate as to maintain gentle reflux. A white precipitate formed

instantly. Once the addition was complete, the addition funnel was refilled with Et_2O (50 mL), which was also added to the mixture. The mixture was stirred at ambient temperature for 6 h and then cooled to 0 °C (bath temperature). A solution of MeMgBr (3 M in Et_2O, 190 mL) was added dropwise while maintaining gentle reflux. The resulting mixture was stirred at ambient temperature for 12 h. The black suspension that had formed was cooled to 0 °C and the reaction quenched by careful addition of sat. aq. NH_4Cl (170 mL) (***Note***: *strong effervescence!*). The mixture was partitioned between H_2O and Et_2O (100 mL each), the organic phase was washed with H_2O (2 × 60 mL), and the combined aqueous layers were extracted with Et_2O (2 × 60 mL). The combined organic layers were washed with brine (100 mL), dried over $MgSO_4$, and filtered. The mixture was carefully concentrated (> 400 mbar, 40 °C bath temperature). The resulting crude product was distilled under reduced pressure, affording the title compound as a colorless oil (18.2 g, 75%). b.p: 56-58 °C (40 mbar); ^{1}H NMR (400 MHz, $CDCl_3$): δ = 5.31 (dd, J = 1.7, 0.9 Hz, 1H), 5.22 (d, J = 1.5 Hz, 1H), 2.11 (d, J = 1.0 Hz, 2H), 0.12 ppm (s, 9H); ^{13}C NMR (101 MHz, $CDCl_3$): δ = 131.3, 114.1, 33.5, −1.4 ppm.[3]

Compound 16. A two-necked flask (25 mL) equipped with a reflux condenser and a Teflon-coated

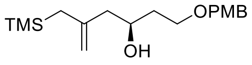

stirbar was charged with magnesium turnings (2.73 g, 112 mmol) and THF (20 mL). The magnesium was activated by addition of 1,2-dibromoethane (3 drops). Next, alkenyl bromide **S8** (10.8 g, 56 mmol) was added dropwise alongside more THF (20 mL, gradual addition to keep the mixture homogeneous). The mixture was stirred at reflux temperature (70 °C, preheated oil bath) for ca. 30 min. The resulting brown solution of the Grignard reagent **15** was cooled to ambient temperature before it was added in portions to a jacketed vessel (250 mL) containing a suspension of CuI (801 mg, 4.21 mmol, 15 mol%) in THF (50 mL) at −50 °C (bath temperature). The residual magnesium was washed with THF (3 × 10 mL). The resulting yellow suspension was stirred for 10 min, before a solution of epoxide **14** (5.84 g, 28.0 mmol) in THF (20 mL) was added dropwise over 10 min. The resulting beige suspension was stirred at −50 °C for 1.5 h and the reaction quenched at this temperature by careful addition of sat. aq. NH_4Cl (100 mL). The biphasic mixture was stirred for 30 min at 0 °C before it was diluted with *tert*-butyl methyl ether (200 mL) and sat. aq. NH_4Cl (100 mL). The organic phase was washed with sat. aq. NH_4Cl (2 × 100 mL) and the combined aqueous layers were extracted with *tert*-butyl methyl ether (2 × 100 mL). The combined extracts were washed with brine (100 mL), dried over $MgSO_4$, filtered, and concentrated in vacuo. The residual yellow oil was purified by flash chromatography on silica (*n*-pentane/*tert*-butyl methyl ether, 90:10 to 80:20) to give the title compound as a colorless oil (8.43 g, 93%). $[\alpha]_D^{20}$ = +5.8° (c = 1.22 in CH_2Cl_2); ^{1}H NMR (400 MHz, CD_2Cl_2): δ = 7.27 – 7.23 (m, 2H), 6.90 – 6.84 (m, 2H), 4.65 (dt, J = 2.2, 1.1 Hz, 1H), 4.63 (dt, J = 2.1, 1.0 Hz, 1H), 4.43 (s, 2H), 3.92 – 3.84 (m, 1H), 3.79 (s, 3H), 3.66 (ddd, J = 9.4, 6.4, 5.3 Hz, 1H), 3.59 (ddd, J = 9.4, 7.2, 5.2 Hz, 1H), 2.51 (d, J = 2.5 Hz, 1H), 2.10 (t, J = 1.1 Hz, 1H), 2.08 (dd, J = 2.7, 1.1 Hz, 1H), 1.80 – 1.71 (m, 1H), 1.71 – 1.62 (m, 1H), 1.58 (dd, J = 13.2, 1.2 Hz, 1H), 1.54 (dd, J =

13.3, 1.1 Hz, 1H), 0.02 ppm (s, 9H); ^{13}C NMR (101 MHz, CD_2Cl_2): δ = 159.7, 145.3, 130.9, 129.6, 114.1, 110.0, 73.1, 68.8, 68.4, 55.6, 46.9, 36.9, 27.0, −1.3 ppm; IR (film) $\tilde{\nu}$ = 3462, 2951, 2912, 2860, 1630, 1613, 1586, 1513, 1464, 1441, 1420, 1362, 1301, 1246, 1173, 1091, 1036, 970, 842, 771, 757, 695, 659, 632, 568, 517, 458, 433, 421 cm^{-1}; HRMS (ESI): *m/z*: calcd. for $C_{18}H_{30}O_3NaSi$ $[M+Na]^+$: 345.18564; found: 345.18562.

Compound 17. A solution of allylsilane **16** (2.30 g, 7.13 mmol) in THF (35 mL) was cooled to −78 °C

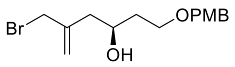

(bath temperature) before NBS (1.40 g, 7.85 mmol) was added. The mixture was stirred in the dark for 2 h at this temperature before it was diluted with *tert*-butyl methyl ether (20 mL) and the reaction quenched by addition of a mixture of water, sat. aq. $Na_2S_2O_3$, and sat. aq. $NaHCO_3$ (1:1:1, 10 mL). The mixture was stirred for 10 min and warmed to ambient temperature. The aq. layer was extracted with *tert*-butyl methyl ether (2 × 20 mL), the combined organic phases were washed with brine (20 mL), dried over $MgSO_4$, filtered and concentrated. The residue was purified by flash chromatography on silica (*n*-pentane/*tert*-butyl methyl ether, 70:30) to afford the title compound as a pale-yellow oil (2.00 g, 85%). $[\alpha]_D^{20}$ = −10.2° (c = 1.05 in CH_2Cl_2); ^{1}H NMR (400 MHz, $CDCl_3$): δ = 7.28 – 7.22 (m, 2H), 6.90 – 6.85 (m, 2H), 5.26 (q, *J* = 1.1 Hz, 1H), 5.04 (q, *J* = 1.2 Hz, 1H), 4.44 (s, 2H), 4.06 (t, *J* = 1.0 Hz, 2H), 4.02 – 3.94 (m, 1H), 3.79 (s, 3H), 3.69 (ddd, *J* = 9.3, 5.7, 4.9 Hz, 1H), 3.65 – 3.59 (m, 1H), 2.81 (d, *J* = 2.9 Hz, 1H), 2.39 (ddd, *J* = 14.5, 4.5, 1.2 Hz, 1H), 2.30 (ddd, *J* = 14.5, 8.4, 1.0 Hz, 1H), 1.80 – 1.65 ppm (m, 2H); ^{13}C NMR (101 MHz, $CDCl_3$): δ = 159.7, 143.6, 130.7, 129.7, 117.7, 114.1, 73.3, 69.8, 69.1, 55.6, 41.7, 37.6, 36.8 ppm; IR (film) $\tilde{\nu}$ = 3448, 2936, 2861, 1612, 1586, 1513, 1463, 1440, 1362, 1302, 1247, 1211, 1174, 1086, 1033, 914, 821, 725, 637, 595, 517 cm^{-1}; HRMS (ESI): *m/z*: calcd. for $C_{15}H_{21}O_3BrNa$ $[M+Na]^+$: 351.05664; found: 351.05644.

Compound S9. A flame-dried Schlenk flask was charged with alkyne **11** (40 mg, 82 µmol), allyl bromide

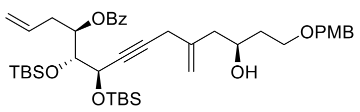

17 (29.6 mg, 90 µmol), and DMF (1 mL). CuI (1.6 mg, 8.2 µmol, 10 mol%), K_2CO_3 (11 mg, 82 µmol), and Na_2SO_3 (5.2 mg, 41 µmol) were added, followed by DBU (5 µL, 33 µmol, 30 mol%). The mixture was stirred for 2.5 h at room temperature before it was diluted with *tert*-butyl methyl ether (8 mL) and sat. aq. NH_4Cl (3 mL). The organic phase was washed with sat. aq. NH_4Cl (2 mL) and brine (2 mL), dried over $MgSO_4$, filtered and concentrated in vacuo. The residue was purified by flash chromatography on silica (hexanes/*tert*-butyl methyl ether, 90:10 to 70:30) to give the title compound as a colorless oil (57 mg, 94%). $[\alpha]_D^{20}$ = −5.3° (c = 1.84 in CH_2Cl_2); ^{1}H NMR (600 MHz, CD_2Cl_2): δ = 8.04 – 8.00 (m, 2H), 7.58 – 7.53 (m, 1H), 7.46 – 7.41 (m, 2H), 7.27 – 7.22 (m, 2H), 6.89 – 6.85 (m, 2H), 5.84 (dddd, *J* = 16.8, 10.2, 7.6, 6.4 Hz, 1H), 5.53 (dt, *J* = 9.8, 2.9 Hz, 1H), 5.19 (q, *J* = 1.6 Hz, 1H), 5.08 (ddt, *J* = 17.1, 2.2, 1.4 Hz, 1H), 4.97 (ddt, *J* = 10.1, 2.2, 1.1 Hz, 1H), 4.92 (dt, *J* = 1.6, 1.1 Hz, 1H), 4.49 (dt, *J* = 5.5, 2.1 Hz, 1H), 4.43 (s, 2H), 3.98 (dd, *J* = 5.5, 2.7 Hz, 1H), 3.93 (dddd, *J* = 10.8, 7.8, 5.4, 2.8 Hz, 1H), 3.79 (s, 3H), 3.67 (ddd, *J* =

9.4, 6.1, 5.1 Hz, 1H), 3.61 (ddd, J = 9.4, 7.6, 4.9 Hz, 1H), 3.02 (q, J = 1.6 Hz, 2H), 2.79 – 2.72 (m, 1H), 2.62 (dddt, J = 15.0, 9.9, 7.6, 1.2 Hz, 1H), 2.28 – 2.19 (m, 2H), 1.78 – 1.64 (m, 2H), 0.93 (s, 9H), 0.92 (s, 9H), 0.13 (s, 3H), 0.10 (s, 3H), 0.07 (s, 3H), 0.05 ppm (s, 3H); ^{13}C NMR (151 MHz, CD_2Cl_2): δ = 166.0, 159.7, 141.9, 135.5, 133.1, 131.1, 130.8, 130.0, 129.7, 128.7, 117.1, 114.1, 113.9, 84.4, 82.1, 76.8, 75.1, 73.2, 69.2, 68.9, 66.0, 55.6, 44.3, 36.8, 34.4, 26.7, 26.1, 26.1, 18.6, 18.5, −4.2, −4.4, −4.4, −4.6 ppm; IR (film) $\tilde{\nu}$ = 2952, 2929, 2856, 1719, 1513, 1361, 1248, 1174, 1092, 1070, 1037, 1027, 1005, 980, 939, 879, 834, 777, 711, 673 cm^{-1}; HRMS (ESI): *m/z*: calcd. for $C_{42}H_{64}O_7Si_2Na$ $[M+Na]^+$: 759.40828; found: 759.40779.

Compound 18. A flame-dried Schlenk flask was charged with compound **S9** (18 mg, 24 µmol) and CH_2Cl_2 (0.5 mL). The mixture was cooled to −78 °C before DIBAL-H (1 M in THF, 0.1 mL, 0.1 mmol) was added dropwise. After 2 h, the reaction was quenched by dropwise addition of EtOAc (0.1 mL), followed by sat. aq. Rochelle salt (0.5 mL). The biphasic mixture was stirred vigorously at ambient temperature for 30 min and the aqueous phase was extracted with *tert*-butyl methyl ether (3 × 1 mL). The combined organic layers were passed through $MgSO_4$ and concentrated. The residue was purified by flash chromatography on silica (hexanes/EtOAc, 90:10 to 85:15), affording the title compound as a colorless oil (15 mg, 97%). $[\alpha]_D^{20}$ = +16.0° (c = 1.51 in CH_2Cl_2); ^{1}H NMR (400 MHz, $CDCl_3$): δ = 7.27 – 7.22 (m, 2H), 6.90 – 6.85 (m, 2H), 5.94 (dddd, J = 16.6, 10.2, 7.6, 6.3 Hz, 1H), 5.21 (q, J = 1.6 Hz, 1H), 5.17 – 5.11 (m, 1H), 5.11 – 5.07 (m, 1H), 4.92 (q, J = 1.3 Hz, 1H), 4.56 (dt, J = 4.2, 2.0 Hz, 1H), 4.45 (s, 2H), 4.09 (dddd, J = 9.1, 7.7, 3.3, 1.3 Hz, 1H), 3.95 (pent, J = 6.4 Hz, 1H), 3.80 (s, 3H), 3.69 (dt, J = 9.4, 5.4 Hz, 1H), 3.66 – 3.58 (m, 2H), 3.48 (dd, J = 7.7, 4.4 Hz, 1H), 3.01 (q, J = 1.6 Hz, 2H), 2.89 (s, 1H), 2.51 (dddd, J = 14.2, 6.1, 3.0, 1.4 Hz, 1H), 2.27 – 2.21 (m, 2H), 2.12 (dddt, J = 14.2, 8.9, 7.6, 1.1 Hz, 1H), 1.78 – 1.69 (m, 2H), 0.90 (s, 9H), 0.89 (s, 9H), 0.15 (s, 3H), 0.13 (s, 3H), 0.09 (s, 3H), 0.08 ppm (s, 3H); ^{13}C NMR (101 MHz, $CDCl_3$): δ = 159.4, 141.1, 135.9, 130.2, 129.5, 116.8, 114.1, 114.0, 84.1, 80.7, 77.4, 75.0, 73.1, 72.7, 69.1, 68.7, 68.3, 55.4, 44.1, 38.0, 36.4, 26.4, 25.9, 25.8, 18.2, 18.2, −4.3, −4.4, −4.6, −5.1 ppm; IR (film) $\tilde{\nu}$ = 2953, 2930, 2886, 2857, 1613, 1514, 1463, 1415, 1391, 1361, 1302, 1250, 1173, 1091, 1040, 1006, 938, 883, 836, 778 cm^{-1}; HRMS (ESI): *m/z*: calcd. for $C_{35}H_{60}O_6Si_2Na$ $[M+Na]^+$: 655.38206; found: 655.38154.

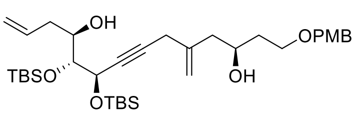

Compound 21. A flame-dried Schlenk flask was charged with diol **18** (15 mg, 24 µmol) and CH_2Cl_2 (0.5 mL). The colorless solution was cooled to −78 °C (bath temperature) before camphorsulfonic acid (1 mg, 5 µmol, 20 mol%) and the gold complex **cat1** (1.8 mg, 2.4 µmol, 10 mol%) were added. After 3 h at −78 °C, cooling was suspended and sirring continued at ambient temperature for precisely 20 min, after which the mixture was diluted with *n*-pentane (1 mL) and filtered through a pipette packed with silica (eluting with *tert*-butyl methyl ether). The combined filtrates were concentrated and the residue subjected to

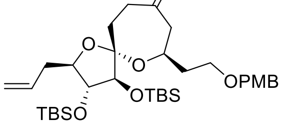

flash chromatography on silica (*n*-pentane/*tert*-butyl methyl ether, 95:5 to 85:15) to furnish the title compound as a colorless oil (12 mg, 80%). $[\alpha]_D^{20}$ = +43.0° (c = 1.18 in CH_2Cl_2); ^{1}H NMR (400 MHz, $CDCl_3$): δ = 7.29 – 7.24 (m, 2H), 6.90 – 6.84 (m, 2H), 5.81 (ddt, *J* = 17.2, 10.2, 6.9 Hz, 1H), 5.09 (dq, *J* = 17.2, 1.6 Hz, 1H), 5.03 (ddt, *J* = 10.2, 2.2, 1.2 Hz, 1H), 4.65 (s, 1H), 4.59 (t, *J* = 2.0 Hz, 1H), 4.44 (d, *J* = 11.3 Hz, 1H), 4.39 (d, *J* = 11.3 Hz, 1H), 4.08 (dddd, *J* = 10.5, 8.2, 4.3, 2.4 Hz, 1H), 3.91 – 3.85 (m, 2H), 3.80 (s, 3H), 3.77 (dd, *J* = 3.4, 1.3 Hz, 1H), 3.66 – 3.51 (m, 2H), 2.38 – 2.17 (m, 6H), 2.13 – 2.02 (m, 1H), 1.80 – 1.63 (m, 3H), 0.91 (s, 9H), 0.89 (s, 9H), 0.11 (s, 3H), 0.09 (s, 6H), 0.06 ppm (s, 3H).; ^{13}C NMR (101 MHz, $CDCl_3$): δ = 159.2, 149.8, 135.2, 131.0, 129.4, 117.0, 113.9, 111.5, 110.5, 84.4, 83.5, 83.2, 72.8, 67.5, 67.5, 55.4, 44.8, 38.7, 37.5, 32.3, 31.7, 25.9, 18.1, 18.1, −3.9, −4.0, −4.1, −4.6 ppm; IR (film) $\tilde{\nu}$ = 2952, 2929, 2857, 1513, 1463, 1361, 1249, 1170, 1099, 1038, 1005, 912, 886, 868, 835, 776, 671 cm^{-1}; HRMS (ESI): *m/z*: calcd. for $C_{35}H_{60}O_6Si_2Na$ $[M+Na]^+$: 655.38206; found: 655.38281.

Compound 22. A flame-dried Schlenk flask was charged with alkyne **10** (3.00 g, 6.27 mmol), DMF

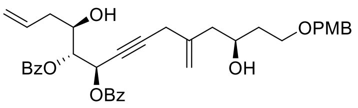

(31 mL), and allyl bromide **17** (2.48 g, 7.52 mmol). CuI (298 mg, 1.57 mmol, 25 mol%), K_2CO_3 (866 mg, 6.27 mmol), and Na_2SO_3 (395 mg, 3.13 mmol) were added, followed by DBU (0.1 mL, 0.63 mmol, 10 mol%). The mixture was stirred for 2.5 h at ambient temperature. The grey opaque mixture was diluted with *tert*-butyl methyl ether (50 mL) and sat. aq. NH_4Cl (15 mL), and the organic phase was washed with sat. aq. NH_4Cl (15 mL). The aqueous layers were extracted with *tert*-butyl methyl ether (2 × 20 mL), the combined organic phases were washed with H_2O (10 mL) and brine (15 mL), dried over $MgSO_4$, filtered and concentrated *in vacuo*.

The resulting crude yellow oil was dissolved in CH_2Cl_2/MeOH (80:20, 50 mL) before camphorsulfonic acid (146 mg, 0.627 mmol, 10 mol%) was added. The mixture was stirred for 24 h at ambient temperature and then concentrated under reduced pressure (30 °C bath temperature). Purification of the residue by flash chromatography on silica (*n*-pentane/*tert*-butyl methyl ether, 50:50) afforded the title compound as a pale-yellow viscous oil (3.16 g, 82%). $[\alpha]_D^{20}$ = +18.5° (c = 1.05 in $CHCl_3$); ^{1}H NMR (600 MHz, $CDCl_3$): δ = 8.09 – 8.05 (m, 4H), 7.59 – 7.53 (m, 2H), 7.46 – 7.41 (m, 4H), 7.25 – 7.21 (m, 2H), 6.89 – 6.84 (m, 2H), 6.10 (dt, *J* = 4.7, 2.0 Hz, 1H), 5.87 (dddd, *J* = 17.1, 10.4, 7.4, 6.7 Hz, 1H), 5.52 (dd, *J* = 6.8, 4.7 Hz, 1H), 5.18 – 5.15 (m, 1H), 5.14 (m, 1H), 5.03 (m, 1H), 4.81 (m, 1H), 4.42 (s, 2H), 4.06 (m, 1H), 3.88 (m, 1H), 3.79 (s, 3H), 3.63 (ddd, *J* = 9.3, 5.8, 5.1 Hz, 1H), 3.59 – 3.55 (m, 1H), 2.94 (m, 2H), 2.88 (br s, 1H), 2.62 (br s, 1H), 2.46 (dddt, *J* = 14.4, 6.5, 3.5, 1.3 Hz, 1H), 2.34 (dddt, *J* = 14.4, 8.6, 7.4, 1.1 Hz, 1H), 2.16 (br s, 1H), 2.15 (t, *J* = 1.4 Hz, 1H), 1.71 – 1.63 ppm (m, 2H); ^{13}C NMR (151 MHz, $CDCl_3$): δ = 166.0, 165.9, 159.4, 140.4, 133.9, 133.6, 133.5, 130.2, 130.1, 130.0, 129.7, 129.5, 129.5, 128.6, 128.6, 119.0, 114.4, 114.0, 85.7, 76.8, 76.6, 73.1, 69.0, 69.0, 68.6, 64.1, 55.4, 43.8, 37.4, 36.2, 26.3 ppm; IR (film) $\tilde{\nu}$ = 3489, 2935, 1723, 1612, 1585, 1513, 1451, 1419, 1315, 1247, 1176, 1093, 1069, 1026, 904,

821, 711, 687, 567, 517 cm^{-1}; HRMS (ESI): *m/z*: calcd. for $C_{37}H_{40}O_8Na$ [M+Na]$^+$: 635.26154; found: 635.26101.

Compound 23. A flame-dried Schlenk flask was charged with diol **22** (480 mg, 0.783 mmol) and CH_2Cl_2 (28 mL). The colorless solution was cooled to −78 °C before camphorsulfonc acid (18 mg, 0.078 mmol, 10 mol%) and a solution of the gold complex **cat1** (12 mg, 0.016 mmol, 2 mol%) in CH_2Cl_2 (2 mL) were added. The pale-yellow solution was stirred at −78 °C for 2 h. Next, the cooling bath was removed and the mixture stirred for 4.5 h, during which the progress of the reaction was monitored by ^{1}H NMR. After 4.5 h, the mixture was diluted with *n*-pentane (30 mL) and then filtered through a plug of silica, eluting with *tert*-butyl methyl ether. The filtrate was concentrated and the residue purified by flash chromatography on silica (*n*-pentane/*tert*-butyl methyl ether, 90:10 to 80:20) to afford the title compound as a colorless viscous oil (365 mg, 76%) alongside 8-*epi*-**23** (colorless viscous oil, 43 mg, 9%). *Analytical and spectral data of the major isomer* (**23**): $[\alpha]_D^{20}$ = +22.8° (c = 1.04 in $CHCl_3$); ^{1}H NMR (600 MHz, $CDCl_3$): δ = 7.96 – 7.92 (m, 4H), 7.52 – 7.47 (m, 2H), 7.39 – 7.34 (m, 4H), 7.29 – 7.23 (m, 2H), 6.84 – 6.78 (m, 2H), 5.80 – 5.72 (m, 1H), 5.70 (ddd, *J* = 11.4, 9.6, 5.4 Hz, 1H), 5.27 (t, *J* = 9.7 Hz, 1H), 4.97 – 4.92 (m, 2H), 4.79 (m, 1H), 4.77 (m, 1H), 4.54 (d, *J* = 11.4 Hz, 1H), 4.46 (d, *J* = 11.4 Hz, 1H), 3.90 – 3.85 (m, 1H), 3.85 – 3.80 (m, 1H), 3.79 – 3.75 (m, 1H), 3.74 (s, 3H), 3.65 – 3.60 (m, 1H), 2.51 (dd, *J* = 12.6, 5.4 Hz, 1H), 2.37 (dd, *J* = 13.6, 1.3 Hz, 1H), 2.33 – 2.23 (m, 4H), 2.02 – 1.96 (m, 1H), 1.90 – 1.80 ppm (m, 3H); ^{13}C NMR (151 MHz, $CDCl_3$): δ = 166.0, 166.0, 159.3, 140.6, 133.8, 133.3, 133.1, 130.7, 130.0, 129.8, 129.8, 129.3, 128.5, 128.5, 117.6, 113.9, 110.8, 97.9, 73.6, 73.0, 71.1, 69.6, 67.4, 66.4, 55.4, 43.4, 40.7, 39.9, 36.2, 36.0 ppm; IR (film) $\tilde{\nu}$ = 3072, 2951, 2857, 1725, 1656, 1613, 1585, 1513, 1451, 1361, 1315, 1277, 1248, 1197, 1175, 1096, 1070, 1027, 991, 918, 893, 821, 711, 687, 515 cm^{-1}; HRMS (ESI): *m/z*: calcd. for $C_{37}H_{40}O_8Na$ [M+Na]$^+$: 635.26154; found: 635.26196.

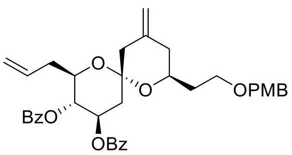

Analytical and spectral data of the minor isomer (8-*epi*-**23**): $[\alpha]_D^{20}$ = −23.4 (c = 1.93 in $CHCl_3$); ^{1}H NMR (400 MHz, $CDCl_3$): δ = 8.02 – 7.96 (m, 4H), 7.57 – 7.49 (m, 2H), 7.45 – 7.37 (m, 4H), 7.28 – 7.22 (m, 2H), 6.87 – 6.82 (m, 2H), 5.94 (ddt, *J* = 17.2, 10.3, 6.8 Hz, 1H), 5.41 (ddd, *J* = 7.8, 3.4, 1.7 Hz, 2H), 5.11 – 5.03 (m, 2H), 5.03 – 5.00 (m, 1H), 4.96 – 4.93 (m, 1H), 4.44 (s, 2H), 3.96 – 3.90 (m, 1H), 3.90 – 3.82 (m, 1H), 3.78 (s, 3H), 3.64 (ddd, *J* = 9.4, 8.0, 5.2 Hz, 1H), 3.60 – 3.52 (m, 1H), 2.89 (d, *J* = 13.2 Hz, 1H), 2.84 – 2.77 (m, 1H), 2.50 – 2.44 (m, 2H), 2.41 (d, *J* = 13.2 Hz, 1H), 2.32 (dd, *J* = 13.5, 3.0 Hz, 1H), 2.18 – 2.09 (m, 1H), 2.00 (ddt, *J* = 13.6, 8.1, 5.3 Hz, 1H), 1.84 – 1.71 ppm (m, 2H).; ^{13}C NMR (101 MHz, $CDCl_3$): δ = 166.0, 165.7, 159.3, 140.6, 133.8, 133.3, 133.3, 130.7, 129.8, 129.7, 129.7, 129.5, 128.5, 128.5, 117.6, 113.9, 112.3, 100.0, 73.3, 72.8, 72.4, 71.3, 71.0, 66.4, 55.4, 41.0, 39.5, 36.7, 36.2, 36.2 ppm; IR (film) $\tilde{\nu}$ = 2948, 1722, 1612, 1513, 1451, 1364, 1315, 1275, 1248, 1176, 1093, 1070, 1028, 1000, 913, 848, 821, 710, 687, 577 cm^{-1}; HRMS (ESI): *m/z*: calcd. for $C_{37}H_{40}O_8Na$ [*M*+Na$^+$]: 635.26154; found: 635.26169.

Compound 24. A flame-dried Schlenk flask was charged with $Pt(dba)_3$ (27 mg, 0.030 mmol, 5 mol%), ligand (*R,R*)-**30** (41 mg, 0.046 mmol, 7.5 mol%) and $B_2(pin)_2$ (freshly recrystallized from *n*-pentane, 231 mg, 0.911 mmol). Ar was passed over the mixture for 10 min before THF (2 mL) was added. The resulting black/violet solution was stirred at 80 °C for 30 min (pre-heated oil bath). The now brown solution was cooled to ambient temperature before a solution of spiroketal **23** (372 mg, 0.607 mmol) in THF (4 mL) was introduced. The mixture was sparged with Ar for 1 min and then stirred at 60 °C (bath temperature) for 3 h. The mixture was allowed to cool before sodium perborate tetrahydrate (374 mg, 2.43 mmol) was added, followed by H_2O (6 mL). The biphasic mixture was stirred vigorously overnight (ca. 16 h), then partitioned between EtOAc (10 mL) and sat. aq. $Na_2S_2O_3$ (5 mL). The biphasic mixture was stirred for 5 min, the organic layer was separated, and the aqueous phase was extracted with EtOAc (2 × 10 mL). The combined organic extracts were washed with brine (10 mL), dried over $MgSO_4$, filtered, and concentrated. Purification of the residue by flash chromatography on silica (*n*-pentane/*tert*-butyl methyl ether, 50:50 to 30:70) afforded the title compound as a white amorphous solid (319 mg, 81%, d.r. = 10:1). $[\alpha]_D^{20}$ = +54.4° (c = 1.40 in $CHCl_3$); ^1H NMR (400 MHz, $CDCl_3$): δ = 8.01 – 7.95 (m, 2H), 7.95 – 7.89 (m, 2H), 7.56 – 7.45 (m, 2H), 7.42 – 7.32 (m, 4H), 7.22 – 7.16 (m, 2H), 6.72 – 6.64 (m, 2H), 5.73 (ddd, *J* = 11.4, 9.6, 5.3 Hz, 1H), 5.26 (t, *J* = 9.7 Hz, 1H), 4.93 – 4.89 (m, 1H), 4.85 – 4.81 (m, 1H), 4.50 (d, *J* = 11.0 Hz, 1H), 4.45 (d, *J* = 11.0 Hz, 1H), 4.12 (ddd, *J* = 10.9, 9.7, 2.3 Hz, 1H), 3.94 – 3.84 (m, 2H), 3.69 (s, 3H), 3.67 – 3.61 (m, 2H), 3.55 – 3.49 (m, 1H), 3.23 (ddd, *J* = 10.5, 5.2, 2.5 Hz, 1H), 3.15 (dt, *J* = 11.2, 5.7 Hz, 1H), 2.57 (dd, *J* = 12.7, 5.3 Hz, 1H), 2.39 – 2.25 (m, 3H), 2.08 – 1.76 (m, 5H), 1.66 (ddd, *J* = 14.6, 11.0, 9.9 Hz, 1H), 1.47 ppm (br d, *J* = 14.5 Hz, 1H); ^{13}C NMR (101 MHz, $CDCl_3$): δ = 166.0, 165.9, 159.3, 140.9, 133.5, 133.3, 130.5, 129.8, 129.8, 129.5, 129.3, 128.6, 128.5, 113.7, 111.9, 98.7, 73.6, 73.1, 71.7, 71.5, 70.3, 67.7, 66.6, 66.1, 55.3, 43.6, 40.2, 39.9, 35.6, 34.3 ppm; IR (film) $\tilde{\nu}$ = 2923, 1722, 1612, 1513, 1451, 1366, 1315, 1272, 1248, 1174, 1096, 1070, 1027, 988, 898, 823, 807, 755, 711, 688 cm^{-1}; HRMS (ESI): *m/z*: calcd. for $C_{37}H_{42}O_{10}Na$ $[M+Na]^+$: 669.26702; found: 669.26672.

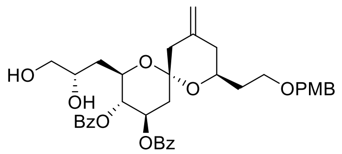

Preparation of the (*R,R*)- and (*S,S*)-MTPA Esters of Diol 24. A screw-cap vial was charged with a solution of diol **24** (5.0 mg, 7.7 µmol) in CH_2Cl_2 (0.2 mL). Pyridine (0.1 mL) was added and the mixture was cooled in an ice/water bath while (*S*)-(+)-MTPA-Cl (15 µL, 77 µmol) was added. The vial was sealed with a screw-cap and parafilm. The mixture was warmed to ambient temperature and stirred overnight before it was diluted with hexanes (0.5 mL). The resulting mixture was directly submitted to flash chromatography on silica (hexanes/EtOAc, 80:20), affording the (*R,R*)-MTPA ester as a colorless film (7.3 mg, 87%). ^1H NMR (600 MHz, $CDCl_3$): δ = 7.94 – 7.89 (m, 4H), 7.52 – 7.47 (m, 2H), 7.45 – 7.41 (m, 2H), 7.40 – 7.23 (m, 12H), 7.23 – 7.20 (m, 2H), 6.77 – 6.74 (m, 2H), 5.72 (ddd, *J* = 11.5, 9.5, 5.3 Hz, 1H), 5.60 (ddt, *J* = 9.2, 6.4, 2.4 Hz, 1H), 5.28 (t, *J* =

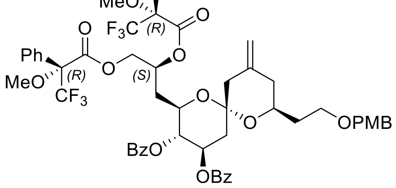

9.6 Hz, 1H), 4.77 (m, 2H), 4.69 (dd, J = 12.6, 2.0 Hz, 1H), 4.47 (d, J = 11.4 Hz, 1H), 4.44 (d, J = 11.4 Hz, 1H), 4.10 (dd, J = 12.7, 6.4 Hz, 1H), 3.98 (td, J = 9.4, 3.1 Hz, 1H), 3.78 – 3.66 (m, 5H), 3.60 (ddd, J = 9.2, 6.2, 4.6 Hz, 1H), 3.36 (s, 3H), 3.32 (s 3H), 2.52 (dd, J = 12.7, 5.3 Hz, 1H), 2.40 – 2.34 (m, 1H), 2.27 – 2.23 (m, 1H), 2.19 (dt, J = 13.1, 2.3 Hz, 1H), 2.04 (ddd, J = 14.3, 9.5, 3.1 Hz, 1H), 1.99 (t, J = 12.4 Hz, 1H), 1.88 – 1.74 ppm (m, 4H).

The analogous reaction of **24** (5.0 mg, 7.7 µmol) with (*R*)-(−)-MTPA-Cl (15 µL, 77 µmol) afforded the (*S*,*S*)-MTPA ester as a colorless film (7.9 mg, 94%). ^{1}H NMR (600 MHz, $CDCl_3$): δ = 7.94 – 7.88 (m, 4H), 7.52 – 7.47 (m, 2H), 7.46 – 7.41 (m, 2H), 7.40 – 7.28 (m, 12H), 7.28 – 7.24 (m, 2H), 7.23 – 7.20 (m, 2H), 6.77 – 6.74 (m, 2H), 5.69 (ddd, J = 11.4, 9.5, 5.2 Hz, 1H), 5.57 (tt, J = 9.6, 2.3 Hz, 1H), 5.26 (t, J = 9.7 Hz, 1H), 4.82-4.79 (m, 2H), 4.73 (dd, J = 12.8, 2.0 Hz, 1H), 4.46 (d, J = 11.4 Hz, 1H), 4.42 (d, J = 11.4 Hz, 1H), 4.18 (dd, J = 12.8, 7.3 Hz, 1H), 3.93 (td, J = 9.3, 3.1 Hz, 1H), 3.77 – 3.65 (m, 5H), 3.55 (ddd, J = 9.3, 5.9, 4.6 Hz, 1H), 3.40 (s, 3H), 3.26 (s, 3H), 2.50 (dd, J = 12.7, 5.3 Hz, 1H), 2.37 (dd, J = 13.7, 1.4 Hz, 1H), 2.27 – 2.22 (m, 2H), 2.00 (t, J = 12.1 Hz, 1H), 1.90 (ddd, J = 14.6, 9.5, 3.1 Hz, 1H), 1.85 – 1.69 (m, 4H).

Table S5. Determination of absolute configuration of the newly set stereogenic center in diol **24** by Mosher ester analysis;[4] the recorded NMR data ($CDCl_3$) suggest that it is (*S*)-configured (benthol A numbering scheme).

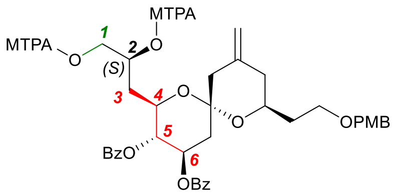

##	$\delta_{H\,NMR}$ (*S*-ester) (ppm)	$\delta_{H\,NMR}$ (*R*-ester) (ppm)	$\Delta\delta$ (δ_S -δ_R, ppm)
1a	4.18	4.10	0.08
1b	4.73	4.69	0.04
2	5.57	5.60	−0.03
3a	1.78	1.85	−0.07
3b	1.90	2.04	−0.14
4	3.93	3.98	−0.05
5	5.26	5.28	−0.02
6	5.69	5.72	−0.03

Compound S10. A flame-dried Schlenk flask was charged with diol **24** (351 mg, 0.543 mmol) and CH_2Cl_2 (5.8 mL). The solution was cooled to 0 °C (bath temperature) before 2,6-lutidine (0.63 mL, 5.4 mmol) was introduced followed by dropwise addition of TBSOTf (0.37 mL, 1.6 mmol). The mixture was stirred at 0°C for 1 h before it was diluted with *tert*-butyl methyl ether (10 mL) and the reaction was quenched with sat.

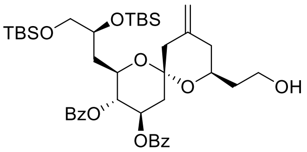

aq. NH_4Cl (10 mL). The aqueous layer was extracted with *tert*-butyl methyl ether (2 × 10 mL), the combined organic phases were washed with aq. citric acid (5 wt%, 2 × 10 mL) and brine (10 mL), dried over $MgSO_4$, filtered, and concentrated *in vacuo*.

Note*: Purification of the silylated and PMB-protected intermediate is not necessary, but analytically pure samples can be obtained by flash chromatography on silica (n-*pentane/*tert*-butyl methyl ether, 100:0 to 90:10*).* $[\alpha]_D^{20}$ = +10.7° (c = 1.66 in $CHCl_3$); ^{1}H NMR (400 MHz, $CDCl_3$): δ = 7.97 – 7.90 (m, 4H), 7.53 – 7.43 (m, 2H), 7.38 – 7.32 (m, 4H), 7.32 – 7.27 (m, 2H), 6.90 – 6.84 (m, 2H), 5.68 (ddd, *J* = 11.4, 9.6, 5.3 Hz, 1H), 5.24 (t, *J* = 9.7 Hz, 1H), 4.81 (q, *J* = 1.9 Hz, 1H), 4.76 (q, *J* = 1.9 Hz, 1H), 4.57 (d, *J* = 11.6 Hz, 1H), 4.46 (d, *J* = 11.6 Hz, 1H), 3.99 – 3.88 (m, 2H), 3.77 (s, 4H), 3.69 – 3.62 (m, 2H), 3.61 – 3.51 (m, 2H), 2.51 (dd, *J* = 12.5, 5.3 Hz, 1H), 2.34 (dd, *J* = 13.6, 1.3 Hz, 1H), 2.30 – 2.20 (m, 2H), 2.07 – 1.75 (m, 5H), 1.63 (ddd, *J* = 14.3, 10.4, 2.2 Hz, 1H), 0.83 (s, 9H), 0.80 (s, 9H), 0.01 (s, 3H), 0.00 (s, 3H), −0.03 (s, 3H), −0.04 ppm (s, 3H); ^{13}C NMR (101 MHz, $CDCl_3$): δ = 166.1, 166.0, 159.3, 140.5, 133.2, 133.1, 130.7, 130.0, 129.9, 129.8, 129.7, 129.5, 128.5, 128.4, 113.9, 111.0, 98.1, 77.4, 73.9, 72.9, 71.0, 70.6, 68.5, 67.5, 67.0, 66.5, 55.4, 43.6, 40.7, 39.7, 36.4, 35.9, 26.1, 26.1, 18.4, 18.4, −4.3, −4.8, −5.1, −5.1 ppm; IR (film) $\tilde{\nu}$ = 2952, 2926, 2855, 1727, 1513, 1463, 1452, 1315, 1275, 1248, 1174, 1093, 1069, 1027, 992, 889, 833, 777, 709, 686 cm^{-1}; HRMS (ESI): *m/z*: calcd. for $C_{49}H_{70}O_{10}Si_2Na$ $[M+Na]^+$: 897.43998; found: 897.44126.

The resulting pale-yellow oil was dissolved in CH_2Cl_2 (10 mL) and H_2O (0.5 mL) and DDQ (156 mg, 0.687 mmol) was added in one portion. The mixture turned black and was stirred at ambient temperature for 1 h. For work up, it was diluted with *tert*-butyl methyl ether (20 mL) and the reaction quenched with sat. aq. $NaHCO_3$ (20 mL). The biphasic mixture was stirred until all solids had dissolved (15 min). The mixture was then partitioned between *tert*-butyl methyl ether (20 mL) and H_2O (10 mL) and the organic phase was washed with sat. aq. $NaHCO_3$ (2 × 10 mL). The aqueous layer was extracted with *tert*-butyl methyl ether (2 × 20 mL), and the combined organic phases were washed with brine (10 mL), dried over $MgSO_4$, filtered and concentrated. The residue was purified by flash chromatography on silica (*n*-pentane/*tert*-butyl methyl ether, 90:10 to 80:20) to furnish the title compound as an amorphous white solid (347 mg, 84%). $[\alpha]_D^{20}$ = +21.3° (c = 1.11 in $CHCl_3$); ^{1}H NMR (400 MHz, $CDCl_3$): δ = 7.98 – 7.94 (m, 2H), 7.92 – 7.87 (m, 2H), 7.53 – 7.44 (m, 2H), 7.39 – 7.31 (m, 4H), 5.62 (ddd, *J* = 11.5, 9.6, 5.3 Hz, 1H), 5.23 (t, *J* = 9.8 Hz, 1H), 4.85 – 4.82 (m, 1H), 4.80 – 4.77 (m, 1H), 3.99 (td, *J* = 10.1, 1.6 Hz, 1H), 3.95 – 3.82 (m, 4H), 3.60 – 3.55 (m, 2H), 2.52 (dd, *J* = 12.7, 5.3 Hz, 1H), 2.37 (d, *J* = 13.3 Hz, 1H), 2.28 (s, 1H), 2.27 – 2.20 (m, 2H), 2.11 (t, *J* = 12.5 Hz, 1H), 1.92 – 1.78 (m, 4H), 1.72 – 1.63 (m, 1H), 0.84 (s, 9H), 0.82 (s, 9H), 0.01 (s, 6H), 0.00 (s, 3H), −0.02 ppm (s, 3H); ^{13}C NMR (101 MHz, $CDCl_3$): δ = 166.1, 165.9, 140.2, 133.3, 133.1, 130.0, 129.9, 129.8, 129.6, 128.5, 128.4, 111.3, 98.4, 73.6, 71.0, 70.9, 69.3, 68.0, 67.3, 59.8, 43.5, 40.7, 39.6, 38.3, 36.5, 26.2, 26.1, 18.5, 18.4, −4.4,

−4.7, −5.1, −5.2 ppm; IR (film) $\tilde{\nu}$ = 2953, 2928, 2856, 1726, 1452, 1315, 1276, 1257, 1175, 1107, 1095, 1070, 1027, 999, 890, 834, 813, 778, 710, 685 cm^{-1}; HRMS (ESI): *m/z*: calcd. for $C_{41}H_{62}O_9Si_2Na$ [M+Na]$^+$: 777.38246; found: 777.38217.

Compound 25. $NaHCO_3$ (120 mg, 1.43 mmol) and DMP (202 mg, 0.477 mmol) were added at ambient

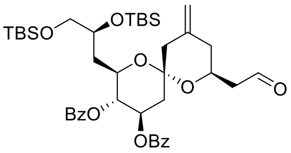

temperature to a solution of alcohol **S10** (180 mg, 0.238 mmol) in CH_2Cl_2 (3 mL). The mixture was stirred for 4 h before it was diluted with *tert*-butyl methyl ether (10 mL) and sat. aq. $NaHCO_3$ (5 mL). The biphasic mixture was stirred until two clear layers had formed. The aqueous layer was extracted with *tert*-butyl methyl ether (2 × 10 mL), and the combined extracts were washed with sat. aq. $NaHCO_3$ (2 × 10 mL) and brine (10 mL), dried over $MgSO_4$, filtered, and concentrated. Purification of the residue by flash chromatography on silica (*n*-pentane/*tert*-butyl methyl ether, 90:10 to 80:20) afforded the title compound as a colorless viscous syrup (163 mg, 90%). $[\alpha]_D^{20}$ = +14.3° (c = 1.04 in $CHCl_3$); ^{1}H NMR (400 MHz, CD_2Cl_2): δ = 9.90 (dd, *J* = 2.3, 1.5 Hz, 1H), 7.98 – 7.94 (m, 2H), 7.91 – 7.86 (m, 2H), 7.55 – 7.46 (m, 2H), 7.37 (m, 4H), 5.52 (ddd, *J* = 11.5, 9.6, 5.3 Hz, 1H), 5.21 (t, *J* = 9.7 Hz, 1H), 4.89 (q, *J* = 1.9 Hz, 1H), 4.83 (q, *J* = 1.9 Hz, 1H), 4.20 (dddd, *J* = 11.6, 7.4, 4.8, 2.6 Hz, 1H), 4.09 (td, *J* = 10.3, 1.9 Hz, 1H), 3.94 (dtt, *J* = 8.7, 5.9, 2.8 Hz, 1H), 3.66 (dd, *J* = 10.6, 3.4 Hz, 1H), 3.58 (dd, *J* = 10.6, 6.3 Hz, 1H), 2.76 (ddd, *J* = 16.8, 7.6, 2.4 Hz, 1H), 2.63 (ddd, *J* = 16.9, 4.8, 1.5 Hz, 1H), 2.49 (dd, *J* = 12.7, 5.3 Hz, 1H), 2.42 – 2.24 (m, 3H), 2.15 – 2.05 (m, 1H), 1.92 (ddd, *J* = 14.2, 8.8, 1.9 Hz, 1H), 1.83 (dd, *J* = 12.8, 11.5 Hz, 1H), 1.65 (ddd, *J* = 14.3, 10.5, 2.2 Hz, 1H), 0.84 (s, 18H), 0.02 (s, 3H), 0.01 (s, 3H), 0.01 (s, 3H), 0.00 ppm (s, 3H); ^{13}C NMR (101 MHz, CD_2Cl_2): δ = 200.3, 166.2, 166.0, 140.0, 133.5, 133.4, 130.3, 130.1, 130.0, 129.8, 128.7, 111.8, 98.8, 73.9, 71.1, 71.1, 67.9, 67.5, 66.7, 49.6, 43.5, 40.6, 39.3, 36.6, 26.2, 26.1, 26.1, 18.6, 18.5, −4.3, −4.7, −5.1, −5.2 ppm; IR (film) $\tilde{\nu}$ = 2953, 2928, 2856, 1725, 1471, 1452, 1315, 1276, 1256, 1175, 1096, 1069, 1027, 998, 894, 834, 813, 777, 710, 686 cm^{-1}; HRMS (ESI): *m/z*: calcd. for $C_{41}H_{60}O_9Si_2Na$ [M+Na]$^+$: 775.36681; found: 775.36628.

Compounds 28 and 28a. An oven-dried jacketed Schlenk flask was charged with (−)-$(Ipc)_2BH$ (powder,

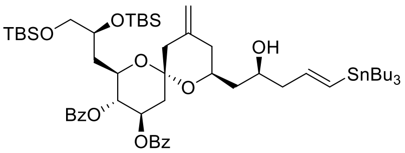

15.7 mg, 0.055 mmol). The solid was suspended in Et_2O (50 µL) and the mixture cooled to −40 °C with a cryostat. 1,2-Propadienyltributyltin (**26**) (≥90% pure, 27 µL, 0.080 mmol) was added dropwise via syringe. The walls of the reaction vessel were rinsed with Et_2O (70 µL) after complete addition of the stannane. The resulting suspension was stirred at −40 °C for ca. 30 min, then warmed to −20 °C over the course of 1 h and stirred at this temperature until a clear yellow solution had formed (2 h). The solution was cooled to −78 °C, before a solution of aldehyde **25** (20 mg, 0.027 mmol) in Et_2O (100 µL) was added dropwise. The mixture was stirred at −78 °C for 24 h and then quenched by dropwise addition of MeOH (0.2 mL) at −78 °C. The mixture was diluted with *tert*-butyl

methyl ether (0.5 mL) and warmed to 0 °C, before sodium perborate tetrahydrate (40 mg, ca. 10 equiv.) and H_2O (0.5 mL) were added. The biphasic mixture was warmed to ambient temperature and vigorously stirred overnight. The phases were separated and the aqueous layer was extracted with pentane (2 × 10 mL). The combined extracts were dried over $MgSO_4$, filtered, and concentrated. Purification of the residue by flash chromatography on silica (0.5% Et_3N in *n*-pentane/*tert*-butyl methyl ether, 100:0 to 99:1, then 75:25) afforded compound **28** as a yellow viscous oil (20.5 mg, 71%). $[\alpha]_D^{20}$ = +19.7° (c = 1.54 in CH_2Cl_2); ^1H NMR (400 MHz, CD_2Cl_2): δ = 7.97 – 7.93 (m, 2H), 7.91 – 7.87 (m, 2H), 7.54 – 7.47 (m, 2H), 7.41 – 7.33 (m, 4H), 6.19 – 5.95 (m, 2H), 5.59 (ddd, *J* = 11.5, 9.6, 5.2 Hz, 1H), 5.20 (t, *J* = 9.7 Hz, 1H), 4.84 (q, *J* = 1.9 Hz, 1H), 4.78 (q, *J* = 1.9 Hz, 1H), 4.12 – 4.05 (m, 1H), 4.01 (td, *J* = 10.2, 1.8 Hz, 1H), 3.98 – 3.90 (m, 2H), 3.65 – 3.55 (m, 2H), 2.49 (dd, *J* = 12.7, 5.3 Hz, 1H), 2.46 – 2.22 (m, 5H), 2.14 (t, *J* = 12.5 Hz, 1H), 1.92 (ddd, *J* = 14.3, 9.0, 1.7 Hz, 1H), 1.84 (dd, *J* = 12.7, 11.5 Hz, 1H), 1.79 – 1.58 (m, 3H), 1.58 – 1.47 (m, 6H), 1.38 – 1.22 (m, 6H), 0.95 – 0.86 (m, 15H), 0.84 (s, 9H), 0.83 (s, 9H), 0.02 (s, 3H), 0.02 (s, 3H), 0.01 (s, 3H), −0.02 ppm (s, 3H); ^{13}C NMR (101 MHz, CD_2Cl_2): δ = 166.2, 166.0, 145.5, 141.0, 133.5, 133.4, 132.7, 130.4, 130.1, 130.1, 129.9, 128.7, 128.7, 111.1, 98.6, 74.0, 71.2, 71.0, 69.3, 67.8, 67.5, 67.2, 47.5, 43.8, 42.6, 40.9, 39.7, 36.8, 34.5, 29.6, 27.7, 26.2, 26.2, 22.8, 18.7, 18.5, 14.2, 13.9, 9.8, −4.4, −4.6, −5.1 ppm; ^{119}Sn NMR (149 MHz, CD_2Cl_2) δ = −51.3 ppm; IR (film) $\tilde{\nu}$ = 2954, 2926, 2854, 1728, 1315, 1275, 1257, 1175, 1105, 1094, 1069, 1027, 992, 892, 834, 811, 777, 709, 686, 666 cm^{-1}; HRMS (ESI): *m/z*: calcd. for $C_{56}H_{92}O_9Si_2SnNa$ $[M+Na]^+$: 1107.51940; found: 1107.51932.

***Note**: 1,2-Propadienyltributyltin has a tendency to slowly isomerize to 1-propinyltributyltin unless stored under argon atmosphere at −20 °C. Contamination with 1-propinyltributyltin disrupts this reaction, leading to incomplete hydroboration and competitive reduction of the aldehyde to the corresponding alcohol.*

Note: *When compound **28** was chromatographed on silica (*n*-pentane/tert-butyl methyl ether, 100:0 to 90:10) without added NEt_3 in the eluent, proto-destannylated compound 28a was obtained in ≈40% yield.* $[\alpha]_D^{20}$ = +21.5 (c = 1.1 in CH_2Cl_2); ^1H NMR (400 MHz, $CDCl_3$): δ = 7.98 – 7.93 (m, 2H), 7.92 – 7.88 (m, 2H), 7.53 – 7.44 (m, 2H), 7.39 – 7.31 (m, 4H), 5.99 – 5.84 (m, 1H), 5.64 (ddd, *J* = 11.4, 9.6, 5.3 Hz, 1H), 5.26 – 5.13 (m, 3H), 4.82 (q, *J* = 1.9 Hz, 1H), 4.77 (q, *J* = 1.9 Hz, 1H), 4.07 (d, *J* = 12.6 Hz, 1H), 4.02 – 3.86 (m, 3H), 3.64 – 3.54 (m, 2H), 2.59 (s, 1H), 2.51 (dd, *J* = 12.7, 5.3 Hz, 1H), 2.41 – 2.19 (m, 5H), 2.11 (t, *J* = 12.4 Hz, 1H), 1.90 (ddd, *J* = 14.5, 9.0, 1.6 Hz, 1H), 1.83 (dd, *J* = 12.7, 11.5 Hz, 1H), 1.77 – 1.60 (m, 3H), 0.82 (s, 9H), 0.82 (s, 9H), 0.01 (s, 3H), 0.00 (s, 3H), −0.01 (s, 3H), −0.05 ppm (s, 3H); ^{13}C NMR (101 MHz, $CDCl_3$): δ = 166.1, 165.9, 140.4, 135.0, 133.3, 133.1, 130.0, 129.9, 129.8, 129.6, 128.5, 128.4, 118.1, 111.2, 98.3, 73.7, 70.9, 70.8, 68.7, 67.7, 67.4, 67.0, 43.6, 42.9, 42.4, 40.8, 39.6, 36.6, 26.2, 26.1, 18.5, 18.4, −4.4, −4.7, −5.1, −5.2. ppm; IR (film) $\tilde{\nu}$ = 2953, 2927, 2855, 1729,

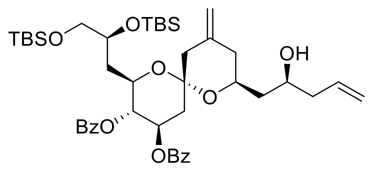

1277, 1260, 1107, 1070, 1028, 991, 836, 778, 710 cm^{-1}; HRMS (ESI): *m/z*: calcd. for $C_{44}H_{66}O_9Si_2Na$ $[M+Na]^+$: 817.41376; found: 817.41443.

Preparation of the (*R*)- and (*S*)-MTPA Ester of the Homoallylic Alcohol 28. A screw-cap vial was charged with a solution of homoallylic alcohol **28** (2 mg, 2 µmol) in CH_2Cl_2 (0.1 mL). Pyridine (0.1 mL) was added, followed by (*S*)-(+)-MTPA-Cl (1 drop). The vial was sealed with a screw-cap and parafilm. The mixture was warmed to ambient temperature and stirred overnight, then diluted with *n*-pentane (0.5 mL) and Et_3N (1 drop). The resulting suspension was directly submitted to flash chromatography on silica (2% Et_3N in *n*-pentane), affording the (*R*)-MTPA ester as a colorless film (1 mg, 40%). ^{1}H NMR (400 MHz, CD_2Cl_2): δ = 7.97 – 7.93 (m, 2H), 7.90 – 7.87 (m, 2H), 7.59 – 7.54 (m, 2H), 7.53 – 7.47 (m, 2H), 7.44 – 7.39 (m, 3H), 7.39 – 7.34 (m, 4H), 6.18 (d, *J* = 18.9 Hz, 1H), 6.05 – 5.94 (m, 1H), 5.61 – 5.53 (m, 1H), 5.33 (m, 1H, *superimposed with* CD_2Cl_2), 5.23 (t, *J* = 9.7 Hz, 1H), 4.76 (s, 2H), 3.97 – 3.87 (m, 1H), 3.75 – 3.67 (m, 1H), 3.62 (dd, *J* = 10.7, 3.3 Hz, 1H), 3.56 (s, 3H), 3.55 – 3.52 (m, 1H), 2.79 – 2.70 (m, 1H), 2.62 (dt, *J* = 14.7, 7.0 Hz, 1H), 2.41 (dd, *J* = 12.8, 5.3 Hz, 1H), 2.33 (d, *J* = 13.4 Hz, 1H), 2.23 (br s, 1H), 2.21 (br s, 1H), 2.04 – 1.86 (m, 4H), 1.83 – 1.74 (m, 1H), 1.64 (ddd, *J* = 14.1, 9.2, 2.5 Hz, 1H), 1.51 (m, 6H), 1.51 – 1.39 (m, 6H), 1.34 – 1.23 (m, 6H), 0.91 – 0.85 (m, 15H), 0.84 (s, 9H), 0.82 (s, 9H), 0.02 (s, 3H), 0.01 (s, 3H), 0.00 (s, 3H), −0.01 (s, 3H).

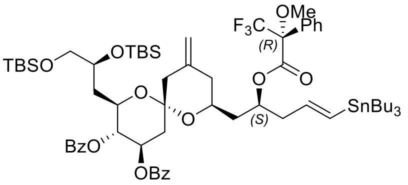

Analogous reaction of **28** (2 mg, 2 µmol) with (*R*)-(−)-MTPA-Cl (1 drop) gave the (*S*)-MTPA ester as a colorless film (1 mg, 40%). ^{1}H NMR (400 MHz, CD_2Cl_2): δ = 7.99 – 7.94 (m, 2H), 7.92 – 7.87 (m, 2H), 7.59 – 7.55 (m, 2H), 7.54 – 7.47 (m, 2H), 7.46 – 7.40 (m, 3H), 7.39 – 7.33 (m, 4H), 6.08 (d, *J* = 19.0 Hz, 1H), 5.86 (dt, *J* = 19.0, 6.4 Hz, 1H), 5.59 (ddd, *J* = 11.5, 9.5, 5.3 Hz, 1H), 5.38 – 5.34 (m, 1H, *partially superimposed with* CD_2Cl_2), 5.26 (t, *J* = 9.7 Hz, 1H), 4.83 (s, 1H), 4.79 (s, 1H), 3.99 – 3.87 (m, 2H), 3.75 (dd, *J* = 7.7, 4.4 Hz, 1H), 3.63 (dd, *J* = 10.7, 3.2 Hz, 1H), 3.55 (s, 3H), 3.53 – 3.49 (m, 1H), 2.72 – 2.63 (m, 1H), 2.56 (dt, *J* = 14.0, 6.4 Hz, 1H), 2.48 (dd, *J* = 12.8, 5.3 Hz, 1H), 2.38 – 2.24 (m, 3H), 2.09 – 1.94 (m, 3H), 1.89 (ddd, *J* = 14.5, 8.7, 2.2 Hz, 1H), 1.82 (dd, *J* = 12.8, 11.5 Hz, 1H), 1.65 (ddd, *J* = 14.4, 8.8, 2.6 Hz, 1H), 1.51 – 1.42 (m, 6H), 1.34 – 1.20 (m, 6H), 0.90 – 0.85 (m, 15H), 0.84 (s, 9H), 0.82 (s, 9H), 0.02 (s, 3H), 0.01 (s, 3H), 0.00 (s, 3H), −0.02 ppm (s, 3H).

Table S6. Determination of absolute configuration of the newly set stereocenter in **28** by Mosher ester analysis;[4] the recorded NMR data (CD_2Cl_2) suggest that it is (*S*)-configured (benthol A numbering scheme).

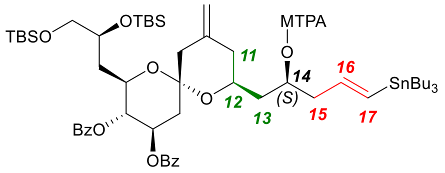

##	$\delta_{H\ NMR}$ (*S*-ester) (ppm)	$\delta_{H\ NMR}$ (*R*-ester) (ppm)	$\Delta\delta$ (δ_S -δ_R, ppm)
11a	2.05	1.98	0.07
11b	2.33	2.21	0.12
12	3.75	3.71	0.04
13a	1.99	1.91	0.08
13b	2.05	1.99	0.06
14	5.35	5.33	0.02
15a	2.56	2.62	−0.06
15b	2.67	2.75	−0.08
16	5.86	6.00	−0.14
17	6.08	6.18	−0.1

Sub-Structure Verification: Fragment A

Compounds 29 and 29a. K_2CO_3 (3 mg, 21 µmol) was added to a solution of alkenylstannane **28** (13.7 mg, 12.6 µmol) in MeOH (0.5 mL) and the resulting suspension was stirred at ambient temperature for 2 h before it was carefully concentrated by sparging with Ar. The residue was dissolved in THF (0.5 mL) and TBAF (1 M in THF, 0.05 mL, 50 µmol) was added drop-wise at 0°C (ice/water bath). Stirring was continued at room temperature for 2 h, at which point $CaCO_3$ (30 mg, 0.30 mmol), DOWEX 50WX8-400 (90 mg), and MeOH (1.0 mL) were added. The suspension was stirred for another 1 h, all insoluble materials were filtered off through a pad of Celite, and the filter cake was thoroughly washed with MeOH. The combined filtrates were concentrated under reduced pressure and the residue purified by flash chromatography (silica; CH_2Cl_2/MeOH, 95:5 to 90:10) to give compound **29a** as a

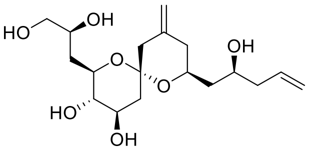

colorless syrup (3.1 mg, 68%), which analyzed as follows: $[\alpha]_D^{20}$ = +96.1 (c = 0.30 in $CHCl_3$); ^{1}H NMR (600 MHz, $CDCl_3$): δ = 5.87 – 5.78 (m, 1H), 5.15 – 5.07 (m, 2H), 4.85 (s, 1H), 4.77 (s, 1H), 4.01 – 3.94 (m, 1H), 3.94 – 3.89 (m, 2H), 3.86 (t, *J* = 10.9 Hz, 1H), 3.75 (t, *J* = 9.8 Hz, 1H), 3.57 (dd, *J* = 11.0, 5.2 Hz, 1H), 3.50 (dd, *J* = 11.0, 5.1 Hz, 1H), 3.11 (t, *J* = 9.2 Hz, 1H), 2.30 – 2.09 (m, 7H), 1.98 (t, *J* = 12.4 Hz, 1H), 1.67 – 1.59 (m, 1H),

1.59 – 1.48 ppm (m, 3H); ^{13}C NMR (151 MHz, CD_2Cl_2): δ = 141.2, 134.7, 118.2, 111.4, 98.7, 76.5, 72.3, 71.9, 69.4, 67.3, 66.5, 66.2, 43.9, 43.2, 42.4, 42.2, 40.0, 35.0 ppm; IR (film) $\tilde{\nu}$ = 3352, 2926, 1421, 1384, 1260, 1174, 1159, 1062, 1021, 983, 915, 893, 816, 755, 665, 608 cm^{-1}; HRMS (ESI): *m/z*: calcd. for $C_{18}H_{30}O_7Na$ [M+Na]$^+$: 381.18837; found: 381.18809.

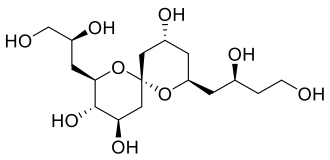

A flame-dried Schlenk flask was charged with compound **29a** (3 mg, 8.4 µmol) and CH_2Cl_2 and methanol (1:1, 4 mL). The solution was cooled to −78 °C before ozone was bubbled through for 5 min. Excess ozone was removed by purging with Ar, before $NaBH_4$ (excess, ca. 5 mg) was added. The mixture was stirred for 3 h at −78 °C, then warmed to room temperature and concentrated. The residue was dissolved in H_2O and passed through a C18-cartridge (elution with H_2O, then methanol). The product-containing fractions were concentrated and the resulting colorless film purified by HPLC (YMC Triart C18, MeOH/H_2O = 10:90), affording polyol **29** as a colorless film (1.6 mg, 52%). $[\alpha]_D^{20}$ = +52.8 (c = 0.06 in MeOH); ^{1}H NMR (600 MHz, [D$_4$]methanol): δ = 4.34 – 4.27 (m, 1H), 4.07 – 4.00 (m, 2H), 3.95 – 3.89 (m, 1H), 3.82 (ddd, *J* = 11.5, 8.9, 5.2 Hz, 1H), 3.74 (td, *J* = 10.1, 2.1 Hz, 1H), 3.70 (ddd, *J* = 6.9, 6.0, 3.3 Hz, 2H), 3.47 (dd, *J* = 11.0, 6.0 Hz, 1H), 3.42 (dd, *J* = 11.0, 5.5 Hz, 1H), 2.97 (dd, *J* = 9.6, 8.9 Hz, 1H), 2.17 (dt, *J* = 14.4, 2.1 Hz, 1H), 1.97 (dd, *J* = 12.8, 5.2 Hz, 1H), 1.81 (dt, *J* = 14.3, 2.2 Hz, 1H), 1.74 – 1.55 (m, 5H), 1.53 – 1.45 (m, 3H), 1.42 ppm (ddd, *J* = 14.5, 10.4, 9.2 Hz, 1H); ^{13}C NMR (151 MHz, [D$_4$]methanol): δ = 99.1, 77.3, 74.3, 73.6, 69.9, 67.5, 65.9, 65.3, 61.9, 60.1, 44.7, 44.5, 42.1, 40.6, 39.1, 36.3 ppm; IR (film) $\tilde{\nu}$ = 3355, 2924, 2884, 1598, 1399, 1124, 1074, 1055, 1018, 958, 672, 666, 582.92 cm^{-1}; HRMS (ESI): *m/z*: calcd. for $C_{16}H_{30}O_9Na$ [M+Na]$^+$: 389.17820; found: 389.17842.

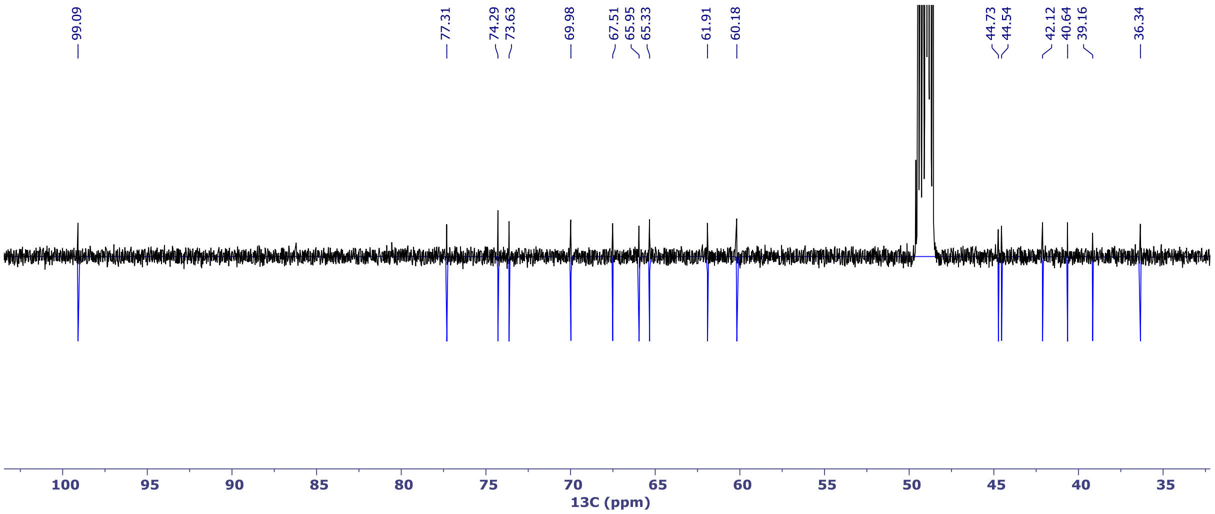

Figure S5. Visual comparison of the ^{13}C NMR spectrum of synthetic polyol **29** (top, black) with a simulated spectrum generated from the tabulated shift data reported for one of the degradation compounds derived from natural benthol A (bottom, blue; the deliberately uniform signal intensity is intended to show the simulated character).[5]

The Central Fragment B

Sub-Fragment B1

Compound 32. An oven-dried flask was charged with copper(I) iodide (1.70 g, 8.93 mmol) and THF (20 mL). The suspension was stirred at –78 °C (bath temperature) while vinylmagnesium bromide (1 M in THF, 67.0 mL, 67.00 mmol) was added dropwise. The resulting yellow solution was stirred at –78 °C for 10 min before a solution of (*S*)-(+)-benzyl-glycidylether (**31**) (7.33 g, 44.64 mmol) in THF (20 mL) was added dropwise. The mixture was then stirred at 0 °C (bath temperature) for 2 h. The reaction was quenched with sat. aq. NH_4Cl solution (30 mL) and the organic layer was separated. The aqueous phase was extracted with *tert*-butyl methyl ether (3 x 30 mL), the combined organic layers were washed with brine (50 mL), dried over Na_2SO_4 and concentrated under reduced pressure to give a pale-yellow oil that was used in the next step without further purification.

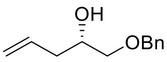

An aliquot was subjected to flash chromatography (silica; hexanes/EtOAc, 20:1 to 10:1) to give an analytically pure sample. $[\alpha]_D^{20}$ = + 3.0° (*c* = 1.44, $CHCl_3$). ^1H NMR (400 MHz, $CDCl_3$) δ 7.45 – 7.26 (m, 5H), 5.83 (ddt, *J* = 17.2, 10.3, 7.1 Hz, 1H), 5.17 – 5.05 (m, 2H), 4.56 (s, 2H), 3.95 – 3.81 (m, 1H), 3.52 (dd, *J* = 9.5, 3.4 Hz, 1H), 3.39 (dd, *J* = 9.5, 7.4 Hz, 1H), 2.39 (br, 1H), 2.31 – 2.20 (m, 2H). ^{13}C NMR (101 MHz, $CDCl_3$) δ 138.1, 134.4, 128.6, 127.9, 127.9, 117.8, 74.0, 73.5, 69.8, 38.0. IR (film) $\tilde{\nu}$ 3444, 3072, 3031, 2978, 2901, 2861, 1642, 1454, 1100, 997, 915, 738, 698 cm^{-1}. HRMS (EI): *m/z:* calcd. for $C_{12}H_{16}O_2$ $[M+H]^+$: 192.11448, found: 192.11442.

Compound S11. An oven-dried Schlenk tube was charged with the crude allylic alcohol **32** (44.64 mmol). The flask was evacuated and back-filled with Argon. Methyl acrylate (12.2 mL, 134.00 mmol) and degassed CH_2Cl_2 (90 mL) were added, followed by Grubbs II catalyst (379.0 mg, 0.45 mmol). The resulting mixture was stirred at 40 °C (bath temperature) under a gentle stream of Ar for 2 h before a second batch of Grubbs II catalyst (379.0 mg, 0.45 mmol) in degassed CH_2Cl_2 (5 mL) was added. Stirring was continued at 40 °C for another 1 h. Celite was added and the suspension stirred at room temperature for 10 min. The mixture was filtered through a plug of silica, rinsing with *tert*-butyl methyl ether. The combined filtrates were concentrated under reduced pressure and the residue was purified by flash chromatography (silica; hexanes/EtOAc, 5:1 to 3:1) to give the title compound as a dark-yellow oil (10.90 g, 98% over two steps, *E/Z* = 28:1). $[\alpha]_D^{20}$ = +2.3° (*c* = 1.36, $CHCl_3$). ^1H NMR (400 MHz, $CDCl_3$) δ 7.38 – 7.26 (m, 5H), 6.97 (dt, *J* = 15.7, 7.3 Hz, 1H), 5.89 (dt, *J* = 15.7, 1.5 Hz, 1H), 4.53 (s, 2H), 3.98 – 3.88 (m, 1H), 3.71 (s, 3H), 3.48 (dd, *J* = 9.5, 3.5 Hz, 1H), 3.37 (dd, *J* = 9.5, 7.1 Hz, 1H), 2.73 (br, 1H), 2.44 – 2.29 (m, 2H). ^{13}C NMR (101 MHz, $CDCl_3$) δ 166.8, 145.0, 137.8, 128.5, 127.9, 127.8, 123.4, 73.8, 73.5, 69.2, 51.5, 36.3. IR (film) $\tilde{\nu}$ 3474, 2950, 2903, 2861, 1719, 1657, 1436, 1324, 1273, 1209, 1167, 1094, 982, 739, 699 cm^{-1}. HRMS (ESI) *m/z* calcd. for $C_{14}H_{18}O_4Na$ $[M+Na]^+$: 273.10973, found: 273.10948.

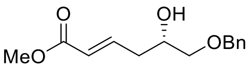

Compound 33. An oven-dried flask was charged with compound **S11** (10.90 g, 43.55 mmol) and CH_2Cl_2 (20 mL). The solution was stirred at 0 °C (bath temperature) before triethylamine (12.1 mL, 87.10 mmol) and methanesulfonyl chloride (5.1 mL, 65.32 mmol) were added. Stirring was continued at 0 °C for 30 min before the reaction was quenched with water (50 mL). The organic layer was separated and the aqueous phase extracted with CH_2Cl_2 (3 x 30 mL). The combined organic layers were dried over $MgSO_4$ and concentrated under reduced pressure, and the residue was purified by flash chromatography (silica; hexanes/EtOAc, 5:1 to 3:1) to give the title compound as a yellow oil (14.15 g, 99%). $[\alpha]_D^{20}$ = + 12.1° (*c* = 1.08, $CHCl_3$). ^{1}H NMR (400 MHz, $CDCl_3$) δ 7.40 – 7.27 (m, 5H), 6.88 (dt, *J* = 15.7, 7.5 Hz, 1H), 5.94 (dt, *J* = 15.7, 1.5 Hz, 1H), 4.96 – 4.83 (m, 1H), 4.57 (d, *J* = 11.8 Hz, 1H), 4.53 (d, *J* = 11.8 Hz, 1H), 3.74 (s, 3H), 3.67 – 3.57 (m, 2H), 3.01 (s, 3H), 2.72 – 2.58 (m, 2H). ^{13}C NMR (101 MHz, $CDCl_3$) δ 166.3, 141.7, 137.3, 128.7, 128.2, 127.9, 125.1, 79.5, 73.7, 70.8, 51.8, 38.7, 34.8. IR (film) $\tilde{\nu}$ 3030, 2951, 2870, 1719, 1661, 1437, 1348, 1275, 1171, 1105, 937, 916, 741, 700 cm^{-1}. HRMS (ESI) *m/z* calcd. for $C_{15}H_{20}O_6SNa$ [M+Na]$^+$: 351.08728, found: 351.08732.

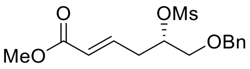

Compound S12. A Schlenk flask was charged with $K_3Fe(CN)_6$ (39.51 g, 120.00 mmol), K_2CO_3 (16.60 g, 120.00 mmol), $MeSO_2NH_2$ (7.61 g, 80.00 mmol), $(DHQD)_2PHAL$ (1.25 g, 1.60 mmol), $K_2OsO_2(OH)_4$ (295.0 mg, 0.80 mmol), *t*BuOH (100 mL) and H_2O (100 mL). The resulting mixture was vigorously stirred at room temperature for 15 min until a homogeneous solution had formed. This solution was cooled to 0 °C (bath temperature) before a solution of compound **33** (13.14 g, 40.00 mmol) in CH_2Cl_2 (20 mL) was added. Stirring was continued at 0 °C for 8 h and then for another 12 h at room temperature. The reaction was quenched with sat. aq. Na_2SO_3 and the mixture stirred for 1 h before the organic layer was separated and the aqueous phase was extracted with CH_2Cl_2 (3 x 50 mL). The combined organic layers were washed with HCl (2 M, 50 mL) and brine (100 mL), dried over Na_2SO_4 and concentrated under reduced pressure to give a pale-yellow oil that was used in the next step without further purification.

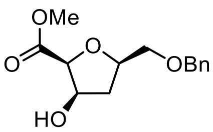

This crude material was dissolved in 2,6-lutidine (40 mL) and the resulting mixture stirred at 120 °C (bath temperature) for 6 h. The mixture was concentrated under reduced pressure and the residue purified by flash chromatography (silica; CH_2Cl_2/EtOAc, 10:1 to 1:1) to give the title compound as a yellow oil (8.48 g, 80% over two steps), along with fractions of three minor diastereomers (1.05 g, 10%). $[\alpha]_D^{20}$ = –69.1° (*c* = 1.57, $CHCl_3$). ^{1}H NMR (400 MHz, $CDCl_3$) δ 7.45 – 7.26 (m, 5H), 4.74 (d, *J* = 11.8 Hz, 1H), 4.58 (d, *J* = 11.9 Hz, 1H), 4.50 – 4.31 (m, 4H), 3.84 (dd, *J* = 10.6, 2.2 Hz, 1H), 3.80 (s, 3H), 3.49 (dd, *J* = 10.6, 2.1 Hz, 1H), 2.38 (ddd, *J* = 13.8, 10.1, 4.9 Hz, 1H), 2.03 (dd, *J* = 13.8, 2.5 Hz, 1H). ^{13}C NMR (101 MHz, $CDCl_3$) δ 169.5, 137.1, 128.7, 128.2, 128.1, 84.0, 78.3, 74.0, 73.0, 71.6, 52.1, 36.6. IR (film) $\tilde{\nu}$

3406, 2951, 2910, 2866, 1758, 1452, 1439, 1207, 1127, 1087, 1069, 1028, 739, 700 cm^{-1}. HRMS (ESI) m/z calcd. for $C_{14}H_{18}O_5Na$ $[M+Na]^+$: 289.10464, found: 289.10448.

Preparation of the (*S*)- and (*R*)-MTPA Esters of Alcohol S12. In an oven-dried HPLC vial equipped with a stirring bar, alcohol **S12** (10 mg, 37 µmol) was dissolved in CH_2Cl_2 (0.5 mL). Pyridine (9 µL, 113 µmol) was added, followed by (*S*)-(+)-MTPA-Cl (14 µL, 75 µmol). The vial was capped and stirred overnight at room temperature. The reaction was quenched with water (1 mL) and the aq. phase extracted with *tert*-butyl methyl ether (3 × 1 mL). The combined organic phases were washed with brine (3 mL) and dried over Na_2SO_4 before they were filtered and concentrated under reduced pressure. The residue was purified by flash chromatography on silica (hexanes/EtOAc, 9:1 to 7:3) to afford the (*R*)-MTPA ester as a colorless oil (17 mg, 94%). ^1H NMR (600 MHz, $CDCl_3$): δ = 7.49 – 7.44 (m, 2H), 7.43 – 7.36 (m, 3H), 7.35 – 7.24 (m, 5H), 5.72 (ddd, J = 6.4, 5.0, 2.6 Hz, 1H), 4.62 (d, J = 5.0 Hz, 1H), 4.50 (d, J = 12.0 Hz, 1H), 4.43 (d, J = 12.0 Hz, 1H), 4.30 (ddt, J = 8.2, 6.4, 5.6 Hz, 1H), 3.63 (s, 3H), 3.56 (dd, J = 9.9, 6.4 Hz, 1H), 3.46 (q, J = 1.1 Hz, 3H), 3.31 (dd, J = 10.0, 5.7 Hz, 1H), 2.54 (ddd, J = 14.5, 8.2, 6.3 Hz, 1H), 1.92 (ddd, J = 14.4, 5.6, 2.6 Hz, 1H) ppm.

The (*S*)-MTPA ester was prepared analogously from (*R*)-(+)-MTPA-Cl and alcohol **S12** (1.0 eq., 37 µmol, 10 mg) as a colorless oil (14.5 mg, 80%). ^1H NMR (600 MHz, $CDCl_3$): δ = 7.49 – 7.47 (m, 2H), 7.42 – 7.25 (m, 8H), 5.73 (ddd, J = 6.5, 5.1, 2.9 Hz, 1H), 4.58 (d, J = 5.1 Hz, 1H), 4.57 (d, J = 12.0 Hz, 1H), 4.53 (d, J = 11.9 Hz, 1H), 4.31 (dq, J = 8.0, 6.0 Hz, 1H), 3.68 (dd, J = 10.0, 6.0 Hz, 1H), 3.49 (dd, J = 10.1, 5.7 Hz, 1H), 3.47 (s, 3H), 3.44 (q, J = 1.2 Hz, 3H), 2.57 (ddd, J = 14.4, 8.0, 6.5 Hz, 1H), 2.03 (ddd, J = 14.4, 6.1, 3.0 Hz, 1H) ppm.

Table S7. Determination of absolute configuration of the newly set chiral center in **S12** via Mosher ester analysis.[4] The recorded NMR data ($CDCl_3$) suggest that the C3-center is (*R*)-configured.

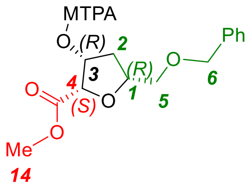

##	$\delta_{H\ NMR}$ (*S*-ester) (ppm)	$\delta_{H\ NMR}$ (*R*-ester) (ppm)	$\Delta\delta$ (δ_S -δ_R, ppm)
1	4.31	4.3	0.01
2a	2.57	2.54	0.03
2b	2.03	1.92	0.11
3	5.73	5.72	0.01
4	4.58	4.62	−0.04
5a	3.68	3.56	0.12
5b	3.49	3.31	0.18
6a	4.57	4.5	0.07
6b	4.53	4.43	0.1
14	3.47	3.63	−0.16

Compound 35. Imidazole (7.58 g, 111.46 mmol), DMAP (0.58 g, 4.78 mmol) and TBSCl (14.40 g, 95.53 mmol) were added to a solution of alcohol **S12** (8.48 g, 31.85 mmol) in DMF (30 mL) at 0 °C (bath temperature). The mixture was stirred at room temperature for 16 h before the reaction was quenched with water (300 mL). The mixture was extracted with EtOAc (3 x 100 mL), the combined organic layers were washed with brine (100 mL), dried over Na_2SO_4 and concentrated under reduced pressure. The residue was purified by flash chromatography (silica; hexanes/EtOAc, 10:1 to 1:1) to give the title compound as a pale-yellow oil (12.15 g, quant.). $[\alpha]_D^{20}$ = –7.0° (*c* = 2.14, $CHCl_3$). ^1H NMR (400 MHz, $CDCl_3$) δ 7.42 – 7.20 (m, 5H), 4.69 – 4.53 (m, 3H), 4.47 (d, *J* = 5.4 Hz, 1H), 4.35 – 4.24 (m, 1H), 3.82 (dd, *J* = 9.7, 6.7 Hz, 1H), 3.72 (s, 3H), 3.59 (dd, *J* = 9.6, 5.7 Hz, 1H), 2.21 (ddd, *J* = 13.4, 7.5, 5.9 Hz, 1H), 1.87 (ddd, *J* = 12.9, 5.9, 4.1 Hz, 1H), 0.84 (s, 9H), 0.05 (s, 3H), 0.04 (s, 3H). ^{13}C NMR (101 MHz, $CDCl_3$) δ 170.4, 138.4, 128.4, 128.0, 127.7, 82.5, 78.7, 73.7, 73.5, 73.1, 51.8, 37.6, 25.7, 17.9, –4.8, –5.2. IR (film) $\tilde{\nu}$ 2951, 2929, 2886, 2856, 1767, 1437, 1255, 1205, 1099, 1062, 937, 837, 777, 735, 698 cm^{-1}. HRMS (ESI) *m/z* calcd. for $C_{20}H_{32}O_5SiNa$ $[M+Na]^+$: 403.19112, found: 403.19140.

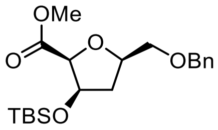

Compound S13. A mixture comprising Pd/C (10 wt %, 0.20 g) and benzyl ether (2.00 g, 5.26 mmol) in MeOH (20 mL) was stirred under an atmosphere of H_2 (balloon) for 12 h. The mixture was filtered through a short pad of Celite that was rinsed with EtOAc. The combined filtrates were concentrated under reduced pressure and the residue was purified by flash chromatography (silica; pentane/EtOAc, 3:1 to 1:1) to give the title compound (605.0 mg, 40%) and a second fraction consisting of the TBS-deprotected compound **S14** (583.0 mg, 60%).

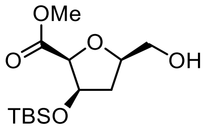

Analytical and spectral data of compound **S13**: White solid. $[\alpha]_D^{20}$ = + 13.8° (*c* = 1.03, $CHCl_3$). ^1H NMR (400 MHz, $CDCl_3$) δ 4.65 (q, *J* = 6.2 Hz, 1H), 4.45 (d, *J* = 6.3 Hz, 1H), 4.29 – 4.21 (m, 1H), 3.80 (dd, *J* = 12.0, 2.9 Hz, 1H), 3.72 (s, 3H), 3.65 (dd, *J* = 12.0, 4.5 Hz, 1H), 2.17 – 1.99 (m, 2H), 0.84 (s, 9H), 0.06 (s, 3H), 0.04 (s, 3H). ^{13}C NMR (101 MHz, $CDCl_3$) δ 172.2, 80.9, 80.2, 74.0, 63.9, 52.0, 34.9, 25.6, 18.0, –4.9, –5.2. IR (film) $\tilde{\nu}$ 3468, 2952, 2930, 2890, 2859, 1743, 1439, 1256, 1216, 1128, 1103, 1059, 837, 778 cm^{-1}. HRMS (ESI) *m/z* calcd. for $C_{13}H_{26}O_5SiNa$ $[M+Na]^+$: 313.14417, found: 313.14445.

Single crystals suitable for X-ray diffraction analysis were grown from pentane/CH_2Cl_2.

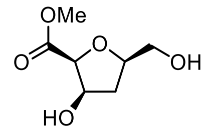

Analytical and spectral data of compound **S14**: Colorless oil. $[\alpha]_D^{20}$ = –26.3° (*c* = 1.40, $CHCl_3$). ^1H NMR (400 MHz, $CDCl_3$) δ 4.49 (ddd, *J* = 5.8, 4.4, 1.5 Hz, 1H), 4.46 (d, *J* = 4.3 Hz, 1H), 4.41 – 4.26 (m, 3H), 3.93 (dd, *J* = 12.2, 2.6 Hz, 1H), 3.75 (s, 3H), 3.48 (dd, *J* = 12.3, 1.9 Hz, 1H), 2.39 (ddd, *J* = 13.9, 9.5, 5.7 Hz, 1H), 1.99 (ddd, *J* = 14.3, 3.2, 1.5 Hz, 1H). ^{13}C NMR (101 MHz, $CDCl_3$) δ 171.2, 83.5, 79.8, 72.8, 64.0, 52.3, 36.9. IR (film) $\tilde{\nu}$ 3386, 2953, 1741, 1440, 1223,

1121, 1090, 1039, 865, 836, 730, 623 cm^{-1}. HRMS (ESI) *m/z* calcd. for $C_7H_{12}O_5Na$ $[M+Na]^+$: 199.05769, found: 199.05763.

Compound 37. DIBAL-H (1.0 M in hexane, 7.50 mL, 7.50 mmol) was slowly added to a solution of ester

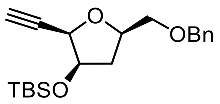

35 (1.90 g, 5.00 mmol) in CH_2Cl_2 (20 mL) at –78 °C (bath temperature). After stirring for 1 h at this temperature, the reaction was quenched with sat. aq. Rochelle salt solution (30 mL). The resulting mixture was vigorously stirred at room temperature for 1 h before the aqueous phase was extracted with CH_2Cl_2 (3 x 20 mL). The combined organic layers were dried over $MgSO_4$ and concentrated under reduced pressure to give a colorless oil that was used in the next step without further purification.

An oven-dried flask was charged with the crude aldehyde (5.00 mmol) and MeOH (20 mL). This mixture was stirred at 0 °C (bath temperature) before K_2CO_3 (2.07 g, 15.00 mmol) was added, followed by slow addition of Bestmann-Ohira reagent (1.44 mL, 6.00 mmol). Stirring was continued at room temperature for 3 h before the reaction was quenched with water (30 mL). The aqueous layer was extracted with *tert*-butyl methyl ether (3 x 30 mL). The combined organic layers were dried over Na_2SO_4 and concentrated under reduced pressure and the residue purified by flash chromatography (silica; hexane/EtOAc, 10:1) to give the title compound as a colorless oil (1.56 g, 90% over two steps). $[\alpha]_D^{20}$ = + 15.1° (*c* = 0.90, $CHCl_3$). ^{1}H NMR (400 MHz, $CDCl_3$) δ 7.43 – 7.19 (m, 5H), 4.62 (d, *J* = 12.0 Hz, 1H), 4.55 (d, *J* = 12.2 Hz, 1H), 4.46 (dd, *J* = 4.8, 2.2 Hz, 1H), 4.35 (dt, *J* = 6.1, 4.7 Hz, 1H), 4.18 (dtd, *J* = 7.9, 6.5, 5.4 Hz, 1H), 3.72 (dd, *J* = 9.8, 6.6 Hz, 1H), 3.55 (dd, *J* = 9.8, 5.6 Hz, 1H), 2.45 (d, *J* = 2.2 Hz, 1H), 2.24 (ddd, *J* = 12.9, 7.8, 6.0 Hz, 1H), 1.84 (ddd, *J* = 12.9, 6.5, 4.6 Hz, 1H), 0.91 (s, 9H), 0.11 (s, 3H), 0.09 (s, 3H). ^{13}C NMR (101 MHz, $CDCl_3$) δ 138.4, 128.4, 127.9, 127.7, 80.4, 77.4, 75.5, 73.5, 73.5, 73.4, 73.3, 37.8, 25.9, 18.3, –4.6, –4.8. IR (film) $\tilde{\nu}$ 3311, 3064, 3031, 2951, 2928, 2896, 2856, 1472, 1463, 1454, 1363, 1253, 1197, 1148, 1102, 1072, 930, 870, 837, 777, 737, 698 cm^{-1}. HRMS (ESI) *m/z* calcd. for $C_{20}H_{30}O_3SiNa$ $[M+Na]^+$: 369.18564, found: 369.18544.

Compound 38. DIBAL-H (1.0 M in hexane, 7.5 mL, 7.50 mmol) was slowly added to a solution of ester

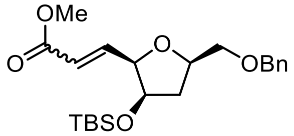

35 (1.90 g, 5.00 mmol) in CH_2Cl_2 (20 mL) at –78 °C. After stirring at this temperature for 1 h, the reaction was quenched with sat. aq. Rochelle salt solution (20 mL). The resulting mixture was vigorously stirred at room temperature for 1 h, the layers were separated, and the aqueous phase extracted with CH_2Cl_2 (3 x 20 mL). The combined organic layers were dried over $MgSO_4$ and concentrated under reduced pressure to give a colorless oil that was used in the next step without further purification.

An oven-dried flask was charged with $MeOOCCH=PPh_3$ (2.51 g, 7.50 mmol) and CH_2Cl_2 (20 mL). The resulting solution was stirred at 0 °C (bath temperature) while a solution of the crude aldehyde in CH_2Cl_2 (10 mL) was added dropwise. Once the addition was complete, stirring was continued at room

temperature for 3 h. The mixture was concentrated under reduced pressure and the residue purified by flash chromatography (silica; hexane/EtOAc, 5:1) to give the title compound as a colorless oil (1.92 g, 94% over two steps, *E*/*Z* = 2.7:1).

Spectral data of the E-isomer: ^{1}H NMR (400 MHz, $CDCl_3$) δ 7.37 – 7.26 (m, 5H), 6.95 (dd, *J* = 15.7, 5.2 Hz, 1H), 6.09 (dd, *J* = 15.8, 1.3 Hz, 1H), 4.64 – 4.53 (m, 3H), 4.42 – 4.39 (m, 1H), 4.30 – 4.18 (m, 1H), 3.73 (s, 3H), 3.66 (dd, *J* = 9.7, 6.8 Hz, 1H), 3.52 (dd, *J* = 9.7, 5.3 Hz, 1H), 2.24 (ddd, *J* = 13.7, 8.0, 5.7 Hz, 1H), 1.81 – 1.75 (m, 1H), 0.83 (s, 9H), 0.02 (s, 3H), 0.01 (s, 3H). ^{13}C NMR (101 MHz, $CDCl_3$) δ 166.8, 145.6, 138.4, 128.5, 127.9, 127.7, 122.0, 82.5, 77.6, 74.2, 73.5, 73.5, 51.6, 38.4, 25.7, 18.1, –4.7, –5.0.

Spectral data of the Z-isomer: ^{1}H NMR (400 MHz, $CDCl_3$) δ 7.37 – 7.26 (m, 5H), 6.35 (dd, *J* = 11.8, 7.1 Hz, 1H), 5.87 (dd, *J* = 11.8, 1.6 Hz, 1H), 5.20 (ddd, *J* = 7.0, 3.8, 1.6 Hz, 1H), 4.64 – 4.53 (m, 1H), 4.42 – 4.39 (m, 2H), 4.30 – 4.18 (m, 1H), 3.70 (s, 3H), 3.69 (dd, *J* = 9.8, 7.0 Hz, 1H), 3.53 (dd, *J* = 9.8, 5.3 Hz, 1H), 2.30 (ddd, *J* = 13.3, 8.6, 5.6 Hz, 1H), 1.75 – 1.70 (m, 1H), 0.82 (s, 9H), –0.02 (s, 3H), –0.07 (s, 3H).

^{13}C NMR (101 MHz, $CDCl_3$) δ 166.5, 149.0, 138.5, 128.5, 127.9, 127.7, 119.7, 81.3, 77.7, 74.5, 73.6, 73.4, 51.4, 39.2, 25.8, 18.1, –4.8, –5.1. IR (film) $\tilde{\nu}$ 2953, 2929, 2990, 2857, 1724, 1664, 1454, 1438, 1361, 1307, 1256, 1198, 1104, 1072, 937, 837, 777 cm^{-1}.

HRMS (ESI) *m/z* calcd. for $C_{22}H_{34}O_5SiNa$ $[M+Na]^+$: 429.20677, found: 429.20664.

Compound S15. A solution of compound **38** (1.92 g, 4.72 mmol) in EtOAc (47 mL) was added dropwise at 0 °C (bath temperature) to a suspension of $Pd(OH)_2/C$ (20 wt %, 332.0 mg) in EtOAc (110 mL). The mixture was stirred under an atmospheric pressure of dihydrogen (balloon) at room temperature for 6 h before it was filtered through a short pad of Celite, rinsing with EtOAc. The combined filtrates were concentrated under reduced pressure and the residue was purified by flash chromatography (silica; hexane/EtOAc, 2:1) to give the title compound as a colorless oil (1.41 g, 94%). $[\alpha]_D^{20}$ = –17.8° (*c* = 2.48, $CHCl_3$). ^{1}H NMR (400 MHz, $CDCl_3$) δ 4.22 (ddd, *J* = 5.7, 3.7, 2.2 Hz, 1H), 4.10 (dtd, *J* = 9.1, 4.7, 2.7 Hz, 1H), 3.75 – 3.67 (m, 2H), 3.66 (s, 3H), 3.53 (ddd, *J* = 11.5, 6.1, 4.5 Hz, 1H), 2.58 (t, *J* = 5.8 Hz, 1H), 2.45 (td, *J* = 7.1, 1.2 Hz, 2H), 2.21 (ddd, *J* = 13.4, 8.9, 5.5 Hz, 1H), 1.95 (ddt, *J* = 14.1, 8.9, 7.0 Hz, 1H), 1.88 – 1.78 (m, 2H), 0.89 (s, 9H), 0.08 (s, 3H), 0.07 (s, 3H). ^{13}C NMR (101 MHz, $CDCl_3$) δ 174.4, 82.4, 78.1, 73.2, 64.9, 51.7, 37.2, 31.1, 25.9, 25.2, 18.2, –4.6, –4.9. IR (film) $\tilde{\nu}$ 3453, 2953, 2931, 2887, 2857, 1739, 1472, 1463, 1438, 1362, 1256, 1195, 1172, 1130, 1108, 1059, 952, 938, 837, 776 cm^{-1}. HRMS (ESI) *m/z* calcd. for $C_{15}H_{30}O_5SiNa$ $[M+Na]^+$: 341.17547, found: 341.17585.

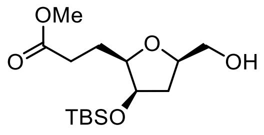

Compound 39. An oven-dried flask was charged with oxalyl chloride (1.62 mL, 18.56 mmol) and CH_2Cl_2 (50 mL). The solution was stirred at –78 °C (bath temperature) for 5 min before dry DMSO (1.32 mL, 18.56 mmol) was slowly added. Stirring was continued at –78 °C for 30 min before a solution of alcohol **S15** (1.97 g, 6.19 mmol) in CH_2Cl_2 (12 mL) was added dropwise. After stirring at –78 °C for 1 h, Et_3N (5.17 mL, 37.11 mmol) was slowly added. The mixture was stirred for 30 min at –78 °C and for another 30 min at 0 °C for before ice water (50 mL) was introduced. The aqueous layer was extracted with CH_2Cl_2 (3 x 30 mL), the combined organic phases were washed with brine (50 mL), dried over Na_2SO_4 and concentrated under reduced pressure to give a yellow oil that was used in the next step without further purification.

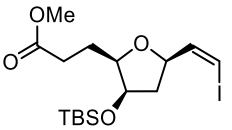

An oven-dried flask was charged with iodomethyl triphenylphosphonium iodide (4.92 g, 9.28 mmol) and THF (40 mL). This mixture was stirred at 0 °C while a solution of KHMDS (1.85 g, 9.28 mmol) in THF (10 mL) was added dropwise. The resulting mixture was stirred at –78 °C before HMPA (4.84 mL, 27.84 mmol) was added, followed by the slow addition of a solution of the crude aldehyde in THF (12 mL). After stirring at –78 °C for 30 min and for another 30 min at 0 °C, the reaction was quenched with sat. aq. NH_4Cl (30 mL) and the aqueous phase extracted with *tert*-butyl methyl ether (3 x 30 mL). The combined organic layers were washed with water (30 mL) and brine (30 mL), dried over Na_2SO_4, and concentrated under reduced pressure. The residue was purified by flash chromatography (silica; hexane/EtOAc, 10:1) to give the title compound as a yellow oil (2.32 g, 85% over two steps). $[\alpha]_D^{20}$ = +18.8° (*c* = 1.14, $CHCl_3$). ^1H NMR (400 MHz, $CDCl_3$) δ 6.45 (t, *J* = 7.4 Hz, 1H), 6.21 (dd, *J* = 7.6, 1.4 Hz, 1H), 4.64 – 4.53 (m, 1H), 4.26 (ddd, *J* = 5.3, 3.9, 2.5 Hz, 1H), 3.78 – 3.71 (m, 1H), 3.66 (s, 3H), 2.56 – 2.36 (m, 3H), 2.02 – 1.82 (m, 2H), 1.69 (ddd, *J* = 13.3, 4.9, 2.5 Hz, 1H), 0.88 (s, 9H), 0.07 (s, 3H), 0.05 (s, 3H). ^{13}C NMR (101 MHz, $CDCl_3$) δ 174.2, 144.1, 82.7, 80.6, 80.1, 73.1, 51.7, 41.7, 30.9, 25.9, 25.4, 18.2, –4.4, –4.9. IR (film) $\tilde{\nu}$ 2952, 2929, 2897, 2856, 1738, 1610, 1471, 1462, 1437, 1361, 1255, 1192, 1170, 1104, 1068, 1046, 1007, 948, 835, 775 cm^{-1}. HRMS (ESI) *m/z* calcd. for $C_{16}H_{29}IO_4SiNa$ $[M+Na]^+$: 463.07721, found: 463.07749.

Compound 40. An oven-dried Schlenk tube was charged with (*Z*)-alkenyl iodide **39** (0.88 g, 2.00 mmol), Et_3N (5.0 mL), $Pd(PPh_3)_2Cl_2$ (140.3 mg, 0.20 mmol) and CuI (76.2 mg, 0.40 mmol). The resulting suspension was stirred at room temperature for 30 min before a solution of alkyne **37** (1.04 g, 3.00 mmol) in Et_3N (5.0 mL) was added dropwise. Stirring was continued in the dark for 2 h before the mixture was filtered through a pad of Celite. The filtrate was concentrated under reduced pressure and the residue was purified by flash chromatography (silica; hexane/EtOAc, 10:1 to 5:1) to give the title compound as a yellow oil (1.23 g, 93%). $[\alpha]_D^{20}$ = + 29.0° (*c* = 0.91, $CHCl_3$). ^1H NMR (600 MHz, $CDCl_3$) δ 7.37 – 7.30 (m, 4H), 7.28 – 7.24 (m, 1H), 6.04 (dd, *J* = 10.9, 8.5 Hz, 1H), 5.49

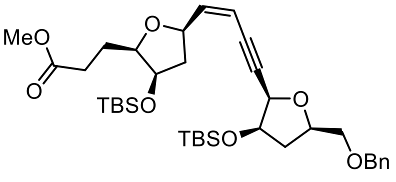

(dt, J = 10.9, 1.5 Hz, 1H), 4.88 (tdd, J = 8.4, 5.5, 1.2 Hz, 1H), 4.62 (d, J = 12.1 Hz, 1H), 4.59 (dd, J = 4.7, 1.9 Hz, 1H), 4.54 (d, J = 12.1 Hz, 1H), 4.34 (dt, J = 6.2, 4.7 Hz, 1H), 4.24 (ddd, J = 5.5, 4.1, 2.9 Hz, 1H), 4.18 (dtd, J = 7.9, 6.6, 5.2 Hz, 1H), 3.74 – 3.67 (m, 2H), 3.66 (s, 3H), 3.53 (dd, J = 9.8, 5.4 Hz, 1H), 2.52 – 2.39 (m, 2H), 2.37 (ddd, J = 13.4, 8.3, 5.5 Hz, 1H), 2.23 (ddd, J = 13.2, 7.8, 6.0 Hz, 1H), 1.97 – 1.84 (m, 2H), 1.80 (ddd, J = 13.0, 6.6, 4.7 Hz, 1H), 1.64 (ddd, J = 13.3, 5.6, 2.9 Hz, 1H), 0.89 (s, 9H), 0.88 (s, 9H), 0.07 (s, 3H), 0.06 (s, 3H), 0.06 (s, 3H), 0.05 (s, 3H). ^{13}C NMR (151 MHz, $CDCl_3$) δ 174.3, 145.4, 138.4, 128.5, 127.9, 127.7, 109.0, 91.3, 83.2, 82.3, 77.2, 75.4, 74.1, 73.6, 73.5, 73.5, 73.4, 51.6, 42.4, 37.8, 31.0, 25.9, 25.9, 25.5, 18.3, 18.2, –4.4, –4.6, –4.8, –4.9. IR (film) ṽ 2952, 2930, 2896, 2856, 1740, 1472, 1463, 1438, 1362, 1255, 1194, 1155, 1103, 1060, 1007, 937, 863, 836, 776, 736, 698 cm^{-1}. HRMS (ESI) *m/z* calcd. for $C_{36}H_{58}O_7Si_2Na$ $[M+Na]^+$: 681.36133, found: 681.36154.

Compound 42. DIBAL-H (1.0 M in hexane, 7.5 mL, 7.5 mmol) was slowly added to a solution of ester **35** (1.90 g, 5.0 mmol) in CH_2Cl_2 (20 mL) at –78 °C (bath temperature) and the resulting mixture was stirred at this temperature for 1 h. The reaction was quenched with sat. aq. Rochelle salt solution (20 mL). The resulting mixture was vigorously stirred at room temperature for 1 h before the aqueous phase was extracted with CH_2Cl_2 (3 x 20 mL). The combined organic layers were dried over $MgSO_4$ and concentrated under reduced pressure to give a colorless oil that was used in the next step without further purification.

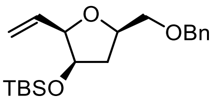

A solution of the crude aldehyde in THF (10 mL) was added dropwise at 0 °C (bath temperature) to a solution of $Ph_3P=CH_2$ (2.07 g, 7.5 mmol) in THF (20 mL). After stirring at this temperature for 1 h and for another 2 h at ambient temperature, the reaction was quenched with H_2O (10 mL). The organic layer was separated and the aqueous phase was extracted with *tert*-butyl methyl ether (3 x 20 mL). The combined organic layers were dried over $MgSO_4$ and concentrated under reduced pressure, and the residue was purified by flash chromatography (silica; hexane/EtOAc = 10:1) to give the title compound as a pale-yellow oil (1.58 g, 91% over two steps). $[\alpha]_D^{20}$ = –19.4° (c = 0.87, $CHCl_3$). ^{1}H NMR (400 MHz, $CDCl_3$) δ 7.40 – 7.23 (m, 5H), 5.95 (ddd, J = 17.5, 10.3, 7.3 Hz, 1H), 5.29 (ddd, J = 17.4, 1.9, 1.0 Hz, 1H), 5.21 (ddd, J = 10.4, 1.9, 0.9 Hz, 1H), 4.61 (d, J = 12.2 Hz, 1H), 4.56 (d, J = 12.2 Hz, 1H), 4.28 (ddd, J = 5.6, 4.1, 2.7 Hz, 1H), 4.24 – 4.13 (m, 2H), 3.68 (dd, J = 9.6, 6.6 Hz, 1H), 3.53 (dd, J = 9.6, 5.6 Hz, 1H), 2.24 (ddd, J = 13.7, 8.2, 5.6 Hz, 1H), 1.77 (ddd, J = 13.2, 5.3, 2.7 Hz, 1H), 0.87 (s, 9H), 0.03 (s, 3H), 0.03 (s, 3H). ^{13}C NMR (101 MHz, $CDCl_3$) δ 138.5, 135.6, 128.4, 127.9, 127.6, 117.8, 85.1, 77.1, 74.3, 73.7, 73.5, 38.7, 25.9, 18.2, –4.7, –4.8. IR (film) ṽ 2954, 2929, 2896, 2857, 1471, 1462, 1454, 1362, 1255, 1198, 1140, 1101, 1064, 992, 922, 872, 836, 776, 736, 698 cm^{-1}. HRMS (ESI) *m/z* calcd. for $C_{20}H_{32}O_3SiNa$ $[M+Na]^+$: 371.20129, found: 371.20129.

Compound 43. An oven-dried Schlenk tube was charged with 9-H-9-BBN dimer (0.61 g, 2.50 mmol), alkene **42** (1.74 g, 5.00 mmol) and THF (20 mL). The mixture was stirred at room temperature for 16 h

before aq. Cs_2CO_3 solution (3.0 M, 4.2 mL, 12.60 mmol) was introduced. After stirring for 20 min at

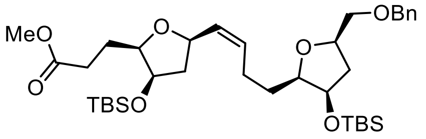

room temperature, alkenyl iodide **39** (1.84 g, 4.17 mmol), DMF (20 mL), $AsPh_3$ (0.51 g, 1.67 mmol) and Pd(dppf)Cl_2 (305.0 mg, 0.417 mmol) were added and stirring was continued for 10 h. The mixture was diluted with brine (100 mL) and the aqueous phase extracted with EtOAc (3 x 50 mL). The combined organic layers were washed with brine (100 mL), dried over $MgSO_4$ and concentrated under reduced pressure. The residue was purified by flash chromatography (silica; hexane/EtOAc, 10:1 to 3:1) to give the title compound as an orange oil (2.66 g, 96%). $[\alpha]_D^{20}$ = –3.0° (*c* = 1.35, $CHCl_3$). ^1H NMR (400 MHz, $CDCl_3$) δ 7.34 – 7.25 (m, 5H), 5.54 (ddt, *J* = 11.0, 8.2, 1.3 Hz, 1H), 5.49 – 5.40 (m, 1H), 4.67 – 4.57 (m, 2H), 4.54 (d, *J* = 12.2 Hz, 1H), 4.25 (ddd, *J* = 6.1, 4.6, 3.5 Hz, 1H), 4.18 (ddd, *J* = 6.0, 3.9, 2.3 Hz, 1H), 4.08 (ddt, *J* = 8.4, 6.7, 5.3 Hz, 1H), 3.67 – 3.57 (m, 6H), 3.46 (dd, *J* = 9.6, 5.3 Hz, 1H), 2.55 – 2.37 (m, 2H), 2.30 (ddd, *J* = 12.9, 7.7, 6.0 Hz, 1H), 2.26 – 2.07 (m, 3H), 1.99 – 1.83 (m, 2H), 1.78 – 1.70 (m, 1H), 1.67 (ddd, *J* = 13.4, 5.4, 2.4 Hz, 1H), 1.61 – 1.53 (m, 2H), 0.89 (s, 9H), 0.86 (s, 9H), 0.05 (s, 3H), 0.05 (s, 3H), 0.03 (s, 6H). ^{13}C NMR (101 MHz, $CDCl_3$) δ 174.4, 138.6, 132.2, 131.3, 128.5, 127.9, 127.7, 82.8, 81.8, 76.6, 73.8, 73.6, 73.4, 73.0, 72.8, 51.6, 42.9, 38.9, 31.0, 29.6, 25.9 (two peaks), 25.4, 24.5, 18.2, 18.2, –4.3, –4.4, –4.9, –5.0. IR (film) $\tilde{\nu}$ 2953, 2930, 2897, 2856, 1740, 1471, 1462, 1439, 1361, 1255, 1194, 1171, 1076, 1007, 939, 870, 836, 775 cm^{-1}. HRMS (ESI) *m/z* calcd. for $C_{36}H_{62}O_7Si_2Na$ $[M+Na]^+$: 685.39263, found: 685.39295.

Compound 44. An oven-dried flask was charged at 0 °C (bath temperature) with the major by-product

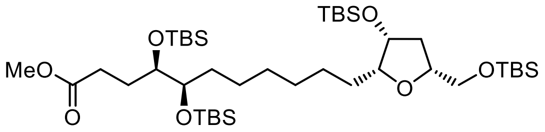

(≈ 0.70 mmol) [formed upon hydrogenation of compound **43** over $Pd(OH)_2$/C], DMF (10 mL), imidazole (0.38 g, 5.60 mmol), DMAP (26.0 mg, 0.21 mmol), and TBSCl (0.85 g, 5.60 mmol). The resulting mixture was stirred at room temperature for 24 h before the reaction was quenched with H_2O (80 mL). The aqueous phase was extracted with EtOAc (3 x 20 mL), and the combined organic layers were washed with brine (80 mL), dried over Na_2SO_4, and concentrated under reduced pressure. The residue was purified by flash chromatography (silica; hexane/EtOAc = 10:1) to give the title compound as a colorless oil (271.0 mg). $[\alpha]_D^{20}$ = + 14.5° (*c* = 1.83, $CHCl_3$). ^1H NMR (400 MHz, $CDCl_3$) δ 4.16 (ddd, *J* = 5.6, 3.7, 2.1 Hz, 1H), 4.00 – 3.89 (m, 1H), 3.75 (dd, *J* = 9.9, 6.0 Hz, 1H), 3.68 – 3.61 (m, 4H), 3.60 – 3.51 (m, 3H), 2.53 – 2.38 (m, 1H), 2.26 (ddd, *J* = 15.8, 9.2, 7.0 Hz, 1H), 2.14 (ddd, *J* = 13.8, 8.4, 5.5 Hz, 1H), 2.02 – 1.90 (m, 1H), 1.73 (ddd, *J* = 13.4, 4.6, 2.0 Hz, 1H), 1.63 – 1.53 (m, 4H), 1.44 – 1.18 (m, 9H), 0.89 (s, 9H), 0.88 (s, 9H), 0.88 (s, 9H), 0.87 (s, 9H), 0.06 (s, 3H), 0.05 (s, 6H), 0.04 (s, 6H), 0.04 – 0.03 (m, 9H). ^{13}C NMR (101 MHz, $CDCl_3$) δ 174.4, 83.9, 78.2, 75.3, 74.4, 72.8, 67.0, 51.6, 38.4, 31.3, 30.1, 30.0 (two peaks), 29.8, 26.9, 26.6, 26.1, 26.0, 26.0, 25.9, 25.7, 18.6, 18.2, 18.1, 18.1, –4.0, –4.0, –4.3, –4.5, –4.6, –5.0, –5.0, –5.1. IR (film) $\tilde{\nu}$ 2952, 2929, 2896, 2857,

1744, 1472, 1463, 1361, 1255, 1168, 1090, 1006, 938, 835, 774 cm^{-1}. HRMS (ESI) *m/z* calcd. for $C_{41}H_{88}O_7Si_4Na$ $[M+Na]^+$: 827.54993, found: 827.55009.

Compound 45. A Schlenk flask was charged with compound **43** (2.05 g, 3.09 mmol) and EtOH (10 mL) before an aqueous suspension of Raney®-nickel 2800 (10 mL) was added. The suspension was stirred at room temperature under an atmosphere of dihydrogen (balloon) for 7 d. The mixture was filtered through a short pad of Celite, rinsing with EtOAc. The combined filtrates were concentrated under reduced pressure to give a mixture of compound **45** and compound **41**. An aliquot was subjected to flash chromatography (silica; hexane/EtOAc = 10:1) to give an analytically pure sample of the title compound as a colorless oil. $[\alpha]_D^{20}$ = –8.7° (*c* = 0.54, $CHCl_3$). ^1H NMR (400 MHz, $CDCl_3$) δ 7.35 – 7.24 (m, 5H), 4.60 (d, *J* = 12.2 Hz, 1H), 4.53 (d, *J* = 12.2 Hz, 1H), 4.31 – 4.20 (m, 1H), 4.17 (ddd, *J* = 5.7, 3.7, 2.1 Hz, 1H), 4.10 (ddt, *J* = 8.5, 6.6, 5.3 Hz, 1H), 3.80 – 3.70 (m, 1H), 3.69 – 3.55 (m, 6H), 3.46 (dd, *J* = 9.6, 5.5 Hz, 1H), 2.57 – 2.35 (m, 2H), 2.27 – 2.14 (m, 2H), 1.94 – 1.83 (m, 2H), 1.67 (ddd, *J* = 13.3, 5.1, 2.0 Hz, 2H), 1.63 – 1.53 (m, 2H), 1.48 (ddd, *J* = 12.9, 7.4, 3.8 Hz, 2H), 1.44 – 1.35 (m, 2H), 1.31 (dt, *J* = 11.0, 4.1 Hz, 2H), 0.88 (s, 9H), 0.86 (s, 9H), 0.05 (s, 6H), 0.03 (s, 6H). ^{13}C NMR (101 MHz, $CDCl_3$) δ 174.4, 138.6, 128.5, 127.9, 127.6, 84.0, 81.5, 77.7, 76.5, 73.8, 73.4, 73.4, 72.7, 51.6, 41.7, 38.9, 36.7, 31.0, 29.7, 26.7, 26.7, 25.9, 25.9, 25.4, 18.2, 18.2, –4.3, –4.4, –4.9, –5.0. IR (film) $\tilde{\nu}$ 2950, 2929, 2898, 2856, 1740, 1471, 1462, 1438, 1362, 1255, 1196, 1169, 1098, 1074, 1006, 938, 865, 836, 775, 736, 698 cm^{-1}. HRMS (ESI) *m/z* calcd. for $C_{36}H_{64}O_7Si_2Na$ $[M+Na]^+$: 687.40828, found: 687.40825.

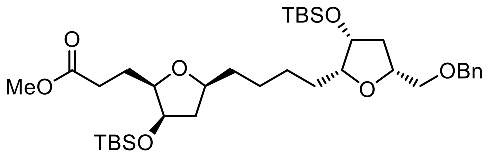

Compound 41. A solution of the crude product formed upon hydrogenation of compound **43** [*see above*] in EtOAc (30 mL) was added dropwise to a suspension of $Pd(OH)_2/C$ (20 wt %, 0.65 g) in EtOAc (30 mL). The mixture was stirred under an atmosphere of dihydrogen (H_2 balloon) at room temperature for 2 h before it was filtered through a short pad of Celite, rinsing with EtOAc. The combined filtrates were concentrated under reduced pressure and the residue purified by flash chromatography (silica; hexane/EtOAc, 2:1 to EtOAc 100%) to give the title compound as a colorless oil (1.49 g, 84%). $[\alpha]_D^{20}$ = –19.3° (*c* = 0.97, $CHCl_3$). ^1H NMR (400 MHz, $CDCl_3$) δ 4.23 (ddd, *J* = 6.5, 4.7, 3.7 Hz, 1H), 4.17 – 4.07 (m, 2H), 3.78 – 3.67 (m, 2H), 3.65 – 3.58 (m, 5H), 3.52 (dt, *J* = 11.4, 4.8 Hz, 1H), 2.56 (t, *J* = 5.5 Hz, 1H), 2.50 – 2.33 (m, 2H), 2.28 – 2.15 (m, 2H), 1.91 – 1.83 (m, 2H), 1.77 (ddd, *J* = 13.6, 4.2, 1.3 Hz, 1H), 1.72 – 1.58 (m, 2H), 1.57 – 1.37 (m, 5H), 1.34 – 1.23 (m, 2H), 0.88 (s, 9H), 0.86 (s, 9H), 0.07 (s, 3H), 0.05 (s, 3H), 0.03 (s, 6H). ^{13}C NMR (101 MHz, $CDCl_3$) δ 174.3, 84.0, 81.5, 77.8, 77.6, 73.3, 73.0, 65.1, 51.5, 41.7, 37.6, 36.6, 31.0, 29.5, 26.7, 26.6, 25.9, 25.9, 25.4, 18.2, 18.2, –4.4, –4.5, –5.0. IR (film) $\tilde{\nu}$ 3467, 2950, 2930, 2897, 2857, 1740, 1463, 1438, 1362, 1254, 1193, 1170,

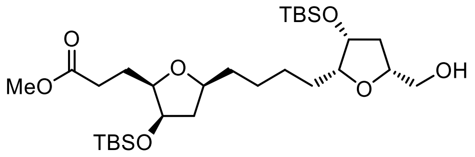

1071, 1006, 938, 836, 775 cm^{-1}. HRMS (ESI) *m/z* calcd. for $C_{29}H_{58}O_7Si_2Na$ $[M+Na]^+$: 597.36133, found: 597.36173.

Compound 46. Triethylamine (0.85 mL, 6.09 mmol) and methanesulfonyl chloride (0.35 mL, 4.57 mmol) were added to a solution of alcohol **41** (1.75 g, 3.04 mmol) in anhydrous CH_2Cl_2 (10 mL) at 0 °C (bath temperature). Stirring was continued at 0 °C for 2 h before the reaction was quenched with H_2O (20 mL). The organic layer was separated and the aqueous phase extracted with CH_2Cl_2 (3 x 20 mL). The combined organic layers were dried over $MgSO_4$ and concentrated under reduced pressure to give the mesylate ester as a pale-yellow oil that was used in the next step without purification. An analytically pure sample was obtained by flash chromatography (silica; hexanes/EtOAc, 2:1). The compound showed the following analytical and spectral properties: $[\alpha]_D^{20}$ = –25.6° (*c* = 0.98, $CHCl_3$). ^1H NMR (400 MHz, $CDCl_3$) δ 4.40 – 4.30 (m, 1H), 4.28 – 4.08 (m, 4H), 3.79 – 3.59 (m, 6H), 3.05 (s, 3H), 2.55 – 2.36 (m, 2H), 2.30 – 2.14 (m, 2H), 1.95 – 1.82 (m, 2H), 1.71 (ddd, *J* = 13.8, 3.7, 1.6 Hz, 1H), 1.67 – 1.55 (m, 3H), 1.49 (ddd, *J* = 12.9, 7.2, 3.8 Hz, 2H), 1.45 – 1.36 (m, 2H), 1.34 – 1.28 (m, 2H), 0.90 (s, 9H), 0.88 (s, 9H), 0.11 – 0.01 (m, 12H). ^{13}C NMR (101 MHz, $CDCl_3$) δ 174.4, 84.7, 81.5, 77.6, 75.1, 73.3, 72.9, 72.4, 51.6, 41.7, 38.1, 37.8, 36.7, 31.0, 29.7, 26.6 (two peaks), 25.9, 25.9, 25.4, 18.2, 18.2, –4.3, –4.4, –4.9, –5.0. IR (film) $\tilde{\nu}$ 2951, 2930, 2900, 2857, 1738, 1471, 1463, 1439, 1359, 1255, 1175, 1073, 1003, 954, 836, 776 cm^{-1}. HRMS (ESI) *m/z* calcd. for $C_{30}H_{60}O_9SSi_2Na$ $[M+Na]^+$: 675.33888, found: 675.33921.

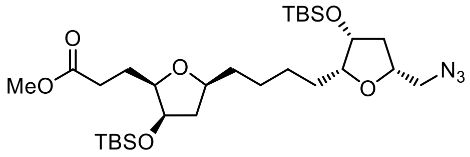

An oven-dried flask was charged with the crude mesylate (3.04 mmol), DMF (20 mL) and sodium azide (0.60 g, 9.13 mmol). The resulting mixture was stirred at 100 °C (bath temperature) for 24 h before H_2O (200 mL) was added. The aqueous phase was extracted with EtOAc (3 x 30 mL), the combined organic layers were washed with brine (100 mL), dried over Na_2SO_4 and concentrated under reduced pressure. The residue was purified by flash chromatography (silica; hexane/EtOAc, 5:1) to give the title compound as a colorless oil (1.44 g, 79% over two steps). $[\alpha]_D^{20}$ = –40.6° (*c* = 0.99, $CHCl_3$). ^1H NMR (400 MHz, $CDCl_3$) δ 4.24 (ddd, *J* = 6.5, 4.8, 3.8 Hz, 1H), 4.18 (ddd, *J* = 5.3, 3.6, 1.8 Hz, 1H), 4.08 (ddt, *J* = 9.0, 7.0, 4.5 Hz, 1H), 3.79 – 3.70 (m, 1H), 3.70 – 3.66 (m, 1H), 3.65 (s, 3H), 3.64 – 3.59 (m, 1H), 3.41 (dd, *J* = 12.4, 7.0 Hz, 1H), 3.22 (dd, *J* = 12.3, 4.8 Hz, 1H), 2.54 – 2.34 (m, 2H), 2.26 – 2.16 (m, 2H), 1.95 – 1.82 (m, 2H), 1.72 – 1.68 (m, 1H), 1.67 – 1.59 (m, 2H), 1.59 – 1.52 (m, 1H), 1.48 (ddd, *J* = 12.9, 7.4, 3.8 Hz, 2H), 1.45 – 1.36 (m, 2H), 1.36 – 1.23 (m, 2H), 0.89 (s, 9H), 0.88 (s, 9H), 0.07 – 0.02 (m, 12H). ^{13}C NMR (101 MHz, $CDCl_3$) δ 174.4, 84.3, 81.5, 77.7, 76.6, 73.3, 72.8, 55.6, 51.6, 41.7, 39.0, 36.7, 31.0, 29.8, 26.6, 26.6, 25.9, 25.9, 25.4, 18.2, 18.2, –4.4, –4.4, –4.9, –5.0. IR (film) $\tilde{\nu}$ 2951, 2929, 2899, 2857, 2097, 1740, 1471, 1463, 1438, 1361, 1255, 1170, 1071, 1006, 938, 835, 774 cm^{-1}. HRMS (ESI) *m/z* calcd. for $C_{29}H_{57}N_3O_6Si_2Na$ $[M+Na]^+$: 622.36781, found: 622.36785.

Compound S16. An oven-dried flask was charged with Pd/C (10 wt%, 138.0 mg) and MeOH (23 mL).

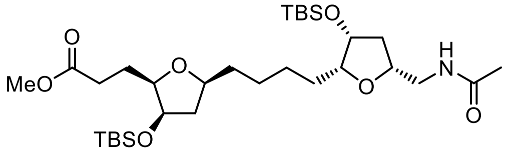

The suspension was stirred at 0 °C (bath temperature) while a solution of azide **46** (1.38 g, 2.30 mmol) in MeOH (23 mL) was added dropwise, followed by the addition of Ac_2O (0.33 mL, 3.50 mmol). The mixture was then stirred under an atmosphere of dihydrogen (balloon) at room temperature for 6 h. The mixture was filtered through a short pad of Celite, rinsing with EtOAc. The combined filtrates were concentrated under reduced pressure and the residue was purified by flash chromatography (silica; hexane/EtOAc, 1:1 to 100% EtOAc) to give the title compound as a colorless oil (1.27 g, 90%). $[\alpha]_D^{20}$ = –25.3° (*c* = 0.93, $CHCl_3$). ^1H NMR (400 MHz, $CDCl_3$) δ 6.05 (br, 1H), 4.30 – 4.20 (m, 1H), 4.17 (ddt, *J* = 4.7, 3.3, 1.5 Hz, 1H), 4.04 (dtd, *J* = 9.0, 5.6, 3.0 Hz, 1H), 3.81 – 3.72 (m, 1H), 3.66 (s, 3H), 3.65 – 3.52 (m, 3H), 3.23 (ddd, *J* = 13.8, 6.1, 4.6 Hz, 1H), 2.54 – 2.35 (m, 2H), 2.31 – 2.18 (m, 2H), 1.99 (s, 3H), 1.94 – 1.84 (m, 2H), 1.68 – 1.57 (m, 4H), 1.49 (ddd, *J* = 12.9, 7.2, 3.7 Hz, 2H), 1.46 – 1.37 (m, 2H), 1.32 (q, *J* = 4.3 Hz, 2H), 0.89 (s, 9H), 0.89 (s, 9H), 0.10 – 0.02 (m, 12H). ^{13}C NMR (101 MHz, $CDCl_3$) δ 174.3, 170.4, 84.1, 81.5, 77.6, 75.7, 73.3, 73.2, 51.6, 44.0, 41.7, 39.0, 36.7, 31.0, 29.5, 26.7, 26.6, 26.0, 25.9, 25.4, 23.5, 18.4, 18.2, –4.4 (2 x), –4.8, –4.9. IR (film) $\tilde{\nu}$ 3308, 2951, 2930, 2897, 2857, 1740, 1658, 1547, 1471, 1463, 1438, 1365, 1255, 1195, 1170, 1074, 1006, 939, 836, 775 cm^{-1}. HRMS (ESI) *m/z* calcd. for $C_{31}H_{61}NO_7Si_2Na$ $[M+Na]^+$: 638.38788, found: 638.38833.

Compound 47. An oven-dried flask was charged with acetamide **S16** (1.55 g, 2.52 mmol), Ac_2O (50 mL)

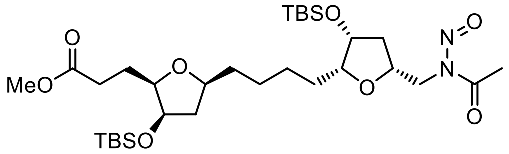

and AcOH (10 mL). The mixture was stirred at 0 °C (bath temperature) while $NaNO_2$ (9.03 g, 130.85 mmol) was added in small portions. Stirring was continued at 0 °C for 7 h before the reaction was quenched with ice-water. The mixture was extracted with EtOAc (3 x 80 mL), the combined extracts were washed with aq. $NaHCO_3$ solution (5% *w/w*, 3 x 200 mL), dried over Na_2SO_4 and concentrated under reduced pressure. The residue was purified by flash chromatography (silica; hexane/EtOAc, 5:1 to 3:1) to give the title compound as a yellow oil (1.36 g, 84%). $[\alpha]_D^{20}$ = –65.4° (*c* = 0.89, $CHCl_3$). ^1H NMR (400 MHz, $CDCl_3$) δ 4.36 (dd, *J* = 13.1, 8.0 Hz, 1H), 4.24 (ddd, *J* = 6.5, 4.8, 3.8 Hz, 1H), 4.14 (ddd, *J* = 5.3, 3.7, 1.9 Hz, 1H), 4.01 – 3.89 (m, 1H), 3.78 – 3.69 (m, 1H), 3.65 (s, 3H), 3.64 – 3.55 (m, 3H), 2.75 (s, 3H), 2.52 – 2.34 (m, 2H), 2.28 – 2.16 (m, 1H), 2.10 (ddd, *J* = 13.6, 8.5, 5.2 Hz, 1H), 1.95 – 1.80 (m, 2H), 1.67 – 1.43 (m, 6H), 1.41 – 1.21 (m, 4H), 0.93 (s, 9H), 0.87 (s, 9H), 0.07 (s, 3H), 0.05 (s, 3H), 0.04 (s, 6H). ^{13}C NMR (101 MHz, $CDCl_3$) δ 174.7, 174.4, 84.2, 81.4, 77.7, 73.7, 73.3, 72.7, 51.6, 43.7, 41.7, 39.2, 36.7, 31.0, 29.8, 26.6, 26.6, 25.9 (two peaks), 25.4, 22.7, 18.2 (two peaks), –4.4, –4.4, –4.9, –5.1. IR (film) $\tilde{\nu}$ 2952, 2930, 2899, 2857, 1739, 1510, 1472, 1463, 1437, 1375, 1255, 1197, 1169, 1115, 1074, 1006, 954, 836, 775 cm^{-1}. HRMS (ESI) *m/z* calcd. for $C_{31}H_{60}N_2O_8Si_2Na$ $[M+Na]^+$: 667.37804, found: 667.37774.

Alternative Route to Compound 41

Compound S17. DIBAL-H (1.0 M in hexane, 7.5 mL, 7.5 mmol) was slowly added to a solution of ester **35** (1.90 g, 5.0 mmol) in CH_2Cl_2 (20 mL) at –78 °C. After stirring at –78 °C for 1 h, the reaction was quenched with sat. aq. Rochelle salt solution (20 mL). The resulting mixture was vigorously stirred at room temperature for 1 h and the aqueous phase extracted with CH_2Cl_2 (3 x 20 mL). The combined organic layers were dried over $MgSO_4$ and concentrated under reduced pressure to give a colorless oil that was used in the next step without further purification.

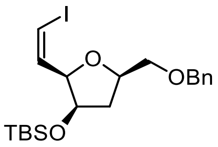

A Schlenk tube was charged iodomethyl triphenylphosphonium iodide (3.98 g, 7.5 mmol) and THF (20 mL) under Argon. The mixture was stirred at 0 °C (bath temperature) while a solution of KHMDS (1.50 g, 7.5 mmol) in THF (10 mL) was added dropwise. The resulting mixture was cooled to –78 °C (bath temperature) before the slow addition of a solution of the crude aldehyde in THF (10 mL). Stirring was continued at –78 °C for 30 min and for another 1 h at 0 °C. The reaction was quenched with sat. aq. NH_4Cl (20 mL) and the aqueous phase extracted with *tert*-butyl methyl ether (3 x 20 mL). The combined organic layers were washed with brine (20 mL), dried over Na_2SO_4 and concentrated under reduced pressure. The crude product was purified by flash chromatography (silica; hexane/EtOAc = 20:1) to give the title compound as a yellow oil (1.88 g, 79% over two steps). $[\alpha]_D^{20}$ = –88.2° (*c* = 0.93, $CHCl_3$). ^1H NMR (400 MHz, $CDCl_3$) δ 7.45 – 7.16 (m, 5H), 6.46 – 6.34 (m, 2H), 4.61 (d, *J* = 12.0 Hz, 1H), 4.55 (d, *J* = 12.2 Hz, 1H), 4.50 – 4.37 (m, 2H), 4.22 (ddt, *J* = 8.5, 6.7, 5.1 Hz, 1H), 3.67 (dd, *J* = 9.8, 6.7 Hz, 1H), 3.52 (dd, *J* = 9.6, 5.3 Hz, 1H), 2.30 (m, 1H), 1.88 – 1.70 (m, 1H), 0.85 (s, 9H), 0.03 (s, 3H), 0.02 (s, 3H). ^{13}C NMR (101 MHz, $CDCl_3$) δ 139.4, 138.4, 128.5, 127.9, 127.7, 86.5, 83.0, 77.6, 73.5, 73.5, 73.3, 38.8, 25.9, 18.2, –4.7, –4.8. IR (film) $\tilde{\nu}$ 3065, 3029, 2953, 2927, 2884, 2856, 1619, 1496, 1471, 1462, 1454, 1362, 1267, 1255, 1194, 1137, 1080, 1054, 1007, 939, 861, 835, 776, 736, 697 cm^{-1}. HRMS (ESI) *m/z* calcd. for $C_{20}H_{31}IO_3SiNa$ $[M+Na]^+$: 497.09794, found: 497.09813.

Compound S18. An oven-dried flask was charged with oxalyl chloride (1.10 mL, 12.60 mmol) and CH_2Cl_2 (30 mL). The solution was stirred at –78 °C for 5 min before dry DMSO (0.90 mL, 12.60 mmol) was slowly added. Stirring was continued at –78 °C for 30 min before a solution of alcohol **S15** (1.41 g, 4.43 mmol) in CH_2Cl_2 (15 mL) was added dropwise. After stirring at this temperature for 1 h, Et_3N (3.70 mL, 26.60 mmol) was slowly introduced. The mixture was stirred at –78 °C for 30 min and at 0 °C for another 30 min before ice water (20 mL) was added. The aqueous phase was extracted with CH_2Cl_2 (3 x 20 mL). The combined organic layers were washed with brine (50 mL), dried over Na_2SO_4 and concentrated under reduced pressure to give a yellow oil that was used in the next step without further purification.

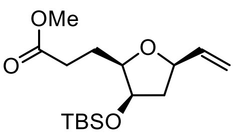

An oven-dried Schlenk tube was charged with $Ph_3P=CH_2$ (1.83 g, 6.64 mmol) and THF (10 mL) under Argon. This solution was stirred at 0 °C while a solution of the crude aldehyde in THF (10 mL) was added dropwise. Stirring was continued at 0 °C for 1 h and then at room temperature overnight. The reaction was quenched with H_2O (20 mL), the organic layer was separated and the aqueous phase extracted with *tert*-butyl methyl ether (3 x 20 mL). The combined organic layers were dried over $MgSO_4$ and concentrated under reduced pressure, and the resulting crude material was purified by flash chromatography (silica; hexane/EtOAc, 10:1) to give the title compound as a pale-yellow oil (1.12 g, 80% over two steps). $[\alpha]^{20}_{D}$ = –8.8° (*c* = 0.84, $CHCl_3$). ^1H NMR (400 MHz, $CDCl_3$) δ 5.93 (ddd, *J* = 17.5, 10.2, 7.5 Hz, 1H), 5.18 (ddd, *J* = 17.1, 1.7, 1.0 Hz, 1H), 5.05 (ddd, *J* = 10.3, 1.7, 0.9 Hz, 1H), 4.31 – 4.21 (m, 2H), 3.72 (dt, *J* = 8.0, 4.9 Hz, 1H), 3.66 (s, 3H), 2.52 – 2.38 (m, 2H), 2.32 (ddd, *J* = 13.1, 7.9, 6.0 Hz, 1H), 2.02 – 1.81 (m, 2H), 1.68 (ddd, *J* = 13.1, 6.4, 3.5 Hz, 1H), 0.89 (s, 9H), 0.06 (s, 3H), 0.06 (s, 3H). ^{13}C NMR (101 MHz, $CDCl_3$) δ 174.3, 140.0, 115.6, 82.0, 79.0, 73.5, 51.6, 42.3, 31.0, 25.9, 25.4, 18.2, –4.4, –4.9. IR (film) $\tilde{\nu}$ 2953, 2930, 2896, 2857, 1739, 1472, 1463, 1437, 1361, 1255, 1194, 1169, 1105, 1072, 1006, 922, 863, 835, 775 cm^{-1}. HRMS (ESI) *m/z* calcd. for $C_{16}H_{30}O_4SiNa$ $[M+Na]^+$: 337.18056, found: 337.18061.

Compound S19. A solution of 9-H-9-BBN dimer (366.0 mg, 1.50 mmol), alkene **S18** (0.95 g, 3.00 mmol) and THF (10 mL) was stirred at room temperature for 16 h. Aq. Cs_2CO_3 solution (3.0 M, 2.50 mL, 7.50 mmol) was added and the resulting mixture vigorously stirred for 20 min before alkenyl iodide **S17** (1.20 g, 2.50 mmol), DMF (10 mL), $AsPh_3$ (306.2 mg, 1.00 mmol) and $Pd(dppf)Cl_2$ (183.0 mg, 0.25 mmol) were introduced. After stirring for 10 h, the mixture was diluted with brine (100 mL), and the aqueous phase extracted with EtOAc (3 x 50 mL). The combined organic layers were washed with brine (100 mL), dried over $MgSO_4$ and concentrated under reduced pressure. The residue was purified by flash chromatography (silica; hexane/EtOAc = 10:1 to 3:1) to give the title compound as an oil (1.57 g, 95%). $[\alpha]^{20}_{D}$ = –24.6° (*c* = 1.06, $CHCl_3$). ^1H NMR (400 MHz, $CDCl_3$) δ 7.35 – 7.24 (m, 5H), 5.65 – 5.51 (m, 2H), 4.60 (d, *J* = 12.2 Hz, 1H), 4.53 (d, *J* = 12.2 Hz, 1H), 4.50 – 4.43 (m, 1H), 4.24 (ddd, *J* = 6.3, 4.7, 3.5 Hz, 1H), 4.21 – 4.12 (m, 2H), 3.82 – 3.72 (m, 1H), 3.69 – 3.64 (m, 4H), 3.62 (dt, *J* = 7.9, 4.9 Hz, 1H), 3.51 (dd, *J* = 9.6, 5.8 Hz, 1H), 2.53 – 2.35 (m, 2H), 2.31 – 2.21 (m, 2H), 2.20 – 2.10 (m, 2H), 1.96 – 1.82 (m, 2H), 1.81 – 1.71 (m, 2H), 1.59 – 1.45 (m, 2H), 0.89 (s, 9H), 0.85 (s, 9H), 0.05 (s, 6H), 0.00 (s, 3H), 0.00 (s, 3H). ^{13}C NMR (101 MHz, $CDCl_3$) δ 174.3, 138.6, 133.1, 128.4, 127.9, 127.6, 127.0, 81.6, 79.2, 76.9, 76.7, 74.3, 73.8, 73.5, 73.4, 51.6, 41.6, 39.3, 36.3, 31.0, 25.9, 25.9, 25.4, 24.7, 18.3, 18.2, –4.4, –4.7, –4.8, –4.9. IR (film) $\tilde{\nu}$ 2952, 2929, 2897, 2856, 1740, 1471, 1462, 1438, 1362, 1254, 1193, 1171, 1100, 1073, 1006, 938, 835, 775 cm^{-1}. HRMS (ESI) *m/z* calcd. for $C_{36}H_{62}O_7Si_2Na$ $[M+Na]^+$: 685.39263, found: 685.39254.

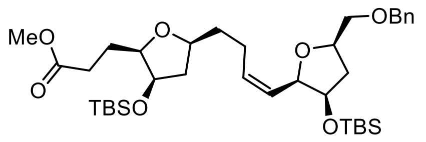

Compound 41. A suspension containing Pd/C (10 wt %, 159.0 mg) and benzyl ether **S19** (794.0 mg, 1.20 mmol) in EtOAc (24 mL) was stirred at room temperature under an atmosphere of dihydrogen (balloon) at room temperature for 1 h. The mixture was filtered through a short pad of Celite, rinsing with EtOAc. The combined filtrates were concentrated under reduced pressure and the residue purified by flash chromatography (silica; hexane/EtOAc, 2:1 to EtOAc 100%) to give the title compound as a colorless oil (520.5 mg, 76%), along a partially TBS-deprotected by-product (62.6 mg, 11%). See above for the analytical and spectral data of product **41**.

Sub-Fragment B2

Compound 48. A mixture of tri-*O*-acetyl-D-glucal (5.45 g, 20.0 mmol) and K_2CO_3 (276.0 mg, 2.0 mmol) in methanol (20 mL) was stirred at room temperature for 4 h. The solvent was removed under reduced pressure and remaining methanol was co-evaporated with $CHCl_3$ (3 x 10 mL) to afford a pale brown syrup.

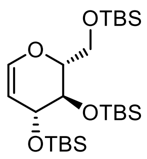

Imidazole (13.62 g, 200.0 mmol) was added to a solution of this crude product in DMF (20 mL). TBSCl (15.07 g, 100.0 mmol) was added at 0 °C (bath temperature) and the resulting solution was stirred at room temperature for 18 h. The mixture was poured into water (200 mL) and the product extracted with EtOAc (3 x 100 mL). The combined organic layers were washed with brine (200 mL), dried over $MgSO_4$ and concentrated under reduced pressure. The by-product TBSOH was removed by azeotroping the material with toluene (3 x 10 mL) and the residue was then purified by flash chromatography (silica; cyclohexane/CH_2Cl_2, 10:1 to 4:1) to give the title compound as a colorless oil (9.50 g, 97%). $[\alpha]_D^{20}$ = –33.2° (*c* = 0.82, $CHCl_3$). ^{1}H NMR (400 MHz, $CDCl_3$) δ 6.32 (dd, *J* = 6.2, 0.9 Hz, 1H), 4.69 (ddd, *J* = 6.3, 4.4, 1.3 Hz, 1H), 3.99 (dtd, *J* = 7.4, 3.6, 1.5 Hz, 1H), 3.93 (dd, *J* = 11.2, 7.4 Hz, 1H), 3.89 (tdd, *J* = 4.3, 1.4, 0.8 Hz, 1H), 3.79 (td, *J* = 3.6, 1.3 Hz, 1H), 3.76 (dd, *J* = 11.2, 3.4 Hz, 1H), 0.90 (s, 9H), 0.89 (s, 9H), 0.89 (s, 9H), 0.10 (s, 6H), 0.08 (s, 3H), 0.08 (s, 3H), 0.06 (s, 3H), 0.05 (s, 3H). ^{13}C NMR (101 MHz, $CDCl_3$) δ 143.1, 101.5, 80.2, 70.4, 66.9, 61.9, 26.1, 26.0, 26.0, 18.6, 18.2, 18.2, –4.1, –4.2, –4.2, –4.6, –5.0, –5.1. IR (film) $\tilde{\nu}$ 2955, 2929, 2886, 2858, 1649, 1472, 1462, 1253, 1099, 1069, 1006, 958, 876, 836, 776 cm^{-1}. HRMS (ESI) *m/z* calcd. for $C_{24}H_{52}O_4Si_3Na$ $[M+Na]^+$: 511.30656, found: 511.30699.

Preparation of Lithium Naphthalenide. An oven-dried Schlenk tube was charged with tetrahydrofuran (45 mL), naphthalene (5.78 g, 45 mmol) and lithium pellets (314 mg, 45 mmol). The mixture, which rapidly developed a deep-green color, was stirred overnight prior to use, constituting a 1 M solution of lithium naphthalenide.

Compound 49. An oven-dried 500 mL flask equipped with a stir bar was charged with compound **48**

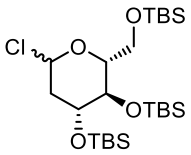

(2.45 g, 5.0 mmol) and THF (50 mL). The mixture was stirred at 0 °C while HCl (4.0 M in dioxane, 6.3 mL, 25.0 mmol) was added dropwise. The solution was then stirred at room temperature for 1 h before all volatile materials were removed at room temperature under reduced pressure and the residue was carefully dried under high vacuum with vigorous stirring for at least 20 h prior to use.

Compound 50. A solution of lithium naphthalenide (1 M in THF, 15.0 mL, 15.0 mmol) was quickly added

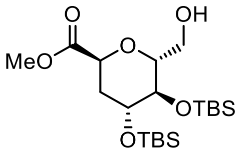

to a solution of the crude glycosyl chloride **49** (5.0 mmol) in THF (50 mL) at –95 °C (acetone/liquid nitrogen bath). After stirring at –95 °C for 15 min, Mander's reagent (0.80 mL, 10.0 mmol) was added dropwise and stirring continued at –95 °C for 1 h and then at room temperature for another 1 h. Sat. aq. NH_4Cl solution (20 mL) was added to the orange mixture, the organic layer was separated, and the aqueous phase extracted with *tert*-butyl methyl ether (3 x 20 mL). The combined organic layers were dried over Na_2SO_4 and concentrated under reduced pressure. The residue was purified by flash chromatography (silica; cyclohexane 100% to cyclohexane/EtOAc = 10:1) to give the anomeric methyl ester as a yellow oil.

PTSA·H_2O (95.1 mg, 0.50 mmol) was added to a solution of this ester in MeOH (20 mL) and the resulting mixture was stirred at 0 °C for 2 h. The mixture was diluted with brine (30 mL) and extracted with EtOAc (3 x 30 mL). The combined organic layers were washed with brine (50 mL), dried over Na_2SO_4 and concentrated under reduced pressure. The residue was purified by flash chromatography (silica; cyclohexane/EtOAc, 10:1 to 5:1) to give the title compound as a pale-yellow oil (1.19 g, 55% over three steps). $[\alpha]_D^{20}$ = + 19.7° (*c* = 0.93, $CHCl_3$). ^{1}H NMR (400 MHz, $CDCl_3$) δ 4.52 (dd, *J* = 6.8, 4.5 Hz, 1H), 3.88 (ddd, *J* = 11.4, 6.7, 4.5 Hz, 1H), 3.76 (s, 3H), 3.75 – 3.64 (m, 3H), 3.40 (t, *J* = 5.6 Hz, 1H), 2.31 (ddd, *J* = 13.4, 6.8, 3.5 Hz, 1H), 2.14 (dd, *J* = 7.7, 4.6 Hz, 1H), 1.78 (ddd, *J* = 13.6, 7.7, 4.6 Hz, 1H), 0.90 (s, 9H), 0.88 (s, 9H), 0.10 (s, 3H), 0.08 (s, 3H), 0.07 (s, 6H). ^{13}C NMR (101 MHz, $CDCl_3$) δ 172.3, 79.3, 70.9, 70.6, 68.9, 61.6, 52.3, 33.4, 26.1, 26.0, 18.2, 18.1, –3.7, –3.8, –4.6, –4.7. IR (film) $\tilde{\nu}$ 3495, 2954, 2930, 2893, 2857, 1752, 1472, 1463, 1253, 1098, 1028, 1006, 938, 880, 835, 777 cm^{-1}. HRMS (ESI) *m/z* calcd. for $C_{20}H_{42}O_6Si_2Na$ $[M+Na]^+$: 457.24122, found: 457.24129.

Methoxymethyl Allyl Ether (51). Chloromethyl methyl ether (5.00 mL, 62.5 mmol) was added dropwise at 0 °C (bath temperature) to a mixture of allyl alcohol (3.40 mL, 50.0 mmol) and Hünig's base (13.06 mL, 75.0 mmol). After stirring at room temperature for 12 h, the reaction was quenched with water (30 mL) and the aqueous phase extracted with CH_2Cl_2 (3 x 10 mL). The combined organic layers were washed with brine (50 mL), dried over Na_2SO_4, filtered and distilled under atmospheric pressure. The fraction boiling in the range between 75 °C to 90 °C was collected, providing methoxymethyl allyl ether as a colorless oil (3.18 g, 62%). ^{1}H NMR (400 MHz, $CDCl_3$) δ 5.93 (ddt, *J* =

17.2, 10.4, 5.7 Hz, 1H), 5.30 (dq, J = 17.2, 1.6 Hz, 1H), 5.19 (ddt, J = 10.4, 1.9, 1.3 Hz, 1H), 4.65 (s, 2H), 4.06 (dt, J = 5.6, 1.4 Hz, 2H), 3.37 (s, 3H). The ^{1}H NMR data was consistent with previously reported data.[6]

Compound 52. $NaHCO_3$ (3.36 g, 40.0 mmol) and Dess-Martin periodinane (8.48 g, 20.0 mmol) were added at 0 °C (bath temperature) to a solution of alcohol **50** (4.35 g, 10.0 mmol) in CH_2Cl_2 (50 mL). The resulting mixture was stirred at room temperature for 2 h before sat. aq. $Na_2S_2O_3$ solution (50 mL) and H_2O (50 mL) were added. The organic layer was separated and the aqueous phase extracted with CH_2Cl_2 (3 x 50 mL). The combined organic layers were washed with sat. aq. $NaHCO_3$ solution (3 x 100 mL), dried over Na_2SO_4 and concentrated under reduced pressure to give a yellow oil that was used in the next step without further purification.

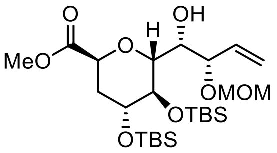

An oven-dried flask was charged with methoxymethyl allyl ether **51** (1.23 g, 12.0 mmol) and THF (20 mL). The solution was cooled to –78 °C (bath temperature) before *sec*-BuLi (1.4 M in cyclohexane, 7.15 mL, 10.0 mmol) was added dropwise. After stirring the resulting yellow solution at –78 °C for 30 min, (–)-B-methoxydiisopinocamphenylborane solution (0.4 M in THF, pre-dried over 3Å molecular sieves, 25.0 mL, 10.0 mmol) was introduced. The mixture was stirred at –78 °C for 1 h and then cooled to –95 °C (acetone/liquid nitrogen bath) before boron trifluoride etherate (1.90 mL, 15.0 mmol) was added dropwise. Immediately afterwards, a solution of the freshly prepared aldehyde (10.0 mmol) in THF (10 mL) was added dropwise. Stirring was continued at –95 °C for 3 h before the mixture was allowed to slowly warm to room temperature over the course of 12 h. For work up, the mixture was cooled to 0 °C (bath temperature) and sat. aq. $NaHCO_3$ solution (50.0 mL) was added, followed by the addition of aq. H_2O_2 (35% *w/w*, 25.0 mL). The mixture was vigorously stirred at room temperature for 30 min. *tert*-Butyl methyl ether (20 mL) was added and the organic layer was collected. The aqueous phase was extracted with *tert*-butyl methyl ether (3 x 50 mL). The combined organic layers were washed with brine (100 mL), dried over Na_2SO_4 and concentrated under reduced pressure. The residue was purified by flash chromatography (silica; cyclohexane/EtOAc = 10:1 to 5:1) to give a mixture of the title compound and isopinocampheol. Isopinocampheol was removed by Kugelrohr distillation (< 0.1 mbar, ≈70 °C) to leave the desired product as a pale-yellow oil (4.39 g, 82% over two steps, dr > 20:1). $[\alpha]_D^{20}$ = + 25.5° (c = 0.99, $CHCl_3$). ^{1}H NMR (400 MHz, $CDCl_3$) δ 5.90 (ddd, J = 17.4, 10.4, 7.1 Hz, 1H), 5.31 (ddd, J = 17.4, 1.8, 1.1 Hz, 1H), 5.25 (ddd, J = 10.5, 1.8, 0.9 Hz, 1H), 4.72 (d, J = 6.5 Hz, 1H), 4.65 (d, J = 6.5 Hz, 1H), 4.36 (dd, J = 12.0, 2.3 Hz, 1H), 4.30 – 4.23 (m, 2H), 3.96 (dt, J = 3.5, 1.3 Hz, 1H), 3.90 (q, J = 3.3 Hz, 1H), 3.78 (d, J = 10.0 Hz, 1H), 3.73 (s, 3H), 3.38 (s, 3H), 2.23 (d, J = 7.9 Hz, 1H), 2.19 (ddd, J = 13.3, 11.8, 2.3 Hz, 1H), 1.70 (dtd, J = 13.2, 2.5, 1.1 Hz, 1H), 0.87 (s, 9H), 0.87 (s, 9H), 0.09 (s, 3H), 0.07 (s, 3H), 0.06 (s, 3H), 0.05 (s, 3H). ^{13}C NMR (101 MHz, $CDCl_3$) δ 172.4, 136.0, 118.3, 94.8, 79.2, 76.1, 70.8, 69.4, 67.0,

66.0, 56.0, 52.2, 30.9, 26.0, 25.9, 18.2, 18.1, –4.5, –4.7, –4.8, –4.9. IR (film) ṽ 3510, 2953, 2930, 2894, 2857, 1741, 1472, 1439, 1255, 1090, 1031, 1005, 922, 878, 833, 809, 776 cm^{-1}. HRMS (ESI) *m/z* calcd. for $C_{25}H_{50}O_8Si_2Na$ $[M+Na]^+$: 557.29365, found: 557.29339.

Table S8. Formation of Compound **52** by Substrate-Controlled *syn*-Oxyallylation

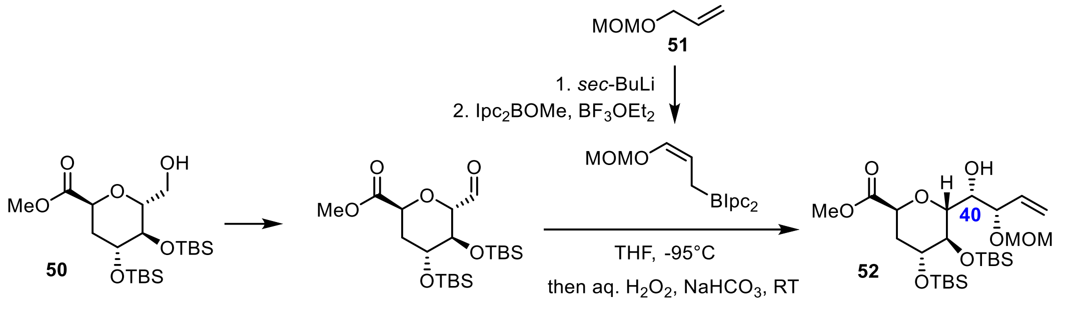

Entry	Reagent	dr	Yield (%)	Scale
1	(–)-Ipc_2BOMe	> 20:1	82%	10 mmol
2	(+)-Ipc_2BOMe	> 20:1	78%	0.4 mmol

Surprisingly, the *syn*-oxyallylation reaction was found to be under strict substrate- rather than reagent-control since the choice of Ipc_2BOMe has no notable impact on the observed stereoinduction, see Table S8

Compound S20. TBAF (1.0 M in THF, 0.22 mL, 220.0 µmol) was added to a solution of compound **52** (40.0 mg, 74.8 µmol) in THF (2.0 mL). The mixture was stirred at room temperature for 2 h before $CaCO_3$ (100 mg), DOWEX 50WX8-400 (300 mg), and MeOH (2.0 mL) were added. The suspension was stirred at for 1 h, all insoluble materials were filtered off through a pad of Celite, and the filter cake was thoroughly rinsed with MeOH. The combined filtrates were concentrated under reduced pressure and the residue purified by flash chromatography (silica; CH_2Cl_2/MeOH, 20:1 to 15:1) to give the title compound as a colorless oil (19.3 mg, 84%). $[\alpha]_D^{20}$ = + 53.8° (*c* = 0.50, CH_3OH). ^1H NMR (400 MHz, [D_4]-MeOH) δ 5.92 (ddd, *J* = 17.4, 10.5, 7.6 Hz, 1H), 5.39 – 5.23 (m, 2H), 4.73 (d, *J* = 6.6 Hz, 1H), 4.70 (d, *J* = 6.6 Hz, 1H), 4.54 (dd, *J* = 6.1, 2.5 Hz, 1H), 4.30 (ddt, *J* = 7.6, 4.3, 1.0 Hz, 1H), 3.81 – 3.70 (m, 5H), 3.59 – 3.47 (m, 2H), 3.39 (s, 3H), 2.32 (ddd, *J* = 13.4, 4.2, 2.6 Hz, 1H), 1.85 – 1.71 (m, 1H). ^{13}C NMR (101 MHz, [D_4]-MeOH) δ 173.3, 137.3, 118.8, 96.2, 79.0, 77.9, 75.9, 74.6, 72.8, 70.5, 56.2, 52.5, 34.6. IR (film) ṽ 3410, 2926,

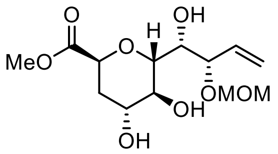

2854, 1744, 1438, 1215, 1122, 1029, 921, 857 cm^{-1}. HRMS (ESI) *m/z* calcd. for $C_{13}H_{22}O_8Na$ $[M+Na]^+$: 329.12069, found: 329.12077.

Compound 53. A solution of TMSBr (0.5 M in CH_2Cl_2, 81.0 mL, 40.5 mmol) was slowly added at –78 °C to a solution of compound **52** (4.33 g, 8.10 mmol) in CH_2Cl_2 (150 mL). The reaction mixture was warmed to –10 °C over the course of 5 h and stirred at this temperature for another 1 h. The reaction was then quenched with sat. aq. $NaHCO_3$ (100 mL) and the aqueous phase was extracted with CH_2Cl_2 (3 x 50 mL). The combined organic layers were washed with brine (100 mL), dried over Na_2SO_4, and concentrated under reduced pressure. The residue was purified by flash chromatography (silica; hexane/EtOAc, 5:1) to give the title compound as a pale-yellow oil (2.49 g, 62%). $[\alpha]_D^{20}$ = –6.8° (*c* = 0.81, $CHCl_3$). ^1H NMR (400 MHz, $CDCl_3$) δ 5.94 (ddd, *J* = 17.2, 10.5, 5.2 Hz, 1H), 5.39 (dt, *J* = 17.2, 1.6 Hz, 1H), 5.24 (dt, *J* = 10.6, 1.6 Hz, 1H), 4.47 (dd, *J* = 10.9, 3.2 Hz, 1H), 4.43 – 4.38 (m, 1H), 4.14 (ddd, *J* = 8.7, 7.4, 2.5 Hz, 1H), 3.91 – 3.84 (m, 2H), 3.79 (dd, *J* = 8.6, 2.2 Hz, 1H), 3.76 (s, 3H), 2.67 (d, *J* = 5.2 Hz, 1H), 2.41 (d, *J* = 7.5 Hz, 1H), 2.23 (ddd, *J* = 13.4, 11.0, 2.2 Hz, 1H), 1.81 – 1.75 (m, 1H), 0.90 (s, 9H), 0.88 (s, 9H), 0.11 (s, 3H), 0.11 (s, 3H), 0.07 (s, 6H). ^{13}C NMR (101 MHz, $CDCl_3$) δ 172.5, 137.7, 116.4, 79.3, 71.5, 71.1, 69.8, 68.4, 67.0, 52.4, 31.1, 26.0, 25.9, 18.1, 18.1, –4.5, –4.5, –4.5, –4.8. IR (film) $\tilde{\nu}$ 3473, 2954, 2930, 2895, 2858, 1744, 1472, 1439, 1257, 1087, 1005, 900, 881, 834, 810, 777 cm^{-1}. HRMS (ESI) *m/z* calcd. for $C_{23}H_{46}O_7Si_2Na$ $[M+Na]^+$: 513.26743, found: 513.26769.

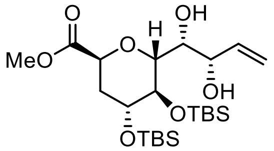

Mosher Ester Analysis: Compounds 55. Reaction of diol **53** and (*S*)-(+)-MTPA-Cl furnished the (*R*)-(+)-MTPA ester (*R*)-**55** as a colorless oil (5.2 mg, 72%). $[\alpha]_D^{20}$ = + 18.3° (*c* = 0.52, $CHCl_3$). ^1H NMR (600 MHz, $CDCl_3$) δ 7.57 – 7.52 (m, 2H), 7.41 – 7.37 (m, 3H), 6.00 (ddd, *J* = 17.3, 10.6, 6.9 Hz, 1H), 5.66 (ddt, *J* = 6.9, 3.3, 1.1 Hz, 1H), 5.47 (dt, *J* = 17.3, 1.2 Hz, 1H), 5.39 (dt, *J* = 10.6, 1.1 Hz, 1H), 4.46 – 4.41 (m, 1H), 4.39 (dd, *J* = 11.7, 2.6 Hz, 1H), 3.91 – 3.87 (m, 1H), 3.86 – 3.84 (m, 1H), 3.75 (s, 3H), 3.57 – 3.53 (m, 4H), 2.20 (ddd, *J* = 13.4, 11.7, 2.4 Hz, 1H), 1.97 (br, 1H), 1.76 – 1.70 (m, 1H), 0.89 (s, 9H), 0.85 (s, 9H), 0.10 (s, 6H), 0.02 (s, 3H), –0.03 (s, 3H). ^{13}C NMR (151 MHz, $CDCl_3$) δ 172.1, 165.4, 132.6, 132.3, 129.8, 128.5, 127.9, 123.6 (q, *J* = 288.5 Hz), 120.4, 84.9 (q, *J* = 27.5 Hz), 78.4, 76.6, 70.4, 69.4, 67.0, 66.4, 55.6, 52.2, 30.7, 25.9, 25.9, 18.1 (2 x), –4.7 (2 x), –4.8 (2 x). IR (film) $\tilde{\nu}$ 3506, 2953, 2929, 2857, 1755, 1674, 1463, 1362, 1256, 1187, 1170, 1093, 1017, 937, 876, 834, 811, 778 cm^{-1}. HRMS (ESI) *m/z* calcd. for $C_{33}H_{53}F_3O_9Si_2Na$ $[M+Na]^+$: 729.30724, found: 729.30720.

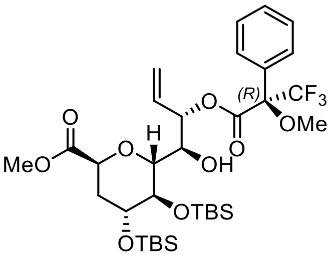

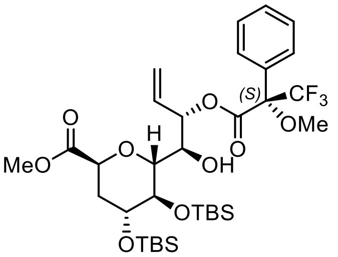

Analogously, the reaction of diol **53** with (*R*)-(–)-MTPA-Cl gave the corresponding (*S*)-(–)-MTPA ester (*S*)-**55** as a colorless oil (5.0 mg, 69%). $[\alpha]_D^{20}$ = –3.2° (*c* = 0.50, $CHCl_3$). ^1H NMR (600 MHz, $CDCl_3$) δ 7.58 – 7.54 (m, 2H), 7.41 – 7.37 (m, 3H), 5.89 (ddd, *J* = 17.2, 10.7, 6.3 Hz, 1H), 5.60 (ddt, *J* = 6.2, 3.5, 1.2 Hz, 1H), 5.38 – 5.31 (m, 2H), 4.49 – 4.40 (m, 2H), 3.91 (td, *J* = 3.6, 2.4 Hz, 1H), 3.87 (dt, *J* = 3.5, 1.3 Hz, 1H), 3.74 (s, 3H), 3.70 (d, *J* = 9.0 Hz, 1H), 3.58 – 3.54 (m, 3H), 2.22 (ddd, *J* = 13.4, 11.8, 2.5 Hz, 1H), 2.05 (br, 1H), 1.79 – 1.73 (m, 1H), 0.90 (s, 9H), 0.84 (s, 9H), 0.11 (s, 6H), 0.03 (s, 3H), –0.03 (s, 3H). ^{13}C NMR (151 MHz, $CDCl_3$) δ 172.1, 165.8, 132.5, 132.3, 129.8, 128.6, 127.9, 123.6 (q, *J* = 288.2 Hz), 119.7, 85.0 (q, *J* = 28.0 Hz), 78.5, 76.5, 70.3, 69.4, 67.0, 66.5, 55.6, 52.2, 30.6, 25.9, 25.8, 18.1, 18.0, –4.7, –4.8 (3 x). IR (film) $\tilde{\nu}$ 3587, 2954, 2930, 2857, 1754, 1463, 1362, 1256, 1182, 1091, 1017, 937, 876, 833, 809, 777 cm^{-1}. HRMS (ESI) *m/z* calcd. for $C_{33}H_{53}F_3O_9Si_2Na$ $[M+Na]^+$: 729.30724, found: 729.30701.

Table S9. Determination of absolute configuration of the stereogenic center at C41 set by Brown allylation via Mosher ester analysis.

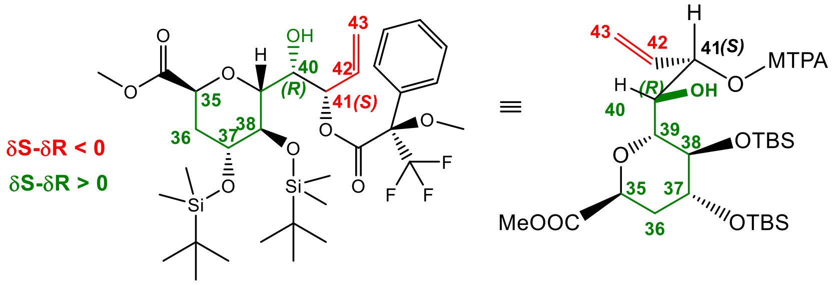

##	$\delta_{H\ NMR}$ (*S*-ester) (ppm)	$\delta_{H\ NMR}$ (*R*-ester) (ppm)	$\Delta\delta$ (δ_S -δ_R, ppm)
43-trans	5.35	5.47	–0.12
43-cis	5.34	5.39	–0.05
42	5.89	6.00	–0.11
41	5.60	5.66	–0.06
40	4.433	4.428	0.004
40'	2.056	1.970	0.086
39	3.70	3.56	0.14
38	3.87	3.85	0.02
37	3.91	3.88	0.03
36ax	2.22	2.20	0.02
36eq	1.76	1.73	0.03
35	4.46	4.39	0.07

Compound 54. 2,6-Lutidine (0.87 mL, 7.5 mmol) was added at 0 °C to a solution of diol **53** (1.47 g, 3.0

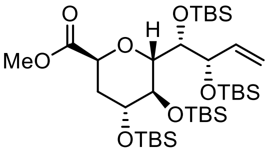

mmol) in CH_2Cl_2 (20 mL), followed by slow addition of TBSOTf (1.72 mL, 7.5 mmol). The resulting mixture was stirred at room temperature for 2 h before the reaction was quenched with sat. aq. $NaHCO_3$ solution (30 mL). The mixture was extracted with CH_2Cl_2 (3 x 30 mL), the combined extracts were washed with brine (50 mL), dried over $MgSO_4$ and concentrated under reduced pressure. The residue was purified by flash chromatography (silica; hexane/EtOAc, 20:1) to give the title compound as a colorless oil (2.16 g, quant.). $[\alpha]_D^{20}$ = –24.2° (*c* = 0.93, $CHCl_3$). ^1H NMR (400 MHz, $CDCl_3$) δ 6.12 (ddd, *J* = 17.4, 10.5, 6.8 Hz, 1H), 5.28 – 5.10 (m, 2H), 4.60 (dd, *J* = 12.3, 2.0 Hz, 1H), 4.40 (dd, *J* = 9.9, 2.2 Hz, 1H), 4.15 (ddt, *J* = 6.8, 2.4, 1.3 Hz, 1H), 3.95 – 3.88 (m, 1H), 3.73 – 3.65 (m, 5H), 2.12 (ddd, *J* = 13.3, 12.2, 2.4 Hz, 1H), 1.82 – 1.72 (m, 1H), 0.94 (s, 9H), 0.89 (s, 9H), 0.87 (s, 9H), 0.85 (s, 9H), 0.15 – 0.12 (m, 9H), 0.12 (s, 3H), 0.05 (s, 3H), 0.04 (s, 6H), –0.01 (s, 3H). ^{13}C NMR (101 MHz, $CDCl_3$) δ 173.0, 138.2, 116.8, 80.5, 78.2, 72.9, 70.5, 67.5, 66.3, 51.8, 31.5, 26.3, 26.1, 26.0 (two peaks), 18.3 (two peaks), 18.3, 18.1, –3.1, –3.7, –3.9, –4.2, –4.3, –4.8, –4.9, –4.9. IR (film) $\tilde{\nu}$ 2954, 2930, 2895, 2858, 1767, 1738, 1472, 1463, 1256, 1080, 1034, 1005, 880, 835, 811, 776 cm^{-1}. HRMS (ESI) *m/z* calcd. for $C_{35}H_{74}O_7Si_4Na$ $[M+Na]^+$: 741.44039, found: 741.44031.

Compound 56. Pyridinium *p*-toluenesulfonate (PPTS, 1.3 mg, 0.005 mmol) was added to a solution of

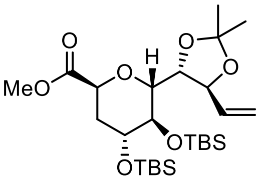

diol **53** (24.5 mg, 0.05 mmol) in 2,2-dimethoxy propane (0.5 mL). The mixture was stirred at room temperature overnight before the reaction was quenched with sat. aq. $NaHCO_3$ (5 mL). The mixture was extracted with CH_2Cl_2 (3 x 5 mL), the combined organic layers were washed with brine (10 mL), dried over Na_2SO_4, concentrated under reduced pressure, and the residue purified by flash chromatography (silica; hexane/EtOAc, 10:1) to give the title compound as a pale-yellow oil (21.1 mg, 80%). $[\alpha]_D^{20}$ = –6.0° (*c* = 0.48, $CHCl_3$). ^1H NMR (400 MHz, $CDCl_3$) δ 5.84 (ddd, *J* = 17.0, 10.4, 6.5 Hz, 1H), 5.43 (ddd, *J* = 17.2, 1.8, 0.8 Hz, 1H), 5.17 (ddd, *J* = 10.5, 1.8, 0.6 Hz, 1H), 4.49 – 4.38 (m, 2H), 4.32 (dd, *J* = 12.2, 2.3 Hz, 1H), 3.91 – 3.85 (m, 1H), 3.81 (d, *J* = 9.8 Hz, 1H), 3.78 (dt, *J* = 3.3, 1.1 Hz, 1H), 3.73 (s, 3H), 2.17 (ddd, *J* = 13.3, 12.0, 2.3 Hz, 1H), 1.71 (dtd, *J* = 13.3, 2.4, 1.1 Hz, 1H), 1.39 (s, 3H), 1.36 (s, 3H), 0.91 (s, 9H), 0.89 (s, 9H), 0.10 (s, 3H), 0.09 (s, 3H), 0.07 (s, 3H), 0.06 (s, 3H). ^{13}C NMR (101 MHz, $CDCl_3$) δ 172.3, 136.1, 117.9, 109.5, 82.4, 82.2, 76.9, 69.2, 67.4, 66.1, 52.2, 31.2, 27.4, 27.2, 26.0, 25.9, 18.1, 18.1, –4.7, –4.8, –4.9 (2 x). IR (film) $\tilde{\nu}$ 2955, 2929, 2894, 2857, 1765, 1738, 1462, 1453, 1379, 1257, 1091, 1066, 1034, 878, 835, 778 cm^{-1}. HRMS (ESI) *m/z* calcd. for $C_{26}H_{50}O_7Si_2Na$ $[M+Na]^+$: 553.29873, found: 553.29858.

Figure S6. Characteristic NOE's

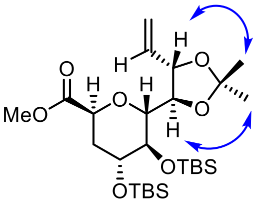

Compound 57. PTSA·H_2O (0.6 mg, 3.0 µmol) was added to a solution of triol **S20** (9.3 mg, 30.4 µmol), *p*-anisaldehyde dimethyl acetal (7.2 mg, 40.0 µmol) and DMF (1.0 mL). After stirring at room temperature for 24 h, the reaction was quenched with sat. aq. $NaHCO_3$ solution (10 mL) and the mixture was extracted with EtOAc (3 x 10 mL). The combined organic layers were washed with brine (10 mL), dried over Na_2SO_4, and concentrated under reduced pressure. The residue was purified by flash chromatography (silica; hexane/EtOAc = 3:1 to 1:1) to give the title compound as a white solid (13.1 mg, dr = 25:1, quant.). $[\alpha]_D^{20}$ = + 40.5° (*c* = 1.31, CH_2Cl_2). ^{1}H NMR (600 MHz, CD_2Cl_2) δ 7.42 – 7.37 (m, 2H), 6.91 – 6.86 (m, 2H), 6.04 (ddd, *J* = 17.3, 10.4, 7.7 Hz, 1H), 5.57 (s, 1H), 5.33 (ddd, *J* = 17.3, 1.8, 1.1 Hz, 1H), 5.25 (ddd, *J* = 10.4, 1.8, 1.0 Hz, 1H), 4.75 (d, *J* = 6.6 Hz, 1H), 4.72 (d, *J* = 6.6 Hz, 1H), 4.56 (dd, *J* = 6.8, 1.5 Hz, 1H), 4.28 (ddt, *J* = 7.6, 2.4, 1.0 Hz, 1H), 3.90 (dddd, *J* = 11.3, 9.0, 4.9, 2.0 Hz, 1H), 3.84 – 3.80 (m, 1H), 3.80 (s, 3H), 3.75 (s, 3H), 3.72 (dd, *J* = 9.1, 2.5 Hz, 1H), 3.44 (dd, *J* = 9.3, 9.3 Hz, 1H), 3.35 (s, 3H), 2.56 – 2.49 (m, 2H), 1.94 (ddd, *J* = 13.5, 11.5, 6.8 Hz, 1H). ^{13}C NMR (151 MHz, CD_2Cl_2) δ 171.6, 160.6, 136.2, 130.3, 127.9, 117.9, 113.9, 101.9, 96.3, 83.4, 82.4, 76.5, 73.1, 68.2, 67.1, 55.9, 55.7, 52.5, 34.1. IR (film) $\tilde{\nu}$ 3515, 2956, 2932, 2901, 2854, 1711, 1616, 1519, 1441, 1365, 1245, 1118, 1070, 1046, 994, 834 cm^{-1}. HRMS (ESI) *m*/*z* calcd. for $C_{21}H_{28}O_9Na$ [M+Na]$^+$: 447.16255, found: 447.16267.

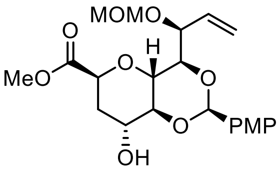

Figure S7. Characteristic NOE's

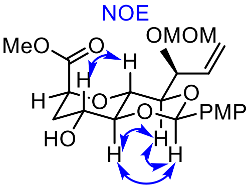

B1/B2 Sub-Fragment Coupling and Substructure Verification

Aldehyde 58. DIBAL-H (1.0 M in hexane, 3.2 mL, 3.20 mmol) was slowly added at –78°C (bath temperature) to a solution of ester **54** (1.55 g, 2.16 mmol) in CH_2Cl_2 (20 mL). After stirring at -78 °C for 1 h, the reaction was quenched with sat. aq. Rochelle salt solution (30 mL). The resulting mixture was vigorously stirred at room temperature for 1 h before the aqueous phase was extracted with CH_2Cl_2 (3 x 20 mL). The combined organic layers were dried over $MgSO_4$ and concentrated under reduced pressure to give the title aldehyde as a colorless oil that was used in the next step without further purification.

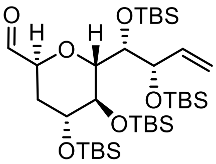

Compound 60. A solution of N-nitrosamide **47** (1.16 g, 1.80 mmol) in toluene (18.0 mL) and MeOH (1.8 mL) was stirred at 0 °C in the dark for 5 min before aq. KOH (40% *w/w*, 2.52 g, 18.00 mmol) was added and the resulting mixture was stirred at 0 °C in the dark for 1 h. $MgSO_4$ (3.06 g) was introduced and the suspension stirred at 0 °C for 5 min before a solution of the crude aldehyde **58** (2.16 mmol) in toluene (18.0 mL) was added dropwise. Stirring was continued at room temperature for 12 h. All solid materials were removed by filtration, the filtrate was concentrated under reduced pressure and the residue purified by flash chromatography (silica; hexane/EtOAc, 25:1 to 10:1) to give the title compound as a colorless oil (1.46 g, 65%).

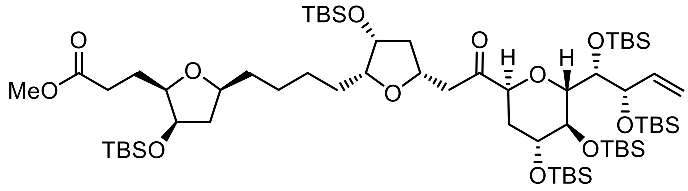

$[\alpha]_D^{20}$ = –27.3° (*c* = 0.94, $CHCl_3$). ^{1}H NMR (400 MHz, $CDCl_3$) δ 6.10 (ddd, *J* = 17.6, 10.6, 7.2 Hz, 1H), 5.23 – 5.18 (m, 1H), 5.18 – 5.14 (m, 1H), 4.36 (dd, *J* = 10.0, 2.2 Hz, 1H), 4.30 (dd, *J* = 12.3, 2.3 Hz, 1H), 4.26 – 4.15 (m, 3H), 4.13 (dd, *J* = 7.2, 2.2 Hz, 1H), 3.91 – 3.86 (m, 1H), 3.76 – 3.69 (m, 2H), 3.65 (s, 3H), 3.64 – 3.59 (m, 2H), 3.53 (td, *J* = 6.6, 3.9 Hz, 1H), 3.17 (dd, *J* = 18.8, 4.8 Hz, 1H), 2.97 (dd, *J* = 18.8, 8.4 Hz, 1H), 2.52 – 2.41 (m, 2H), 2.37 (ddd, *J* = 13.5, 7.7, 6.0 Hz, 1H), 2.22 (dt, *J* = 13.4, 6.8 Hz, 1H), 1.93 – 1.85 (m, 3H), 1.70 – 1.65 (m, 2H), 1.62 – 1.54 (m, 2H), 1.52 – 1.44 (m, 3H), 1.43 – 1.36 (m, 2H), 1.33 – 1.27 (m, 2H), 0.92 (s, 9H), 0.89 (s, 9H), 0.88 (s, 9H), 0.87 (s, 9H), 0.86 (s, 9H), 0.84 (s, 9H), 0.12 (s, 3H), 0.11 (s, 3H), 0.10 (s, 3H), 0.09 (s, 3H), 0.05 – 0.04 (m, 9H), 0.03 (s, 6H), 0.02 (s, 6H), 0.00 (s, 3H). ^{13}C NMR (101 MHz, $CDCl_3$) δ 212.2, 174.4, 138.2, 116.9, 83.3, 81.4, 79.8, 78.3, 77.7, 73.4, 73.2, 73.0, 72.7, 72.5, 70.4, 67.4, 51.6, 46.2, 42.2, 41.8, 36.7, 31.0, 30.6, 29.7, 26.7, 26.7, 26.4, 26.1 (two peaks), 26.0, 25.9 (two peaks), 25.4, 18.4, 18.3, 18.3, 18.2 (two peaks), 18.0, –3.0, –3.6, –3.8, –3.9, –4.2, –4.4 (2 x), –4.7, –4.9 (3 x), –5.0. IR (film) $\tilde{\nu}$ 2953, 2929, 2896, 2857, 1743, 1713, 1472, 1463, 1254, 1077, 1005, 875, 835, 775 cm^{-1}. HRMS (ESI) *m/z* calcd. for $C_{63}H_{128}O_{12}Si_6Na$ $[M+Na]^+$: 1267.79136, found: 1267.79114.

Compounds 34*S*-61 and 34*R*-61. L-Selectride (1.0 M in THF, 1.0 mL, 1.00 mmol) was added dropwise at –78 °C (bath temperature) to a solution of ketone **60** (0.86 g, 0.69 mmol) in THF (15.0 mL). After stirring at this temperature for 40 min, the reaction was quenched with sat. aq. $NaHCO_3$ solution (15 mL) and the mixture stirred at 0 °C before H_2O_2 (35% *w/w*, 3.0 mL) was added. Stirring was continued at room temperature for 1 h. The mixture was extracted with *tert*-butyl methyl ether (3 x 20 mL), the combined organic layers were washed with sat. aq. $Na_2S_2O_3$ solution (20 mL) and brine (20 mL), dried over Na_2SO_4, and concentrated under reduced pressure. The residue was purified by flash chromatography (silica; hexane/EtOAc = 15:1 to 6:1) to give the major diastereomer as a pale-yellow oil (559.3 mg, 65%) and a second fraction consisting of the minor diastereomer as a pale-yellow oil (195.7 mg, 23%).

Analytical and spectral data of the major **34*S*-61** *isomer*: $[\alpha]_D^{20}$ = –27.0° (c = 1.35, $CHCl_3$). ^1H NMR (400 MHz, $CDCl_3$) δ 6.03 (ddd, J = 17.0, 10.5, 6.2 Hz, 1H), 5.21 (dt, J = 17.4, 1.6 Hz, 1H), 5.15 (dt, J = 10.5, 1.6 Hz, 1H), 4.34 (dd, J = 10.1, 1.9 Hz, 1H), 4.30 – 4.21 (m, 2H), 4.18 (ddd, J = 6.7, 4.2, 2.7 Hz, 1H), 4.05 – 3.96 (m, 1H), 3.86 (q, J = 3.3 Hz, 1H), 3.77 (d, J = 3.2 Hz, 1H), 3.73 (t, J = 6.5 Hz, 1H), 3.68 – 3.60 (m, 7H), 3.54 – 3.46 (m, 1H), 3.09 (br, 1H), 2.52 – 2.39 (m, 2H), 2.39 – 2.31 (m, 1H), 2.26 – 2.17 (m, 1H), 1.93 – 1.83 (m, 3H), 1.80 – 1.73 (m, 1H), 1.66 – 1.45 (m, 7H), 1.43 – 1.36 (m, 3H), 1.34 – 1.27 (m, 2H), 0.90 (s, 9H), 0.89 (s, 9H), 0.89 (s, 9H), 0.88 (s, 9H), 0.88 (s, 9H), 0.86 (s, 9H), 0.12 (s, 3H), 0.10 (s, 3H), 0.08 (s, 3H), 0.07 (s, 3H), 0.07 (s, 3H), 0.07 (s, 3H), 0.05 – 0.04 (m, 6H), 0.04 (s, 3H), 0.04 – 0.03 (m, 6H), 0.03 (s, 3H). ^{13}C NMR (101 MHz, $CDCl_3$) δ 174.4, 138.7, 115.9, 83.3, 81.4, 79.2, 77.7, 76.8, 75.1, 73.4 (two peaks), 73.0, 72.2, 70.2, 69.5, 67.4, 51.6, 43.1, 41.8, 39.2, 36.7, 31.0, 29.6, 29.5, 26.7 (two peaks), 26.4, 26.3, 26.2, 26.0, 25.9 (two peaks), 25.4, 18.6, 18.5, 18.3, 18.2, 18.2, 18.1, –2.7, –3.6, –3.6, –3.7, –4.3, –4.4 (two peaks), –4.6, –4.8, –4.9 (3 x). IR (film) $\tilde{\nu}$ 3516, 2953, 2929, 2896, 2857, 1743, 1472, 1463, 1361, 1254, 1076, 1005, 875, 835, 775 cm^{-1}. HRMS (ESI) m/z calcd. for $C_{63}H_{130}O_{12}Si_6Na$ $[M+Na]^+$: 1269.80701, found: 1269.80823.

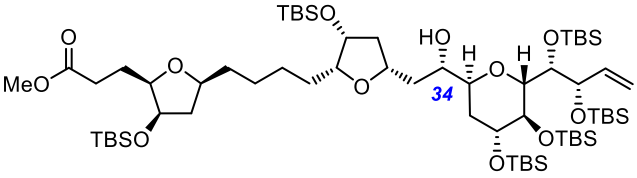

Analytical and spectral data of the minor **34*R*-61** *isomer*: $[\alpha]_D^{20}$ = –19.2° (c = 0.83, $CHCl_3$). ^1H NMR (400 MHz, $CDCl_3$) δ 6.01 (ddd, J = 17.4, 10.5, 7.0 Hz, 1H), 5.15 (dt, J = 17.4, 1.6 Hz, 1H), 5.09 (dt, J = 10.4, 1.3 Hz, 1H), 4.37 (dd, J = 10.0, 1.4 Hz, 1H), 4.28 (dd, J = 7.0, 1.4 Hz, 1H), 4.24 (ddd, J = 6.5, 4.8, 3.9 Hz, 1H), 4.19 (ddd, J = 6.6, 4.2, 2.8 Hz, 1H), 4.03 – 3.94 (m, 1H), 3.90 (q, J = 3.3 Hz, 1H), 3.79 (d, J = 3.3 Hz, 1H), 3.77 – 3.60 (m, 8H), 3.56 (ddd, J = 7.6, 5.5, 4.1 Hz, 1H), 3.26 (br, 1H), 2.52 – 2.37 (m, 2H), 2.33 (ddd, J = 13.7, 7.6, 6.3 Hz, 1H), 2.26 – 2.18 (m, 1H), 1.97 (ddd, J = 13.7, 11.3, 2.6 Hz, 1H), 1.92 – 1.85 (m, 2H), 1.78 (t, J = 6.3 Hz, 2H), 1.70 – 1.63 (m, 2H), 1.57 – 1.44 (m, 5H), 1.44 – 1.37 (m, 2H), 1.33 – 1.27 (m, 2H), 0.90 (s, 9H), 0.89 (s, 9H), 0.88 (s, 9H), 0.88 – 0.88 (m, 18H), 0.87 (s, 9H), 0.11 (s, 3H), 0.09 – 0.07 (m, 9H), 0.06 – 0.05 (m, 6H), 0.05 – 0.04 (m, 6H), 0.04 – 0.03 (m, 6H), 0.03 (s, 3H), 0.02 (s, 3H). ^{13}C NMR (101 MHz, $CDCl_3$) δ 174.4, 140.3, 115.5, 83.8, 81.5, 78.8, 77.7, 77.2, 75.8, 73.7, 73.4, 73.4, 73.0, 70.4, 68.5, 67.4, 51.6, 42.4, 41.8, 38.6, 36.7, 31.0, 29.6, 28.0, 26.8, 26.7, 26.4, 26.2, 26.1, 26.0, 26.0, 25.9, 25.4, 18.6, 18.5, 18.3, 18.2 (two peaks), 18.1, –2.5, –3.4, –3.6, –3.7, –4.3, –4.4 (2 x), –4.5, –4.7, –4.8, –4.9 (2 x). IR (film) $\tilde{\nu}$ 3505, 2952, 2928, 2896, 2856, 1743, 1472, 1462, 1361, 1253, 1076, 1005, 884, 834, 774 cm^{-1}. HRMS (ESI) m/z calcd. for $C_{63}H_{130}O_{12}Si_6Na$ $[M+Na]^+$: 1269.80701, found: 1269.80806.

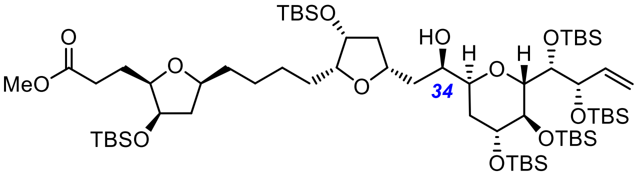

Mosher Ester Analysis of the Major Isomer. Reaction of compound **34*S*-61** and (*S*)-(+)-MTPA-Cl furnished the (*R*)-(+)-MTPA ester as a yellow oil (2.22 mg, 63%). $[\alpha]_D^{20}$ = + 1.4° (*c* = 0.22, $CHCl_3$). ^1H NMR (600 MHz, [D$_4$]-MeOH) δ 7.56 – 7.52 (m, 2H), 7.47 – 7.41 (m, 3H), 6.04 (ddd, *J* = 17.4, 10.3, 7.5 Hz, 1H), 5.34 (dt, *J* = 9.8, 3.5 Hz, 1H), 5.29 (ddd, *J* = 17.3, 1.9, 1.2 Hz, 1H), 5.11 (ddd, *J* = 10.4, 1.9, 1.1 Hz, 1H), 4.53 (dq, *J* = 7.5, 1.1 Hz, 1H), 4.44 (dd, *J* = 10.1, 1.1 Hz, 1H), 4.30 (ddd, *J* = 6.2, 4.4, 3.2 Hz, 1H), 4.24 (ddd, *J* = 6.0, 3.8, 2.0 Hz, 1H), 4.10 – 4.04 (m, 1H), 3.96 – 3.92 (m, 1H), 3.89 – 3.85 (m, 1H), 3.82 – 3.70 (m, 3H), 3.66 – 3.61 (m, 4H), 3.53 (ddd, *J* = 7.8, 5.3, 3.9 Hz, 1H), 3.48 (s, 3H), 2.49 – 2.37 (m, 2H), 2.35 – 2.26 (m, 2H), 2.10 (ddd, *J* = 13.2, 9.5, 4.0 Hz, 1H), 2.05 – 1.99 (m, 2H), 1.90 – 1.85 (m, 2H), 1.72 – 1.64 (m, 2H), 1.63 – 1.58 (m, 1H), 1.56 – 1.49 (m, 4H), 1.47 – 1.42 (m, 1H), 1.39 – 1.34 (m, 3H), 0.93 (s, 9H), 0.92 (s, 9H), 0.91 (s, 18H), 0.90 (s, 9H), 0.84 (s, 9H), 0.16 (s, 3H), 0.15 (s, 3H), 0.12 (s, 3H), 0.11 (s, 3H), 0.10 (s, 3H), 0.08 (s, 3H), 0.08 – 0.07 (m, 12H), 0.06 (s, 3H), 0.03 (s, 3H). ^{13}C NMR (151 MHz, [D$_4$]-MeOH) δ 175.8, 167.7, 142.6, 133.0, 131.0, 129.6, 129.3, 115.9, 85.0, 83.0, 79.7, 79.1, 76.9, 75.6, 75.4, 74.7, 74.5, 74.4, 71.5, 68.4, 66.8, 55.9, 52.0, 43.5, 42.7, 37.7, 37.4, 31.8, 30.8, 29.4, 27.9, 27.7, 26.9, 26.8, 26.7, 26.6, 26.4, 26.4, 26.3, 19.7, 19.3, 19.0, 18.9, 18.8, 17.9, –1.5, –2.6, –3.3, –3.7, –3.9, –4.1, –4.2 (2 x), –4.3, –4.7, –4.8, –5.0 (***Note**: the ^{13}C NMR signals of the CF_3 group and the adjacent quaternary carbon have not been detected*). IR (film) $\tilde{\nu}$ 2954, 2928, 2856, 1744, 1470, 1256, 1081, 836, 775 cm^{-1}. HRMS (ESI) *m/z* calcd. for $C_{73}H_{137}F_3O_{14}Si_6Na$ [M+Na]$^+$: 1485.84682, found: 1485.84858.

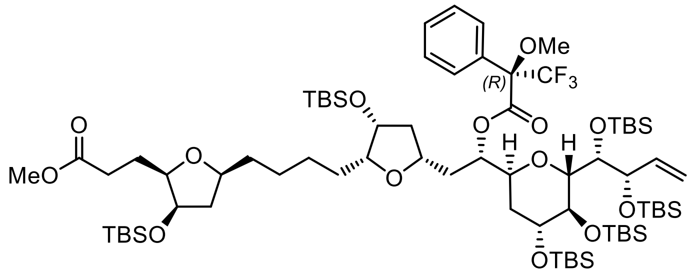

Reaction of compound **34*S*-61** and (*R*)-(–)-MTPA-Cl furnished the (*S*)-(–)-MTPA ester as a yellow oil (1.94 mg, 55%). $[\alpha]_D^{20}$ = –17.5° (*c* = 0.20, $CHCl_3$). ^1H NMR (600 MHz, [D$_4$]-MeOH) δ 7.56 – 7.53 (m, 2H), 7.45 – 7.39 (m, 3H), 6.03 (ddd, *J* = 17.5, 10.4, 7.4 Hz, 1H), 5.33 (dt, *J* = 10.6, 3.2 Hz, 1H), 5.25 (dt, *J* = 17.3, 1.5 Hz, 1H), 5.10 (ddd, *J* = 10.4, 1.8, 1.1 Hz, 1H), 4.52 – 4.49 (m, 1H), 4.42 (dd, *J* = 10.0, 1.1 Hz, 1H), 4.31 (ddd, *J* = 6.2, 4.5, 3.2 Hz, 1H), 4.12 (ddd, *J* = 5.9, 3.7, 1.9 Hz, 1H), 4.04 – 4.00 (m, 1H), 4.00 – 3.98 (m, 1H), 3.91 (dt, *J* = 3.2, 1.1 Hz, 1H), 3.85 – 3.80 (m, 1H), 3.77 (d, *J* = 10.3 Hz, 1H), 3.66 – 3.63 (m, 4H), 3.60 (s, 3H), 3.40 (ddd, *J* = 7.7, 5.3, 3.6 Hz, 1H), 3.38 – 3.33 (m, 1H), 2.50 – 2.36 (m, 2H), 2.31 (ddd, *J* = 13.0, 7.6, 6.3 Hz, 1H), 2.11 – 2.00 (m, 3H), 1.94 – 1.86 (m, 3H), 1.71 – 1.62 (m, 2H), 1.59 – 1.45 (m, 6H), 1.41 – 1.36 (m, 3H), 0.93 (s, 9H), 0.93 (s, 9H), 0.91 (s, 9H), 0.91 (s, 9H), 0.90 (s, 9H), 0.90 (s, 9H), 0.17 (s, 3H), 0.13 (s, 3H), 0.12 (s, 3H), 0.12 (s, 3H), 0.11 (s, 3H), 0.10 (s, 3H), 0.09 (s, 3H), 0.09 (s, 3H), 0.08 (s, 3H), 0.05 (s, 6H), 0.04 (s, 3H). ^{13}C NMR (151 MHz,

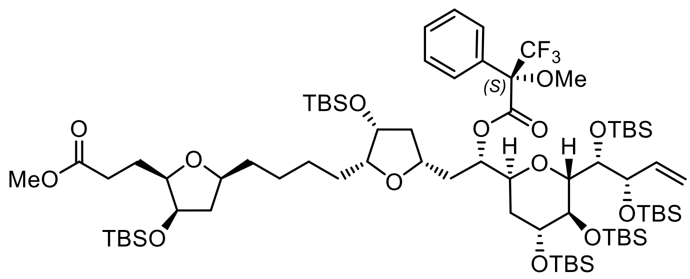

[D4]-MeOH) δ 175.8, 167.4, 142.6, 133.5, 130.8, 129.4, 128.8, 115.9, 84.8, 83.1, 79.7, 79.1, 76.5, 75.4 (two peaks), 74.5, 74.5, 74.0, 71.6, 68.5, 67.6, 56.8, 52.0, 43.2, 42.7, 38.3, 37.7, 31.8, 30.7, 30.4, 27.8, 27.7, 26.9, 26.8, 26.7, 26.6, 26.4, 26.4, 26.4, 19.7, 19.3, 19.0, 19.0, 18.9, 18.9, −1.5, −2.7, −3.4, −3.6, −4.0, −4.1 (2 x), −4.2, −4.3, −4.7, −4.9, −5.0 (***Note***: *the* ^{13}C *NMR signals of the* CF_3 *group and the adjacent quaternary carbon have not been detected*). IR (film) ṽ 2952, 2929, 2856, 1747, 1472, 1256, 1082, 835, 775 cm^{-1}. HRMS (ESI) *m/z* calcd. for $C_{73}H_{137}F_3O_{14}Si_6Na$ $[M+Na]^+$: 1485.84682, found: 1485.84769.

Table S10. Determination of absolute configuration of the stereogenic center at C34 of the major diastereomer by Mosher ester analysis.

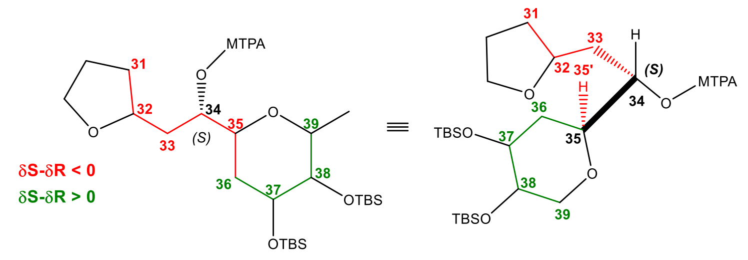

No.	$\delta_{H\ NMR}$ (*S*-ester) (ppm)	$\delta_{H\ NMR}$ (*R*-ester) (ppm)	$\Delta\delta$ (δ_S - δ_R, ppm)
31a	1.38	1.53	−0.15
31b	2.04	2.32	−0.28
32	3.35	3.78	−0.43
33a	1.91	2.01	−0.10
33b	2.02	2.10	−0.08
34	5.33	5.34	−0.01
35'	4.02	4.07	−0.05[a]
36ax	2.08	2.01	0.07
36eq	1.46	1.37	0.09
37	3.99	3.94	0.05
38	3.91	3.87	0.04
39	3.77	3.74	0.03

[a] unexpected negative value, probably due to the steric repulsion that shifts the conformer equilibrium.

Mosher Ester Analysis of the Minor Isomer. Reaction of compound **34*R*-61** and (*S*)-(+)-MTPA-Cl furnished the (*R*)-(+)-MTPA ester as a yellow oil (2.60 mg, 74%). $[\alpha]_D^{20}$ = + 13.5° (*c* = 0.26, $CHCl_3$). ^1H NMR (600 MHz, [D4]-MeOH) δ 7.64 – 7.61 (m, 2H), 7.40 – 7.36 (m, 3H), 6.05 (ddd, *J* = 17.5, 10.5, 7.2 Hz, 1H), 5.51 (ddd, *J* = 8.4, 5.4, 2.5 Hz, 1H),

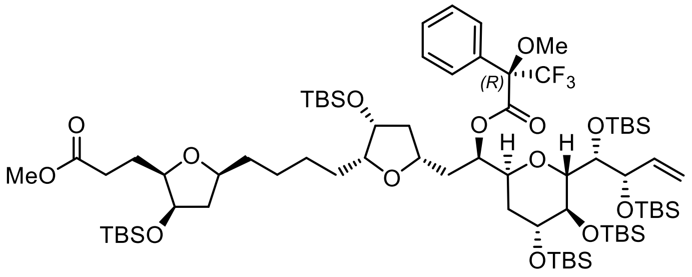

5.23 (dt, J = 17.3, 1.5 Hz, 1H), 5.10 (ddd, J = 10.5, 1.9, 1.1 Hz, 1H), 4.46 (dd, J = 10.2, 1.3 Hz, 1H), 4.39 (dd, J = 7.2, 1.2 Hz, 1H), 4.31 (ddd, J = 6.4, 4.4, 3.2 Hz, 1H), 4.26 (ddd, J = 5.9, 4.0, 2.6 Hz, 1H), 4.07 – 4.03 (m, 1H), 4.00 – 3.97 (m, 1H), 3.87 (d, J = 3.1 Hz, 1H), 3.83 – 3.78 (m, 1H), 3.78 – 3.73 (m, 2H), 3.66 – 3.61 (m, 7H), 3.55 (td, J = 6.8, 4.2 Hz, 1H), 2.49 – 2.38 (m, 2H), 2.36 – 2.27 (m, 2H), 2.17 – 2.12 (m, 1H), 2.07 – 2.02 (m, 1H), 1.91 – 1.86 (m, 2H), 1.66 – 1.49 (m, 7H), 1.47 – 1.41 (m, 3H), 1.33 – 1.30 (m, 2H), 0.93 (s, 18H), 0.92 (s, 9H), 0.92 (s, 9H), 0.91 (s, 9H), 0.83 (s, 9H), 0.17 (s, 3H), 0.15 (s, 3H), 0.13 (s, 3H), 0.12 (s, 3H), 0.10 (s, 3H), 0.09 – 0.09 (m, 9H), 0.09 (s, 3H), 0.08 (s, 3H), 0.08 (s, 3H), 0.08 (s, 3H). ^{13}C NMR (151 MHz, [D$_4$]-MeOH) δ 175.8, 167.4, 141.8, 134.2, 130.4, 129.4, 128.8, 116.3, 84.9, 83.1, 80.7, 79.0, 77.2, 76.9, 75.5, 75.2, 74.5, 74.1, 71.6, 68.9, 67.7, 56.9, 52.0, 43.1, 42.7, 38.4, 37.6, 31.8, 30.8, 28.3, 27.6, 27.5, 26.8, 26.8, 26.8, 26.5, 26.4, 26.4 (two peaks), 19.5, 19.3, 19.0, 19.0, 19.0, 18.9, –1.8, –2.8, –3.3, –3.5, –4.1 (2 x), –4.2, –4.3, –4.4, –4.6, –4.9, –5.0 (***Note**: the ^{13}C NMR signals of the CF_3 group and the adjacent quaternary carbon have not been detected*). IR (film) ṽ 2954, 2926, 2855, 1747, 1463, 1256, 1081, 836, 776 cm^{-1}. HRMS (ESI) *m/z* calcd. for $C_{73}H_{137}F_3O_{14}Si_6Na$ [M+Na]$^+$: 1485.84682, found: 1485.84925.

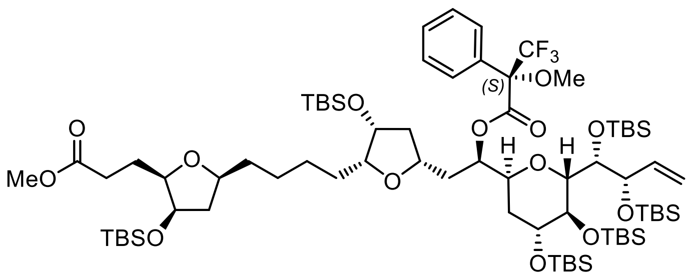

Reaction of compound **34*R*-61** and (*R*)-(–)-MTPA-Cl, the reaction gave (*S*)-(–)-MTPA ester as a yellow oil (2.76 mg, 78%). $[\alpha]_D^{20}$ = –5.0° (*c* = 0.20, CHCl$_3$). ^{1}H NMR (600 MHz, [D$_4$]-MeOH) δ 7.59 – 7.55 (m, 2H), 7.44 – 7.38 (m, 3H), 5.96 (ddd, J = 17.4, 10.5, 6.9 Hz, 1H), 5.45 (ddd, J = 8.7, 4.8, 3.1 Hz, 1H), 5.16 (dt, J = 17.5, 1.7 Hz, 1H), 5.08 (dt, J = 10.5, 1.3 Hz, 1H), 4.39 (dd, J = 10.2, 1.4 Hz, 1H), 4.33 (dd, J = 6.9, 1.3 Hz, 1H), 4.32 – 4.27 (m, 2H), 4.02 – 3.98 (m, 1H), 3.96 – 3.93 (m, 1H), 3.93 – 3.87 (m, 1H), 3.84 (d, J = 3.2 Hz, 1H), 3.82 – 3.77 (m, 1H), 3.67 – 3.59 (m, 6H), 3.51 (s, 3H), 2.49 – 2.33 (m, 3H), 2.30 (ddd, J = 13.0, 7.6, 6.2 Hz, 1H), 2.17 (ddd, J = 13.8, 8.8, 7.5 Hz, 1H), 2.12 – 2.06 (m, 1H), 1.91 – 1.83 (m, 3H), 1.69 – 1.49 (m, 6H), 1.48 – 1.42 (m, 3H), 1.35 – 1.32 (m, 2H), 0.93 (s, 9H), 0.92 – 0.91 (m, 36H), 0.83 (s, 9H), 0.15 (s, 3H), 0.12 (s, 3H), 0.11 (s, 3H), 0.11 (s, 3H), 0.10 (s, 3H), 0.10 (s, 3H), 0.09 (s, 3H), 0.09 (s, 6H), 0.08 (s, 3H), 0.05 (s, 3H), 0.03 (s, 3H). ^{13}C NMR (151 MHz, [D$_4$]-MeOH) δ 175.8, 167.5, 141.6, 133.5, 130.8, 129.5, 129.1, 116.2, 85.0, 83.1, 80.3, 79.1, 77.8, 76.8, 75.8, 74.9, 74.5, 74.2, 71.6, 68.6, 68.0, 56.4, 52.0, 43.2, 42.7, 38.5, 37.6, 31.8, 30.9, 29.2, 27.6, 27.5, 26.9, 26.8, 26.8, 26.5, 26.4, 26.4, 26.3, 19.5, 19.3, 19.0, 19.0, 19.0, 18.8, –1.8, –2.9, –3.3, –3.4, –4.1, –4.3 (4 x), –4.6, –4.9, –5.0 (***Note**: the ^{13}C NMR signals of the CF_3 group and the adjacent quaternary carbon have not been detected*). IR (film) ṽ 2954, 2927, 2856, 1744, 1468, 1255, 1082, 835, 775 cm^{-1}. HRMS (ESI) *m/z* calcd. for $C_{73}H_{137}F_3O_{14}Si_6Na$ [M+Na]$^+$: 1485.84682, found: 1485.84805.

Table S11. Determination of absolute configuration of the stereogenic center at C34 of minor diastereomer via Mosher ester analysis.[4]

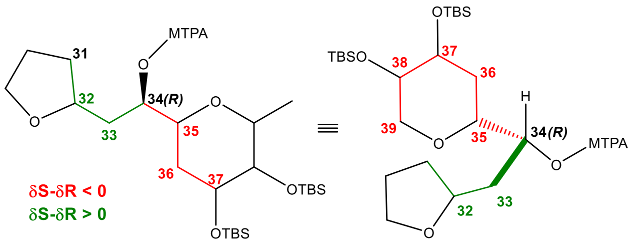

No.	$\delta_{H\,NMR}$ (*S*-ester) (ppm)	$\delta_{H\,NMR}$ (*R*-ester) (ppm)	$\Delta\delta$ (δ_S -δ_R, ppm)
31a	1.58	1.56	0.02
31b	2.35	2.33	0.02
32	3.90	3.75	0.15
33a	1.86	1.62	0.24
33b	2.17	2.05	0.12
34	5.45	5.51	–0.06
35'	4.00	4.05	–0.05
36ax	2.09	2.14	–0.05
36eq	1.45	1.46	–0.01
37	3.95	3.99	–0.04
38	3.84	3.87	–0.03
39	3.66	3.75	–0.09

Compound 34*S*-62. DIBAL-H (1.0 M in hexane, 30.0 µL, 30.0 µmol) was slowly added at –78°C (bath temperature) to a solution of methyl ester **34*S*-61** (10.0 mg, 8.0 µmol) in CH_2Cl_2 (1 mL). After stirring at –78 °C for 1 h and at room temperature for an additional 1 h, the reaction was quenched with sat. Rochelle salt solution (5 mL). The resulting mixture was vigorously stirred at room temperature for 1 h and the aqueous phase extracted with CH_2Cl_2 (3 x 5 mL). The combined organic layers were dried over Na_2SO_4 and concentrated under reduced pressure to give the corresponding primary alcohol that was used in the next step without further purification.

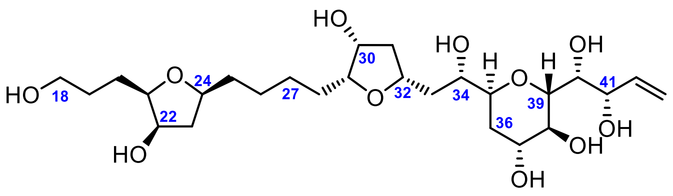

TBAF (1.0 M in THF, 60.0 µL, 60.0 µmol) was added dropwise to a solution of the crude product (8.0 µmol) in THF (1.0 mL). The mixture was stirred at room temperature for 2 h before $CaCO_3$ (30 mg), DOWEX 50WX8-400 (90 mg), and MeOH (1.0 mL) were added. The suspension was stirred at room temperature for 1 h. All insoluble materials were filtered off through a pad of Celite, thoroughly rinsing the filter cake with MeOH. The combined filtrates were concentrated under reduced pressure and the residue was purified by flash chromatography (silica; CH_2Cl_2/MeOH =

4:1 to 2:1) to give the title compound as a colorless oil (3.3 mg, 77% over two steps). $[\alpha]_D^{20}$ = –18.3° (*c* = 0.47, MeOH). ^{1}H NMR (600 MHz, [D$_4$]-MeOH) δ 6.00 (ddd, *J* = 17.3, 10.5, 5.9 Hz, 1H, *H*-42), 5.32 (dt, *J* = 17.3, 1.7 Hz, 1H, *H*-43trans), 5.16 (ddd, *J* = 10.6, 1.9, 1.5 Hz, 1H, *H*-43cis), 4.37 (ddt, *J* = 5.9, 2.3, 1.5 Hz, 1H, *H*-41), 4.22 – 4.14 (m, 2H, *H*-22 & *H*-30), 4.07 – 4.01 (m, 1H, *H*-32), 3.99 – 3.92 (m, 2H, *H*-34 & *H*-40), 3.85 (ddd, *J* = 8.3, 6.3, 4.2 Hz, 1H, *H*-37), 3.77 – 3.72 (m, 1H, *H*-24), 3.68 (dd, *J* = 7.8, 6.2 Hz, 1H, *H*-39'), 3.66 – 3.63 (m, 1H, *H*-35'), 3.62 – 3.60 (m, 1H, *H*-38), 3.58 (t, *J* = 6.0 Hz, 2H, *H*-18), 3.55 – 3.52 (m, 2H, *H*-29 & *H*-21), 2.46 – 2.33 (m, 2H, *H*-31b & *H*-23b), 2.04 (ddd, *J* = 13.8, 5.8, 4.3 Hz, 1H, *H*-36eq), 1.80 (ddd, *J* = 14.0, 9.1, 2.4 Hz, 1H, *H*-33b), 1.74 – 1.59 (m, 9H, *H*-25b, *H*-20b, *H*-19b, *H*-20a, *H*-28, *H*-19a, *H*-33a & *H*-36ax), 1.57 – 1.51 (m, 2H, *H*-25a & *H*-31a), 1.51 – 1.45 (m, 3H, *H*-23a, *H*-27b & *H*-26b), 1.43 – 1.36 (m, 2H, *H*-27a & *H*-26a). ^{13}C NMR (151 MHz, [D$_4$]-MeOH) δ 139.8 (*C*-42), 116.0 (*C*-43), 84.6 (*C*-29), 84.4 (*C*-21), 79.0 (*C*-24), 75.5 (*C*-32), 75.4 (*C*-39), 75.2 (*C*-40), 75.2 (*C*-35), 73.3 (*C*-30), 73.2 (*C*-22), 73.2 (*C*-38), 73.1 (*C*-41), 69.9 (*C*-37), 69.5 (*C*-34), 63.1 (*C*-18), 42.7 (*C*-31), 42.4 (*C*-23), 41.3 (*C*-33), 37.3 (*C*-25), 32.6 (*C*-36), 30.5 (*C*-19), 30.1 (*C*-28), 27.6 (*C*-26), 27.6 (*C*-27), 26.5 (*C*-20). IR (film) $\tilde{\nu}$ 3362, 2926, 2858, 1648, 1436, 1059, 923, 843, 723 cm^{-1}. HRMS (ESI) *m/z* calcd. for $C_{26}H_{46}O_{11}Na$ [M+Na]$^+$: 557.29323, found: 557.29346.

Table S12. Analysis of the NMR data of compound **34*S*-62**

Atom	*δ* (ppm)	*J* (Hz)	COSY	HSQC	HMBC	NOESY
**18 C**	63.12			18	19a, 19b, 20a, 20b	
**H2**	3.58		19a, 19b	18	19, 20	
**19 C**	30.52			19a, 19b	18, 20a, 20b, 21	
**Ha**	1.63		18, 20a, 20b	19	18, 20, 21	
**Hb**	1.66		18, 20a, 20b	19	18, 20, 21	
**20 C**	26.50			20a, 20b	18, 19a, 19b, 21	
**Ha**	1.66		19a, 19b, 20b, 21	20	18, 19, 21, 22	
**Hb**	1.69		19a, 19b, 20a, 21	20	18, 19, 21, 22	
**21 C**	84.36			21	19a, 19b, 20a, 20b, 22, 23a, 23b	
**H**	3.53	4.00(22)	20a, 20b, 22	21	19, 20, 22	22, 23b, 24
**22 C**	73.22			22	20a, 20b, 21, 23a, 23b, 24	
**H**	4.18	4.00(21), 2.69(23a), 6.80(23b)	21, 23a, 23b	22	21, 24	21, 23b, 24
**23 C**	42.35			23a, 23b	25a, 25b	
**Ha**	1.48	13.48(23b), 2.69(22)	22, 23b, 24	23	21, 22, 24, 25	
**Hb**	2.38	13.48(23a), 6.80(22)	22, 23a, 24	23	21, 22, 24, 25	21, 22, 24
**24 C**	79.01			24	22, 23a, 23b, 25a, 25b, 26a, 26b	
**H**	3.74		23a, 23b, 25a, 25b	24	22, 26	21, 22, 23b, 25a, 25b, 26a
**25 C**	37.34			25a, 25b	23a, 23b, 27a, 27b	
**Ha**	1.56		24, 25b, 26a, 26b	25	23, 24, 26, 27	24
**Hb**	1.71		24, 25a, 26a, 26b	25	23, 24, 26, 27	24

**26 C**	27.63			26a, 26b	24, 25a, 25b, 27a, 27b, 28	
**Ha**	1.37		25a, 25b, 26b, 27a, 27b	26	24, 27, 28	24
**Hb**	1.46		25a, 25b, 26a, 27a, 27b	26	24, 27, 28	
**27 C**	27.59			27a, 27b	25a, 25b, 26a, 26b, 28, 29	
**Ha**	1.41		26a, 26b, 27b, 28	27	25, 26, 28, 29	29
**Hb**	1.48		26a, 26b, 27a, 28	27	25, 26, 28, 29	29
**28 C**	30.07			28	26a, 26b, 27a, 27b, 29	
**H2**	1.64		27a, 27b, 29	28	26, 27, 29	29
**29 C**	84.59			29	27a, 27b, 28, 30, 31a, 31b	
**H**	3.54	3.80(30)	28, 30	29	27, 28, 30	27a, 27b, 28, 30, 31b, 32
**30 C**	73.29			30	29, 31a, 31b, 32	
**H**	4.17	2.40(31a), 6.60(31b), 3.80(29)	29, 31a, 31b	30	29, 32	29, 31b
**31 C**	42.70			31a, 31b	33a, 33b	
**Ha**	1.54	13.60(31b), 6.90(32), 2.40(30)	30, 31b, 32	31	29, 30, 32, 33	33a, 33b
**Hb**	2.41	7.90(32), 13.60(31a), 6.60(30)	30, 31a, 32	31	29, 30, 32, 33	29, 30, 32
**32 C**	75.45			32	30, 31a, 31b, 33a, 33b, 34	
**H**	4.04	3.40(33a), 9.10(33b), 7.90(31b), 6.90(31a)	31a, 31b, 33a, 33b	32	30, 34	29, 30, 31b, 33a, 34
**33 C**	41.27			33a, 33b	31a, 31b, 34, 35'	
**Ha**	1.62	3.40(32), 10.00(34), 14.10(33b)	32, 33b, 34	33	31, 32	31a, 32
**Hb**	1.80	9.10(32), 14.10(33a), 2.40(34)	32, 33a, 34	33	31, 32	31a, 34, 35', 36eq
**34 C**	69.49			34	32, 35', 36ax, 36eq	
**H**	3.96	10.00(33a), 2.40(33b), 7.30(35')	33a, 33b, 35'	34	32, 33, 35, 36	32, 33b, 35', 36eq, 37, 39'
**35 C**	75.16			35'	34, 36ax, 36eq, 37, 39'	
**35' H**	3.64	7.30(34), 4.80(36ax), 5.70(36eq)	34, 36ax, 36eq	35	33, 34, 36, 37, 39	33b, 34, 36ax, 36eq, 41
**36 C**	32.56			36ax, 36eq	34, 35', 37, 38	
**Hax**	1.62	13.70(36eq), 8.20(37), 4.80(35')	35', 36eq, 37	36	34, 35, 37, 38, 41	35', 38
**Heq**	2.04	4.30(37), 13.70(36ax), 5.70(35')	35', 36ax, 37	36	34, 35, 37, 38, 41	33b, 34, 35', 37
**37 C**	69.94			37	35', 36ax, 36eq, 38, 39'	
**H**	3.85	6.30(38), 4.30(36eq), 8.20(36ax)	36ax, 36eq, 38	37	35, 36, 38, 39	34, 36eq, 39'
**38 C**	73.18			38	36ax, 36eq, 37, 39', 40	
**H**	3.61	6.30(39'), 6.30(37)	37, 39'	38	36, 37, 39, 40	36ax, 40, 41
**39 C**	75.43			39'	35', 37, 38, 40, 41	
**39' H**	3.68	7.80(40), 6.30(38)	38, 40	39	35, 37, 38, 40, 41	34, 37, 40, 41, 42
**40 C**	75.22			40	38, 39', 42	
**H**	3.94	2.30(41), 7.80(39')	39', 41	40	38, 39, 42	38, 39', 41, 42, 43trans
**41 C**	73.05			41	36ax, 36eq, 39', 42, 43cis, 43trans	

**H**	4.37	5.90(42), 2.30(40)	40, 42, 43cis, 43trans	41	39, 42, 43	35', 38, 39', 40
**42 C**	139.80			42	40, 41, 43cis, 43trans	
**H**	6.00	10.60(43cis), 17.30(43trans), 5.90(41)	41, 43cis, 43trans	42	40, 41	39', 40
**43 C**	115.96			43cis, 43trans	41	
**Hcis**	5.16	10.60(42)	41, 42, 43trans	43	41, 42	
**Htrans**	5.32	17.30(42)	41, 42, 43cis	43	41, 42	40

Table S13. Comparison of NMR data of authentic benthol A and compound **34*S*-62**

Atom	δ (ppm, ^{1}H-NMR in CD_3OD)		Δδ	δ (ppm, ^{13}C-NMR in CD_3OD)		Δδ
	Authentic benthol A	**34*S*-62**		Authentic benthol A	**34*S*-62**	
**18**	4.05	3.58	0.47	73.8	63.1	10.7
**19a**	1.63	1.63	0.00	35.1	30.5	4.6
**19b**	1.63	1.66	−0.03			
**20a**	1.64	1.66	−0.02	26.2	26.5	−0.3
**20b**	1.71	1.69	0.02			
**21**	3.53	3.53	0.00	84.5	84.4	0.1
**22**	4.18	4.18	0.00	73.2	73.2	0.0
**23a**	1.49	1.48	0.01	42.4	42.4	0.0
**23b**	2.38	2.38	0.00			
**24**	3.75	3.74	0.01	79.1	79.0	0.1
**25a**	1.56	1.56	0.00	37.4	37.3	0.1
**25b**	1.71	1.71	0.00			
**26a**	1.38	1.37	0.01	27.7	27.6	0.1
**26b**	1.48	1.46	0.02			
**27a**	1.40	1.41	−0.01	27.6	27.6	0.0
**27b**	1.48	1.48	0.00			
**28a**	1.64	1.64	0.00	30.2	30.1	0.1
**28b**	1.64	1.64	0.00			
**29**	3.56	3.54	0.02	84.7	84.6	0.1
**30**	4.18	4.17	0.01	73.3	73.3	0.0
**31a**	1.55	1.54	0.01	42.7	42.7	0.0
**31b**	2.42	2.41	0.01			
**32**	4.08	4.04	0.04	75.4	75.5	−0.1
**33a**	1.52	1.62	−0.10	41.7	41.3	0.4
**33b**	1.82	1.80	0.02			
**34**	4.18	3.96	0.22	67.1	69.5	−2.4
**35**	3.62	3.64	−0.02	77.8	75.2	2.6
**36ax**	1.72	1.62	0.10	34.2	32.6	1.6
**36eq**	2.03	2.04	−0.01			
**37**	3.76	3.85	−0.09	71.1	69.9	1.2
**38**	3.49	3.61	−0.12	72.8	73.2	−0.4
**39**	3.69	3.68	0.01	73.2	75.4	−2.2
**40**	3.62	3.94	−0.32	73.1	75.2	−2.1
**41**	3.63	4.37	−0.74	72.5	73.1	−0.6

Compound 34*R*-62. Prepared analogously from **34*R*-61** as a colorless oil (4.0 mg, 93% over two steps).

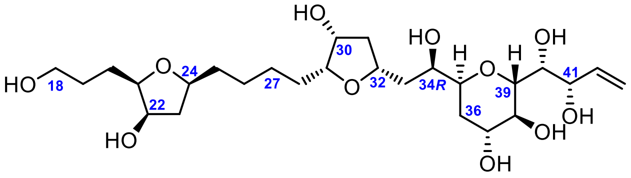

$[\alpha]_D^{20}$ = + 1.5° (*c* = 0.40, MeOH). ^{1}H NMR (600 MHz, [D4]-MeOH) δ 5.99 (ddd, *J* = 17.3, 10.5, 5.8 Hz, 1H), 5.32 (dt, *J* = 17.3, 1.7 Hz, 1H), 5.17 (dt, *J* = 10.5, 1.7 Hz, 1H), 4.33 (ddt, *J* = 5.8, 2.3, 1.5 Hz, 1H), 4.21 – 4.16 (m, 2H), 4.06 – 4.00 (m, 1H), 3.88 – 3.79 (m, 3H), 3.77 – 3.72 (m, 1H), 3.63 – 3.52 (m, 7H), 2.43 (ddd, *J* = 13.5, 8.0, 6.6 Hz, 1H), 2.38 (ddd, *J* = 13.5, 7.7, 6.9 Hz, 1H), 2.24 (dt, *J* = 13.5, 4.5 Hz, 1H), 2.03 (ddd, *J* = 13.8, 7.5, 2.5 Hz, 1H), 1.76 – 1.54 (m, 11H), 1.51 – 1.46 (m, 3H), 1.44 – 1.36 (m, 2H). ^{13}C NMR (151 MHz, [D4]-MeOH) δ140.3, 115.9, 84.8, 84.4, 79.0, 77.1, 76.3, 75.4, 75.1, 73.9, 73.2, 73.2, 72.5, 69.7, 69.6, 63.1, 42.4, 42.0, 41.0, 37.3, 31.8, 30.5, 29.9, 27.6, 27.6, 26.5. IR (film) $\tilde{\nu}$ 3353, 2926, 2857, 1650, 1441, 1062, 928, 844, 726 cm^{-1}. HRMS (ESI) *m/z* calcd. for $C_{26}H_{46}O_{11}Na$ $[M+Na]^+$: 557.29323, found: 557.29371.

Table S14. Analysis of the NMR data of **34*R*-62**

Atom	*δ* (ppm)	*J* (Hz)	COSY	HSQC	HMBC	NOESY
**18 C**	63.13			18	19, 20	
**H2**	3.59		19	18	19, 20	19, 20
**19 C**	30.53			19	18, 20, 21	
**H2**	1.66		18, 20	19	18, 20, 21	18, 21
**20 C**	26.5			20	18, 19	
**H2**	1.68		19, 21	20	18, 19, 21, 22	18, 21, 22
**21 C**	84.37			21	19, 20, 22, 23a, 23b	
**H**	3.54		20, 22	21	19, 22	19, 20, 22, 23b, 24
**22 C**	73.24			22	20, 21, 23a, 23b, 24	
**H**	4.19	6.80(23b), 2.70(23a)	21, 23a, 23b	22	21, 24	20, 21, 23b, 24
**23 C**	42.37			23a, 23b		
**Ha**	1.49	2.70(22), 13.40(23b), 7.40(24)	22, 23b, 24	23	21, 22, 24, 25	
**Hb**	2.38	6.80(22), 7.70(24), 13.40(23a)	22, 23a, 24	23	21, 22, 24, 25	21, 22
**24 C**	79			24	22, 23a, 23b, 25a, 25b, 26a, 26b	
**H**	3.75	7.70(23b), 7.40(23a)	23a, 23b, 25a, 25b	24	22, 26	21, 22, 25a, 25b, 26a
**25 C**	37.3			25a, 25b	23a, 23b, 26a, 26b, 27a, 27b	
**Ha**	1.57		24, 25b, 26a, 26b	25	24, 26, 27	24
**Hb**	1.71		24, 25a, 26a, 26b	25	24, 26	24
**26 C**	27.63			26a, 26b	24, 25a, 25b, 27a, 27b	
**Ha**	1.39		25a, 25b, 26b, 27a, 27b	26	24, 25, 27, 28	24
**Hb**	1.49		25a, 25b, 26a, 27a, 27b	26	24, 25, 28	
**27 C**	27.55			27a, 27b	25a, 26a, 28, 29	

**Ha**	1.42		26a, 26b, 27b, 28	27	25, 26, 28, 29	29
**Hb**	1.48		26a, 26b, 27a, 28	27	25, 26	29
**28 C**	29.91			28	26a, 26b, 27a, 29	
**H2**	1.66		27a, 27b, 29	28	27, 29, 30	29
**29 C**	84.82			29	27a, 28, 30, 31a, 31b	
**H**	3.56		28, 30	29	27, 28, 30	27a, 27b, 28, 30, 32
**30 C**	73.2			30	28, 29, 31a, 31b, 32	
**H**	4.18	2.30(31a), 6.63(31b)	29, 31a, 31b	30	29, 32	29, 31b, 32
**31 C**	41.99			31a, 31b	33a, 33b	
**Ha**	1.63	6.80(32), 13.50(31b), 2.30(30)	30, 31b, 32	31	29, 30, 32, 33	33b
**Hb**	2.43	13.50(31a), 8.00(32), 6.63(30)	30, 31a, 32	31	29, 30, 32, 33	30, 32
**32 C**	77.1			32	30, 31a, 31b, 33a, 33b, 34	
**H**	4.03	6.80(31a), 8.00(31b), 5.90(33a), 7.50(33b)	31a, 31b, 33a, 33b	32	30	29, 30, 31b, 33a, 33b, 34, 35'
**33 C**	40.95			33a, 33b	31a, 31b, 35'	
**Ha**	1.73	5.90(32), 13.80(33b), 9.80(34)	32, 33b, 34	33	31, 32, 35	32, 35'
**Hb**	2.03	7.50(32), 13.80(33a), 2.50(34)	32, 33a, 34	33	31, 32, 35	31a, 32, 34, 35', 41
**34 C**	69.64			34	35', 36a	
**H**	3.84	9.80(33a), 2.50(33b), 8.50(35')	33a, 33b, 35'	34	32, 35, 36	32, 33b
**35 C**	75.38			35'	33a, 33b, 34, 36a, 36b	
**35' H**	3.61	8.50(34), 4.90(36a), 4.50(36b)	34, 36a, 36b	35	33, 34, 36, 37, 39	32, 33a, 33b, 36a, 36b, 40, 41
**36 C**	31.75			36a, 36b	34, 35'	
**Hax**	1.61	4.90(35'), 13.50(36b)	35', 36b, 37	36	34, 35, 37, 38	35', 37
**Heq**	2.24	4.50(35'), 13.50(36a)	35', 36a, 37	36	35, 37, 38	35', 37
**37 C**	69.74			37	35', 36a, 36b, 38, 39'	
**H**	3.86		36a, 36b, 38	37	38, 39	36a, 36b
**38 C**	73.88			38	36a, 36b, 37, 39', 40	
**H**	3.58		37, 39'	38	37, 39, 40	
**39 C**	75.13			39'	35', 37, 38, 40	
**39' H**	3.58		38, 40	39	37, 38, 40, 41	
**40 C**	76.3			40	38, 39', 41, 42	
**H**	3.81		39', 41	40	38, 39, 42	35', 41
**41 C**	72.5			41	39', 42, 43cis, 43trans	
**H**	4.33	1.70(43cis), 1.70(43trans), 5.80(42)	40, 42, 43cis, 43trans	41	40, 42, 43	33b, 35', 40, 43trans
**42 C**	140.25			42	40, 41, 43trans	
**H**	5.99	5.80(41), 10.50(43cis), 17.30(43trans)	41, 43cis, 43trans	42	40, 41	
**43 C**	115.91			43cis, 43trans	41	
**Hcis**	5.17	1.70(41), 1.70(43trans), 10.50(42)	41, 42, 43trans	43	41	
**Htrans**	5.32	1.70(41), 1.70(43cis), 17.30(42)	41, 42, 43cis	43	41, 42	41

Table S15. Comparison of the ^{13}C NMR data of authentic benthol A and synthetic **34*R*-62**

Atom	δ (ppm, ^{13}C NMR in CD_3OD)		Δδ
	Authentic benthol A	**34*R*-62**	
**18**	73.8	63.1	10.7
**19**	35.1	30.5	4.6
**20**	26.2	26.5	−0.3
**21**	84.5	84.4	0.1
**22**	73.2	73.2	0.0
**23**	42.4	42.4	0.0
**24**	79.1	79.0	0.1
**25**	37.4	37.3	0.1
**26**	27.7	27.6	0.1
**27**	27.6	27.6	0.0
**28**	30.2	29.9	0.3
**29**	84.7	84.8	−0.1
**30**	73.3	73.2	0.1
**31**	42.7	42.0	0.7
**32**	75.4	77.1	−1.7
**33**	41.7	41.0	0.7
**34**	67.1	69.6	−2.5
**35**	77.8	75.4	2.4
**36**	34.2	31.8	2.4
**37**	71.1	69.7	1.4
**38**	72.8	73.9	−1.1
**39**	73.2	75.1	−1.9
**40**	73.1	76.3	−3.2
**41**	72.5	72.5	0.0

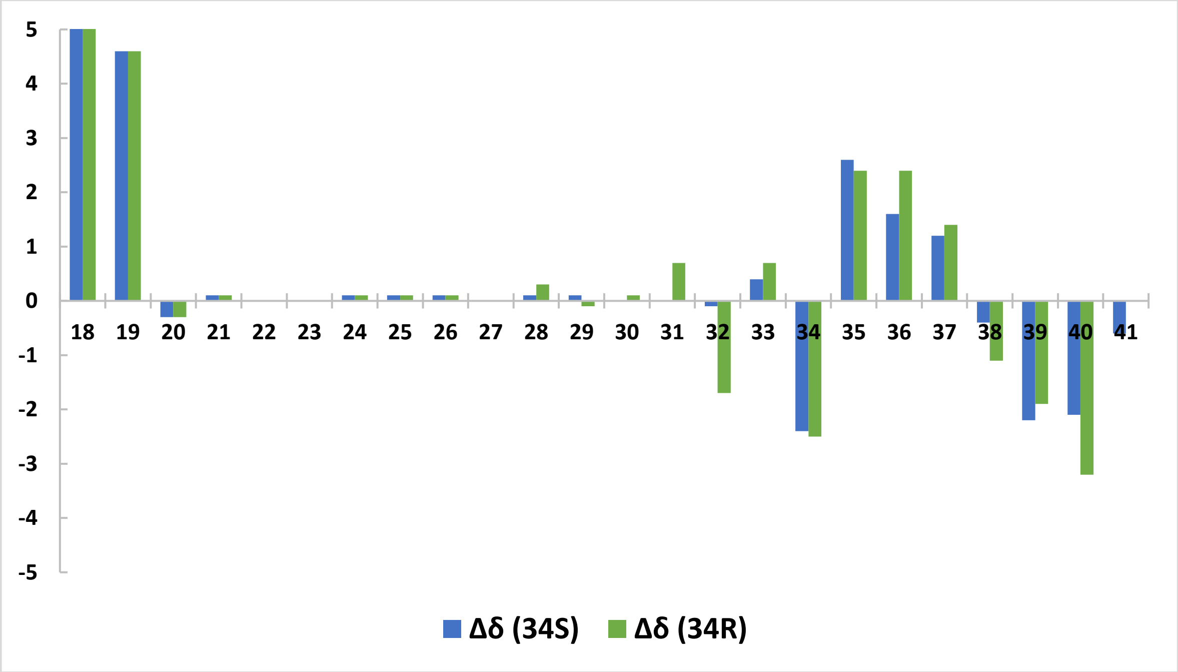

Figure S8. Comparison of the ^{13}C NMR shifts of **34*S*-62** and **34*R*-62** with those of authentic benthol A reported in the literature ($\Delta\delta_C$).

Revised Fragment B2 and Second Substructure Verification

Compound 63. Dess-Martin periodinane (30.0 mg, 71.0 µmol) was slowly added at 0 °C to a solution of alcohol **52** (19.0 mg, 35.5 µmol) and $NaHCO_3$ (12.0 mg, 142.0 µmol) in CH_2Cl_2 (2 mL). The mixture was stirred at room temperature for 2 h before sat. aq. $Na_2S_2O_3$ solution (2 mL) and H_2O (2 mL) were added to quench the reaction. The organic layer was separated and the aqueous phase extracted with CH_2Cl_2 (3 x 10 mL). The combined organic layers were washed with sat. aq. $NaHCO_3$ solution (3 x 10 mL), dried over Na_2SO_4 and concentrated under reduced pressure. The residue was purified by flash chromatography (silica; hexane/EtOAc, 10:1 to 5:1) to give the title compound as a colorless oil (16.2 mg, 86%). $[\alpha]_D^{20}$ = + 79.2° (*c* = 1.05, $CHCl_3$). ^{1}H NMR (400 MHz, $CDCl_3$) δ 5.82 (ddd, *J* = 17.2, 10.3, 6.8 Hz, 1H), 5.43 (d, *J* = 17.2 Hz, 1H), 5.34 (d, *J* = 10.3 Hz, 1H), 5.04 (dd, *J* = 12.0, 2.5 Hz, 1H), 4.81 – 4.69 (m, 3H), 4.64 (d, *J* = 6.7 Hz, 1H), 4.11 (d, *J* = 3.8 Hz, 1H), 3.85 (td, *J* = 3.9, 2.2 Hz, 1H), 3.77 (s, 3H), 3.34 (s, 3H), 2.16 (ddd, *J* = 13.9, 12.0, 2.2 Hz, 1H), 1.76 (ddd, *J* = 13.4, 3.4, 3.4 Hz, 1H), 0.90 (s, 9H), 0.86 (s, 9H), 0.11 (s, 3H), 0.09 (s, 3H), 0.06 (s, 3H), 0.04 (s, 3H). ^{13}C NMR (101 MHz, $CDCl_3$) δ 204.7, 172.5, 132.9, 119.8, 94.8, 81.8, 81.0, 69.0, 67.8, 67.4, 56.2, 52.3, 30.5, 26.0, 25.9, 18.3, 18.1, –4.1, –4.5, –4.8, –5.0. IR (film) $\tilde{\nu}$ 2953, 2930, 2895, 2858, 1767, 1735, 1668, 1472, 1439, 1362, 1257, 1153, 1093, 1035, 922, 834, 777 cm^{-1}. HRMS (ESI) *m*/*z* calcd. for $C_{25}H_{48}O_8Si_2Na$ $[M+Na]^+$: 555.27799, found: 555.27832.

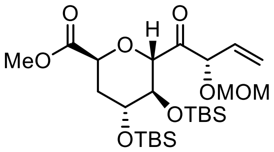

Compound 64 (via Ketone Reduction). $NaBH_4$ (5.8 mg, 153.3 µmol) was added at 0 °C to a solution of ketone **63** (27.0 mg, 50.7 µmol) in EtOH (1.0 mL). The mixture was stirred at this temperature for 2 h before the reaction was quenched with sat. aq. NH_4Cl solution (5 mL). The mixture was extracted with EtOAc (3 x 10 mL), and the combined organic layers were washed with brine (10 mL), dried over Na_2SO_4 and concentrated under reduced pressure. The residue was purified by flash chromatography (silica; hexane/EtOAc = 5:1) to give compound **52** (13.1 mg, 48%) and a second fraction consisting of compound **64** as a colorless oil (5.1 mg, 19%). The analytical and spectral data are compiled below.

Compound 64 (via *anti*-oxy-Allylation). *sec*-BuLi (1.4 M in cyclohexane, 0.72 mL, 1.00 mmol) was added dropwise at –78 °C to a solution of methoxymethyl allyl ether **51** (0.13 g, 1.20 mmol) in THF (20 mL). The resulting yellow solution was stirred at –78 °C for 30 min before thoroughly dried $ZnCl_2$ (0.28 g, 2.00 mmol) was added. The solution was then stirred at 0 °C, leading to the disappearance of the yellow color in a few seconds and the formation of a white suspension. After stirring at 0 °C for 2 h, the mixture was cooled to –78 °C before a solution of the crude aldehyde (1.00 mmol) derived from alcohol **50** (for its preparation, see compound **52**) in THF (5 mL) was added dropwise. The mixture was stirred at –78 °C for 2 h and at 0 °C for additional 30 min before the reaction was quenched with brine (20 mL). The mixture was extracted with *tert*-butyl methyl ether (3 x 20 mL). The combined organic layers were dried over Na_2SO_4 and

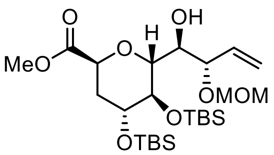

concentrated under reduced pressure, and the residue was purified by flash chromatography (silica; hexane/EtOAc = 10:1 to 5:1) to give the title compound as a colorless oil (415.2 mg, 78% over two steps, dr > 20:1). $[\alpha]_D^{20}$ = + 22.0° (c = 1.69, $CHCl_3$). ^{1}H NMR (400 MHz, $CDCl_3$) δ 5.91 (ddd, J = 17.6, 10.4, 7.4 Hz, 1H), 5.38 – 5.28 (m, 2H), 4.73 (d, J = 6.6 Hz, 1H), 4.69 (d, J = 6.5 Hz, 1H), 4.54 (dd, J = 5.1, 5.1 Hz, 1H), 4.15 – 4.08 (m, 1H), 3.92 – 3.85 (m, 1H), 3.83 (dd, J = 7.1, 2.4 Hz, 1H), 3.76 – 3.68 (m, 4H), 3.58 (dd, J = 7.0, 7.0 Hz, 1H), 3.38 (s, 3H), 2.39 (d, J = 5.7 Hz, 1H), 2.33 (ddd, J = 13.6, 4.9, 3.7 Hz, 1H), 1.81 (ddd, J = 13.4, 9.1, 5.4 Hz, 1H), 0.91 (s, 9H), 0.88 (s, 9H), 0.11 (s, 3H), 0.10 (s, 3H), 0.10 (s, 3H), 0.09 (s, 3H). ^{13}C NMR (101 MHz, $CDCl_3$) δ 172.0, 136.2, 118.9, 95.2, 78.7, 76.5, 71.6, 71.4, 70.6, 70.4, 55.9, 52.1, 34.1, 26.3, 26.1, 18.4, 18.2, –3.2, –3.3, –4.5, –4.7. IR (film) $\tilde{\nu}$ 3525, 2954, 2930, 2893, 2857, 1753, 1472, 1438, 1253, 1099, 1034, 1006, 927, 835, 778 cm^{-1}. HRMS (ESI) m/z calcd. for $C_{25}H_{50}O_8Si_2Na$ $[M+Na]^+$: 557.29365, found: 557.29402.

Compound S21. Prepared by following the procedure described for compound **53**; pale-yellow oil

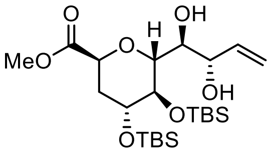

(397.2 mg, 81%). $[\alpha]_D^{20}$ = + 21.5° (c = 0.94, $CHCl_3$). ^{1}H NMR (400 MHz, $CDCl_3$) δ 5.98 (ddd, J = 17.4, 10.6, 5.4 Hz, 1H), 5.41 (ddd, J = 17.2, 1.7, 1.7 Hz, 1H), 5.25 (ddd, J = 10.6, 1.6, 1.6 Hz, 1H), 4.51 (dd, J = 6.1, 4.2 Hz, 1H), 4.35 – 4.22 (m, 1H), 3.77 (s, 3H), 3.74 – 3.69 (m, 1H), 3.68 – 3.58 (m, 3H), 3.34 (br, 1H), 2.61 – 2.20 (m, 2H), 1.92 – 1.81 (m, 1H), 0.90 (s, 9H), 0.86 (s, 9H), 0.11 (s, 3H), 0.10 (s, 3H), 0.09 (s, 3H), 0.09 (s, 3H). ^{13}C NMR (101 MHz, $CDCl_3$) δ 172.6, 138.0, 116.4, 76.4, 75.4, 72.0, 71.6, 71.3, 70.6, 52.5, 33.7, 26.2, 26.1, 18.3, 18.2, –3.1, –3.2, –4.4, –4.8. IR (film) $\tilde{\nu}$ 3522, 2954, 2930, 2894, 2857, 1738, 1472, 1253, 1099, 1005, 928, 835, 778 cm^{-1}. HRMS (ESI) m/z calcd. for $C_{23}H_{46}O_7Si_2Na$ $[M+Na]^+$: 513.26743, found: 513.26808.

Compound 65. DIBAL-H (1.0 M in hexane, 0.20 mL, 0.20 mmol) was slowly added at –78°C to a solution

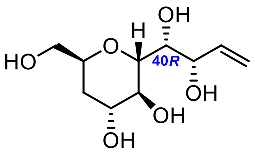

of methyl ester **52** (20.0 mg, 40.8 µmol) in CH_2Cl_2 (1 mL) and the resulting mixture was stirred at this temperature for 1 h and at room temperature for an additional 1 h. The reaction was quenched with sat. Rochelle salt solution (10 mL), the resulting mixture was vigorously stirred at for 1 h before the aqueous phase was extracted with CH_2Cl_2 (3 x 10 mL). The combined organic layers were dried over Na_2SO_4 and concentrated under reduced pressure to give the corresponding alcohol that was used in the next step without further purification.

TBAF (1.0 M in THF, 0.12 mL, 0.12 mmol) was added dropwise to a solution of this crude material in THF (1.0 mL) and the resulting mixture was stirred at room temperature for 2 h. $CaCO_3$ (90 mg), DOWEX 50WX8-400 (270 mg), and MeOH (1.0 mL) were added and the suspension was stirred at room temperature for 1 h before all insoluble materials were filtered off through a pad of Celite, thoroughly rinsing the filter cake with MeOH. The combined filtrates were concentrated under reduced pressure and the residue was purified by flash chromatography (silica; CH_2Cl_2/MeOH, 10:1 to 5:1) to give the

title compound as a colorless oil (6.3 mg, 66%). $[\alpha]_D^{20}$ = + 20.6° (*c* = 0.63, MeOH). ^{1}H NMR (600 MHz, [D$_4$]-MeOH) δ 6.01 (ddd, *J* = 17.2, 10.5, 5.9 Hz, 1H), 5.33 (ddd, *J* = 17.2, 1.7, 1.7 Hz, 1H), 5.17 (ddd, *J* = 10.5, 1.9, 1.4 Hz, 1H), 4.35 (ddt, *J* = 5.7, 2.8, 1.5 Hz, 1H), 4.00 – 3.94 (m, 1H), 3.91 (dd, *J* = 6.9, 2.6 Hz, 1H), 3.80 – 3.73 (m, 2H), 3.63 (dd, *J* = 6.9, 6.9 Hz, 1H), 3.58 (dd, *J* = 6.8, 6.8 Hz, 1H), 3.50 (dd, *J* = 11.7, 4.6 Hz, 1H), 1.92 (ddd, *J* = 13.6, 4.7, 4.7 Hz, 1H), 1.63 (ddd, *J* = 13.6, 9.0, 5.2 Hz, 1H). ^{13}C NMR (151 MHz, [D$_4$]-MeOH) δ 139.9, 116.0, 75.6, 75.3, 73.4, 73.1, 72.9, 69.9, 63.2, 32.8. IR (film) $\tilde{\nu}$ 3356, 2927, 2858, 1647, 1420, 1268, 1103, 1045, 931, 845 cm^{-1}. HRMS (ESI) *m*/*z* calcd. for $C_{10}H_{18}O_6Na$ [M+Na]$^+$: 257.09956, found: 257.09939.

Table S16. Analysis of the NMR data of the 40*R*-configured compound **65**

Atom	*δ* (ppm)	*J* (Hz)	COSY	HSQC	HMBC	NOESY
**34 C**	63.18			34a, 34b	35, 36ax, 36eq	
**Ha**	3.5	4.64(35), 11.71(34b)	34b, 35	34	36	35, 36eq
**Hb**	3.76	8.25(35), 11.71(34a)	34a, 35	34	35, 36	36eq, 39'
**35 C**	72.9			35	34b, 36ax, 36eq, 37, 39'	
**H**	3.97	4.66(36eq), 5.19(36ax), 4.64(34a), 8.25(34b)	34a, 34b, 36ax, 36eq	35	34, 36, 37, 39	34a, 36ax, 36eq, 40, 41
**36 C**	32.83			36ax, 36eq	34a, 34b, 35, 37, 38	
**Hax**	1.63	5.19(35), 13.55(36eq), 8.90(37)	35, 36eq, 37	36	34, 35, 37, 38	35, 38
**Heq**	1.92	4.66(35), 4.66(37), 13.55(36ax)	35, 36ax, 37	36	34, 35, 37, 38	34a, 34b, 35, 37
**37 C**	69.91			37	35, 36ax, 36eq, 38, 39'	
**H**	3.78	6.83(38), 4.66(36eq), 8.90(36ax)	36ax, 36eq, 38	37	35, 36, 38, 39	36eq, 38, 39'
**38 C**	73.37			38	36ax, 36eq, 37, 39', 40	
**H**	3.58	6.83(39'), 6.83(37)	37, 39'	38	36, 37, 39, 40	36ax, 37, 40, 41, 42
**39 C**	75.25			39'	35, 37, 38, 40	
**39' H**	3.63	6.89(40), 6.83(38)	38, 40	39	35, 37, 38, 40, 41	34b, 37, 41, 42
**40 C**	75.56			40	38, 39', 42	
**H**	3.91	2.55(41), 6.89(39')	39', 41	40	38, 39, 42	35, 38, 41, 42
**41 C**	73.1			41	39', 42, 43cis, 43trans	
**H**	4.35	5.90(42), 1.40(43cis), 1.60(43trans), 2.55(40)	40, 42	41	42, 43	35, 38, 39', 40, 42, 43trans
**42 C**	139.86			42	40, 41, 43trans	
**H**	6.01	10.50(43cis), 17.30(43trans), 5.90(41)	41, 43cis, 43trans	42	40, 41	38, 39', 40, 41, 43cis
**43 C**	115.97			43cis, 43trans	41	
**Hcis**	5.17	10.50(42), 1.80(43trans), 1.40(41)	42, 43trans	43	41	42
**Htrans**	5.33	17.30(42), 1.80(43cis), 1.60(41)	42, 43cis	43	41, 42	41

Compound 66. Prepared analogously starting from compound **64** as colorless oil (2.00 mg, 81%). $[\alpha]_D^{20}$

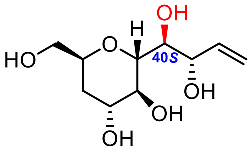

= + 17.5° (*c* = 0.20, MeOH). ^{1}H NMR (600 MHz, [D$_4$]-MeOH) δ 6.05 (ddd, *J* = 17.3, 10.6, 5.8 Hz, 1H), 5.33 (ddd, *J* = 17.3, 2.0, 1.5 Hz, 1H), 5.18 (ddd, *J* = 10.5, 2.0, 1.4 Hz, 1H), 4.19 – 4.13 (m, 1H), 4.07 – 4.01 (m, 1H), 3.88 (dd, *J* = 11.7, 9.3 Hz, 1H), 3.73 – 3.66 (m, 3H), 3.52 – 3.45 (m, 2H), 1.93 (ddd, *J* = 13.2, 5.2, 1.7 Hz, 1H), 1.71 (ddd, *J* = 13.4, 11.4, 6.4 Hz, 1H). ^{13}C NMR (151 MHz, [D$_4$]-MeOH) δ 140.4, 116.0, 74.4, 73.8, 72.8, 72.6, 72.6, 70.9, 61.5, 34.1. IR (film) $\tilde{\nu}$ 3343, 2954, 2924, 2855, 1668, 1413, 1105, 1039, 924 cm^{-1}. HRMS (ESI) *m*/*z* calcd. for $C_{10}H_{18}O_6Na$ [M+Na]$^+$: 257.09956, found: 257.09959.

Table S17. Analysis of the NMR data of the 40*S*-configured compound **66**

Atom	δ (ppm)	J	COSY	HSQC	HMBC	NOESY
**34 C**	61.53			34a, 34b	35, 36ax, 36eq	
**Ha**	3.5	11.70(34b), 5.20(35)	34b, 35	34		35, 36eq, 37
**Hb**	3.88	11.70(34a), 9.20(35)	34a, 35	34	35	37, 39'
**35 C**	74.36			35	34b, 36ax	
**H**	4.04	5.20(34a), 9.20(34b), 6.40(36ax), 1.80(36eq)	34a, 34b, 36ax, 36eq	35	34, 37, 39	34a, 36ax, 36eq
**36 C**	34.09			36ax, 36eq		
**Hax**	1.71	6.40(35), 13.40(36eq), 11.30(37)	35, 36eq, 37	36	34, 35, 37, 38	35, 38
**Heq**	1.93	1.80(35), 13.40(36ax), 5.20(37)	35, 36ax, 37	36	34, 37, 38	34a, 35, 37
**37 C**	70.88			37	35, 36ax, 36eq, 38, 39'	
**H**	3.71	11.30(36ax), 5.20(36eq), 8.60(38)	36ax, 36eq, 38	37	38	34a, 34b, 36eq, 39'
**38 C**	72.61			38	36ax, 36eq, 37, 40	
**H**	3.47	8.60(37), 9.30(39')	37, 39'	38	37, 39, 40	36ax, 40
**39 C**	72.82			39'	35, 38	
**39' H**	3.67	9.30(38), 1.80(40)	38, 40	39	37	34b, 37, 40, 41
**40 C**	72.55			40	38, 41	
**H**	3.69	8.30(41), 1.80(39')	39', 41	40	38, 41, 42	38, 39', 42
**41 C**	73.83			41	40, 42, 43cis, 43trans	
**H**	4.16	8.30(40), 5.80(42), 1.40(43cis), 1.50(43trans)	40, 42, 43cis, 43trans	41	40, 43	39', 42, 43trans
**42 C**	140.44			42	40, 43trans	
**H**	6.05	5.80(41), 10.60(43cis), 17.30(43trans)	41, 43cis, 43trans	42	41	40, 41
**43 C**	115.96			43cis, 43trans	41	
**Hcis**	5.18	1.40(41), 10.60(42), 2.00(43trans)	41, 42, 43trans	43	41	
**Htrans**	5.33	1.50(41), 17.30(42), 2.00(43cis)	41, 42, 43cis	43	41, 42	41

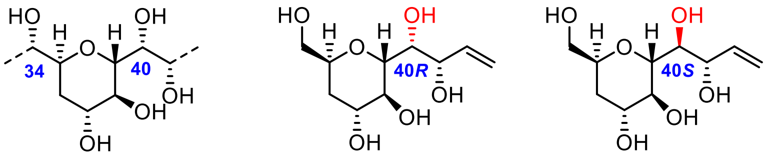

E-ring of nominal Benthol A | **65** 40*R*-isomer | **66** 40*S*-isomer

Table S18. Comparison of the ^{1}H NMR data of ring E of authentic benthol A with those of compounds **65** and **66** differing in the configuration of the C40 stereocenter

Atom	^{1}H NMR [δ (ppm), mult, *J* (Hz)] in CD_3OD		
	Authentic benthol A	**65** (40*R*-isomer)	**66** (40*S*-isomer)
**35**	3.60 m	3.97 m	4.04 m
**36ax**	1.70 m	1.63 ddd (13.6, 9.0, 5.2)	1.71 ddd (13.4, 11.4, 6.4)
**36eq**	2.03 ddd (12.3, 5.0, 1.4)	1.92 ddd (13.6, 4.7, 4.7)	1.93 ddd (13.2, 5.2, 1.7)
**37**	3.76 m	3.78 m	3.71 m
**38**	3.48 dd (9.6, 9.6)	3.58 dd (6.8, 6.8)	3.47 dd (9.3, 8.6)
**39**	3.69 dd (9.6, 1.2)	3.63 dd (6.9, 6.9)	3.67 dd (9.3, 1.8)

Table S19. Comparison of ^{13}C NMR data of authentic benthol A with those of compounds **65** and **66** differing in the configuration of the C40 stereocenter

Atom	δ (ppm, ^{13}C-NMR in CD_3OD)			$\Delta\delta_{40R}$	$\Delta\delta_{40S}$
	Authentic benthol A	**65** (40*R*-isomer)	**66** (40*S*-isomer)		
**34**	67.1	63.2	61.5	3.9	5.6
**35**	77.8	72.9	74.4	4.9	3.4
**36**	34.2	32.8	34.1	1.4	0.1
**37**	71.1	69.9	70.9	1.2	0.2
**38**	72.8	73.4	72.6	–0.6	0.2
**39**	73.2	75.3	72.8	–2.1	0.4
**40**	73.1	75.6	72.6	–2.5	0.5
**41**	72.5	73.1	73.8	–0.6	–1.3

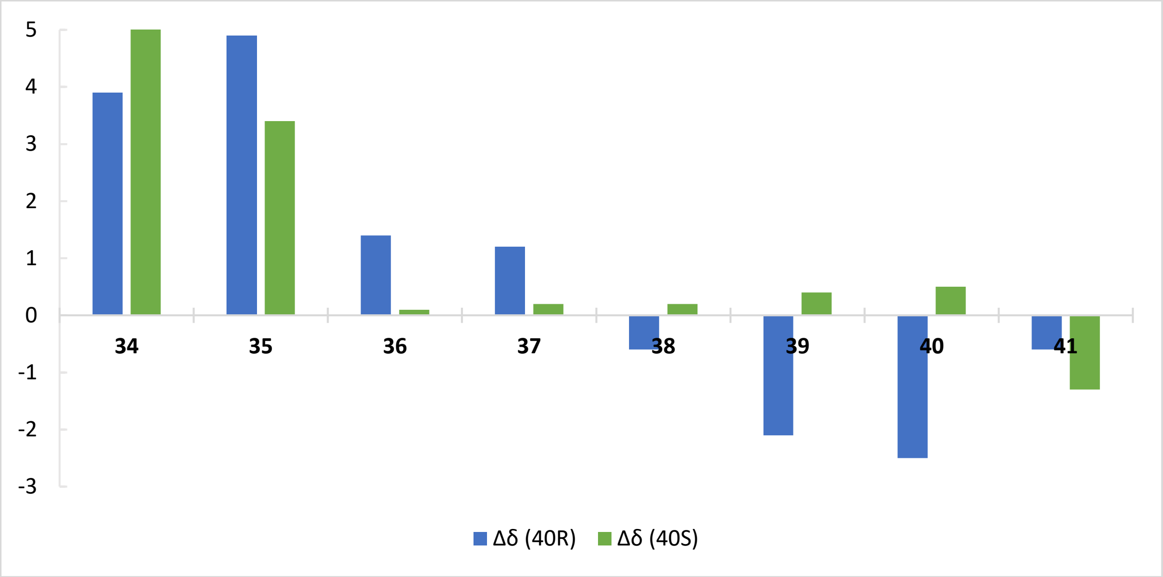

Figure S9. Comparison of the ^{13}C NMR shifts of **65** (40*R*-isomer) and **66** (40*S*-isomer) with those of the E-ring of authentic benthol A reported in the literature ($\Delta\delta_C$).

The Revised Central Fragment B

Compound S22. The TBS-protection of diol **S21** was performed by following the procedure described

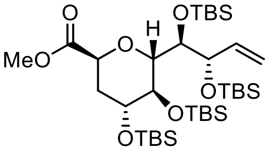

for compound **54**; colorless oil (417.0 mg, 97%). $[\alpha]_D^{20}$ = –7.0° (*c* = 1.17, $CHCl_3$). ^1H NMR (400 MHz, $CDCl_3$) δ 5.98 (ddd, *J* = 17.7, 10.5, 7.2 Hz, 1H), 5.27 – 5.06 (m, 2H), 4.43 (dd, *J* = 9.9, 3.7 Hz, 1H), 4.24 (dd, *J* = 7.0, 2.9 Hz, 1H), 4.21 – 4.16 (m, 1H), 3.88 – 3.83 (m, 1H), 3.78 (dd, *J* = 7.0, 3.7 Hz, 1H), 3.72 (s, 3H), 3.64 – 3.59 (m, 1H), 2.17 (ddd, *J* = 12.8, 9.9, 2.9 Hz, 1H), 1.82 – 1.69 (m, 1H), 0.93 (s, 9H), 0.89 (s, 9H), 0.87 (s, 18H), 0.11 (s, 9H), 0.09 – 0.07 (m, 9H), 0.06 (s, 3H), 0.05 (s, 3H). ^{13}C NMR (101 MHz, $CDCl_3$) δ 173.0, 138.8, 117.1, 78.9, 77.4, 73.8, 70.5, 69.6, 67.3, 52.0, 32.1, 26.4, 26.4, 26.3, 26.0, 18.7, 18.6, 18.4, 18.1, –2.6, –3.5, –3.8, –3.9, –4.0, –4.2, –4.3, –4.5. IR (film) $\tilde{\nu}$ 2953, 2929, 2895, 2857, 1758, 1472, 1251, 1098, 1005, 941, 832, 775 cm^{-1}. HRMS (ESI) *m/z* calcd. for $C_{35}H_{74}O_7Si_4Na$ $[M+Na]^+$: 741.44038, found: 741.44070.

Compound 68. An oven-dried flask was charged with ester **S22** (259.0 g, 0.36 mmol) and CH_2Cl_2 (5 mL).

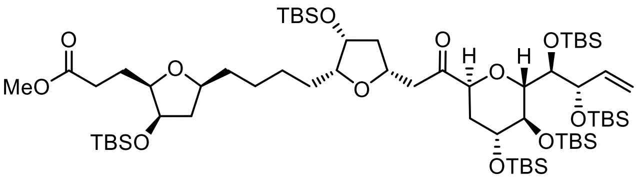

The solution was stirred at –78 °C for 5 min before the slow addition of Dibal-H (1.0 M in toluene, 0.54 mL, 0.54 mmol). After being stirred at –78 °C for another 1 h, the reaction was quenched with sat. Rochelle salt solution (20 mL). The resulting mixture was vigorously stirred at room temperature for 1 h before it was extracted with CH_2Cl_2 (3 x 10 mL). The combined organic layers were dried over $MgSO_4$ and concentrated under reduced pressure to give the crude aldehyde **67** as a pale-yellow oil that was used for the next step without further purification.

A flask was charged with *N*-nitrosoamide **47** (193.5 mg, 0.30 mmol), toluene (3.0 mL) and MeOH (0.3 mL). The resulting solution was stirred at 0 °C in the dark for 5 min before the addition of aqueous KOH (40% *w/w*, 420.8 mg, 3.00 mmol). The mixture was stirred at 0 °C in the dark for 1 h before $MgSO_4$ (0.51 g) was added to the mixture. The suspension was stirred at 0 °C for 5 min before a solution of the crude aldehyde **67** in toluene (3.0 mL) was added dropwise. Stirring was continued at room temperature for 12 h. The solid was removed by filtration, the filtrate was concentrated under reduced pressure, and the residue was purified by flash chromatography (silica; hexane/EtOAc = 25:1 to 10:1) to give target compound as a pale-yellow oil (275.3 mg, 74%). $[\alpha]_D^{20}$ = –17.4° (*c* = 1.90, $CHCl_3$). ^1H NMR (400 MHz, $CDCl_3$) δ 5.97 (ddd, *J* = 17.1, 10.6, 7.6 Hz, 1H), 5.22 – 5.14 (m, 2H), 4.31 – 4.20 (m, 4H), 4.20 – 4.14 (m, 2H), 3.86 – 3.79 (m, 1H), 3.78 – 3.69 (m, 1H), 3.68 – 3.57 (m, 6H), 3.54 (td, *J* = 6.6, 3.9 Hz, 1H), 3.16 (dd, *J* = 17.4, 6.1 Hz, 1H), 2.73 (dd, *J* = 17.5, 7.0 Hz, 1H), 2.52 – 2.32 (m, 3H), 2.22 (ddd, *J* = 12.9, 7.2, 6.5 Hz, 1H), 2.03 (ddd, *J* = 13.4, 10.6, 3.0 Hz, 1H), 1.92 – 1.85 (m, 2H), 1.70 – 1.54 (m, 4H), 1.53 – 1.44 (m, 3H), 1.42 – 1.23 (m, 4H), 0.93 (s, 9H), 0.90 – 0.87 (m, 27H), 0.86 (s, 9H), 0.86 (s, 9H), 0.12 – 0.09 (m, 9H), 0.08 – 0.06 (m, 6H), 0.06 – 0.04 (m, 15H), 0.03 – 0.01 (m, 6H). ^{13}C NMR (101 MHz,

$CDCl_3$) δ 210.7, 174.4, 138.7, 117.5, 83.4, 81.5, 78.9, 77.7, 77.6, 73.4, 73.4, 73.2, 73.1, 73.0, 70.0, 69.0, 51.6, 45.9, 42.2, 41.8, 36.7, 31.0, 30.9, 29.7, 26.7, 26.7, 26.5, 26.5, 26.3, 26.0, 26.0, 25.9, 25.4, 18.7, 18.6, 18.4, 18.2 (two peaks), 18.0, –2.8, –3.2, –3.9, –3.9, –4.0, –4.1, –4.2, –4.3, –4.4, –4.6, –4.9, –4.9. IR (film) $\tilde{\nu}$ 2954, 2930, 2896, 2857, 1743, 1713, 1472, 1463, 1361, 1254, 1074, 1006, 939, 835, 775 cm^{-1}. HRMS (ESI) *m/z* calcd. for $C_{63}H_{128}O_{12}Si_6Na$ $[M+Na]^+$: 1267.79136, found: 1267.79177.

Compound 69. The reduction of ketone **68** with L-Selectride was performed by following the precudrue

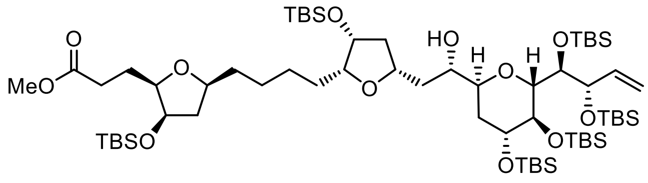

described for the preparation of compound **61**. The diastereomeric ratio (dr = 10.4:1) was determined by ^{1}H NMR of the crude product. Flash chromatography afforded the major diastereomer as a pale-yellow oil (213.4 mg, 77%). $[\alpha]_D^{20}$ = –18.6° (*c* = 0.80, $CHCl_3$). ^{1}H NMR (400 MHz, $CDCl_3$) δ 5.95 (ddd, *J* = 17.5, 10.4, 7.2 Hz, 1H), 5.30 – 5.07 (m, 2H), 4.28 – 4.21 (m, 1H), 4.20 – 4.14 (m, 2H), 4.12 – 4.03 (m, 2H), 3.88 – 3.82 (m, 1H), 3.80 – 3.71 (m, 2H), 3.70 – 3.60 (m, 7H), 3.57 – 3.50 (m, 1H), 2.89 (br, 1H), 2.53 – 2.36 (m, 2H), 2.30 (ddd, *J* = 13.7, 8.0, 6.1 Hz, 1H), 2.26 – 2.17 (m, 1H), 1.94 – 1.84 (m, 2H), 1.81 – 1.45 (m, 10H), 1.44 – 1.36 (m, 2H), 1.34 – 1.24 (m, 2H), 0.90 (s, 9H), 0.89 – 0.87 (m, 36H), 0.87 (s, 9H), 0.12 (s, 3H), 0.10 (s, 3H), 0.09 – 0.06 (m, 15H), 0.06 – 0.03 (m, 12H), 0.03 (s, 3H). ^{13}C NMR (101 MHz, $CDCl_3$) δ 174.4, 139.4, 117.0, 83.4, 81.4, 77.7, 77.5, 76.9, 75.2, 74.8, 73.4, 73.3, 71.5, 71.1, 71.0, 70.5, 51.6, 42.4, 41.8, 39.1, 36.7, 31.0, 29.8, 29.7, 26.8, 26.7, 26.6, 26.4, 26.2, 26.0, 26.0, 25.9, 25.4, 18.7, 18.6, 18.3, 18.2, 18.2, 18.1, –2.6, –3.3, –3.5, –3.7, –3.9, –3.9, –4.2, –4.3, –4.3, –4.4, –4.9, –4.9. IR (film) $\tilde{\nu}$ 3530, 2953, 2929, 2896, 2857, 1743, 1472, 1463, 1361, 1254, 1074, 1005, 939, 834, 775 cm^{-1}. HRMS (ESI) *m/z* calcd. for $C_{63}H_{130}O_{12}Si_6Na$ $[M+Na]^+$: 1269.80701, found: 1269.80732.

Compound 70. Prepared from compound **69** by following the two-step procedure described for the

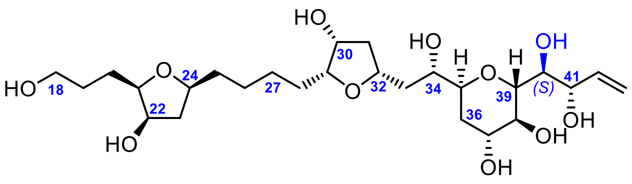

isomeric products **62**; the title compound was purified by flash chromatography (silica 60 cyano, H_2O/MeOH = 9:1). Colorless oil (4.34 mg, quant.). $[\alpha]_D^{20}$ = –22.8° (*c* = 0.43, MeOH). ^{1}H NMR (600 MHz, [D4]-MeOH) δ 6.05 (ddd, *J* = 17.3, 10.6, 5.9 Hz, 1H), 5.33 (ddd, *J* = 17.3, 1.9, 1.5 Hz, 1H), 5.18 (ddd, *J* = 10.5, 2.0, 1.3 Hz, 1H), 4.23 – 4.12 (m, 4H), 4.07 (dddd, *J* = 9.7, 8.0, 6.7, 3.1 Hz, 1H), 3.80 – 3.73 (m, 2H), 3.72 – 3.68 (m, 2H), 3.63 (ddd, *J* = 9.5, 6.2, 2.0 Hz, 1H), 3.61 – 3.58 (m, 2H), 3.58 – 3.53 (m, 2H), 3.50 (dd, *J* = 9.4, 8.4 Hz, 1H), 2.45 – 2.35 (m, 2H), 2.04 (ddd, *J* = 13.5, 5.0, 1.9 Hz, 1H), 1.83 (ddd, *J* = 14.2, 9.3, 2.1 Hz, 1H), 1.76 – 1.61 (m, 8H), 1.59 – 1.46 (m, 6H), 1.46 – 1.37 (m, 2H). ^{13}C NMR (151 MHz, [D4]-MeOH) δ 140.4, 116.1, 84.6, 84.4, 79.0, 77.8, 75.4, 74.2, 73.3, 73.2, 73.1, 72.6, 72.6, 71.0, 67.3, 63.1, 42.7, 42.3, 41.5, 37.3, 34.0, 30.5, 30.1, 27.6, 27.6, 26.5. IR (film) $\tilde{\nu}$ 3360, 2925, 2855,

1646, 1595, 1415, 1063, 924 cm^{-1}. HRMS (ESI) *m/z* calcd. for $C_{26}H_{46}O_{11}Na$ $[M+Na]^+$: 557.29323, found: 557.29351.

Table S20. Analysis of the NMR data of the 40*S*-configured compound **70**

Atom	*δ* (ppm)	*J* (Hz)	COSY	HSQC	HMBC	NOESY
**18 C**	63.12			18	19a, 19b, 20a, 20b	
**H2**	3.59		19a, 19b	18	19, 20	19a, 19b, 20a, 20b
**19 C**	30.51			19a, 19b	18, 20a, 20b, 21	
**Ha**	1.64		18, 19b, 20a, 20b	19	18, 20, 21	18, 21
**Hb**	1.67		18, 19a, 20a, 20b	19	18, 20, 21	18, 21
**20 C**	26.5			20a, 20b	18, 19a, 19b, 21	
**Ha**	1.66		19a, 19b, 21	20	18, 19, 21, 22	18, 21
**Hb**	1.69		19a, 19b, 21	20	18, 19, 21, 22	18, 21
**21 C**	84.36			21	19a, 19b, 20a, 20b, 22, 23a, 23b	
**H**	3.55		20a, 20b, 22	21	19, 20, 22	19a, 19b, 20a, 20b, 22, 23b, 24
**22 C**	73.22			22	20a, 20b, 21, 23a, 23b, 24	
**H**	4.19		21, 23a, 23b	22	21, 24	21, 23b, 24
**23 C**	42.34			23a, 23b		
**Ha**	1.5		22, 23b, 24	23	21, 22, 25	
**Hb**	2.39		22, 23a, 24	23	21, 22, 24, 25	21, 22
**24 C**	79.01			24	22, 23b, 25a, 25b, 26a, 26b	
**H**	3.76		23a, 23b, 25a, 25b	24	22, 26	21, 22, 25a, 25b, 26a
**25 C**	37.34			25a, 25b	23a, 23b, 27a, 27b	
**Ha**	1.57		24, 25b, 26a, 26b	25	24, 26, 27	24
**Hb**	1.71		24, 25a, 26a, 26b	25	24, 26, 27	24
**26 C**	27.62			26a, 26b	24, 25a, 25b, 27a, 27b, 28	
**Ha**	1.4		25a, 25b, 26b, 27a, 27b	26	24, 27, 28	24
**Hb**	1.48		25a, 25b, 26a, 27a, 27b	26	24, 27, 28	
**27 C**	27.58			27a, 27b	25a, 25b, 26a, 26b, 28, 29	
**Ha**	1.42		26a, 26b, 27b, 28	27	25, 26	29
**Hb**	1.49		26a, 26b, 27a, 28	27	25, 26	29
**28 C**	30.11			28	26a, 26b, 29	
**H2**	1.65		27a, 27b, 29	28	26, 27, 29	29
**29 C**	84.63			29	28, 30, 31a, 31b	
**H**	3.56		28, 30	29	27, 28, 30, 31	27a, 27b, 28, 30, 31b, 32
**30 C**	73.27			30	29, 31a, 31b	
**H**	4.18		29, 31a, 31b	30	29, 32	29, 31b
**31 C**	42.66			31a, 31b	29, 33a, 33b	
**Ha**	1.55	13.60(31b), 6.70(32), 2.30(30)	30, 31b, 32	31	29, 30, 32, 33	

**Hb**	2.42	13.60(31a), 8.00(32), 6.60(30)	30, 31a, 32	31	29, 30, 32, 33	29, 30, 32
**32 C**	75.37			32	30, 31a, 31b, 33a, 33b, 34	
**H**	4.07	8.00(31b), 3.10(33a), 9.32(33b), 6.70(31a)	31a, 31b, 33a, 33b	32	34	29, 31b, 33a, 34
**33 C**	41.54			33a, 33b	31a, 31b, 34, 35'	
**Ha**	1.53	3.10(32), 9.90(34), 14.20(33b)	32, 33b, 34	33	31, 32, 34, 35	32, 35'
**Hb**	1.83	9.32(32), 14.20(33a), 2.10(34)	32, 33a, 34	33	31, 32, 35	34, 35', 36b
**34 C**	67.26			34	32, 33a, 35', 36a	
**H**	4.18	9.60(35'), 9.90(33a), 2.10(33b)	33a, 33b, 35'	34	32, 33, 35, 36	32, 33b, 36b, 37, 39'
**35 C**	77.76			35'	33a, 33b, 34, 36a, 36b, 39'	
**35' H**	3.63	9.60(34), 6.20(36a), 1.80(36b)	34, 36a, 36b	35	33, 34, 36, 37, 39	33a, 33b, 36a, 36b
**36 C**	33.99			36a, 36b	34, 35', 37, 38	
**Hax**	1.72	11.20(37), 13.60(36b), 6.20(35')	35', 36b, 37	36	34, 35, 37, 38	35', 38
**Heq**	2.04	13.60(36a), 5.10(37), 1.80(35')	35', 36a, 37	36	35, 37, 38	33b, 34, 35', 37
**37 C**	70.98			37	35', 36a, 36b, 38, 39'	
**H**	3.77	8.50(38), 11.20(36a), 5.10(36b)	36a, 36b, 38	37	36, 38	34, 36b, 39'
**38 C**	72.61			38	36a, 36b, 37, 39', 40	
**H**	3.5	9.40(39'), 8.50(37)	37, 39'	38	36, 37, 39, 40	36a, 41
**39 C**	73.12			39'	35', 38, 40, 41	
**39' H**	3.71	9.40(38)	38, 40	39	35, 37, 38, 40	34, 37, 41
**40 C**	72.59			40	38, 39', 41, 42	
**H**	3.69	8.50(41)	39', 41	40	38, 39, 41, 42	42
**41 C**	74.18			41	40, 42, 43cis, 43trans	
**H**	4.15	8.50(40), 5.90(42), 1.40(43cis), 1.40(43trans)	40, 42, 43cis, 43trans	41	39, 40, 42, 43	38, 39', 42, 43trans
**42 C**	140.44			42	40, 41, 43trans	
**H**	6.05	10.60(43cis), 17.30(43trans), 5.90(41)	41, 43cis, 43trans	42	40, 41	40, 41
**43 C**	116.12			43cis, 43trans	41	
**Hcis**	5.18	10.60(42), 1.90(43trans), 1.30(41)	41, 42, 43trans	43	41	
**Htrans**	5.33	17.30(42), 1.90(43cis), 1.50(41)	41, 42, 43cis	43	41, 42	41

Table S21. Comparison of the NMR data of authentic benthol A with those of the 40*S*-configured compound **70**

Atom	δ (ppm, ^{1}H NMR in CD_3OD)		Δδ	δ (ppm, ^{13}C NMR in CD_3OD)		Δδ
	Authentic benthol A	**70** (40*S*-isomer)		Authentic benthol A	**70** (40*S*-isomer)	
**18**	4.05	3.59	0.46	73.8	63.1	10.7
**19a**	1.63	1.64	−0.01	35.1	30.5	4.6
**19b**	1.63	1.67	−0.04			
**20a**	1.64	1.66	−0.02	26.2	26.5	−0.3
**20b**	1.71	1.69	0.02			
**21**	3.53	3.55	−0.02	84.5	84.4	0.1
**22**	4.18	4.19	−0.01	73.2	73.2	0.0
**23a**	1.49	1.50	−0.01	42.4	42.3	0.1
**23b**	2.38	2.39	−0.01			
**24**	3.75	3.76	−0.01	79.1	79.0	0.1
**25a**	1.56	1.57	−0.01	37.4	37.3	0.1
**25b**	1.71	1.71	0.00			
**26a**	1.38	1.40	−0.02	27.7	27.6	0.1
**26b**	1.48	1.48	0.00			
**27a**	1.40	1.42	−0.02	27.6	27.6	0.0
**27b**	1.48	1.49	−0.01			
**28a**	1.64	1.65	−0.01	30.2	30.1	0.1
**28b**	1.64	1.65	−0.01			
**29**	3.56	3.56	0.00	84.7	84.6	0.1
**30**	4.18	4.18	0.00	73.3	73.3	0.0
**31a**	1.55	1.55	0.00	42.7	42.7	0.0
**31b**	2.42	2.42	0.00			
**32**	4.08	4.07	0.01	75.4	75.4	0.0
**33a**	1.52	1.53	−0.01	41.7	41.5	0.2
**33b**	1.82	1.83	−0.01			
**34**	4.18	4.18	0.00	67.1	67.3	−0.2
**35**	3.62	3.63	−0.01	77.8	77.8	0.0
**36ax**	1.72	1.72	0.00	34.2	34.0	0.2
**36eq**	2.03	2.04	−0.01			
**37**	3.76	3.77	−0.01	71.1	71.0	0.1
**38**	3.49	3.50	−0.01	72.8	72.6	0.2
**39**	3.69	3.71	−0.02	73.2	73.1	0.1
**40**	3.62	3.69	−0.07	73.1	72.6	0.5
**41**	3.63	4.15	−0.52	72.5	74.2	−1.7

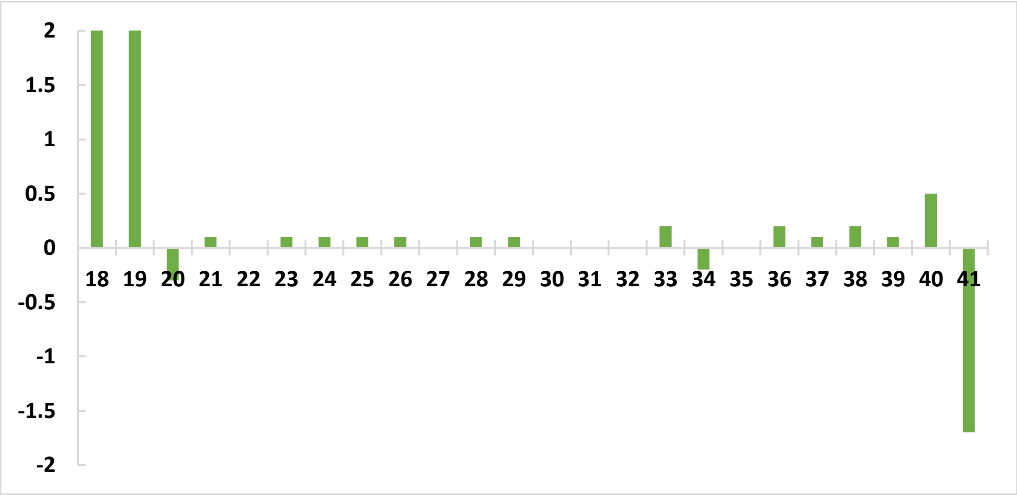

Figure S10. Comparison of the ^{13}C NMR shifts of compound **70** (40*S*-isomer) with those of the E-ring of authentic benthol A reported in the literature ($\Delta\delta_C$).

Copies of Spectra of New Compounds

Compound 4: ^{1}H NMR ($CDCl_3$, 400 MHz)

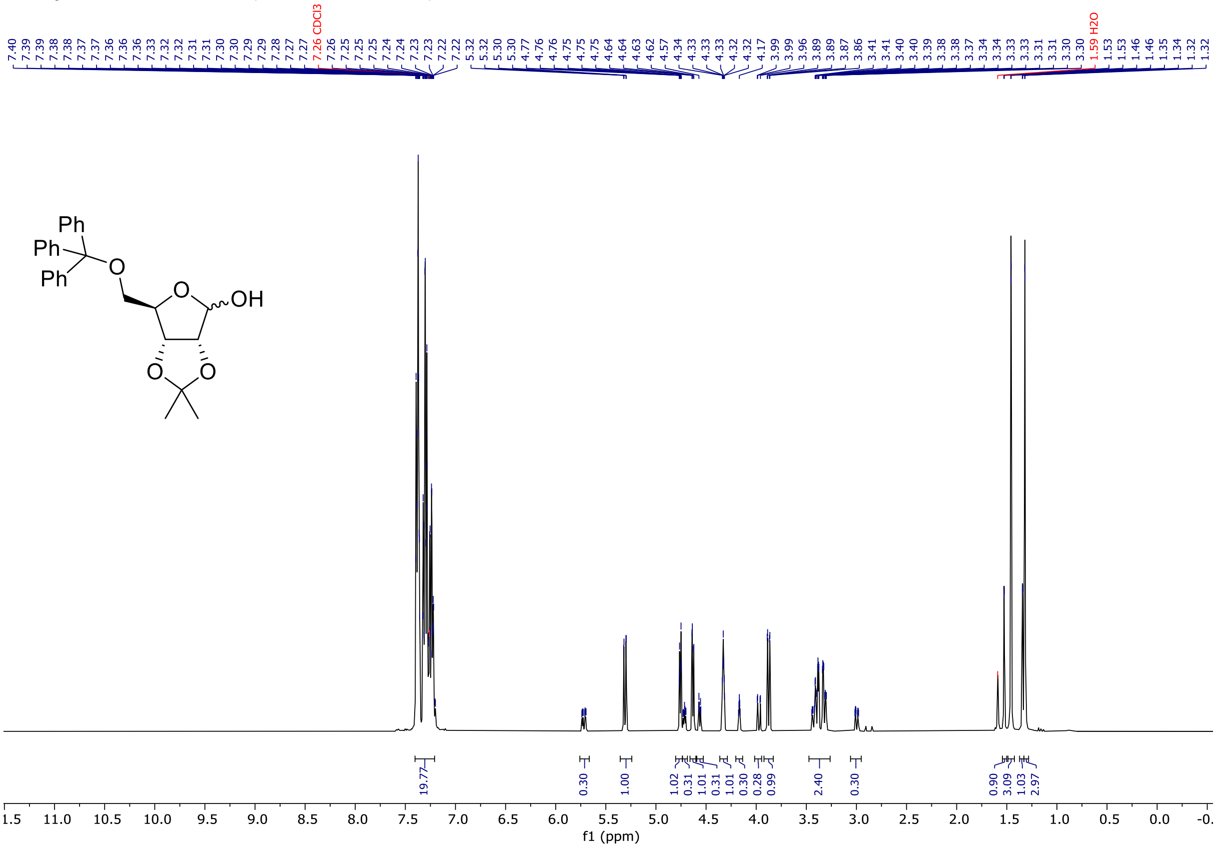

^{13}C NMR ($CDCl_3$, 101 MHz)

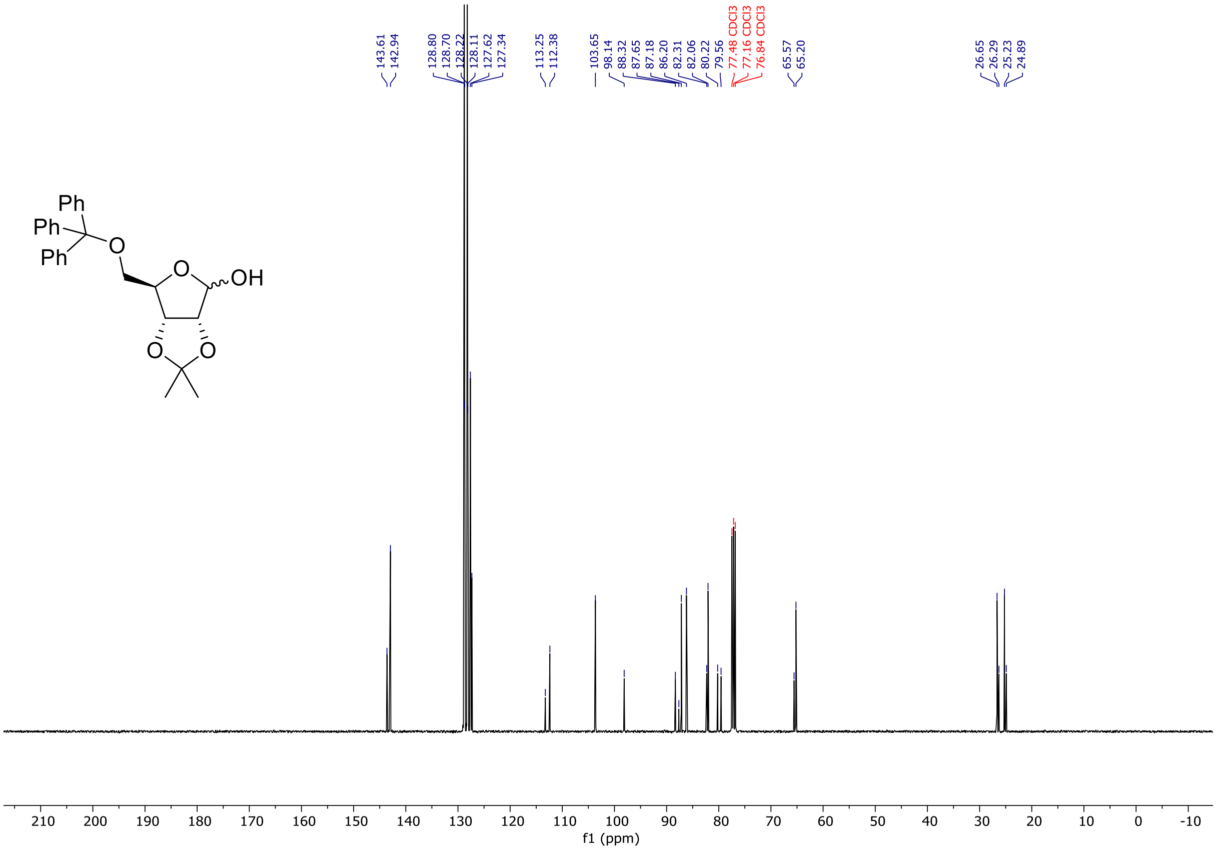

Compound 5: ^{1}H NMR ($CDCl_3$, 400 MHz)

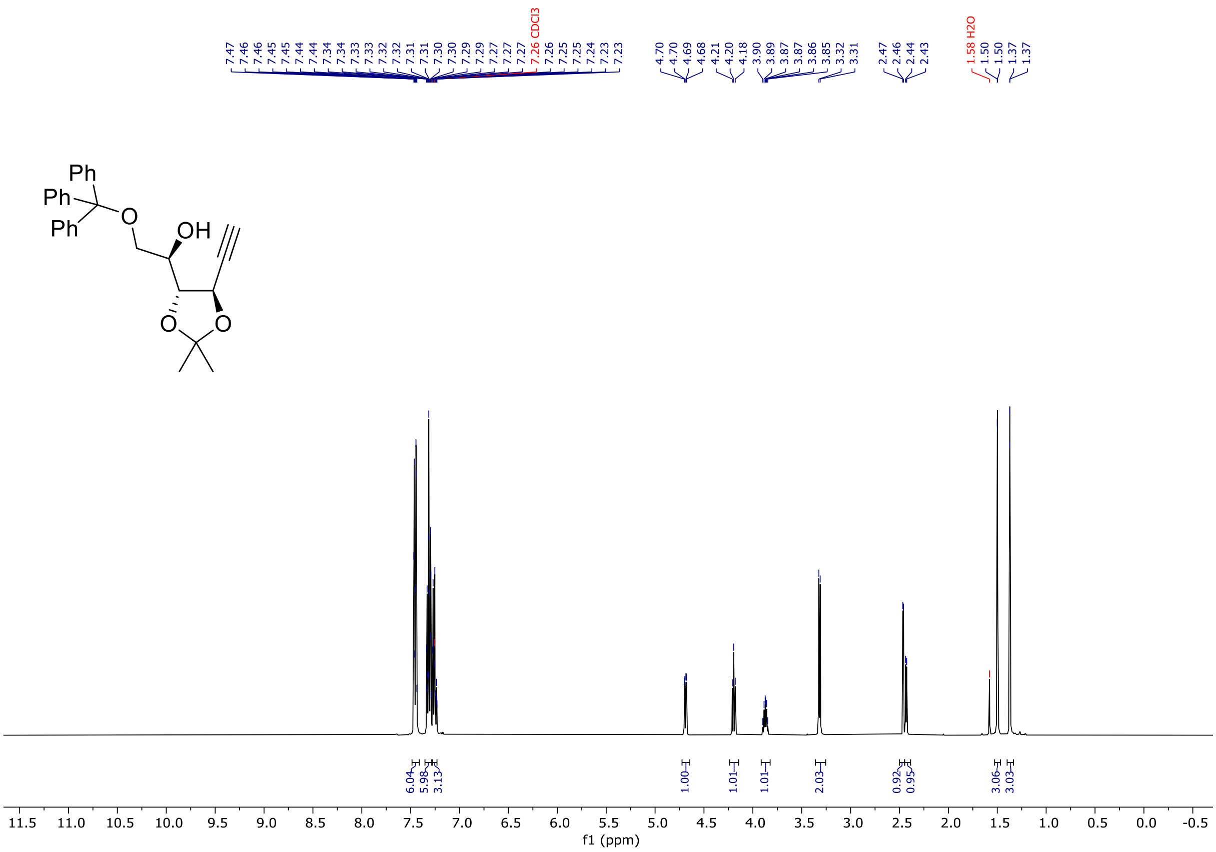

^{13}C NMR ($CDCl_3$, 101 MHz)

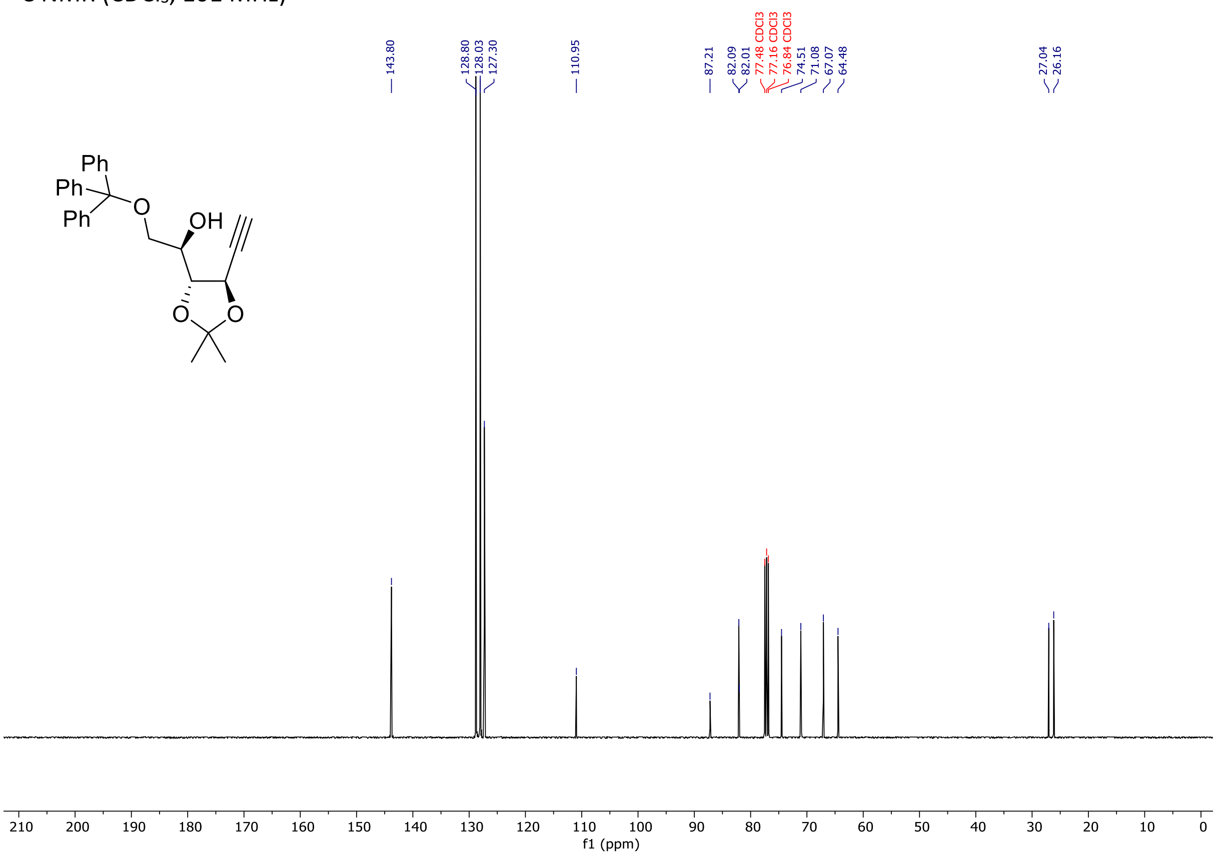

Compound 3: ^{1}H NMR ([D$_4$]-MeOH, 400 MHz)

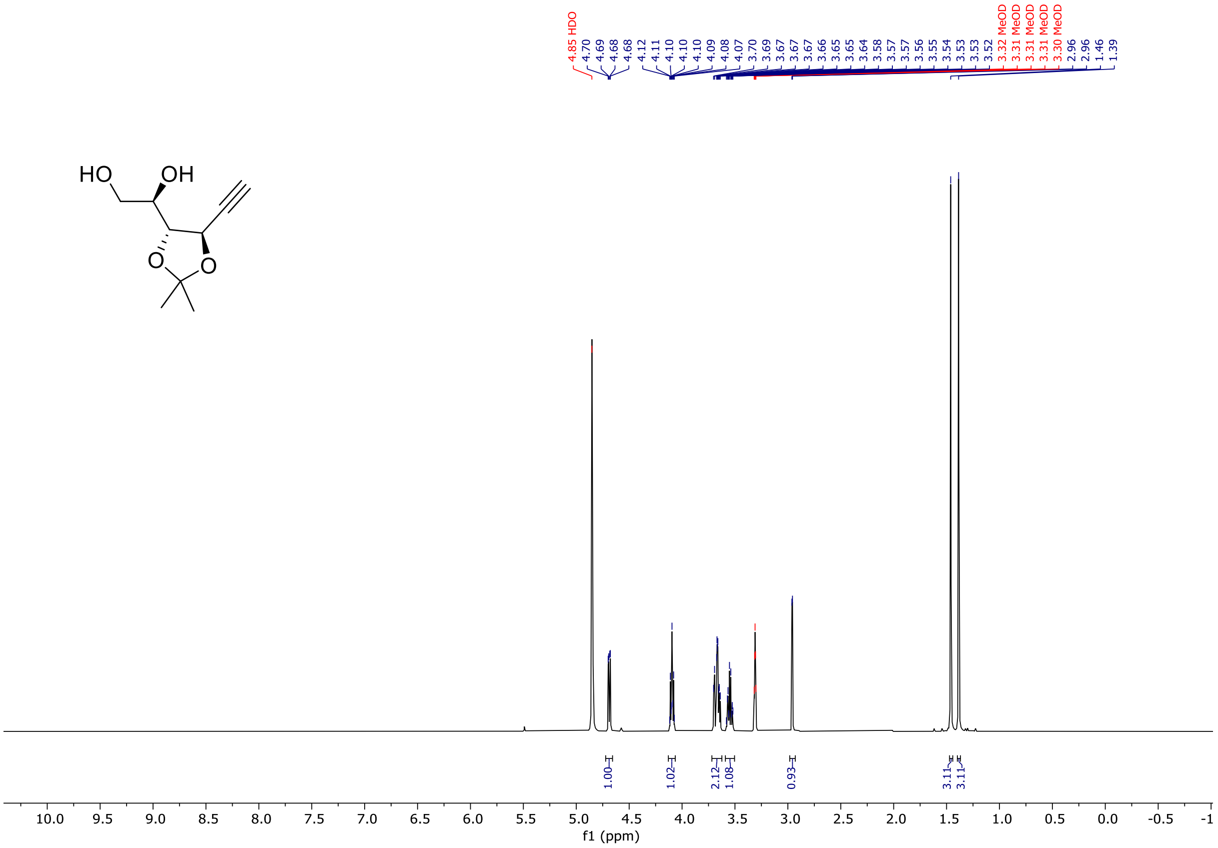

^{13}C NMR ([D$_4$]-MeOH, 101 MHz)

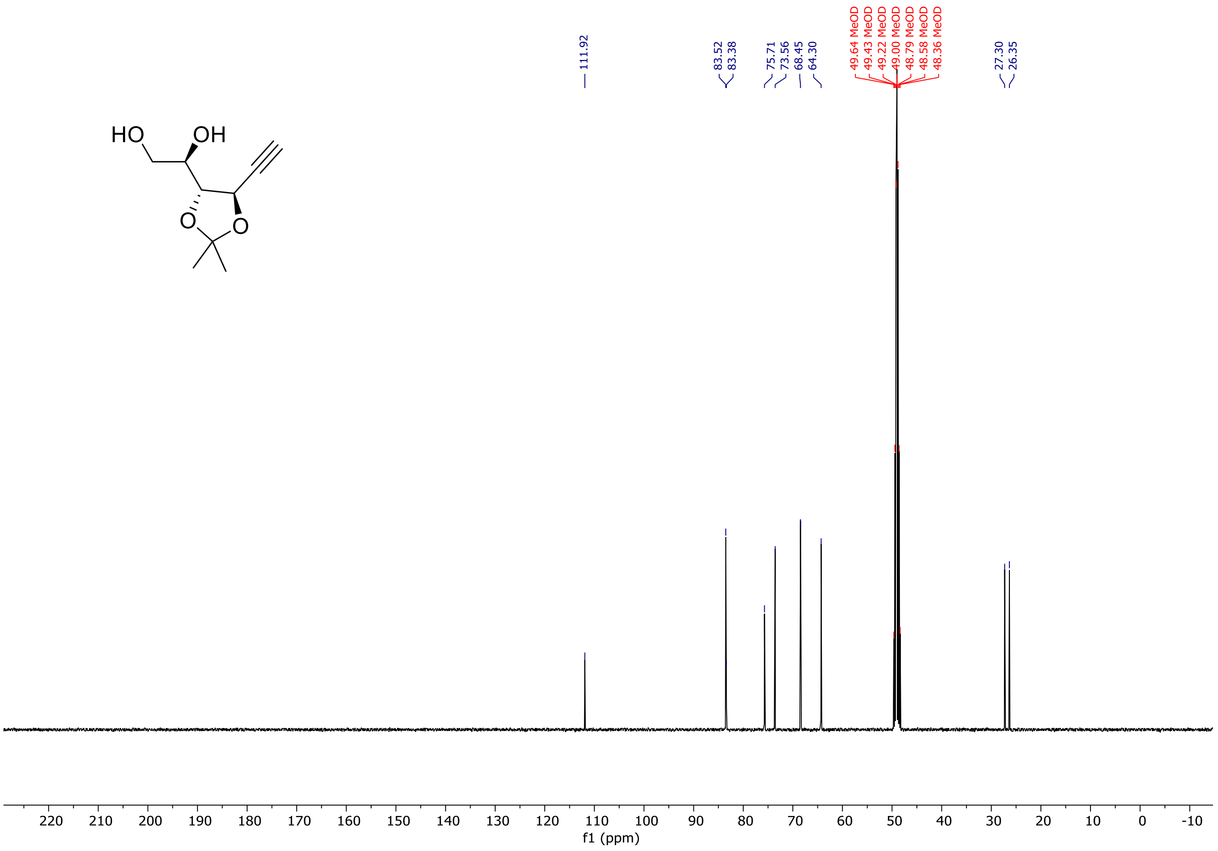

Compound 7: ^{1}H NMR (CD_2Cl_2, 400 MHz)

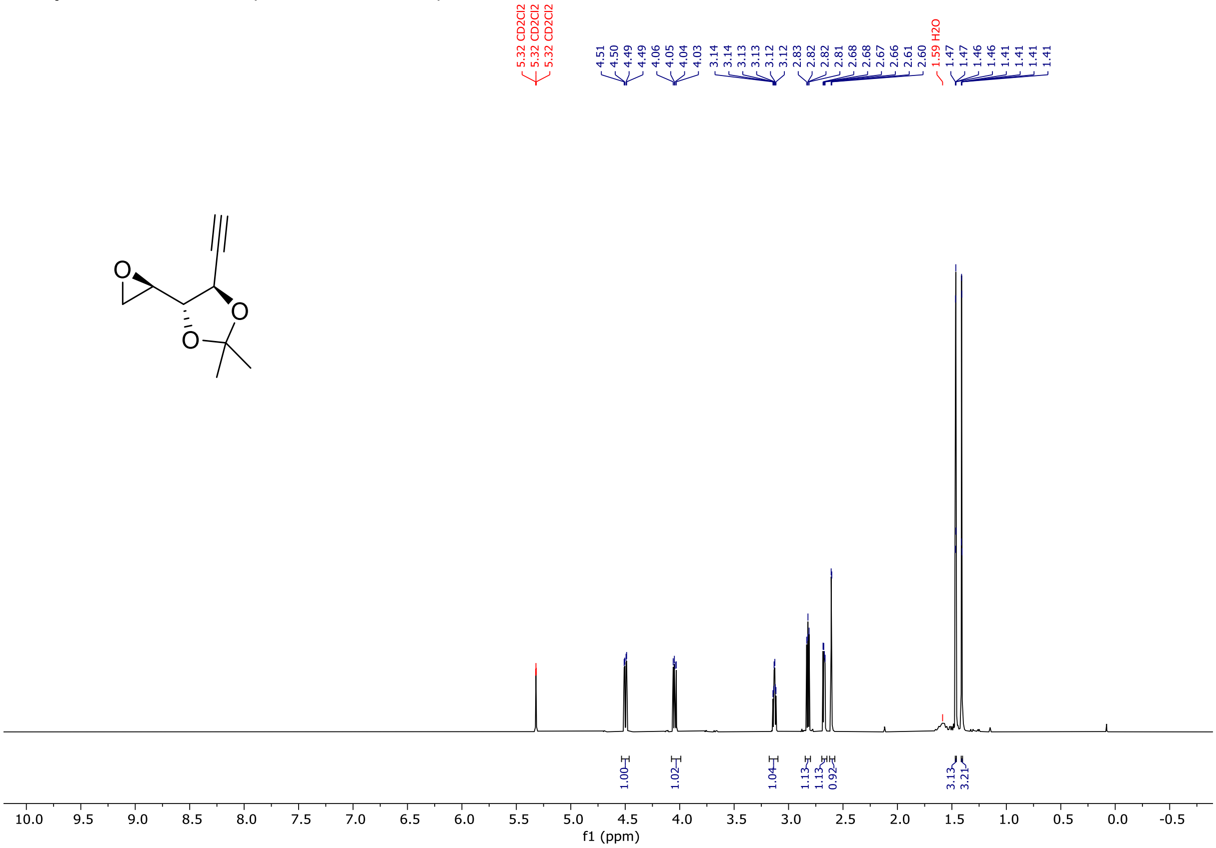

^{13}C NMR (CD_2Cl_2, 101 MHz)

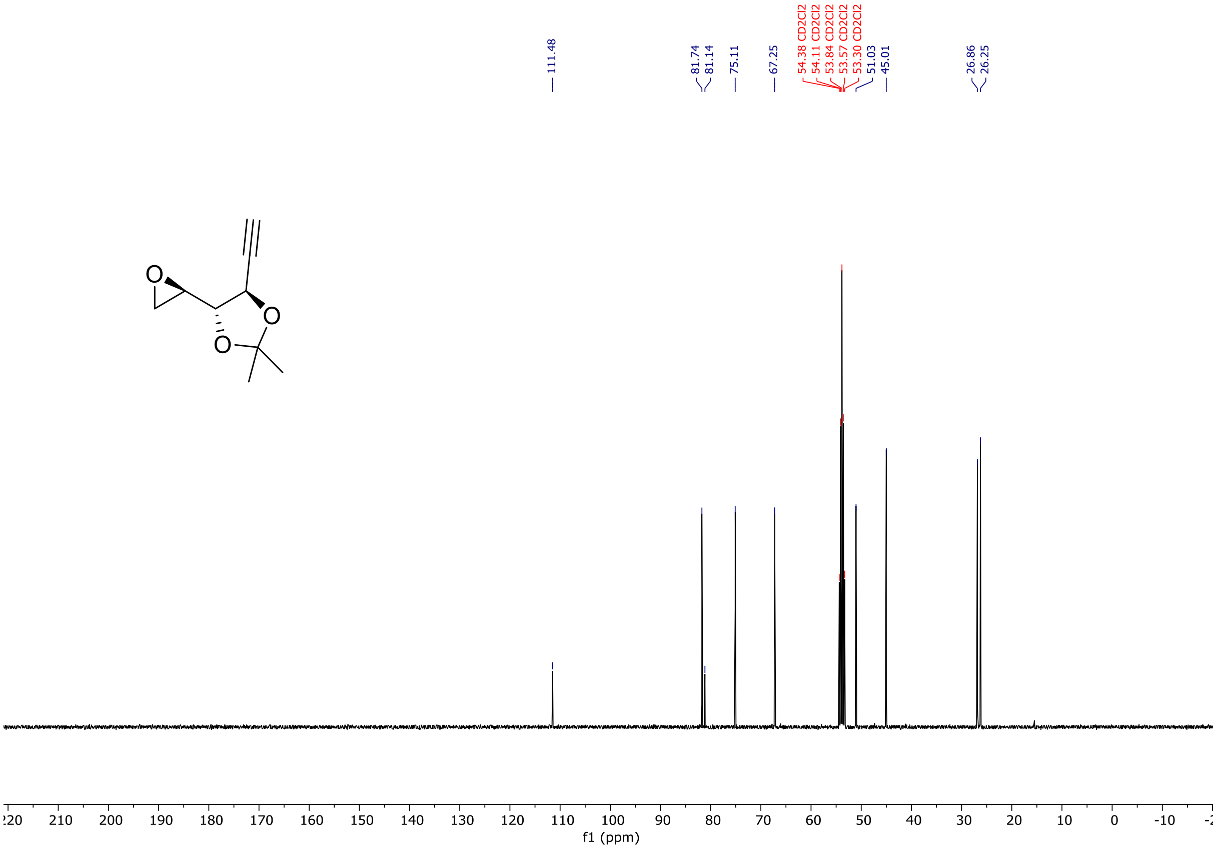

Compound S1: ^{1}H NMR ($CDCl_3$, 400 MHz)

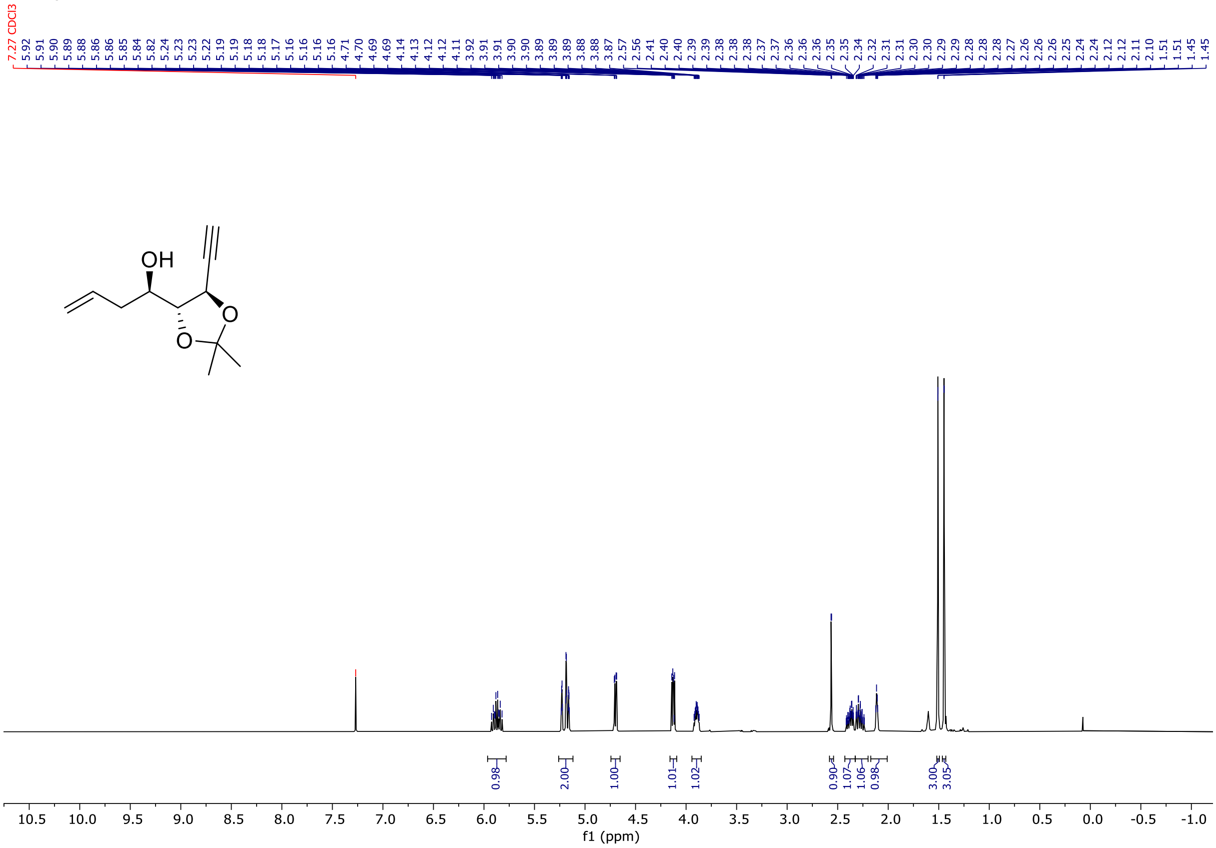

^{13}C NMR ($CDCl_3$, 101 MHz)

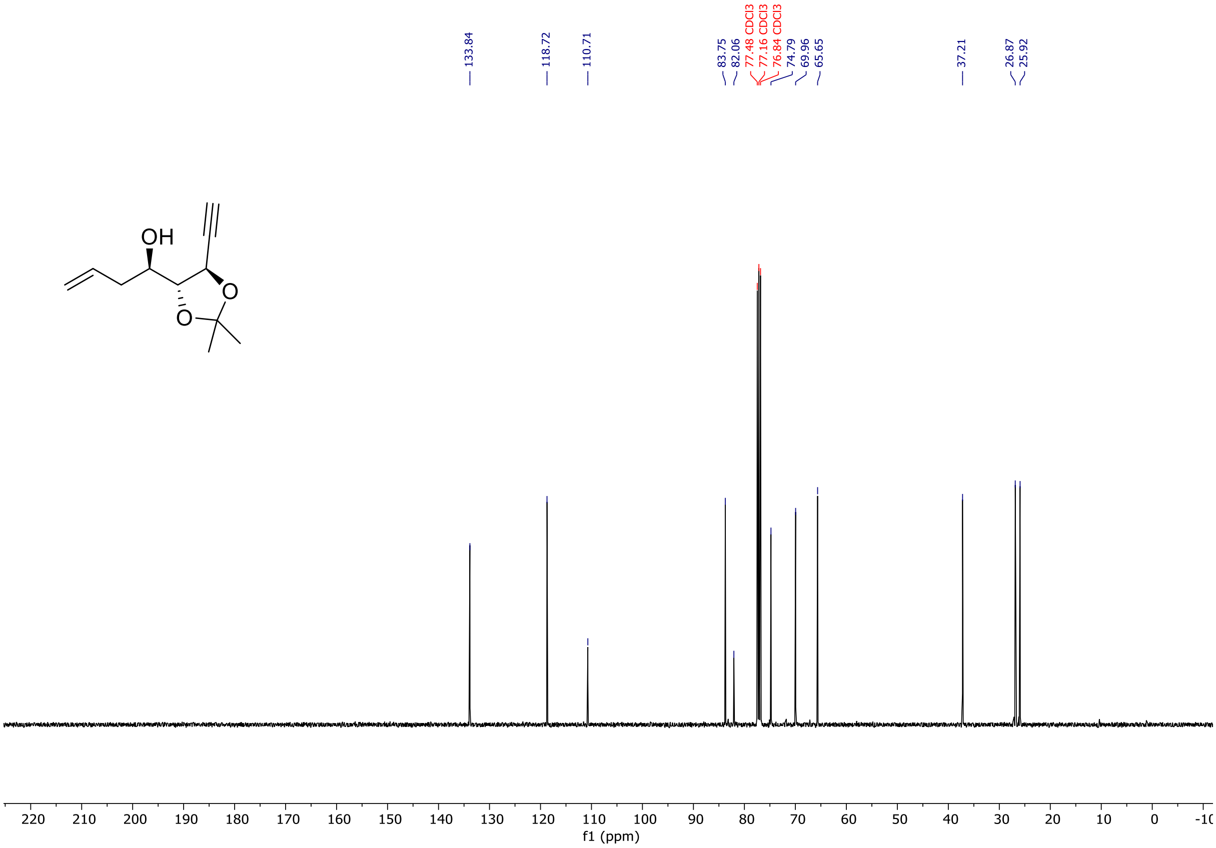

Compound 8: ^{1}H NMR ($CDCl_3$, 400 MHz)

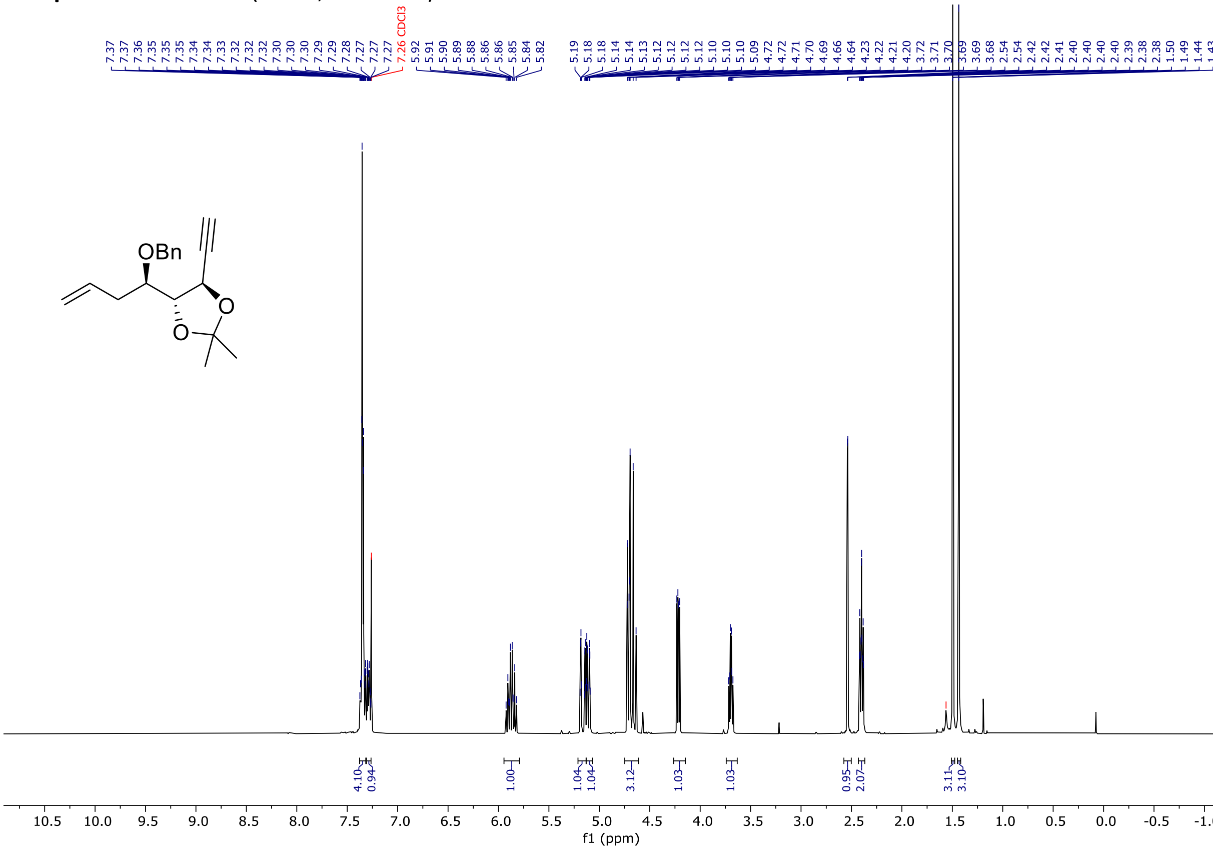

^{13}C NMR ($CDCl_3$, 101 MHz)

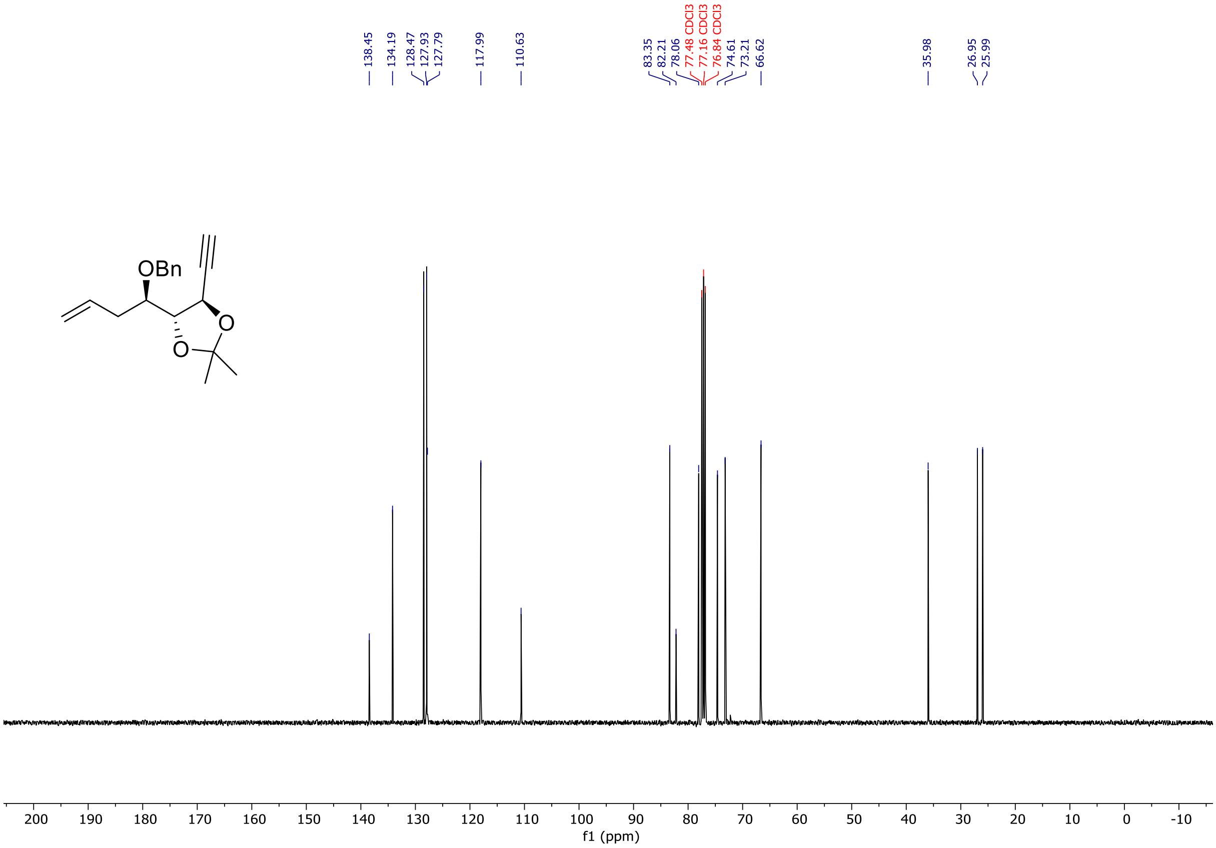

Compound S2: ^{1}H NMR ($CDCl_3$, 400 MHz)

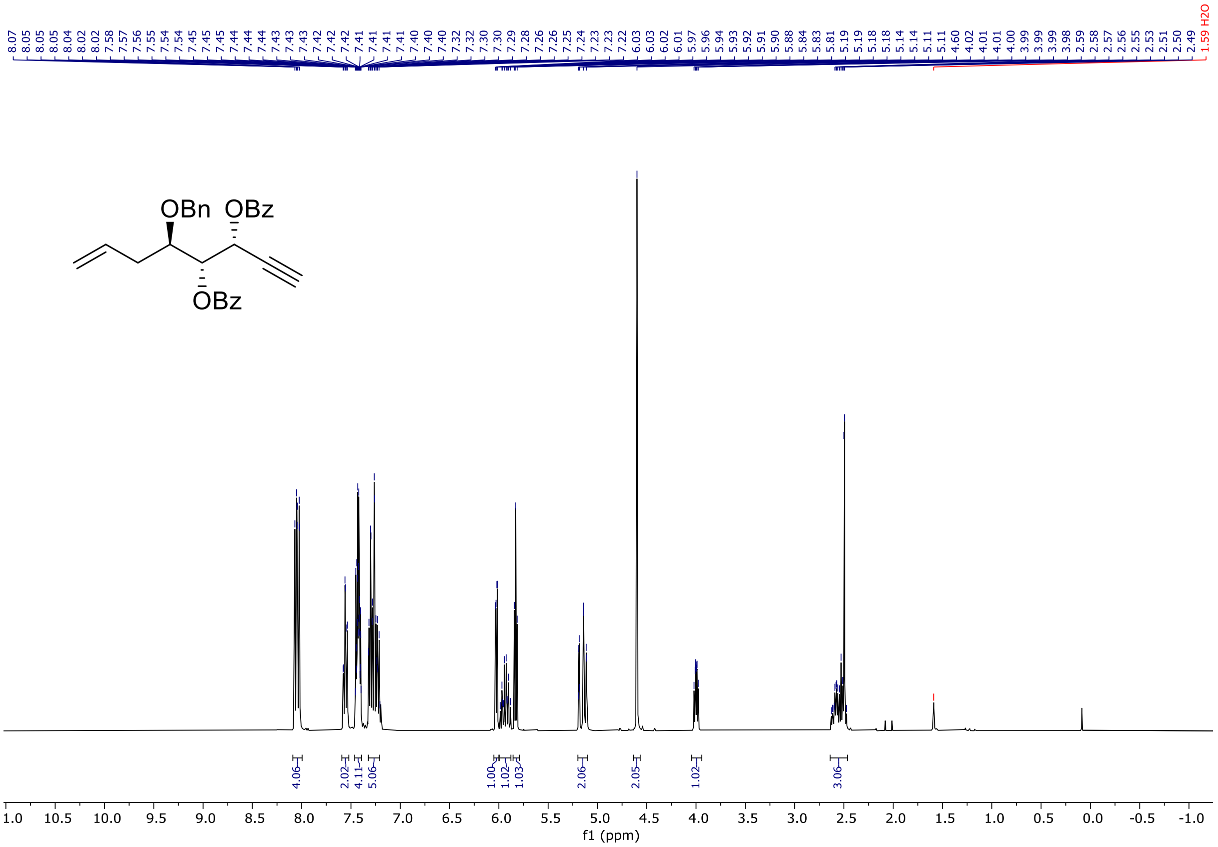

^{13}C NMR ($CDCl_3$, 101 MHz)

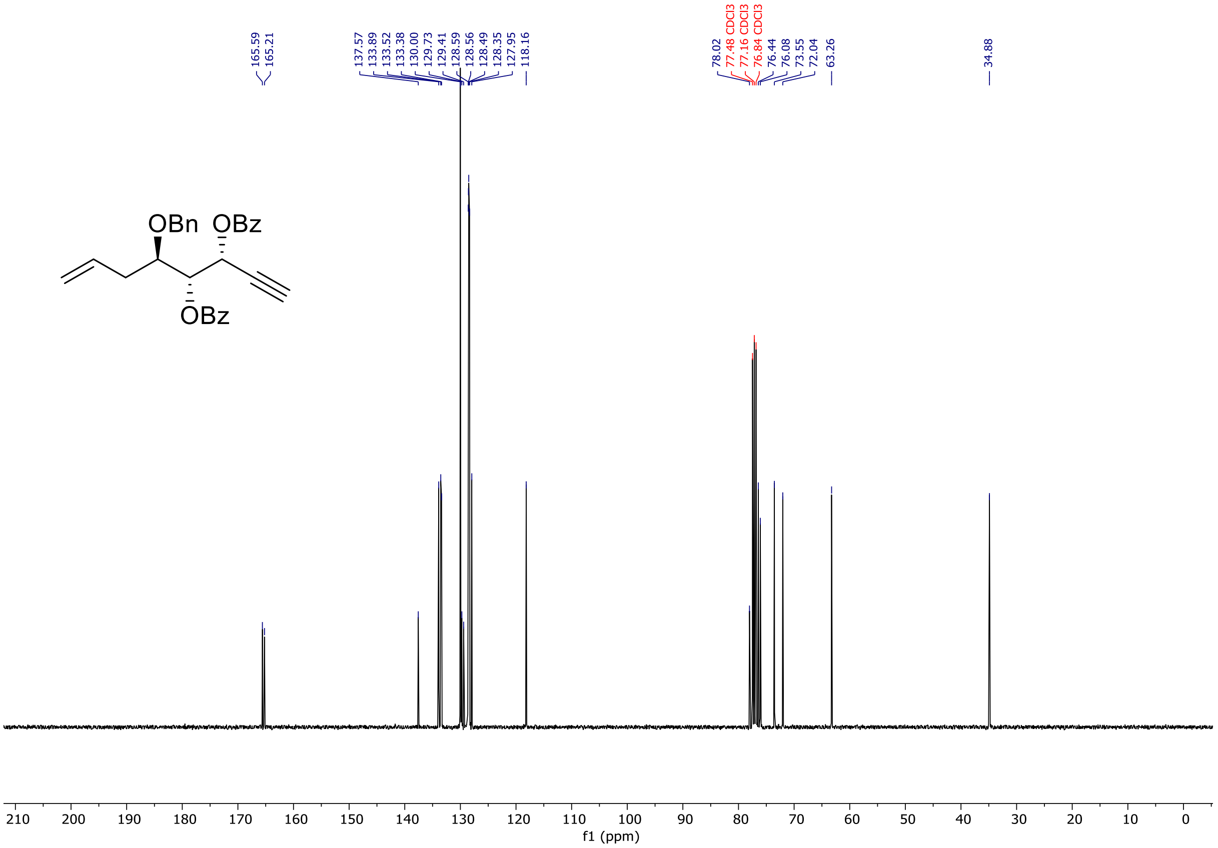

Compound S3: ^{1}H NMR ($CDCl_3$, 400 MHz)

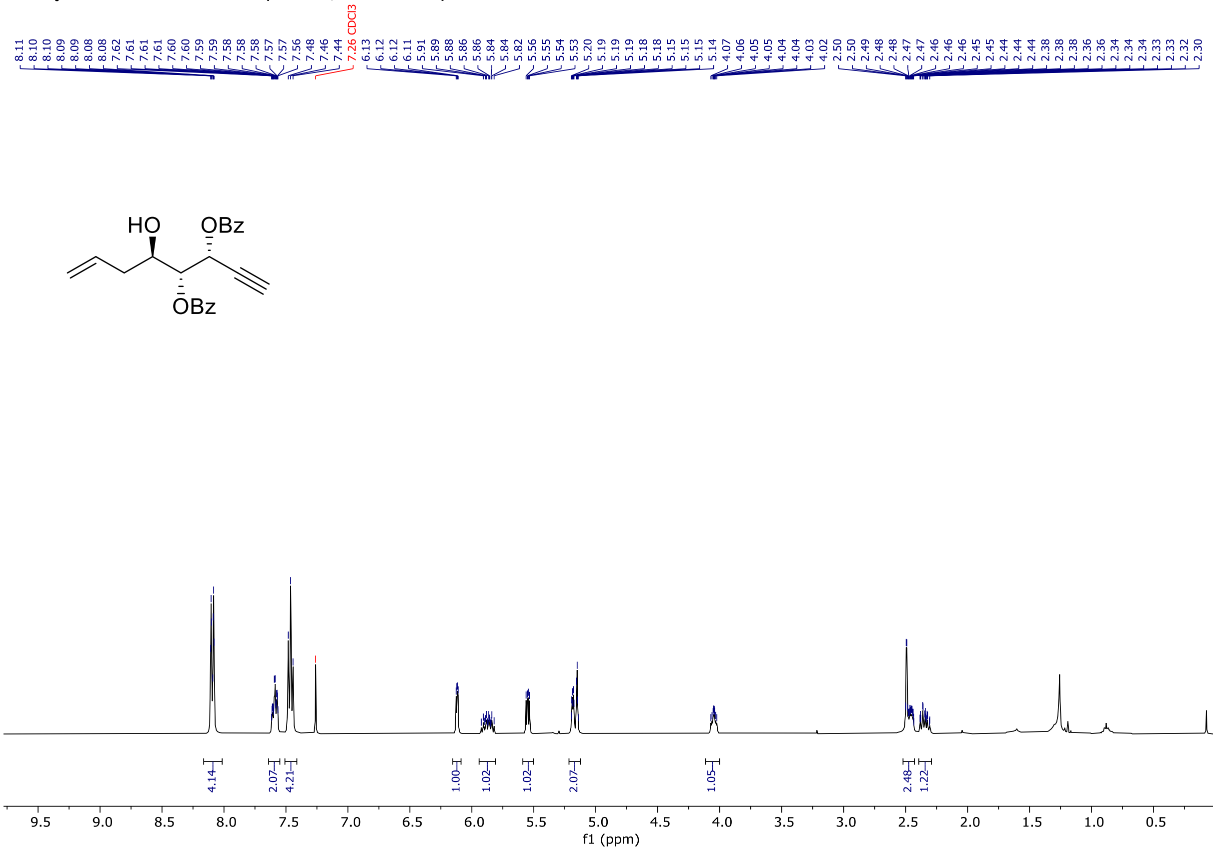

^{13}C NMR ($CDCl_3$, 101 MHz)

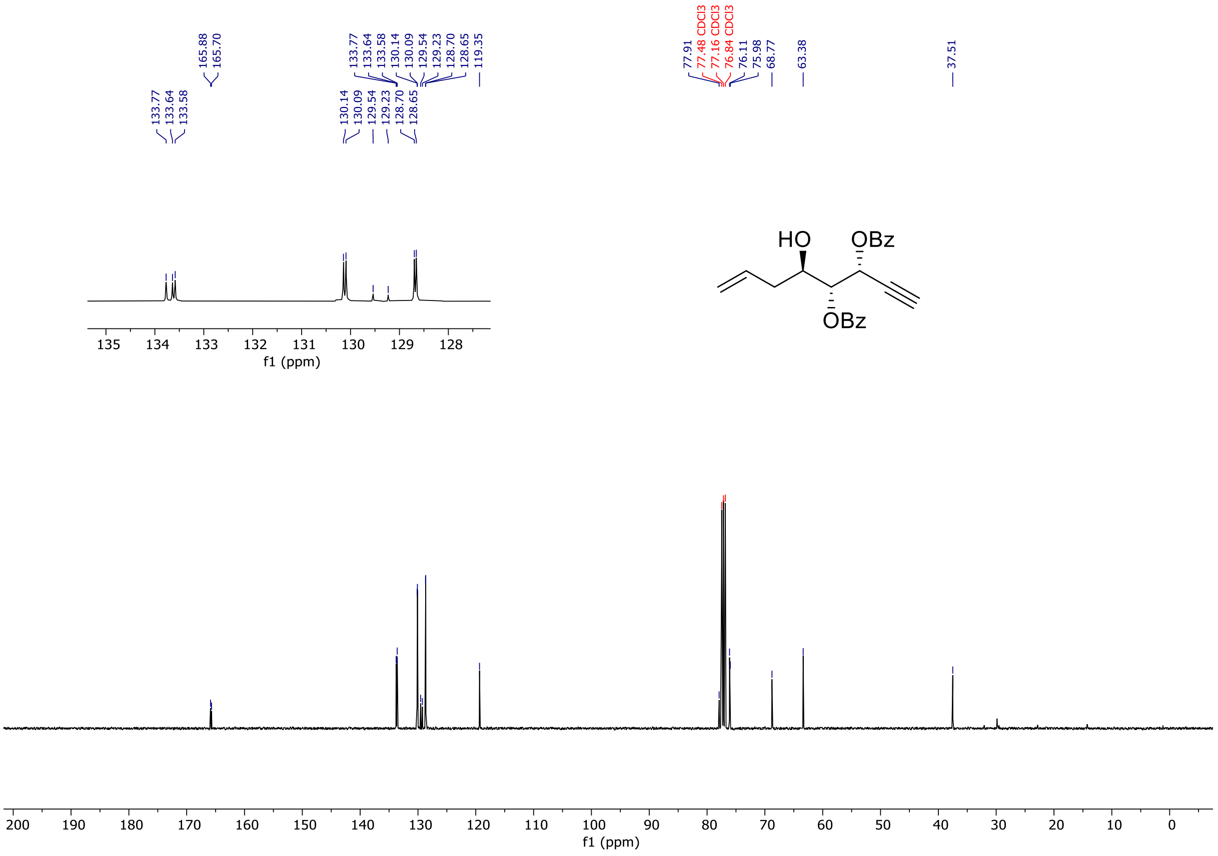

Compound 10: ^{1}H NMR ($CDCl_3$, 400 MHz)

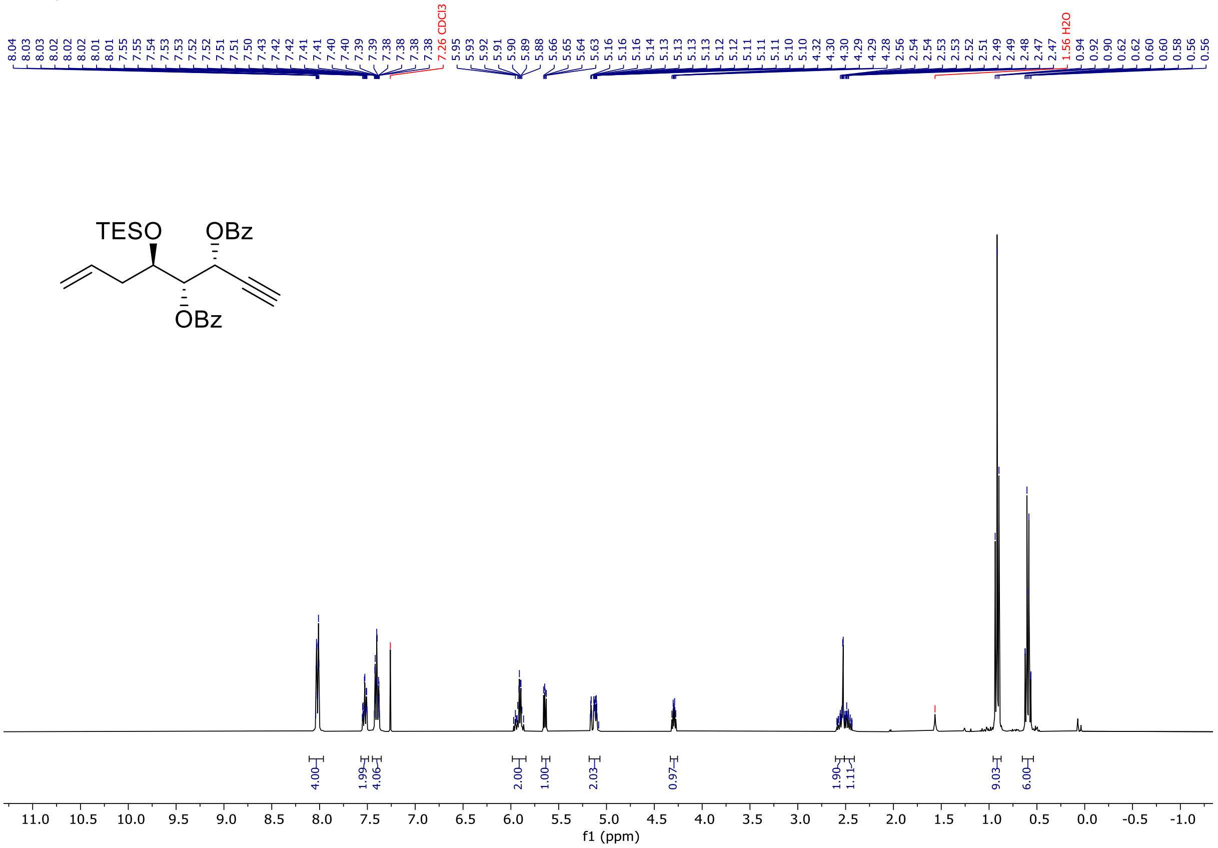

^{13}C NMR ($CDCl_3$, 101 MHz)

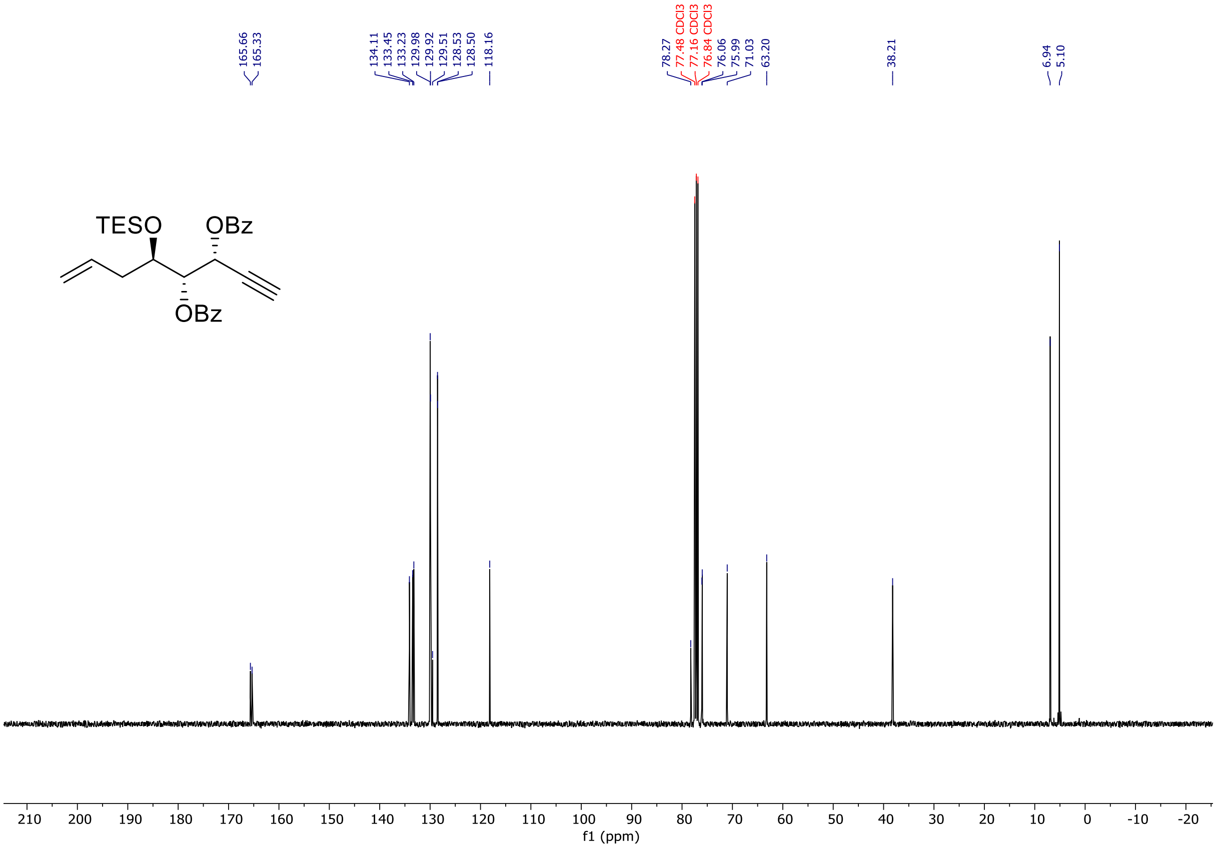

Compound S4: ^{1}H NMR ($CDCl_3$, 400 MHz)

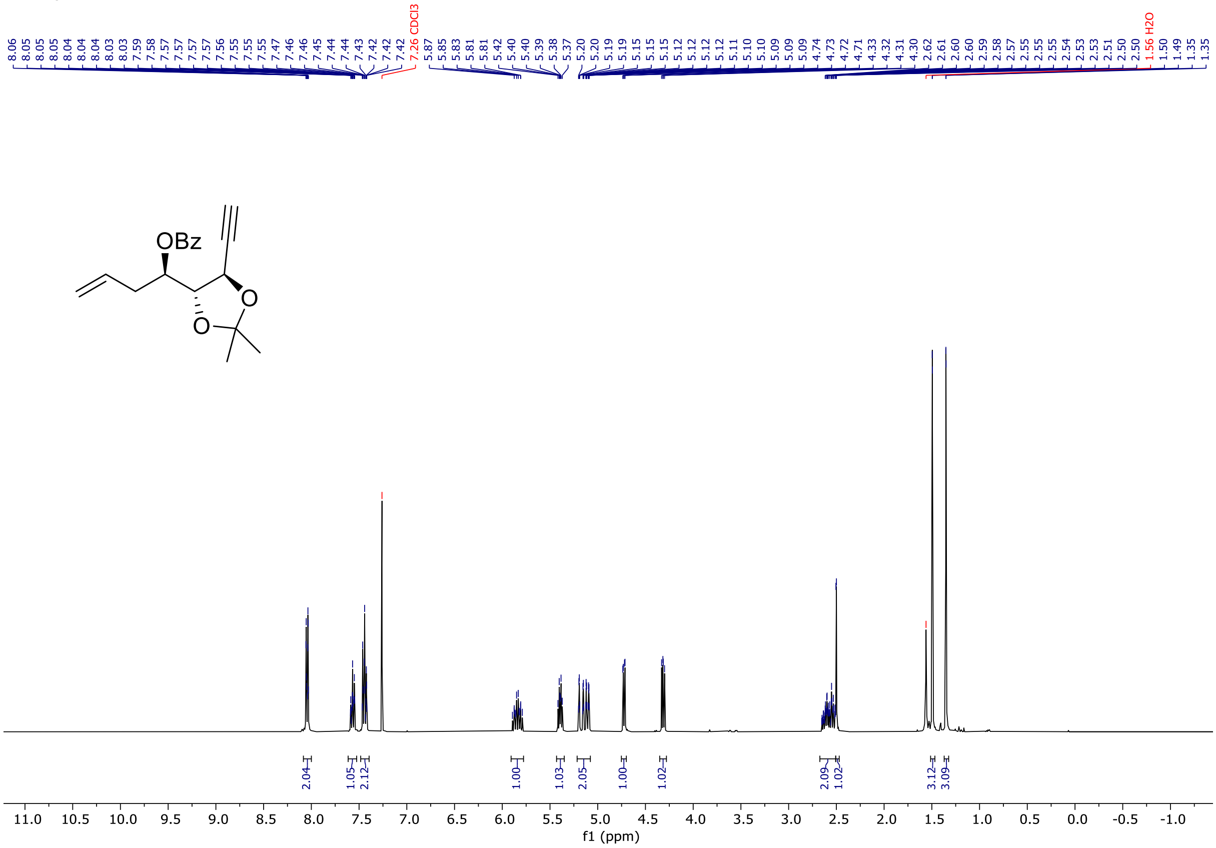

^{13}C NMR ($CDCl_3$, 101 MHz)

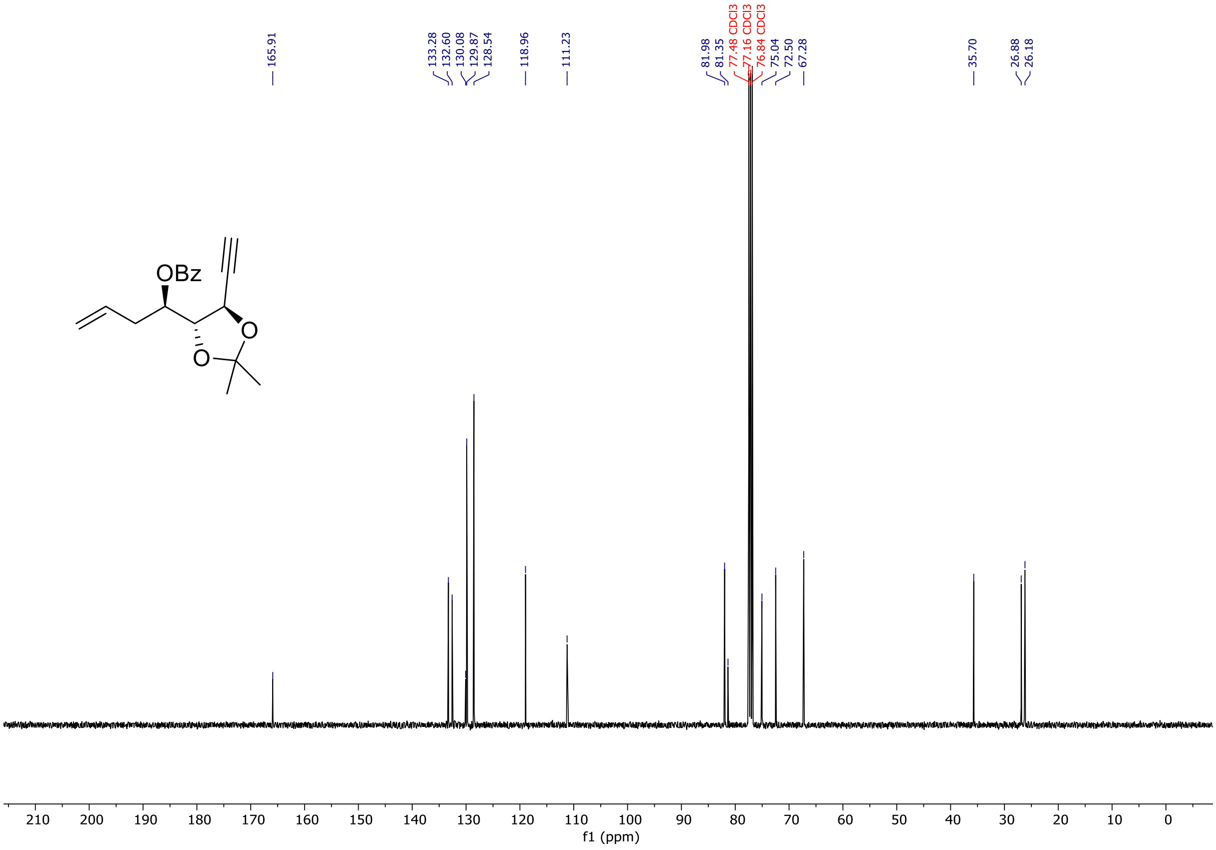

Compound S5: ^{1}H NMR ($CDCl_3$, 400 MHz)

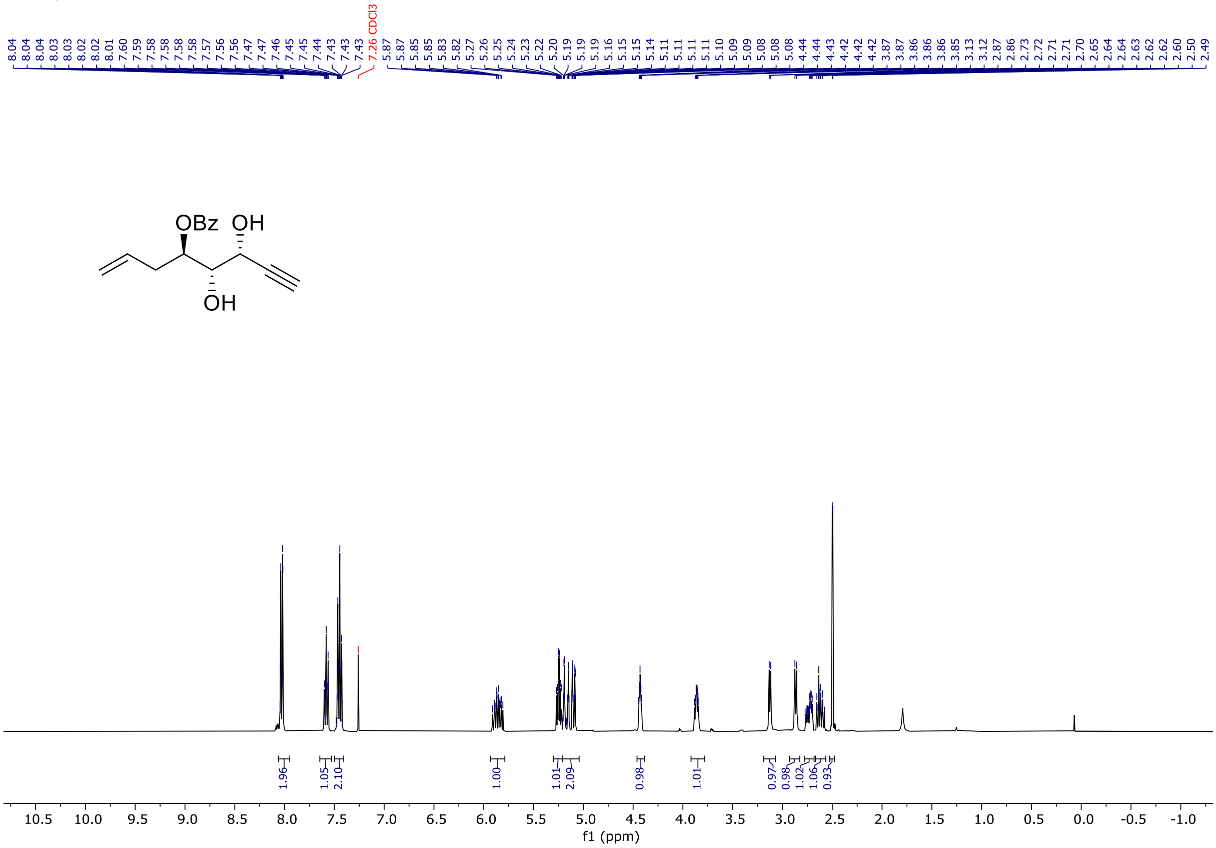

^{13}C NMR ($CDCl_3$, 101 MHz)

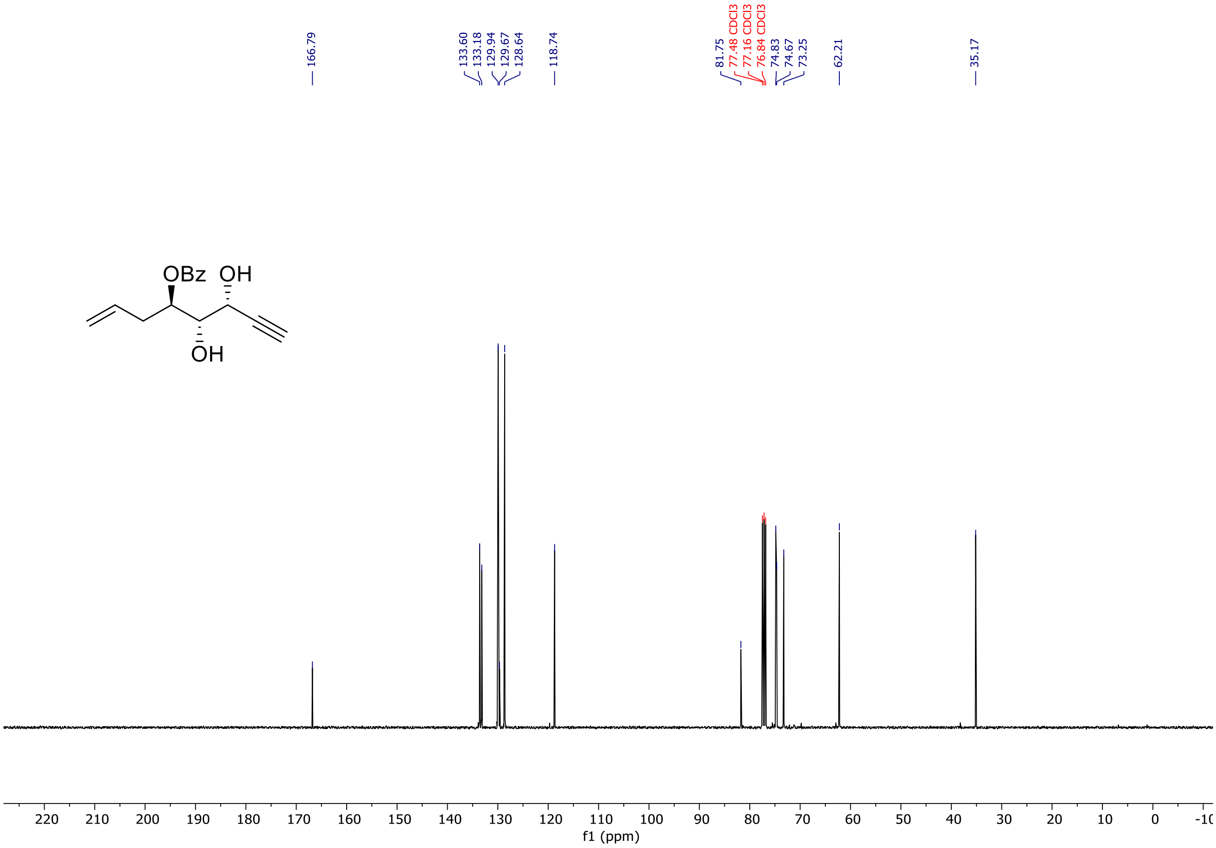

Compound 11: ^{1}H NMR ($CDCl_3$, 400 MHz)

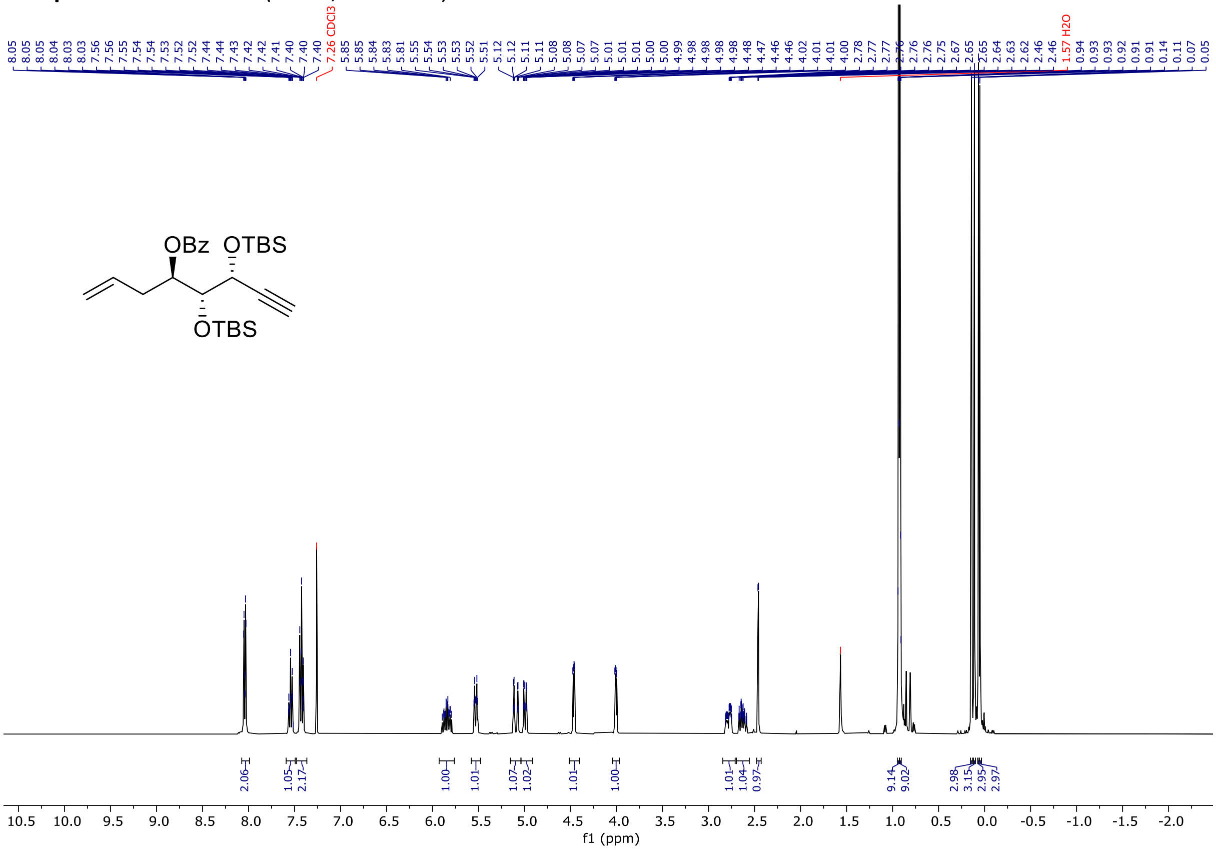

^{13}C NMR ($CDCl_3$, 101 MHz)

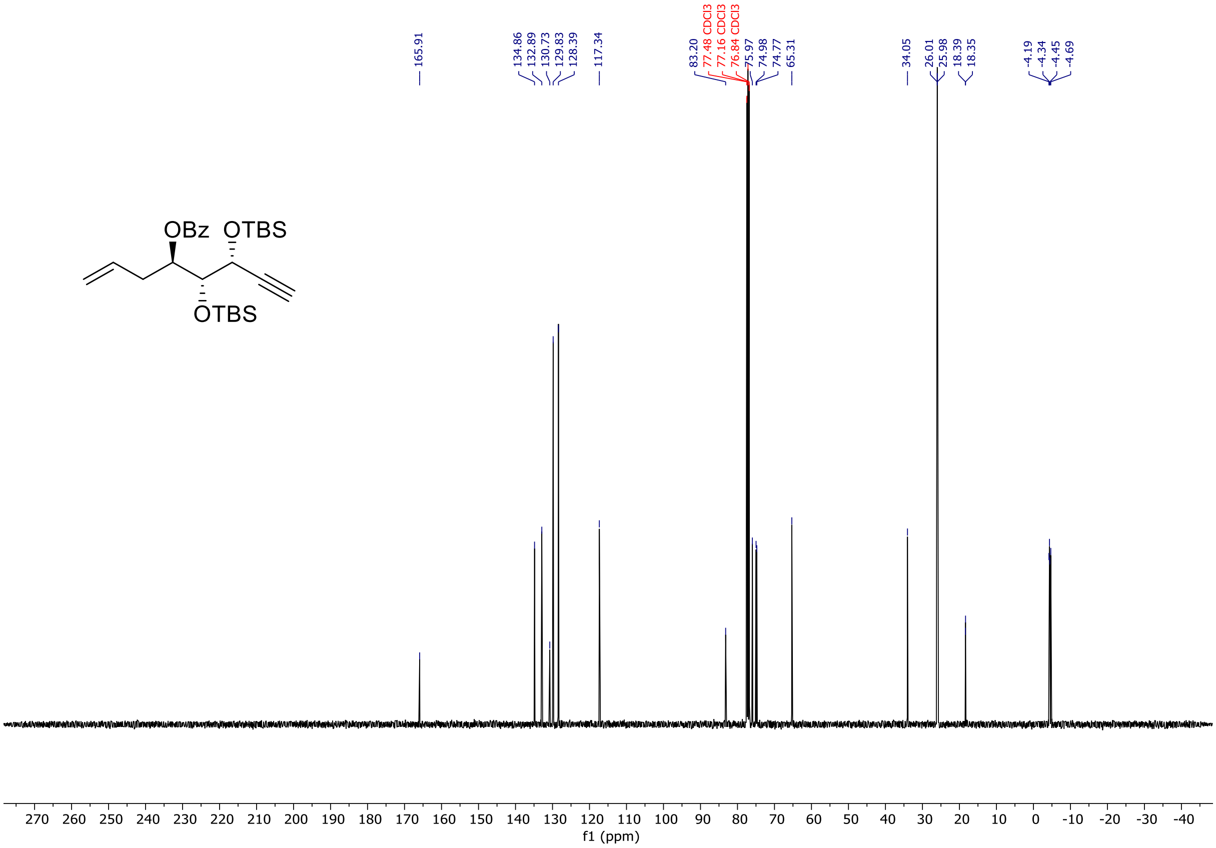

Compound 13: ^1H NMR ([D4]-MeOH, 400 MHz)

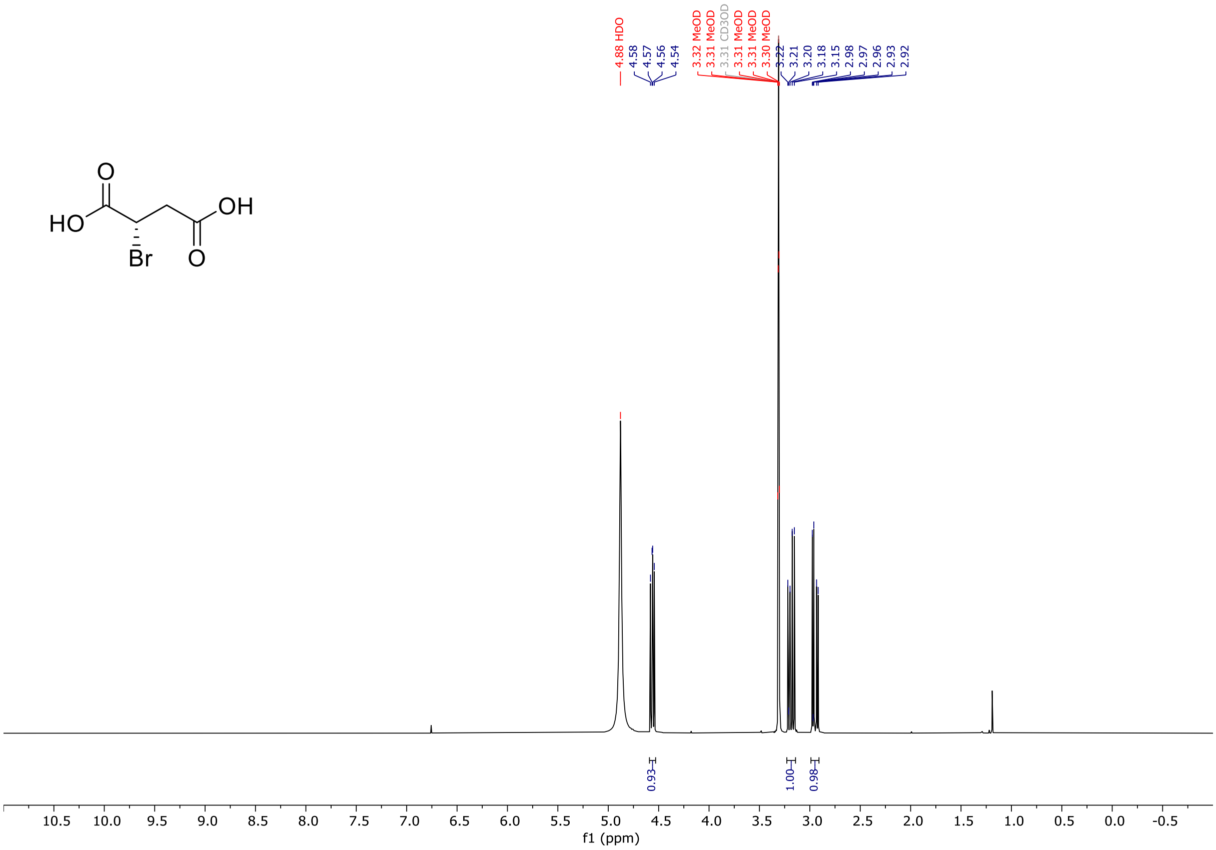

^{13}C NMR ([D4]-MeOH, 101 MHz)

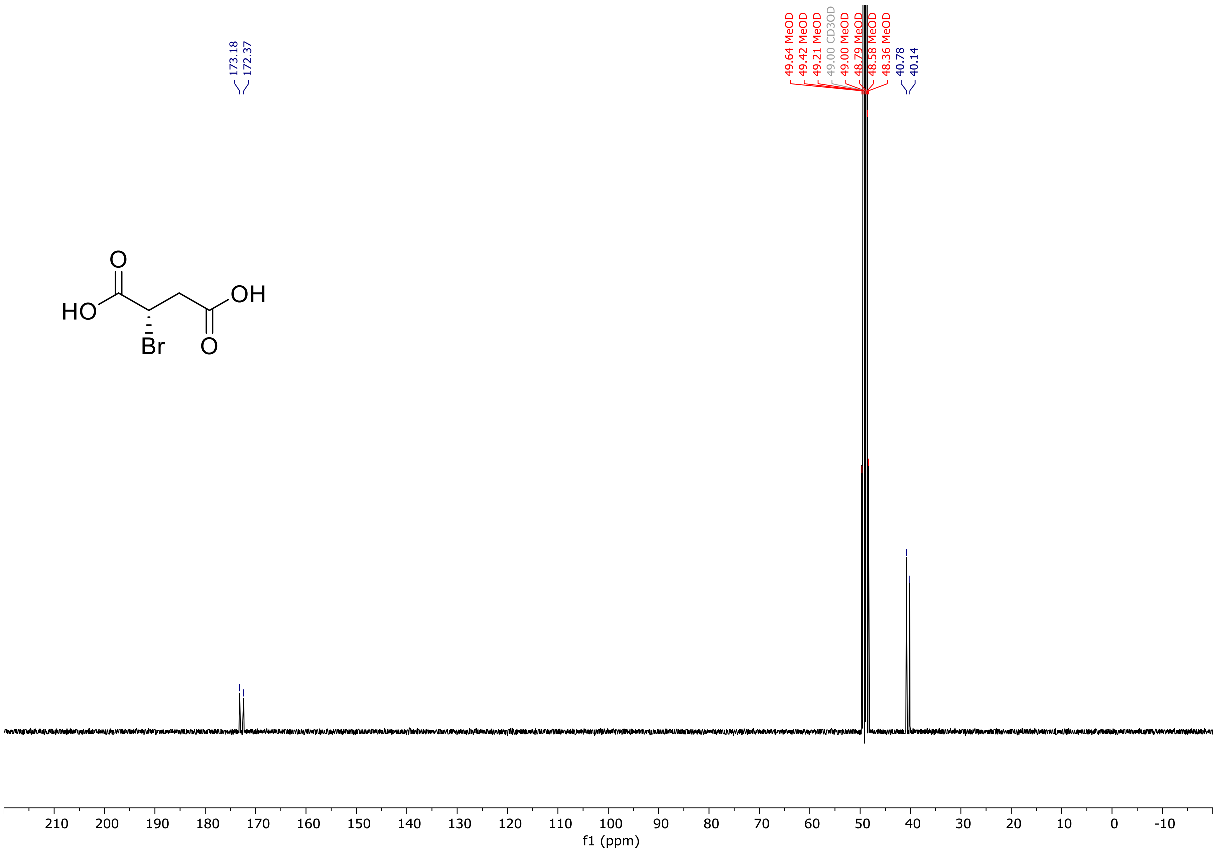

Compound S7: ^{1}H NMR ($CDCl_3$, 400 MHz)

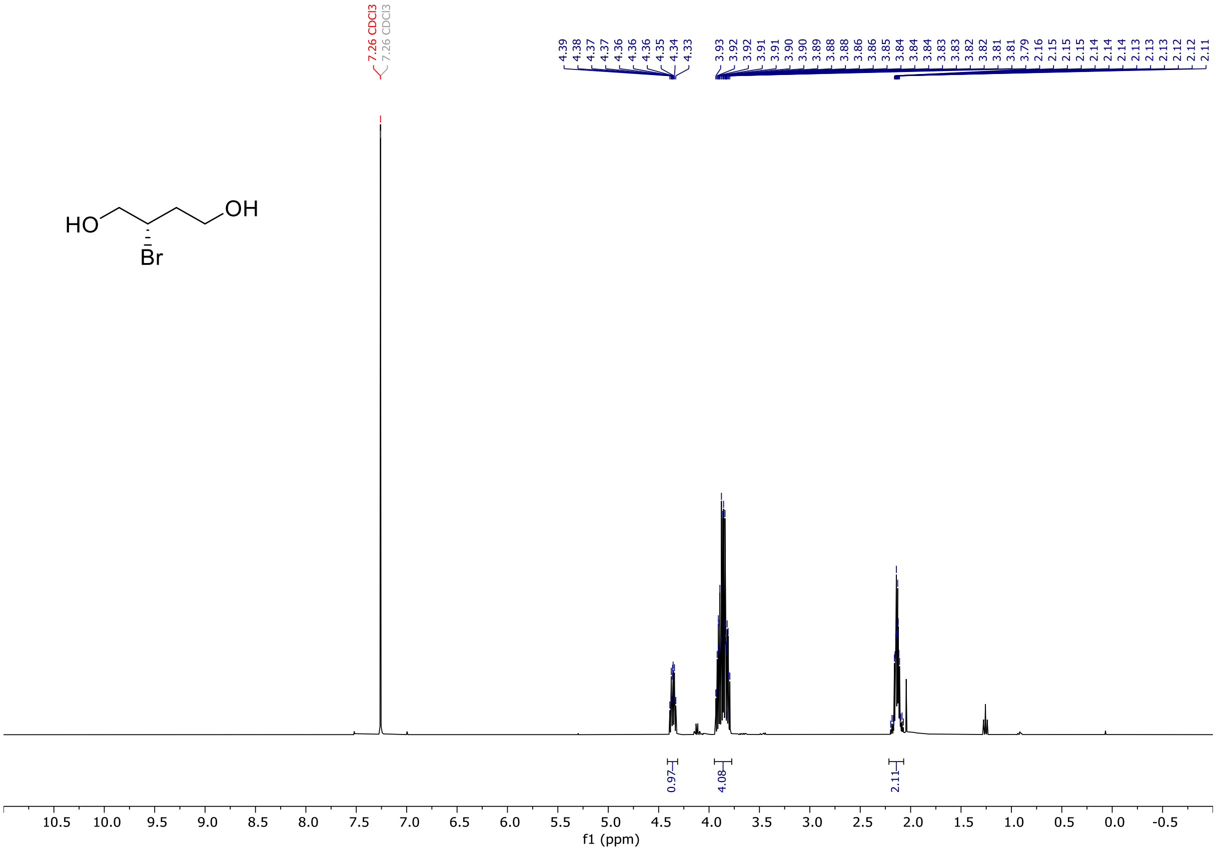

^{13}C NMR ($CDCl_3$, 101 MHz)

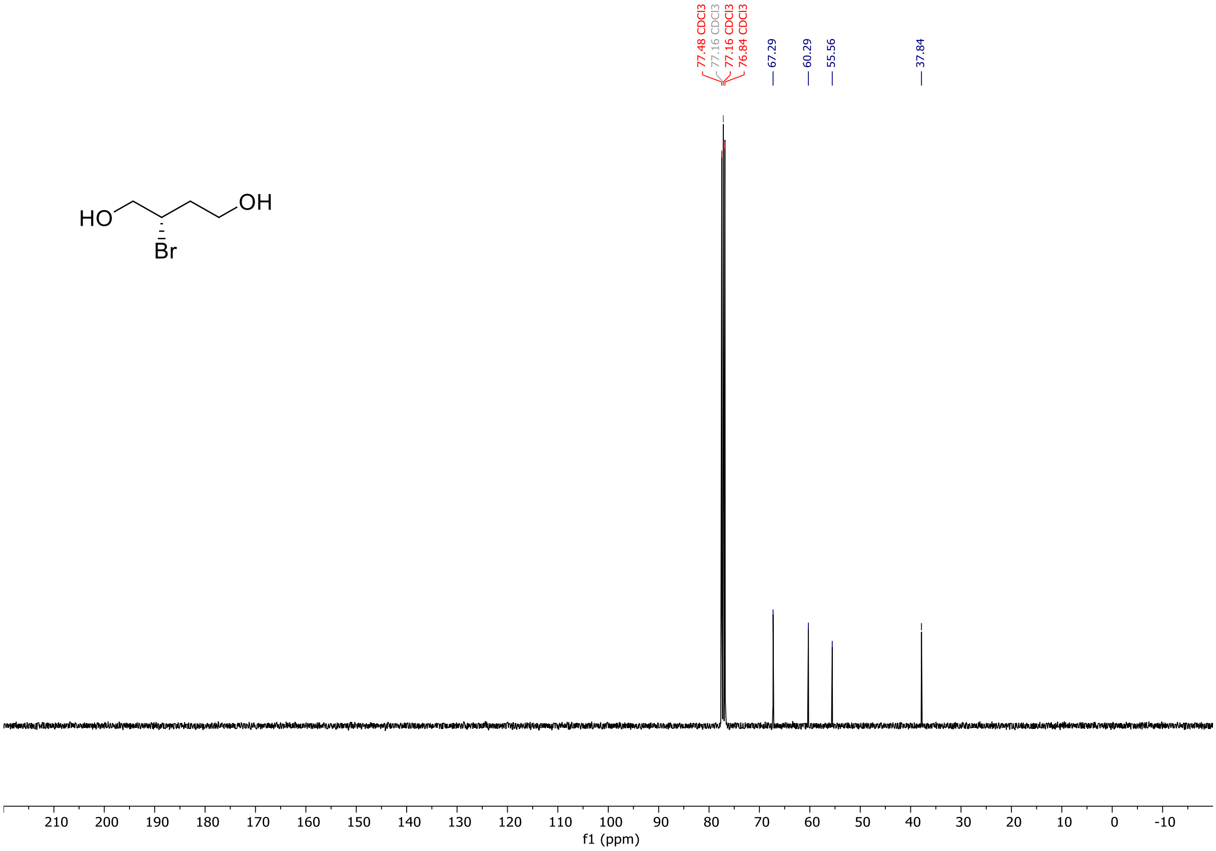

Compound 14: ^{1}H NMR ($CDCl_3$, 400 MHz)

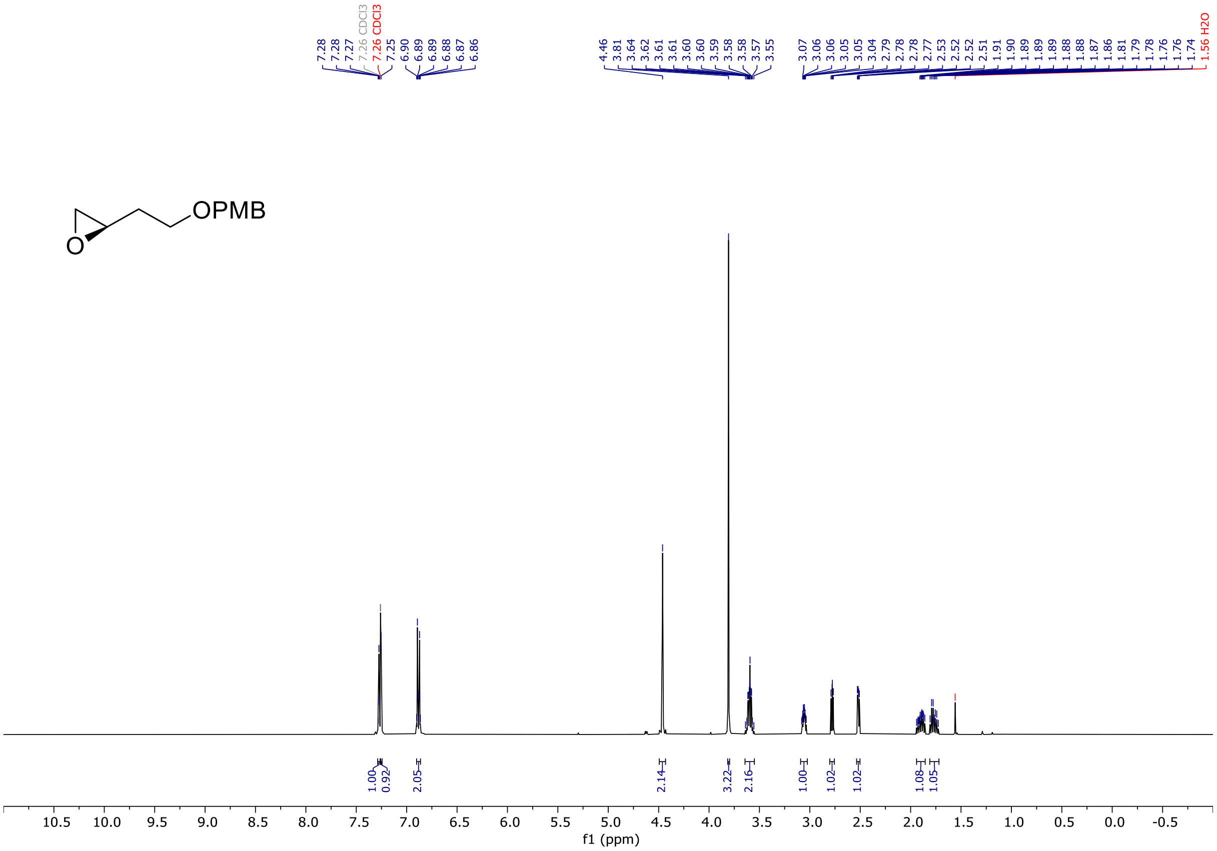

^{13}C NMR ($CDCl_3$, 101 MHz)

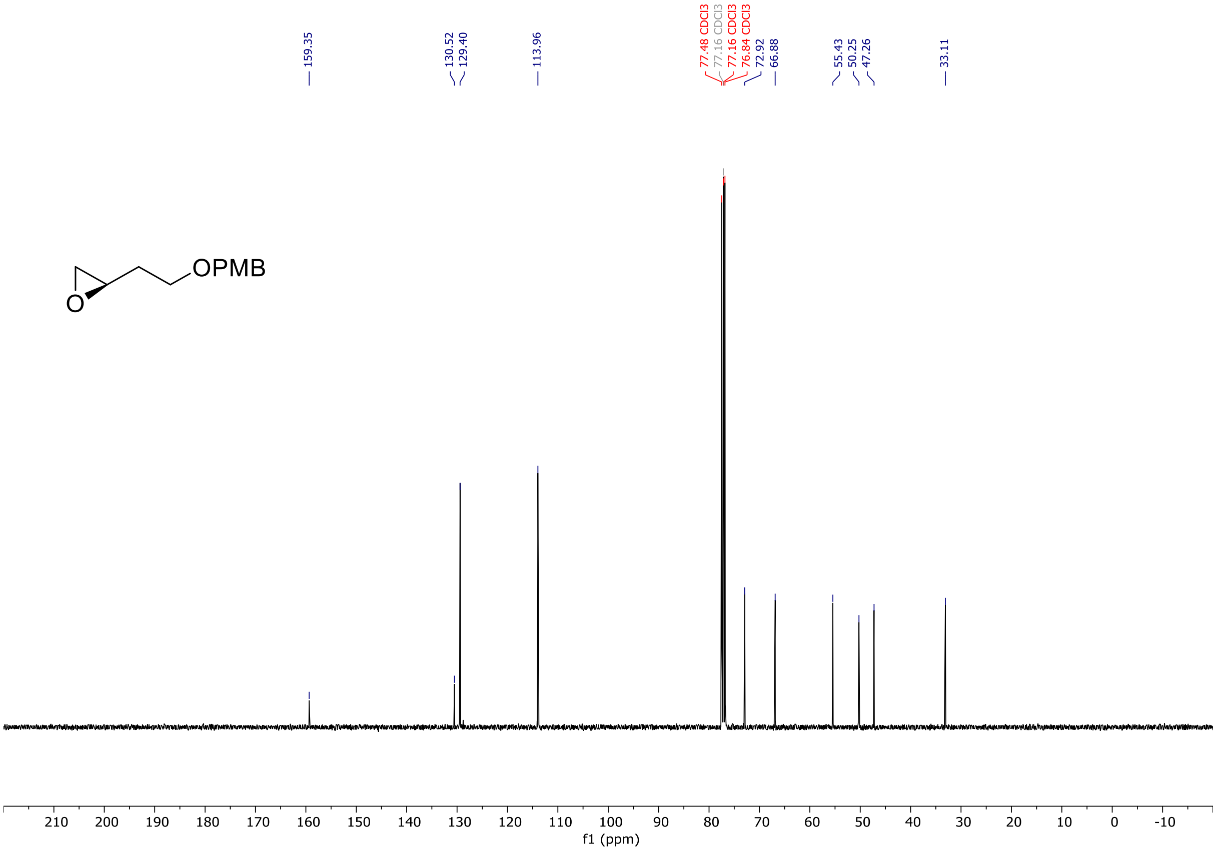

Compound 16: ^{1}H NMR (CD_2Cl_2, 400 MHz)

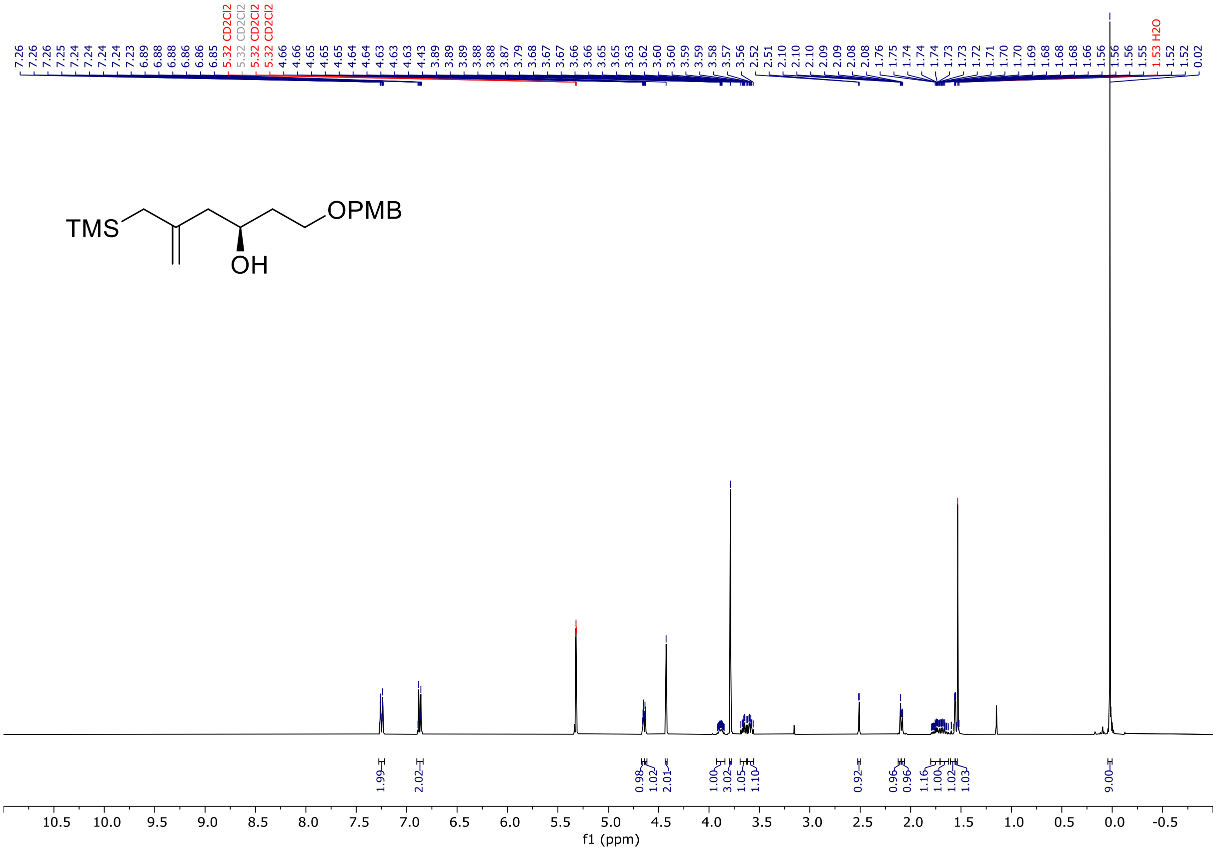

^{13}C NMR (CD_2Cl_2, 101 MHz)

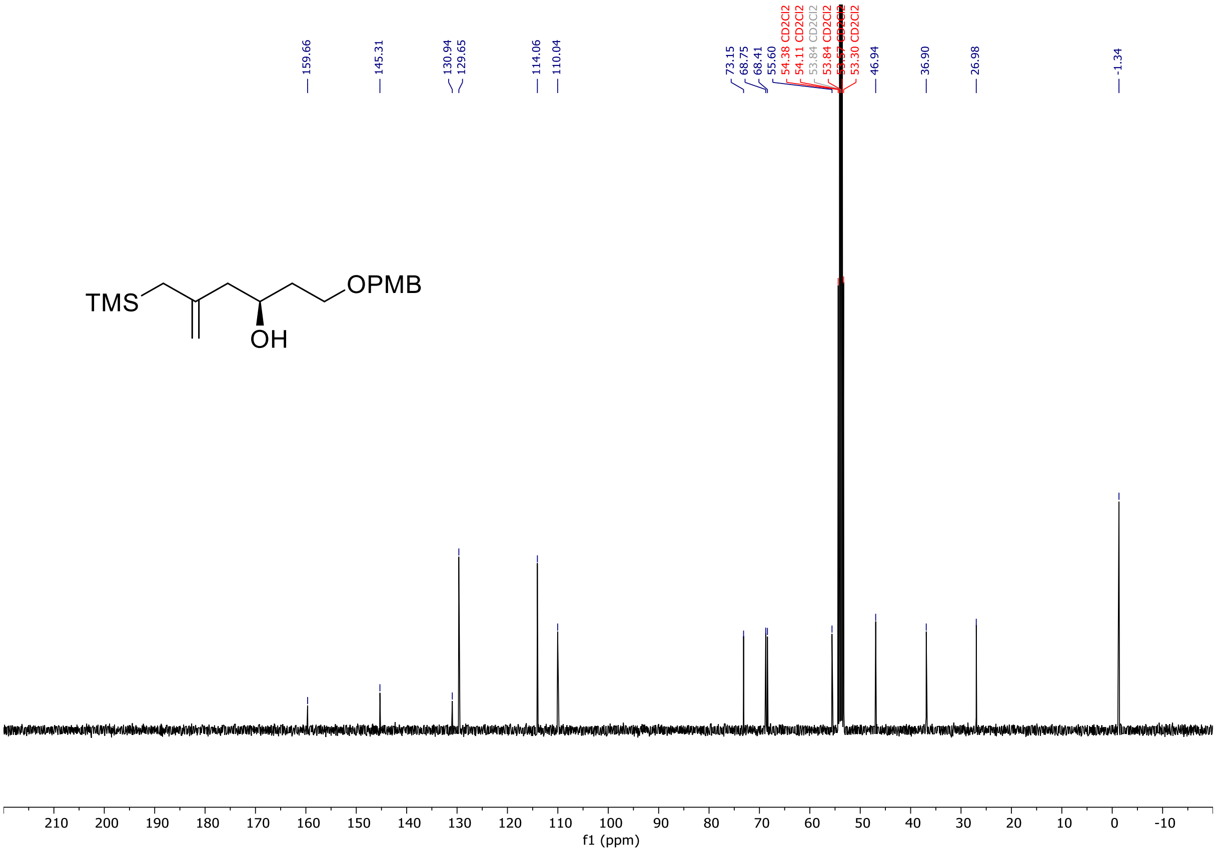

Compound 17: ^{1}H NMR (CD_2Cl_2, 400 MHz)

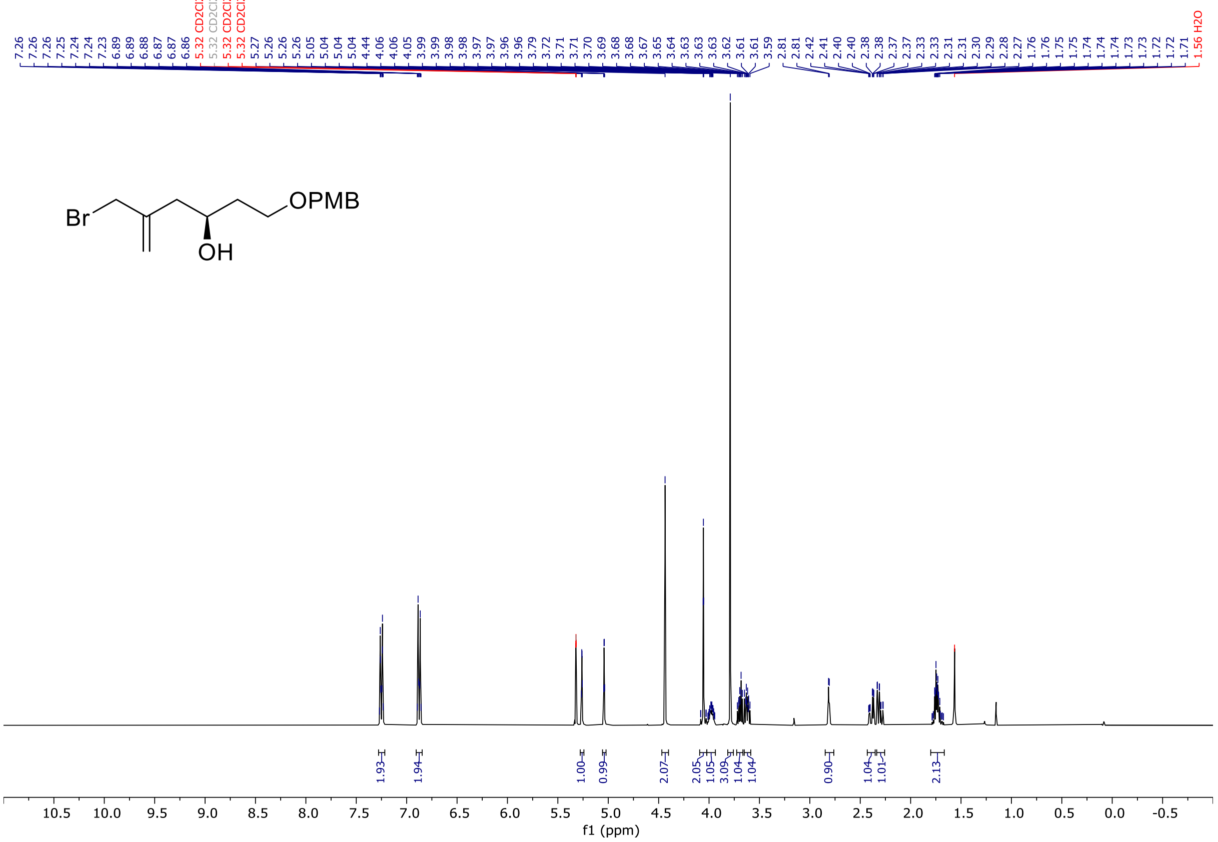

^{13}C NMR (CD_2Cl_2, 101 MHz)

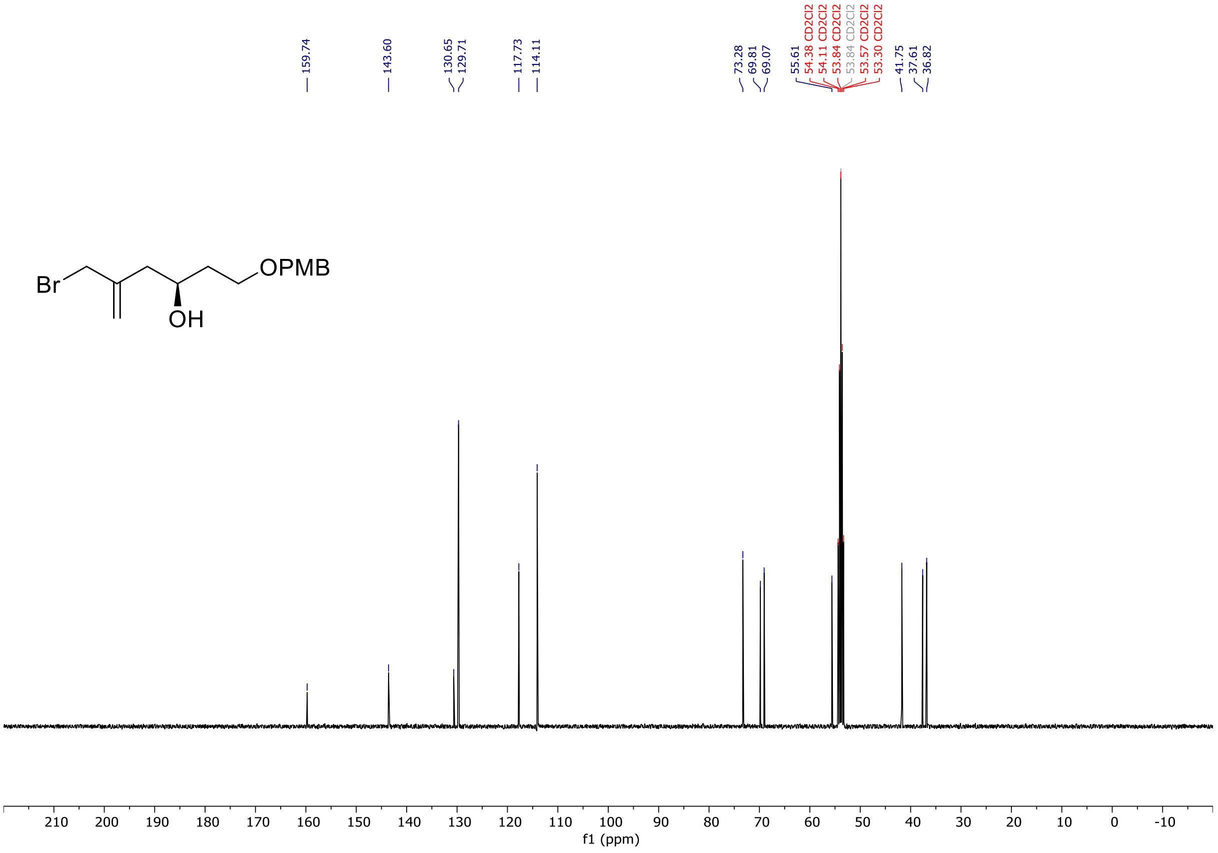

Compound S9: ^{1}H NMR ($CDCl_3$, 600 MHz)

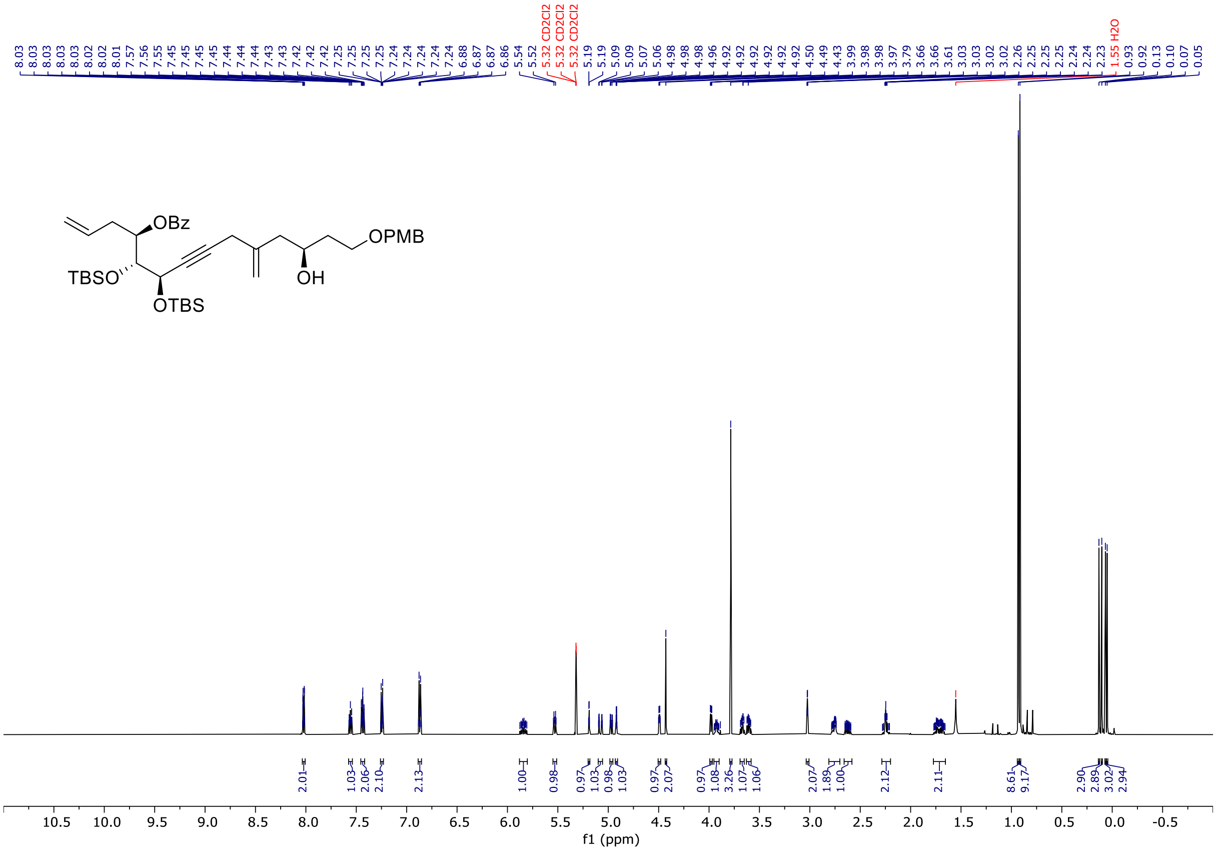

^{13}C NMR ($CDCl_3$, 151 MHz)

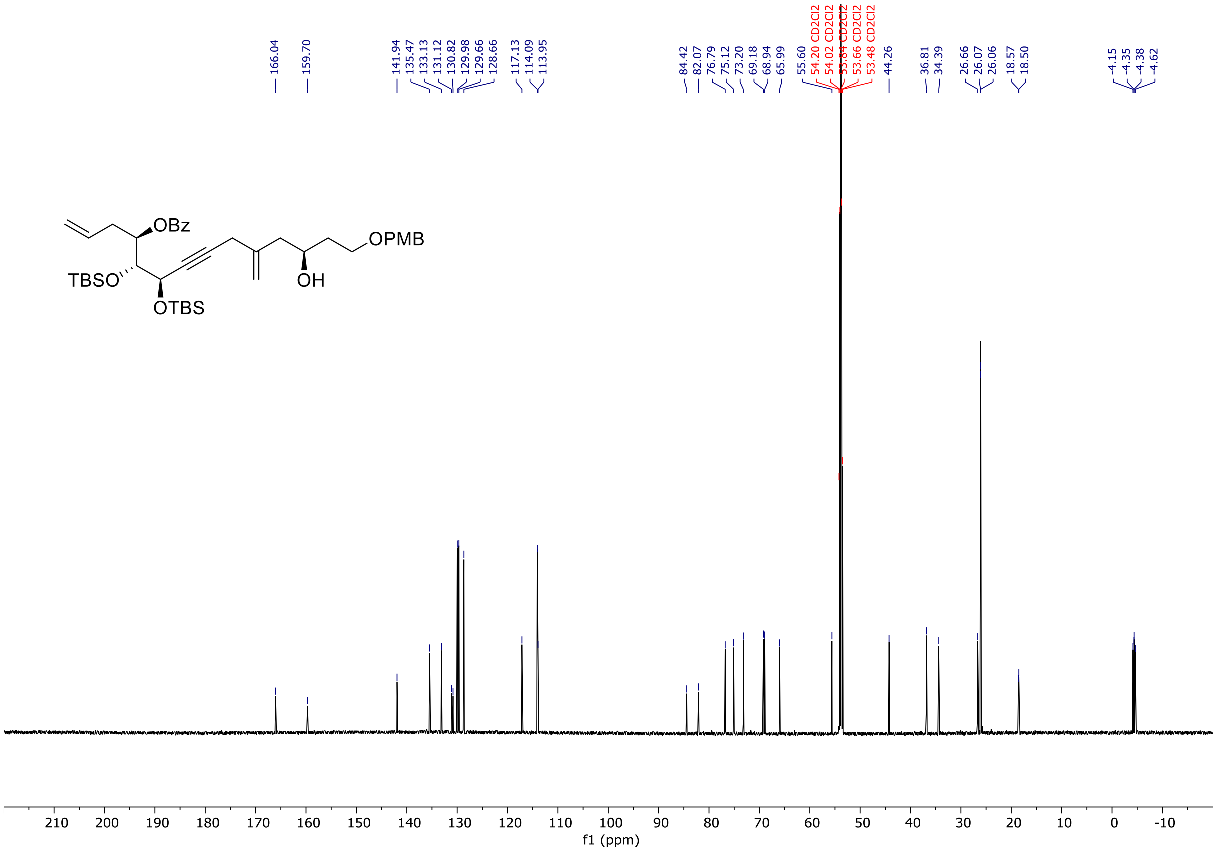

Compound 18: ^{1}H NMR ($CDCl_3$, 400 MHz)

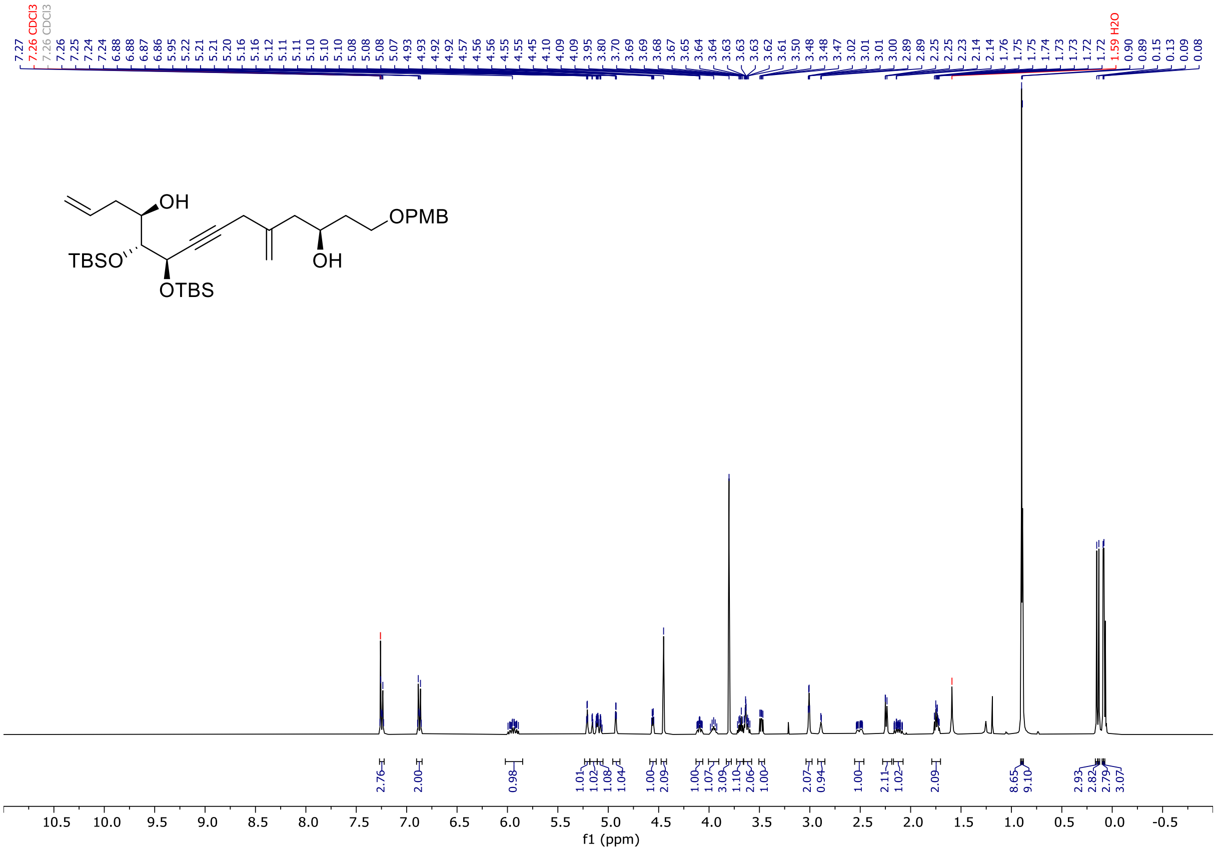

^{13}C NMR ($CDCl_3$, 101 MHz)

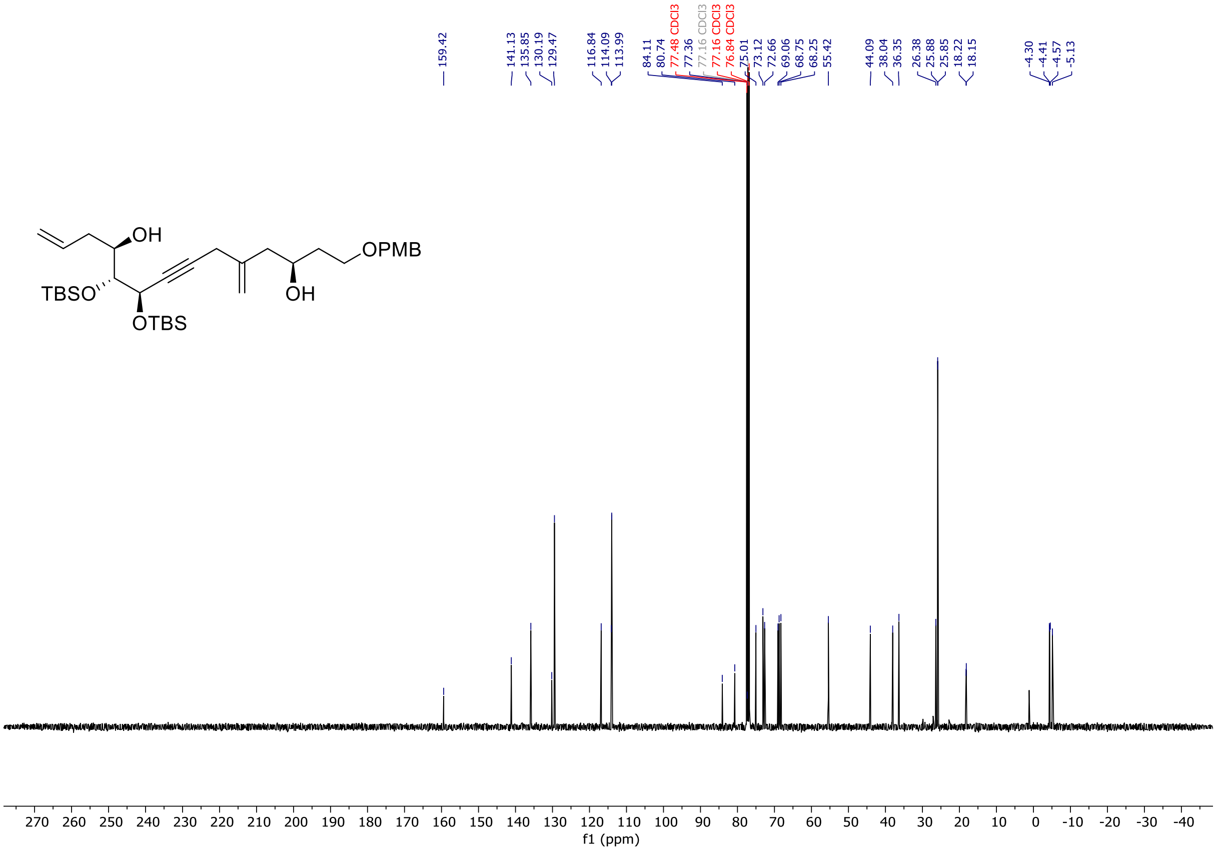

Compound 21: ^{1}H NMR ($CDCl_3$, 400 MHz)

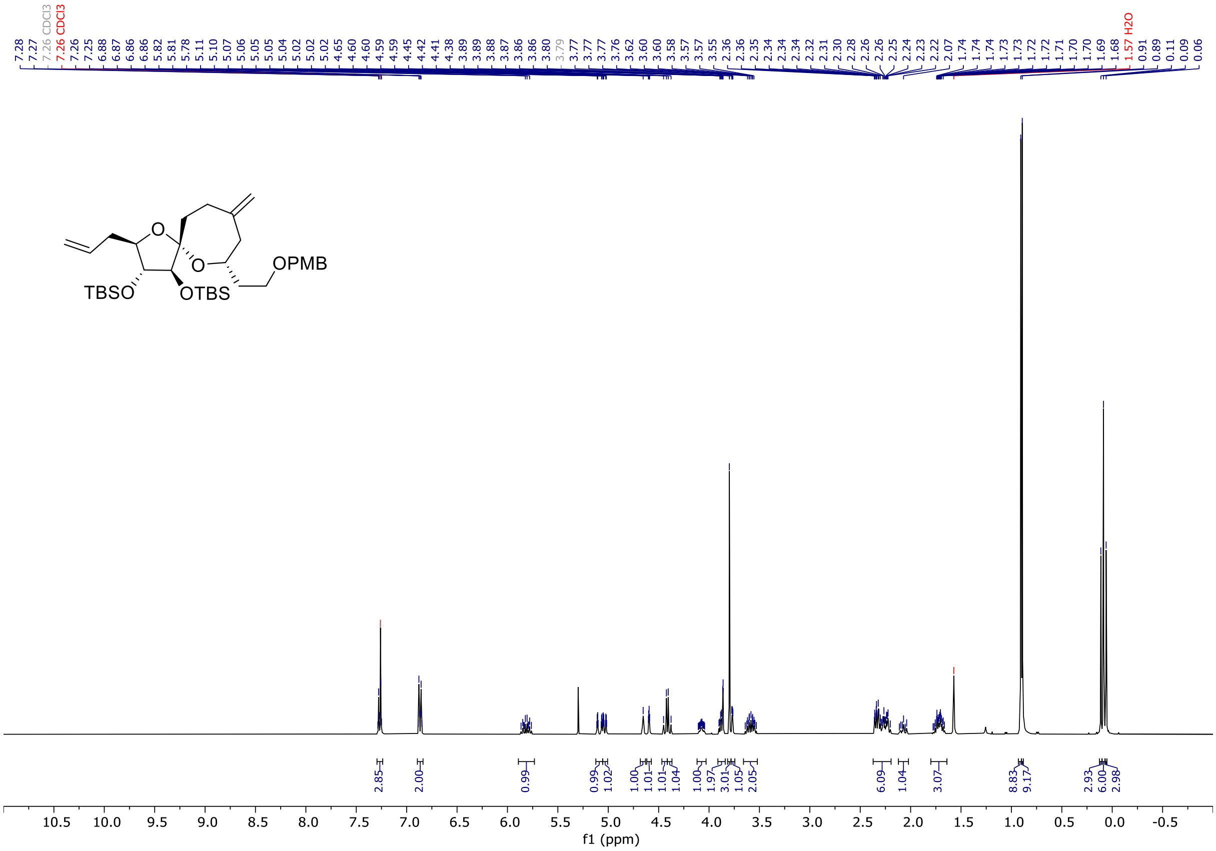

^{13}C NMR ($CDCl_3$, 101 MHz)

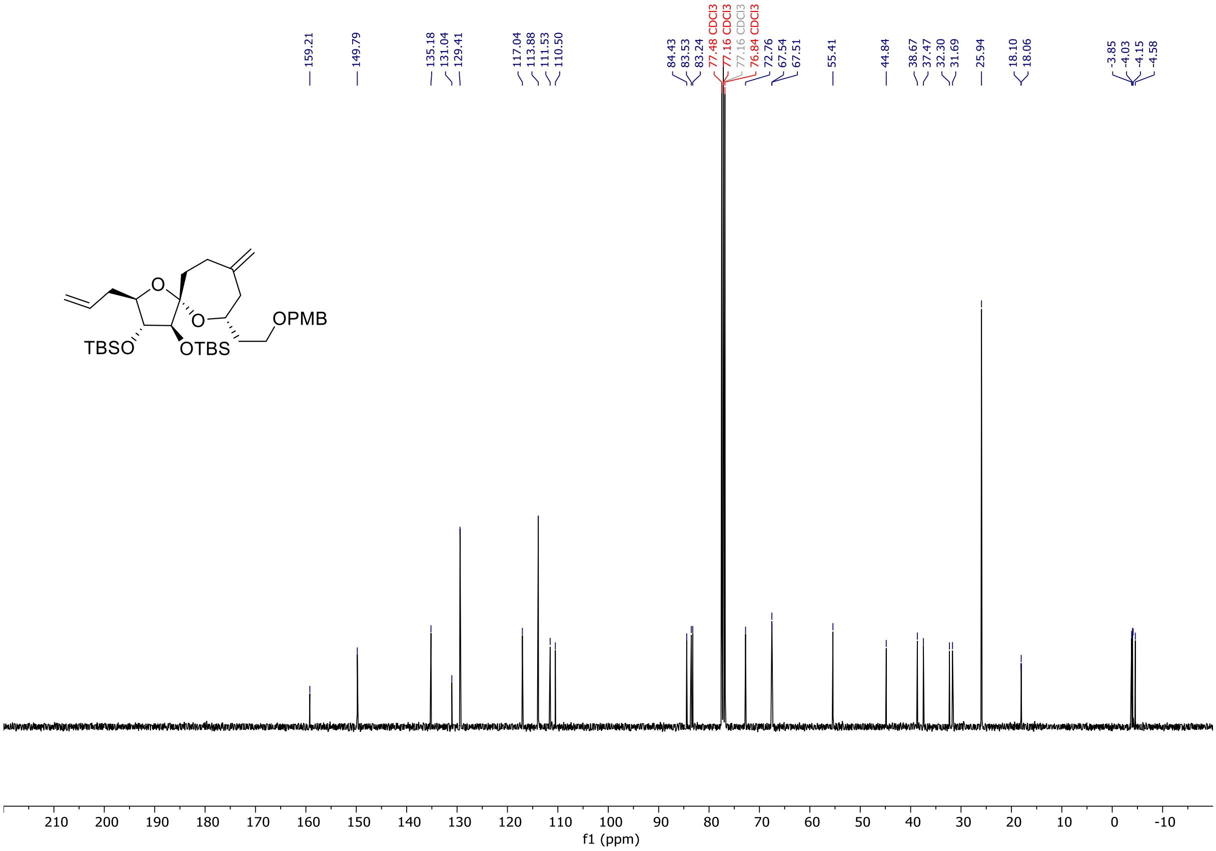

Compound 22: ^{1}H NMR ($CDCl_3$, 600 MHz)

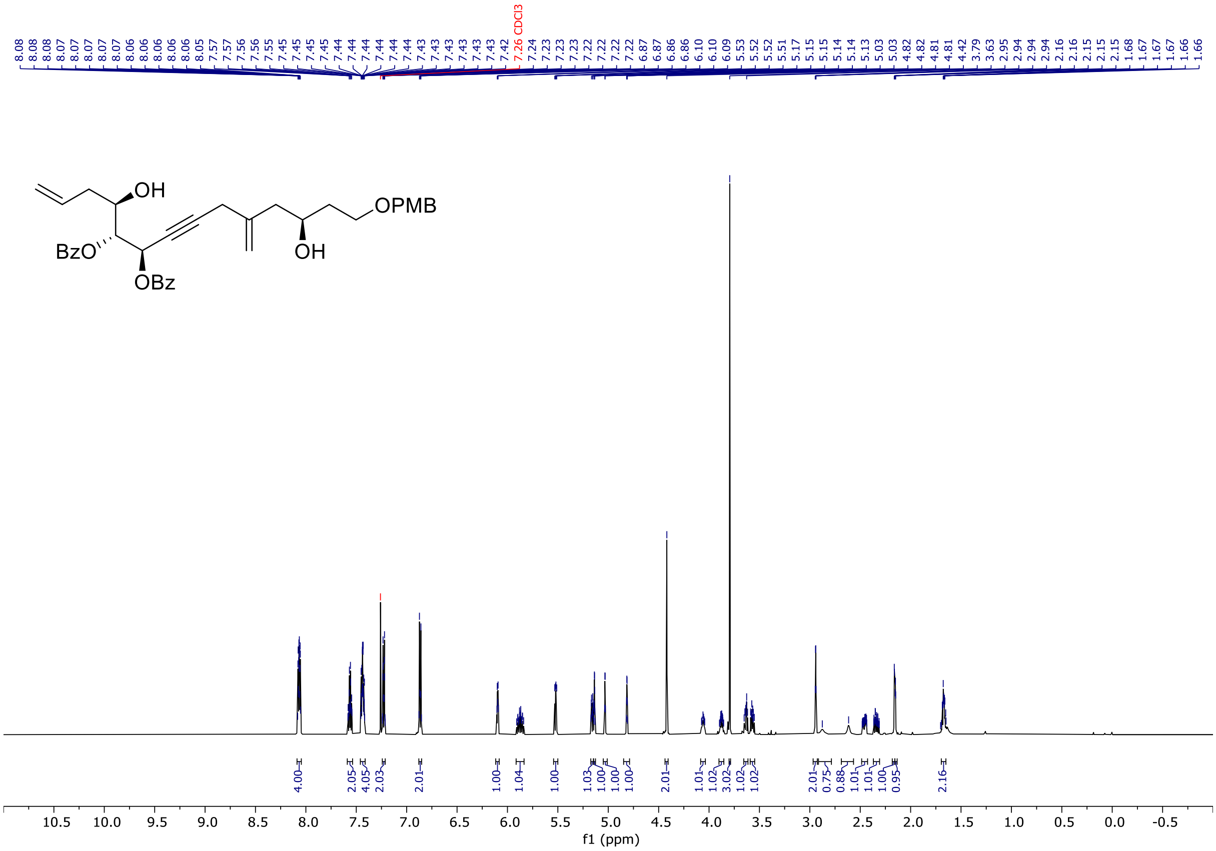

^{13}C NMR ($CDCl_3$, 151 MHz)

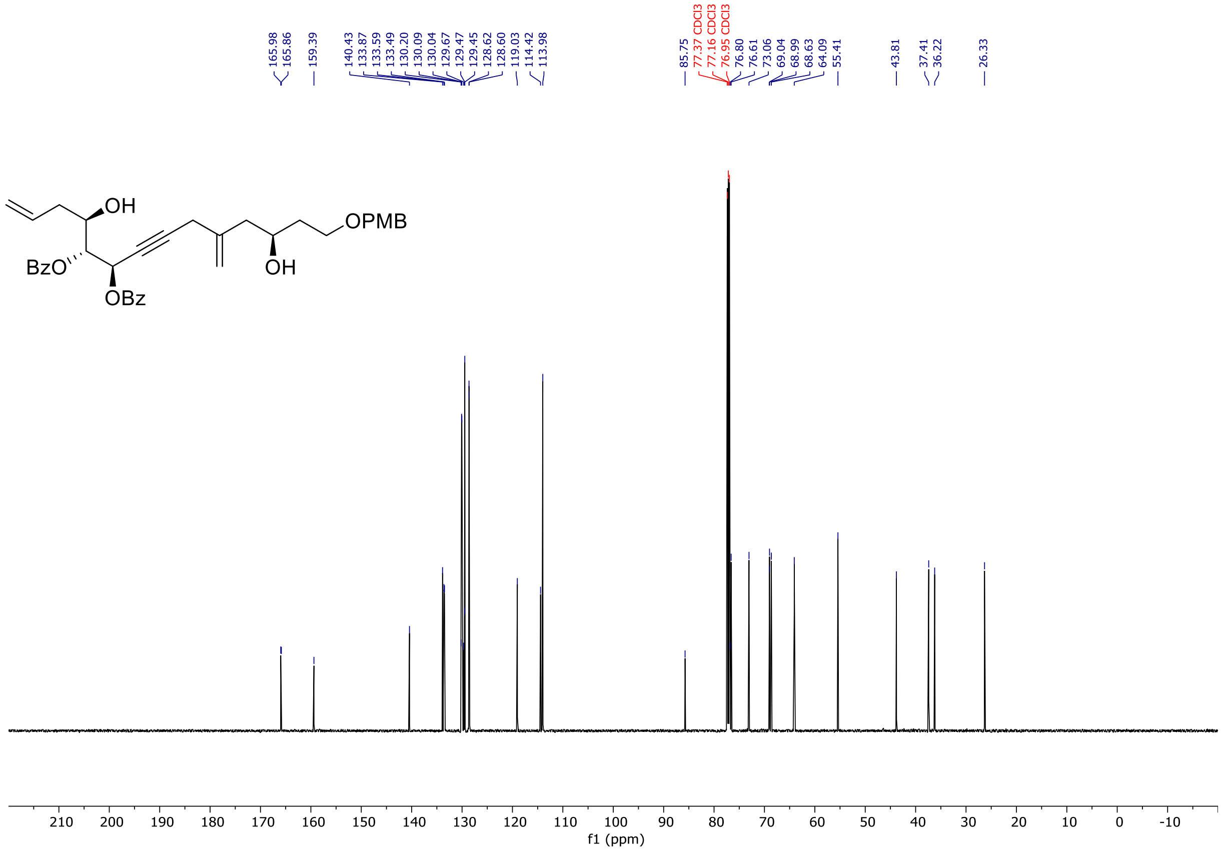

Compound 23: ^{1}H NMR ($CDCl_3$, 600 MHz)

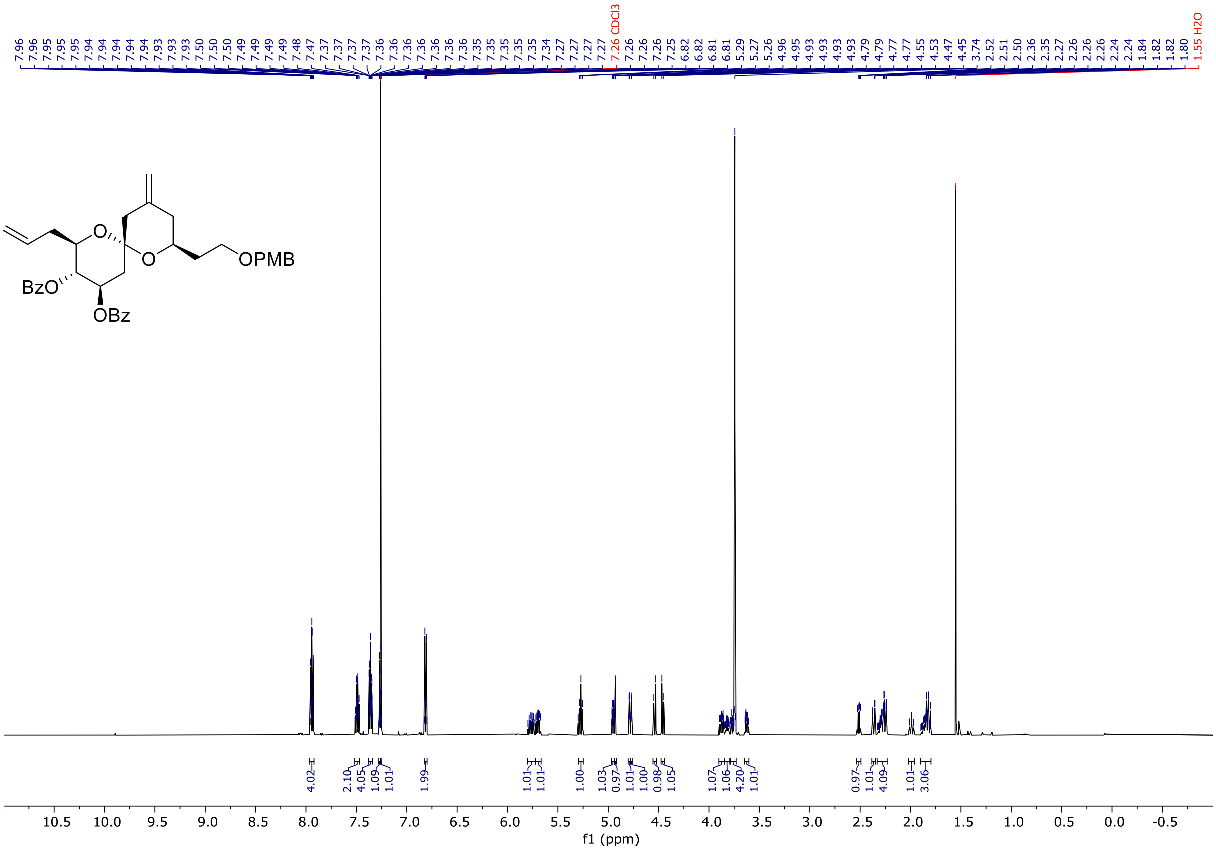

^{13}C NMR ($CDCl_3$, 151 MHz)

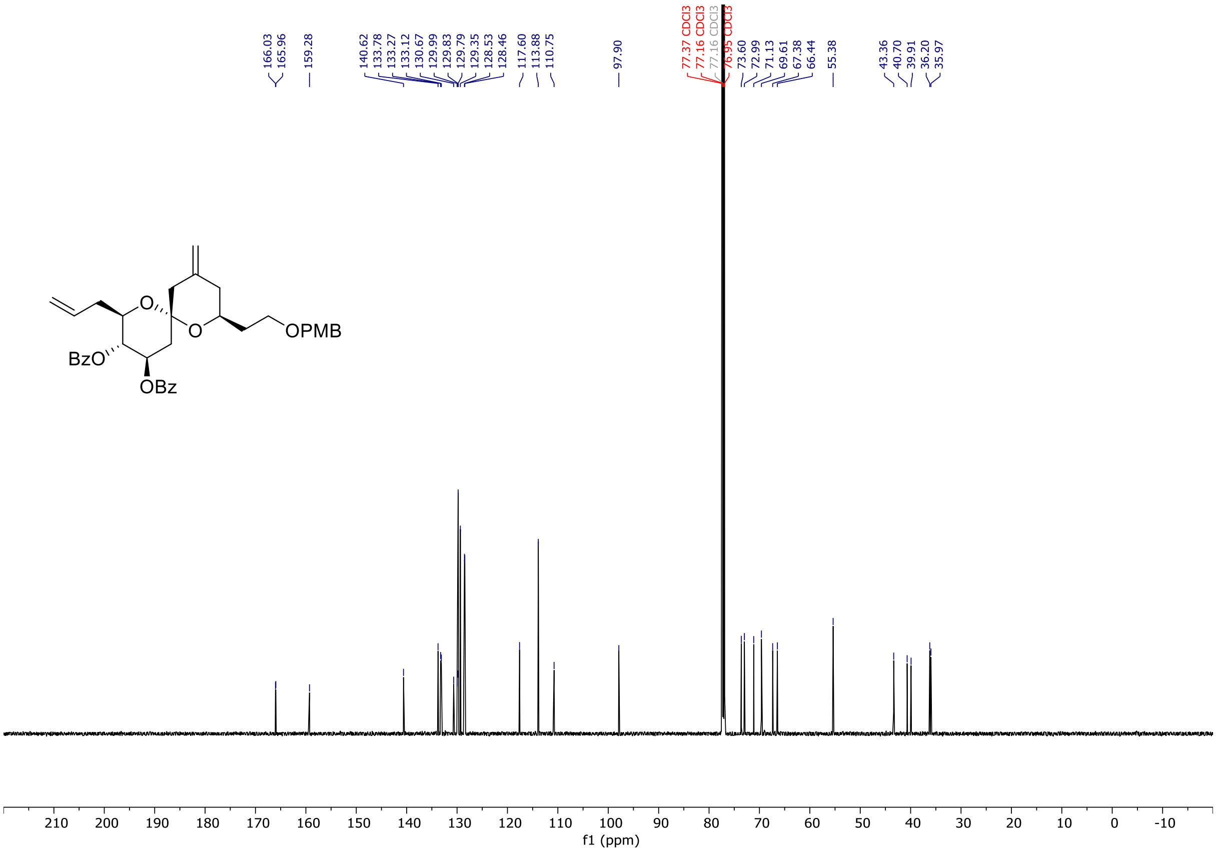

Compound 23: NOESY spectrum ($CDCl_3$, 600 MHz)

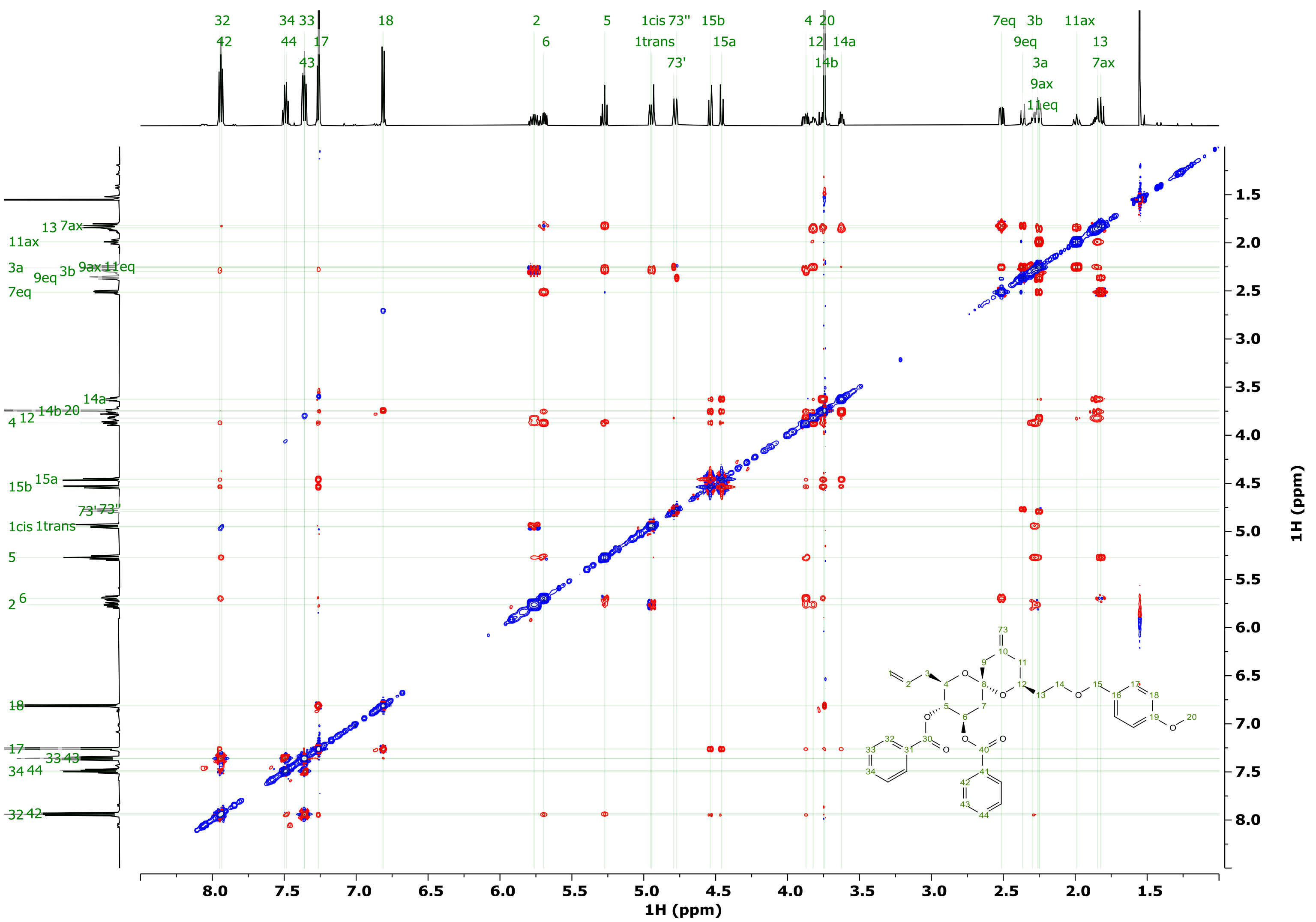

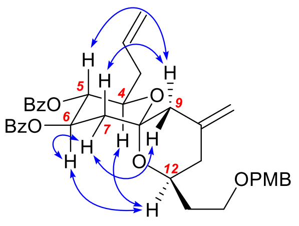

Compound 8-*epi*-23: ^1H NMR ($CDCl_3$, 400 MHz)

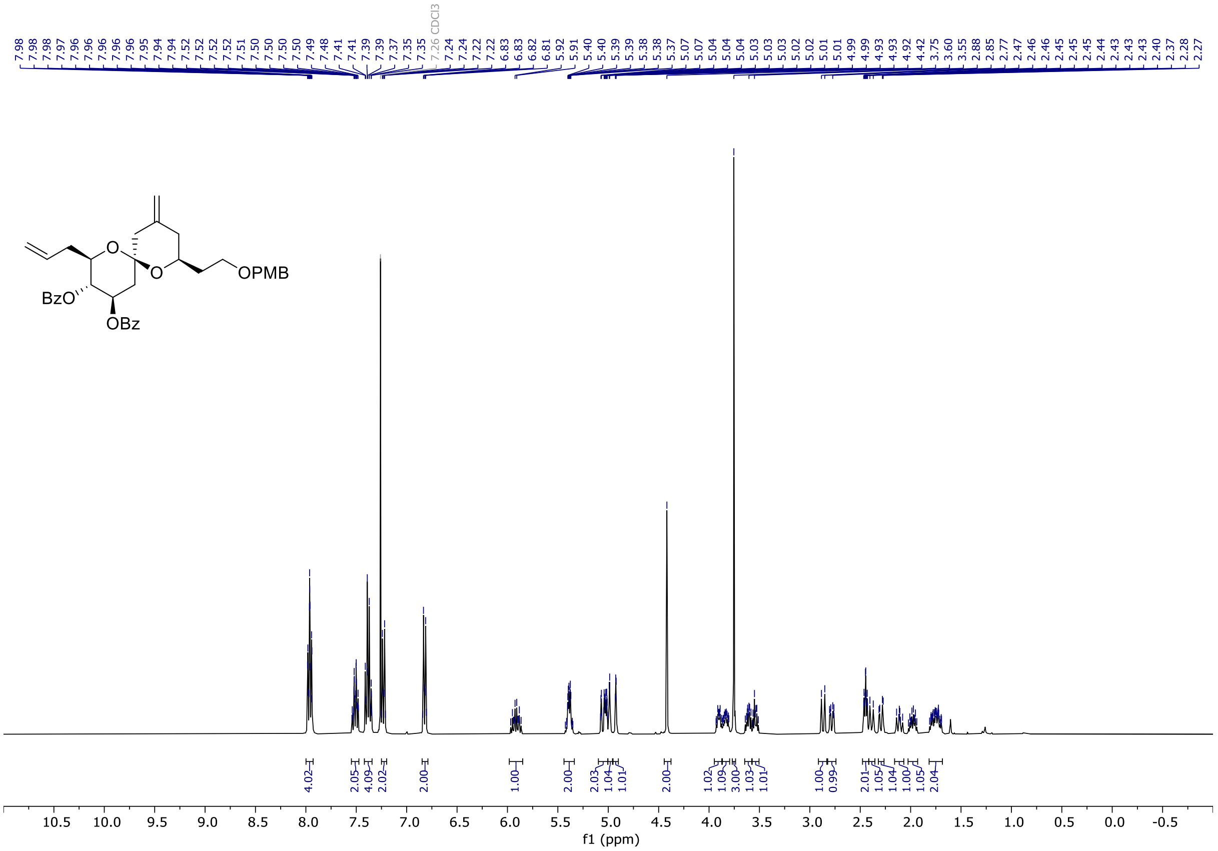

^{13}C NMR ($CDCl_3$, 101 MHz)

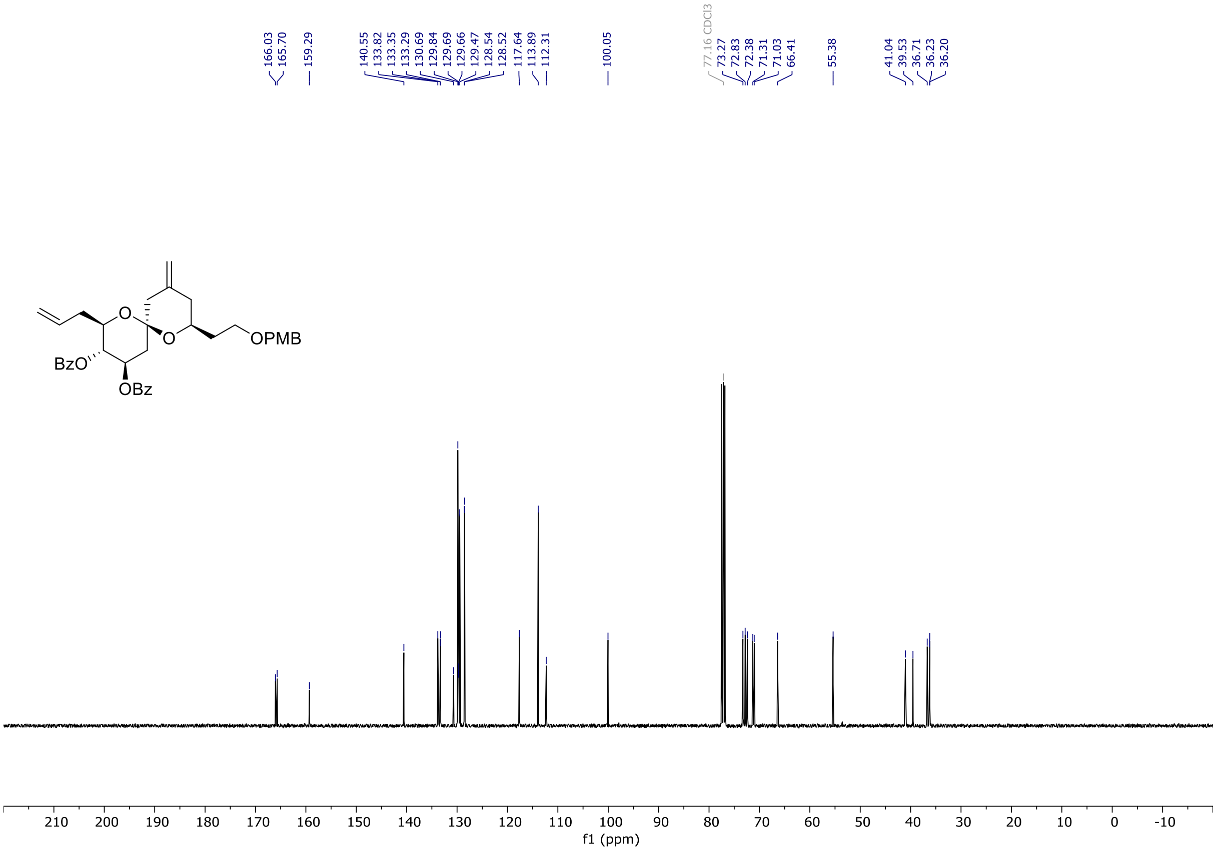

Compound 24: ^{1}H NMR (CDCl$_3$, 400 MHz)

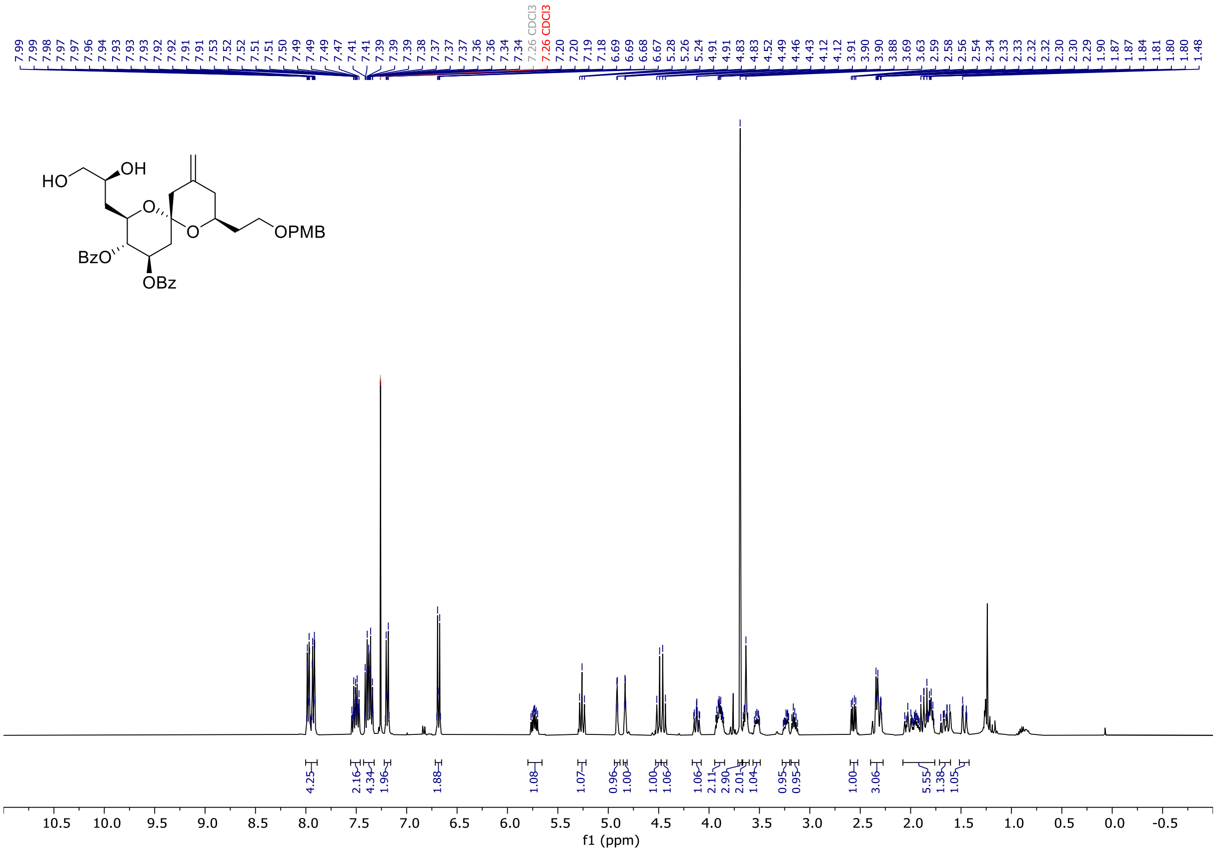

^{13}C NMR (CDCl$_3$, 101 MHz)

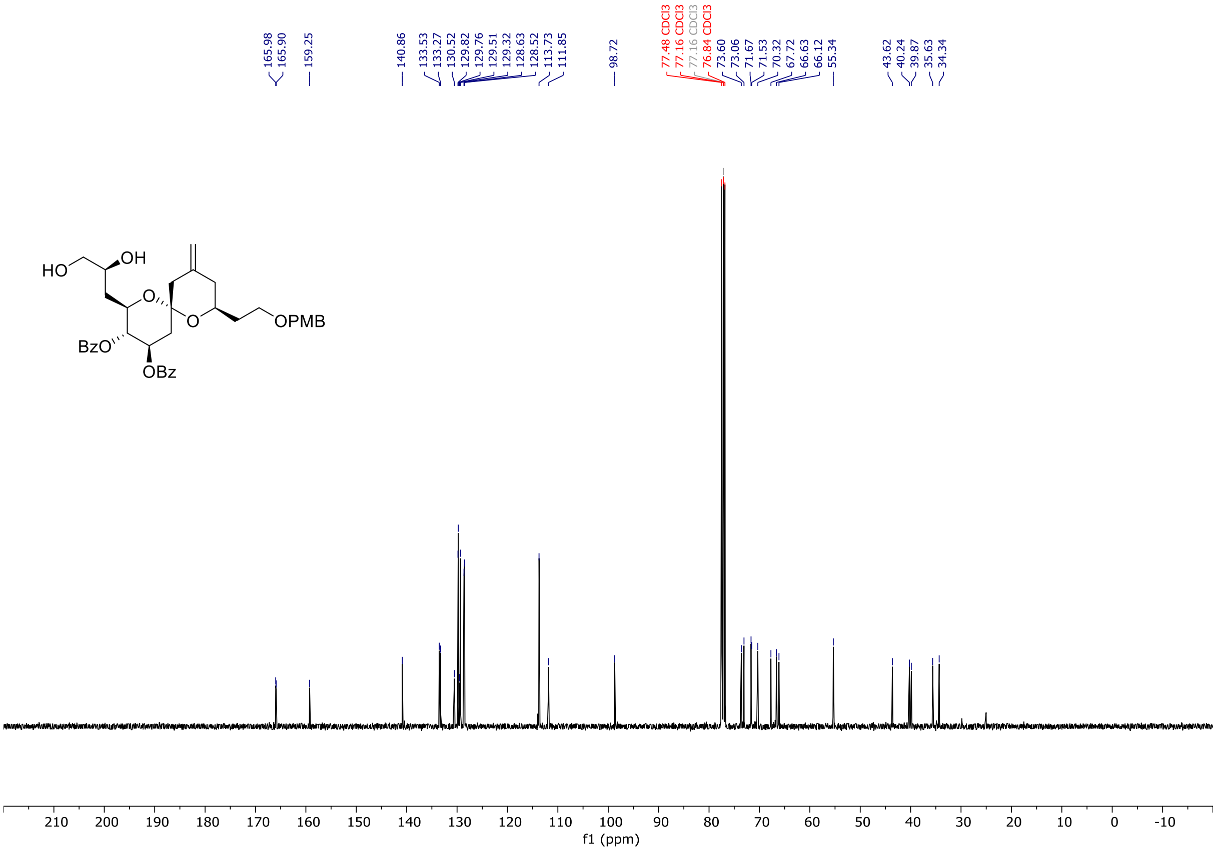

Mosher Esters derived from compound 24: (*R*)-MTPA ester: ^{1}H NMR ($CDCl_3$, 600 MHz)

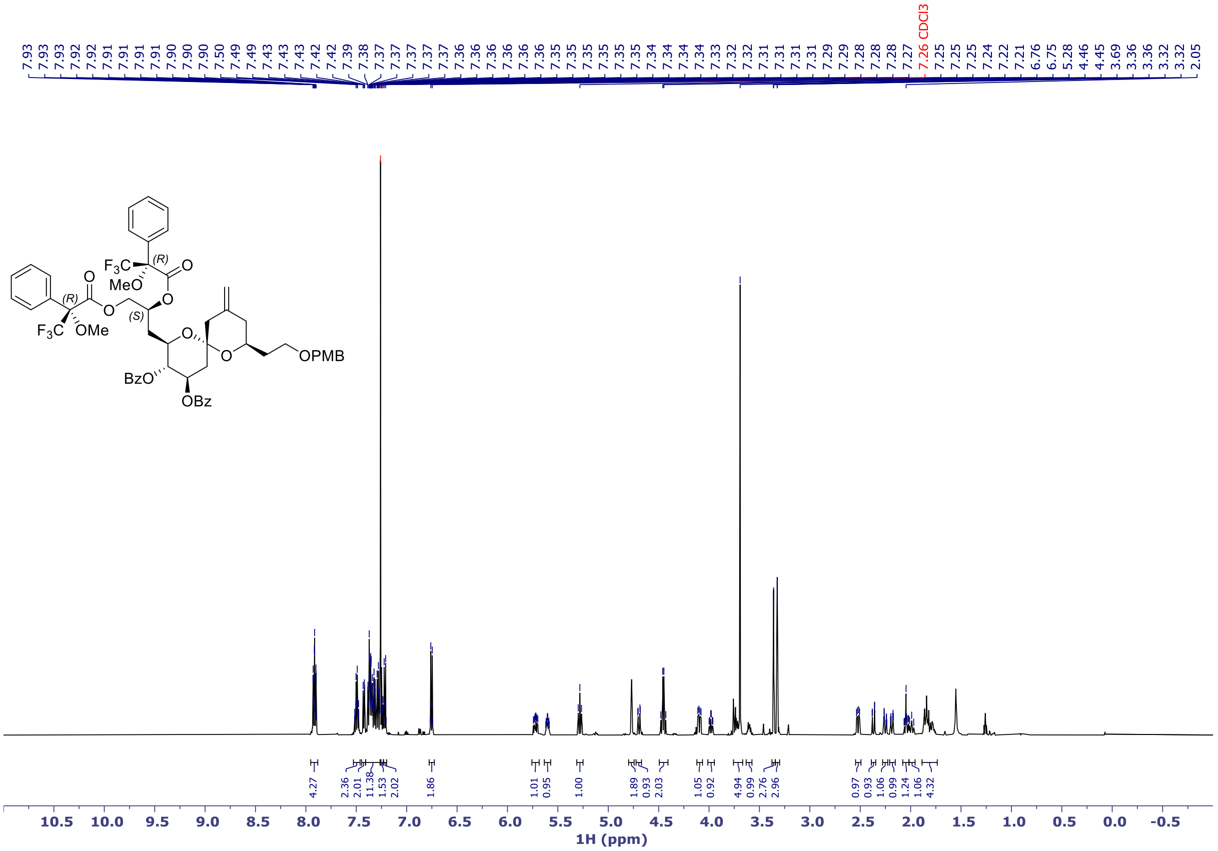

(*S*)-MTPA ester: ^{1}H NMR ($CDCl_3$, 600 MHz)

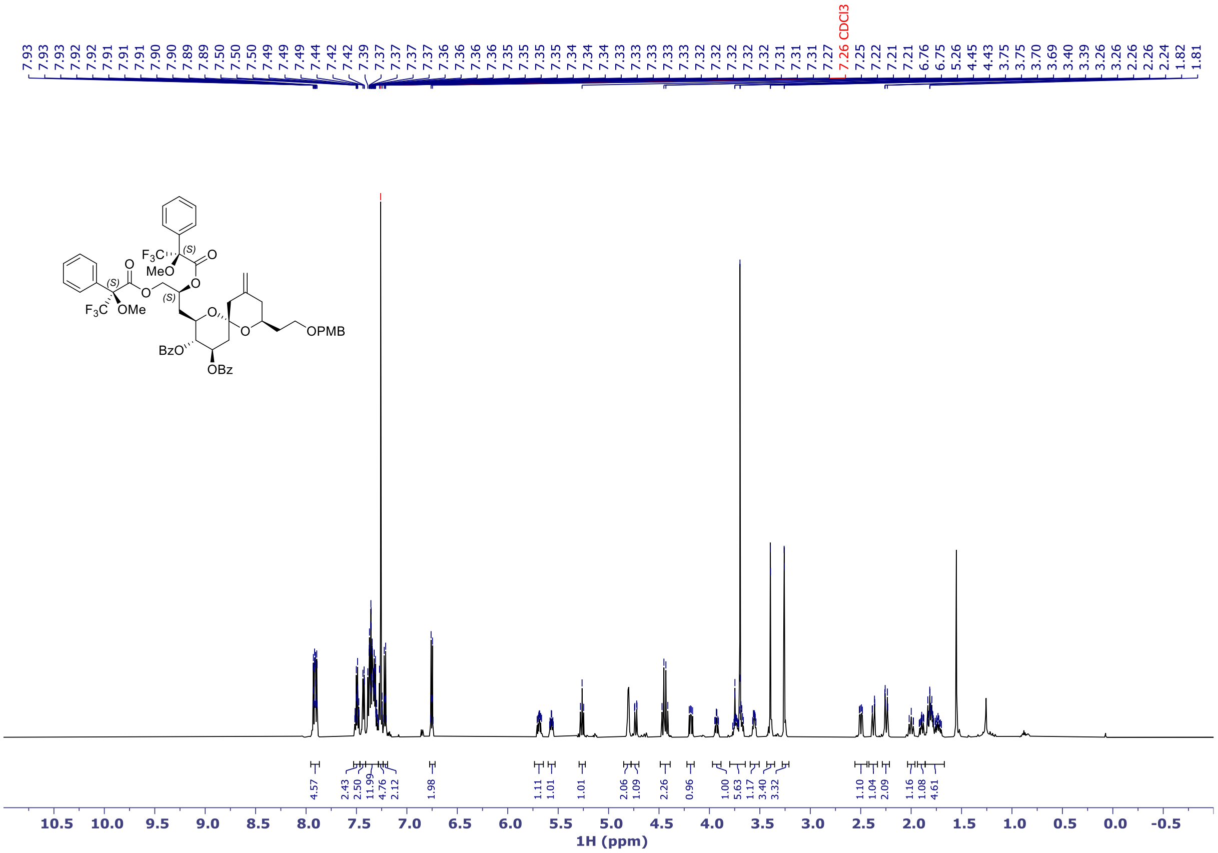

Compound S10a: ^{1}H NMR ($CDCl_3$, 400 MHz)

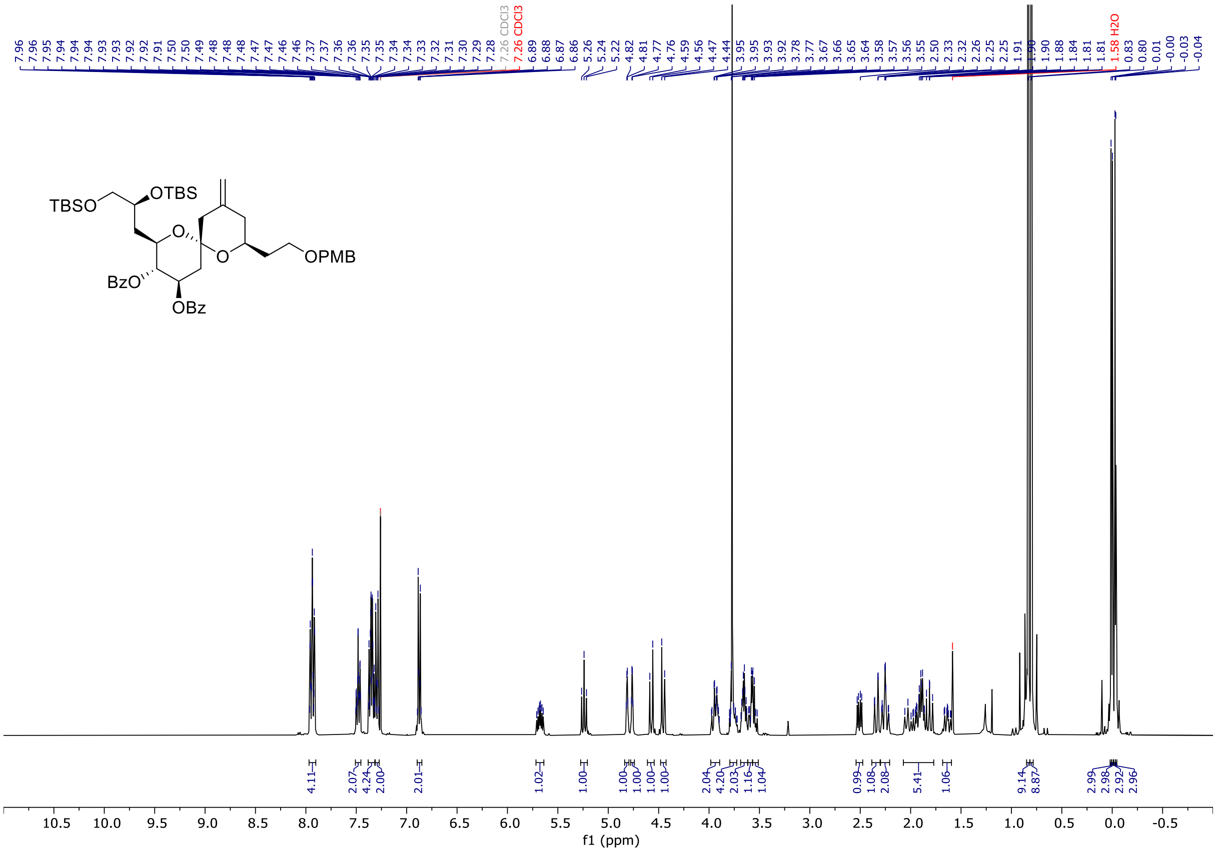

^{13}C NMR ($CDCl_3$, 101 MHz)

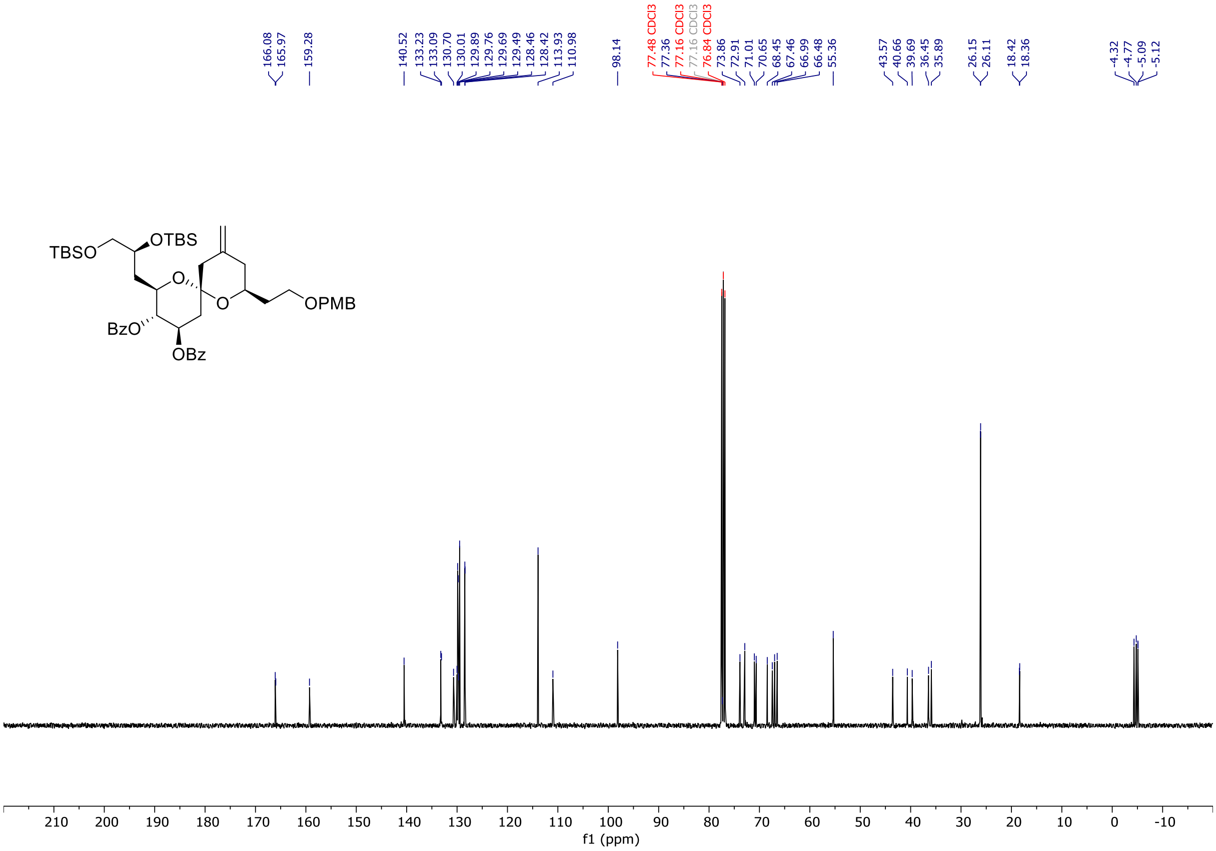

Compound S10: ^{1}H NMR ($CDCl_3$, 400 MHz)

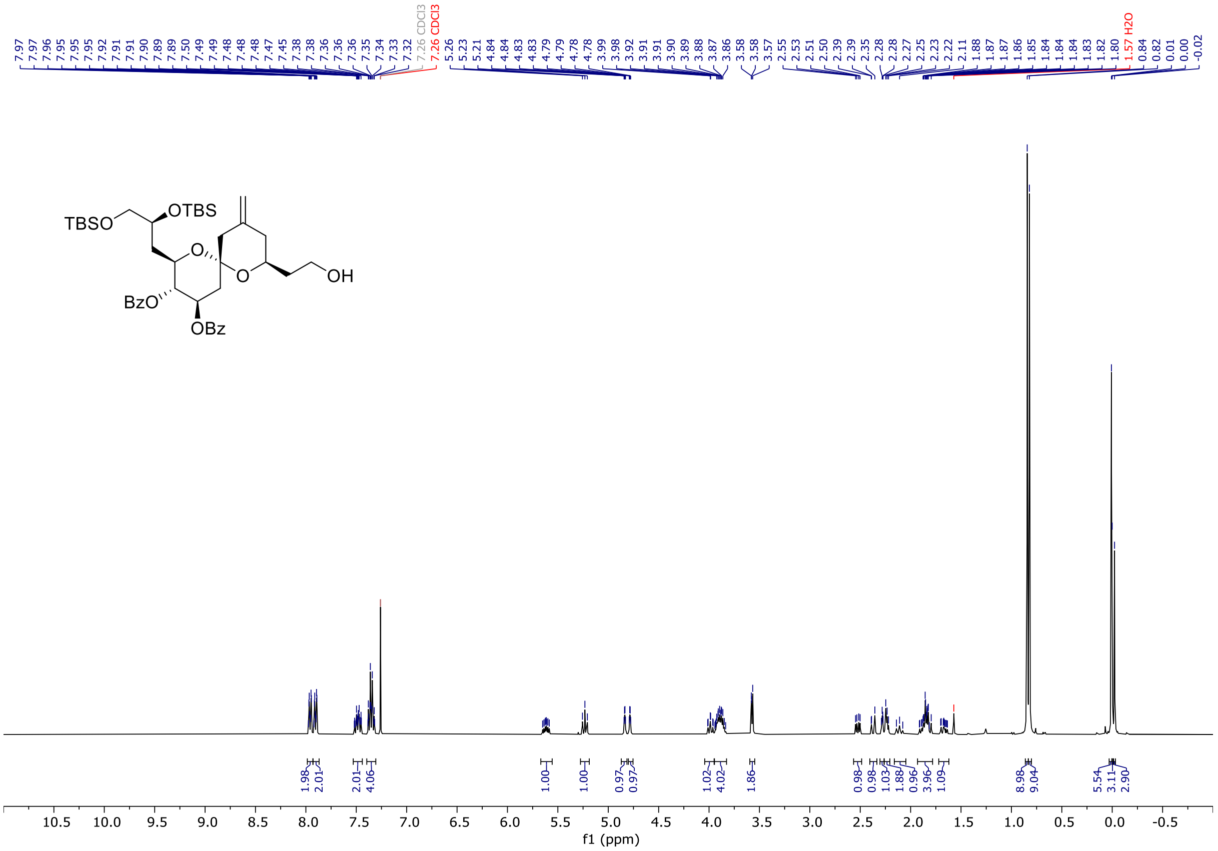

^{13}C NMR ($CDCl_3$, 101 MHz)

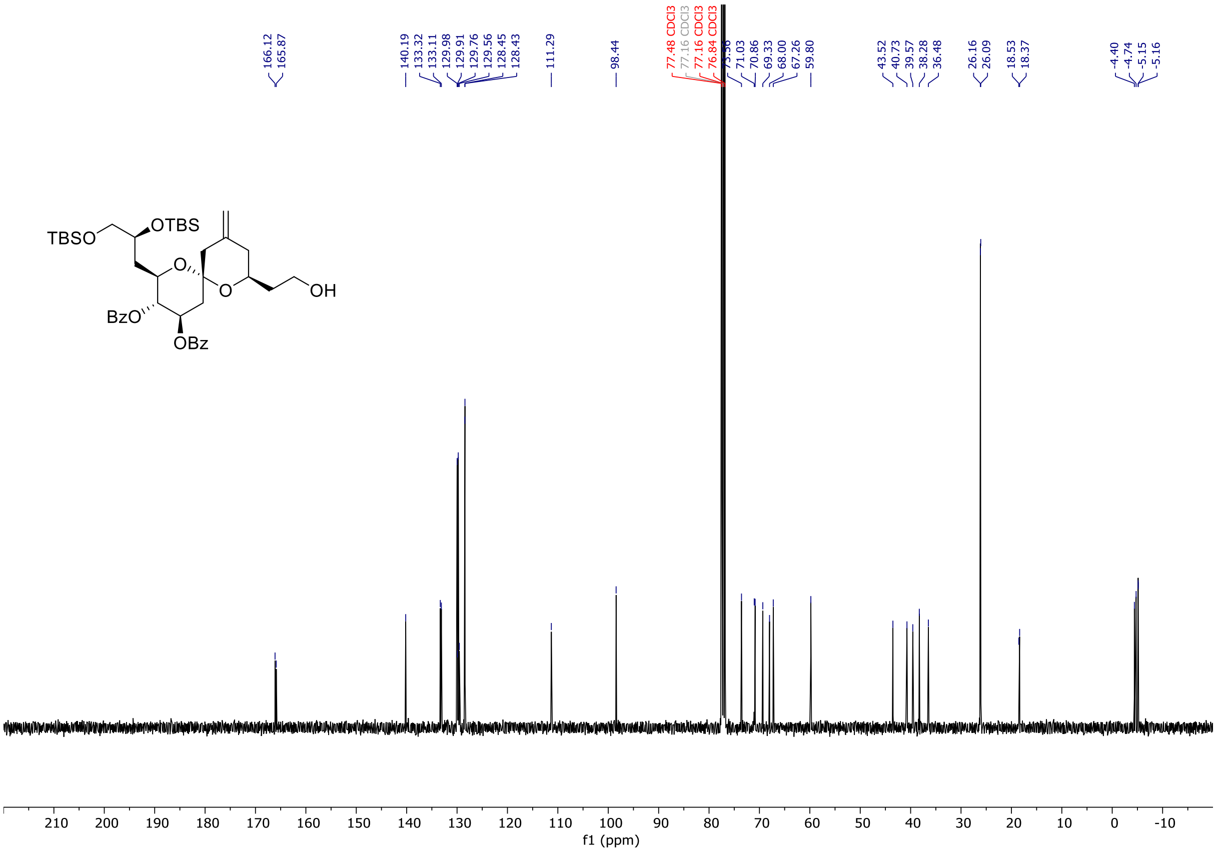

Compound 25: ^{1}H NMR (CD_2Cl_2, 400 MHz)

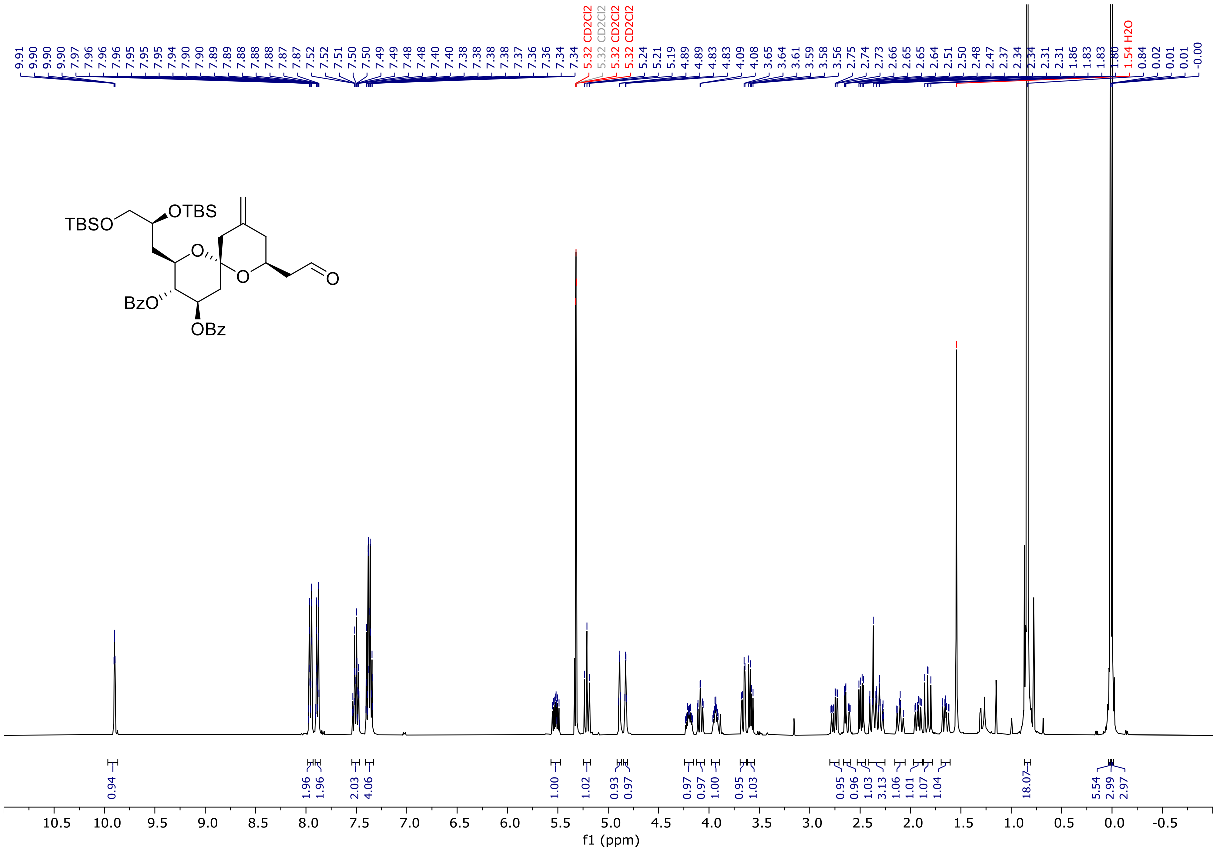

^{13}C NMR (CD_2Cl_2, 101 MHz)

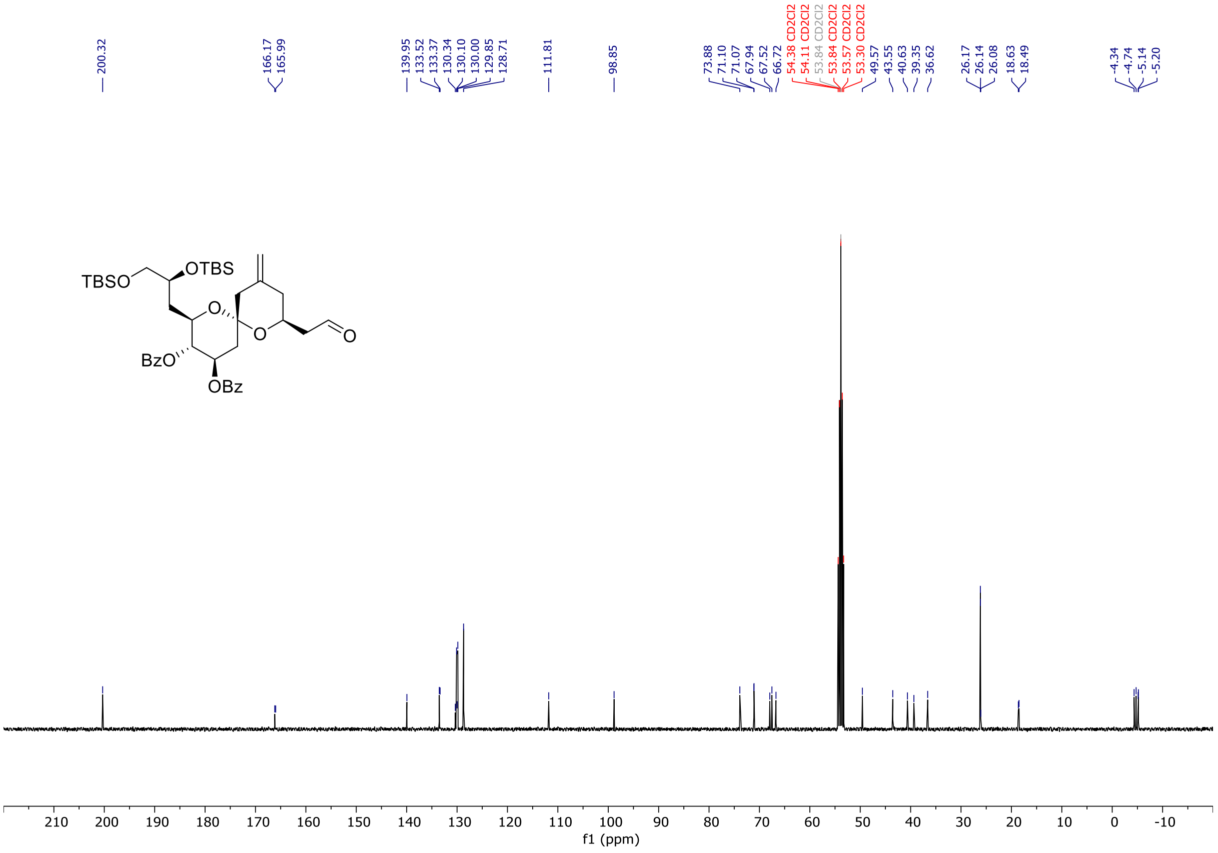

Compound 28: ^{1}H NMR (CD_2Cl_2, 400 MHz)

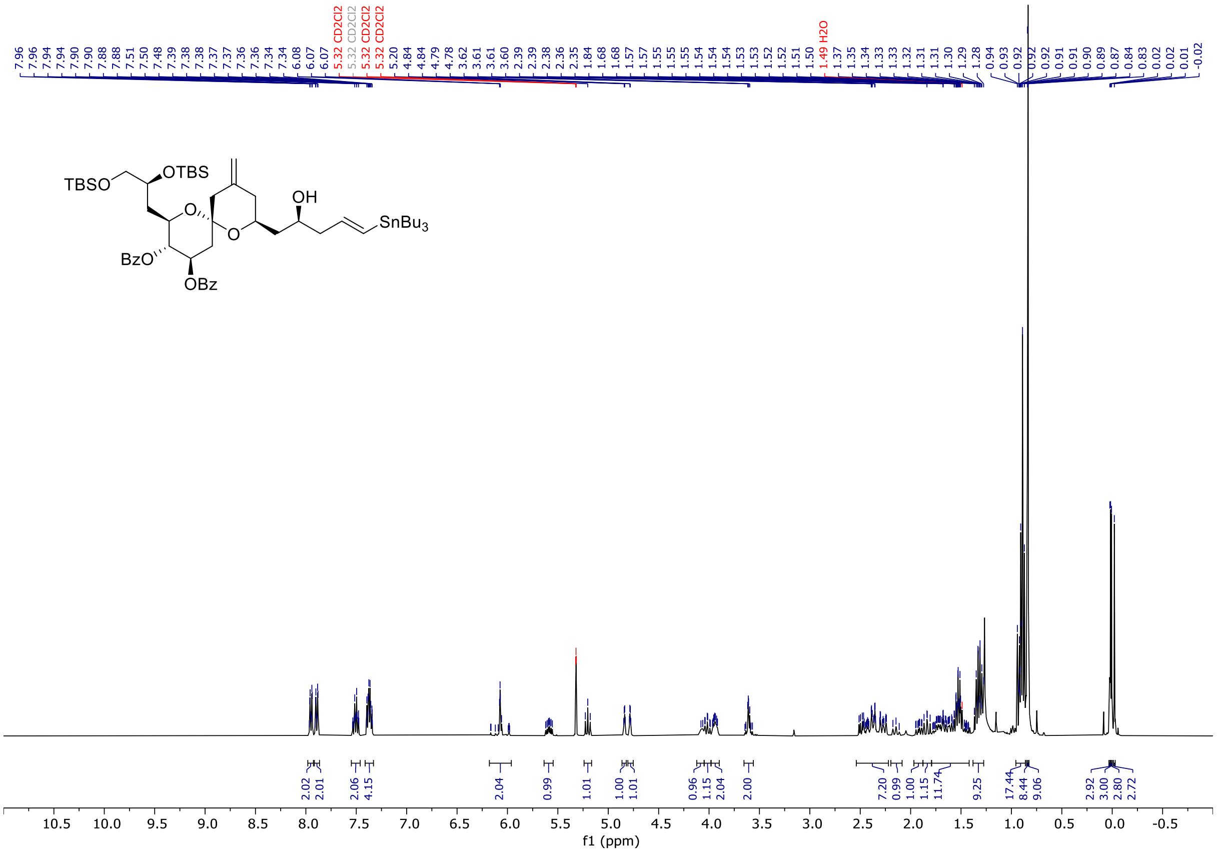

^{13}C NMR (CD_2Cl_2, 101 MHz)

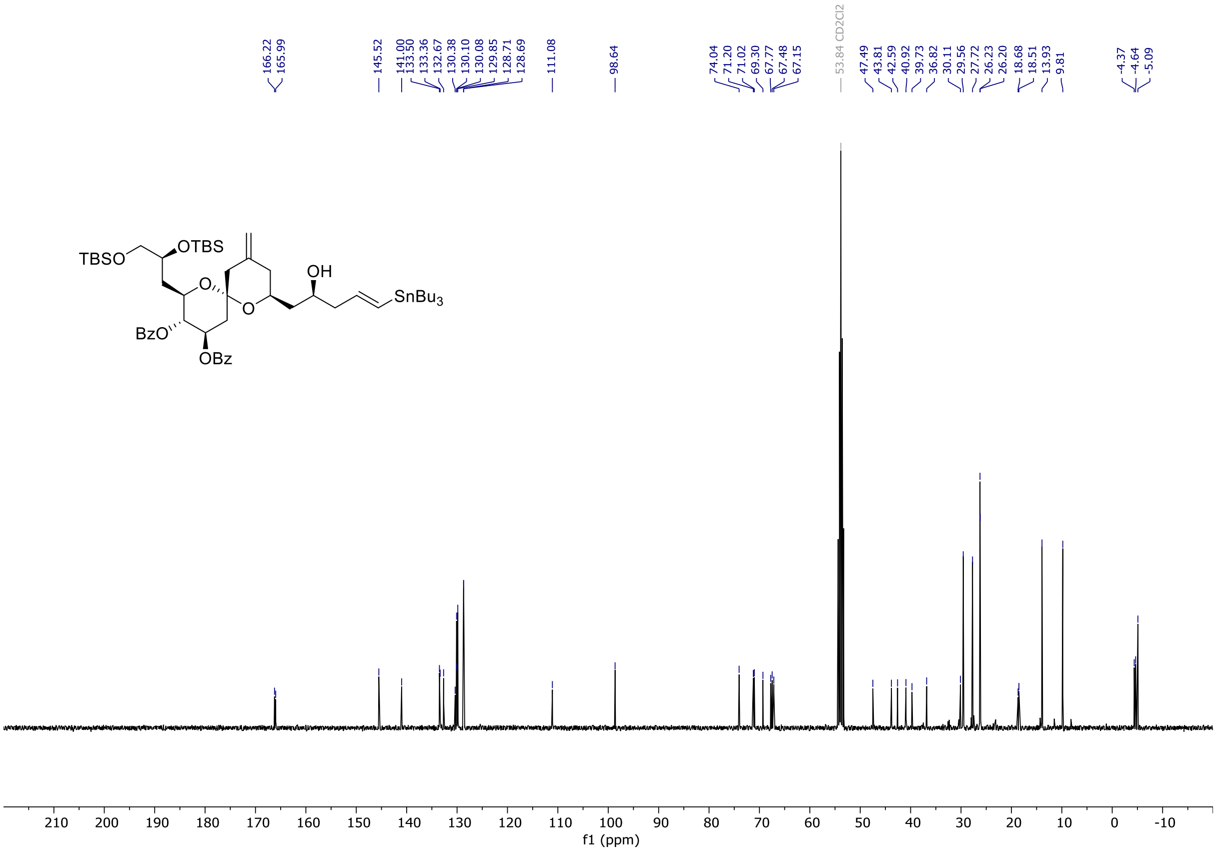

Compound 28: ^{119}Sn NMR (CD_2Cl_2, 149 MHz)

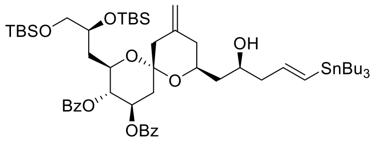

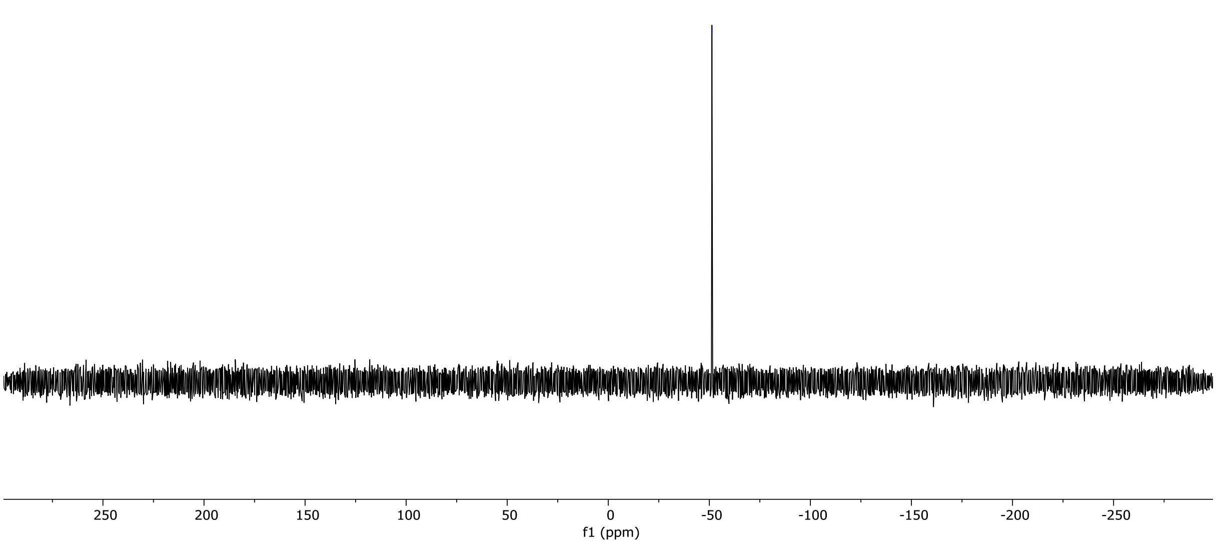

Mosher Esters derived from compound 28: (*R*)-MTPA ester: ^{1}H NMR (CD_2Cl_2, 400 MHz)

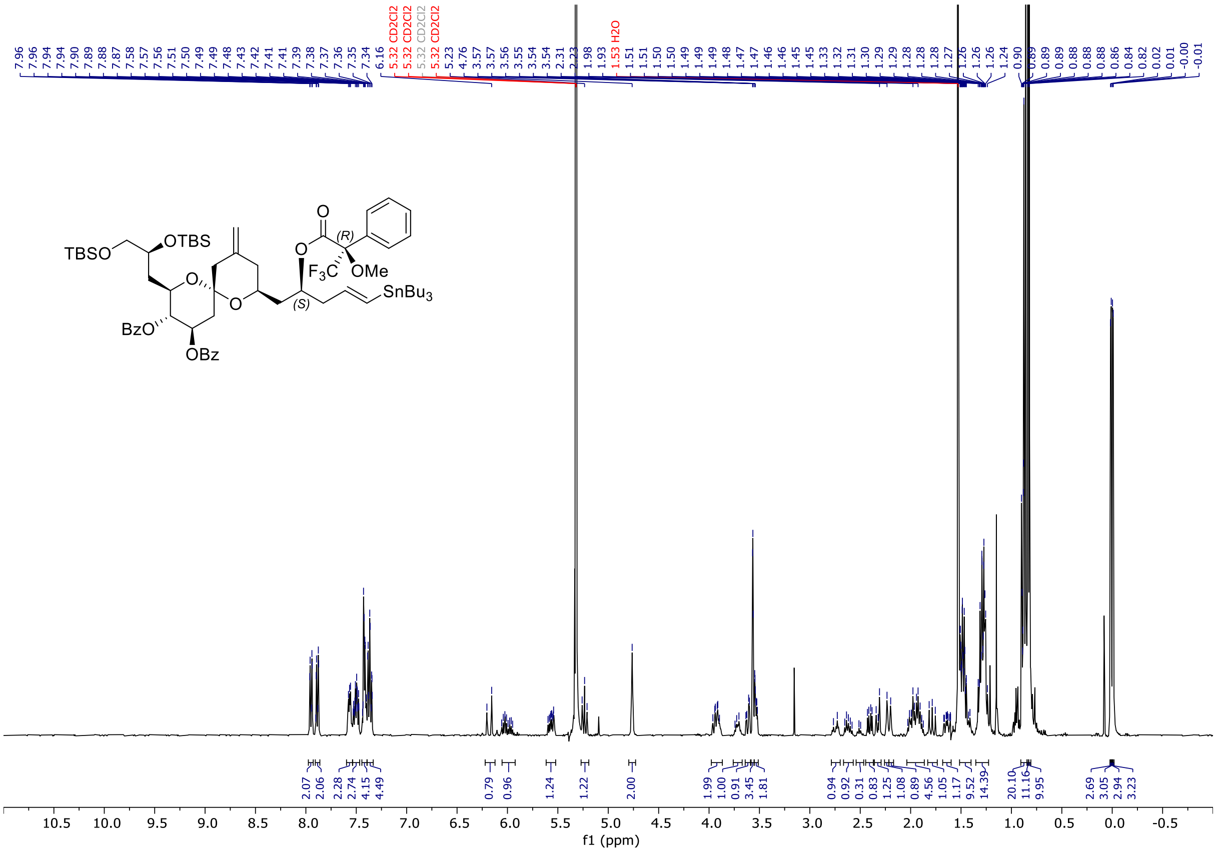

(*S*)-MTPA ester: ^{1}H NMR (CD_2Cl_2, 400 MHz)

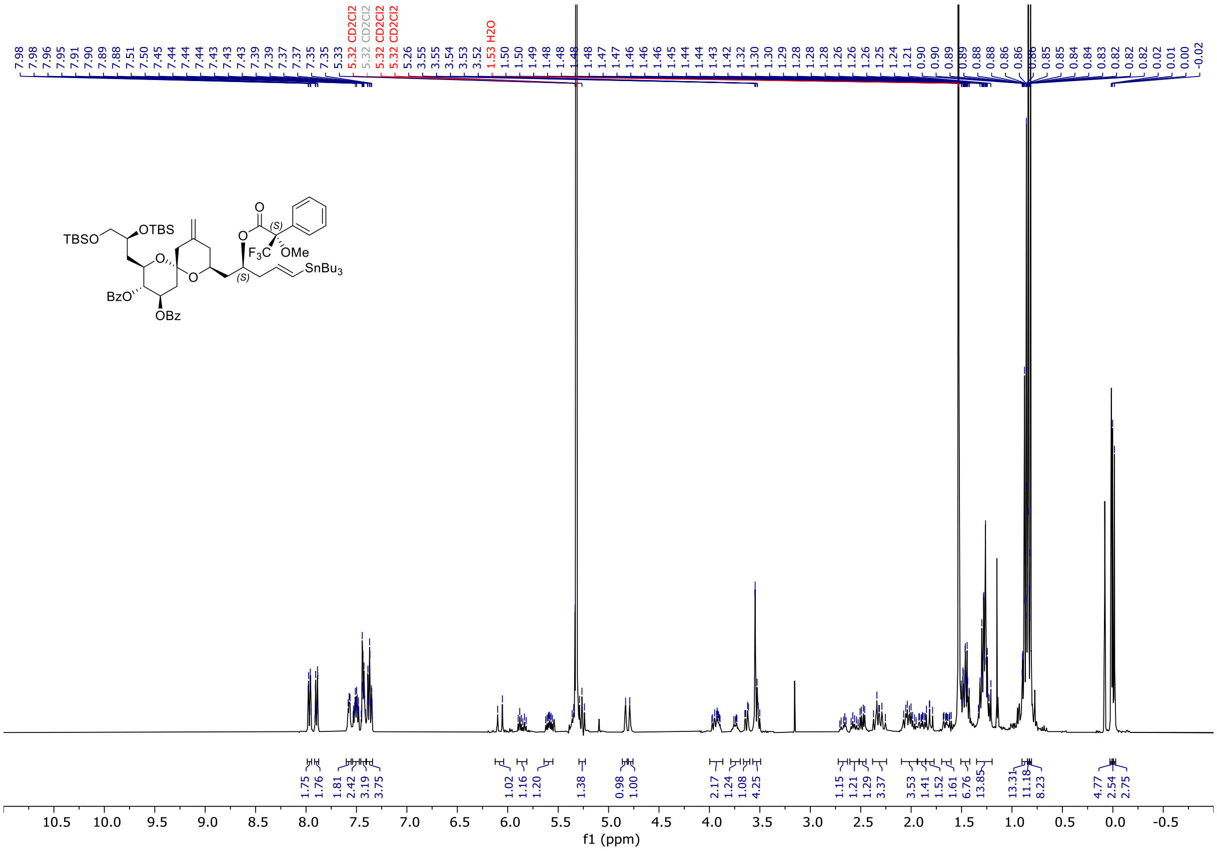

Compound 28a: ^{1}H NMR (400 MHz, $CDCl_3$)

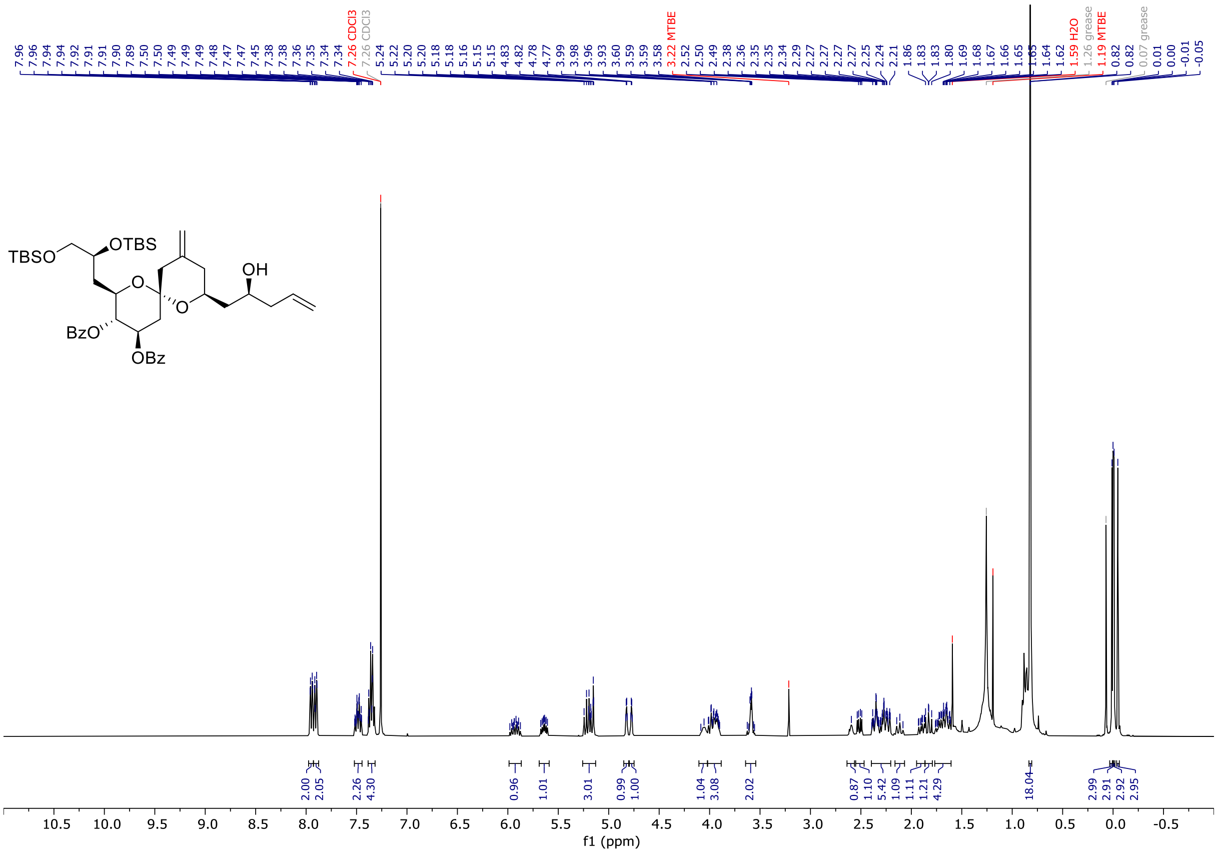

^{13}C NMR (101 MHz, $CDCl_3$)

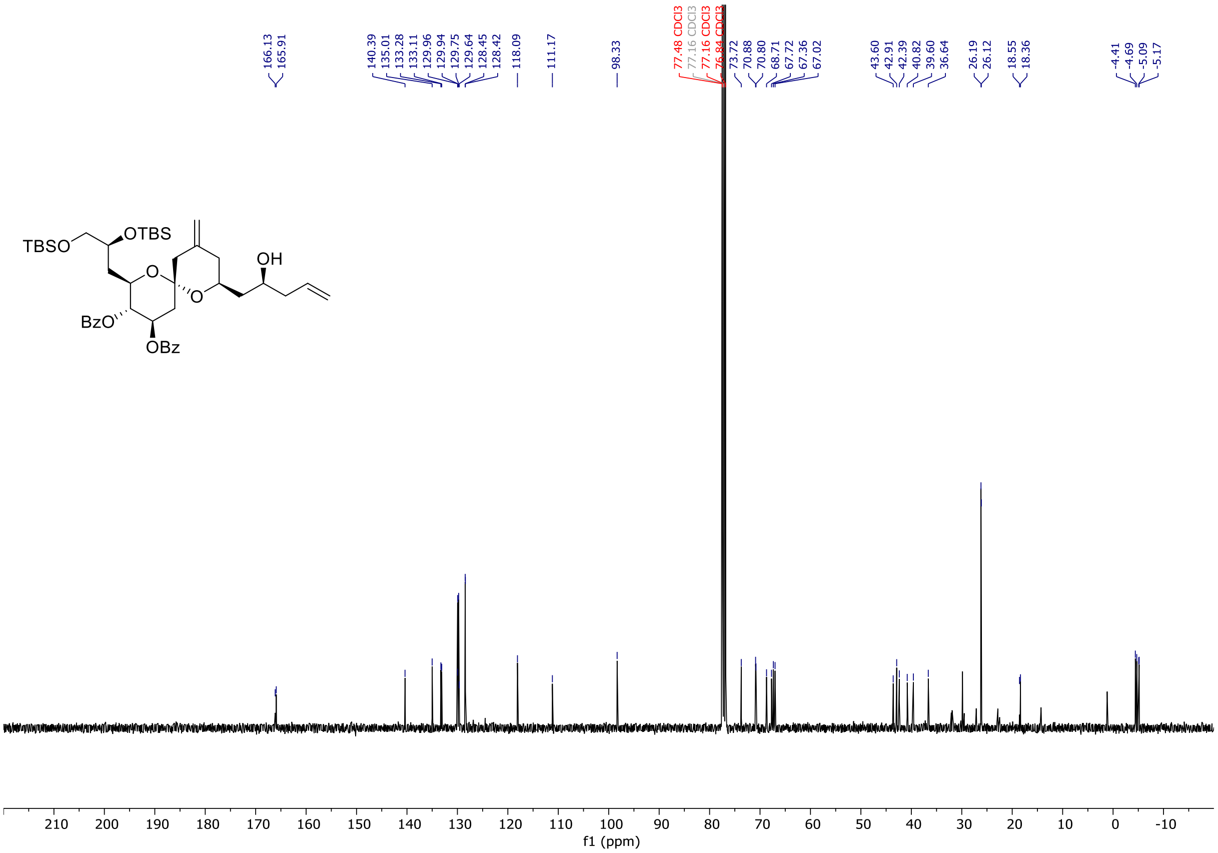

Compound 29a: ^{1}H NMR (600 MHz, $CDCl_3$)

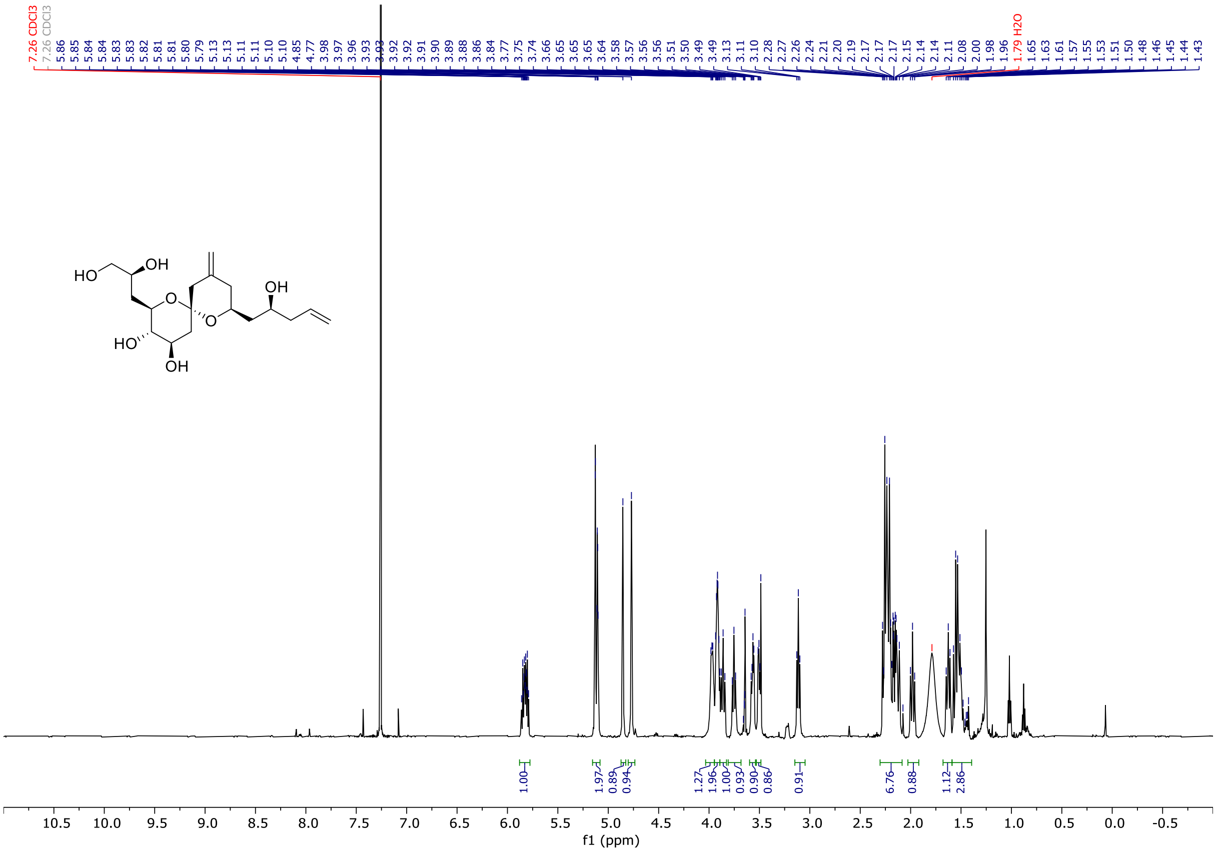

^{13}H NMR (151 MHz, $CDCl_3$)

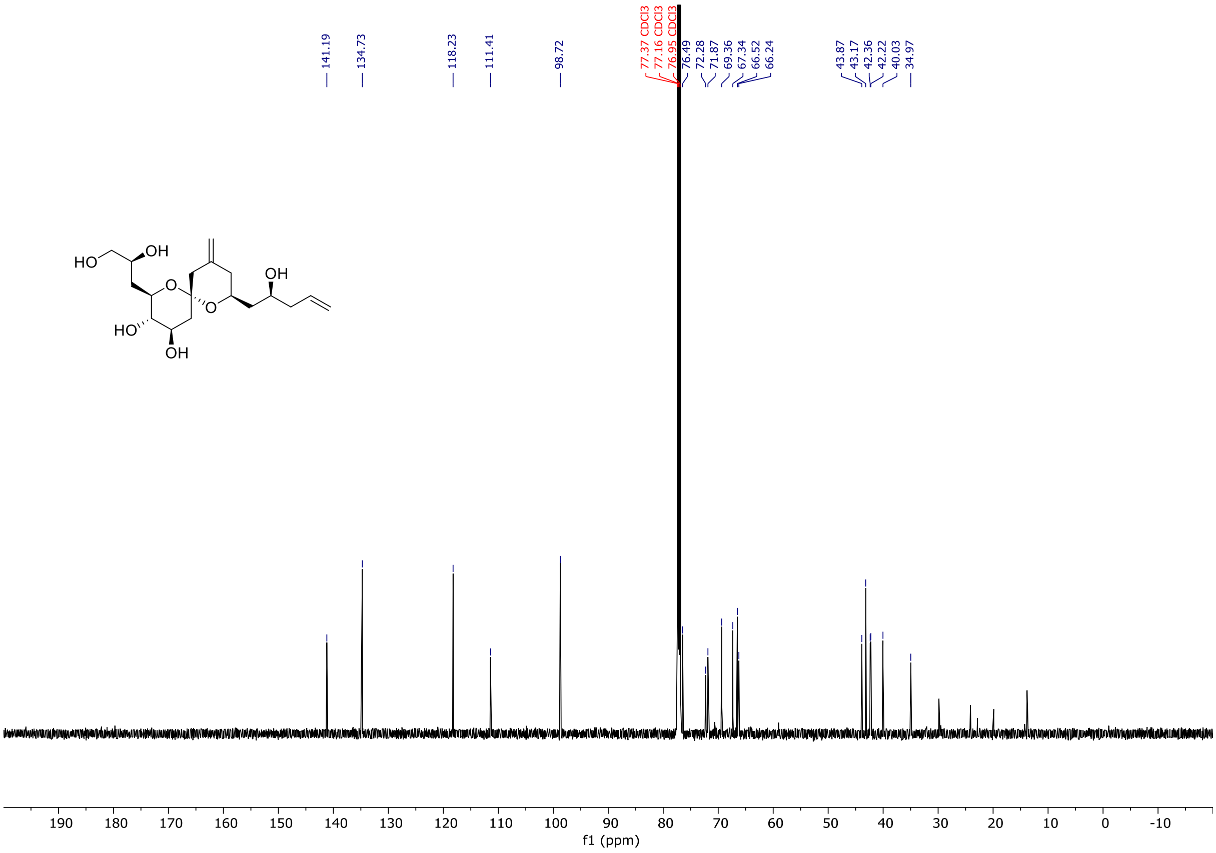

Compound 29: ^{1}H NMR (600 MHz, [D$_4$]-MeOH)

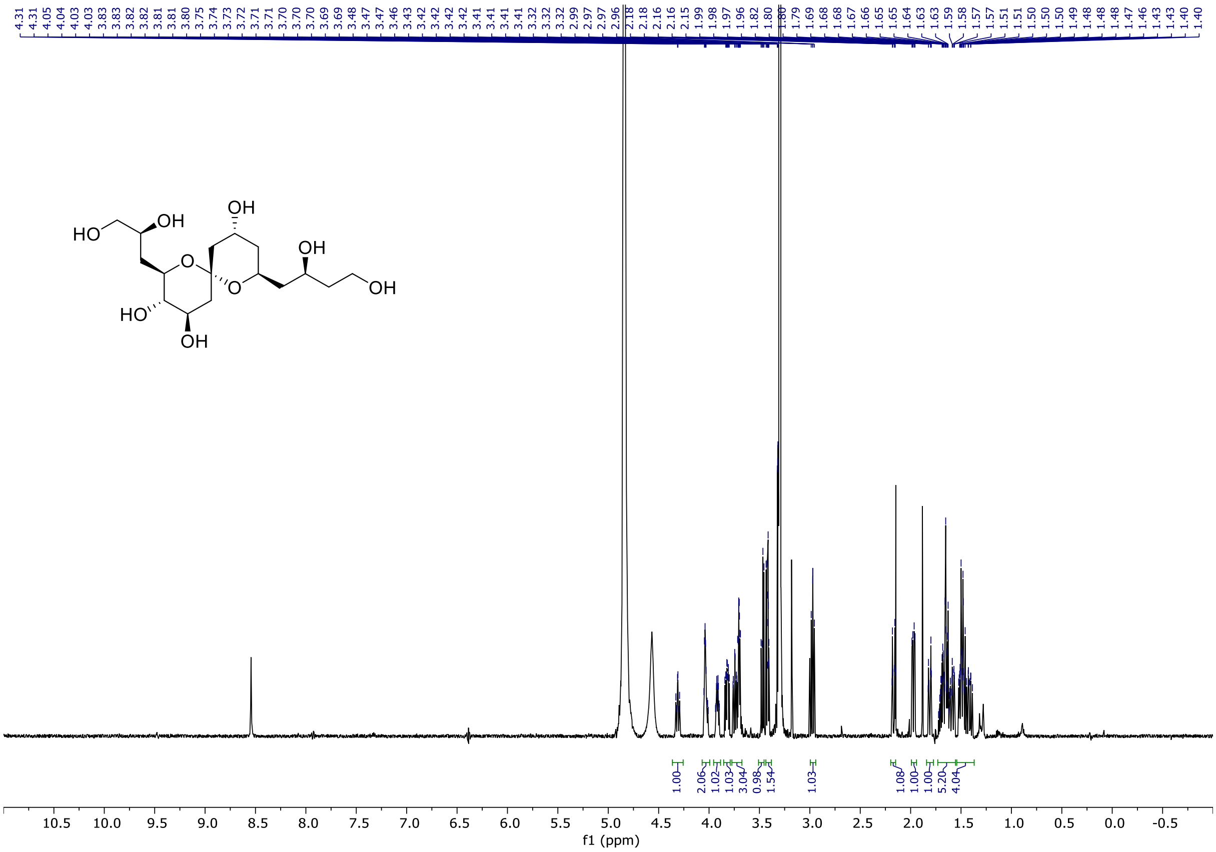

^{13}C NMR (151 MHz, [D$_4$]-MeOH)

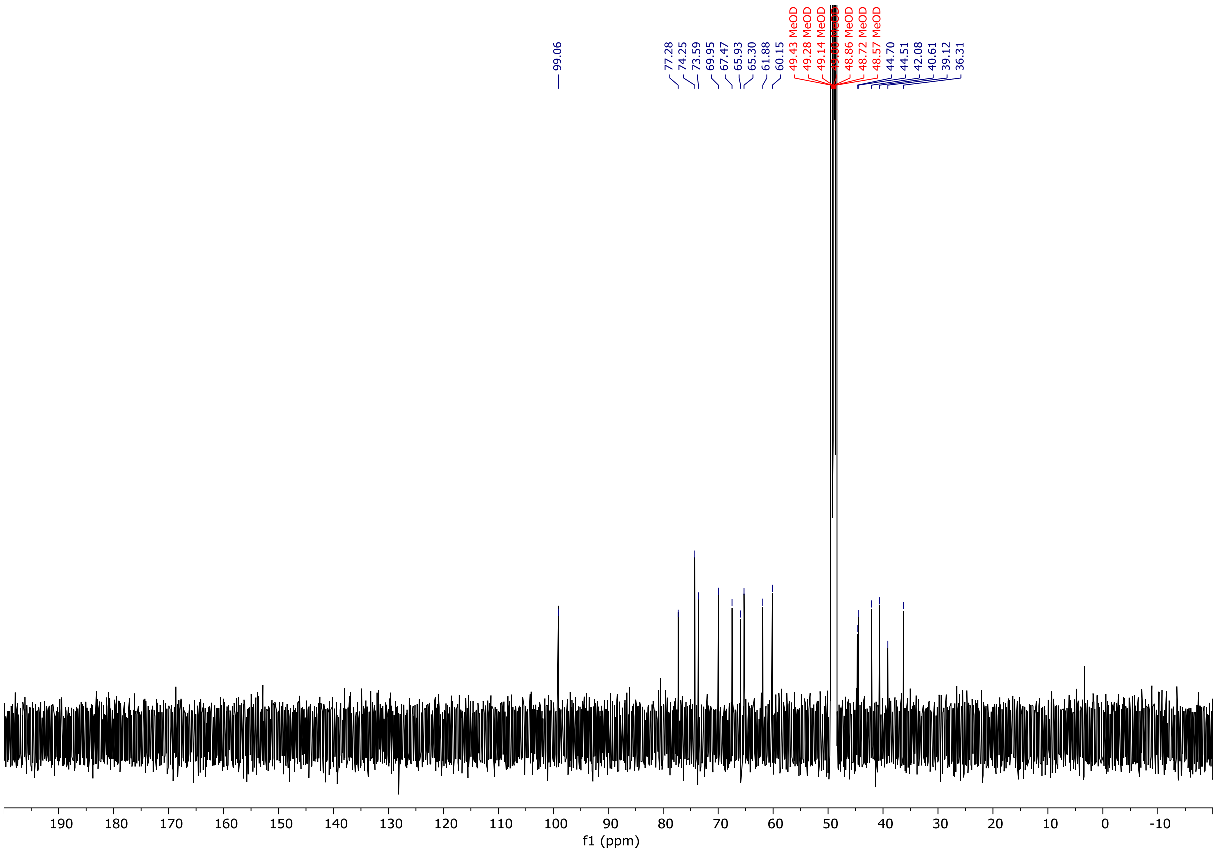

Compound 32: ^{1}H NMR (400 MHz, $CDCl_3$)

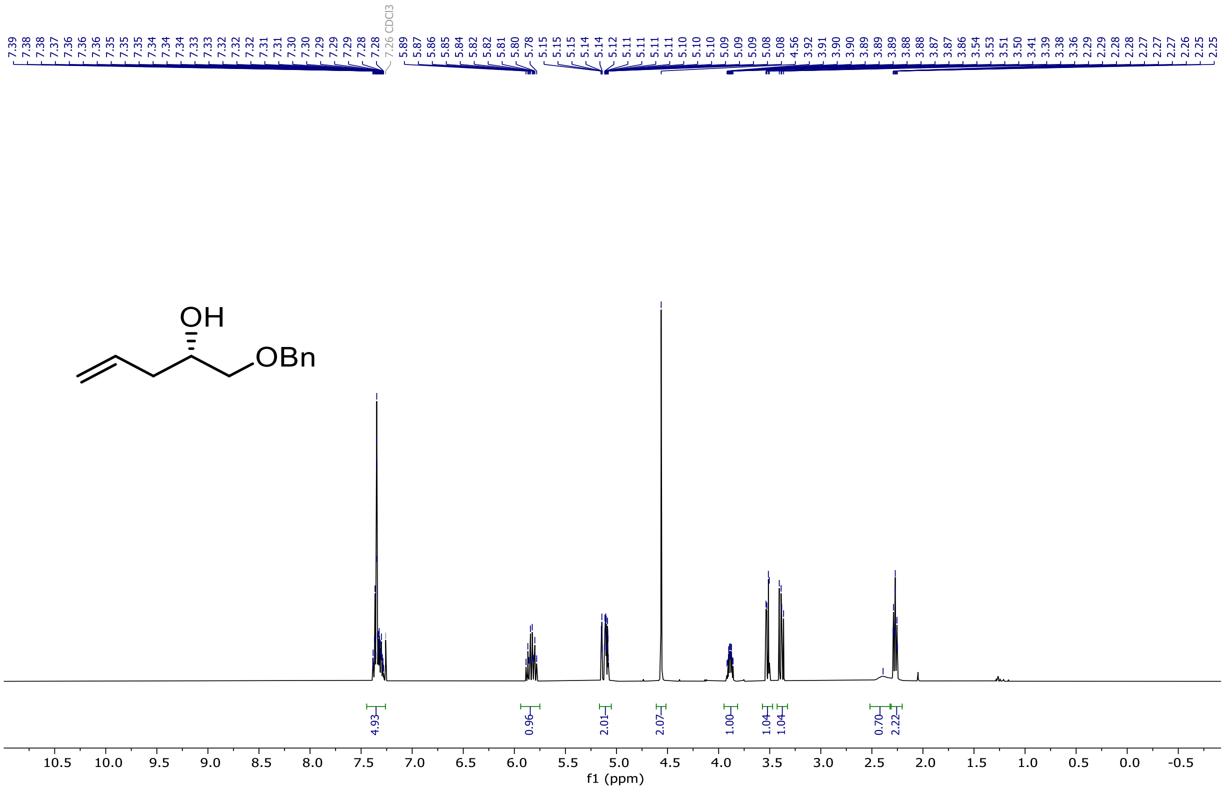

^{13}C NMR (101 MHz, $CDCl_3$)

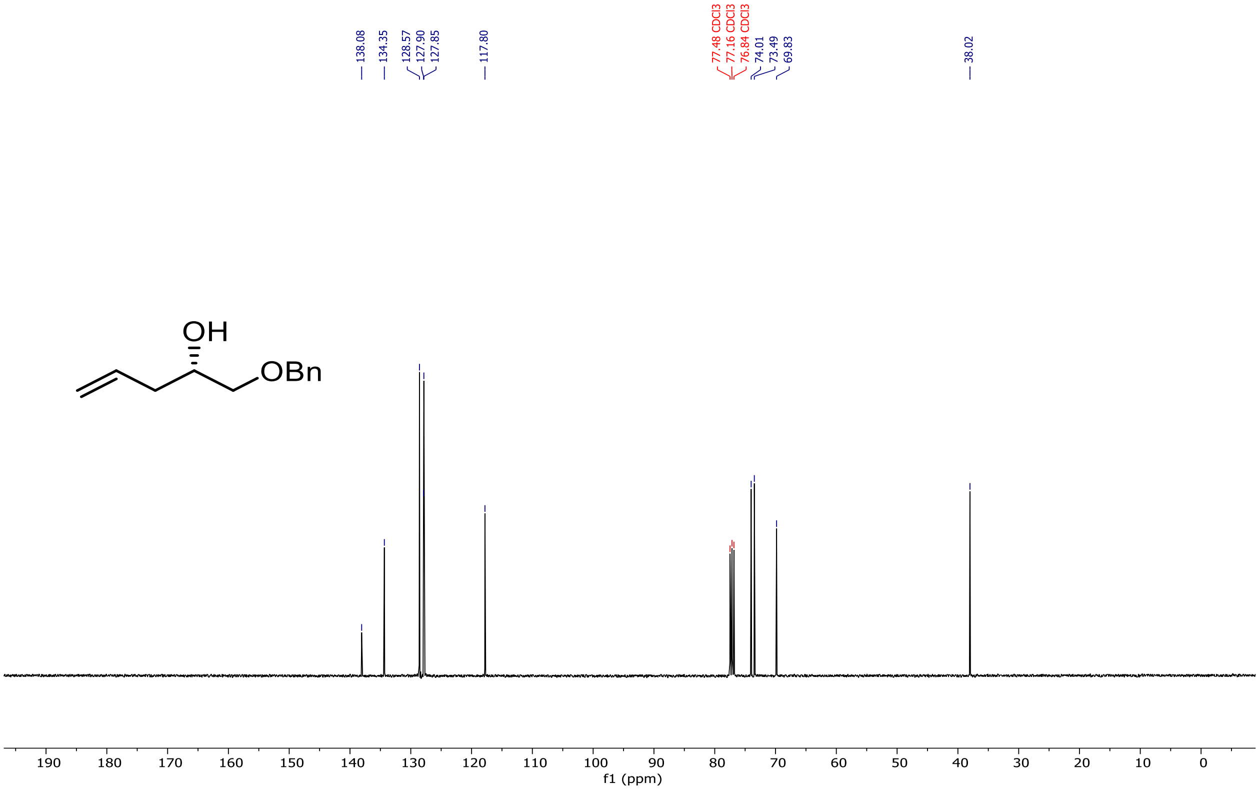

Compound S11: ^{1}H NMR (400 MHz, $CDCl_3$)

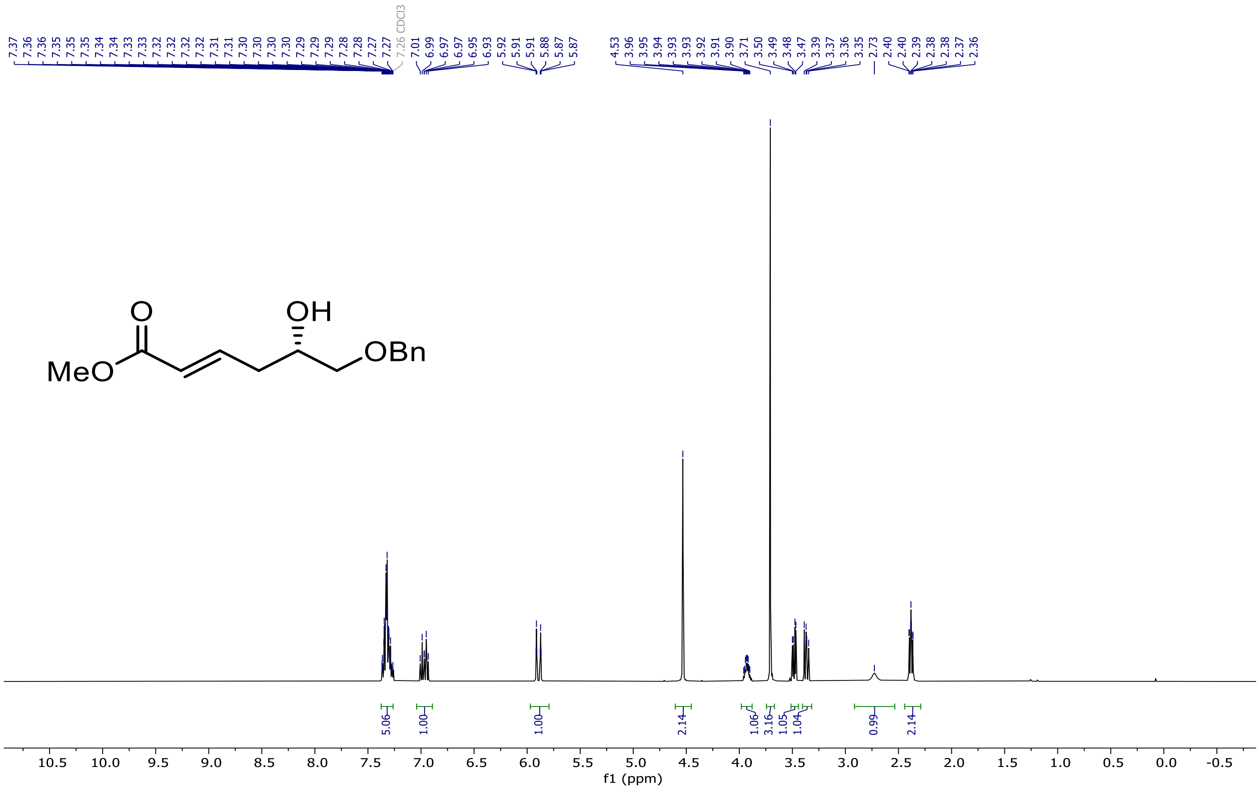

^{13}C NMR (101 MHz, $CDCl_3$)

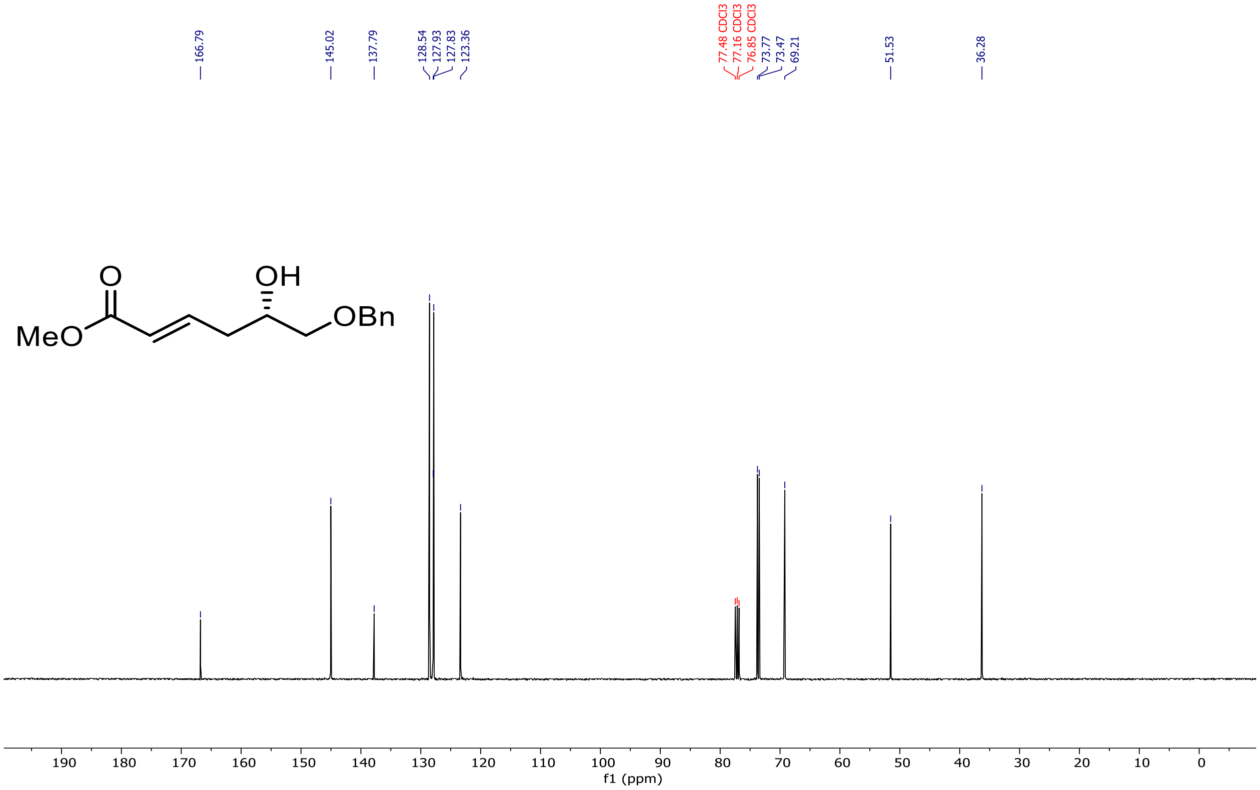

Compound 33: ^{1}H NMR (400 MHz, $CDCl_3$)

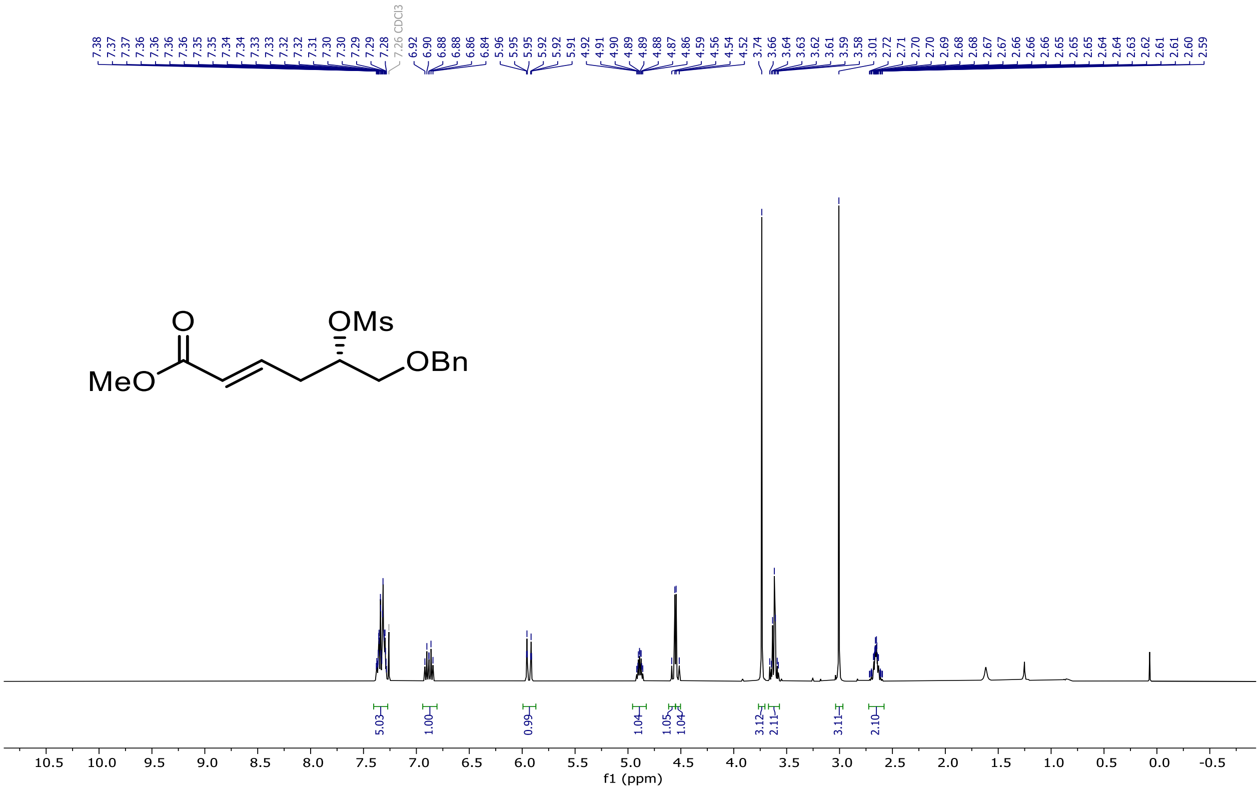

^{13}C NMR (101 MHz, $CDCl_3$)

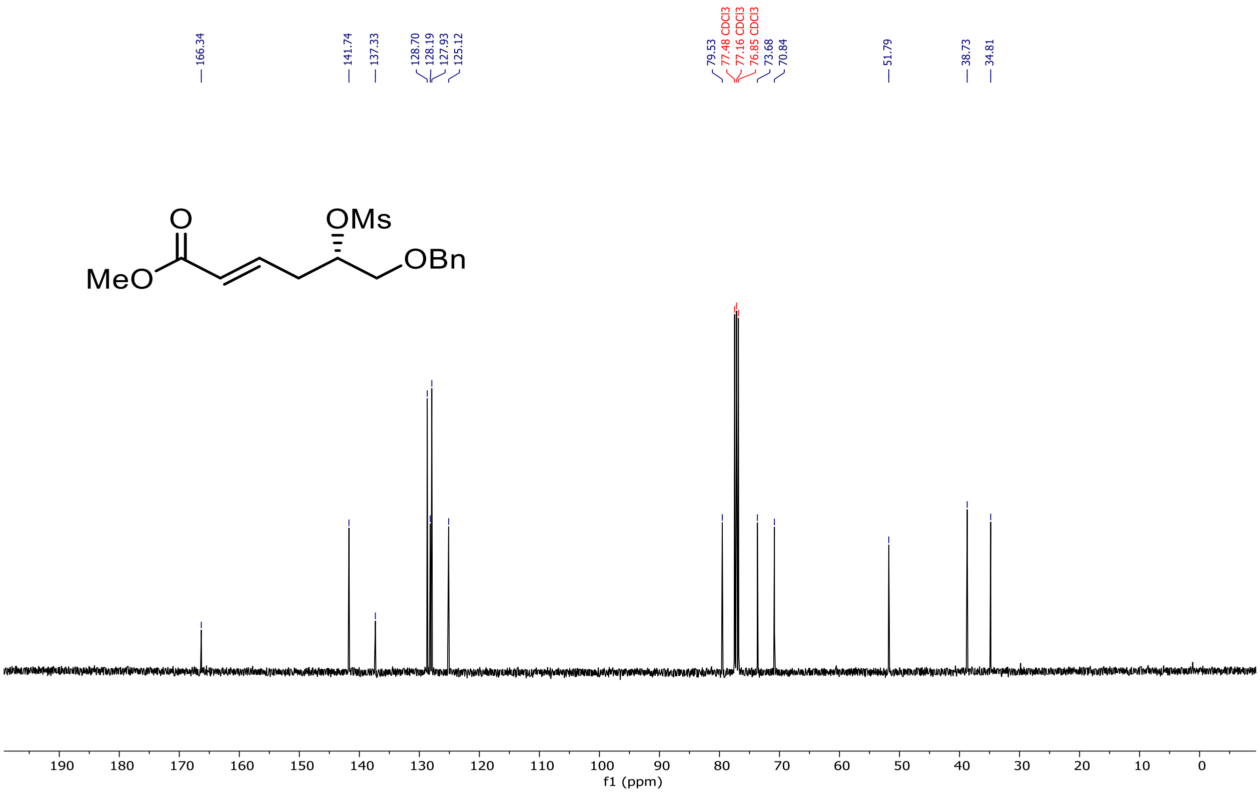

Compound S12: ^{1}H NMR (400 MHz, $CDCl_3$)

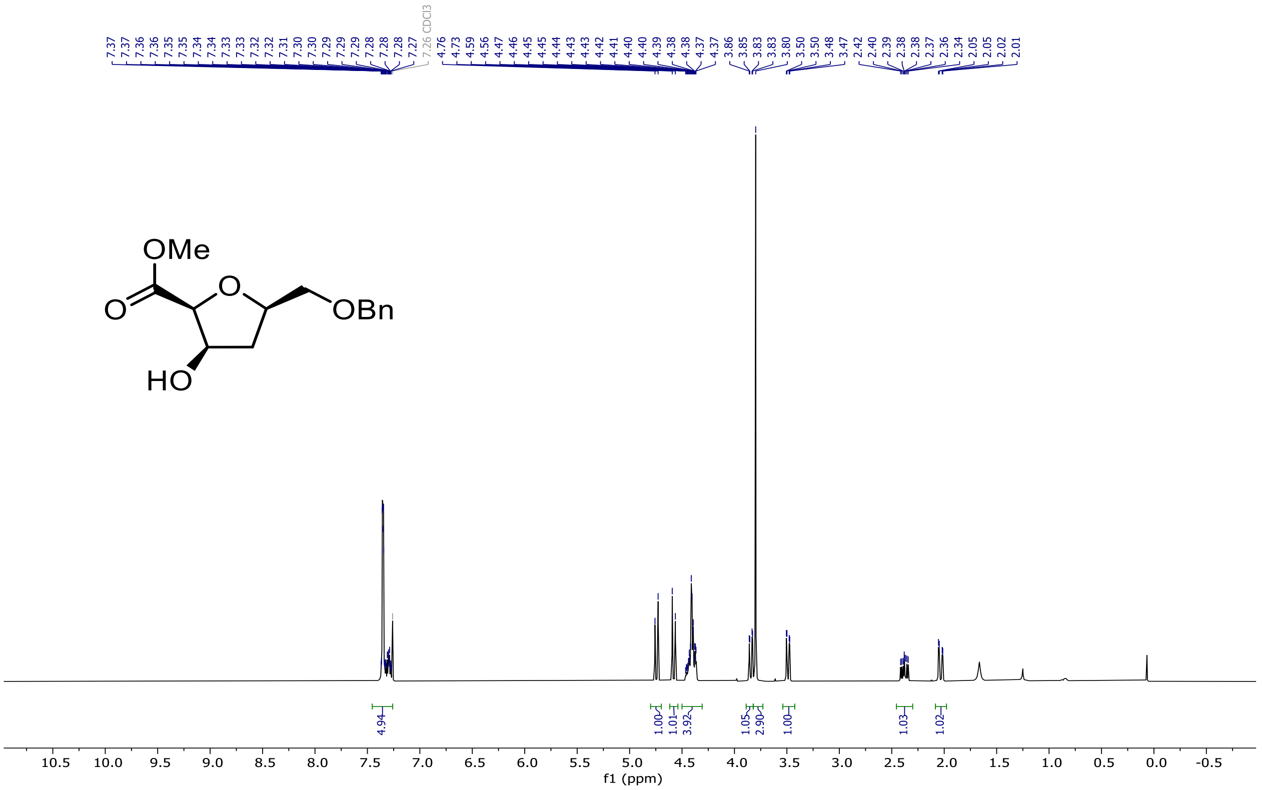

^{13}C NMR (101 MHz, $CDCl_3$)

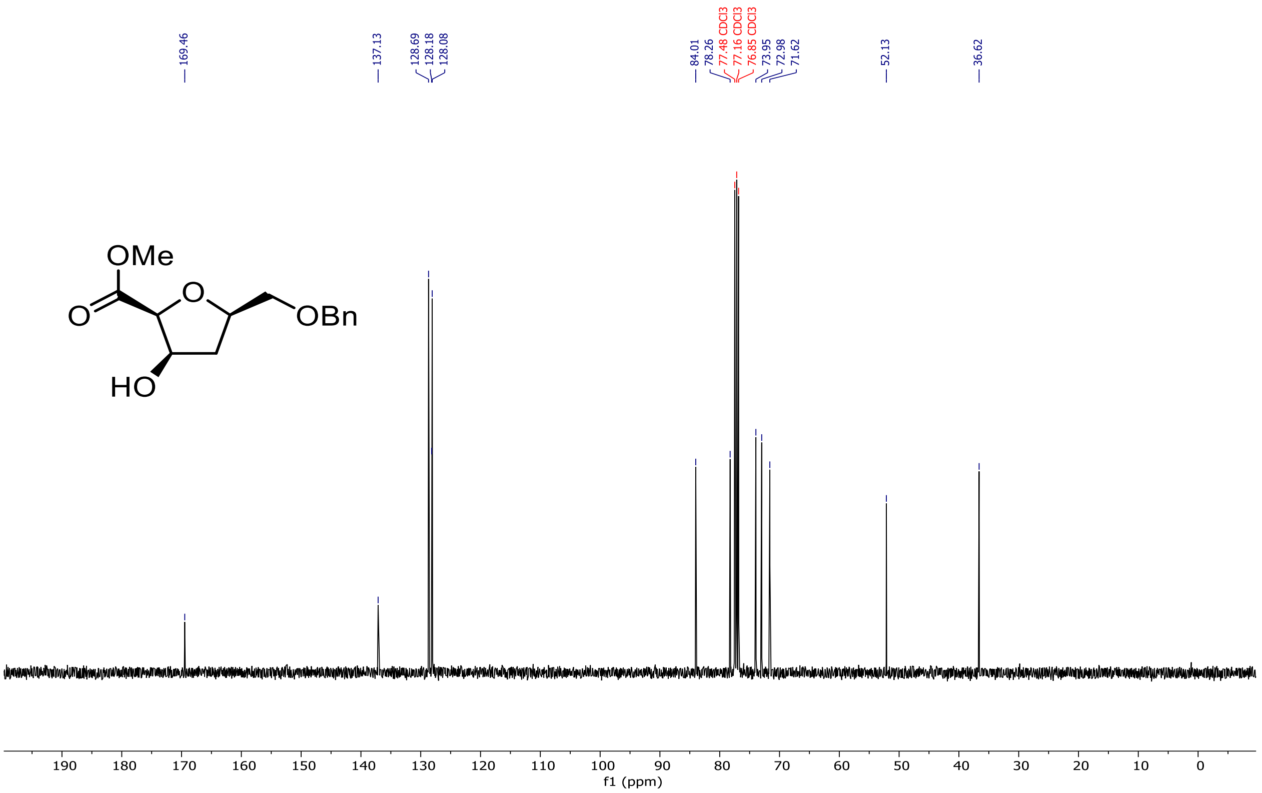

Compound 35: ^{1}H NMR (400 MHz, $CDCl_3$)

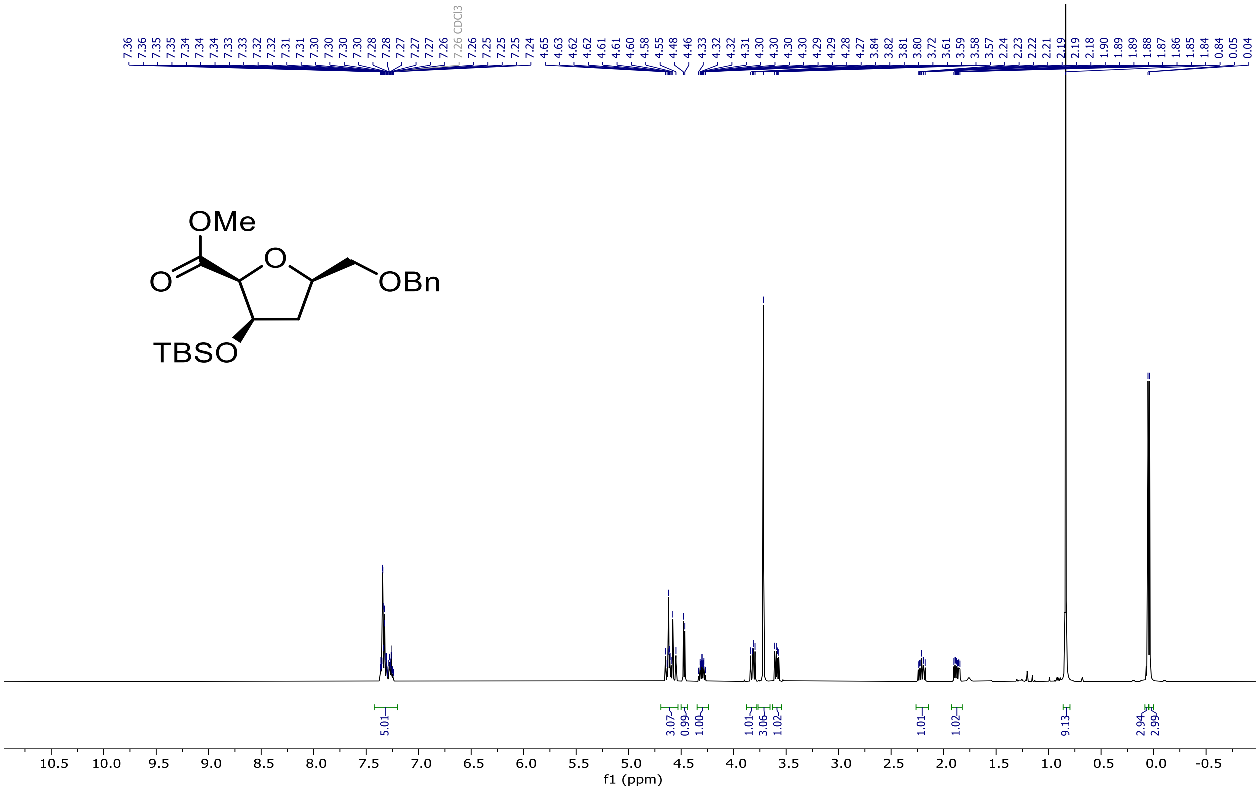

^{13}C NMR (101 MHz, $CDCl_3$)

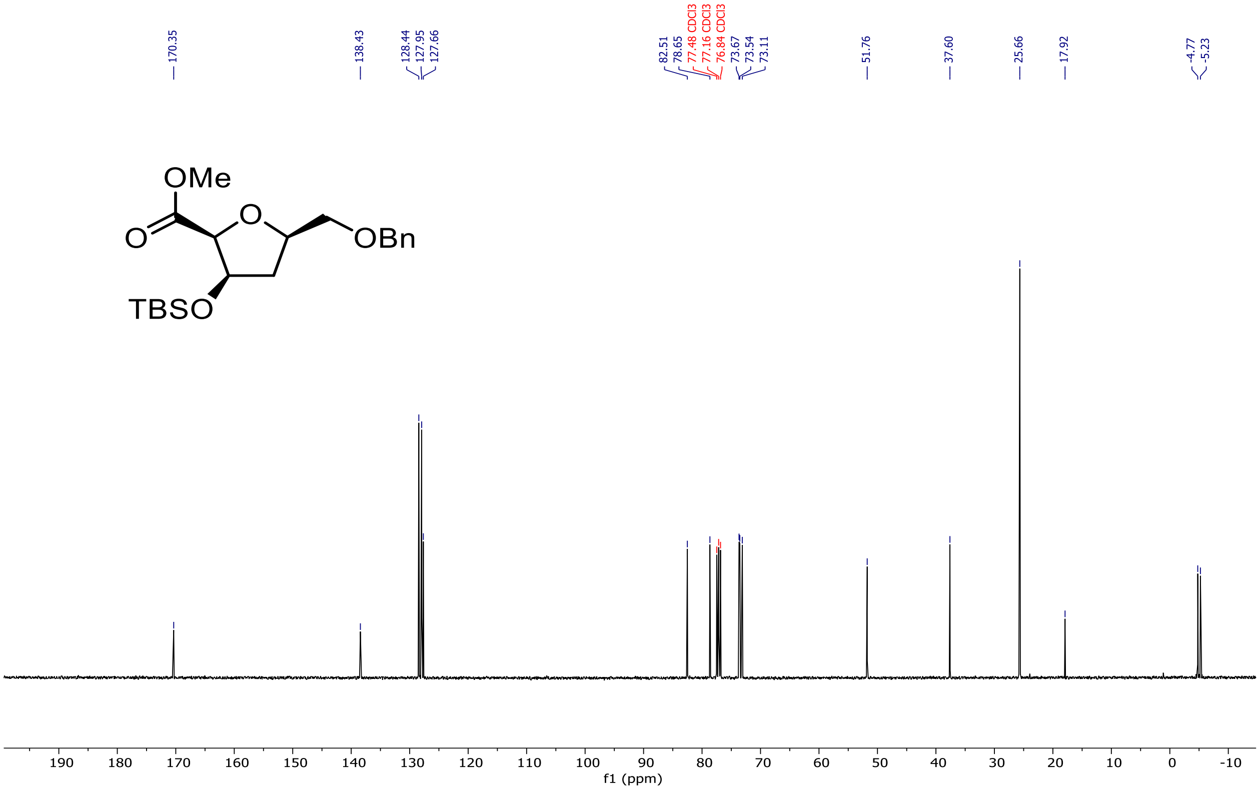

Compound S13: ^{1}H NMR (400 MHz, $CDCl_3$)

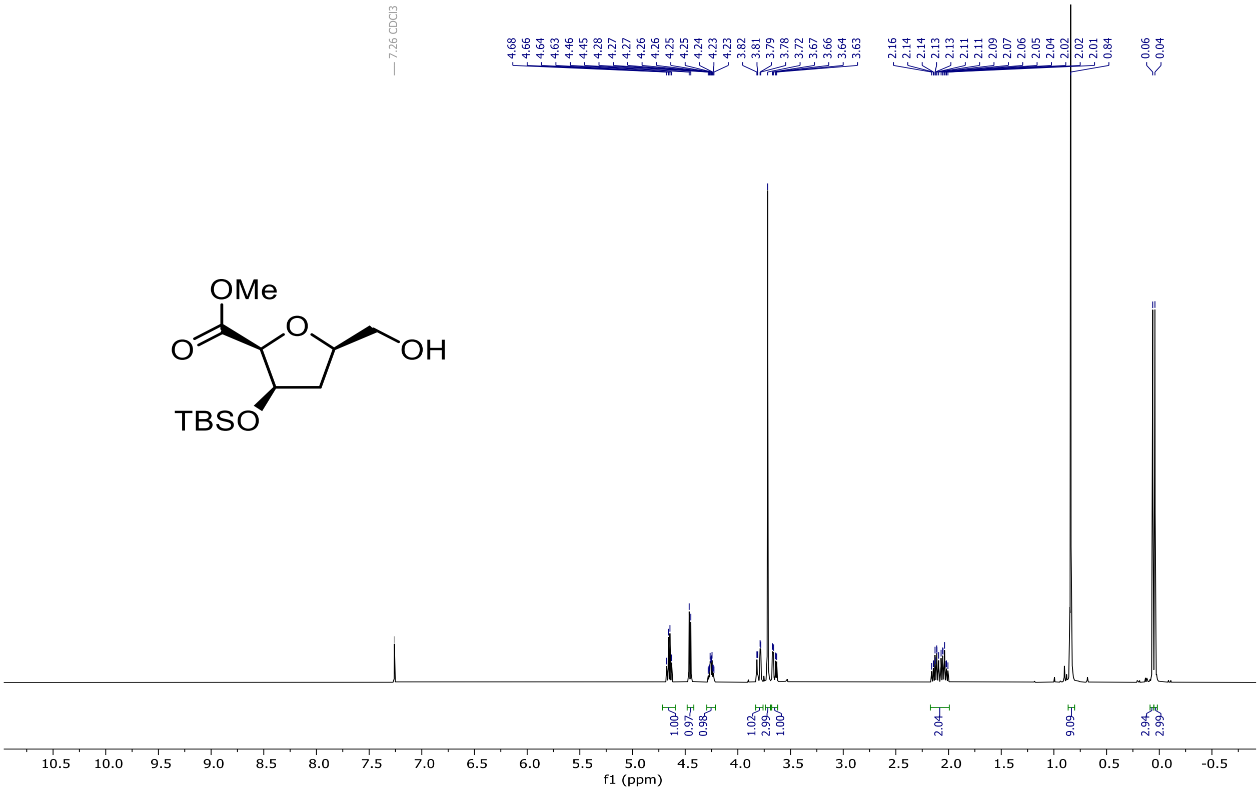

^{13}C NMR (101 MHz, $CDCl_3$)

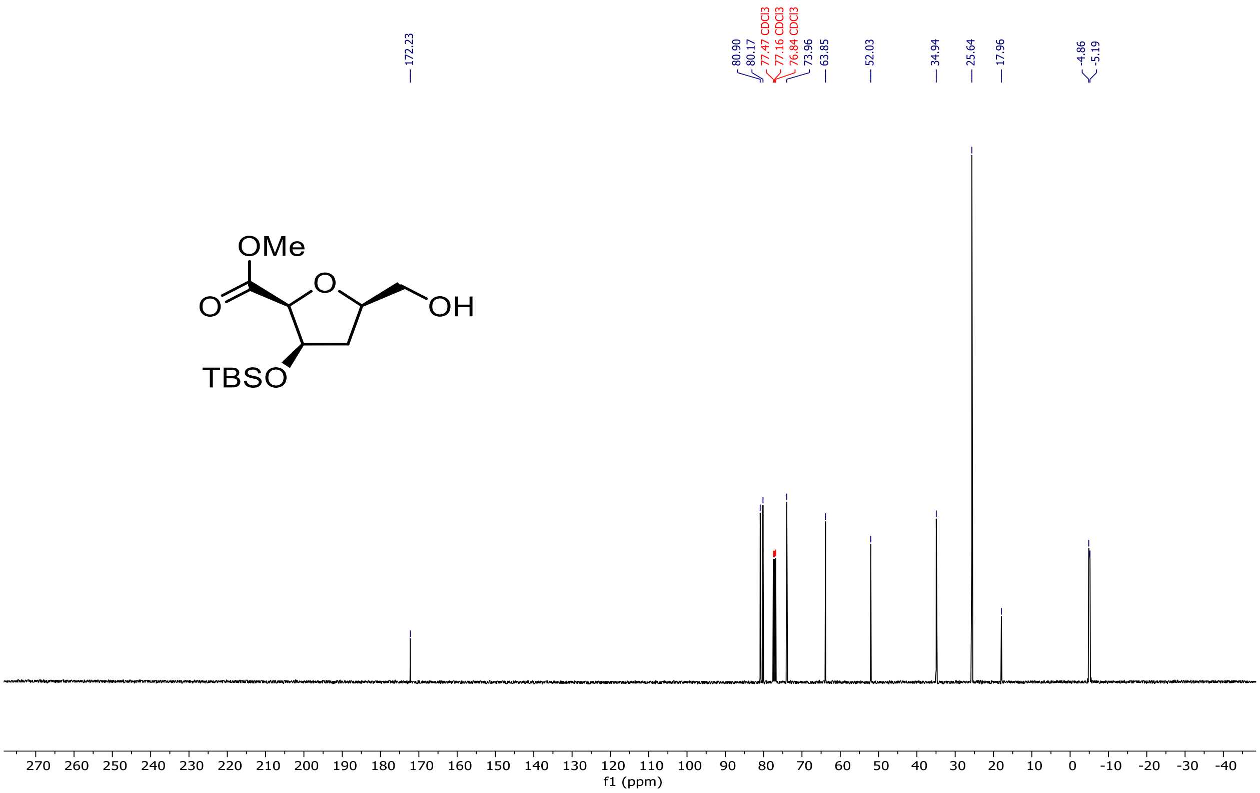

Compound S14: ^{1}H NMR (400 MHz, $CDCl_3$)

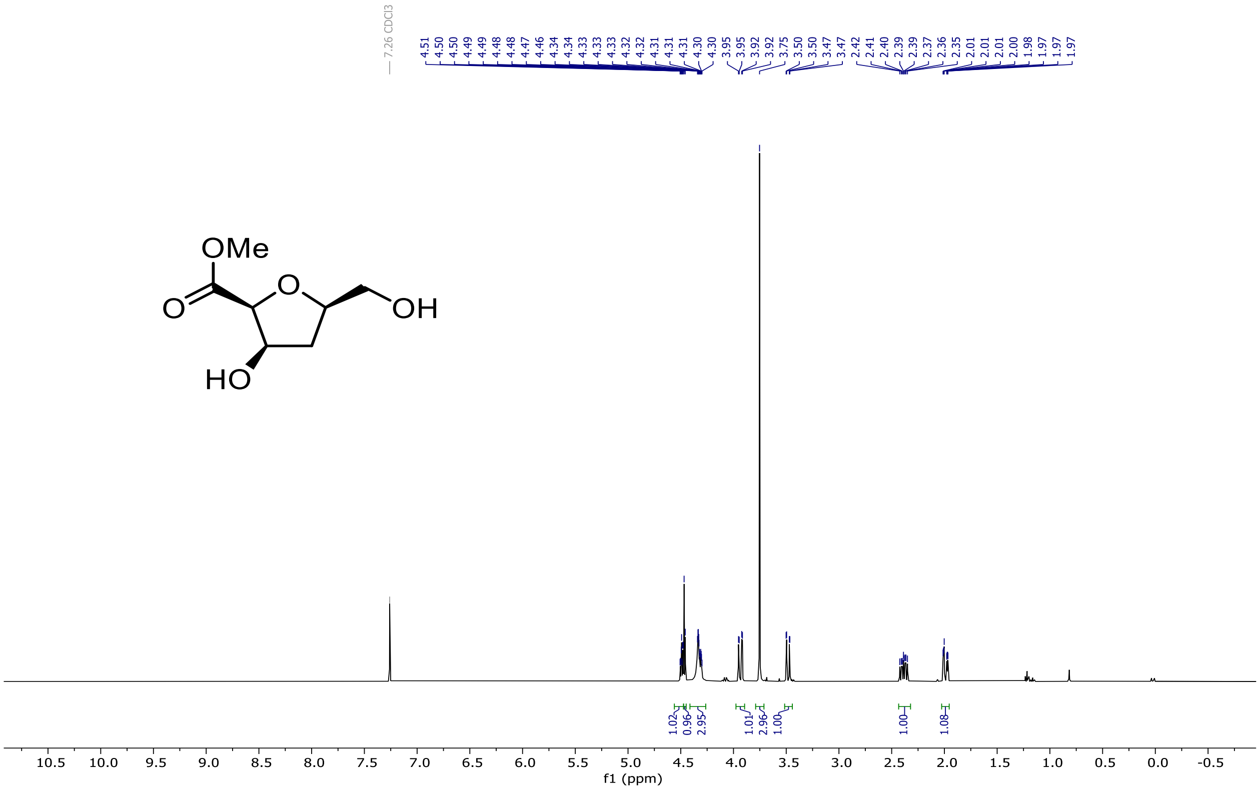

^{13}C NMR (101 MHz, $CDCl_3$)

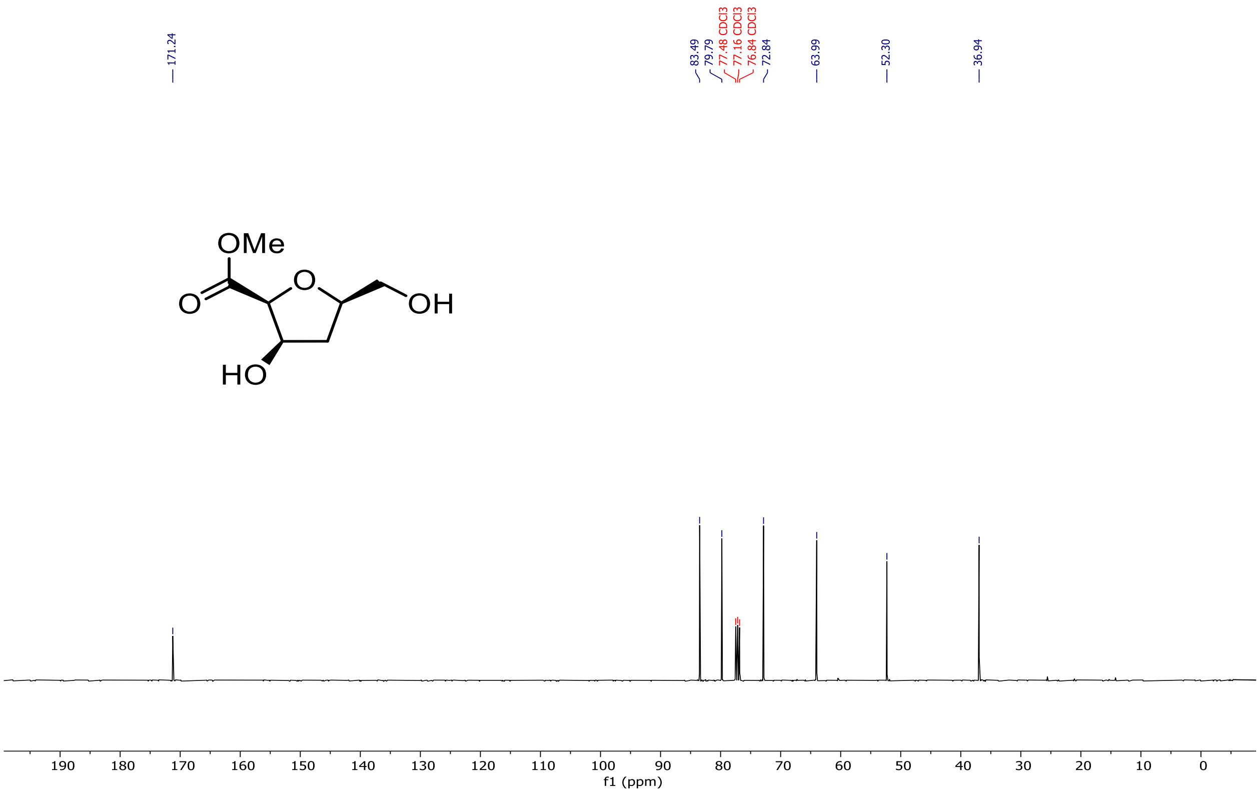

Compound 37: ^{1}H NMR (400 MHz, $CDCl_3$)

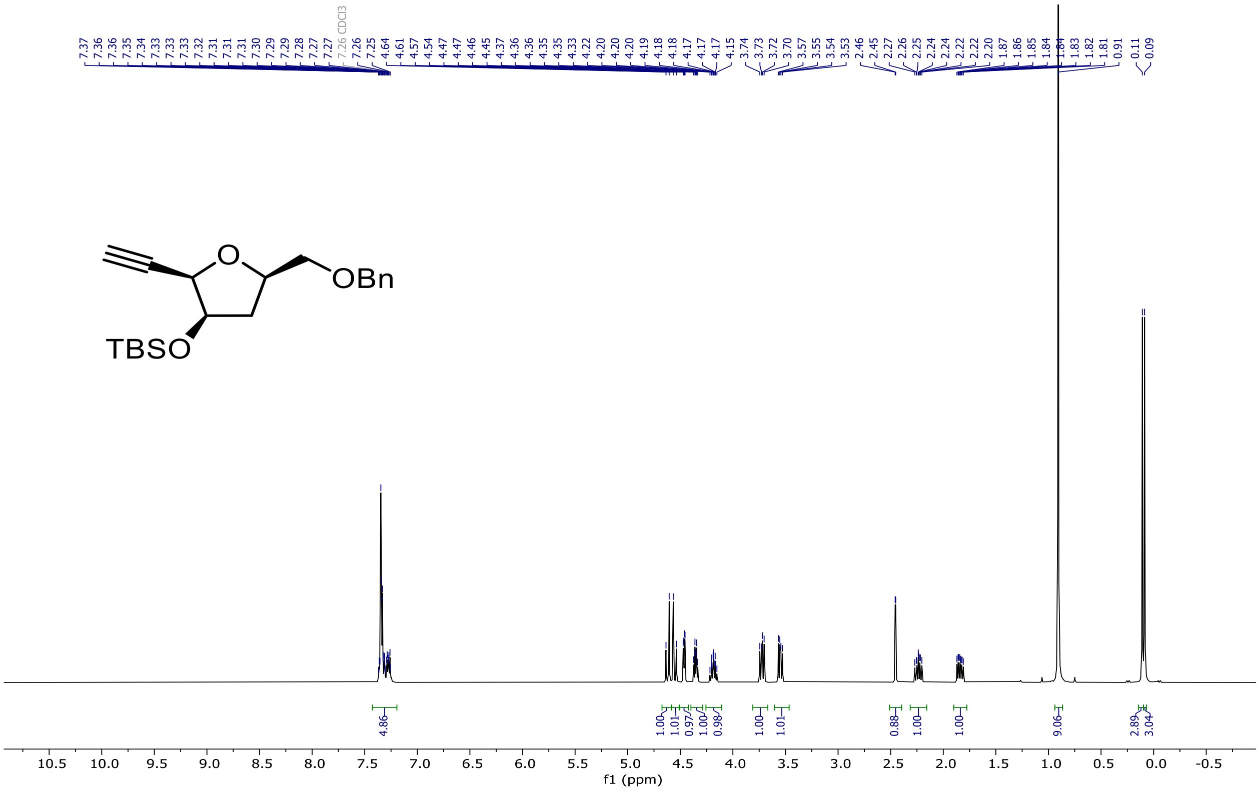

^{13}C NMR (101 MHz, $CDCl_3$)

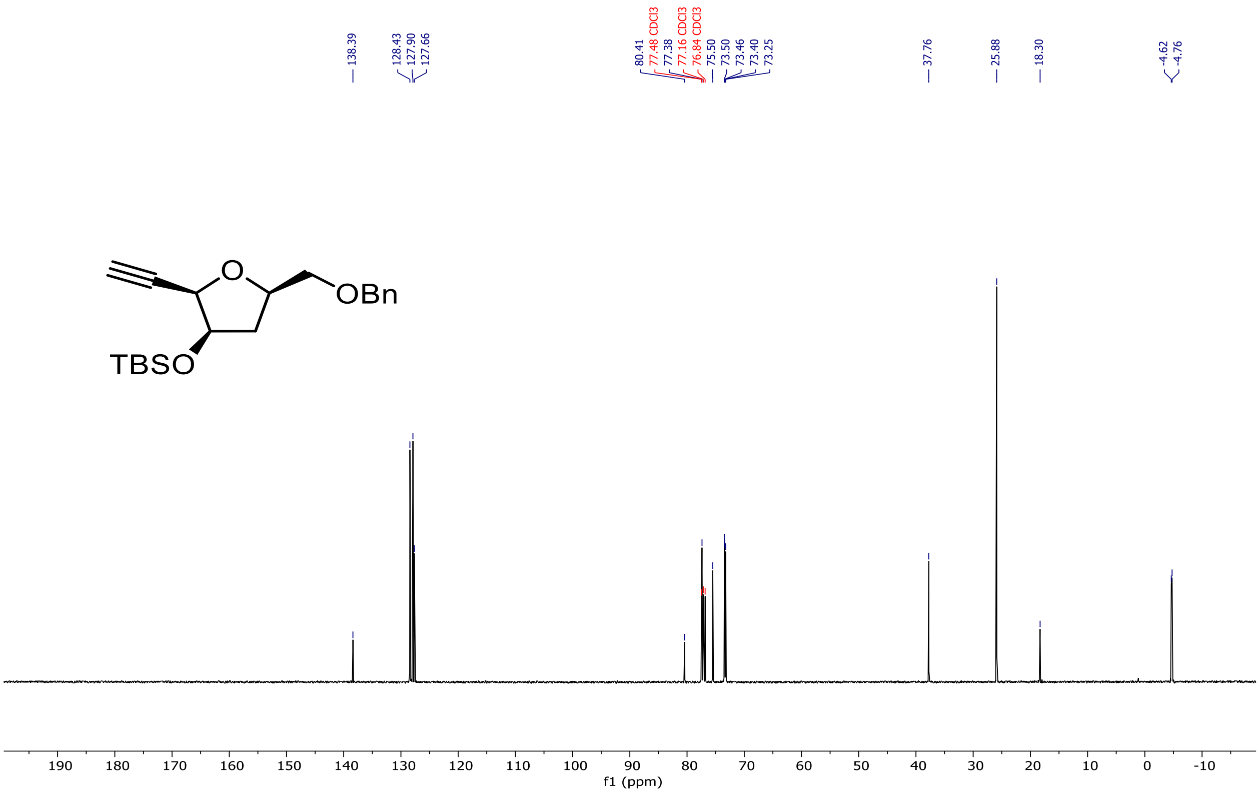

Compound 38: ^{1}H NMR (400 MHz, $CDCl_3$)

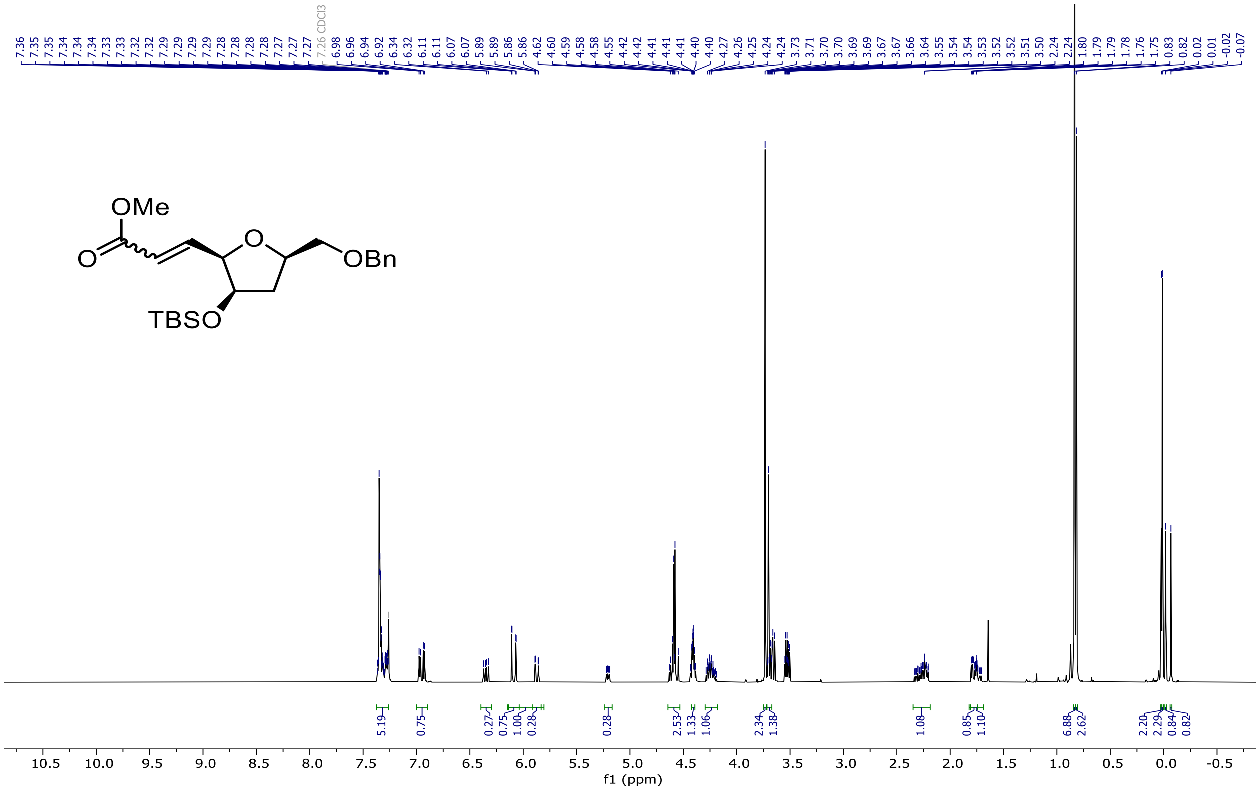

^{13}C NMR (101 MHz, $CDCl_3$)

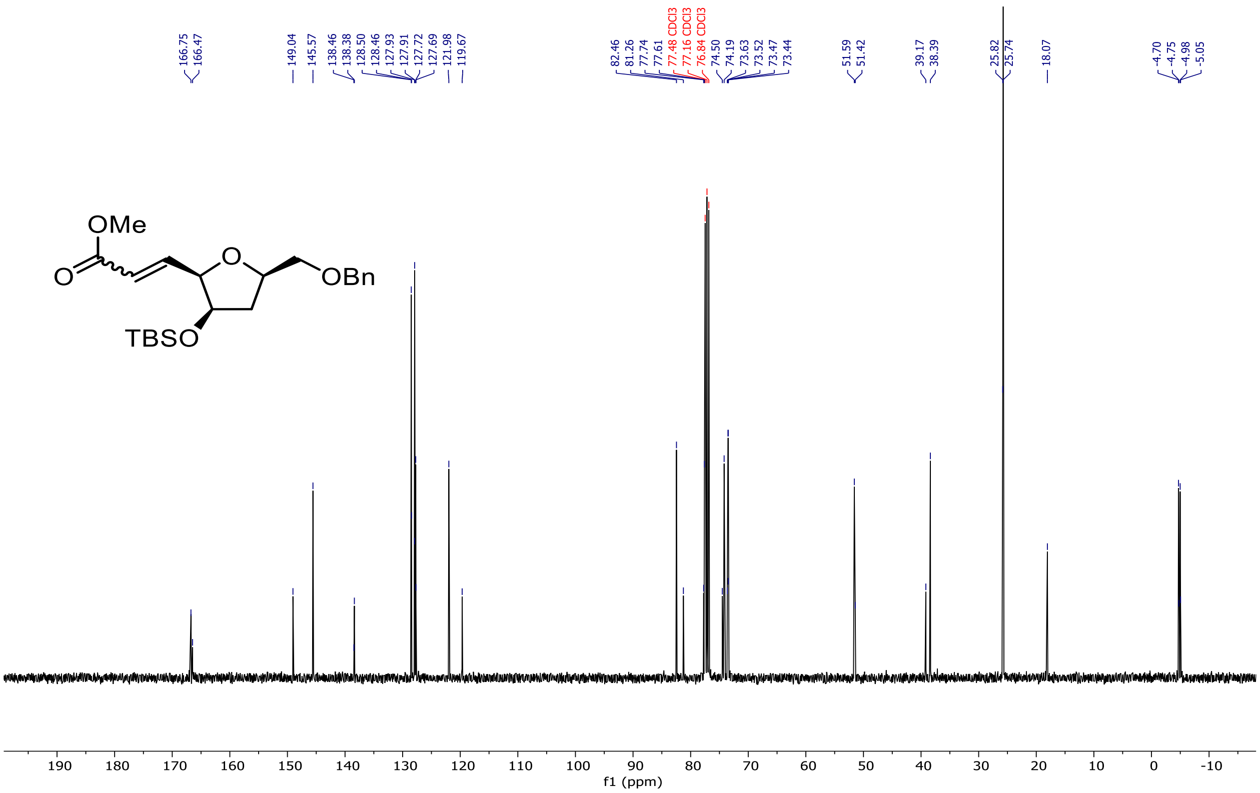

Compound S15: ^{1}H NMR (400 MHz, $CDCl_3$)

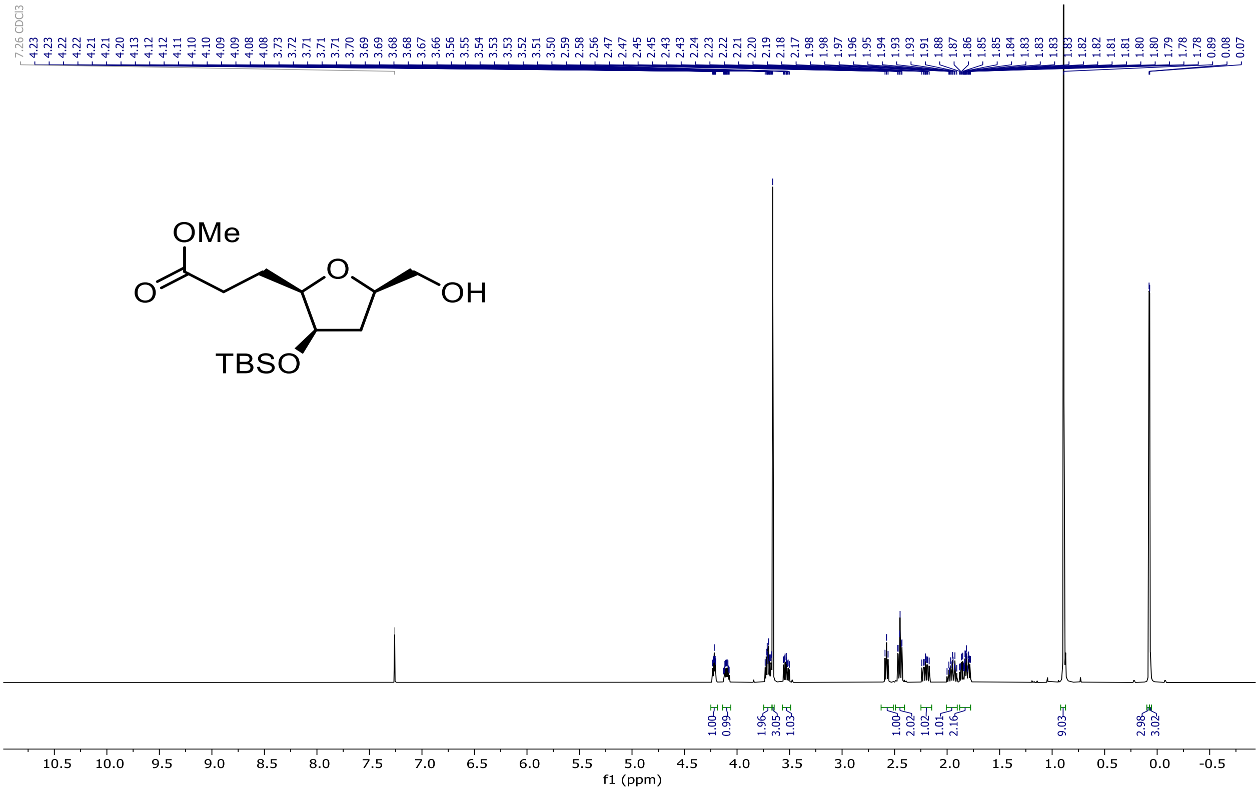

^{13}C NMR (101 MHz, $CDCl_3$)

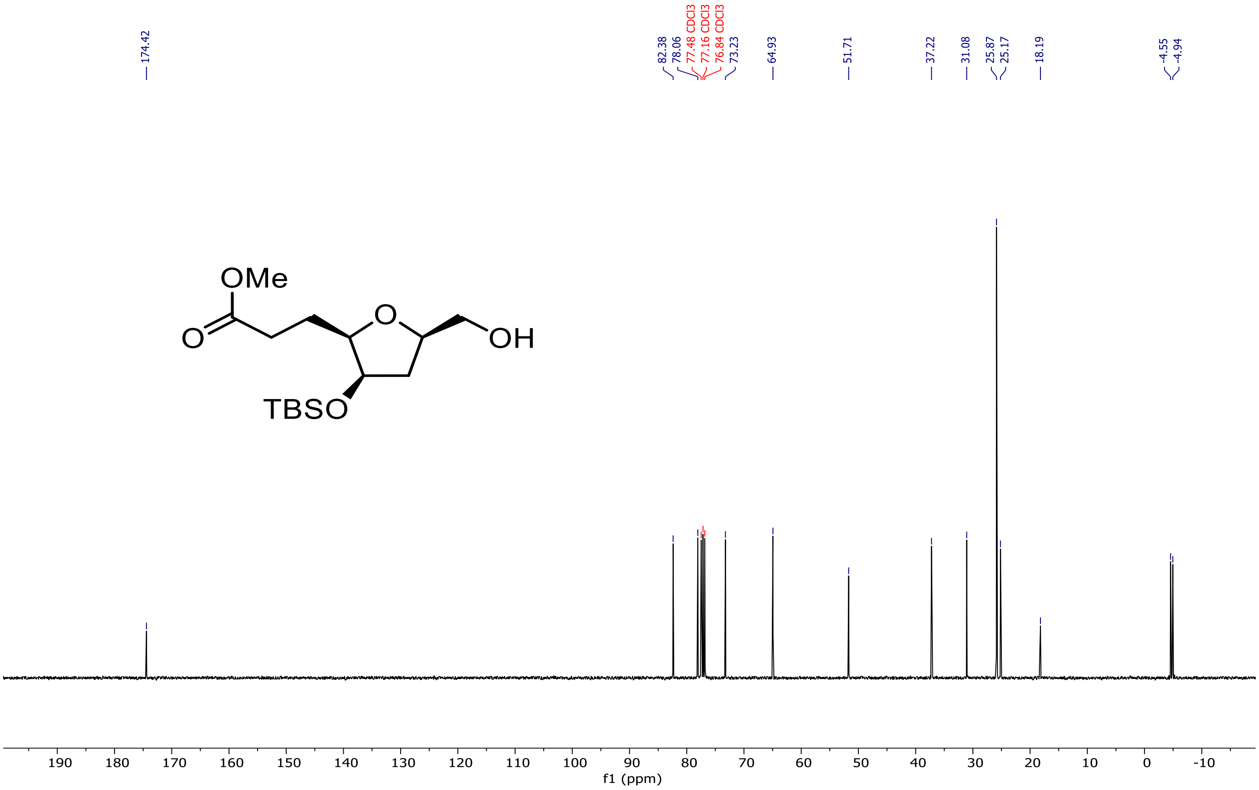

Compound 39: ^{1}H NMR (400 MHz, $CDCl_3$)

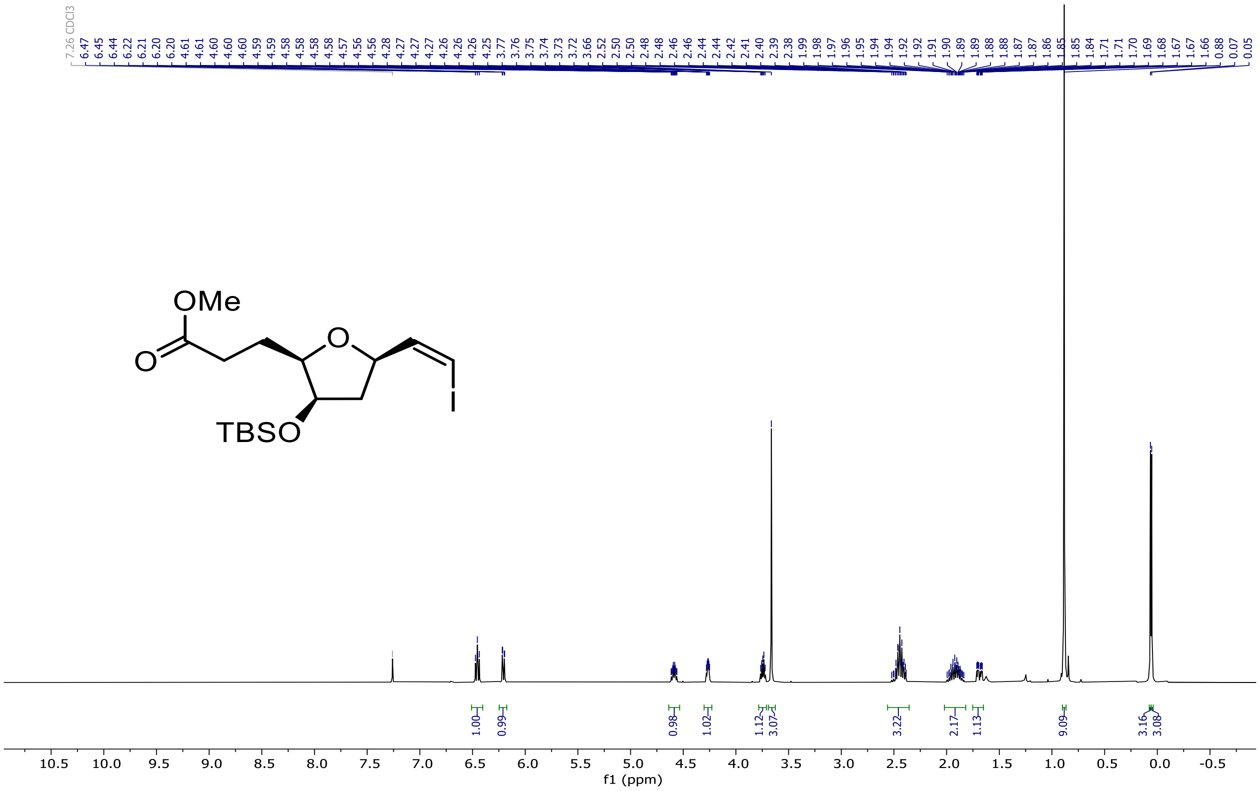

^{13}C NMR (101 MHz, $CDCl_3$)

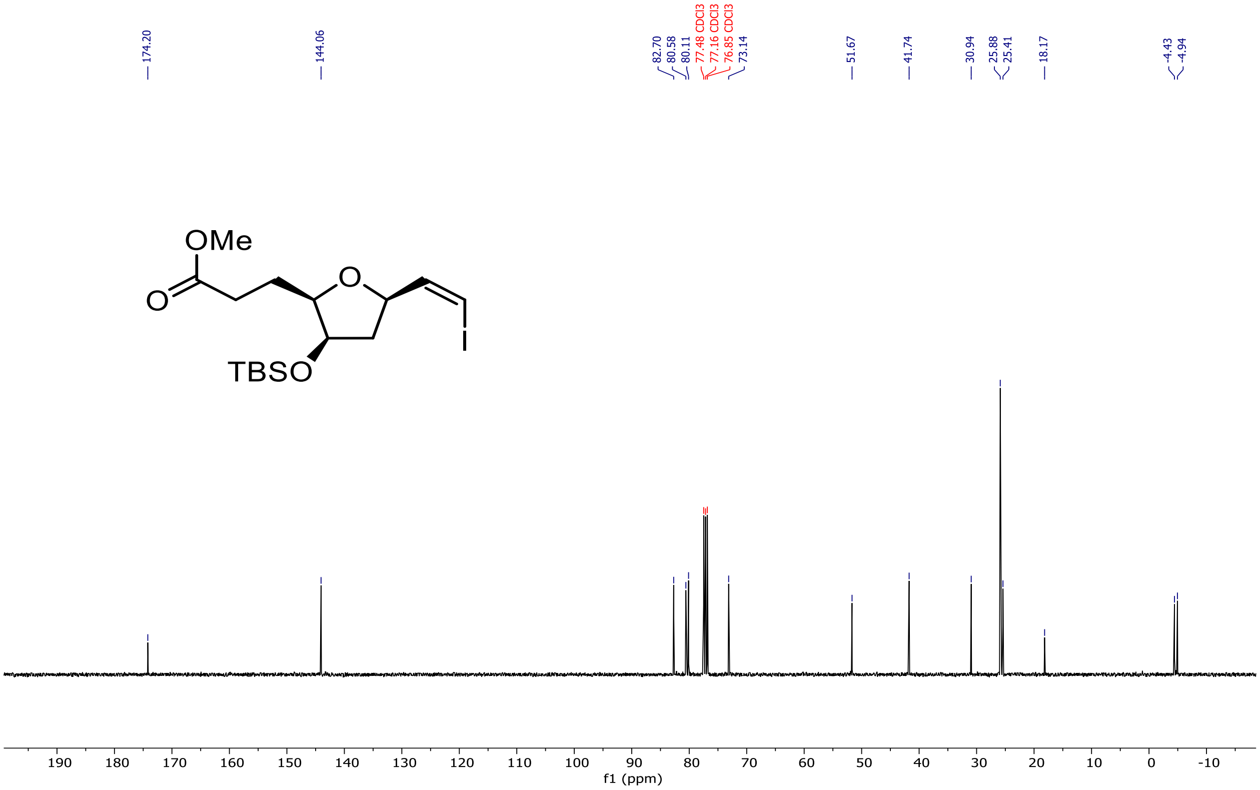

Compound 40: ^{1}H NMR (600 MHz, $CDCl_3$)

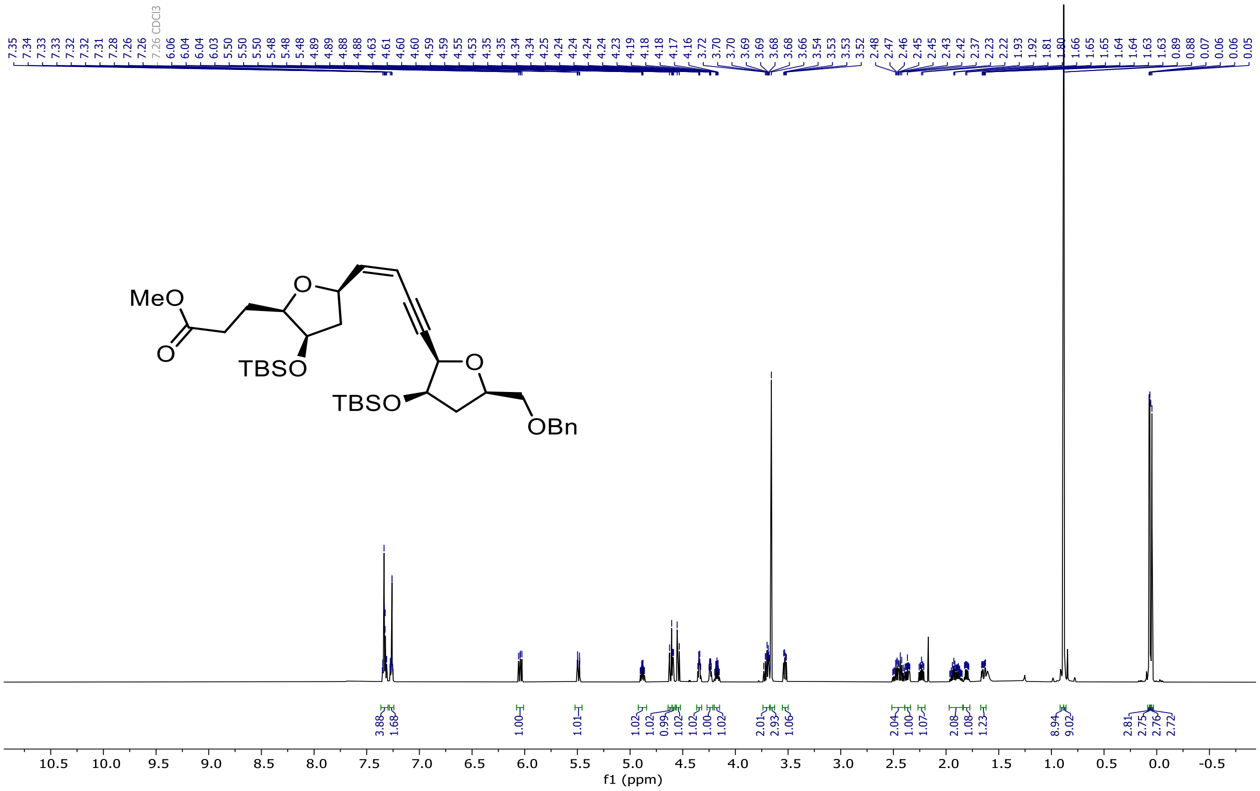

^{13}C NMR (151 MHz, $CDCl_3$)

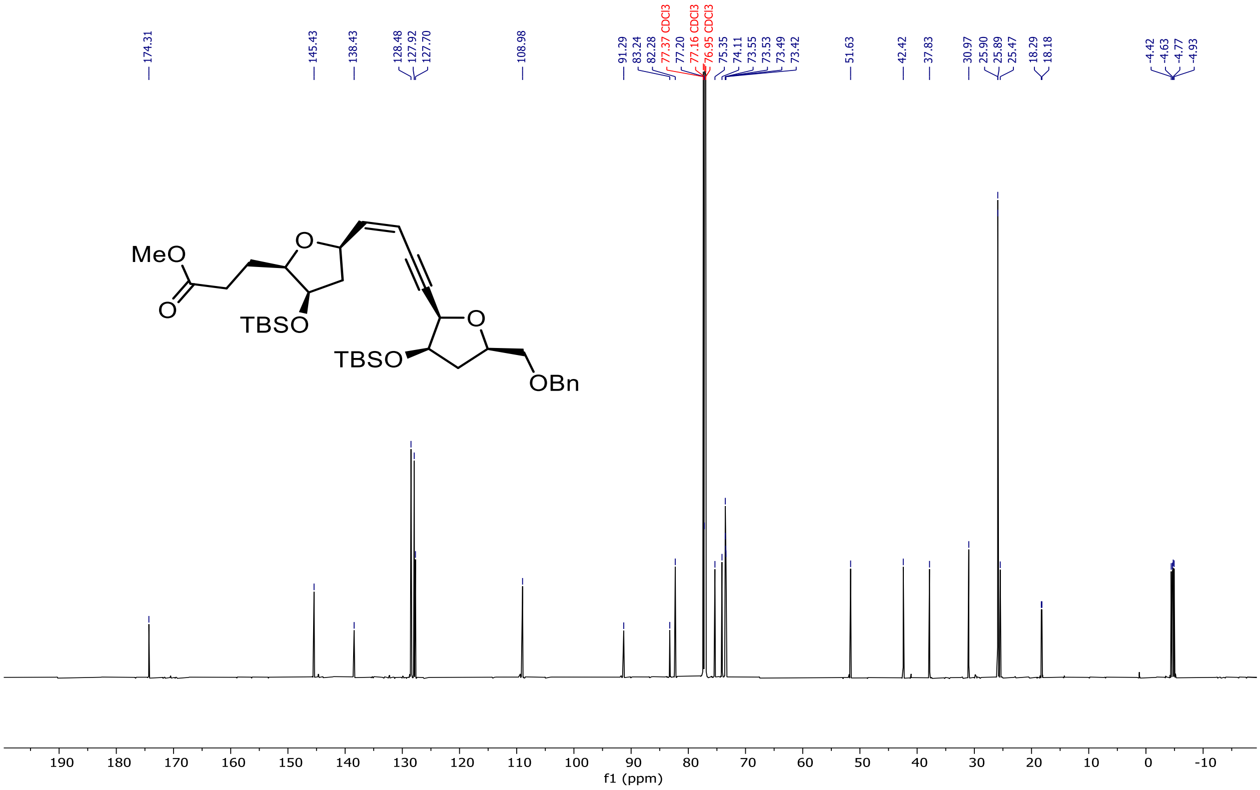

Compound 40: ^1H-^1H COSY ($CDCl_3$)

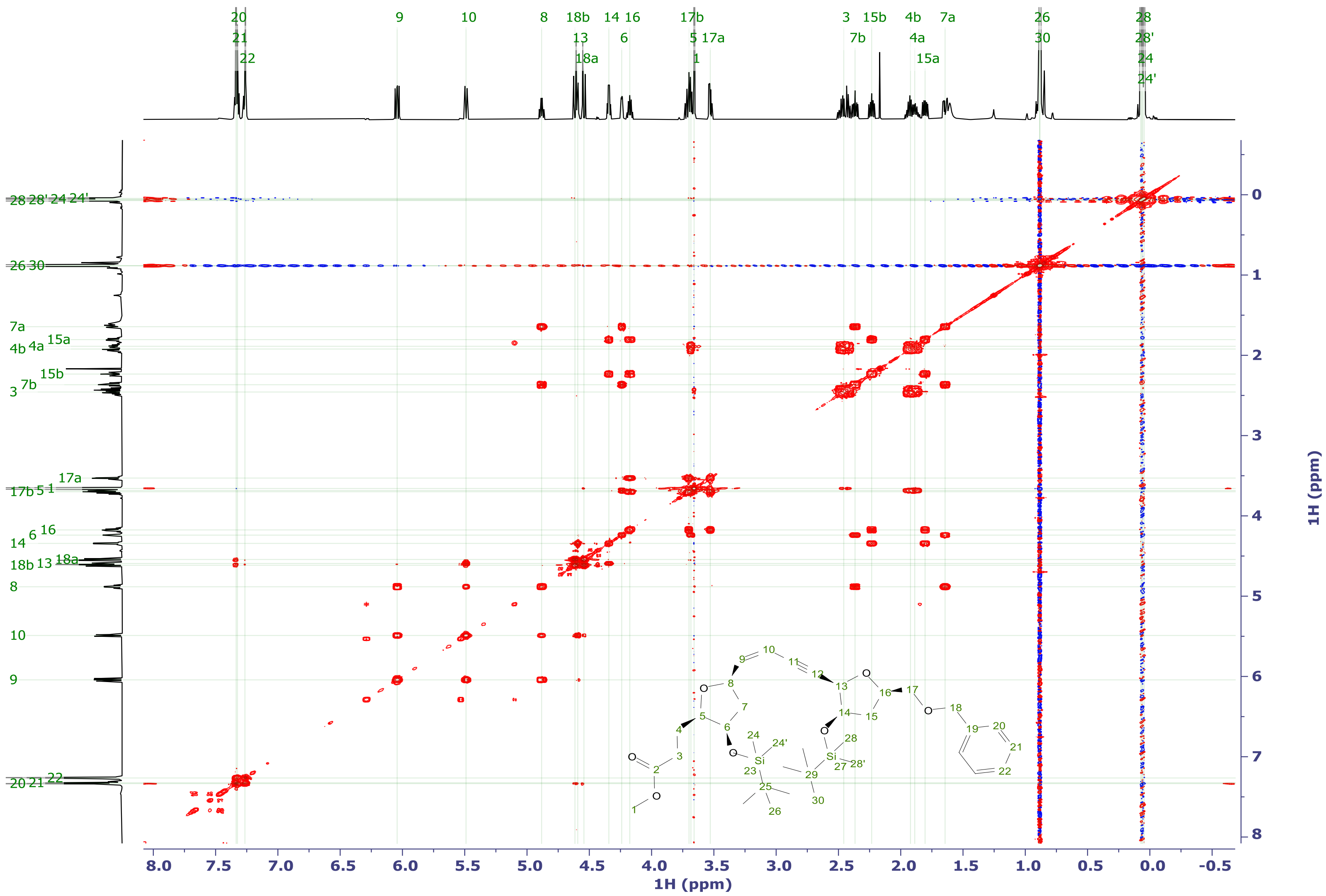

Compound 40: HSQC NMR ($CDCl_3$)

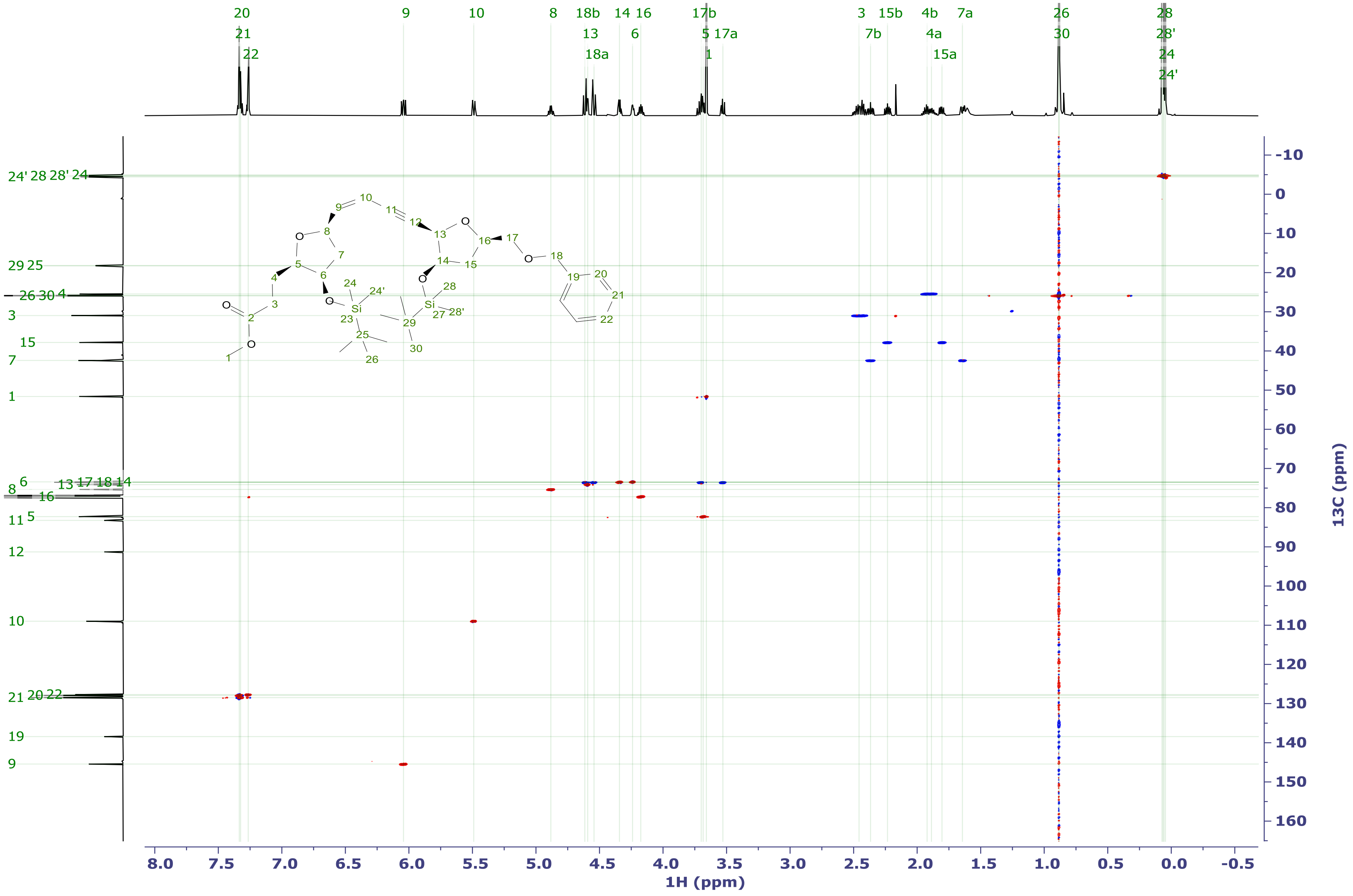

Compound 40: HMBC NMR ($CDCl_3$)

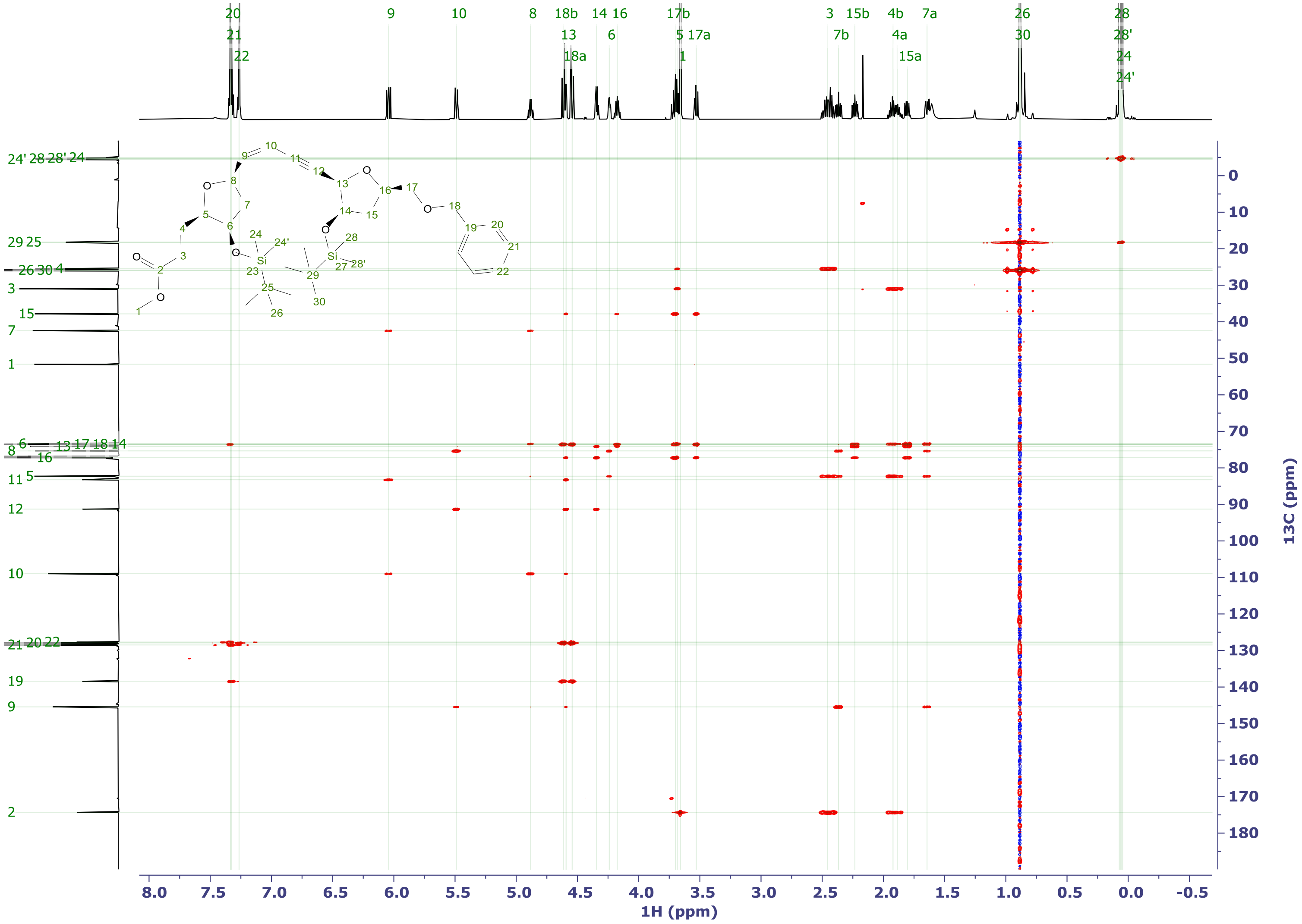

Compound 40: NOESY ($CDCl_3$)

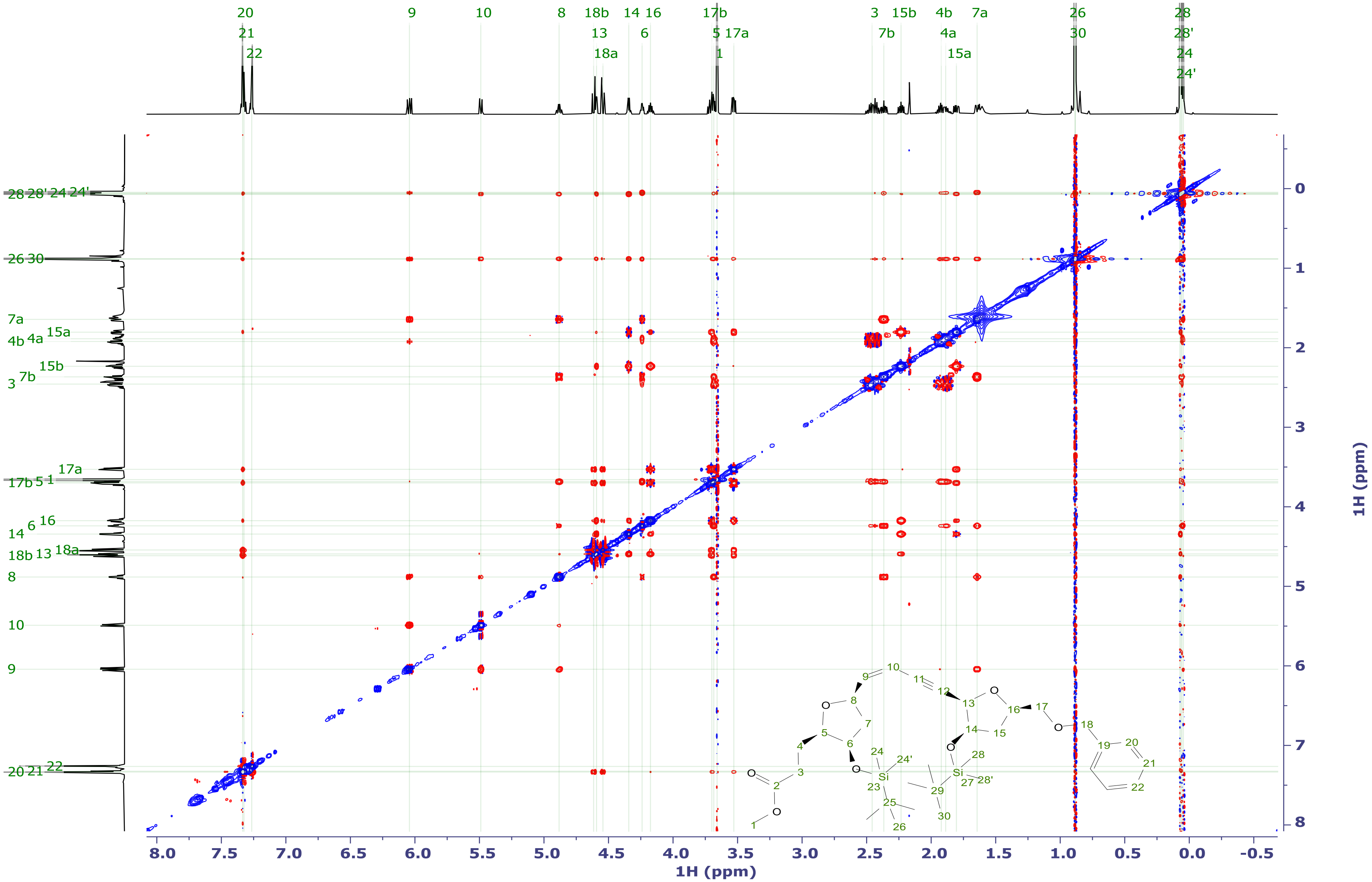

Compound 41: ^{1}H NMR (400 MHz, $CDCl_3$)

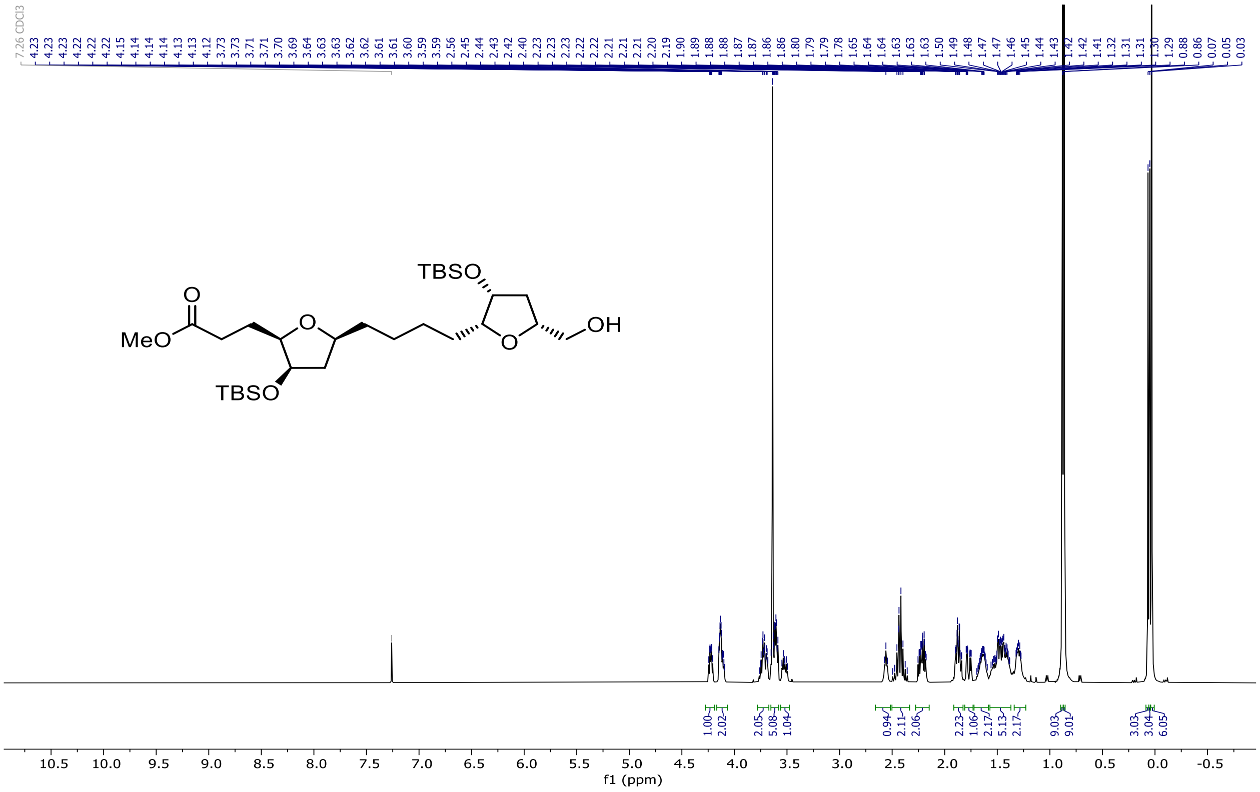

^{13}C NMR (101 MHz, $CDCl_3$)

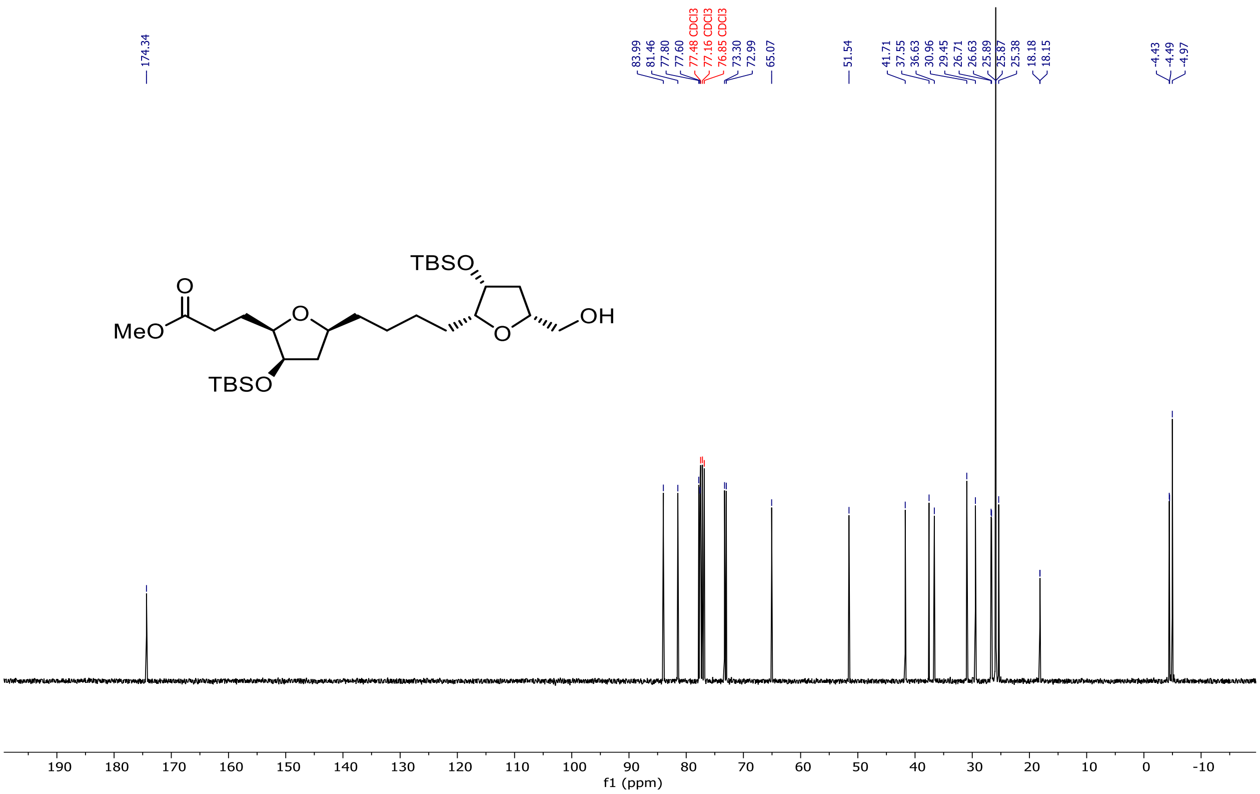

Compound 42: ^{1}H NMR (400 MHz, $CDCl_3$)

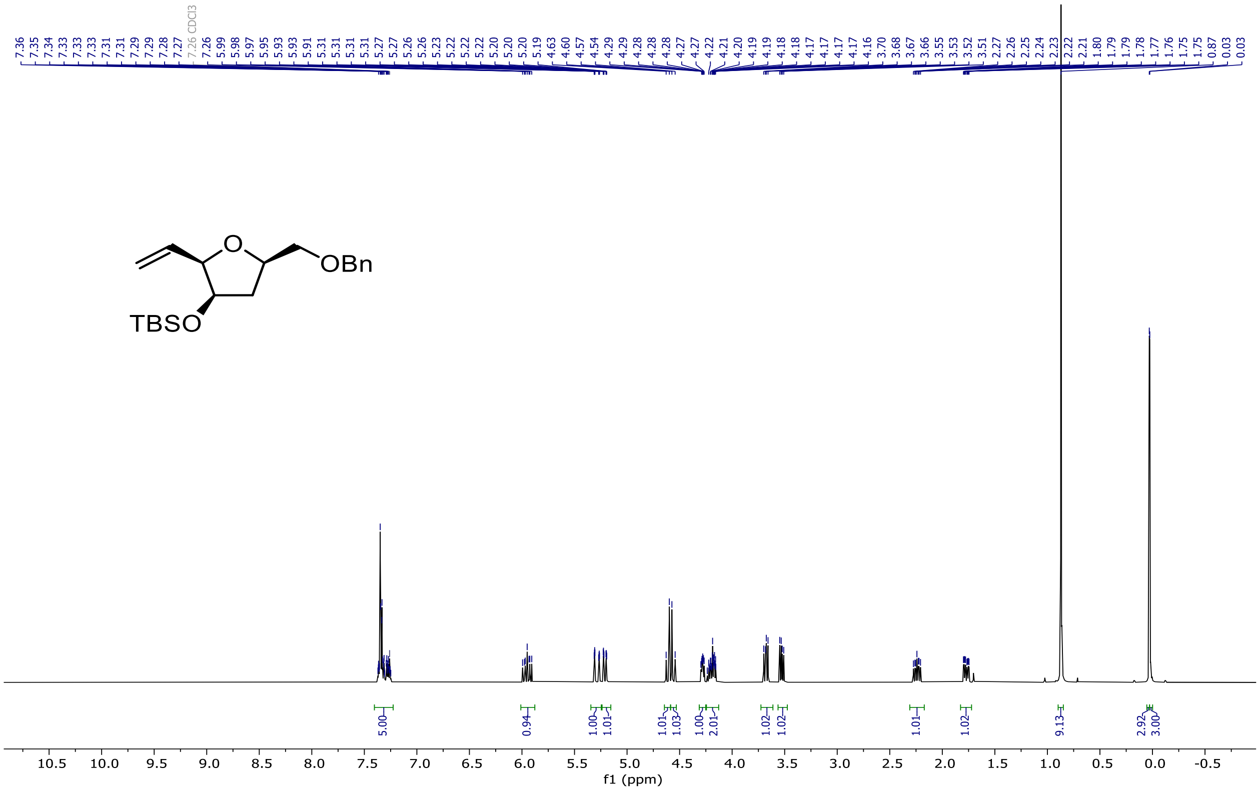

^{13}C NMR (101 MHz, $CDCl_3$)

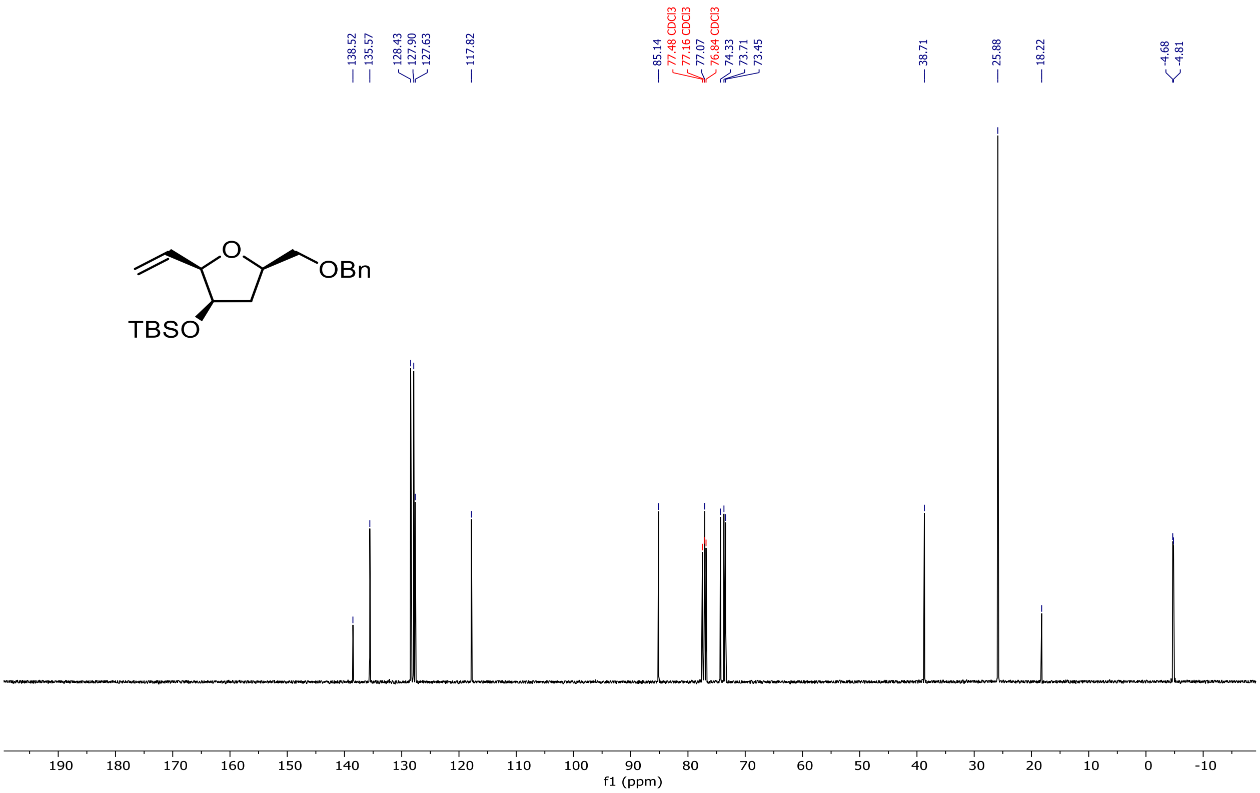

Compound 43: ^1H NMR (400 MHz, $CDCl_3$)

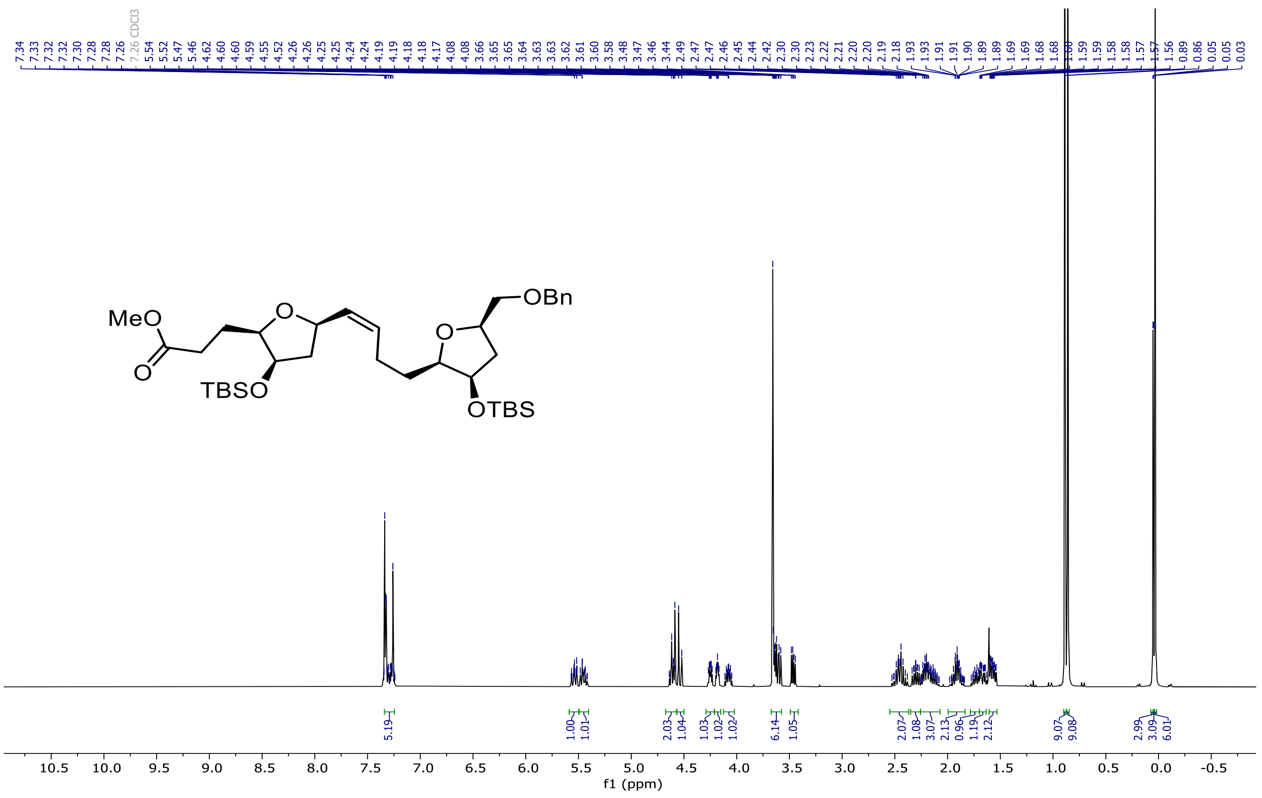

^{13}C NMR (101 MHz, $CDCl_3$)

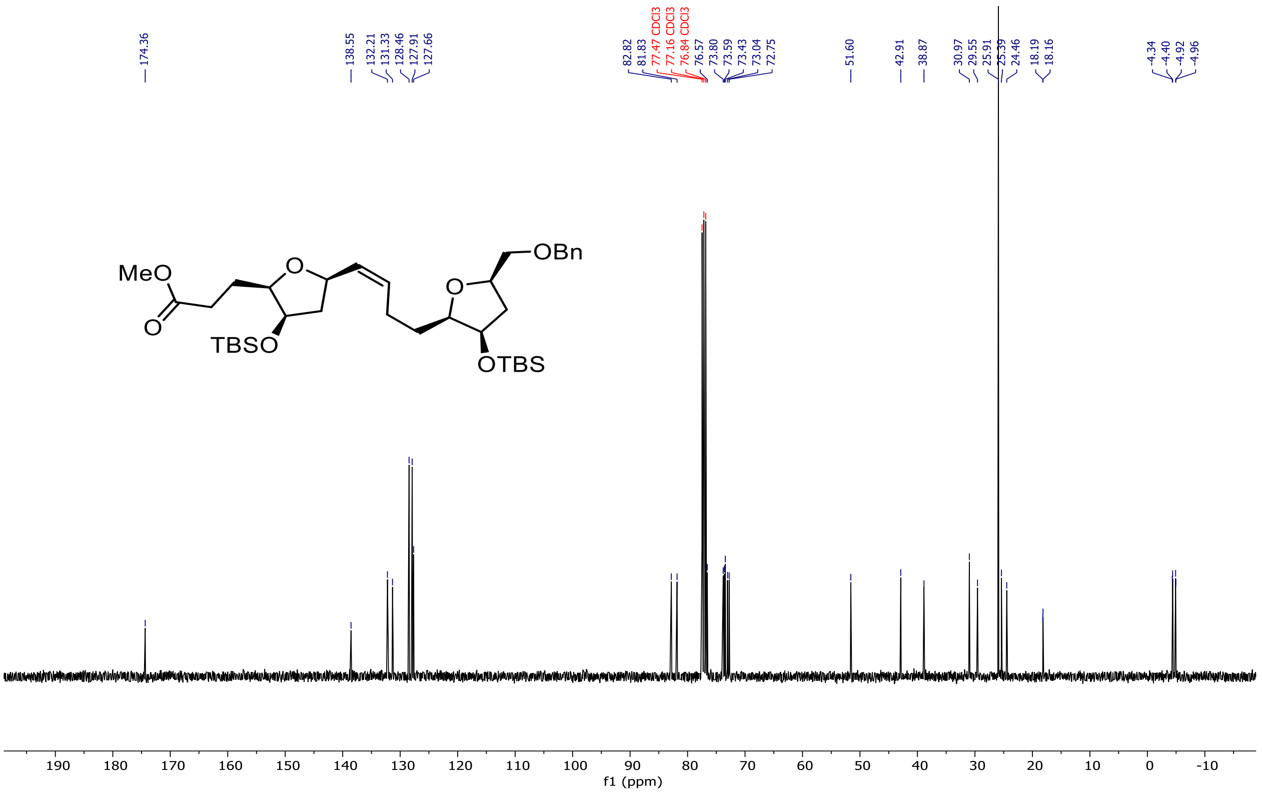

Compound 44: ^{1}H NMR (400 MHz, $CDCl_3$)

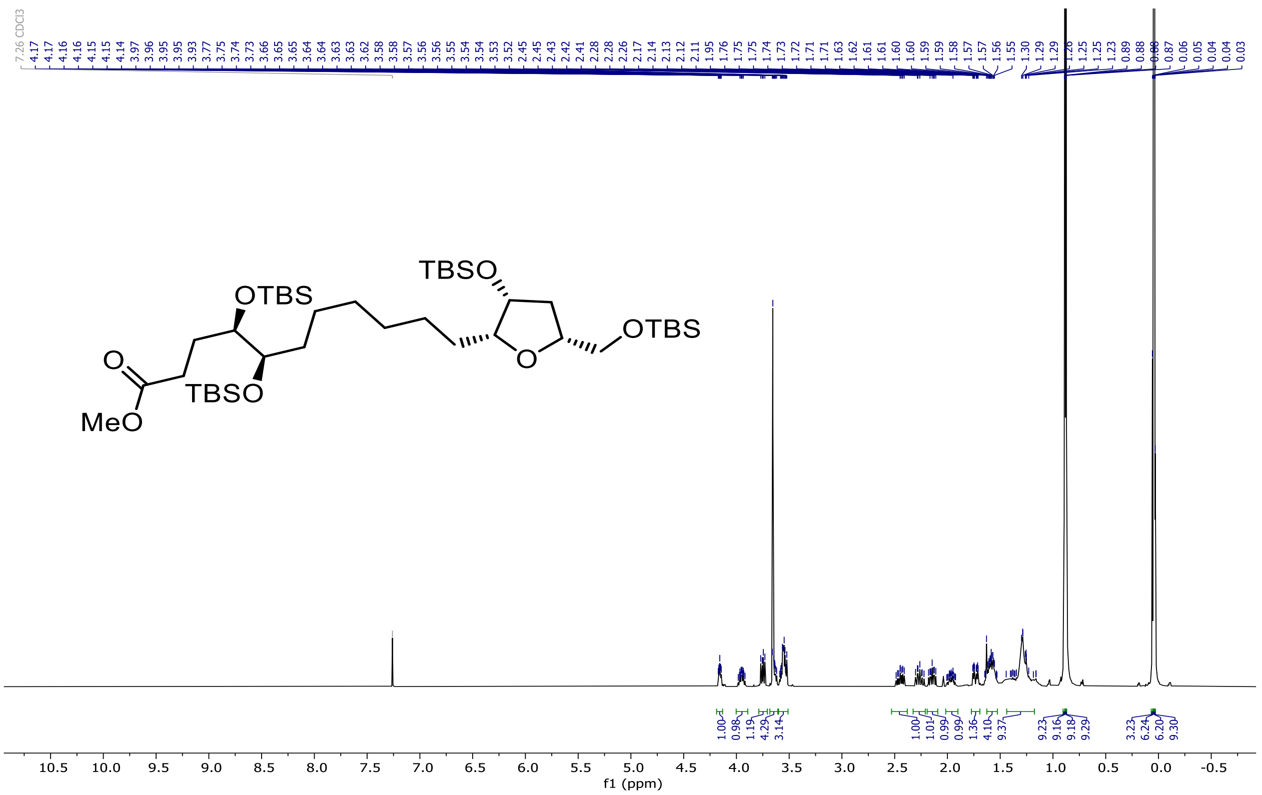

^{13}C NMR (101 MHz, $CDCl_3$)

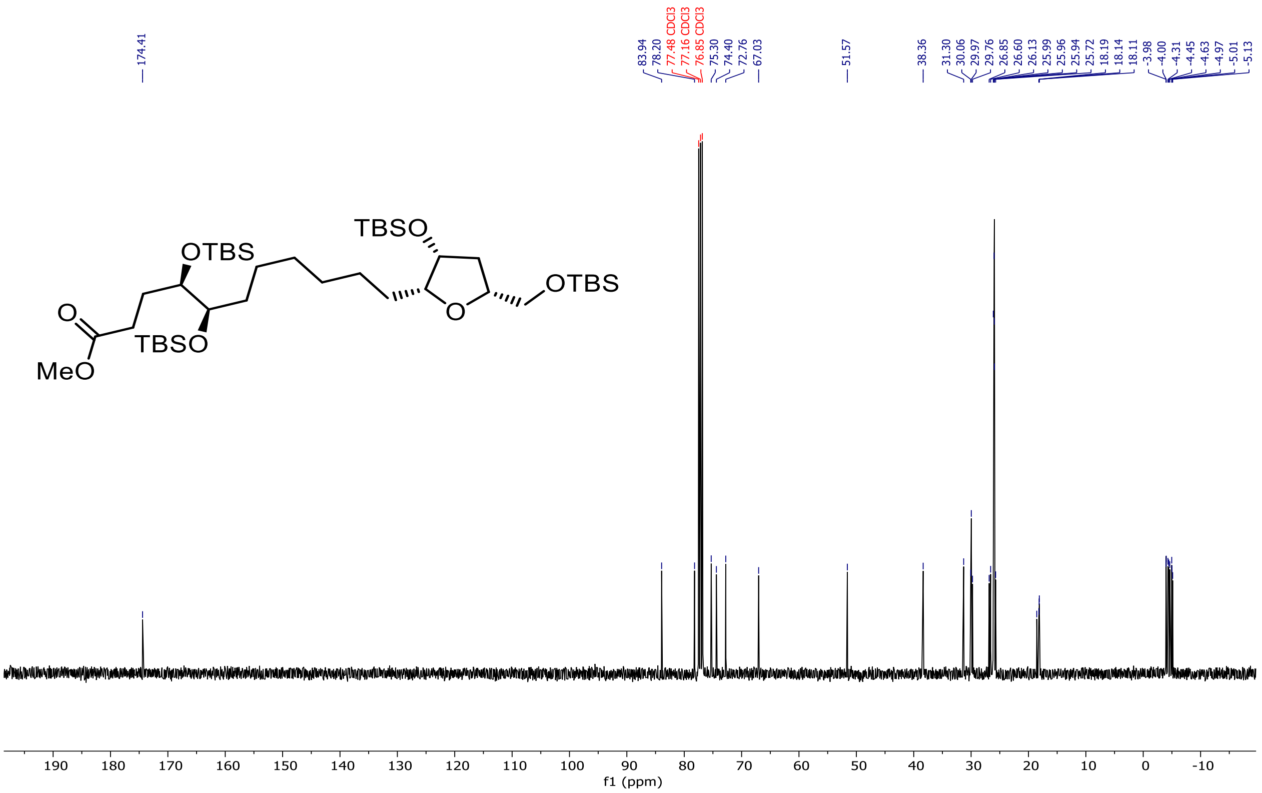

Compound 44: ^{1}H-^{1}H COSY ($CDCl_3$)

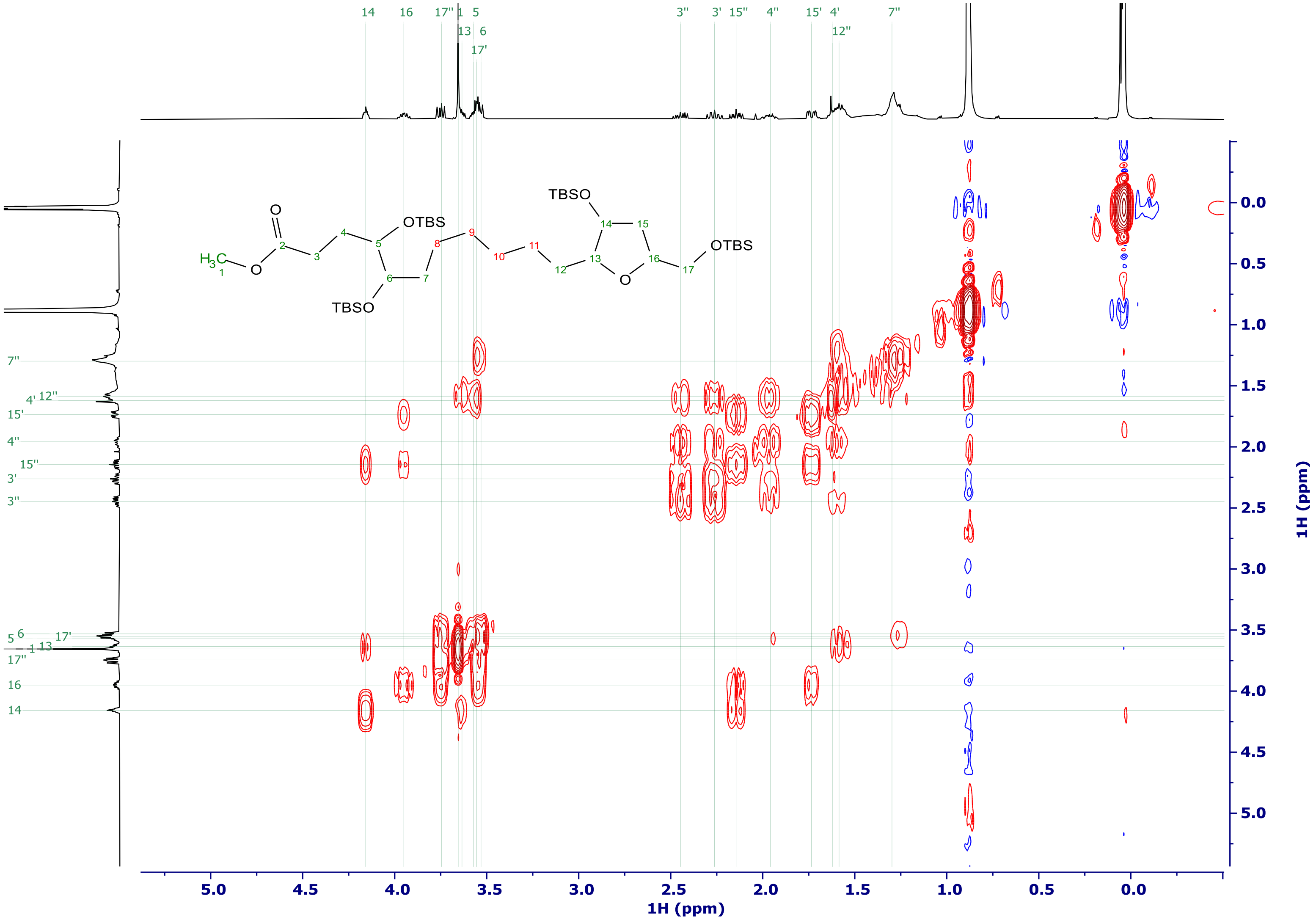

Compound 44: HSQC NMR ($CDCl_3$)

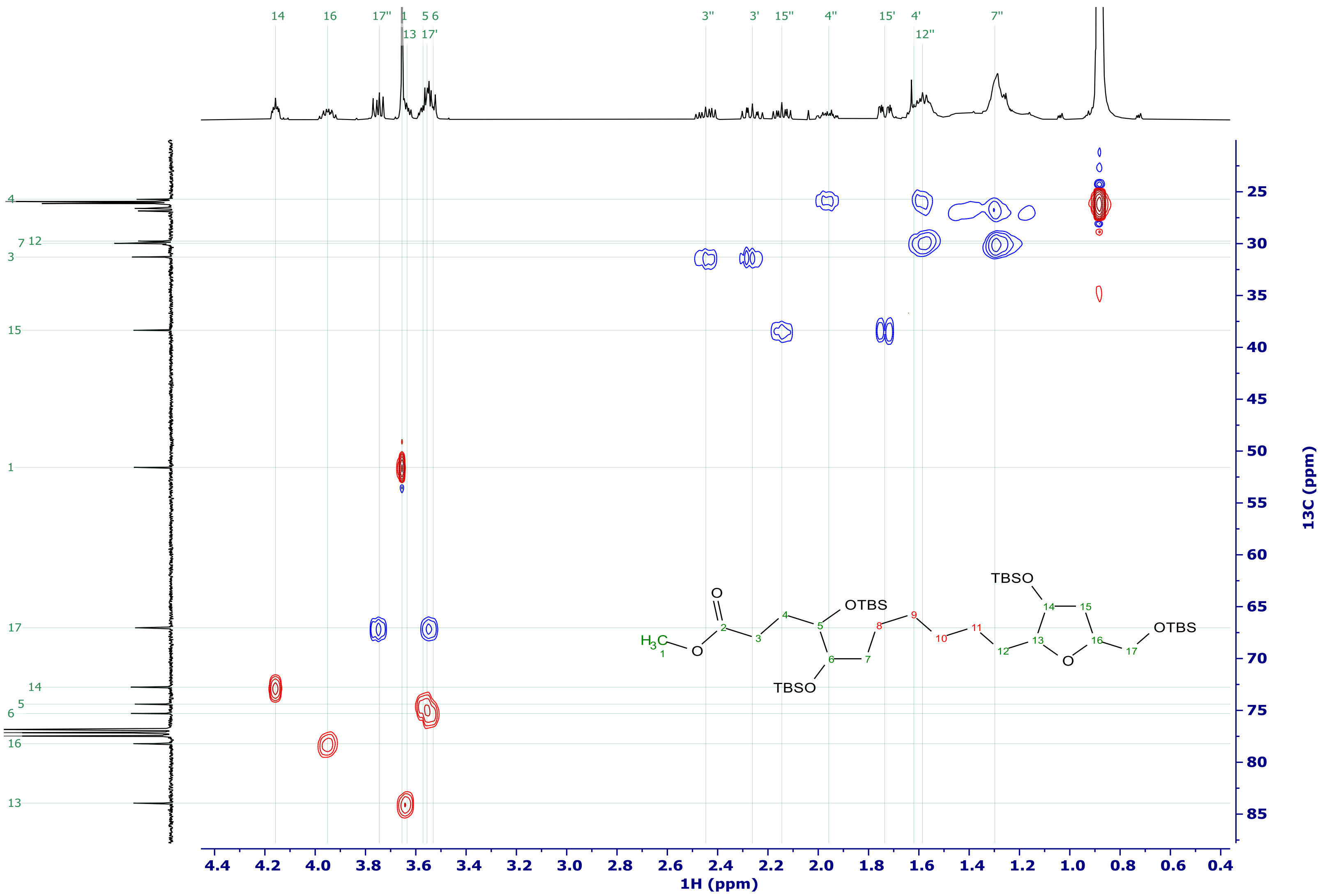

Compound 44: HMBC NMR ($CDCl_3$)

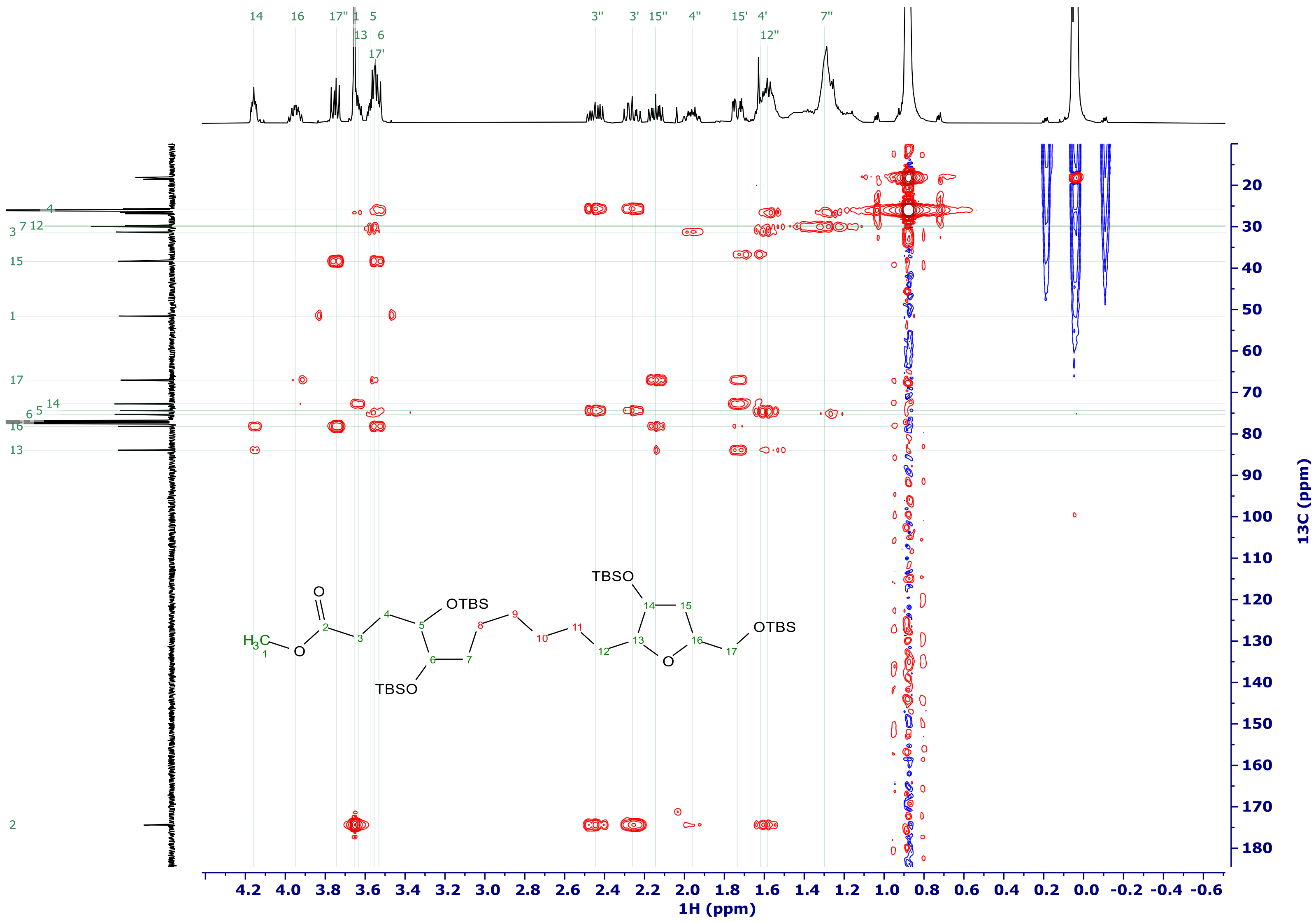

Compound 45: ^{1}H NMR (400 MHz, $CDCl_3$)

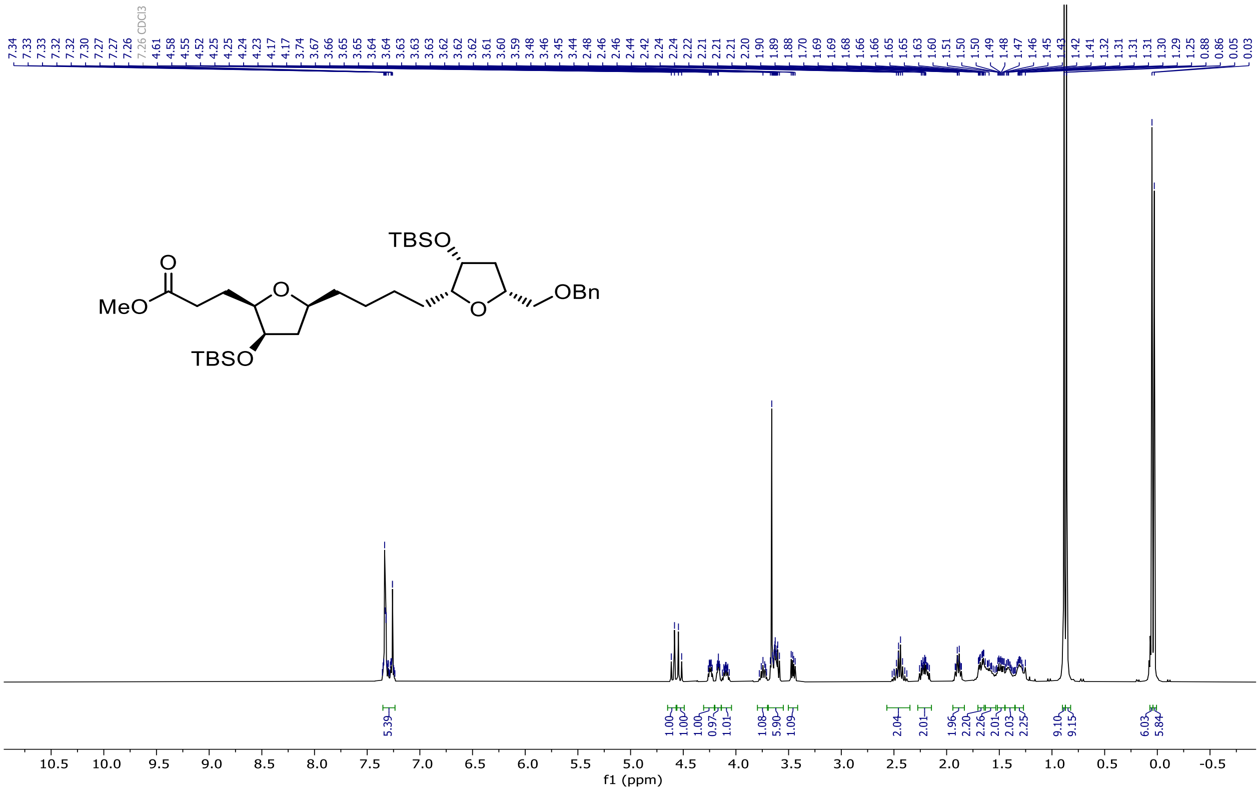

^{13}C NMR (101 MHz, $CDCl_3$)

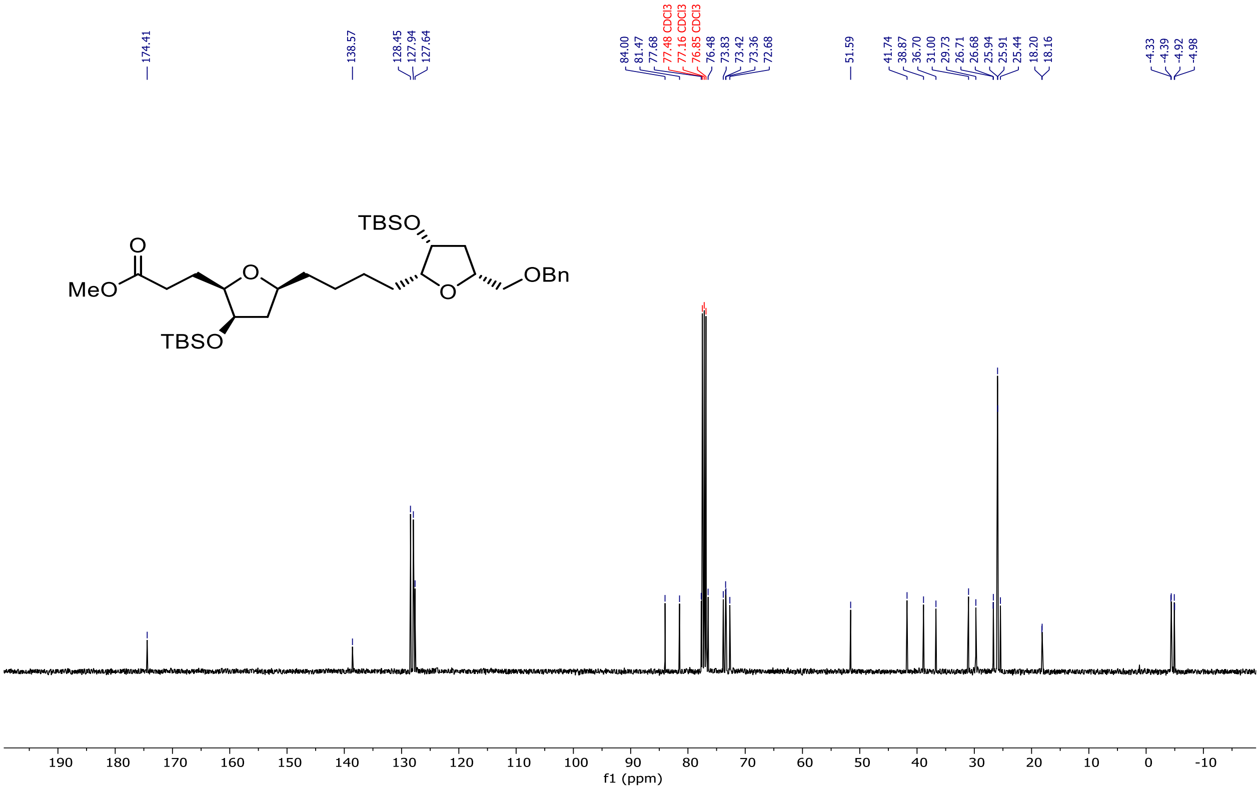

Compound 46a: ^{1}H NMR (400 MHz, $CDCl_3$)

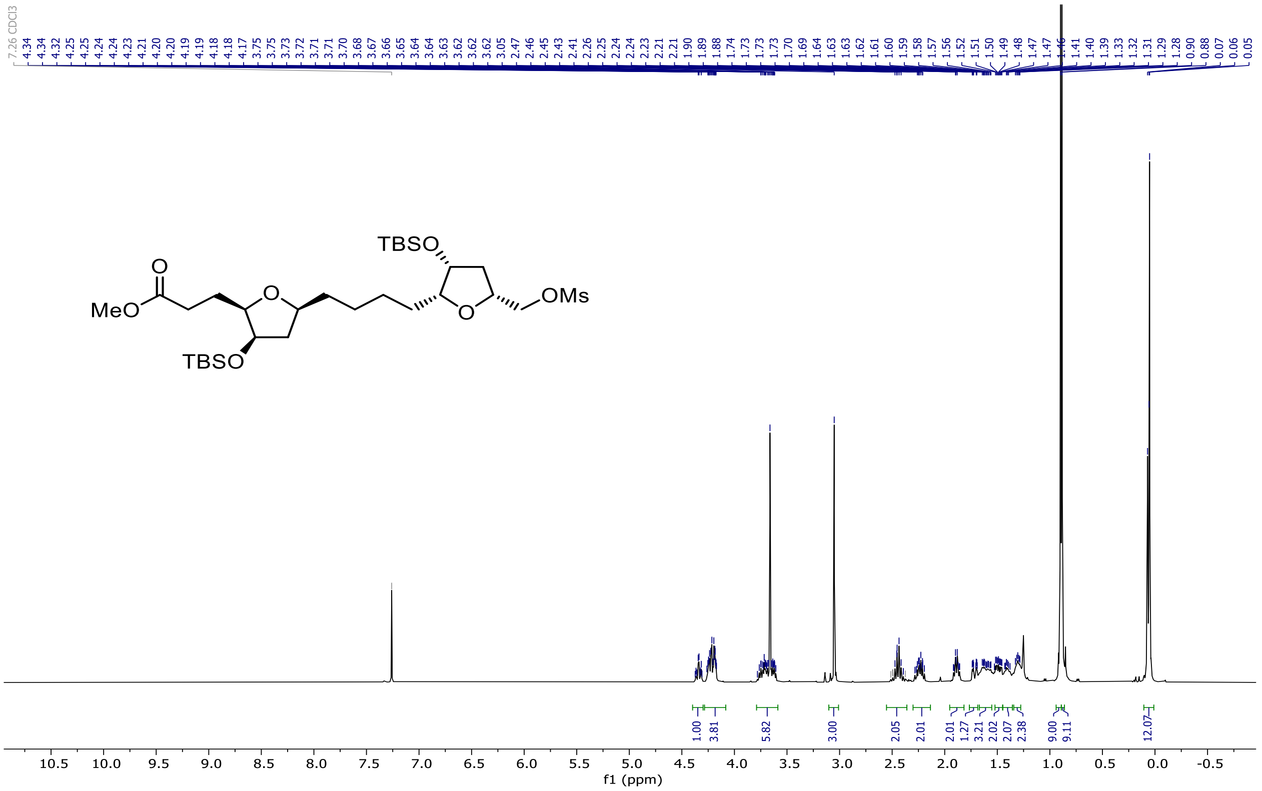

^{13}C NMR (101 MHz, $CDCl_3$)

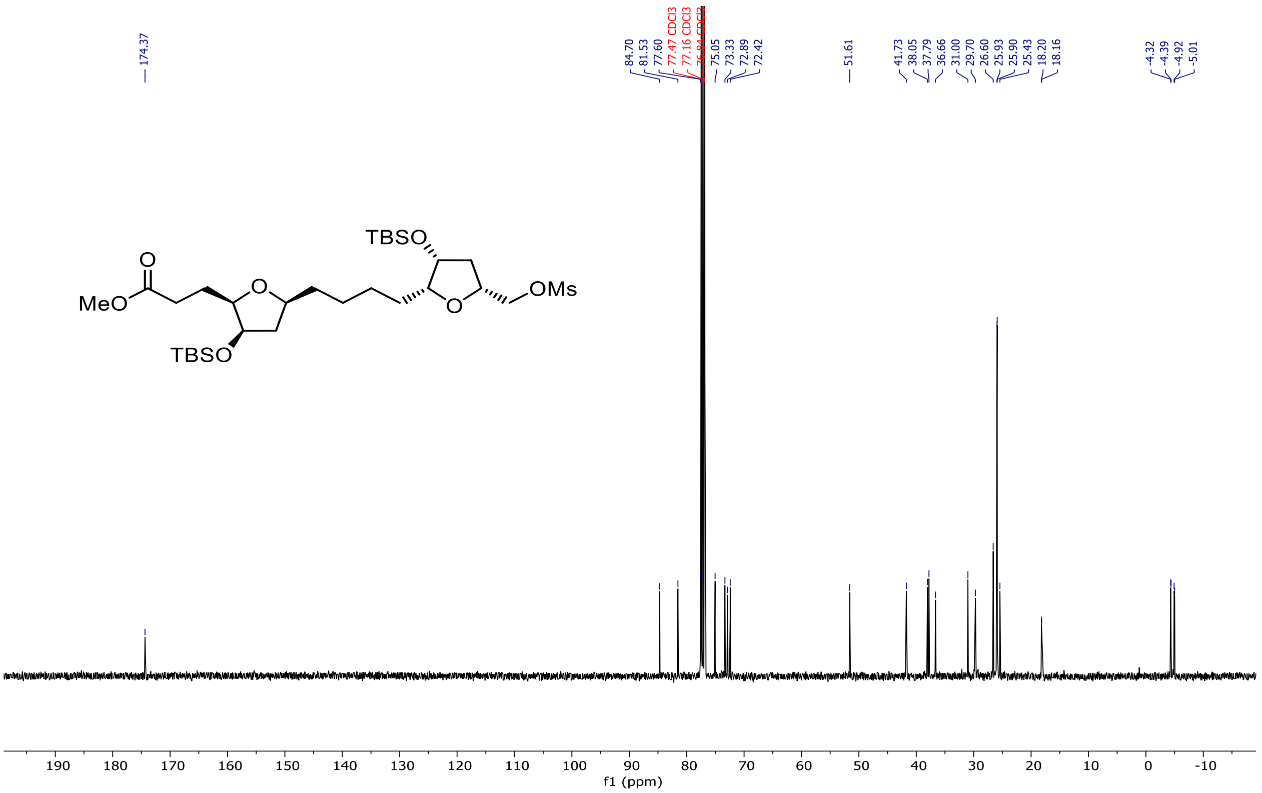

Compound 46: ^{1}H NMR (400 MHz, $CDCl_3$)

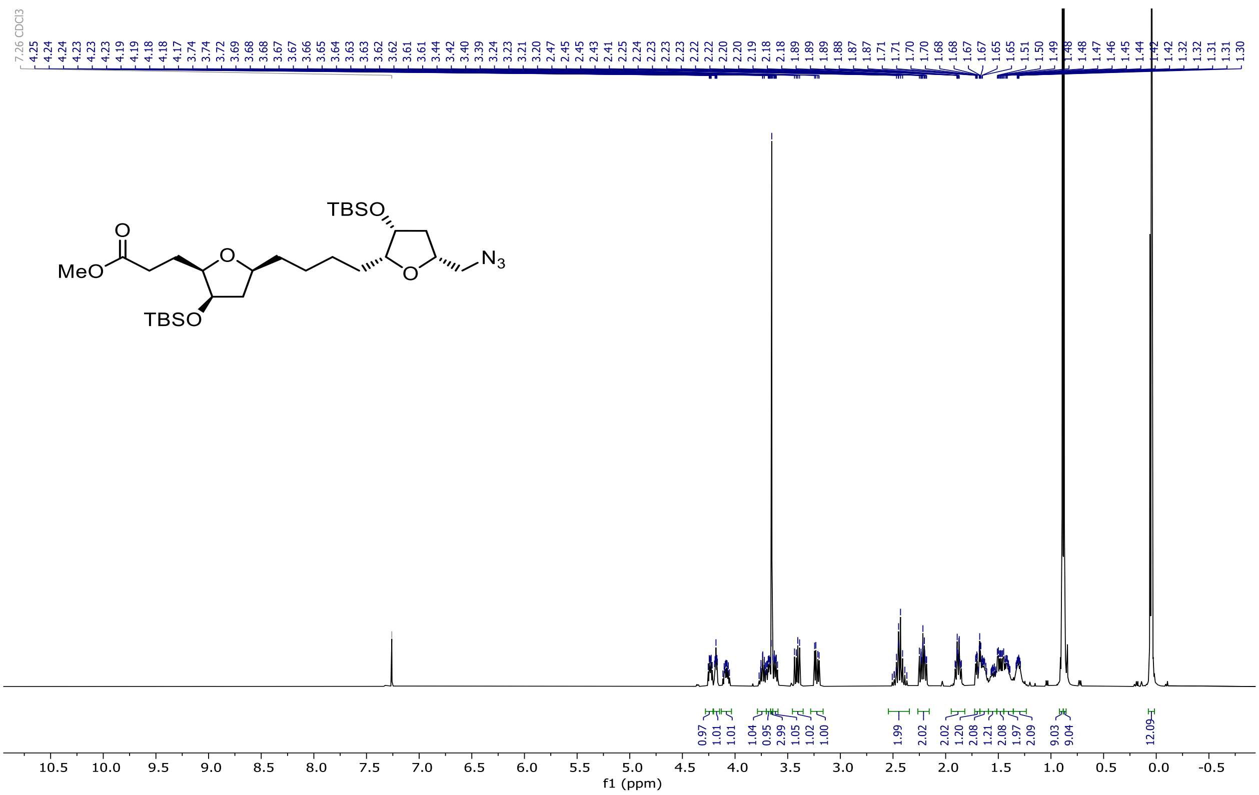

^{13}C NMR (101 MHz, $CDCl_3$)

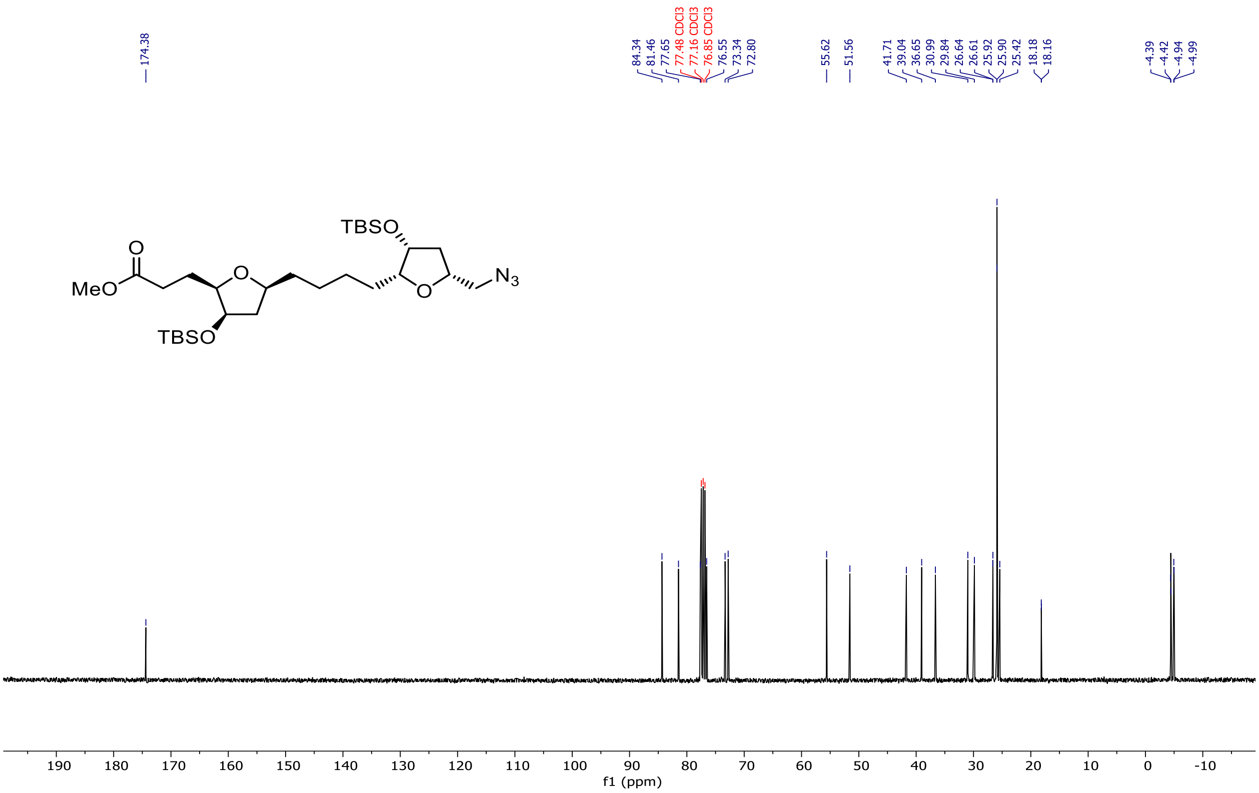

Compound S16: ^{1}H NMR (400 MHz, $CDCl_3$)

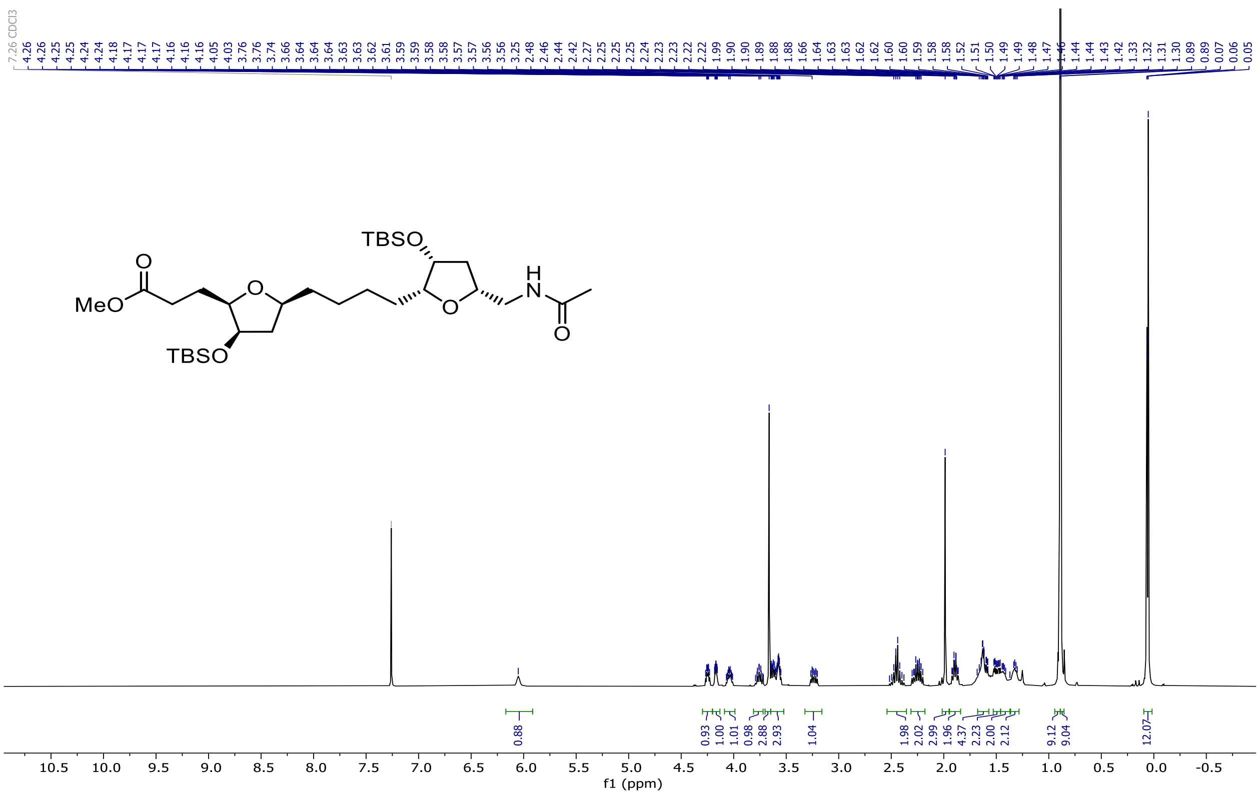

^{13}C NMR (101 MHz, $CDCl_3$)

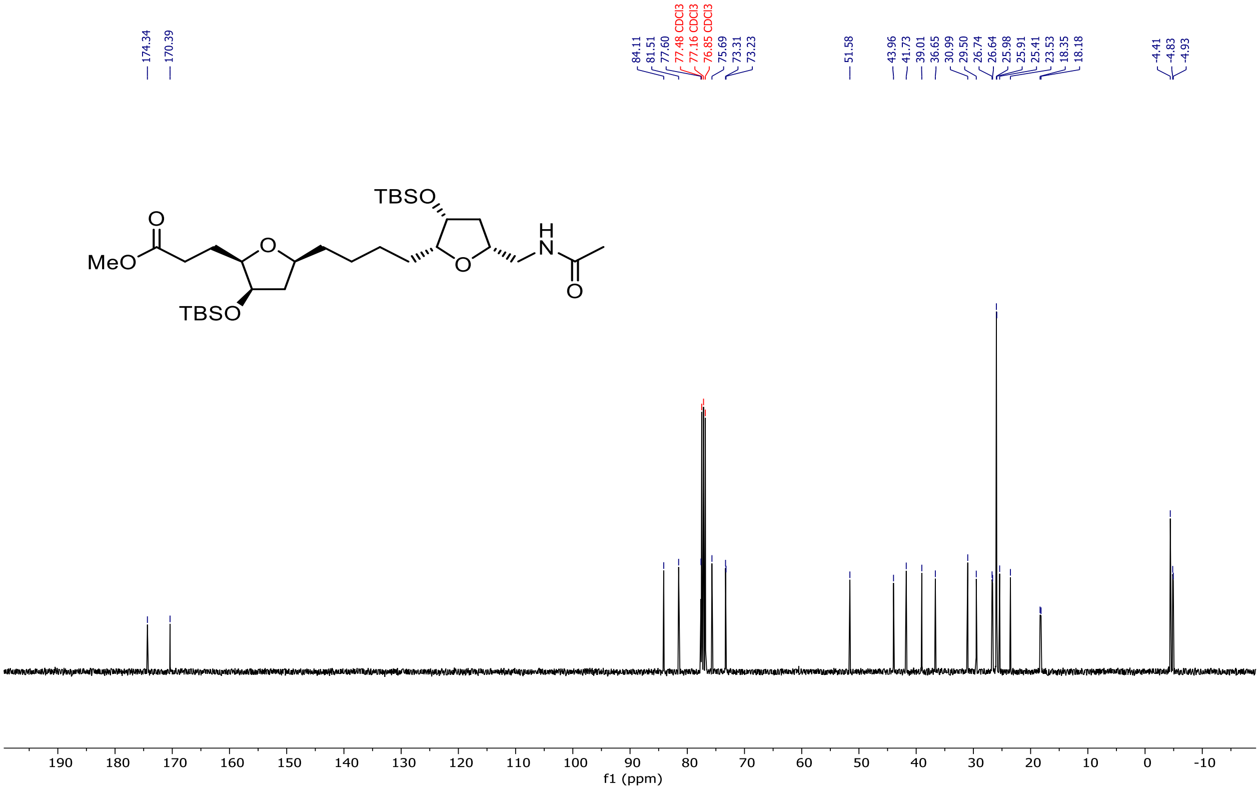

Compound 47: ^{1}H NMR (400 MHz, $CDCl_3$)

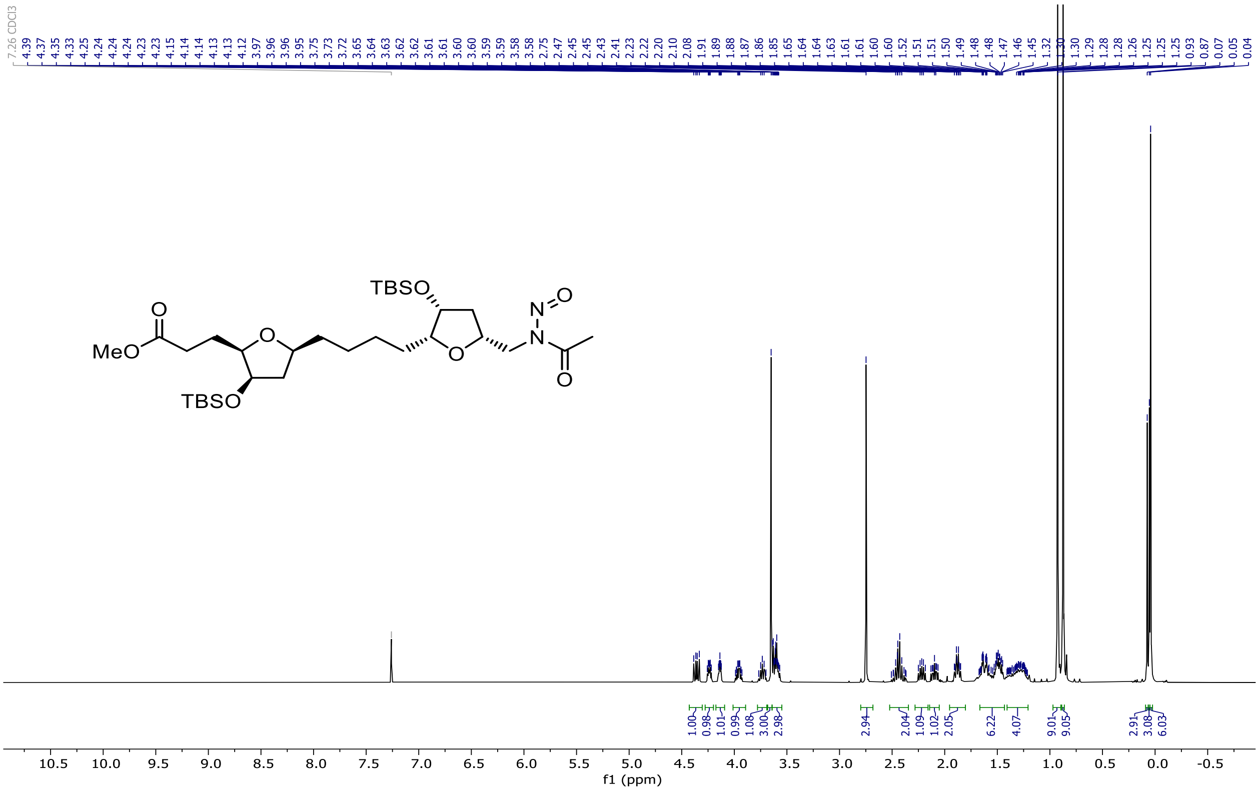

^{13}C NMR (101 MHz, $CDCl_3$)

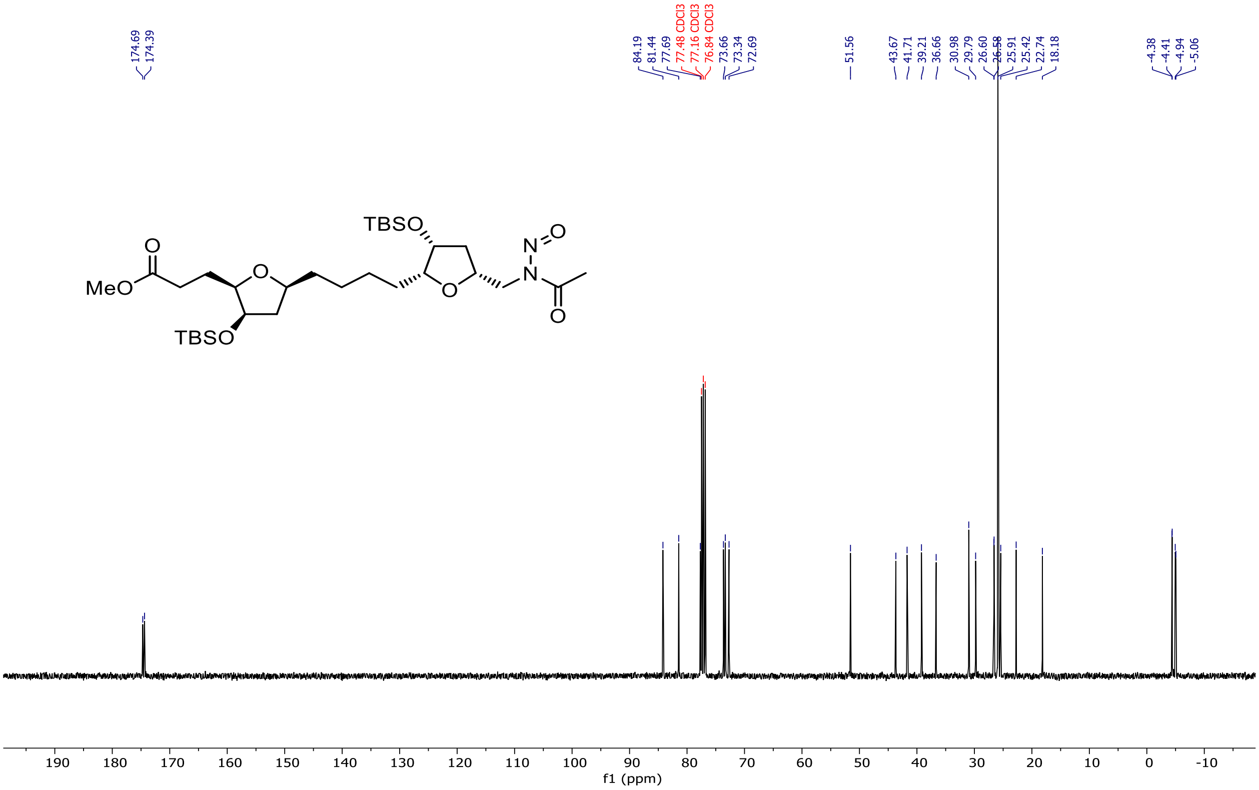

Compound S17: ^{1}H NMR (400 MHz, CDCl$_3$)

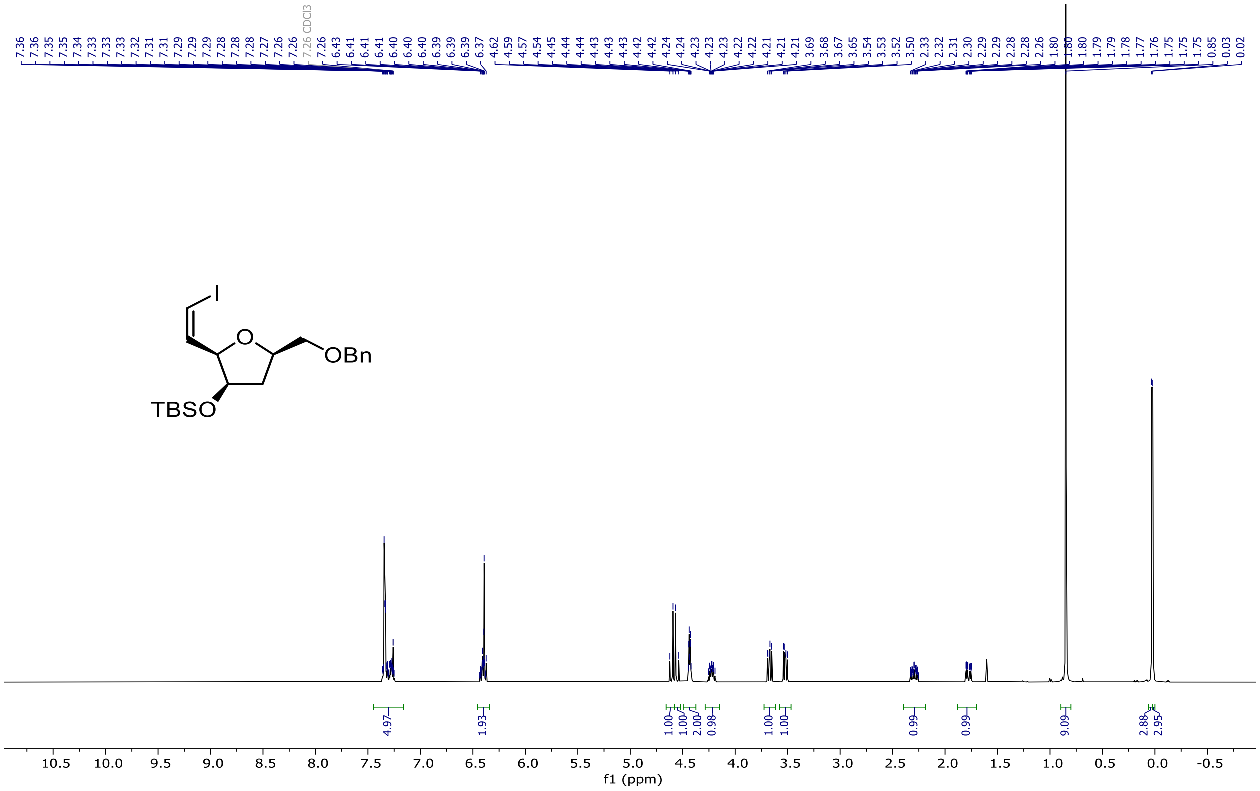

^{13}C NMR (101 MHz, CDCl$_3$)

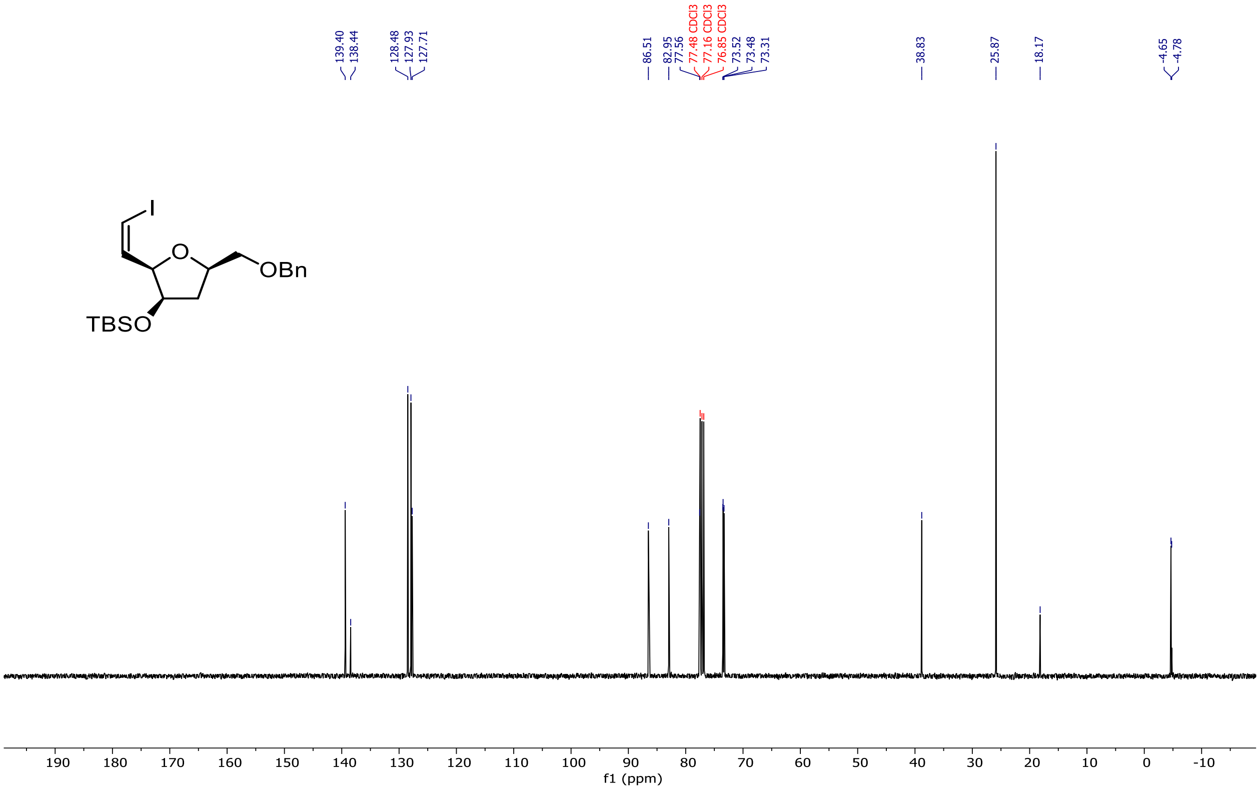

Compound S18: ^{1}H NMR (400 MHz, $CDCl_3$)

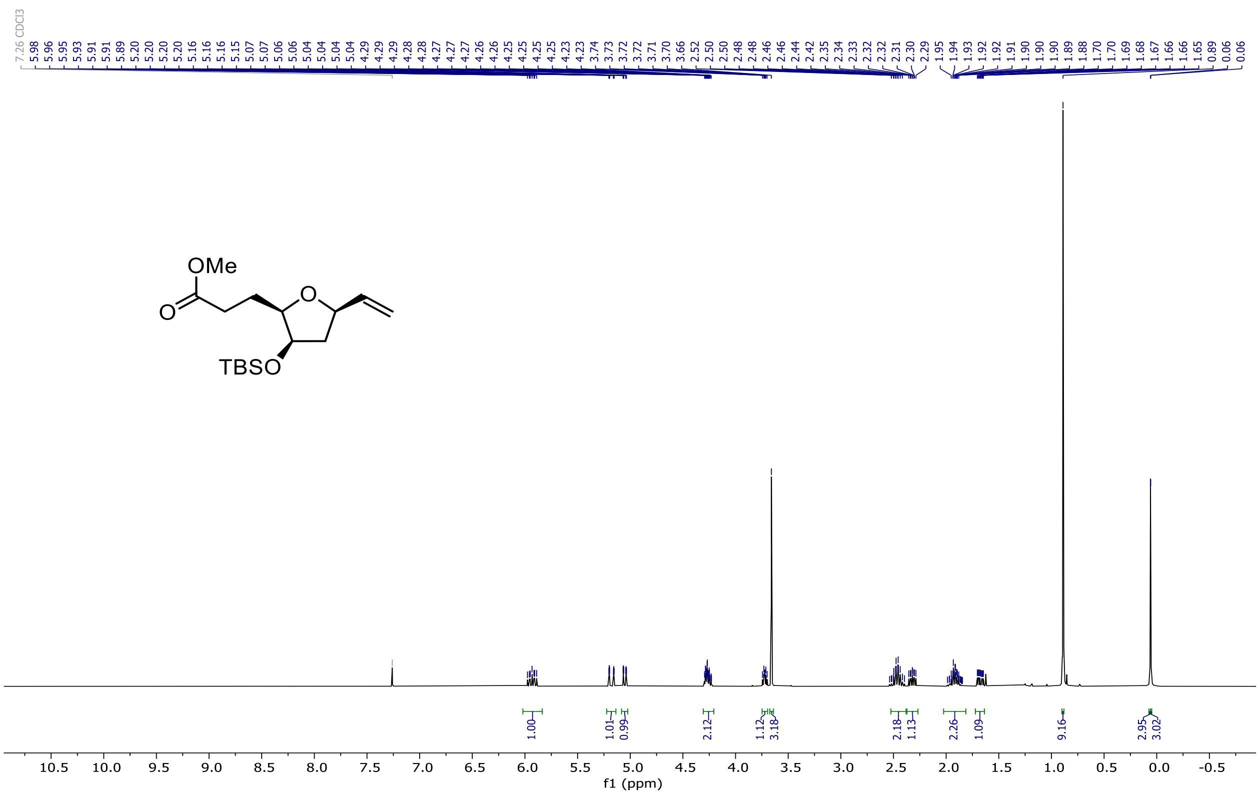

^{13}C NMR (101 MHz, $CDCl_3$)

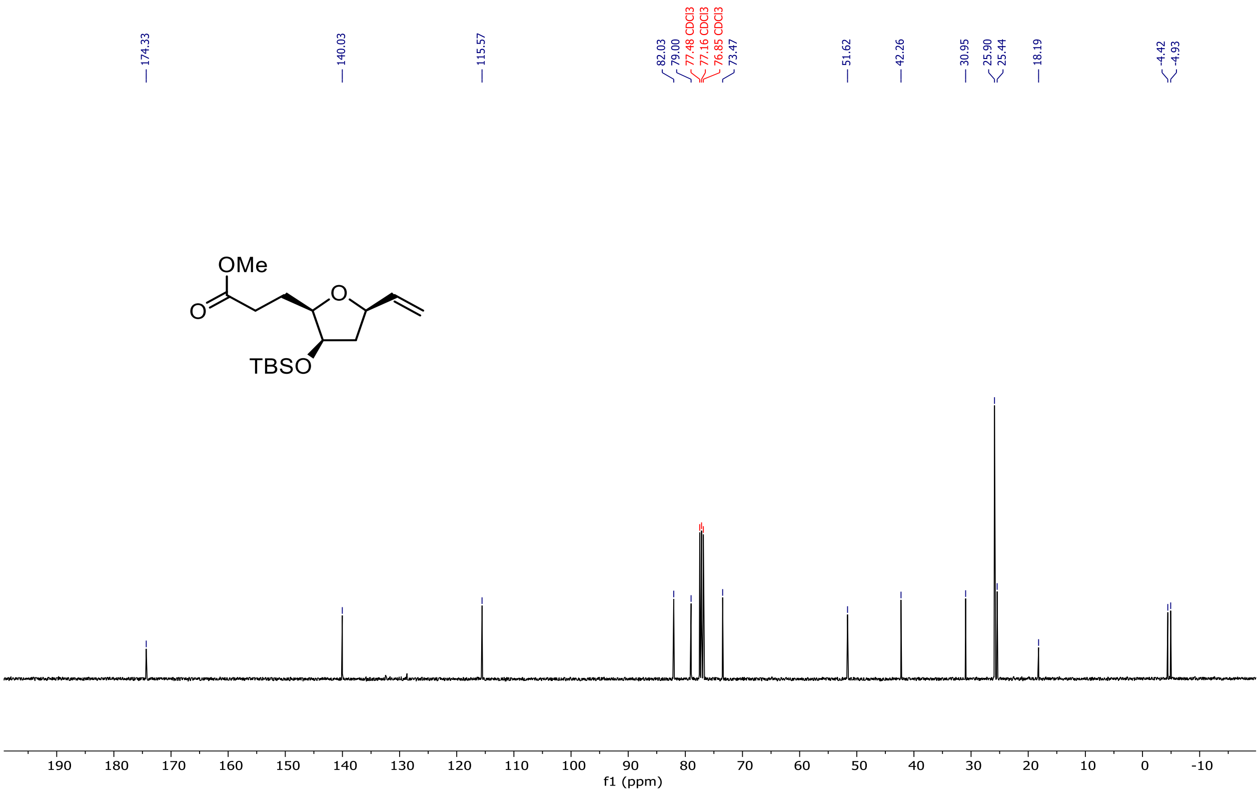

Compound S19: ^{1}H NMR (400 MHz, $CDCl_3$)

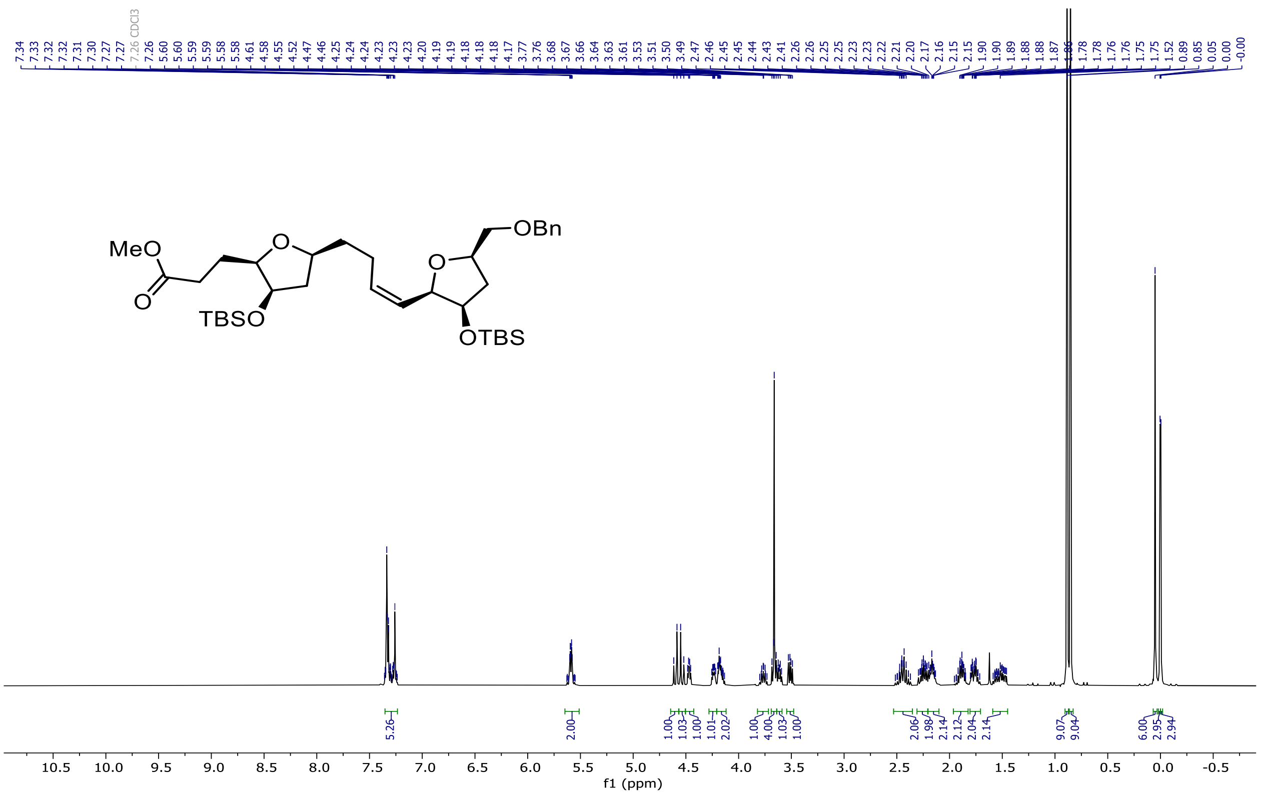

^{13}C NMR (101 MHz, $CDCl_3$)

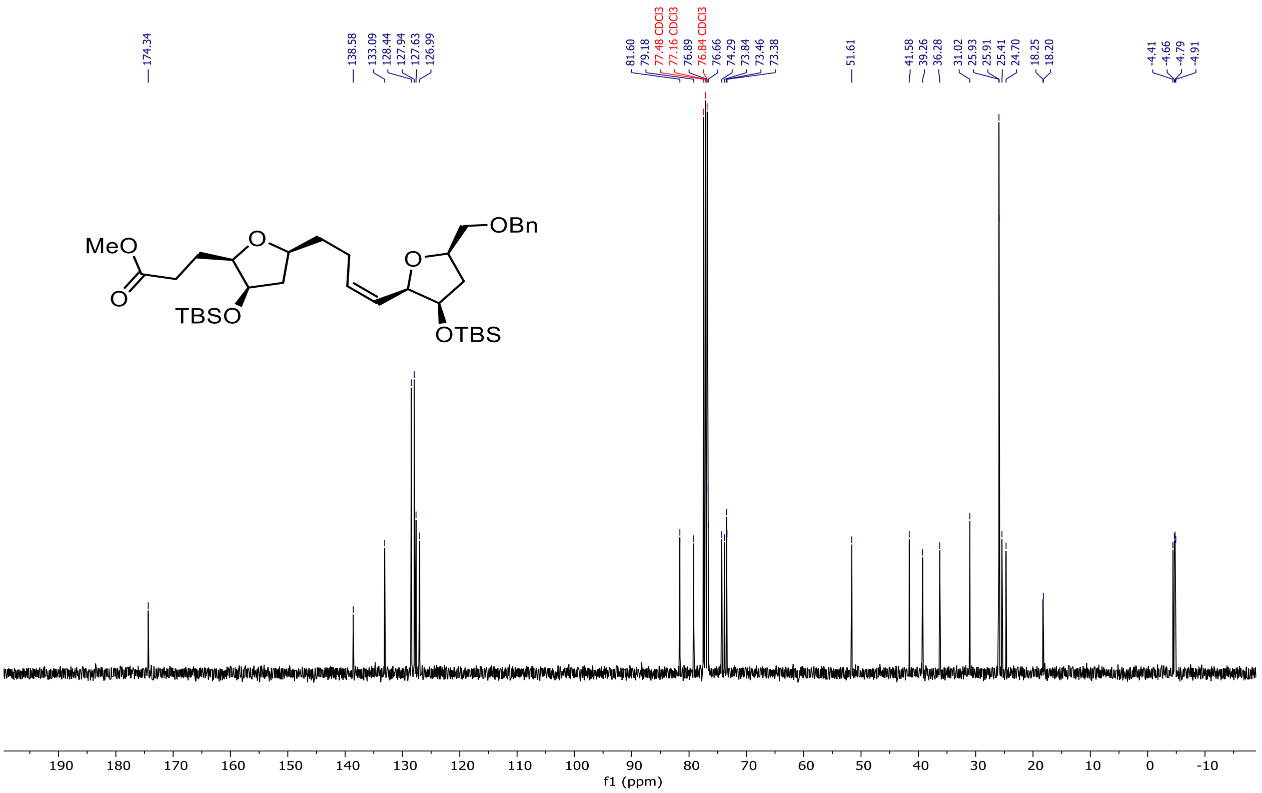

Compound 48: ^{1}H NMR (400 MHz, $CDCl_3$)

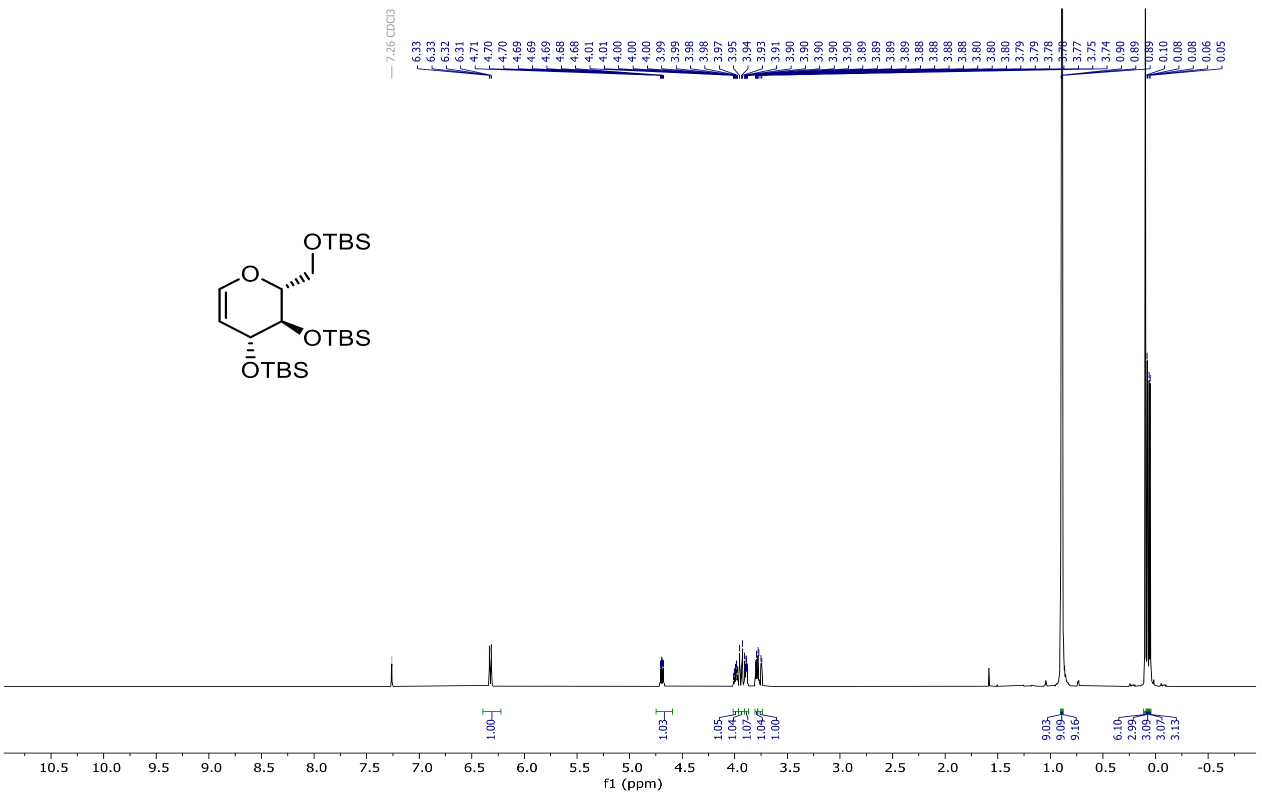

^{13}C NMR (101 MHz, $CDCl_3$)

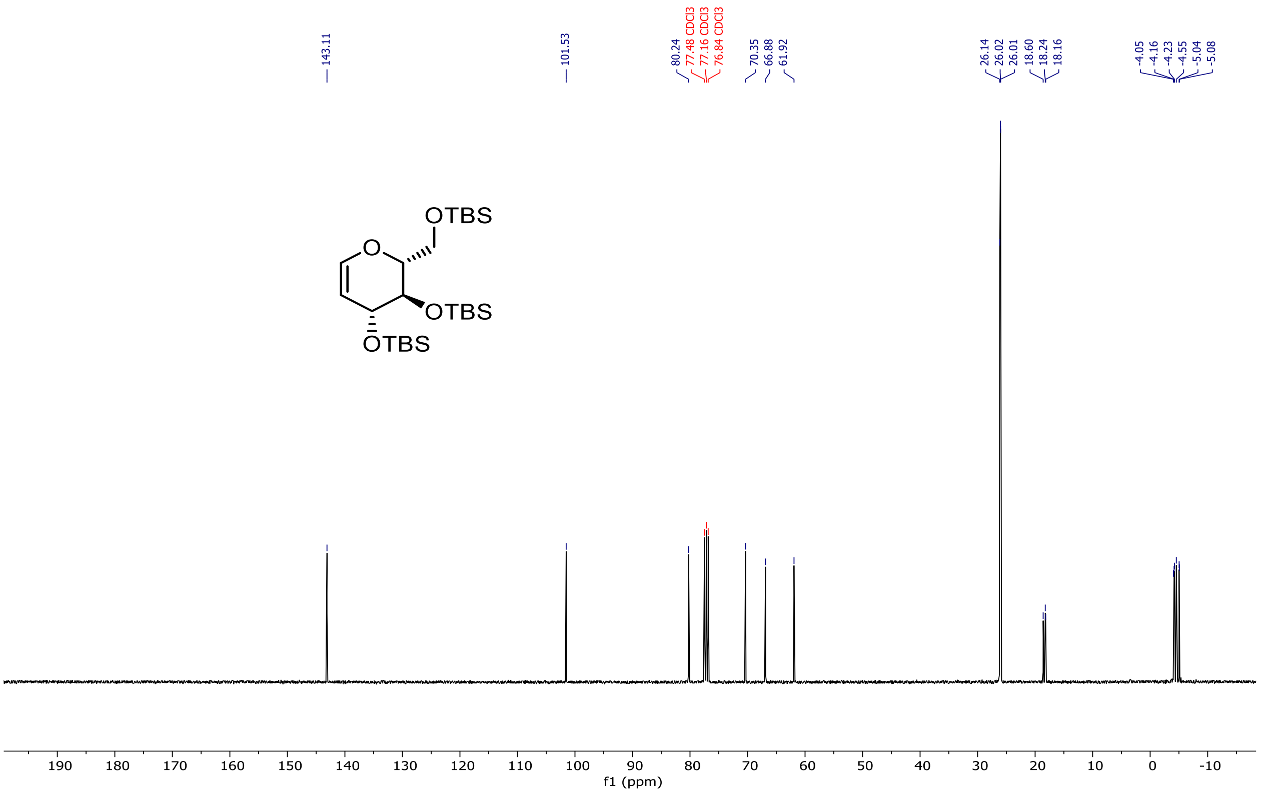

Compound 50: ^{1}H NMR (400 MHz, $CDCl_3$)

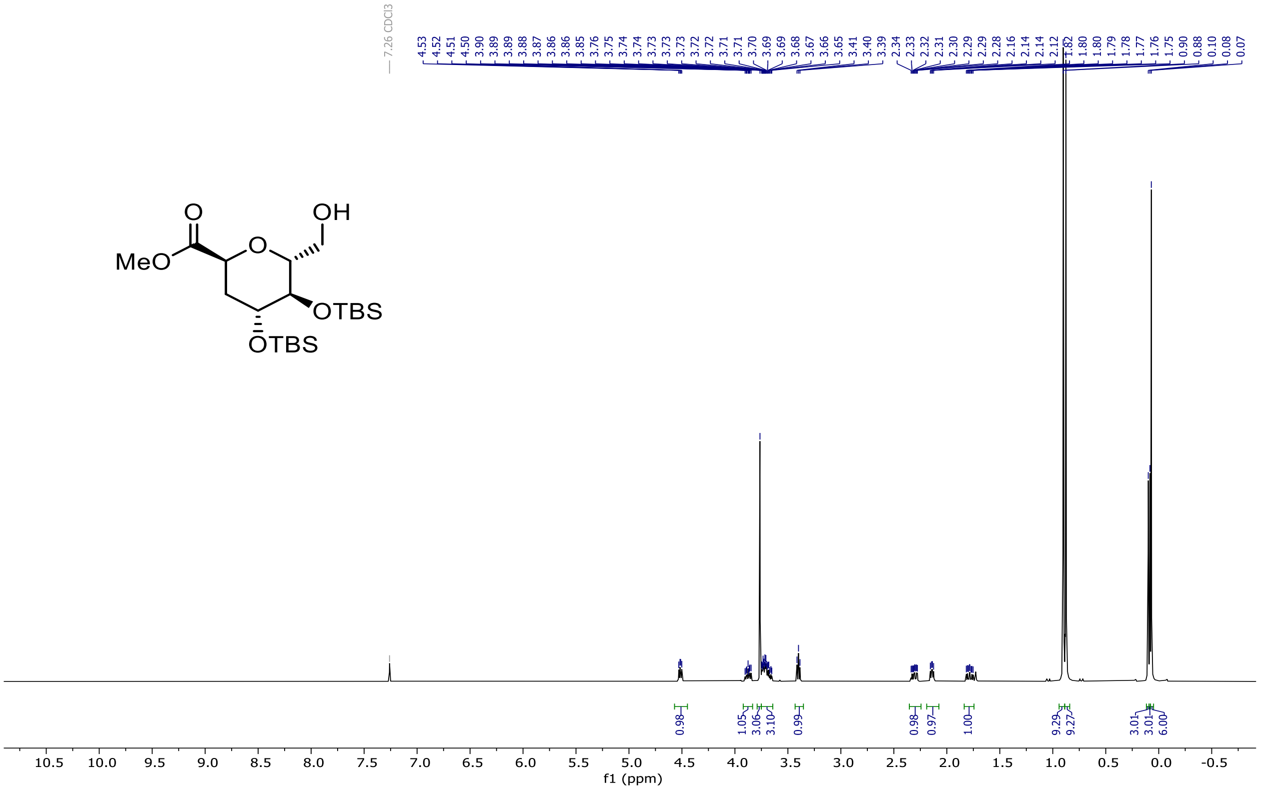

^{13}C NMR (101 MHz, $CDCl_3$)

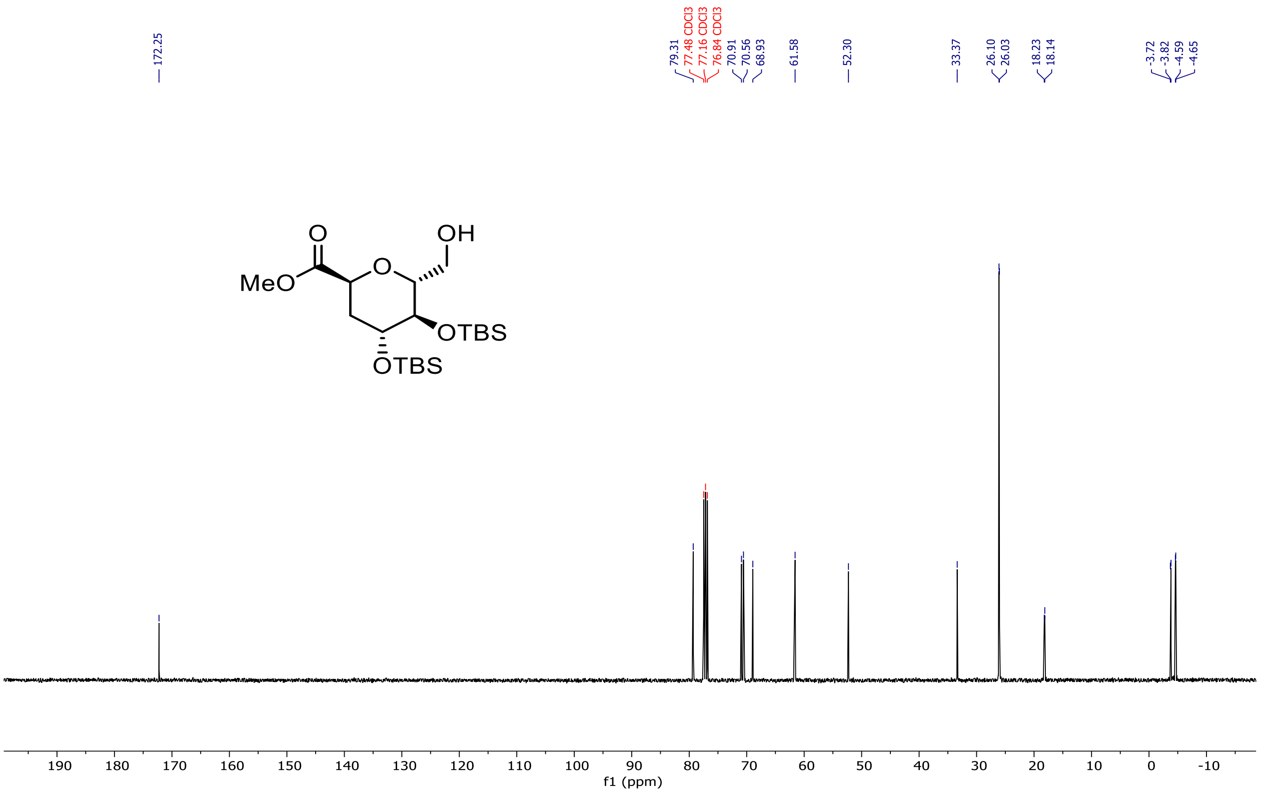

Compound 52: ^{1}H NMR (400 MHz, CDCl$_3$)

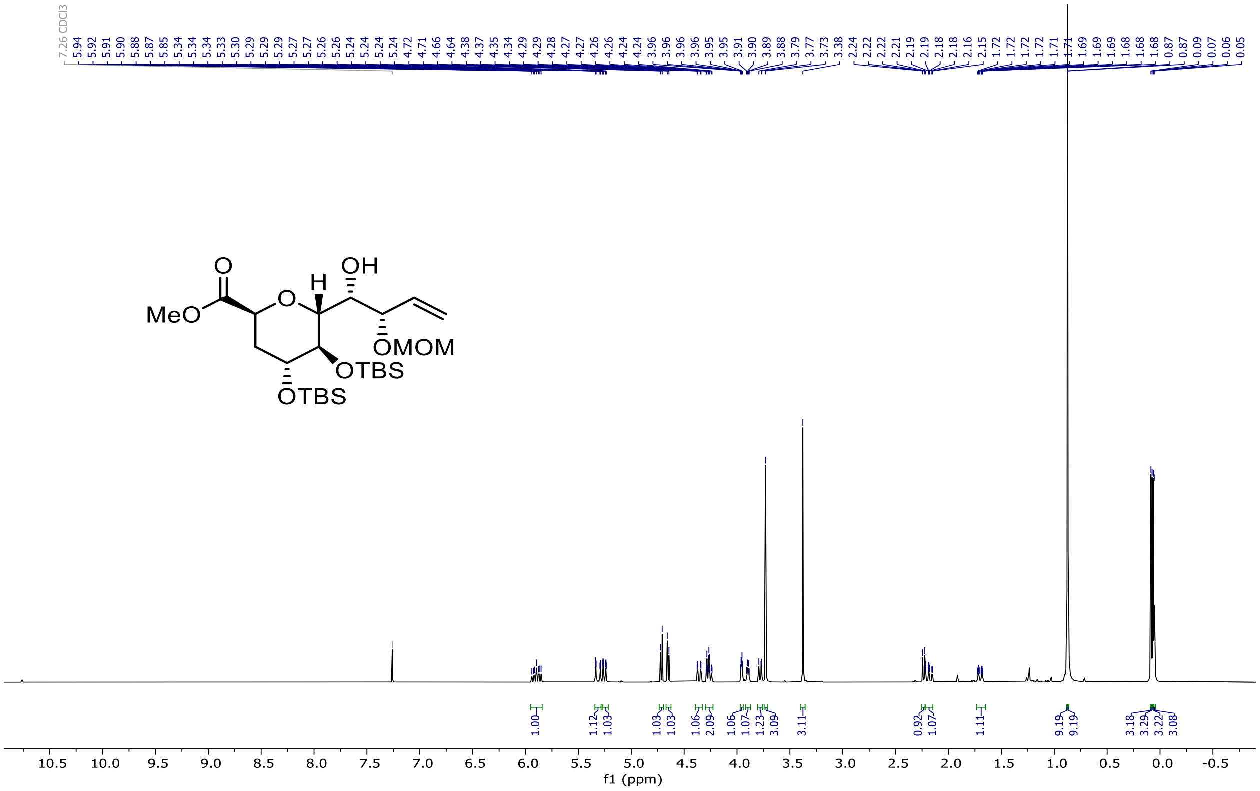

^{13}C NMR (101 MHz, CDCl$_3$)

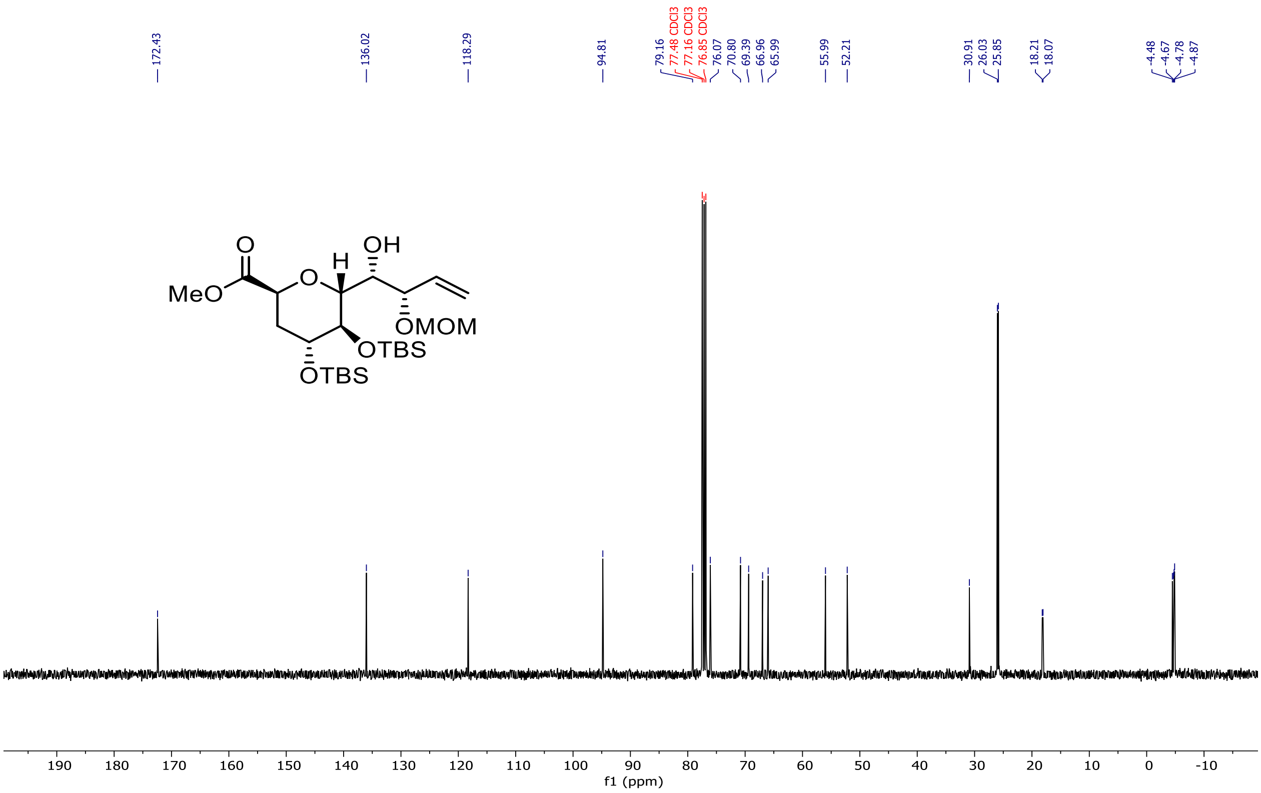

Compound S20: ^{1}H NMR (400 MHz, [D$_4$]-MeOH)

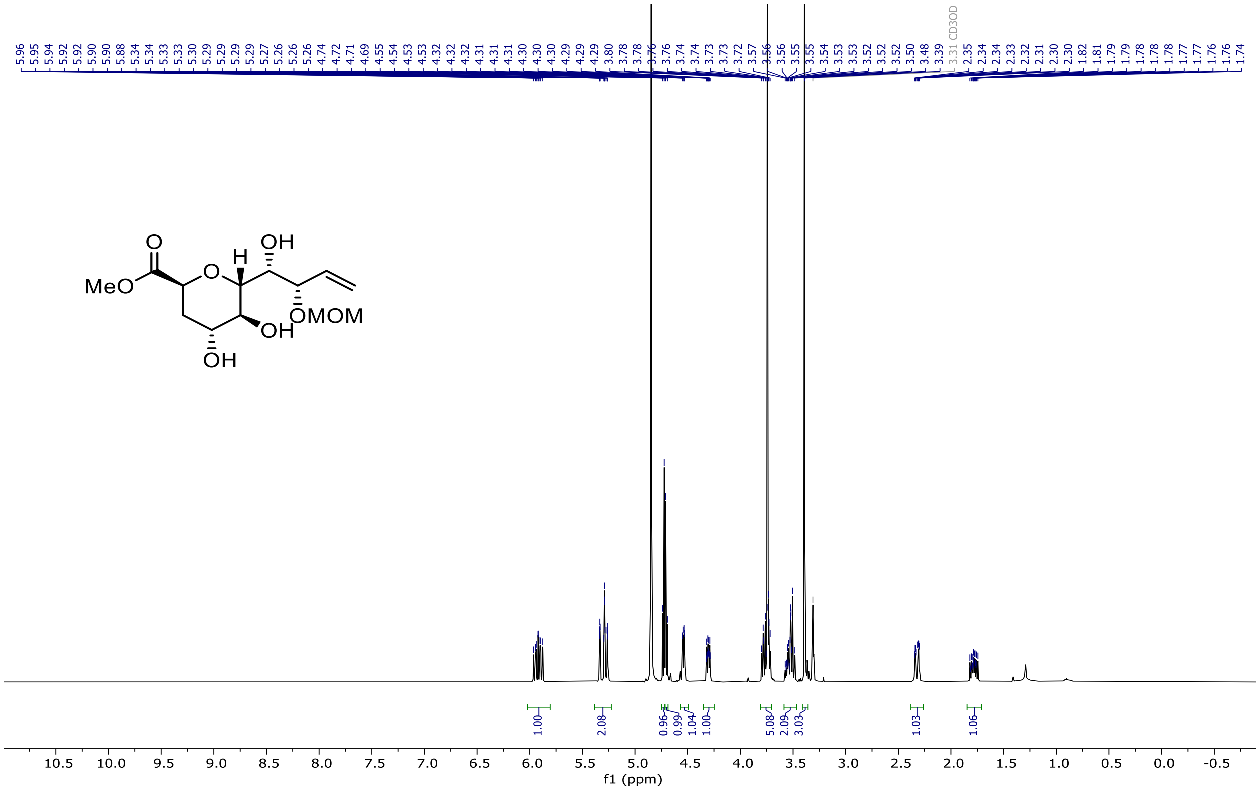

^{13}C NMR (101 MHz, [D$_4$]-MeOH)

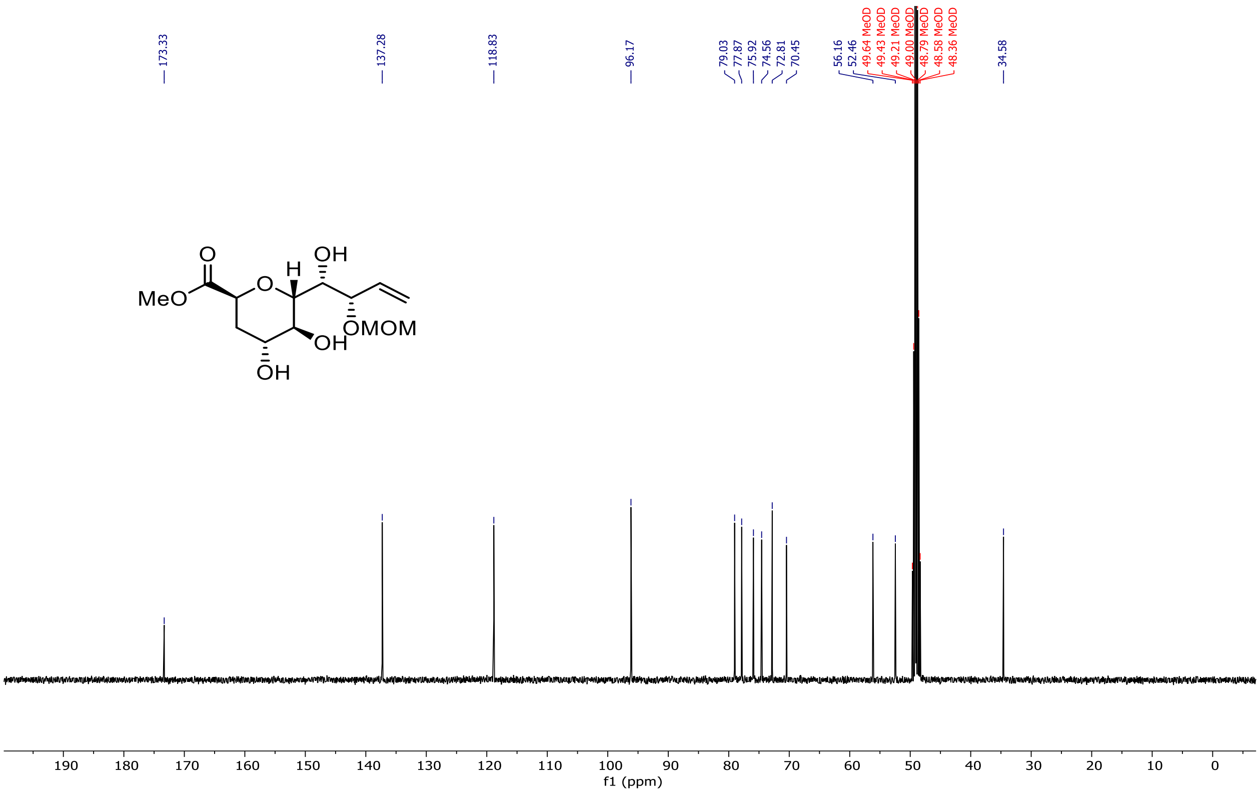

Compound 53: ^{1}H NMR (400 MHz, $CDCl_3$)

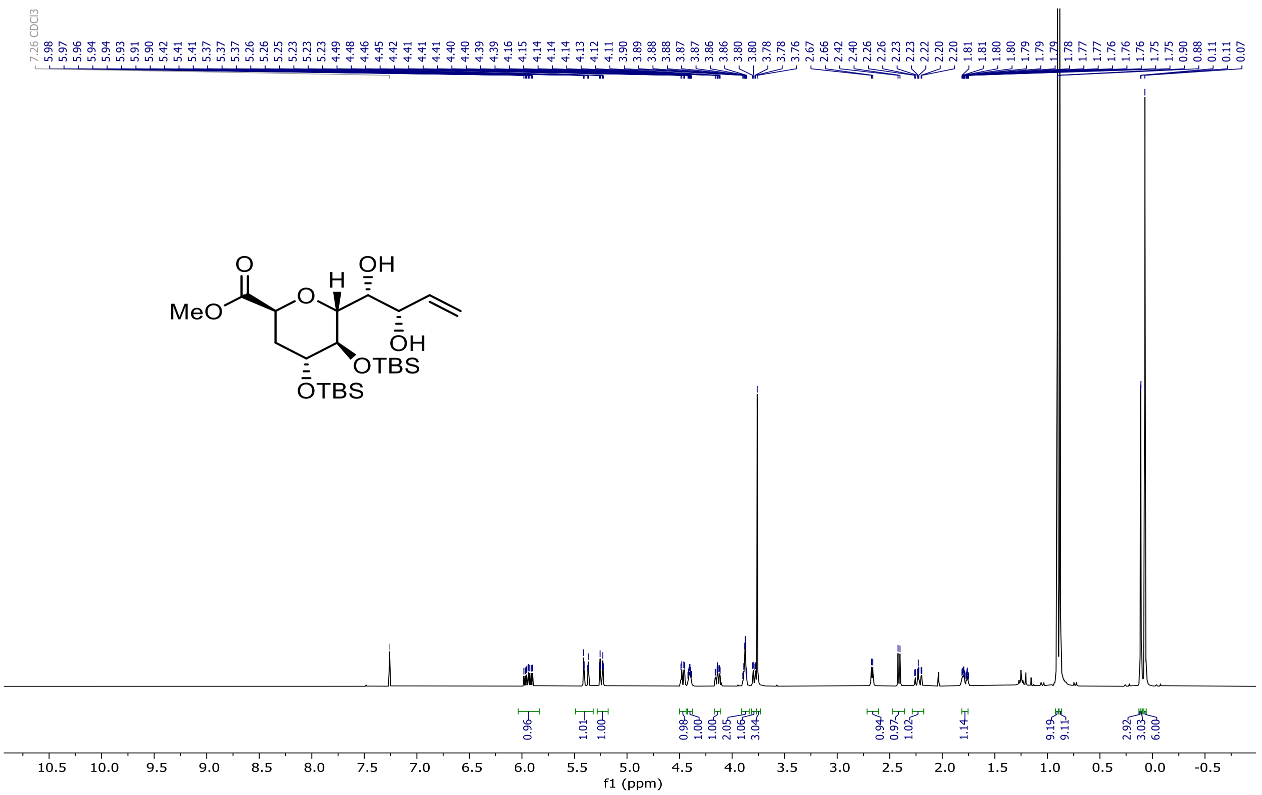

^{13}C NMR (101 MHz, $CDCl_3$)

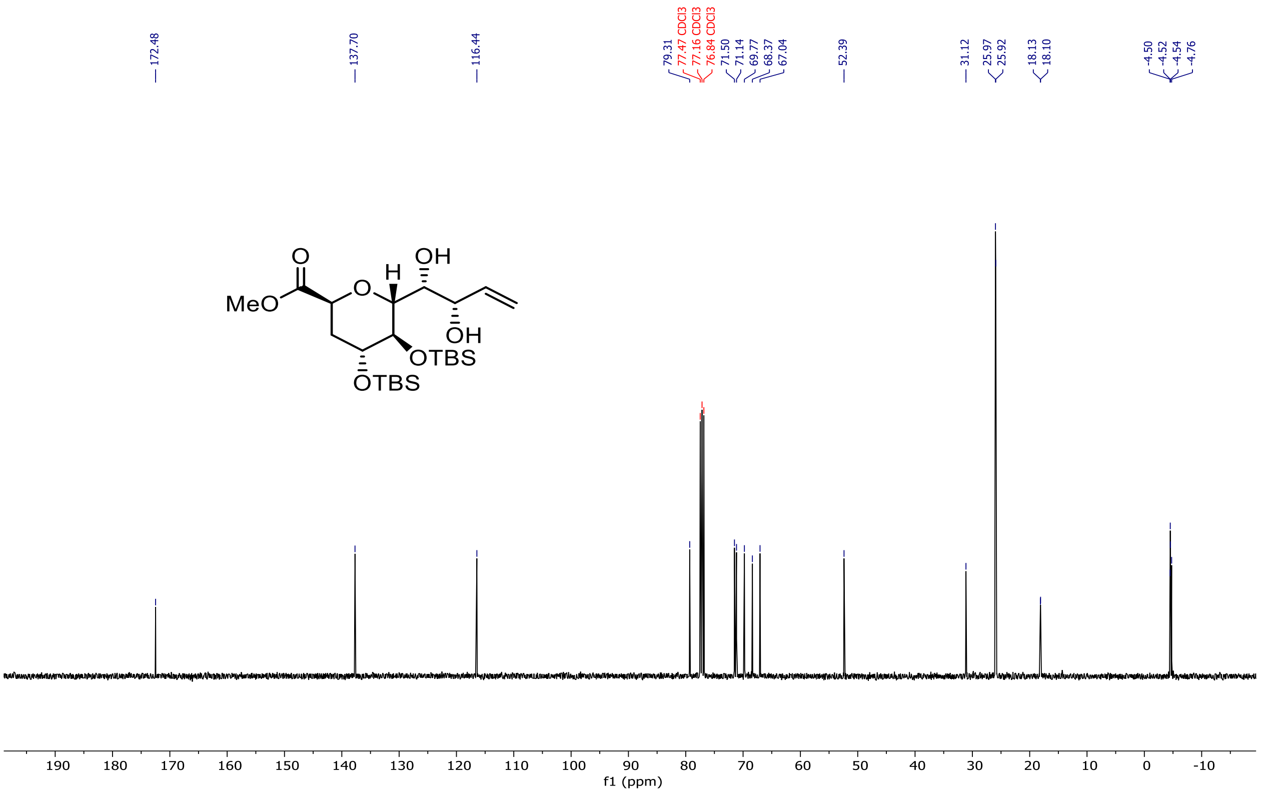

Compound 54: ^{1}H NMR (400 MHz, $CDCl_3$)

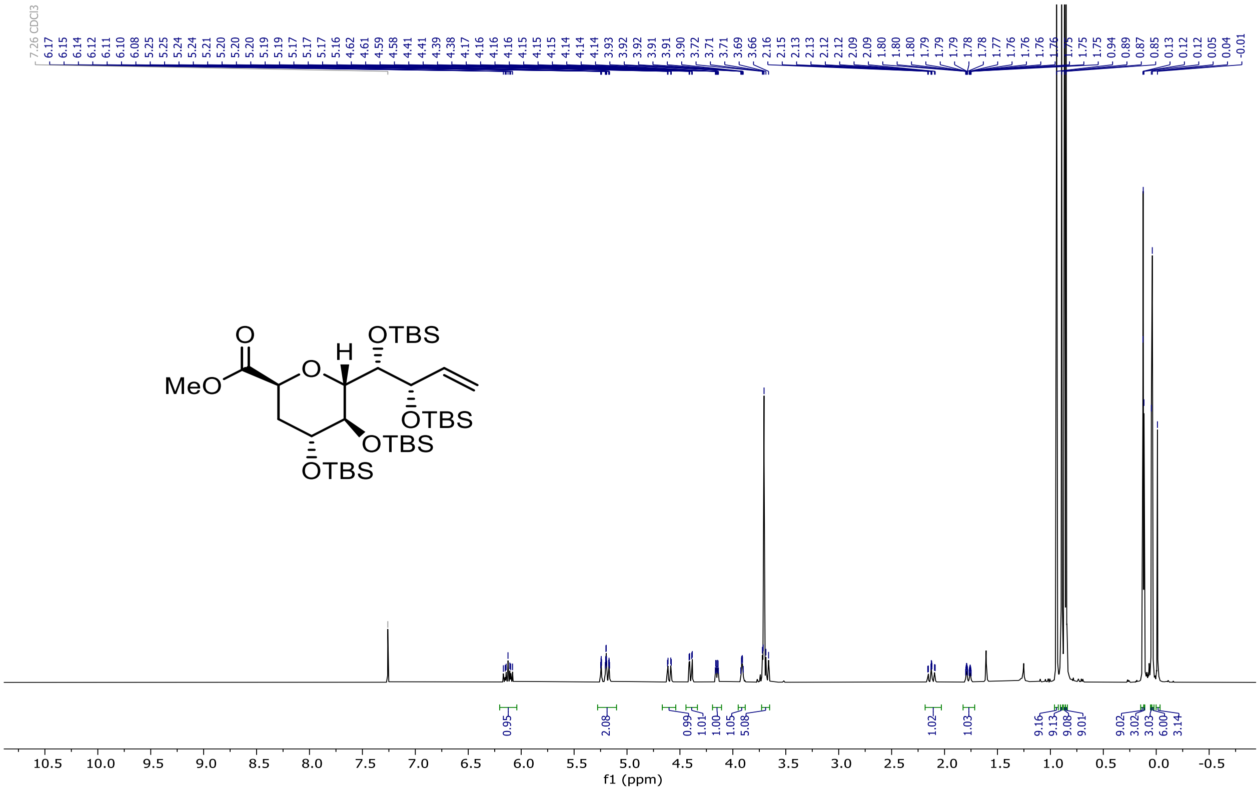

^{13}C NMR (101 MHz, $CDCl_3$)

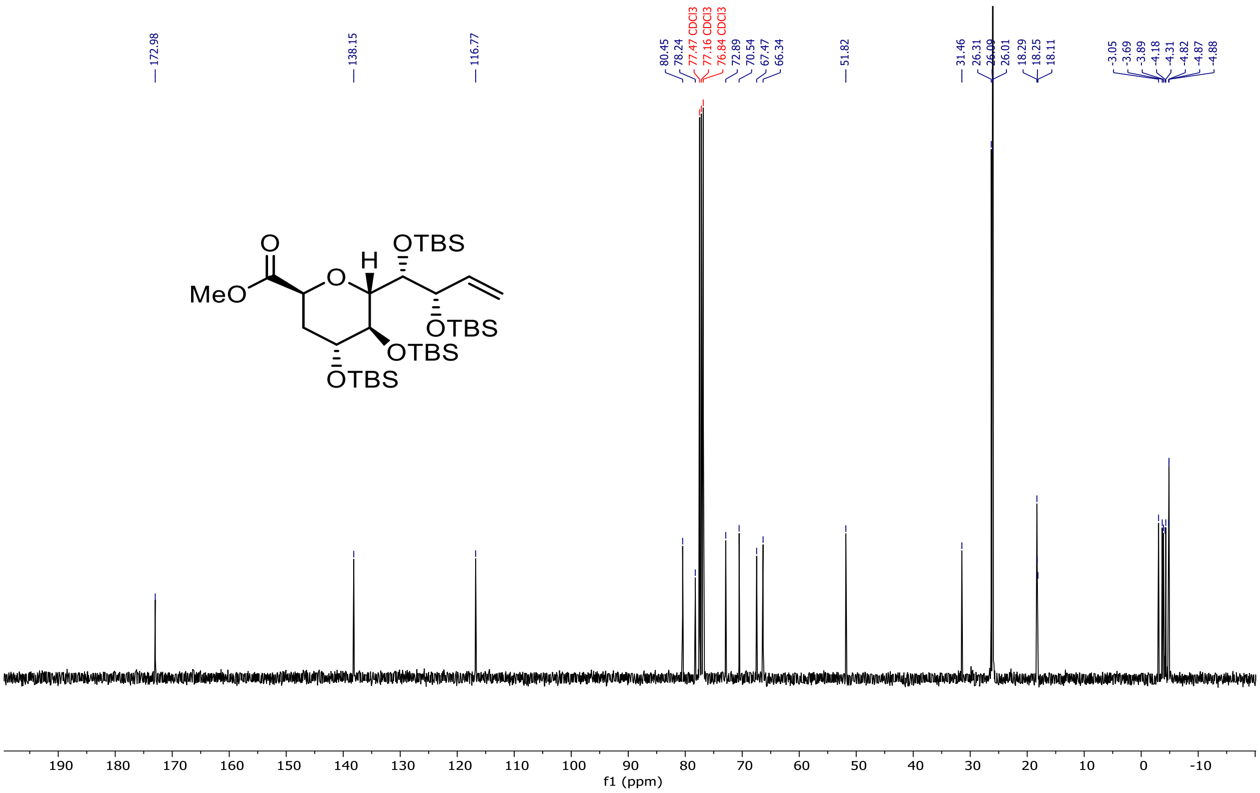

(*R*)-Mosher ester 55: ^{1}H NMR (600 MHz, $CDCl_3$)

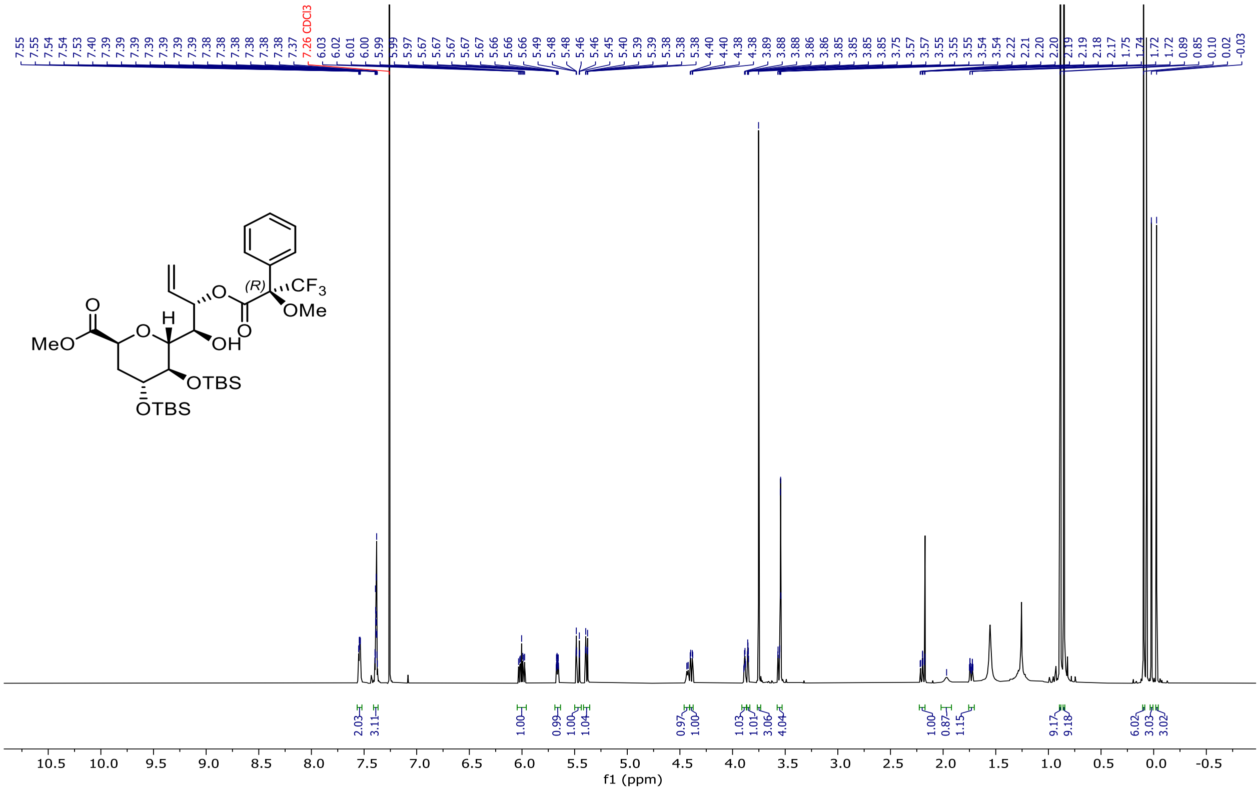

^{13}C NMR (151 MHz, $CDCl_3$)

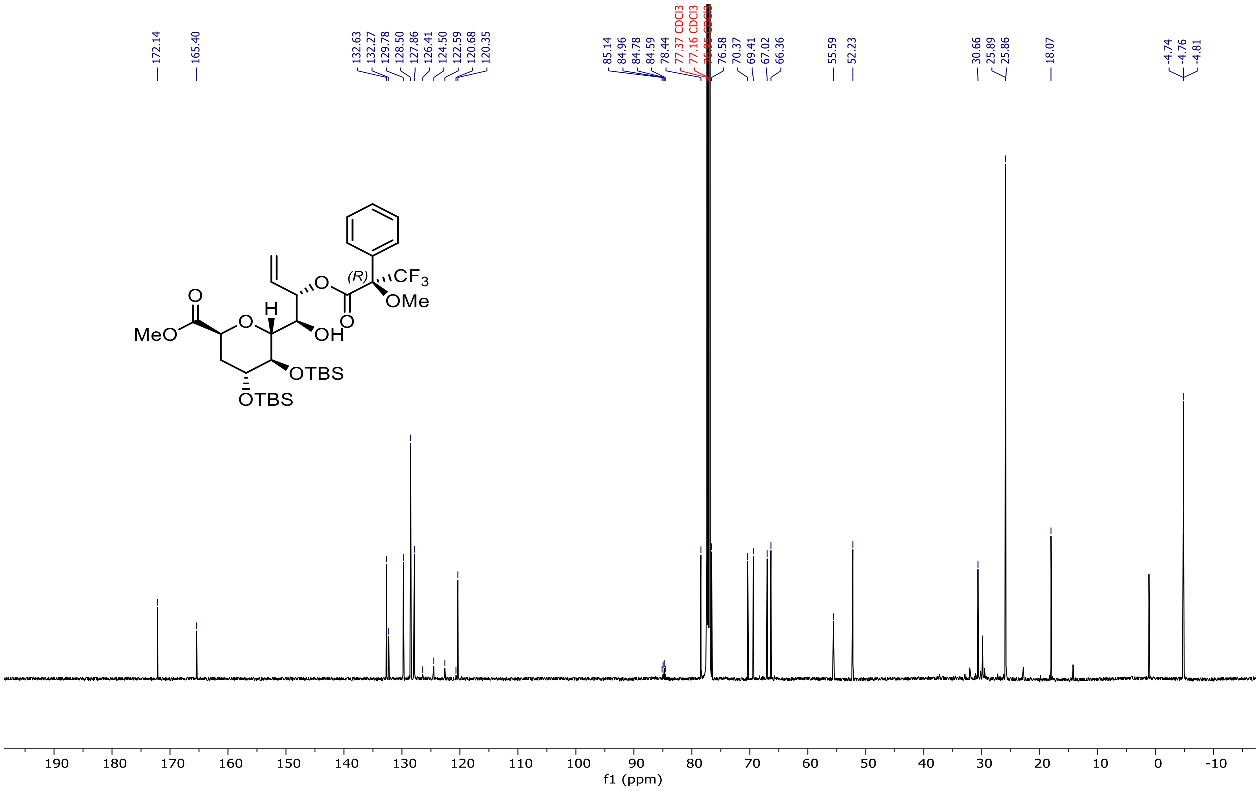

(*R*)-Mosher ester 55: ^1H-^1H COSY ($CDCl_3$)

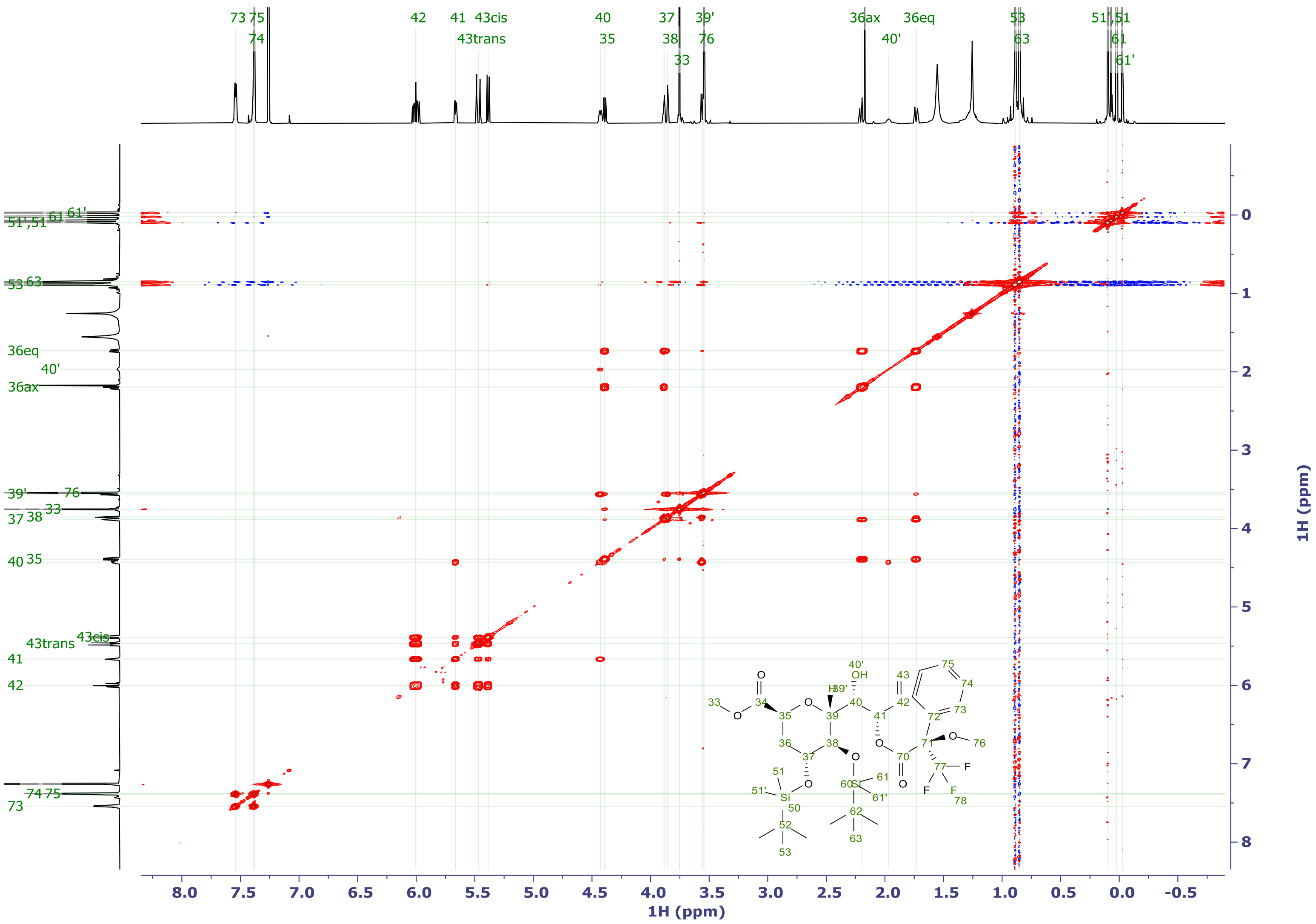

(*R*)-Mosher ester 55: HSQC NMR ($CDCl_3$)

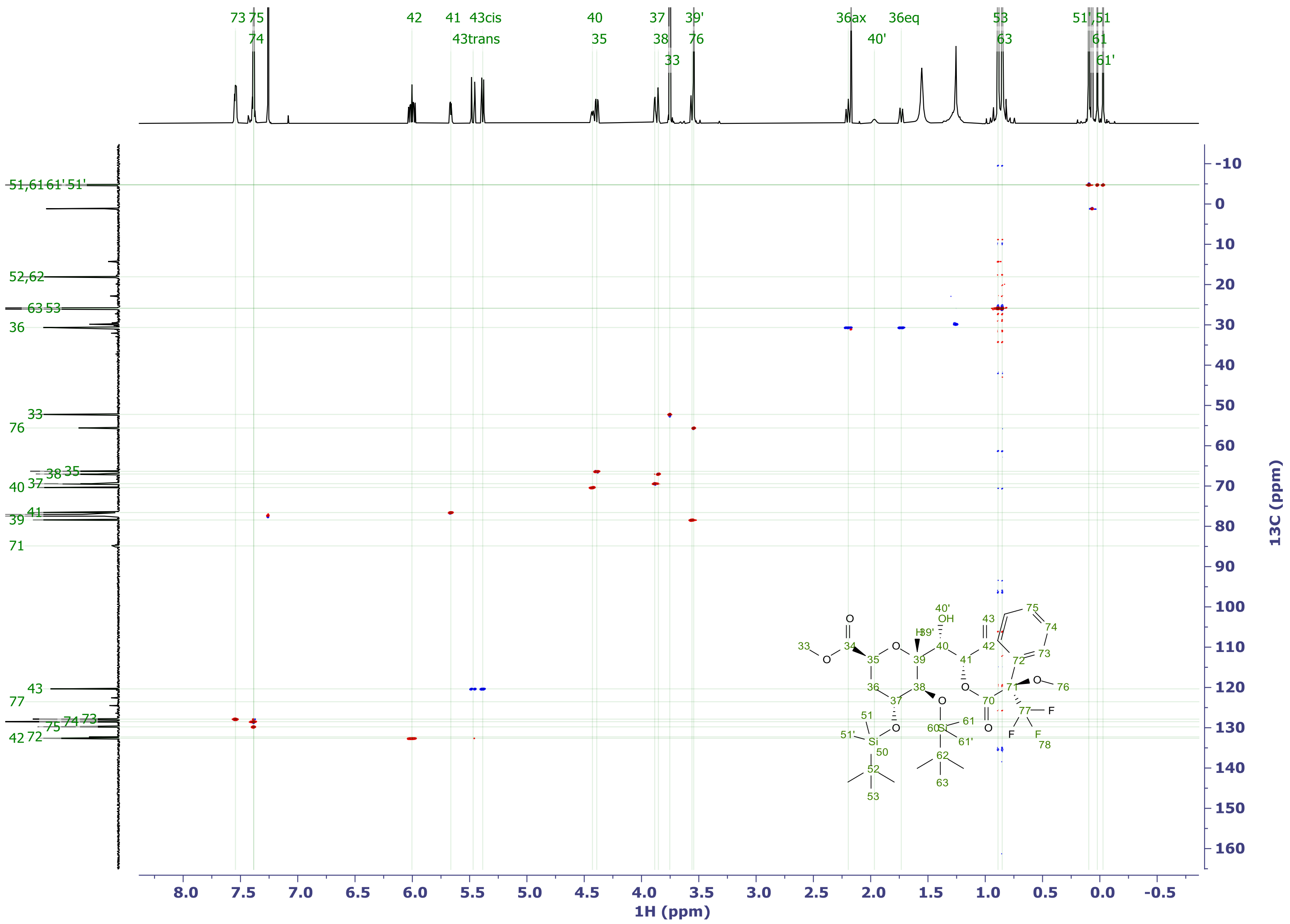

(*R*)-Mosher ester 55: HMBC NMR ($CDCl_3$)

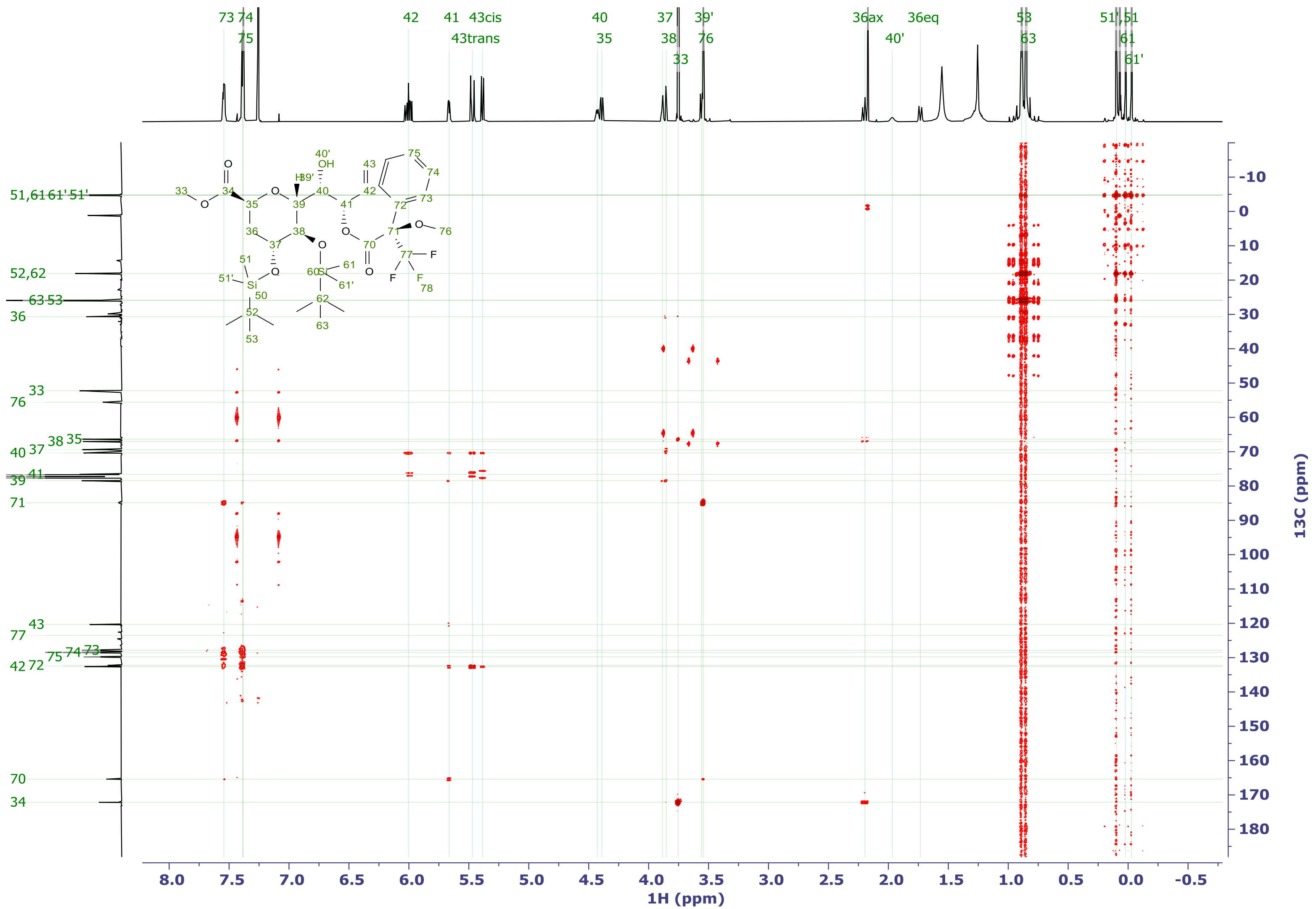

(*R*)-Mosher ester 55: NOESY ($CDCl_3$)

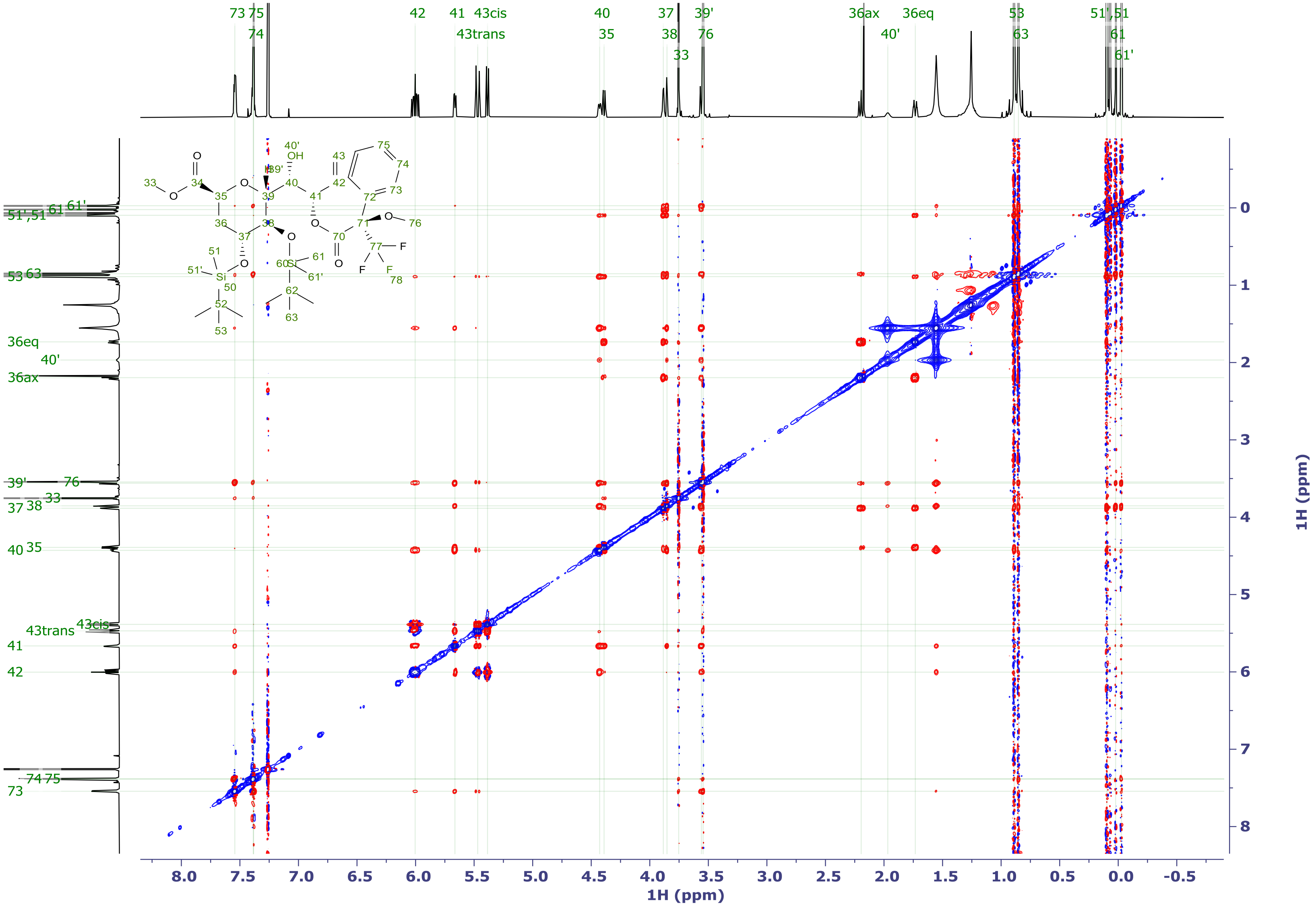

(*S*)-Mosher ester 55: ^{1}H NMR (600 MHz, $CDCl_3$)

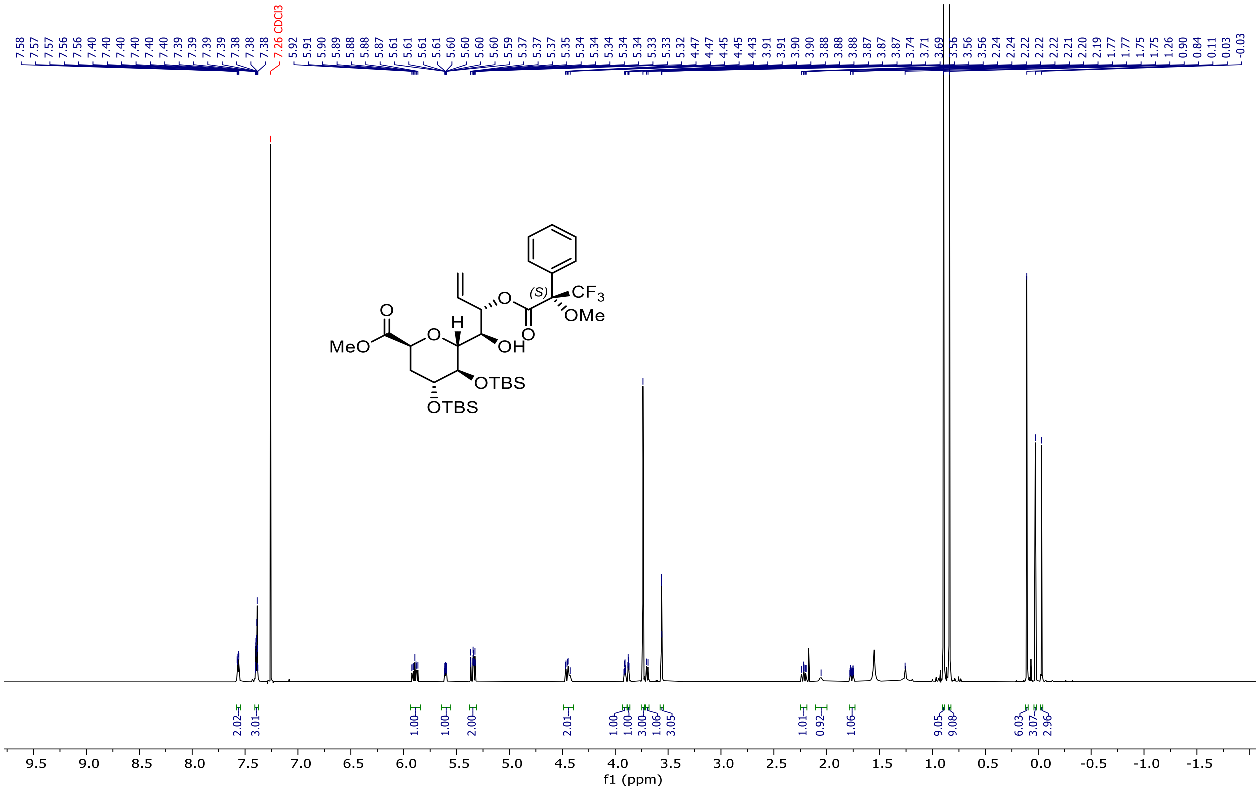

^{13}C NMR (151 MHz, $CDCl_3$)

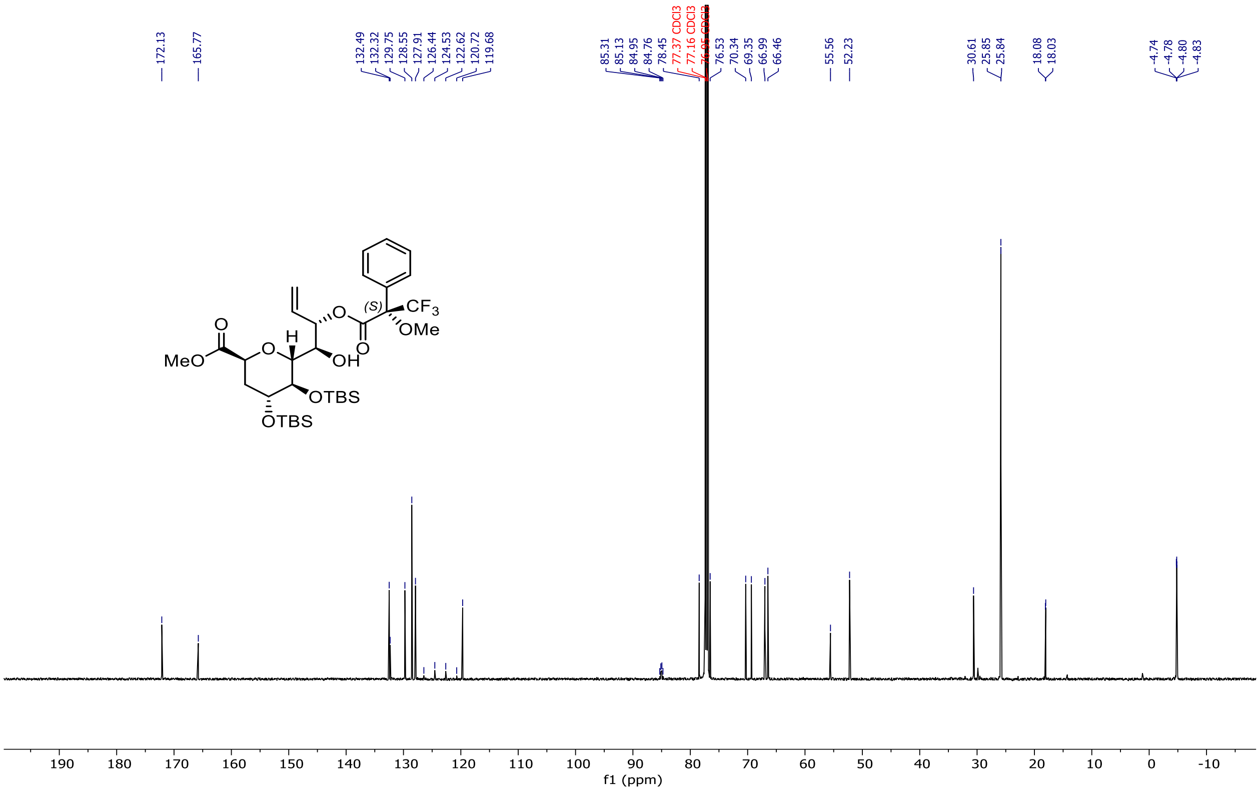

(*S*)-Mosher ester 55: ^{1}H-^{1}H COSY ($CDCl_3$)

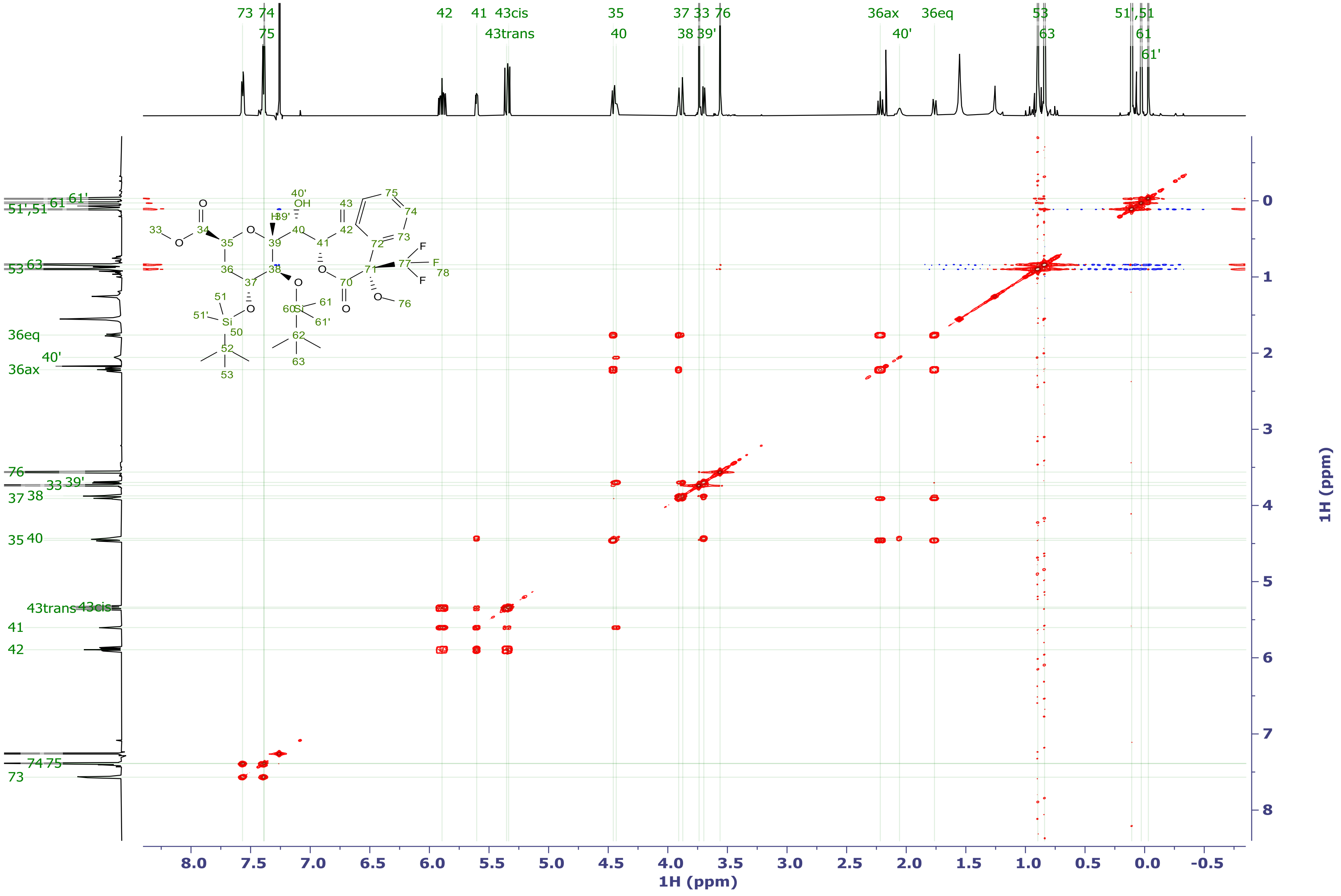

(**_S_)-Mosher ester 55**: HSQC NMR ($CDCl_3$)

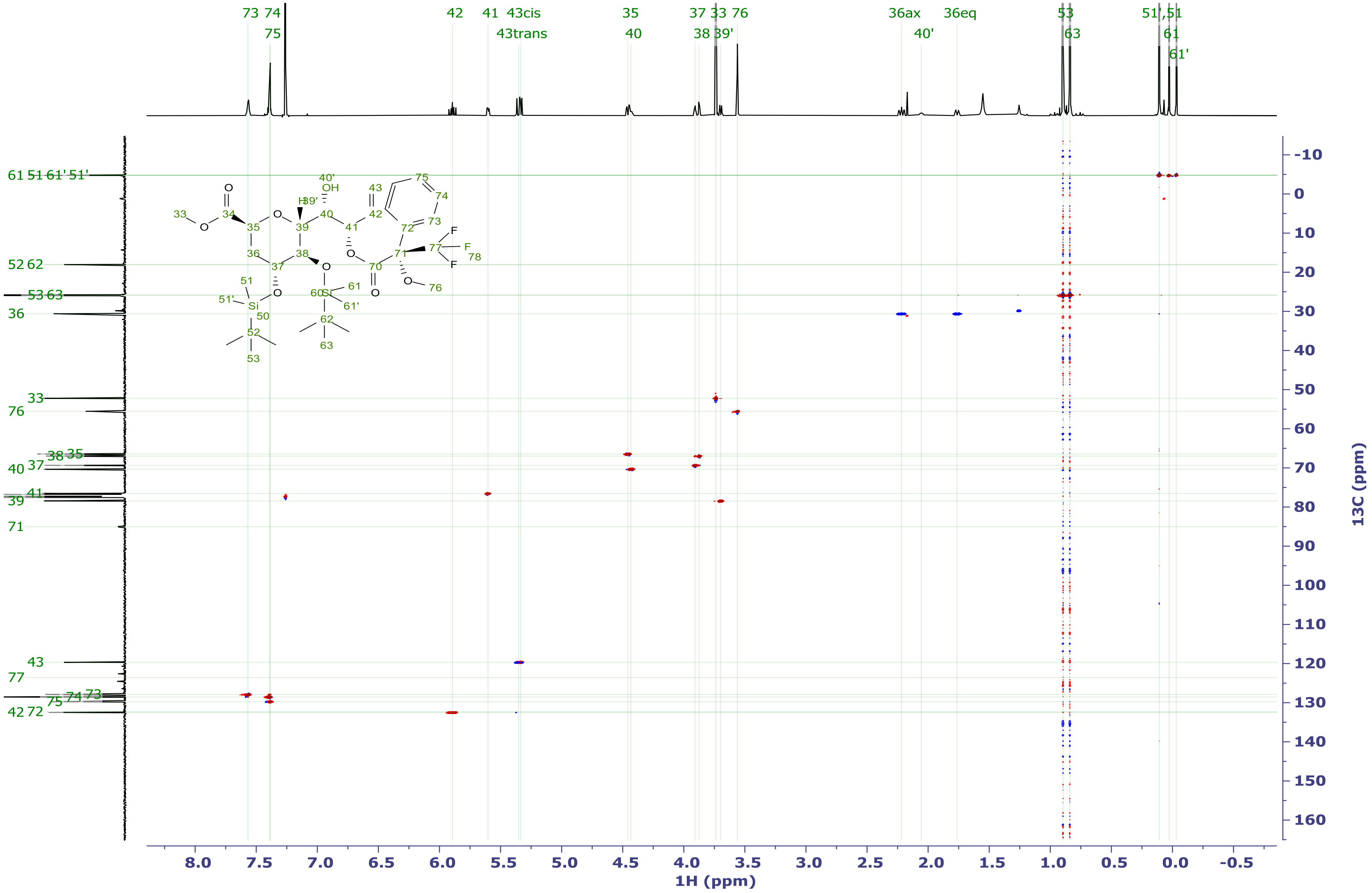

(*S*)-Mosher ester 55: HMBC NMR ($CDCl_3$)

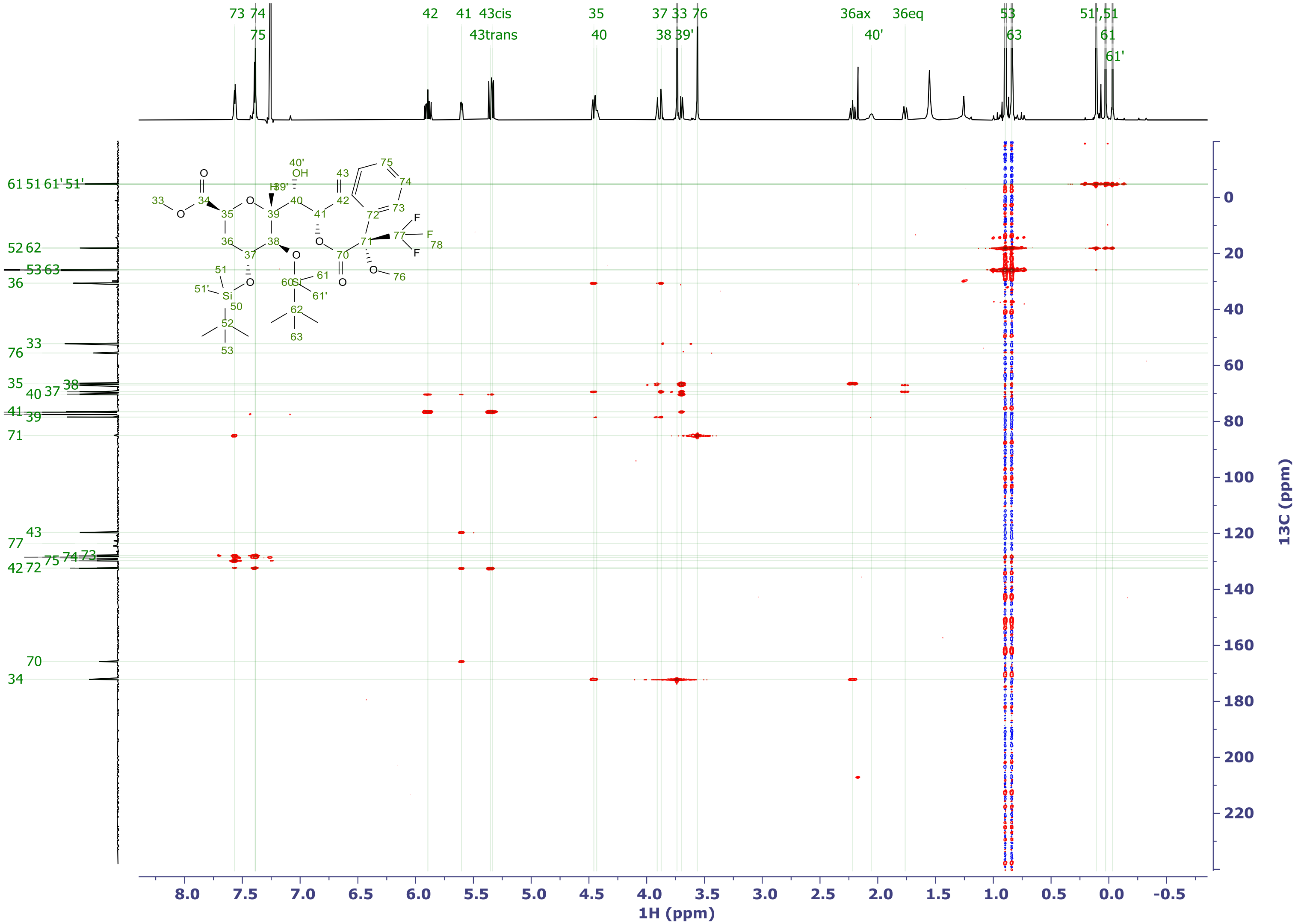

(***S***)**-Mosher ester 55**: NOESY ($CDCl_3$)

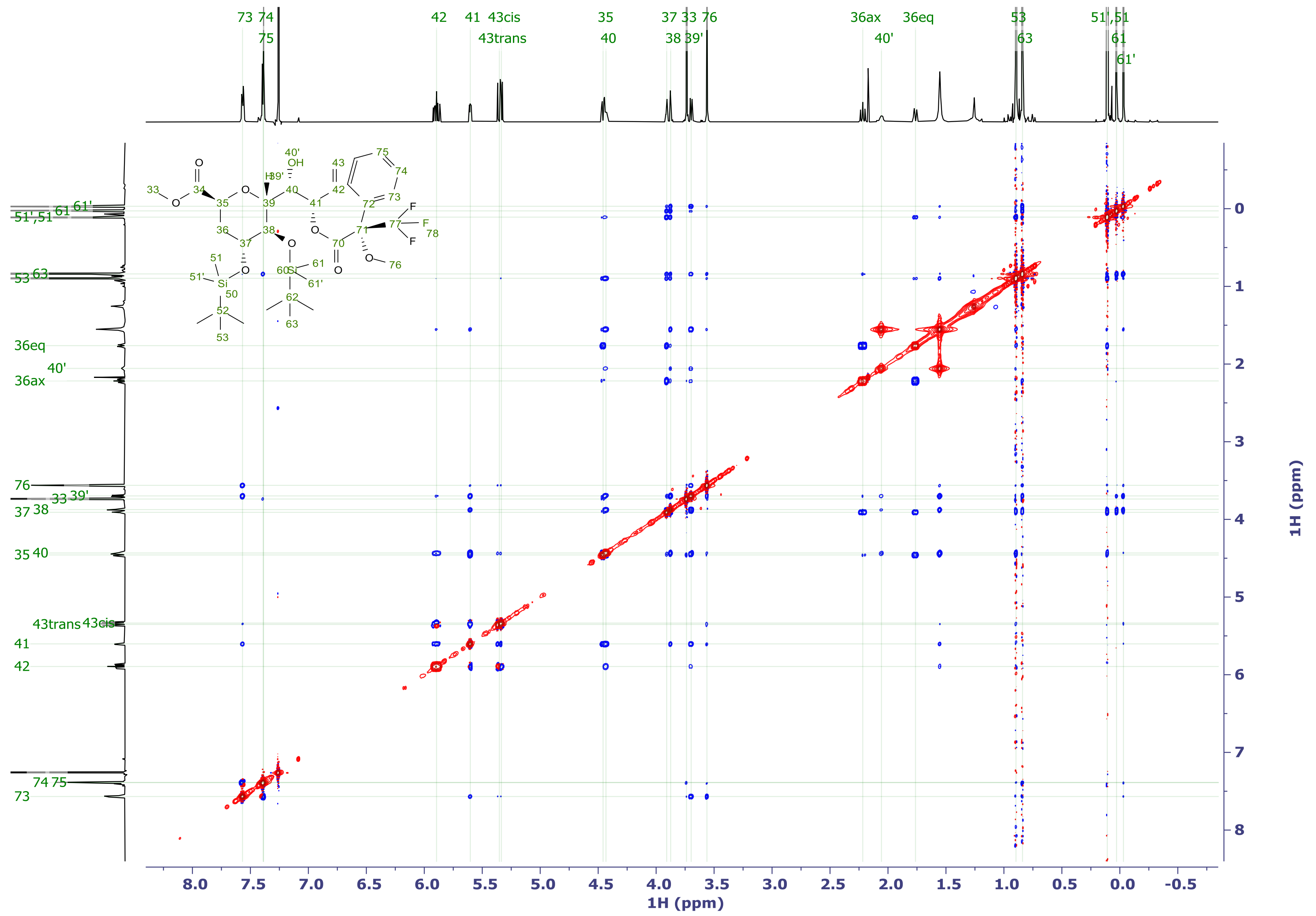

Compound 56: ^{1}H NMR (400 MHz, $CDCl_3$)

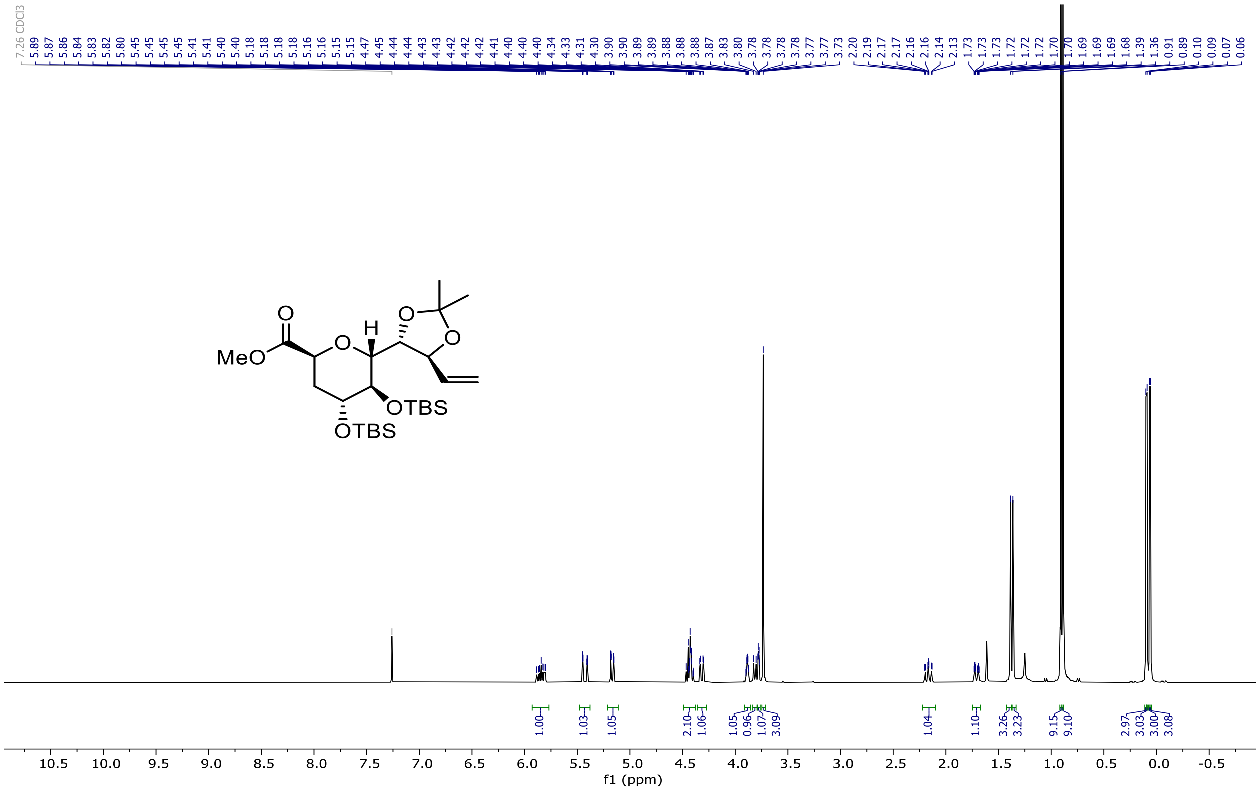

^{13}C NMR (101 MHz, $CDCl_3$)

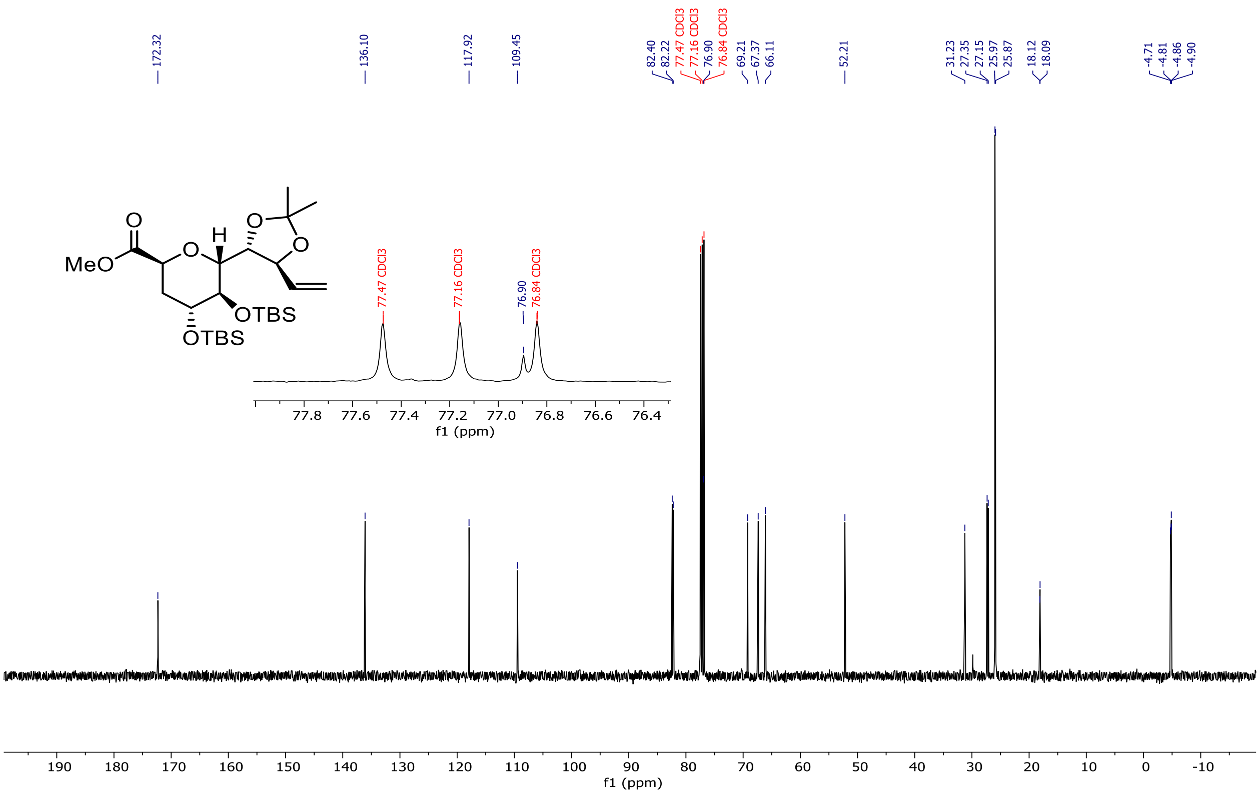

Compound 56: ^{1}H-^{1}H COSY ($CDCl_3$)

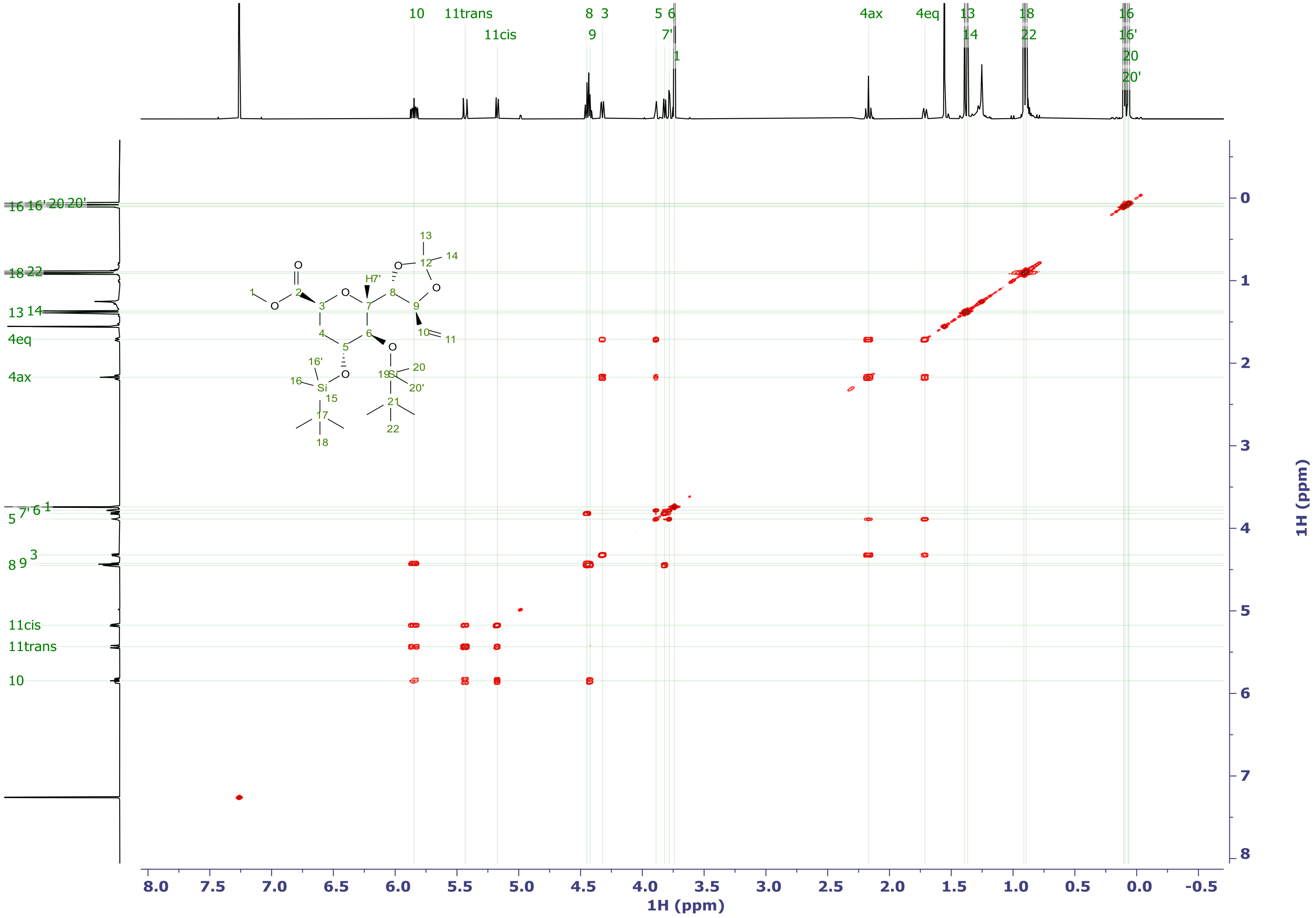

Compound 56: HSQC NMR ($CDCl_3$)

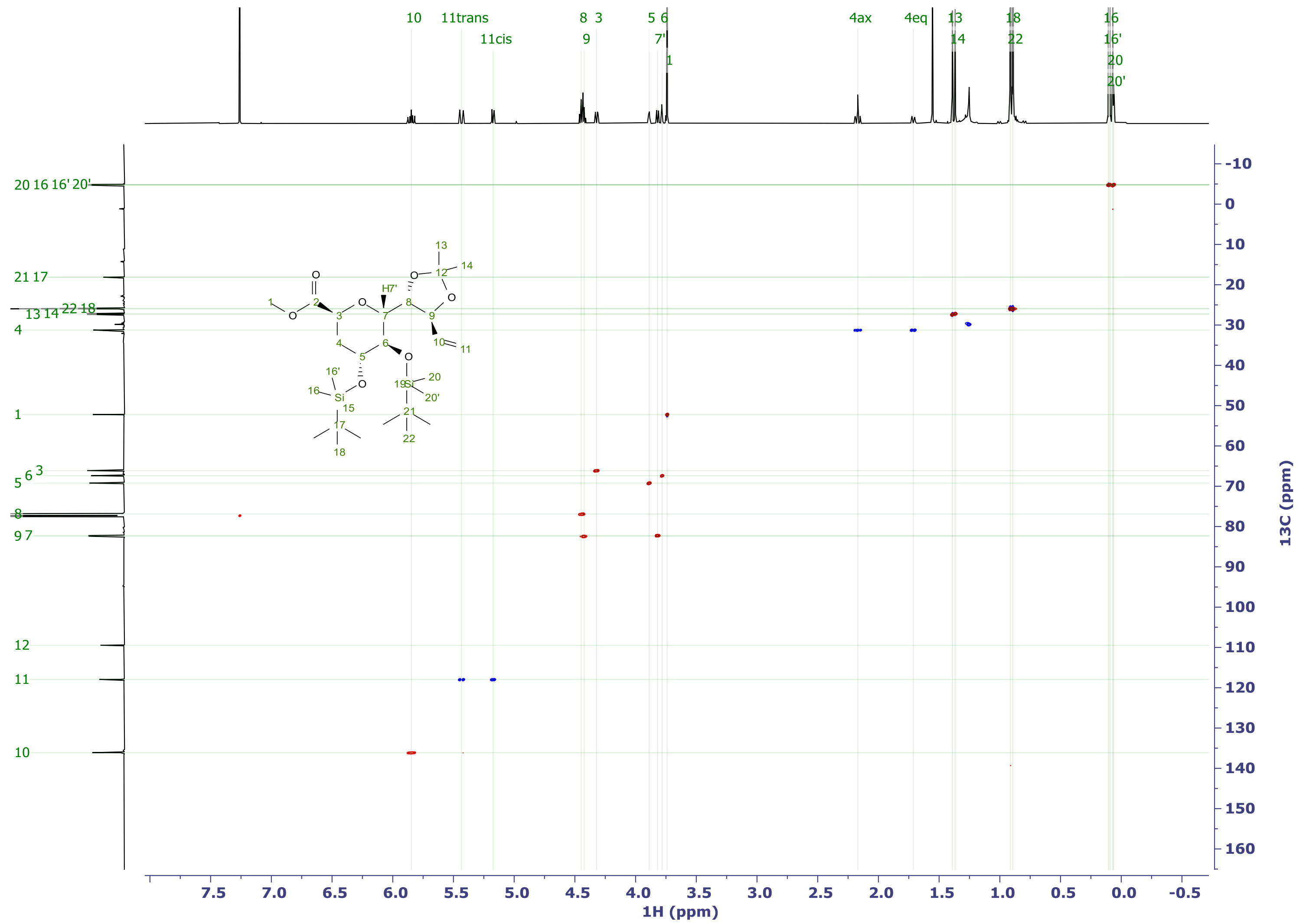

Compound 56: HMBC NMR ($CDCl_3$)

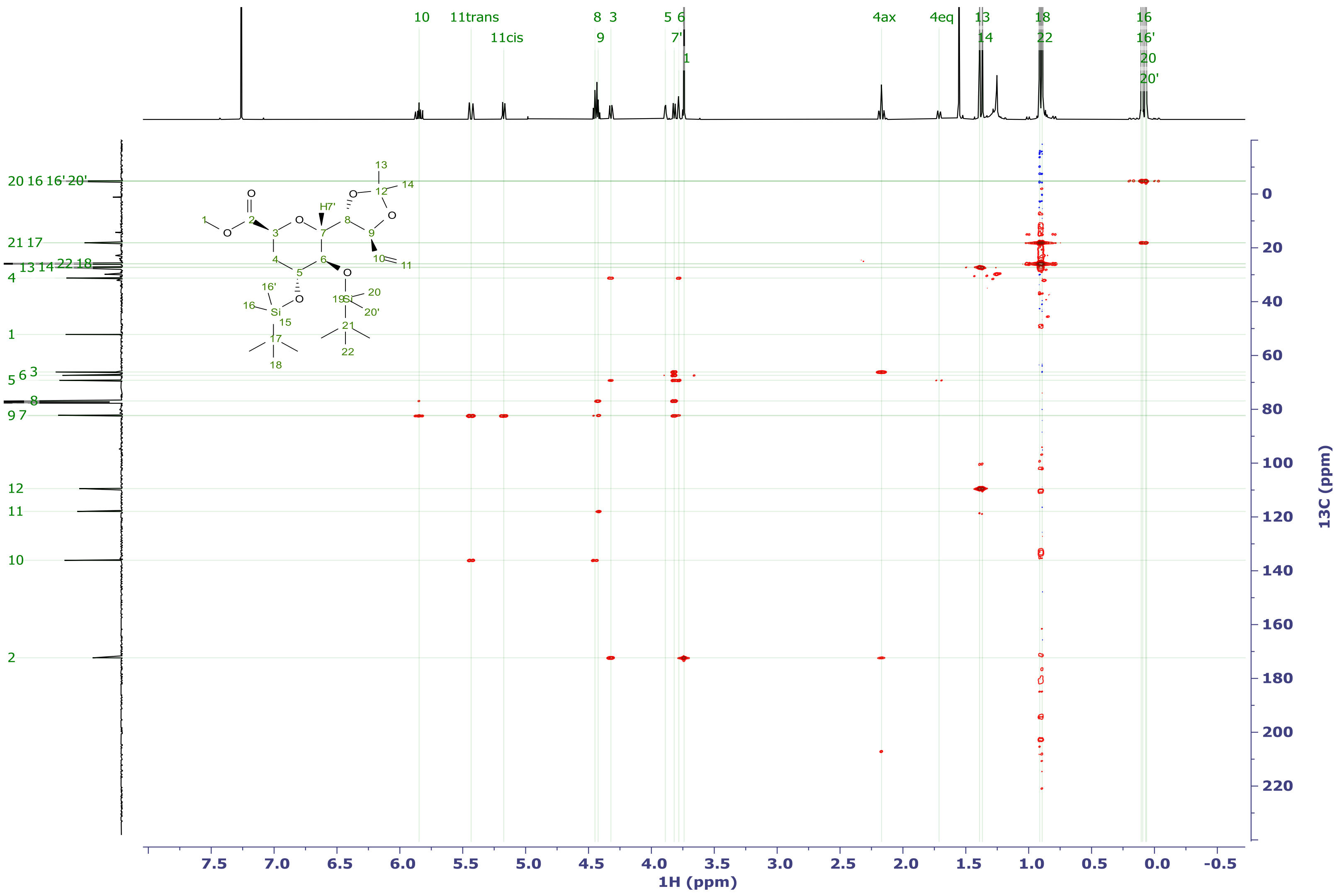

Compound 56: NOESY ($CDCl_3$)

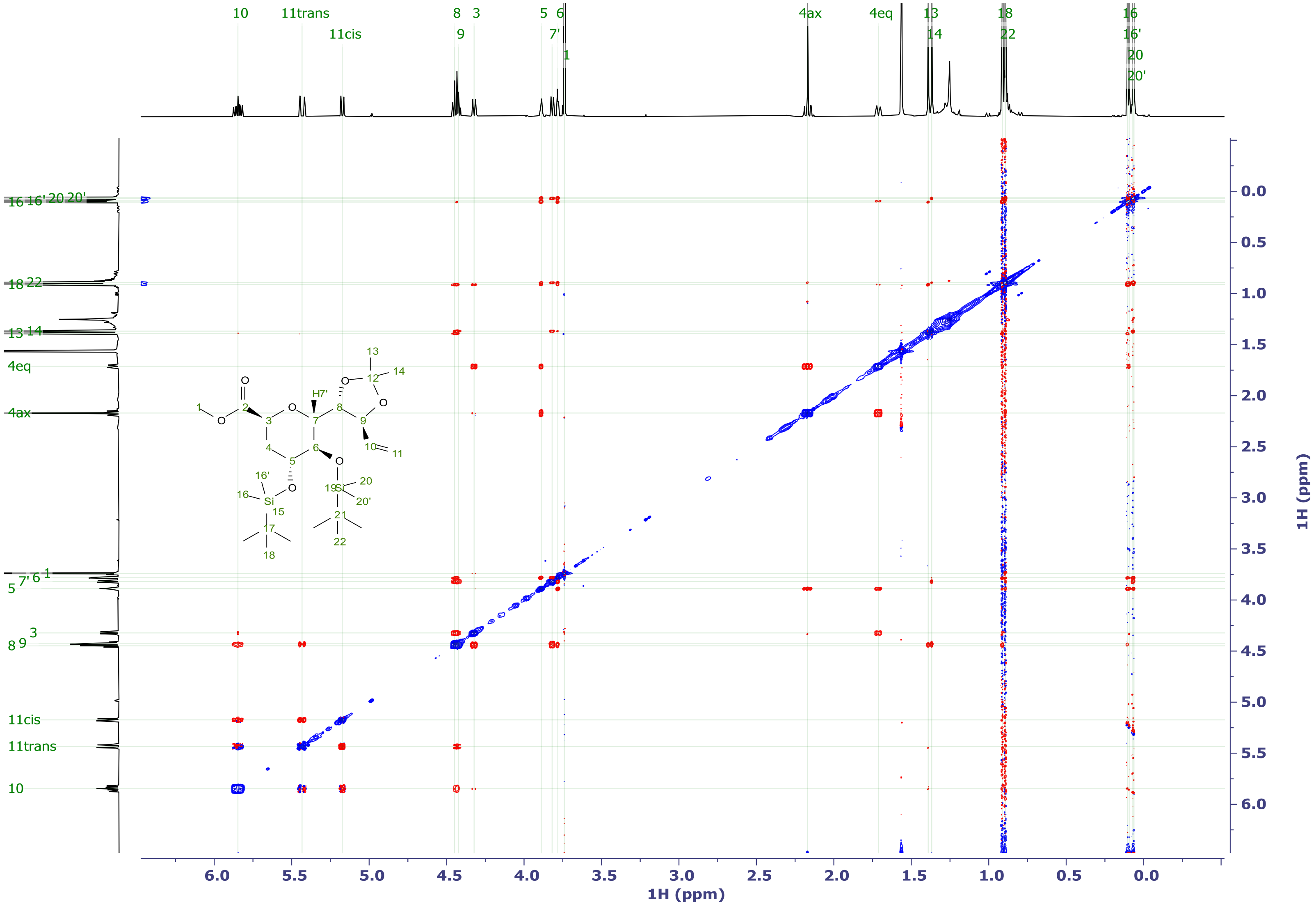

Compound 57: ^{1}H NMR (600 MHz, CD_2Cl_2)

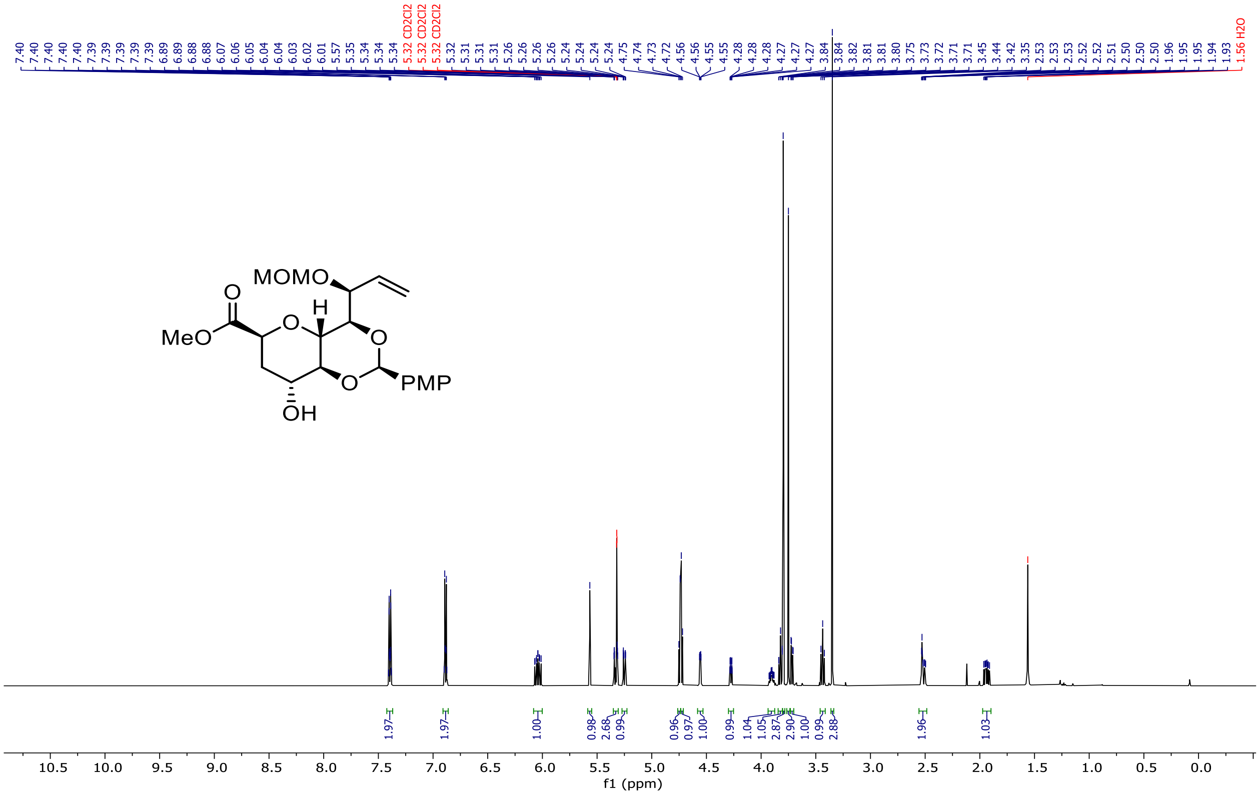

^{13}C NMR (151 MHz, CD_2Cl_2)

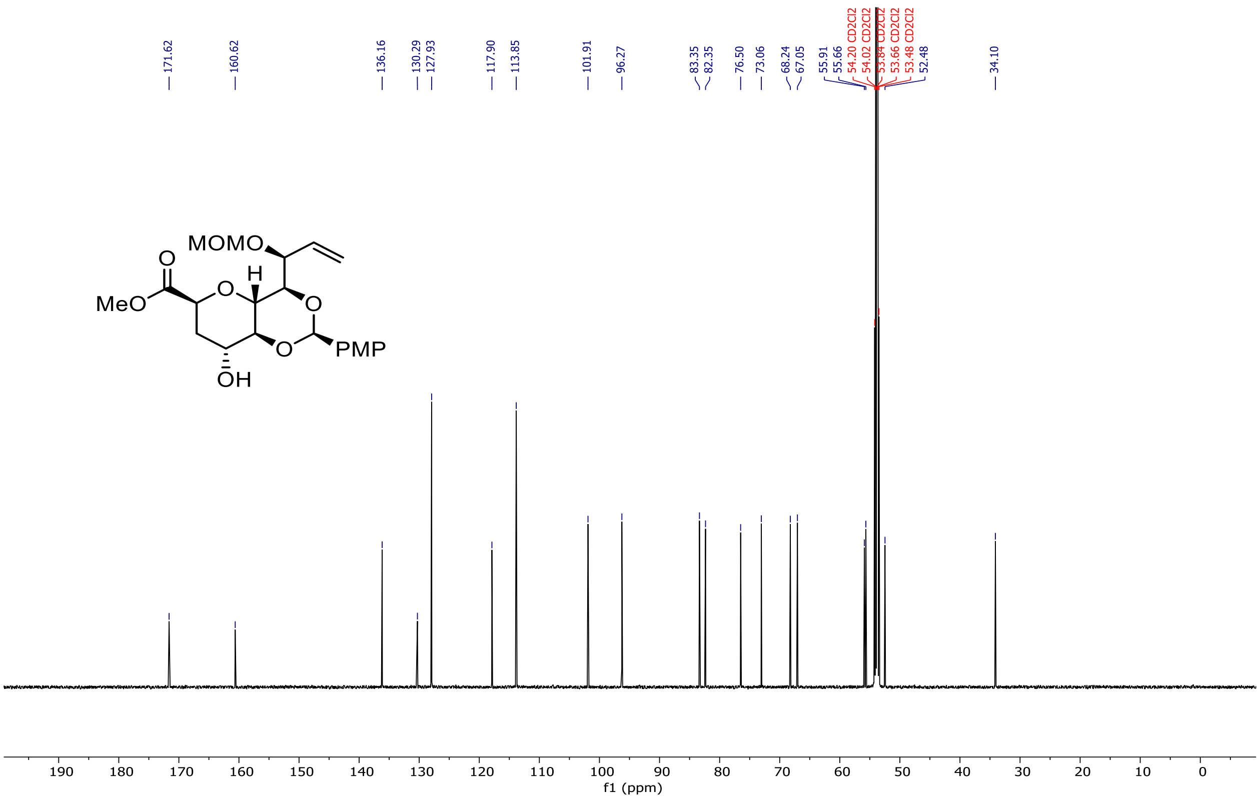

Compound 57: ^{1}H-^{1}H COSY (CD_2Cl_2)

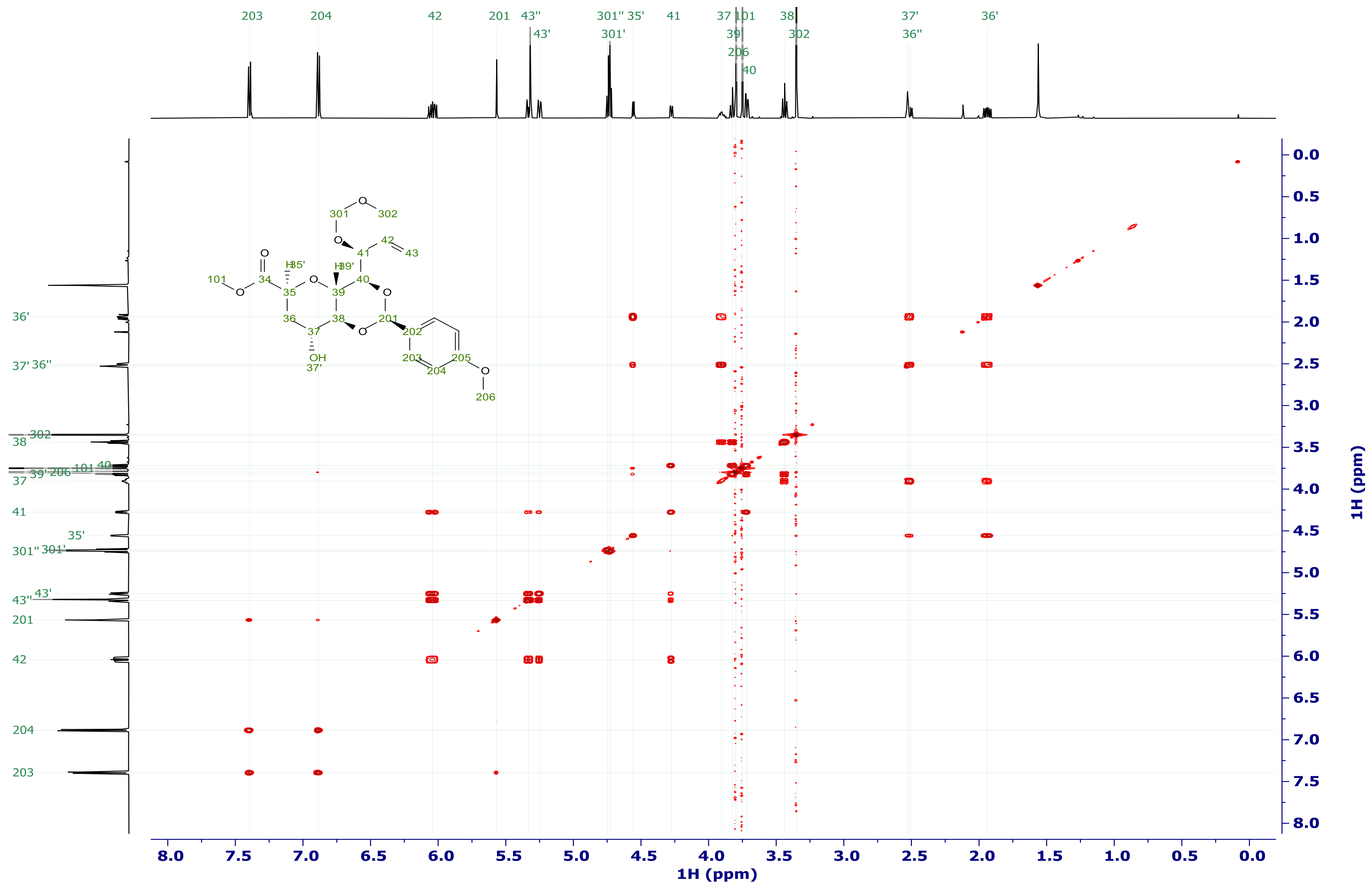

Compound 57: HSQC NMR (CD_2Cl_2)

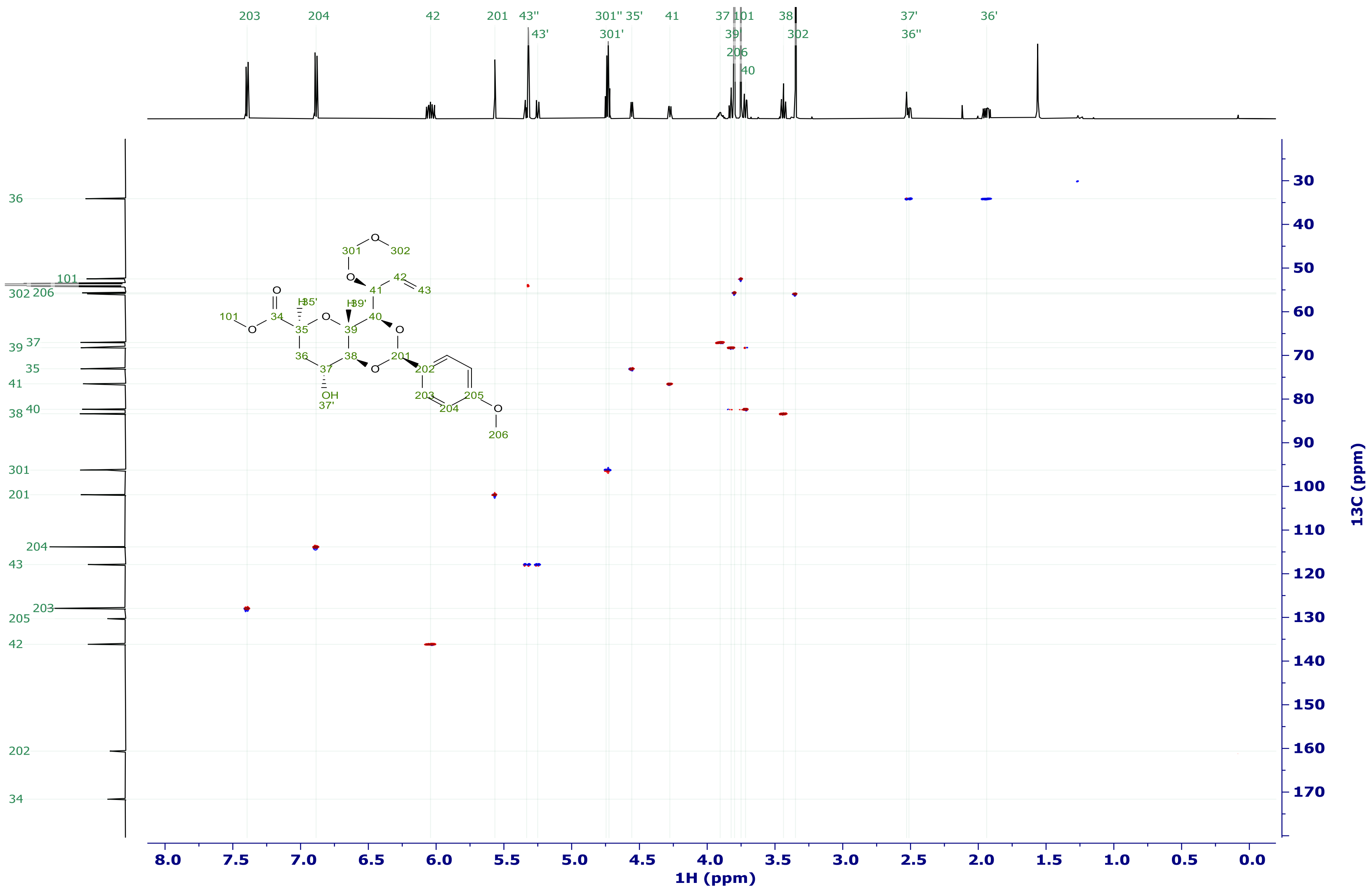

Compound 57: HMBC NMR (CD_2Cl_2)

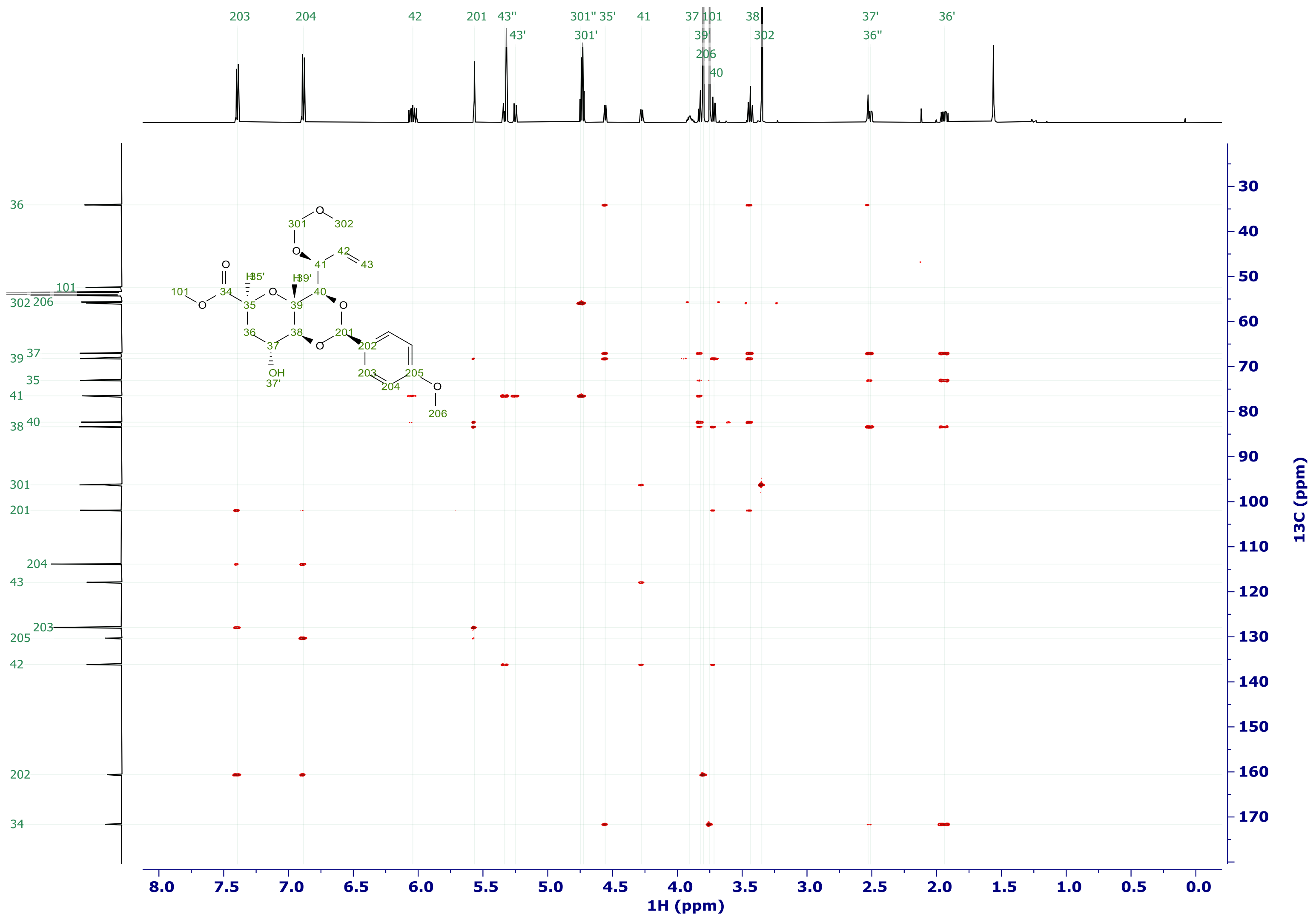

Compound 57: NOESY (CD_2Cl_2)

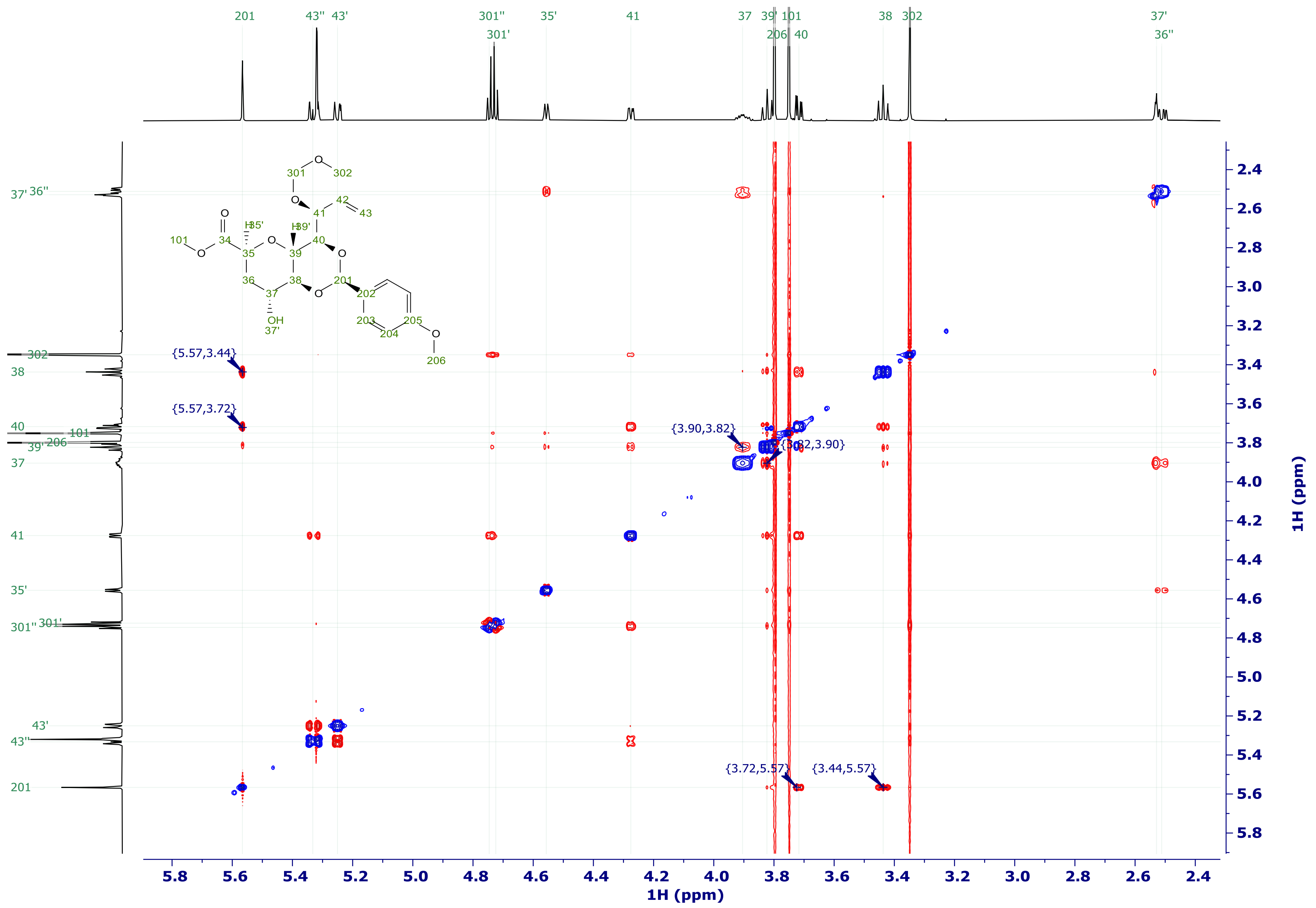

Compound 60: ^{1}H NMR (400 MHz, $CDCl_3$)

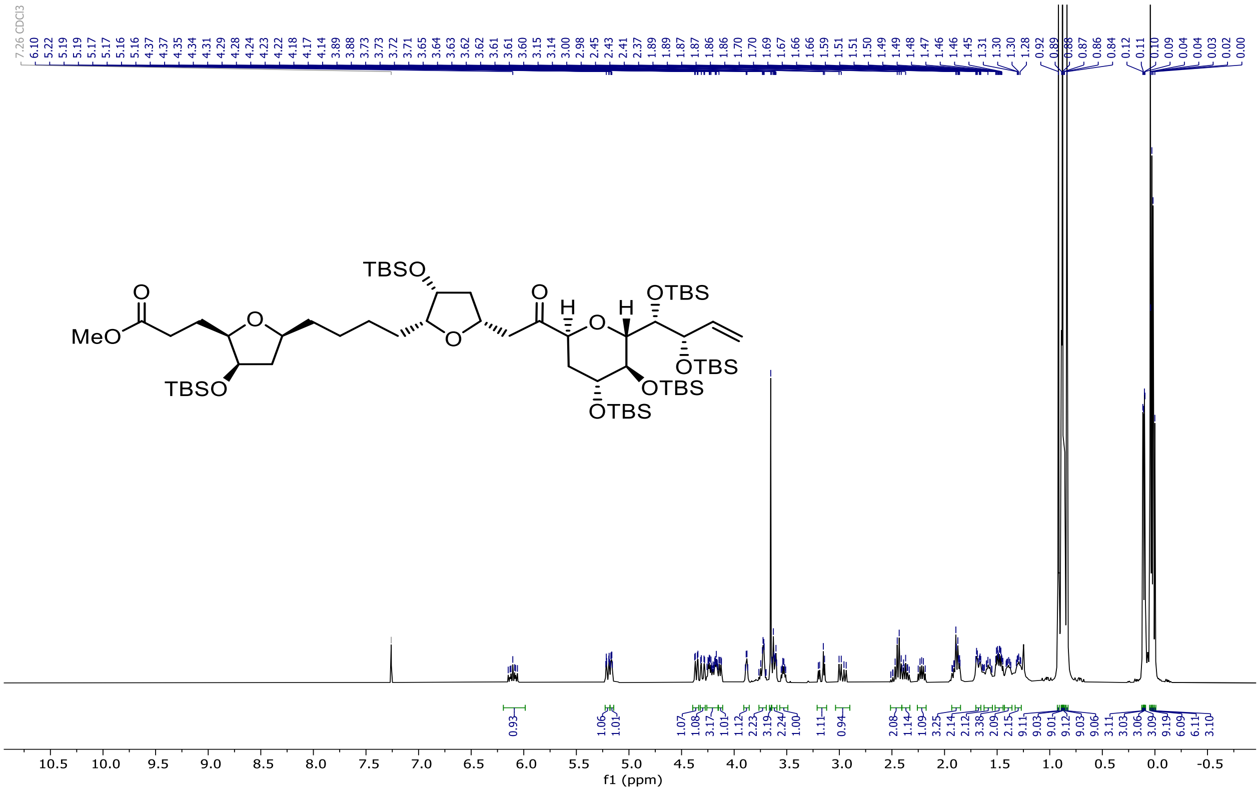

^{13}C NMR (101 MHz, $CDCl_3$)

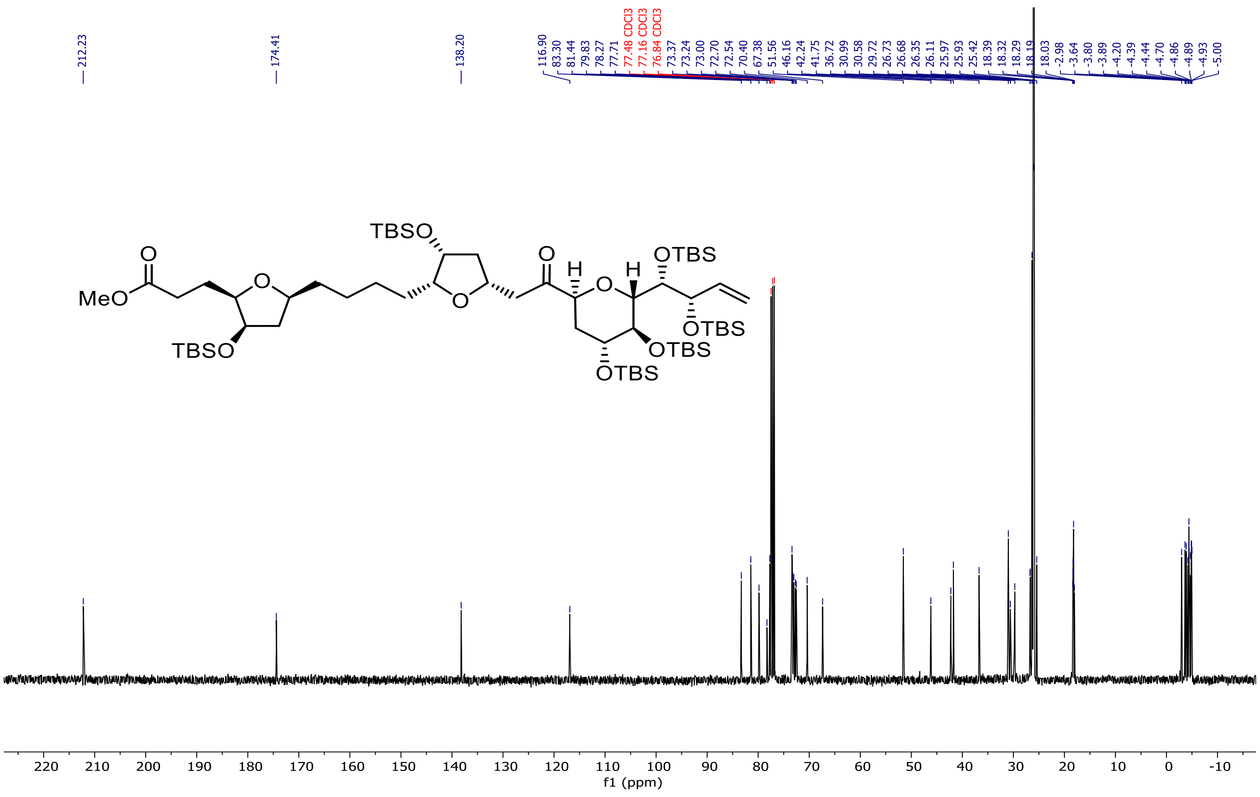

Compound 34*R*-61: ^{1}H NMR (400 MHz, $CDCl_3$)

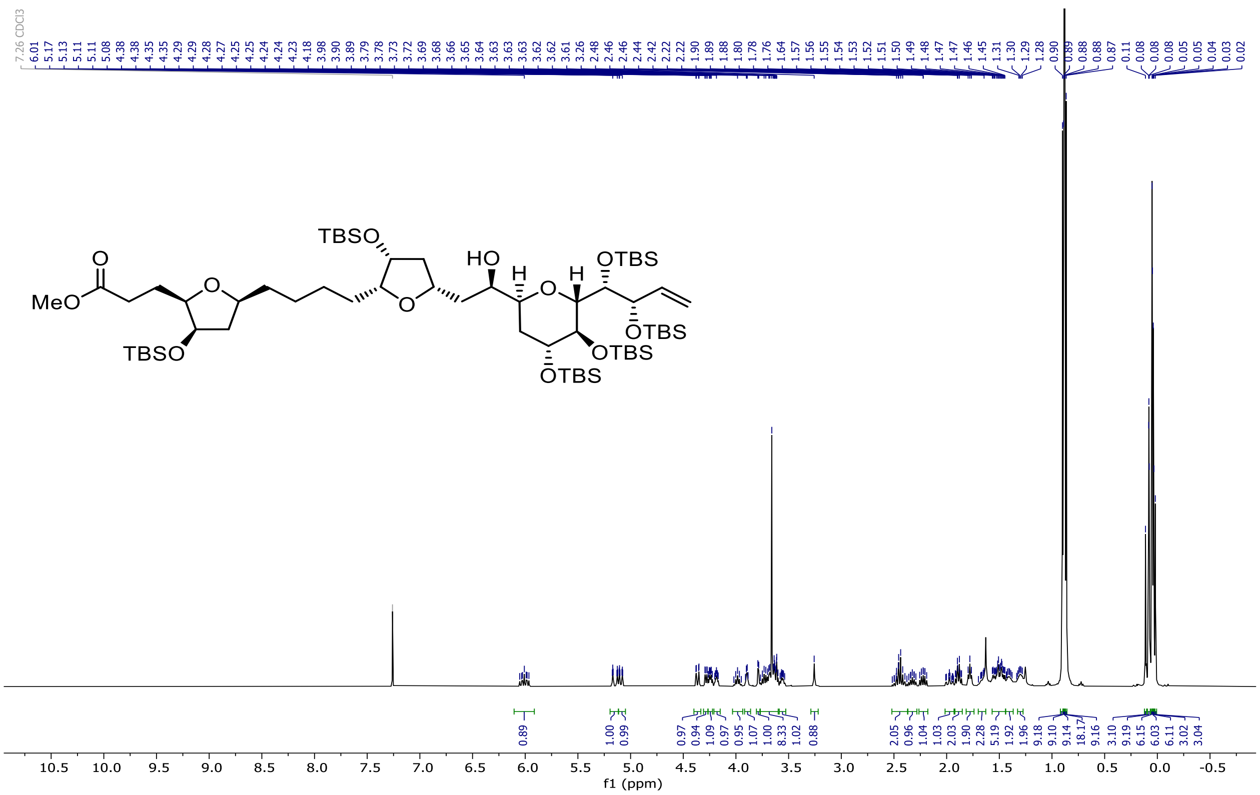

^{13}C NMR (101 MHz, $CDCl_3$)

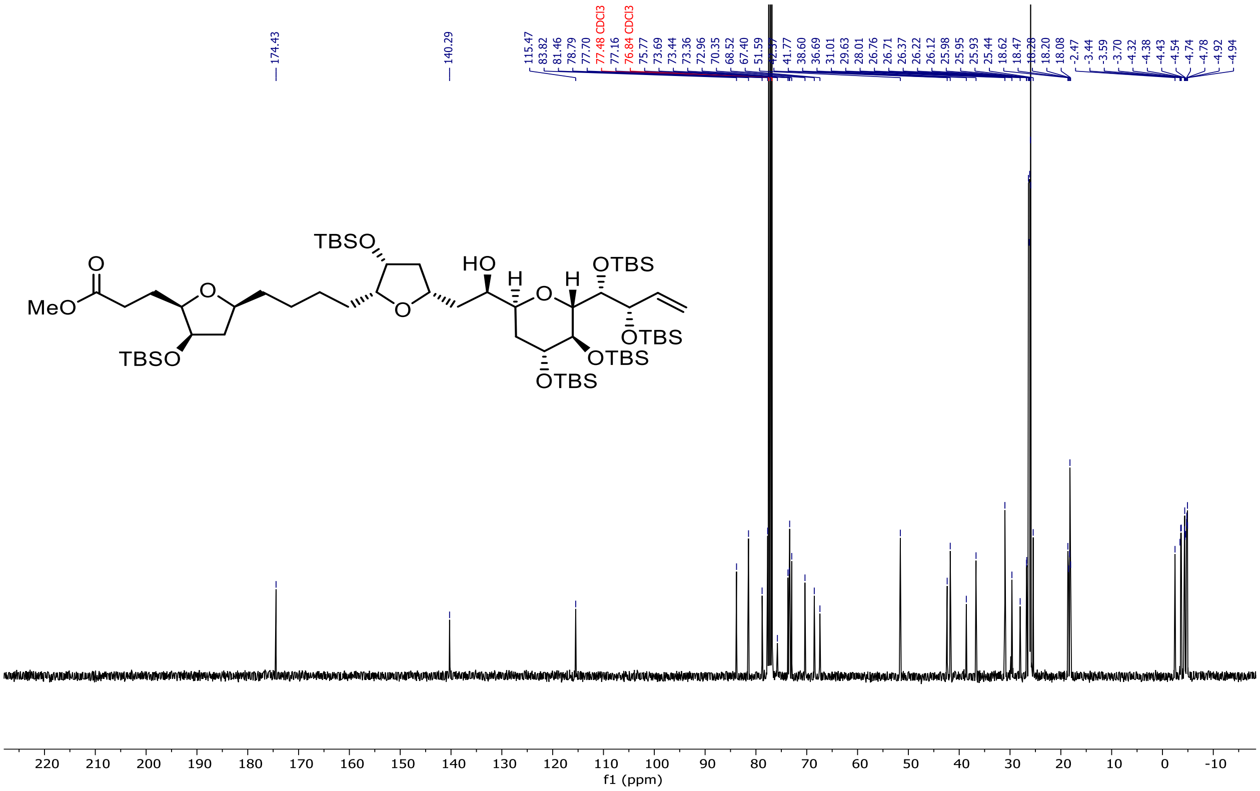

(*R*)-Mosher ester derived from compound 34*R*-61: ^{1}H NMR (600 MHz, [D$_4$]-MeOH)

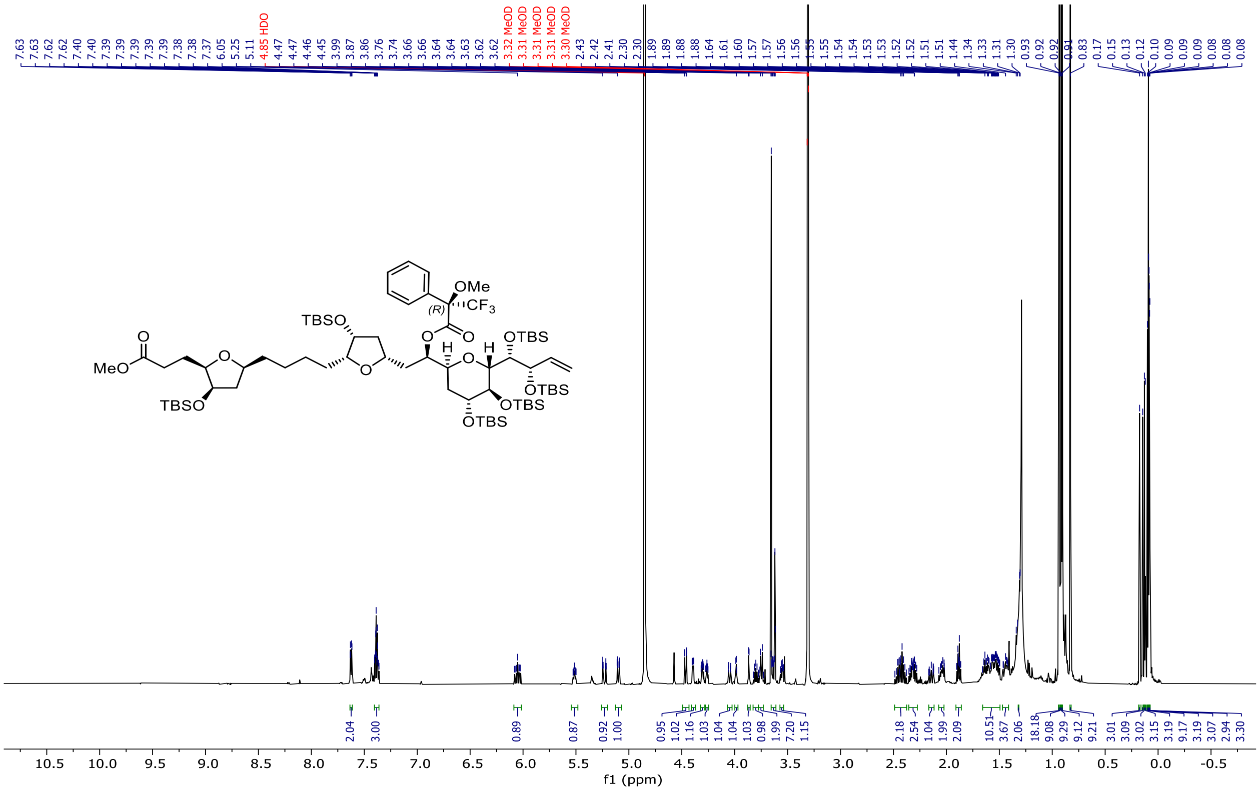

^{13}C NMR (151 MHz, [D$_4$]-MeOH)

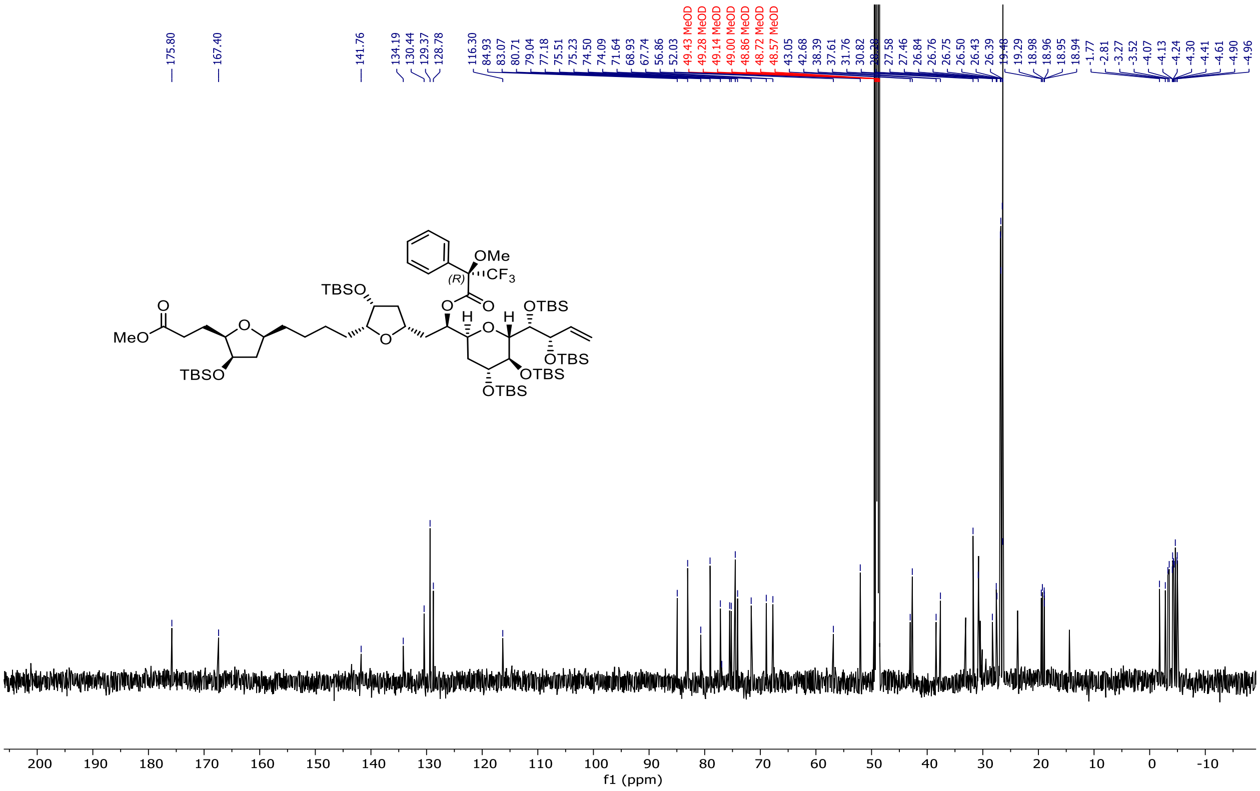

(*R*)-Mosher ester derived from compound 34*R*-61: ^{1}H-^{1}H COSY ([D$_4$]-MeOH)

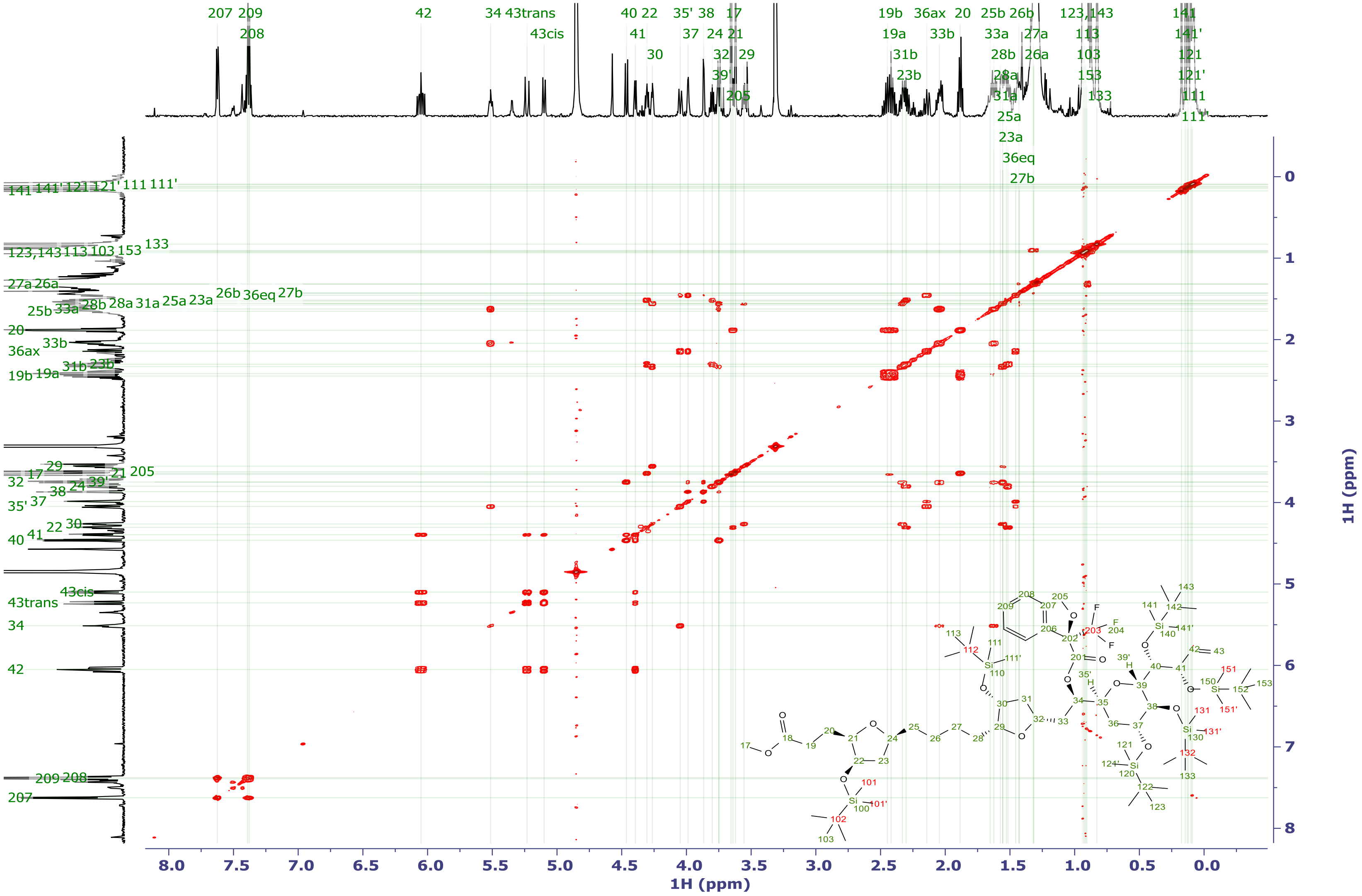

(*R*)-Mosher ester derived from compound 34*R*-61: HSQC NMR ([D$_4$]-MeOH)

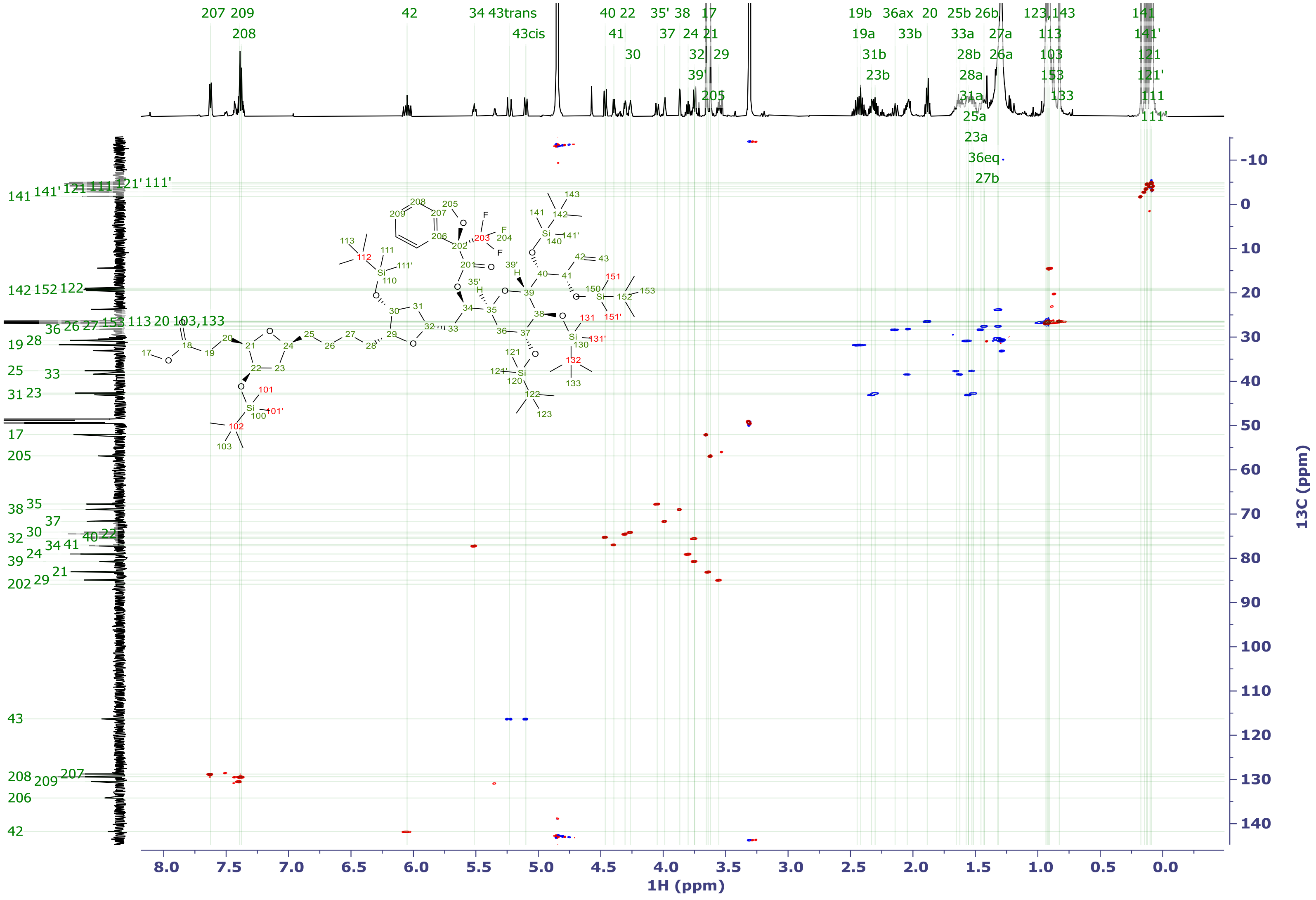

(*R*)-Mosher ester derived from compound 34*R*-61: HMBC NMR ([D_4]-MeOH)

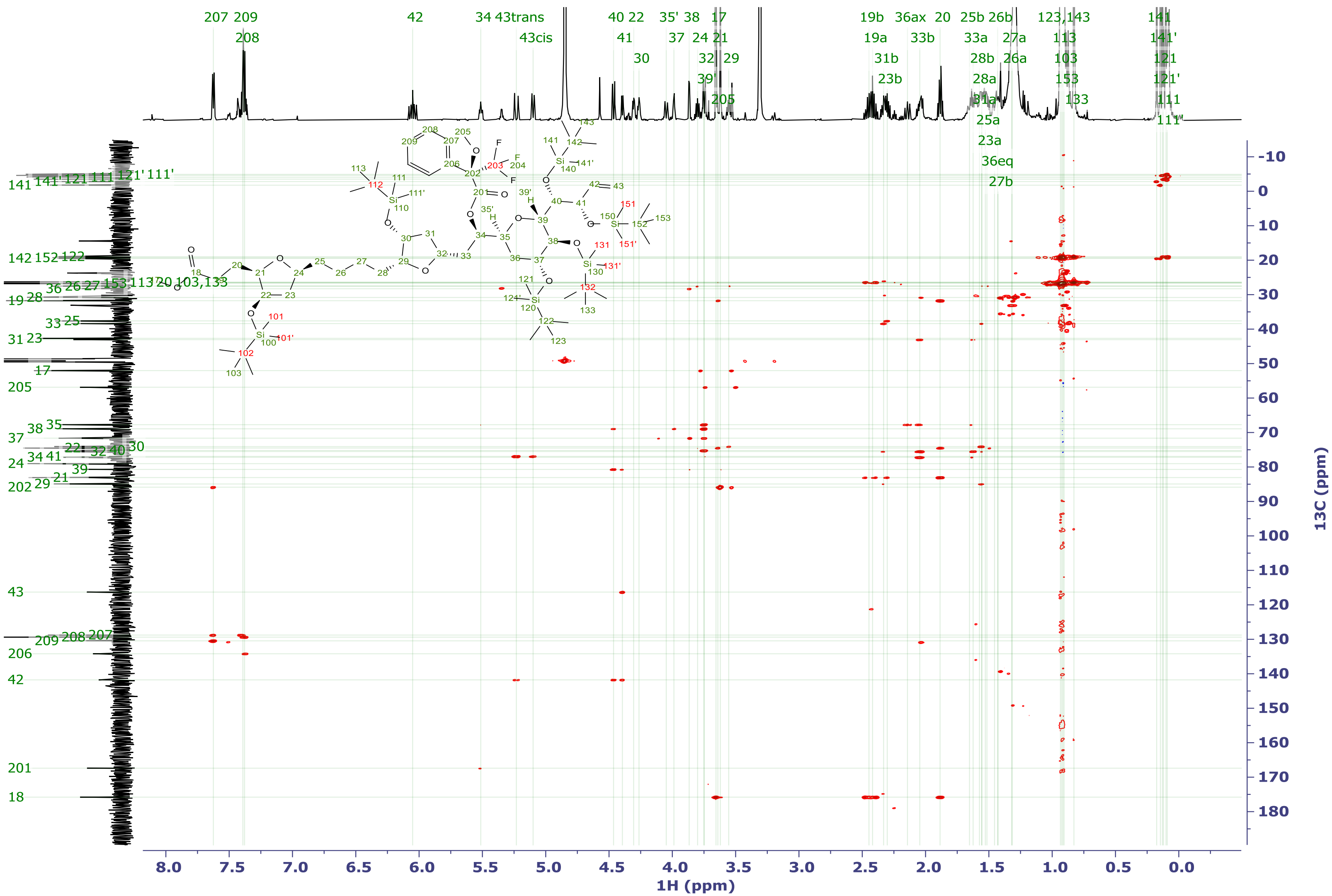

(*R*)-Mosher ester derived from compound 34*R*-61: NOESY ([D$_4$]-MeOH)

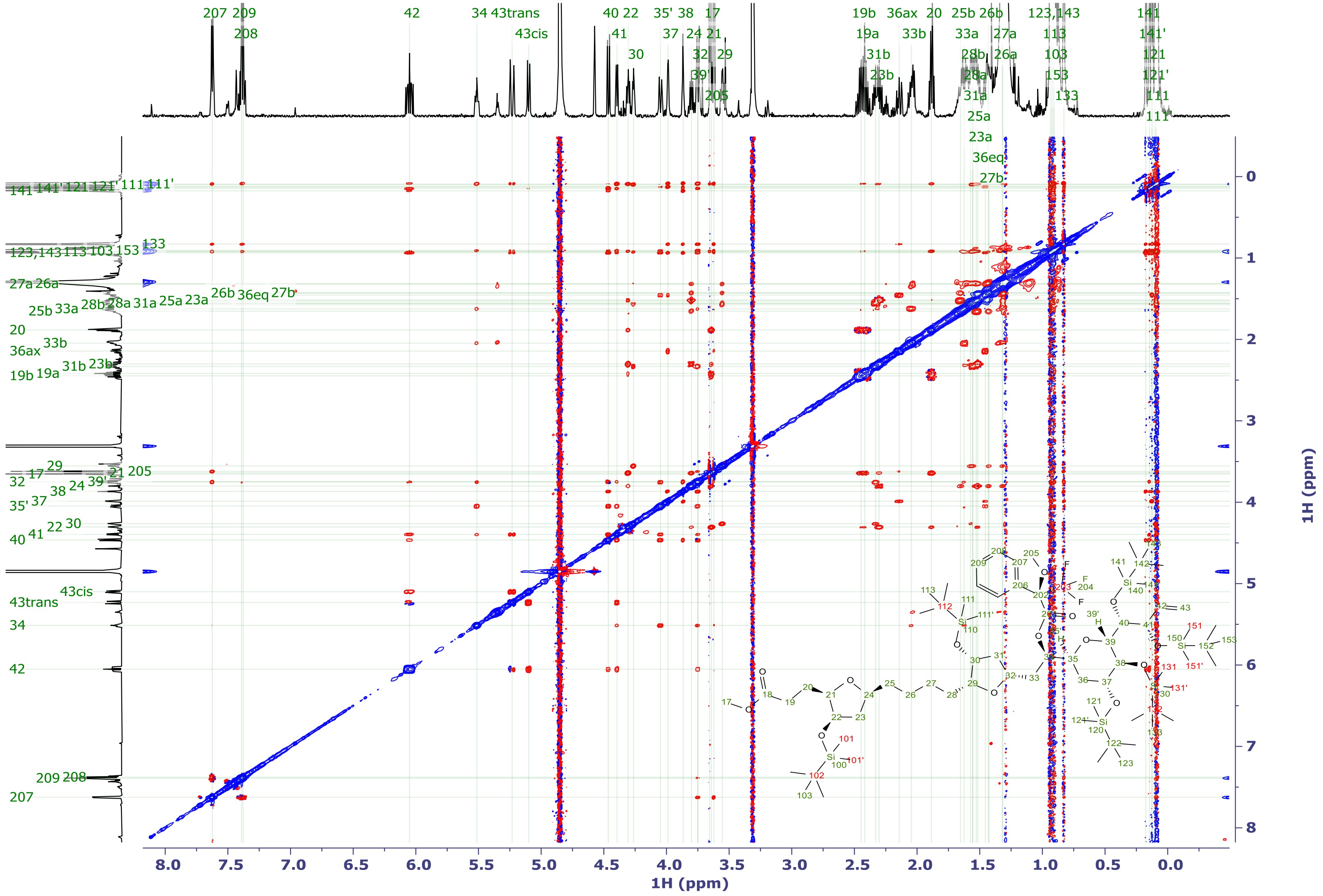

(*S*)-Mosher ester derived from compound 34*R*-61: ^{1}H NMR (600 MHz, [D$_4$]-MeOH)

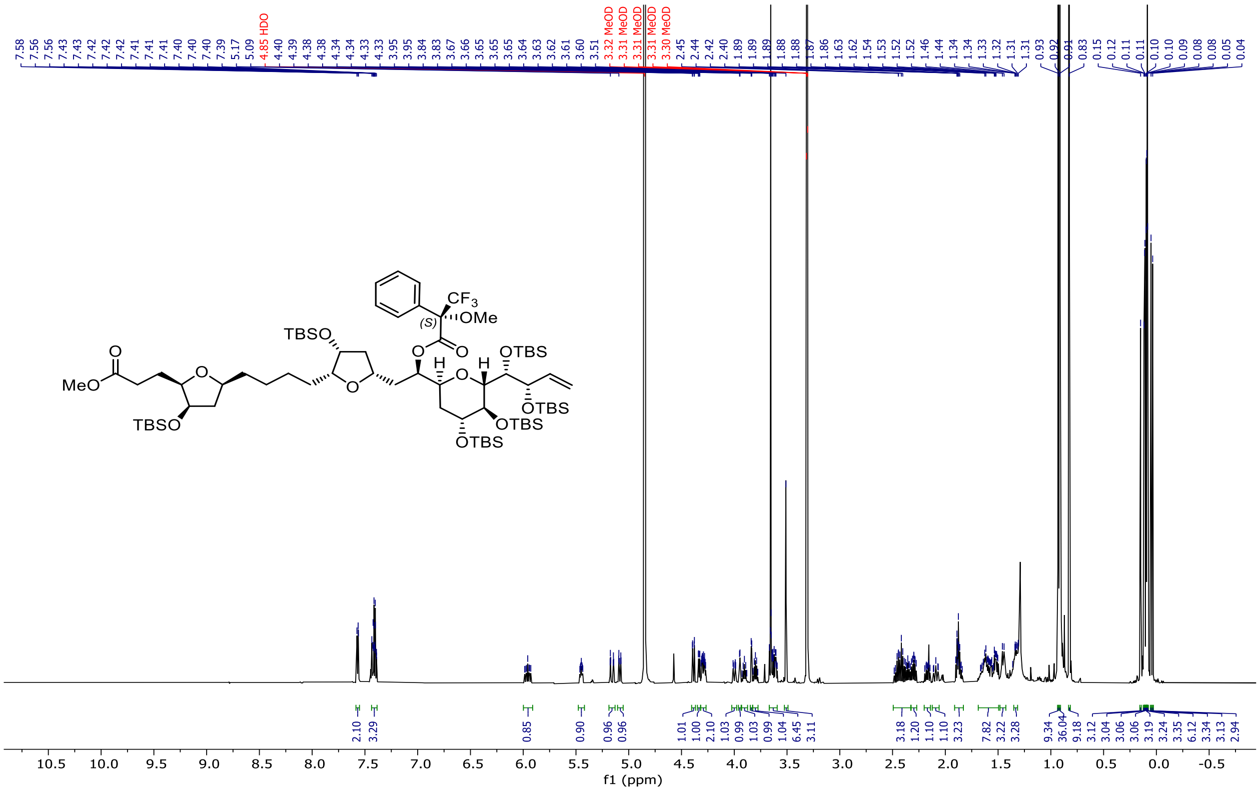

^{13}C NMR (151 MHz, [D$_4$]-MeOH)

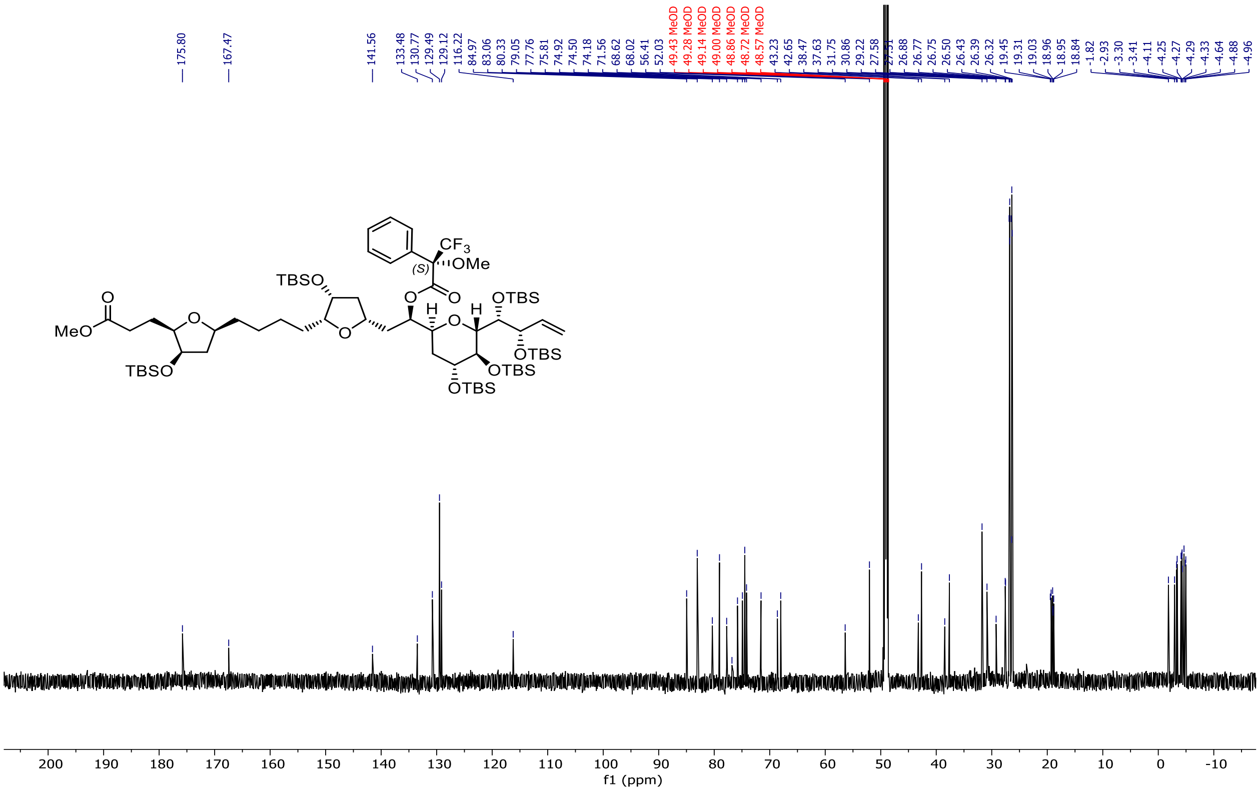

(*S*)-Mosher ester derived from compound 34*R*-61: ^{1}H-^{1}H COSY ([D$_4$]-MeOH)

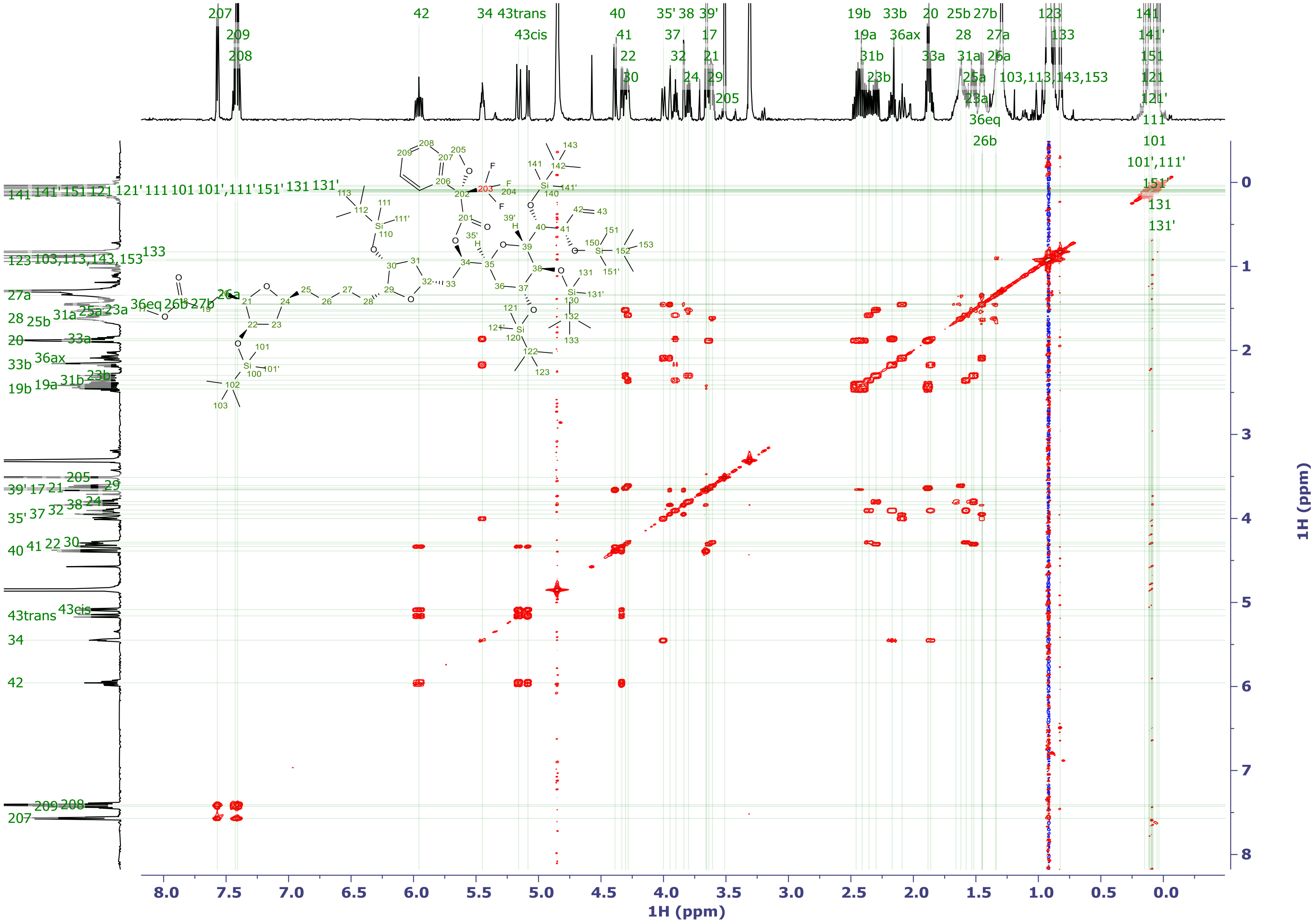

(*S*)-Mosher ester derived from compound 34*R*-61: HSQC NMR ([D$_4$]-MeOH)

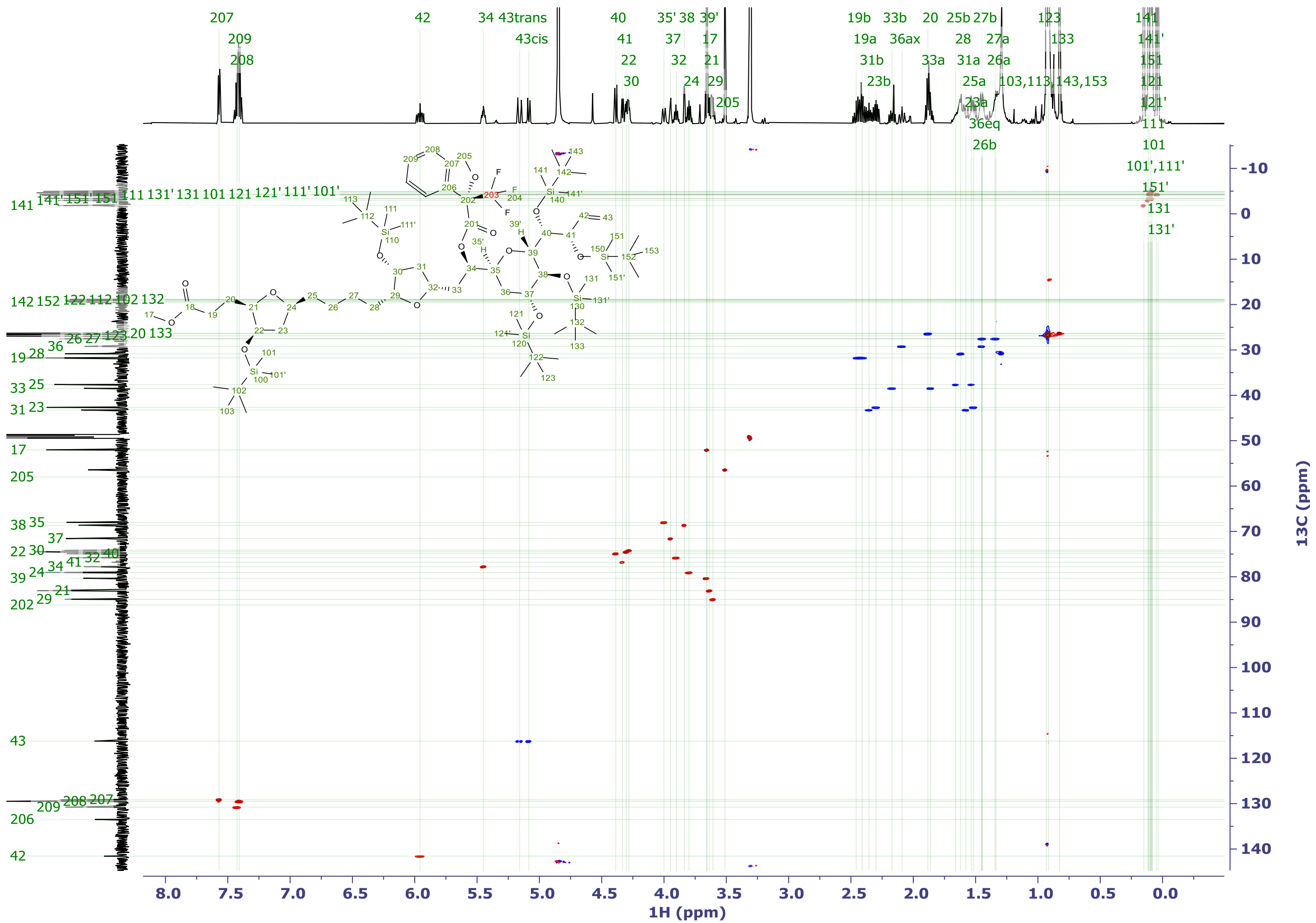

(*S*)-Mosher ester derived from compound 34*R*-61: HMBC NMR ([D$_4$]-MeOH)

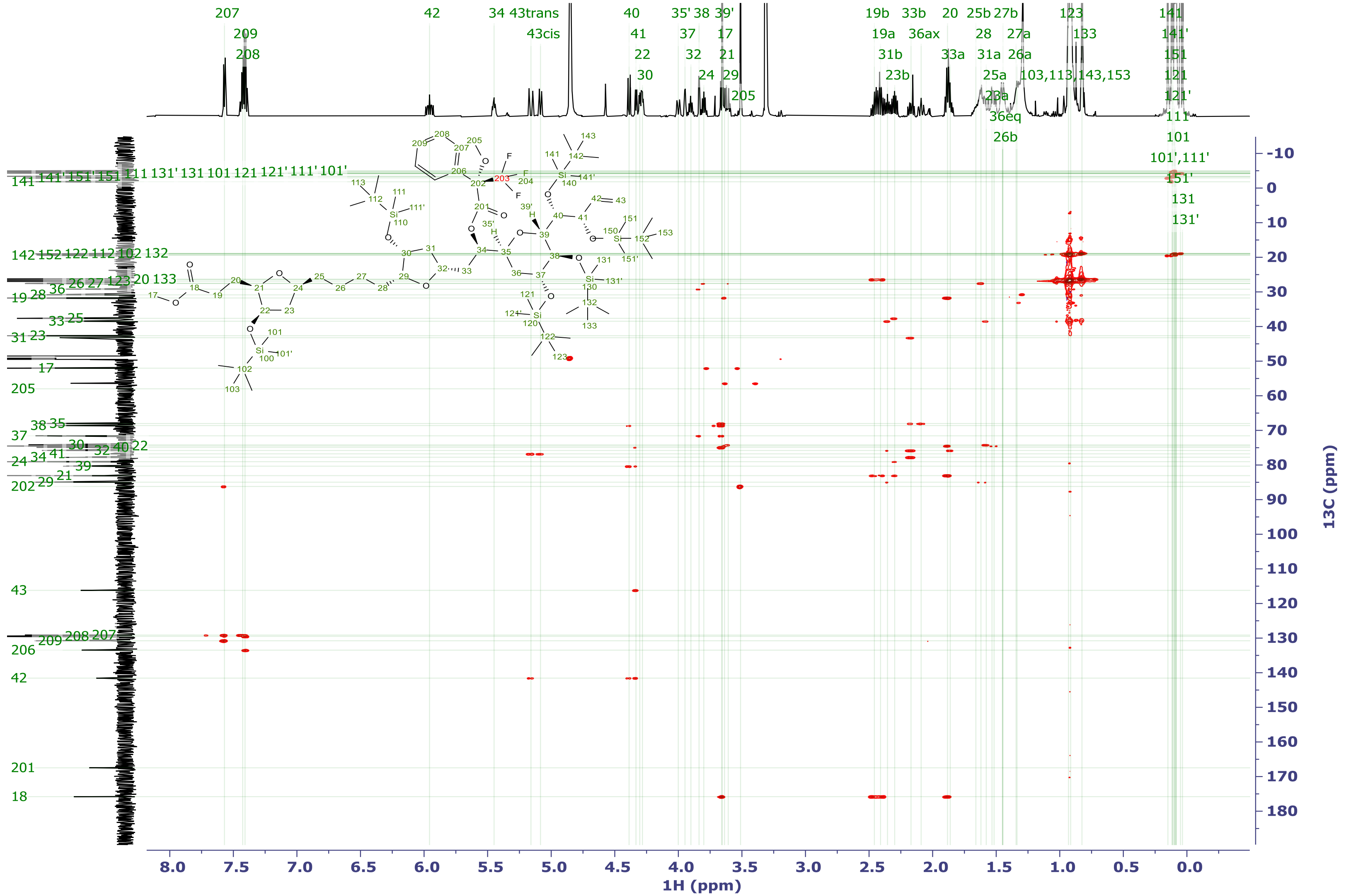

(*S*)-Mosher ester derived from compound 34*R*-61: NOESY ([D$_4$]-MeOH)

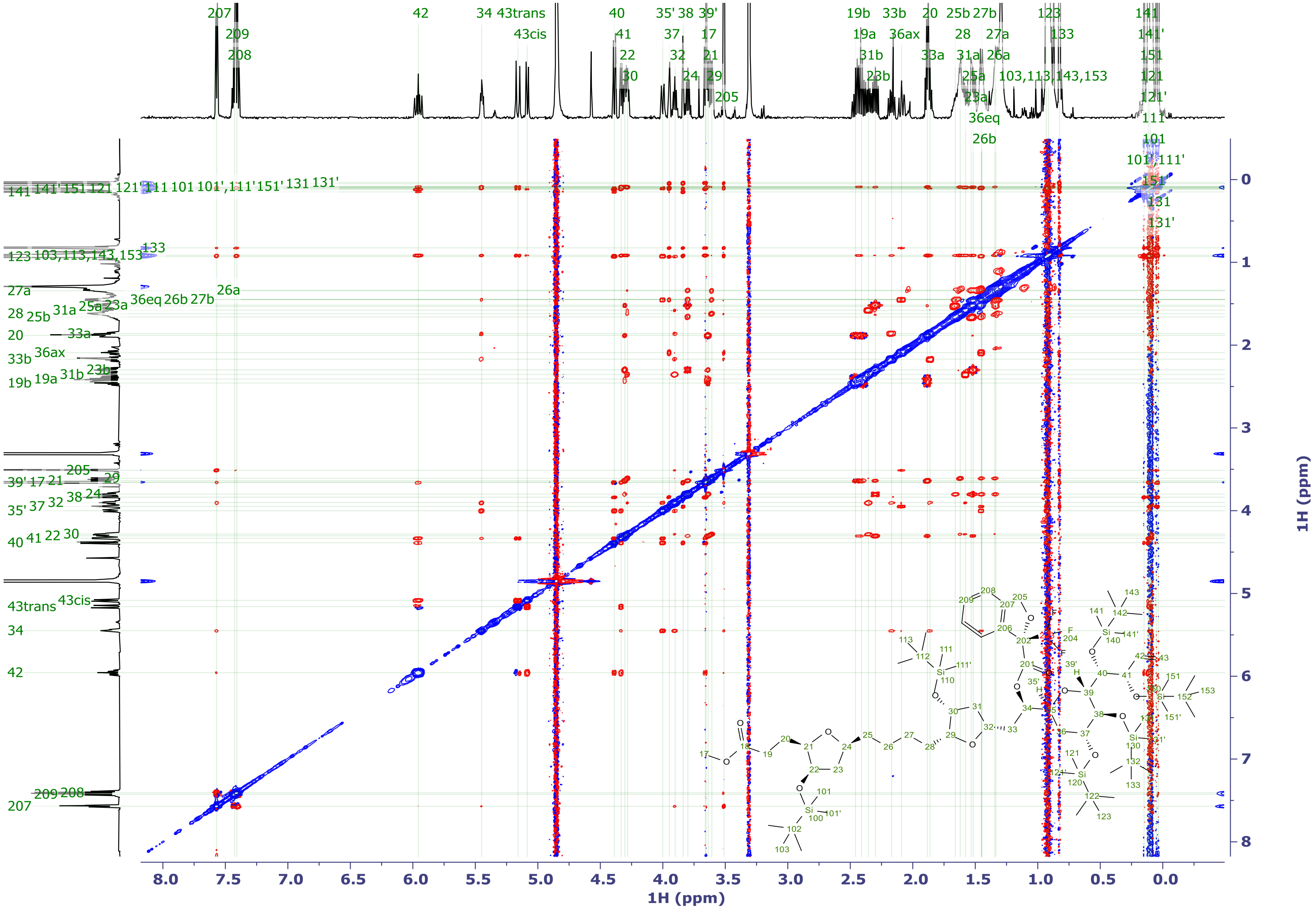

Compound 34*S*-61: ^{1}H NMR (400 MHz, $CDCl_3$)

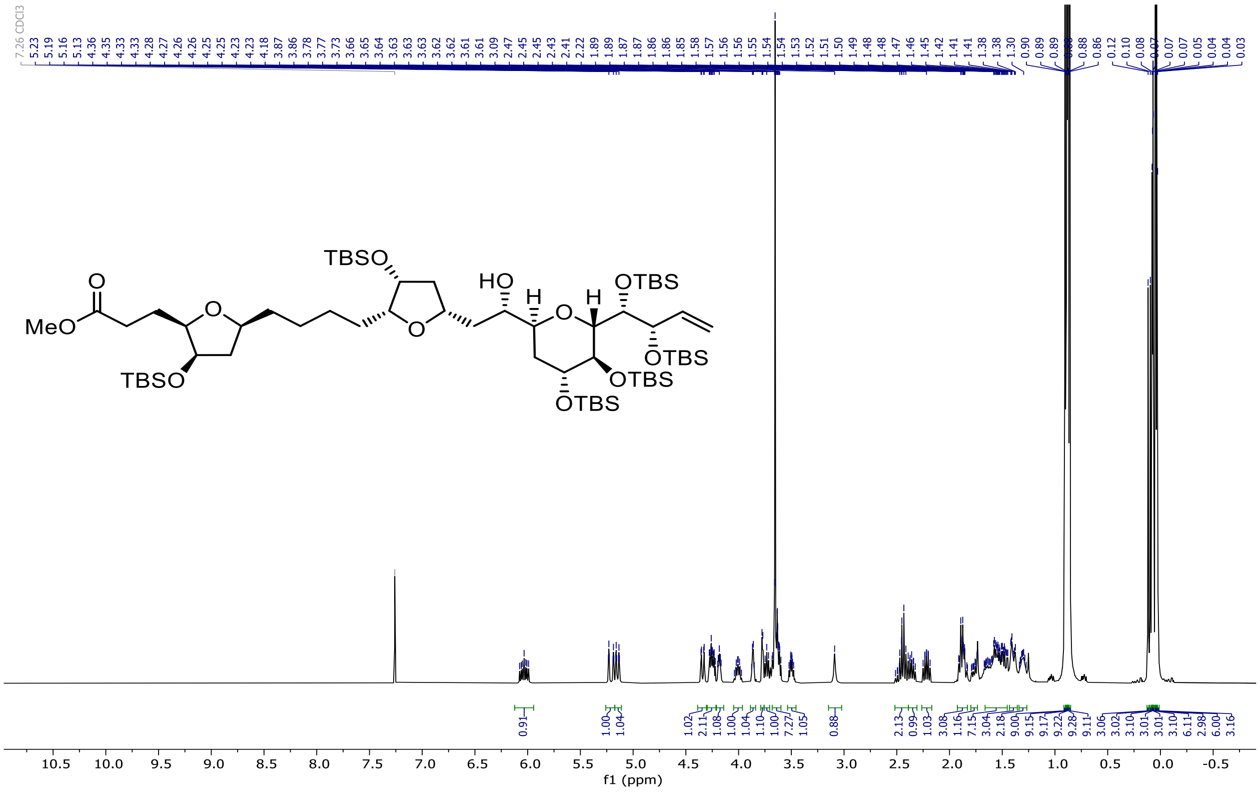

^{13}C NMR (101 MHz, $CDCl_3$)

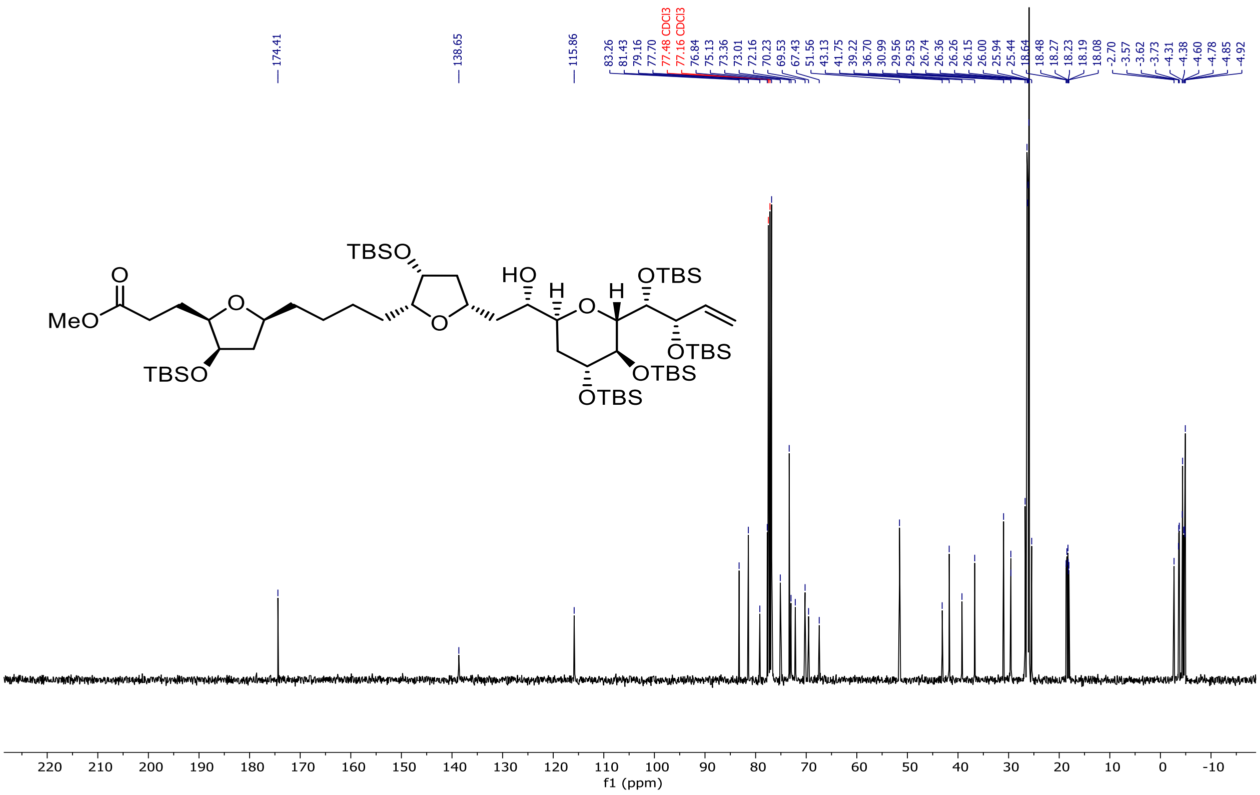

(*R*)-Mosher ester derived from compound 34*S*-61: ^{1}H NMR (600 MHz, [D$_4$]-MeOH)

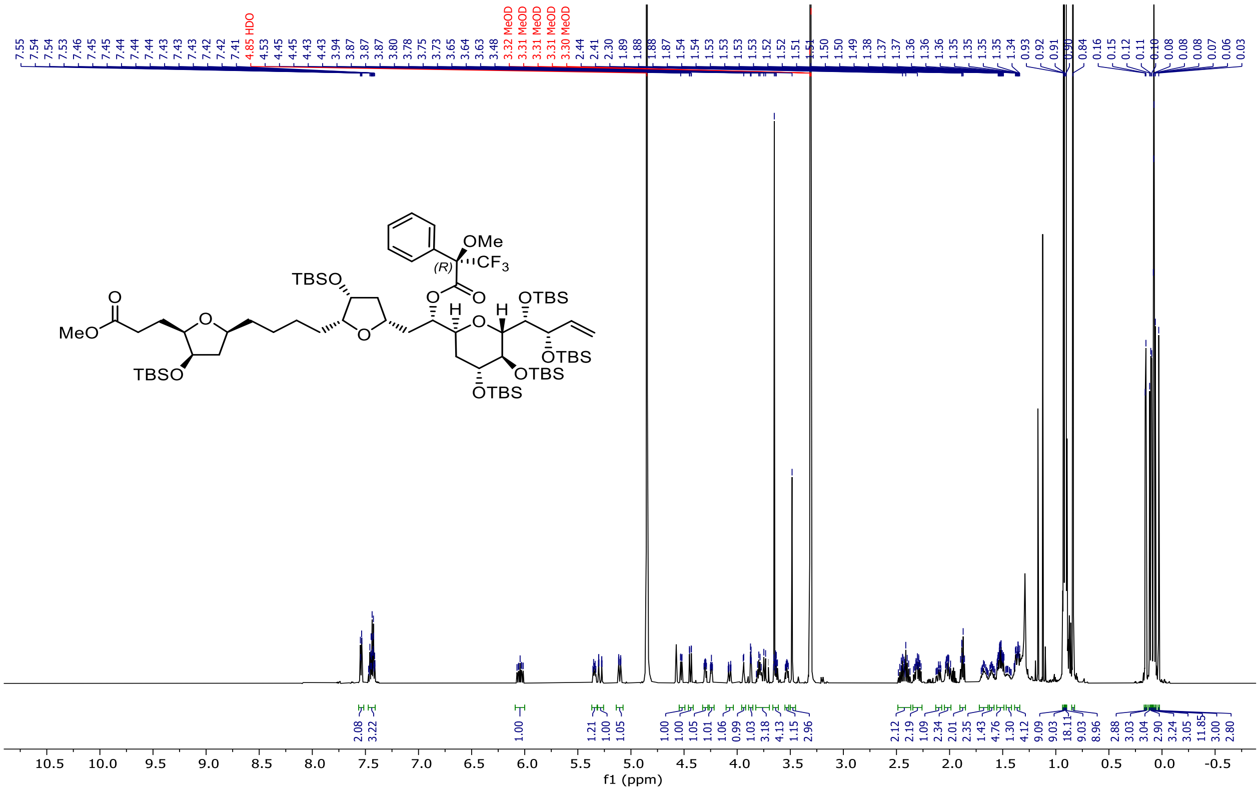

^{13}C NMR (151 MHz, [D$_4$]-MeOH)

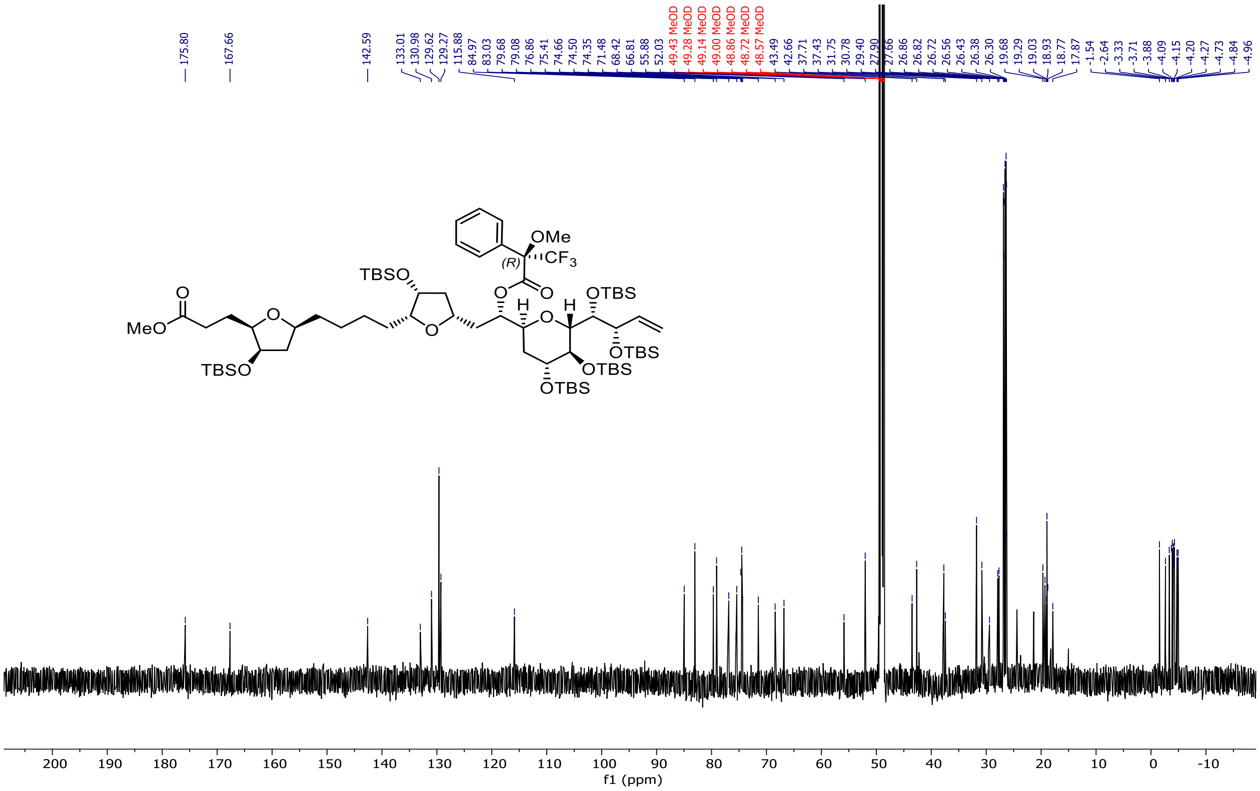

(*R*)-Mosher ester derived from compound 34*S*-61: ^{1}H-^{1}H COSY ([D$_4$]-MeOH)

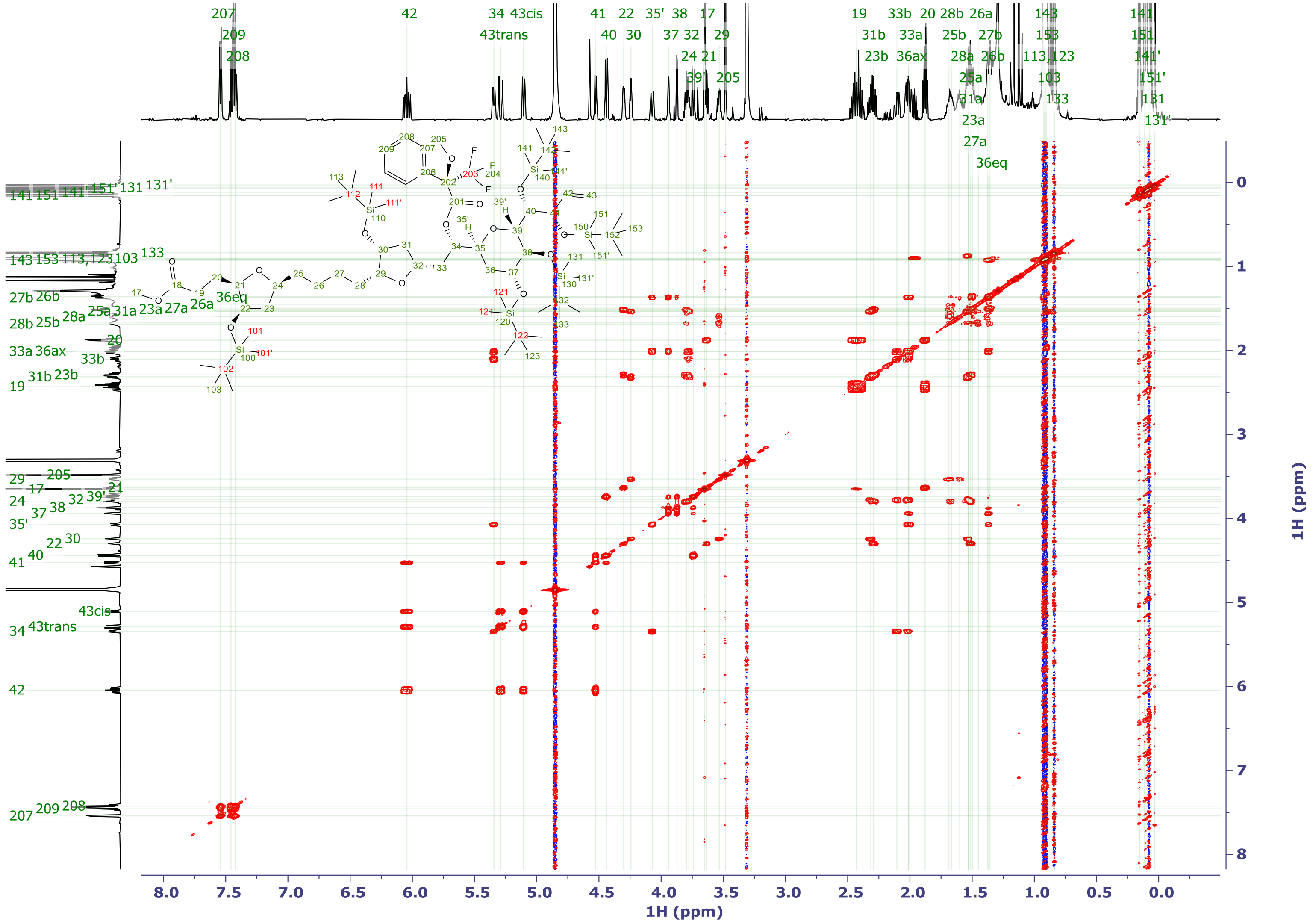

(*R*)-Mosher ester derived from compound 34*S*-61: HSQC NMR ([D$_4$]-MeOH)

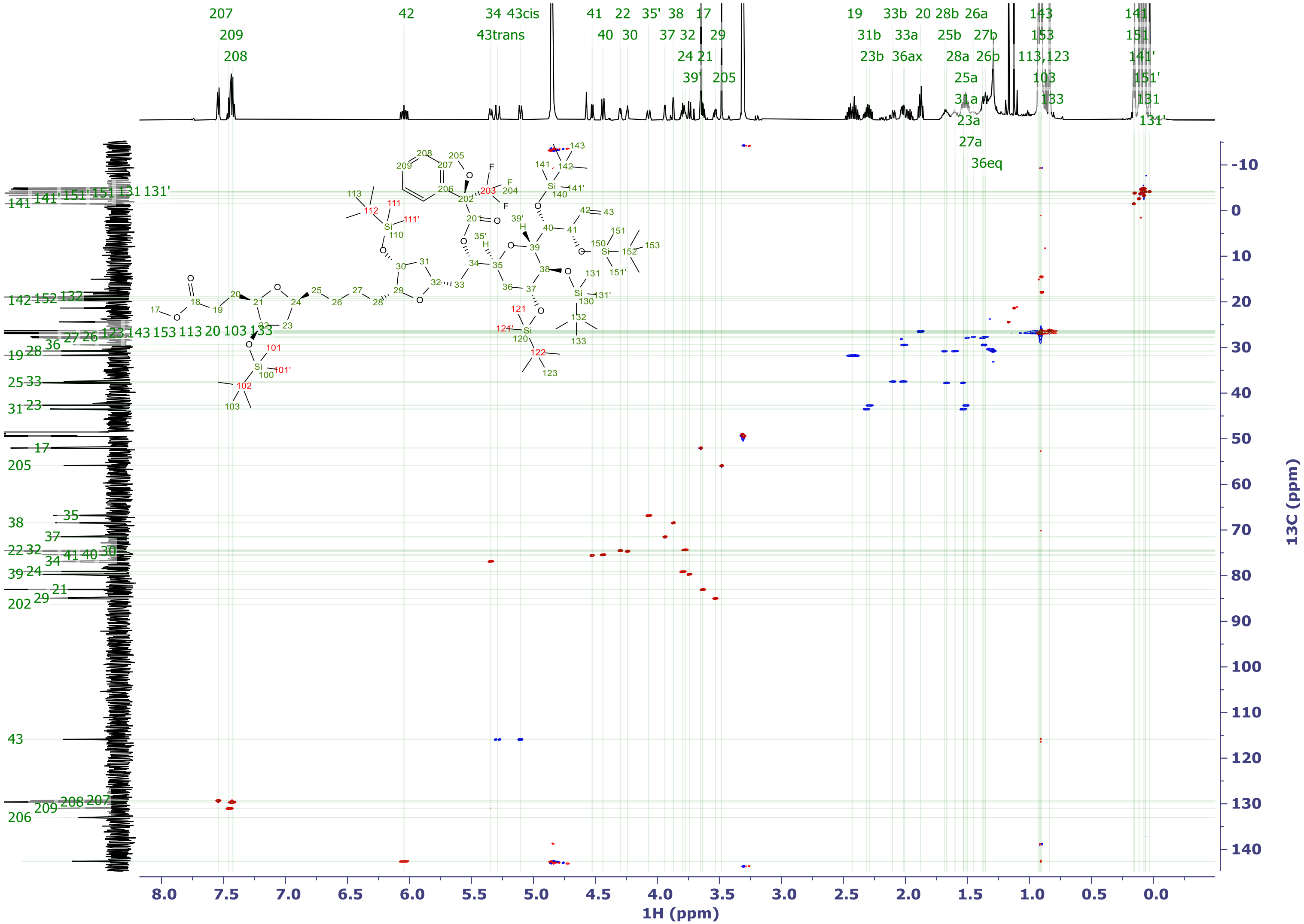

(*R*)-Mosher ester derived from compound 34*S*-61: HMBC NMR ([D$_4$]-MeOH)

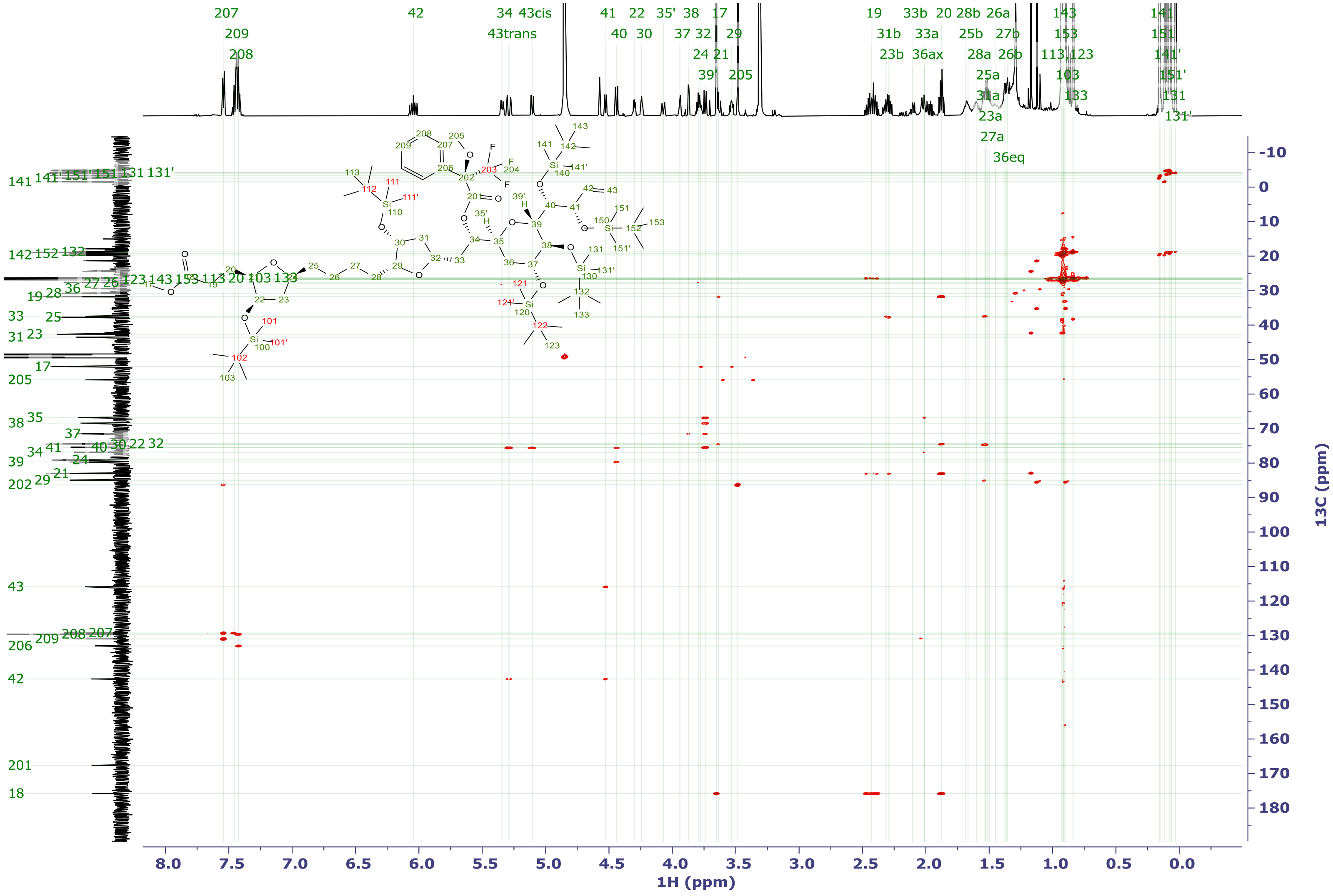

(*R*)-Mosher ester derived from compound 34*S*-61: NOESY ([D4]-MeOH)

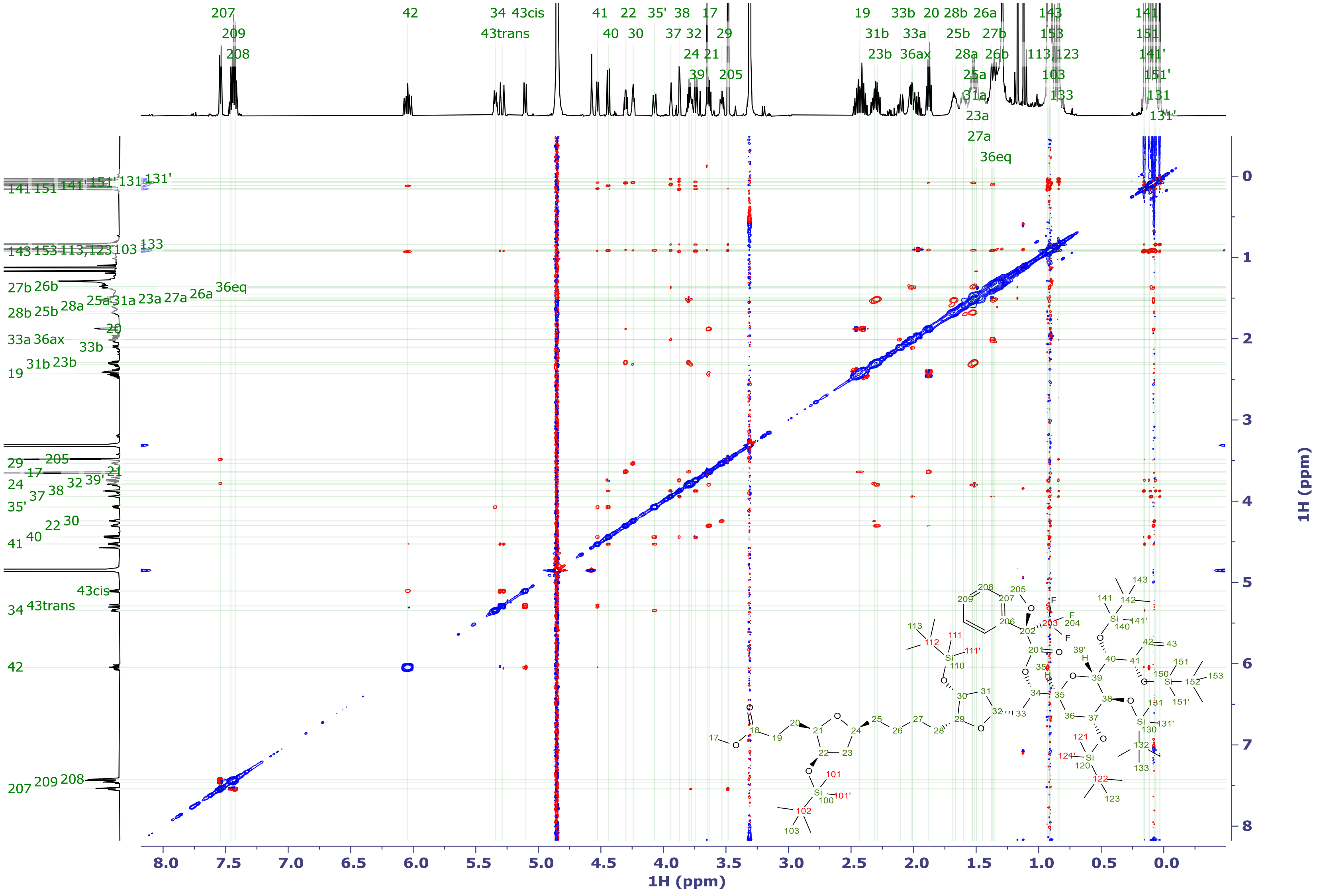

(*S*)-Mosher ester derived from compound 34*S*-61: ^{1}H NMR (600 MHz, [D$_4$]-MeOH)

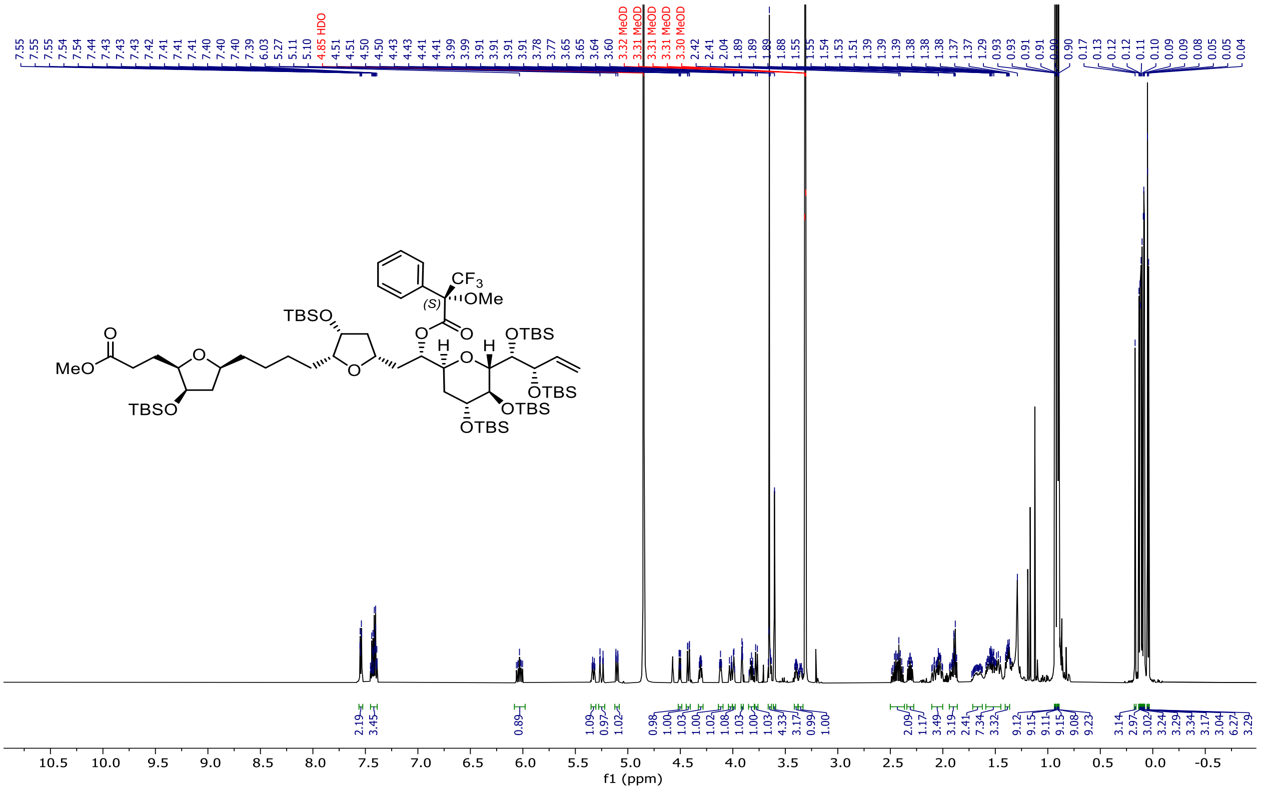

^{13}C NMR (151 MHz, [D$_4$]-MeOH)

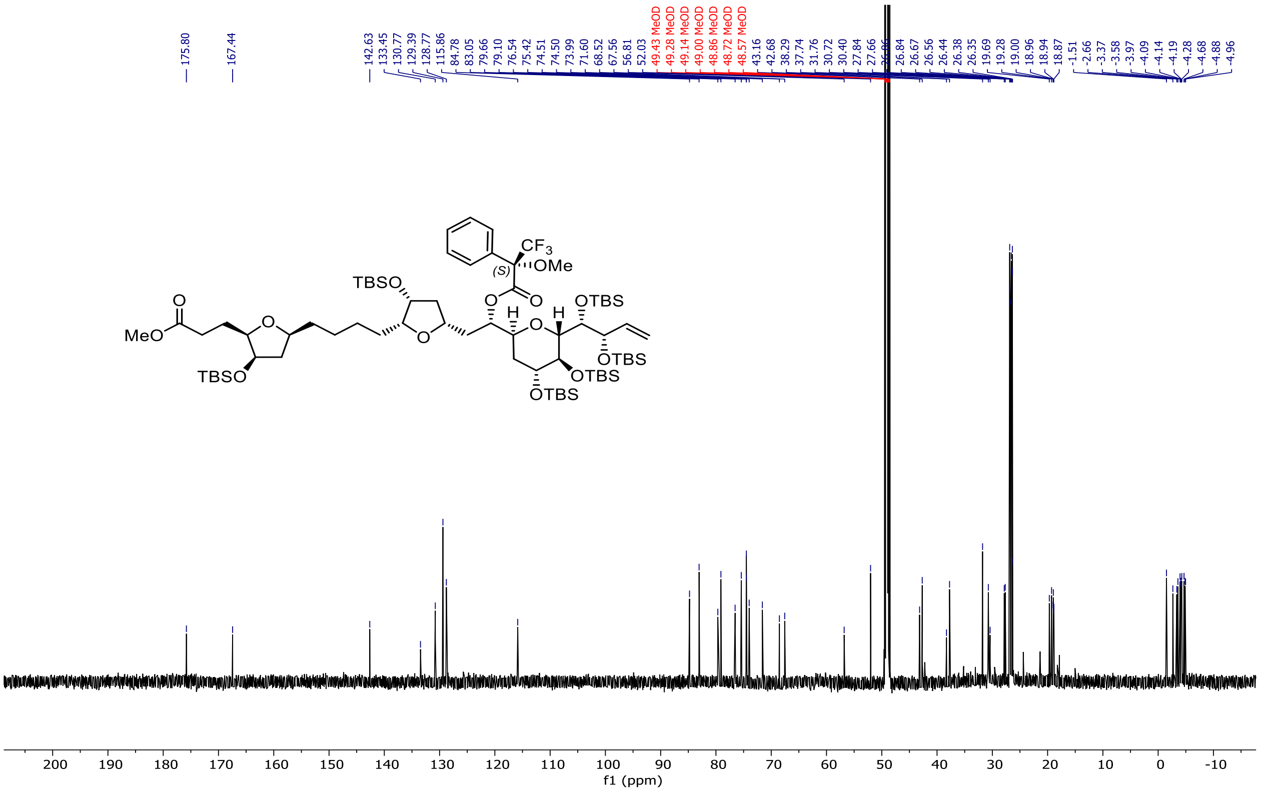

(*S*)-Mosher ester derived from compound 34*S*-61: ^{1}H-^{1}H COSY ([D$_4$]-MeOH)

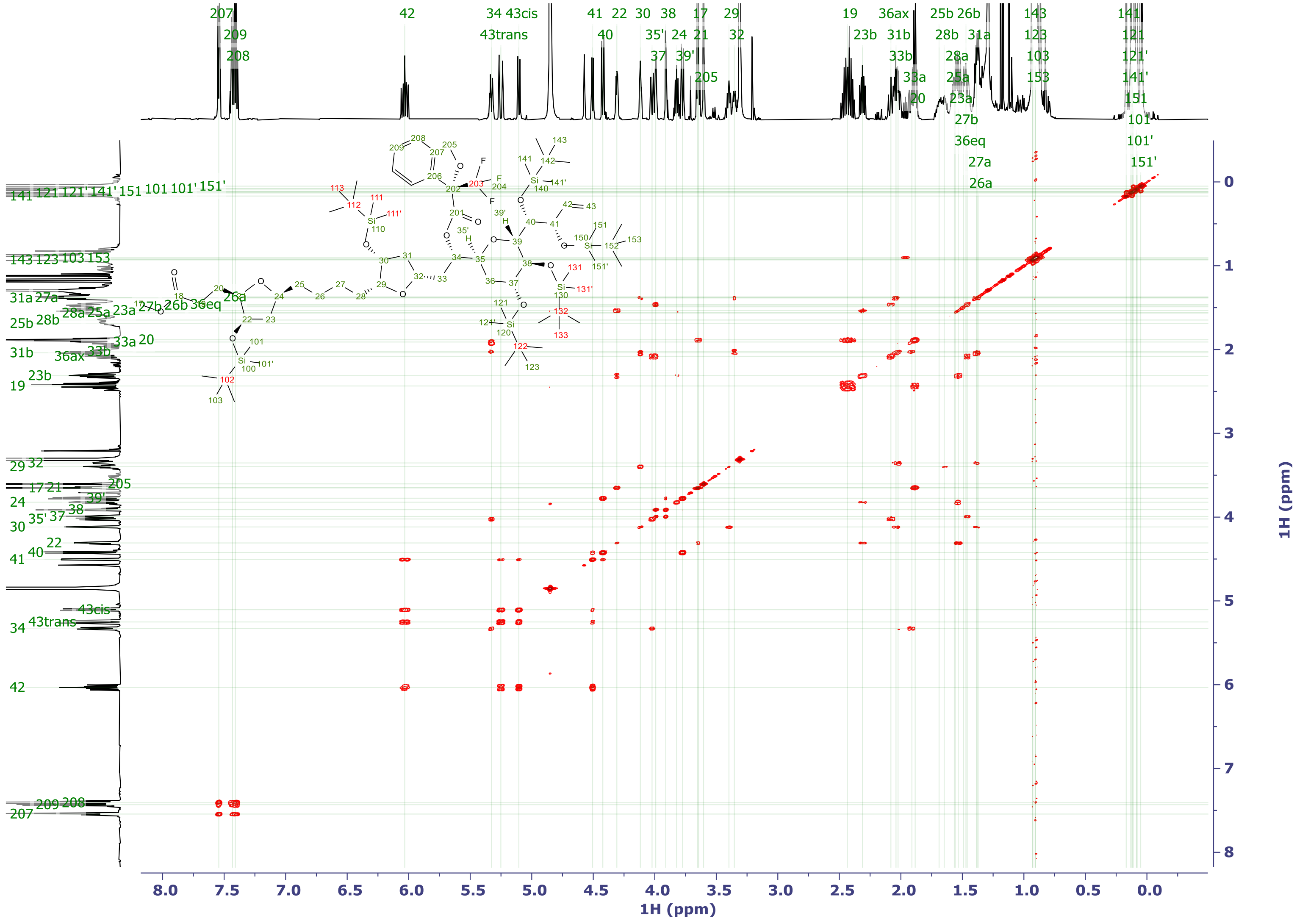

(*S*)-Mosher ester derived from compound 34*S*-61: HSQC NMR ([D$_4$]-MeOH)

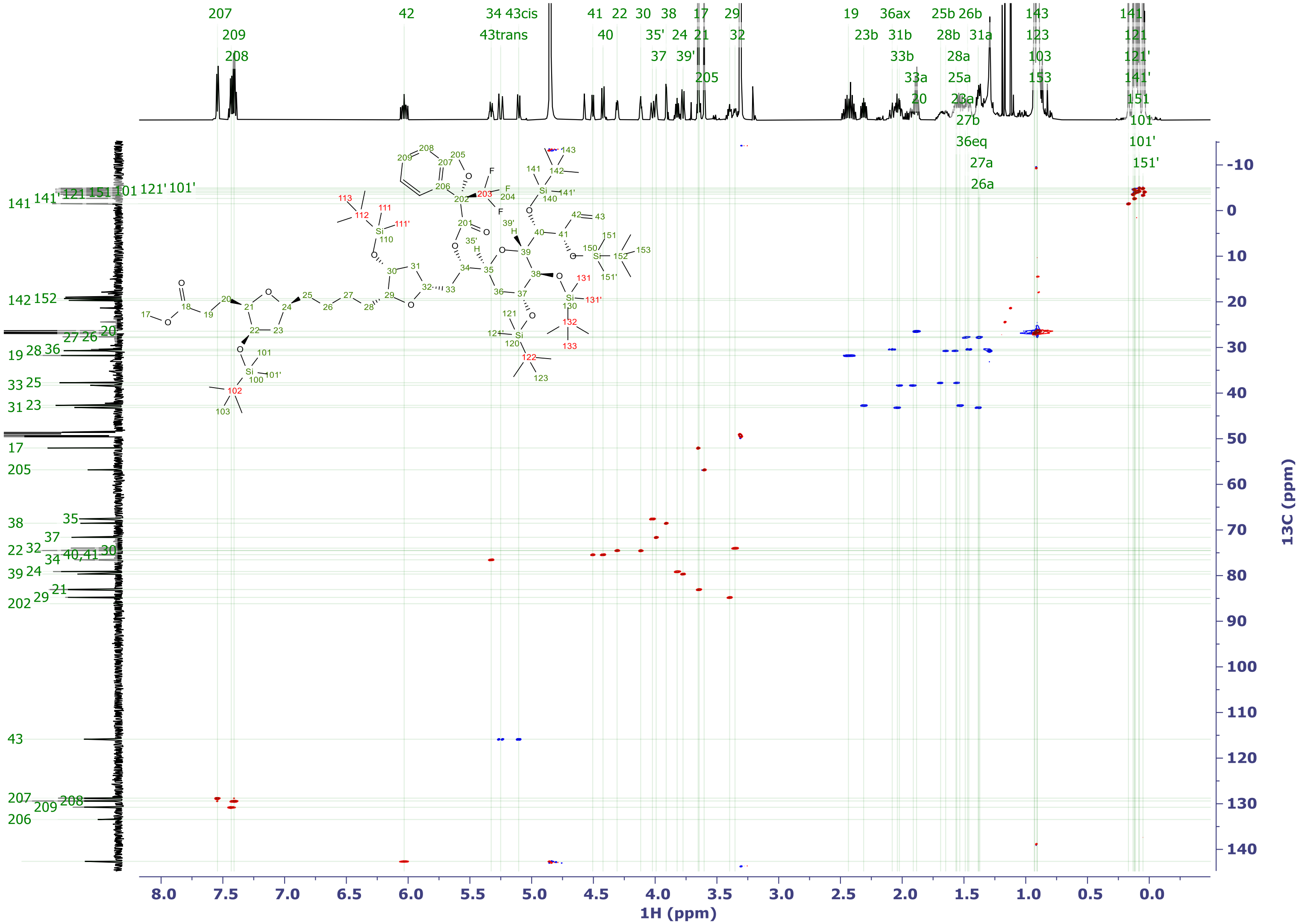

(*S*)-Mosher ester derived from compound 34*S*-61: HMBC NMR ([D$_4$]-MeOH)

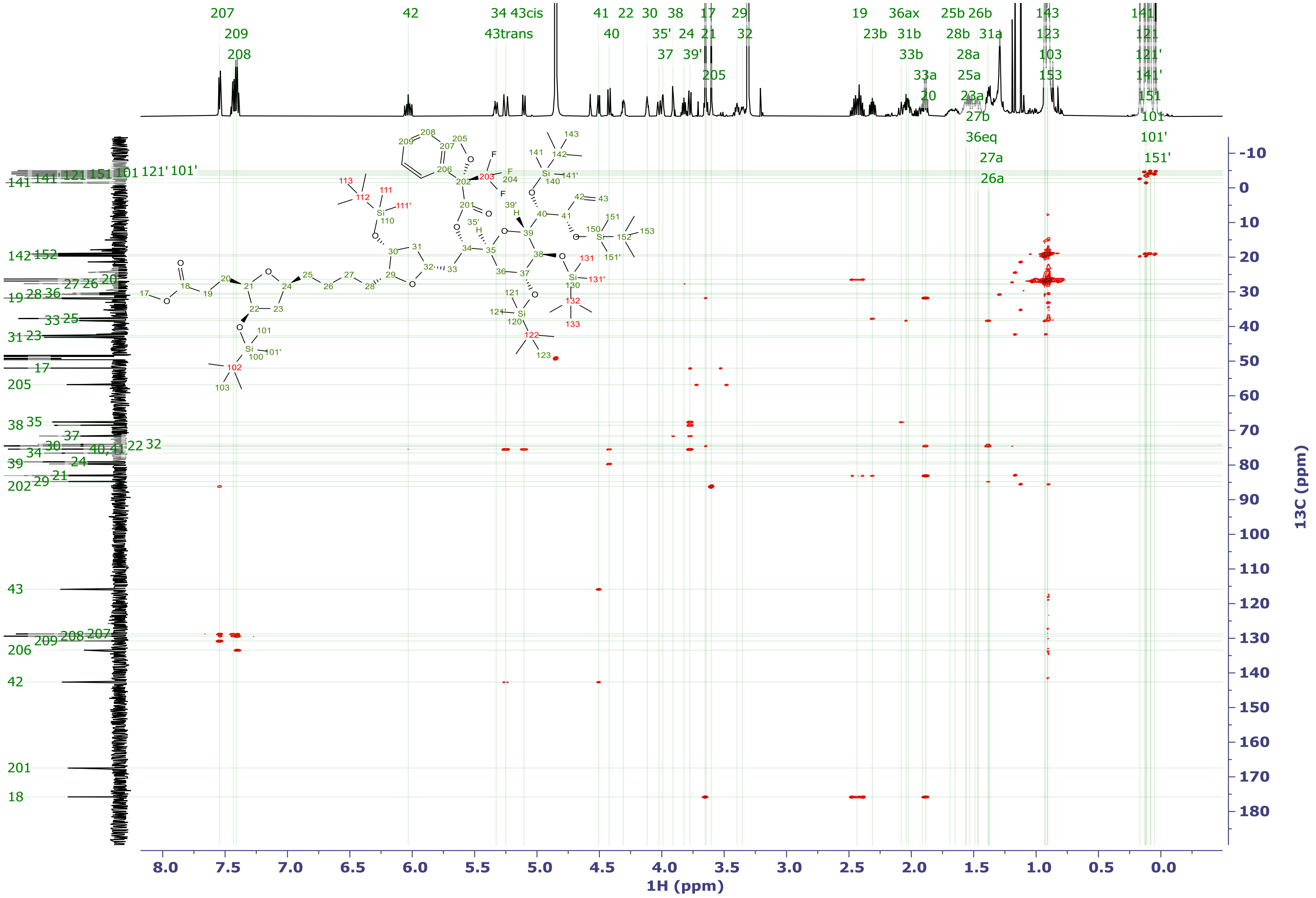

(*S*)-Mosher ester derived from compound 34*S*-61: NOESY ([D$_4$]-MeOH)

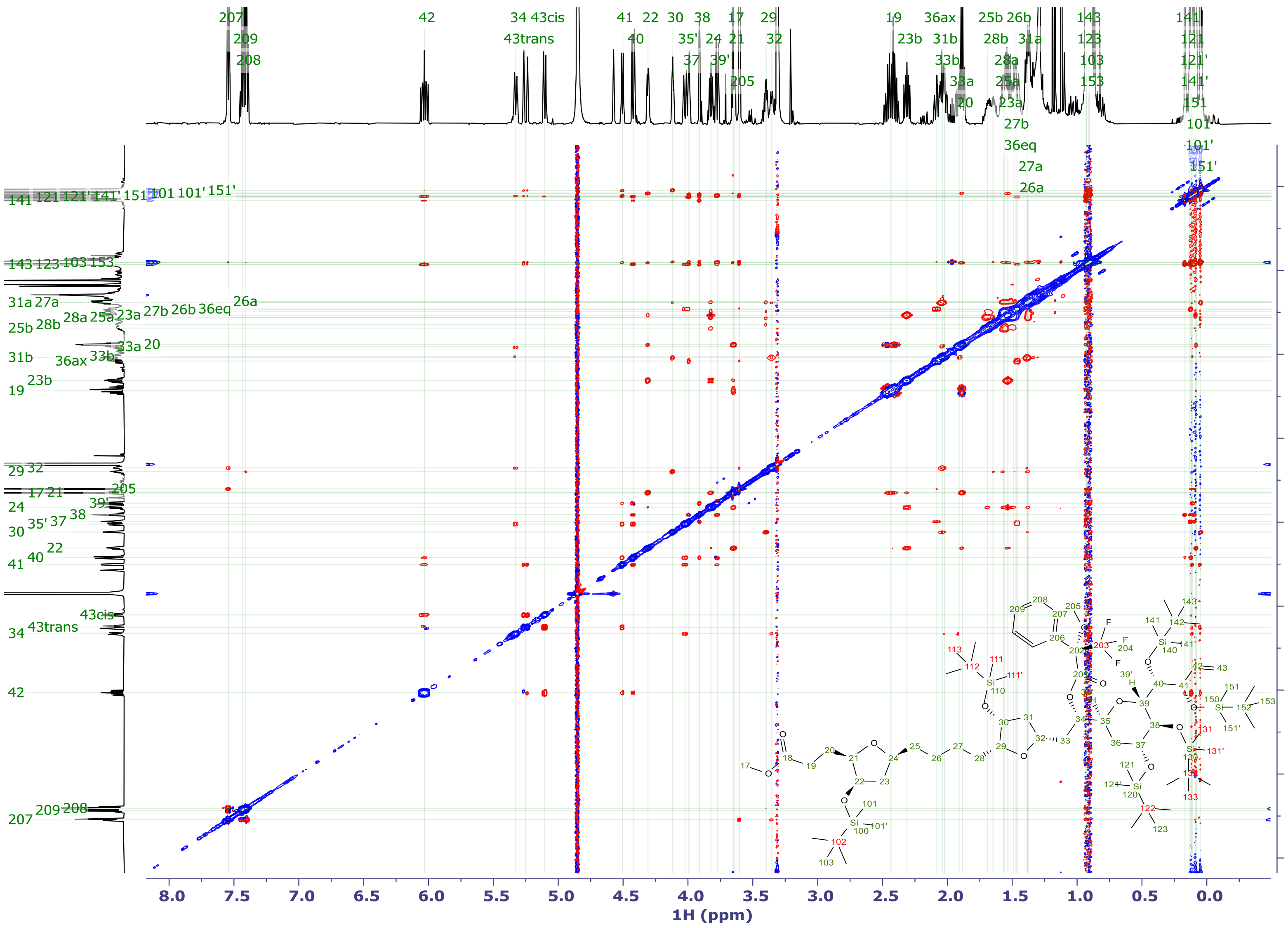

Compound 34*S*-62: ^{1}H NMR (600 MHz, [D$_4$]-MeOH)

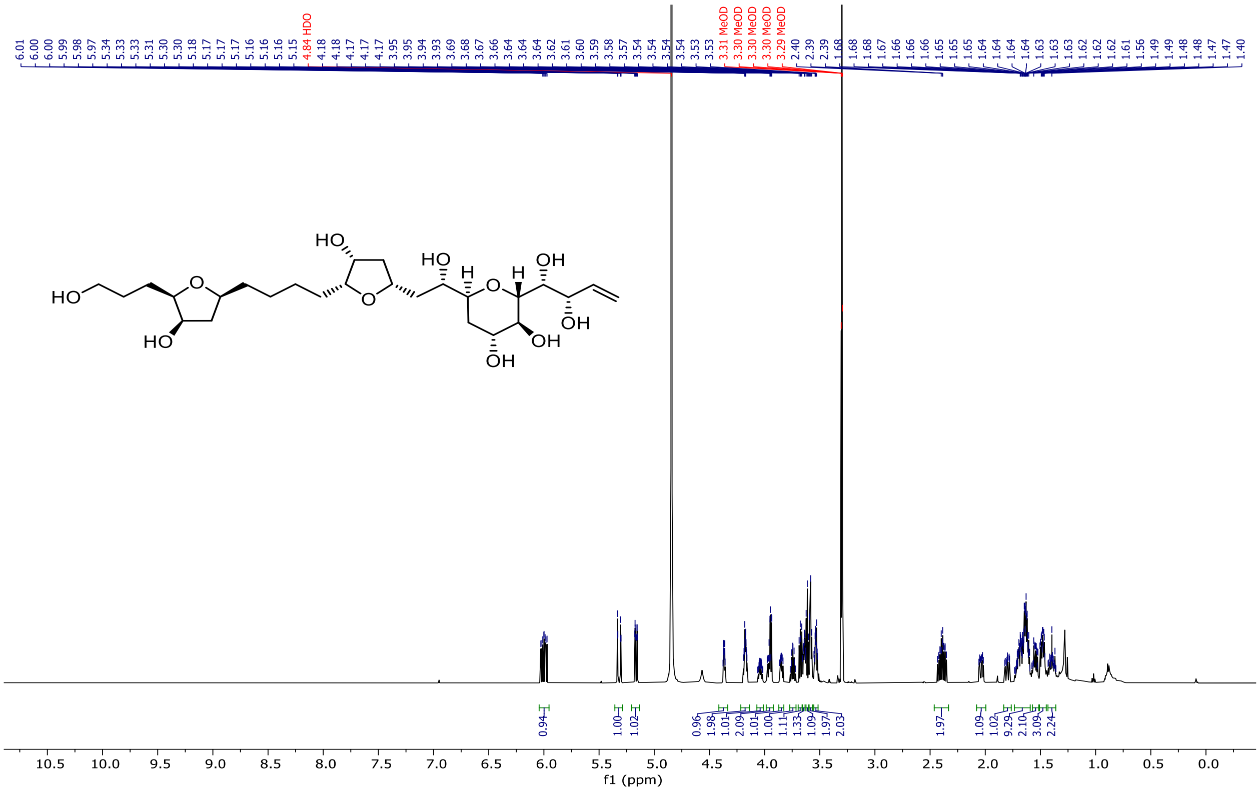

^{13}C NMR (151 MHz, [D$_4$]-MeOH)

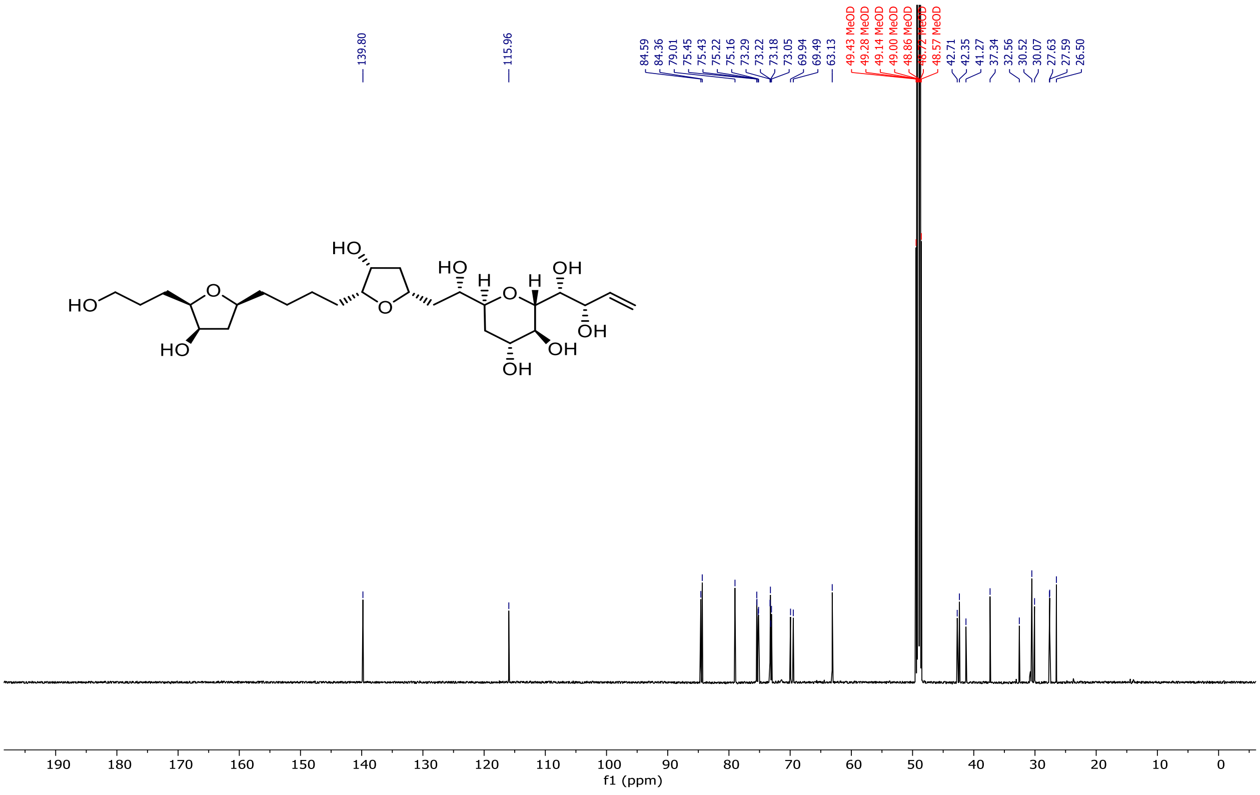

Compound 34*S*-62: ^{1}H-^{1}H COSY ([D$_4$]-MeOH)

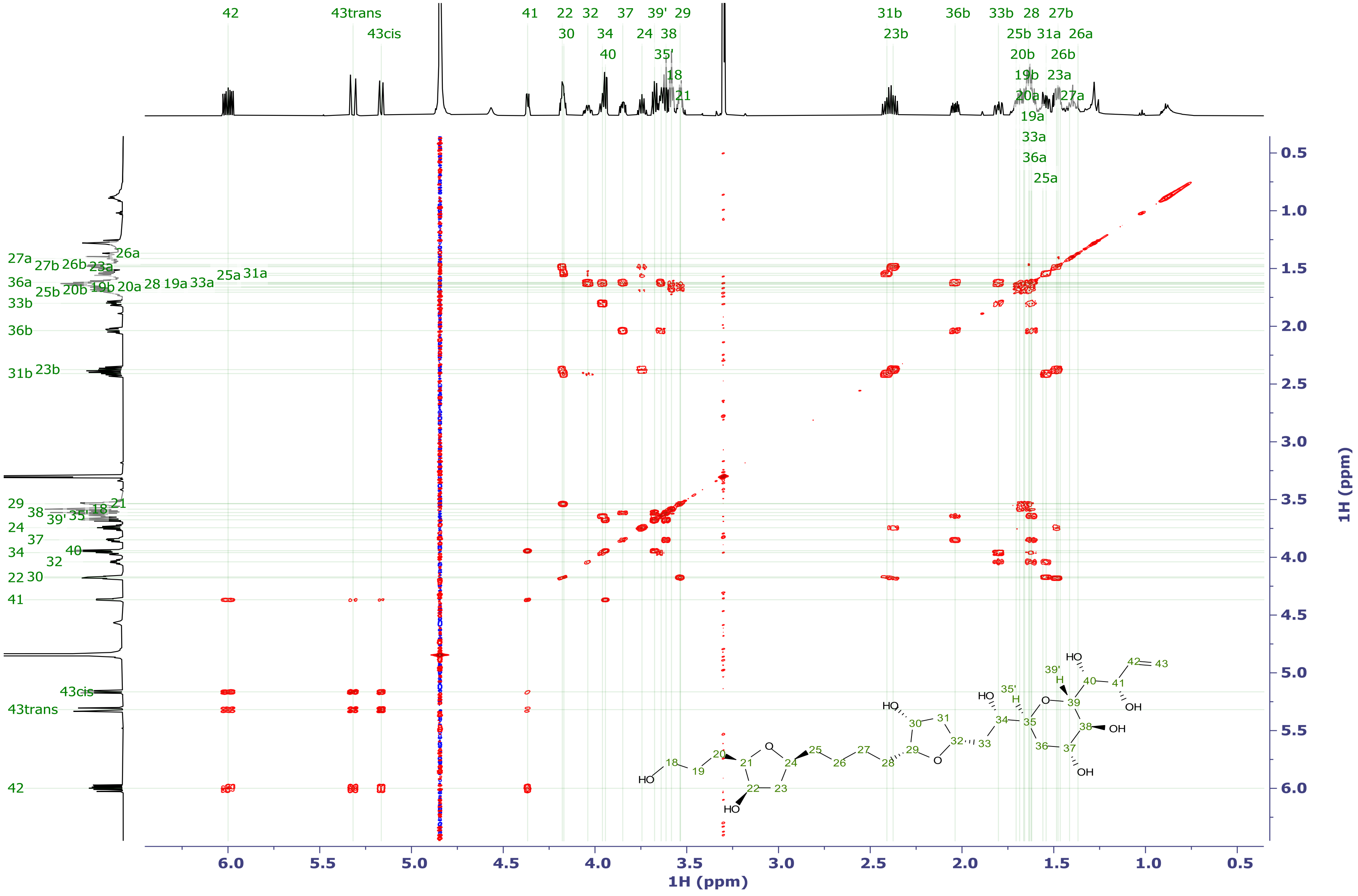

Compound 34*S*-62: HSQC NMR ([D$_4$]-MeOH)

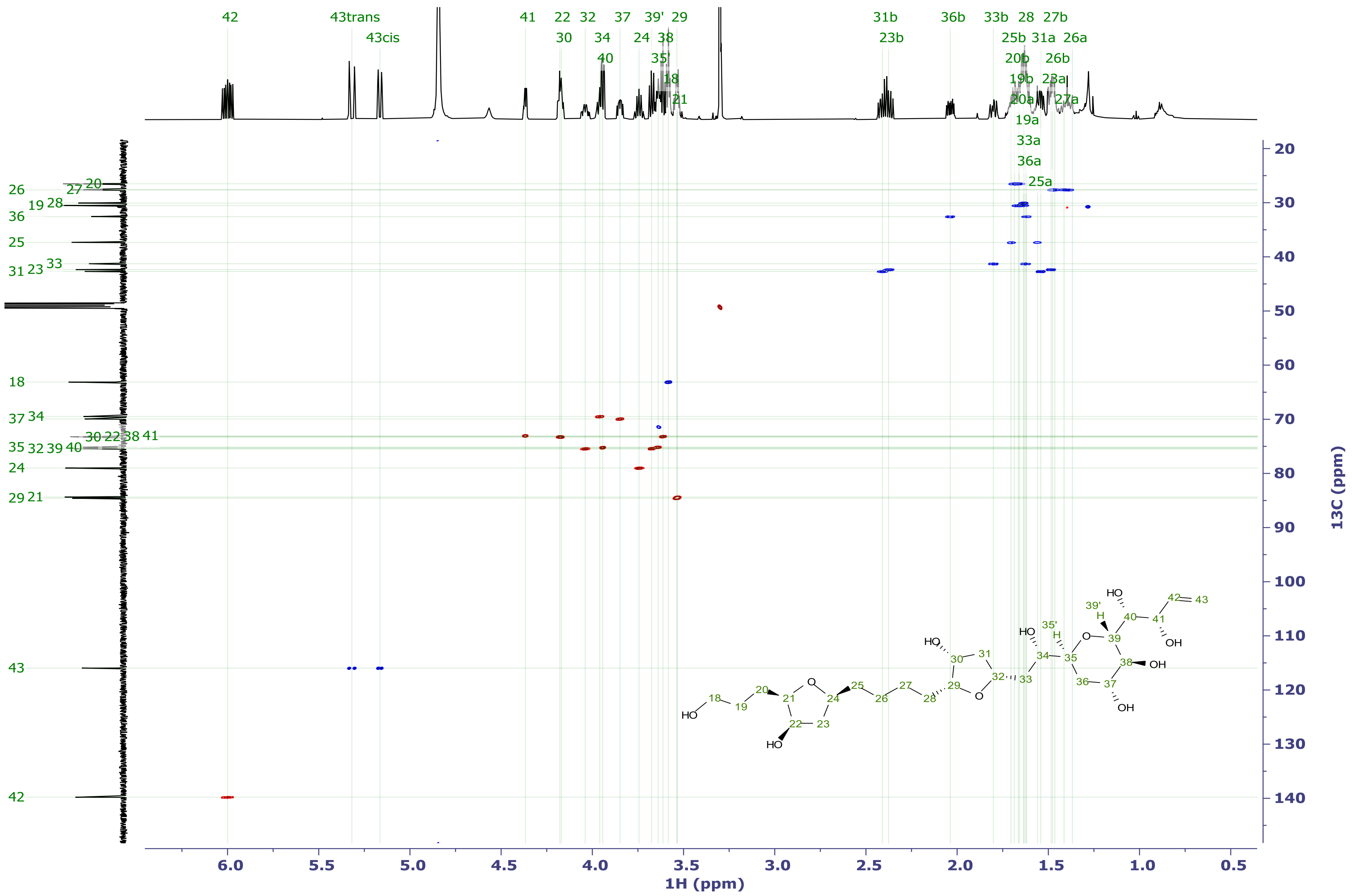

Compound 34*S*-62: HMBC NMR ([D$_4$]-MeOH)

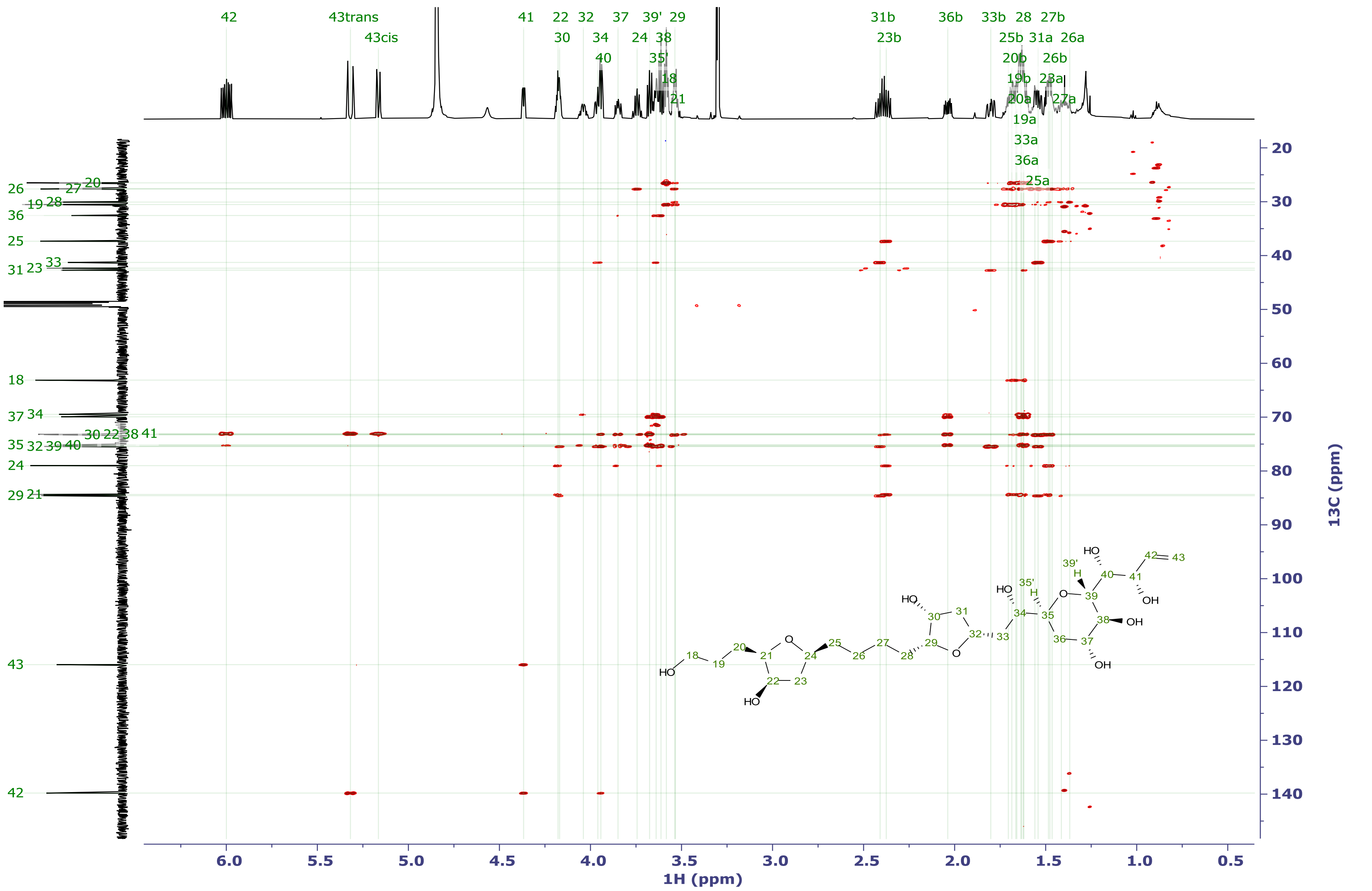

Compound 34*S*-62: NOESY ([D$_4$]-MeOH)

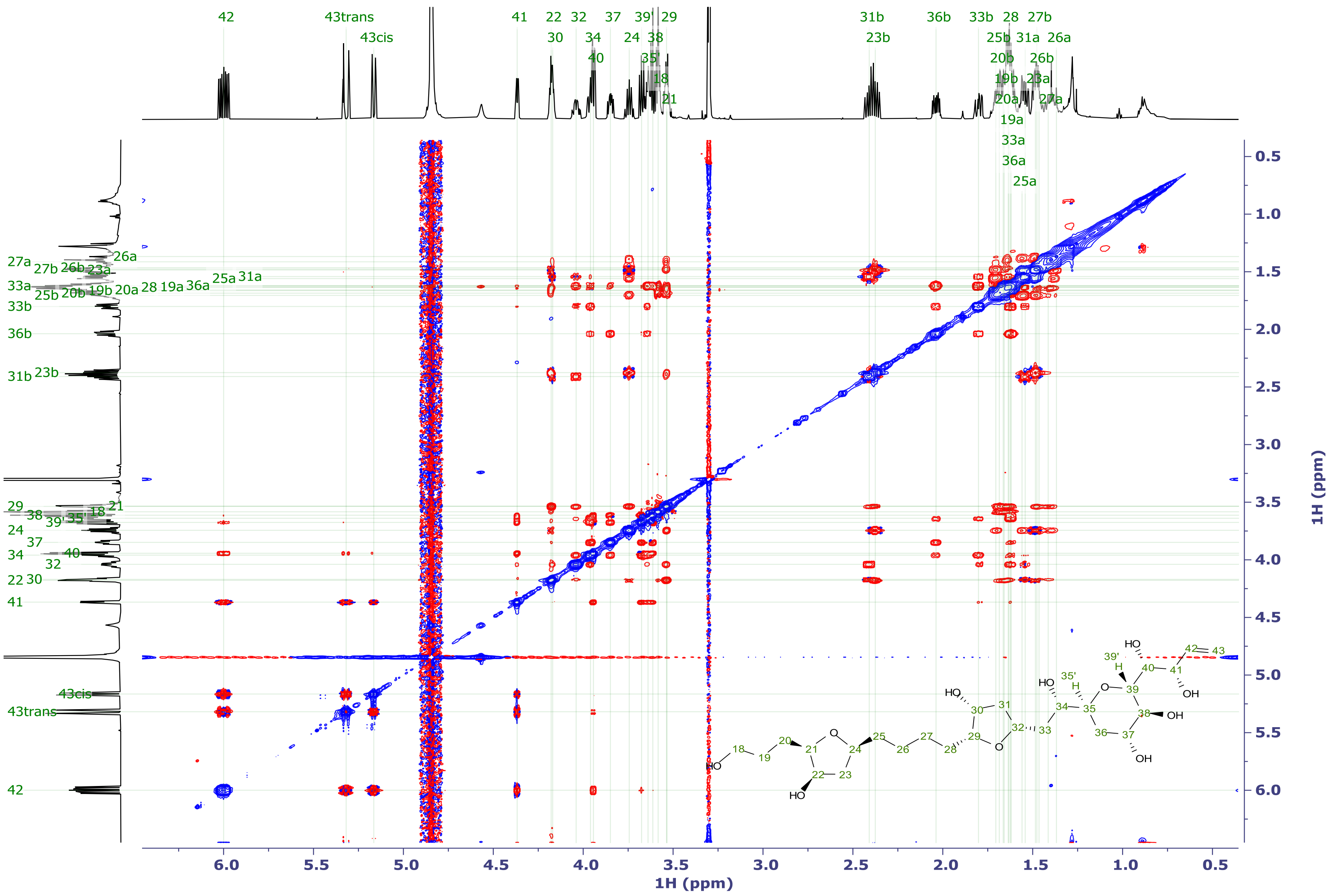

Compound 34*R*-62: ^{1}H NMR (600 MHz, [D$_4$]-MeOH)

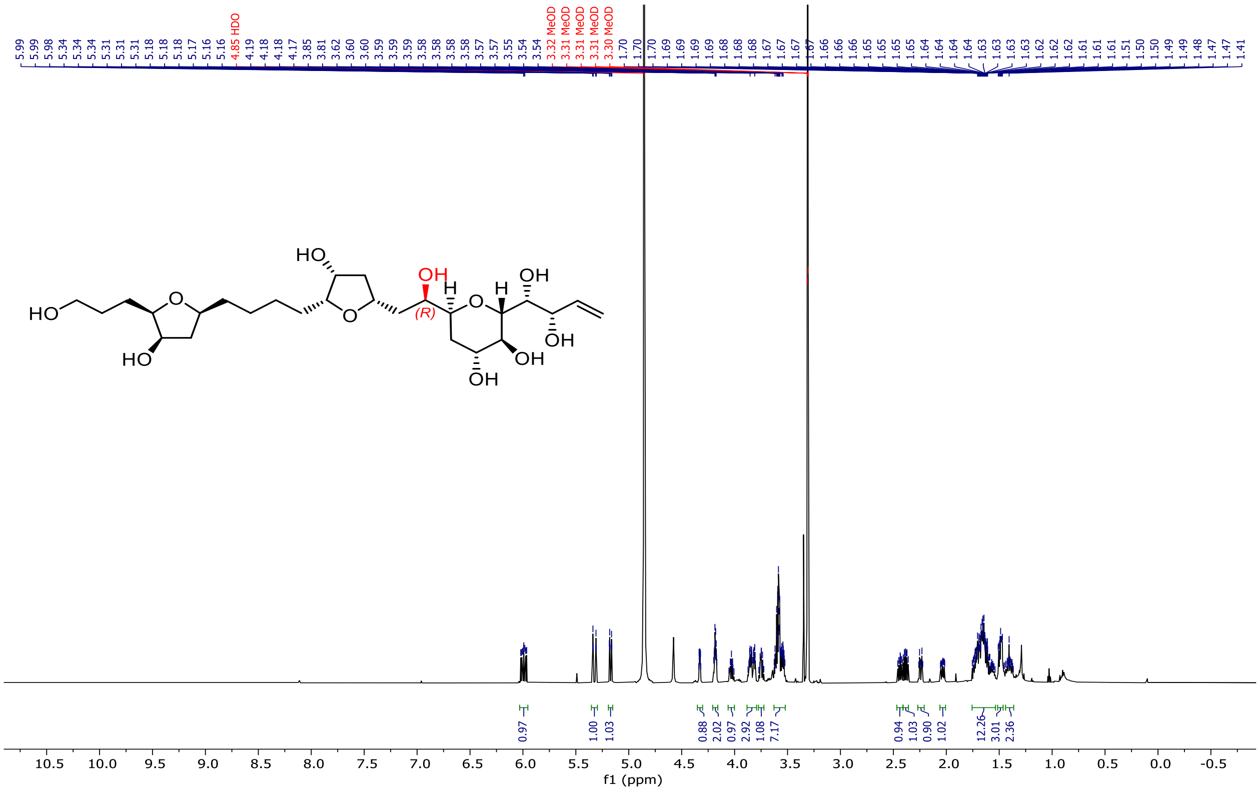

^{13}C NMR (151 MHz, [D$_4$]-MeOH)

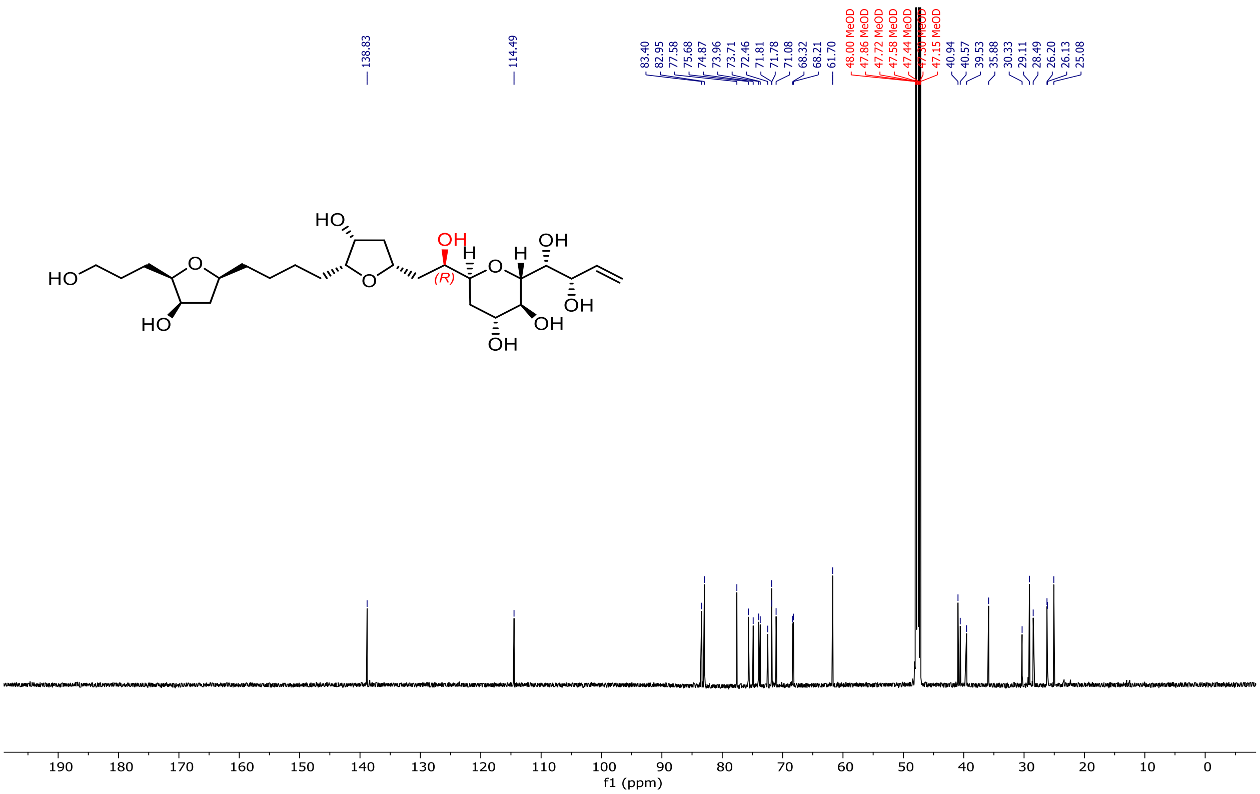

Compound 34*R*-62: ^{1}H-^{1}H COSY ([D$_4$]-MeOH)

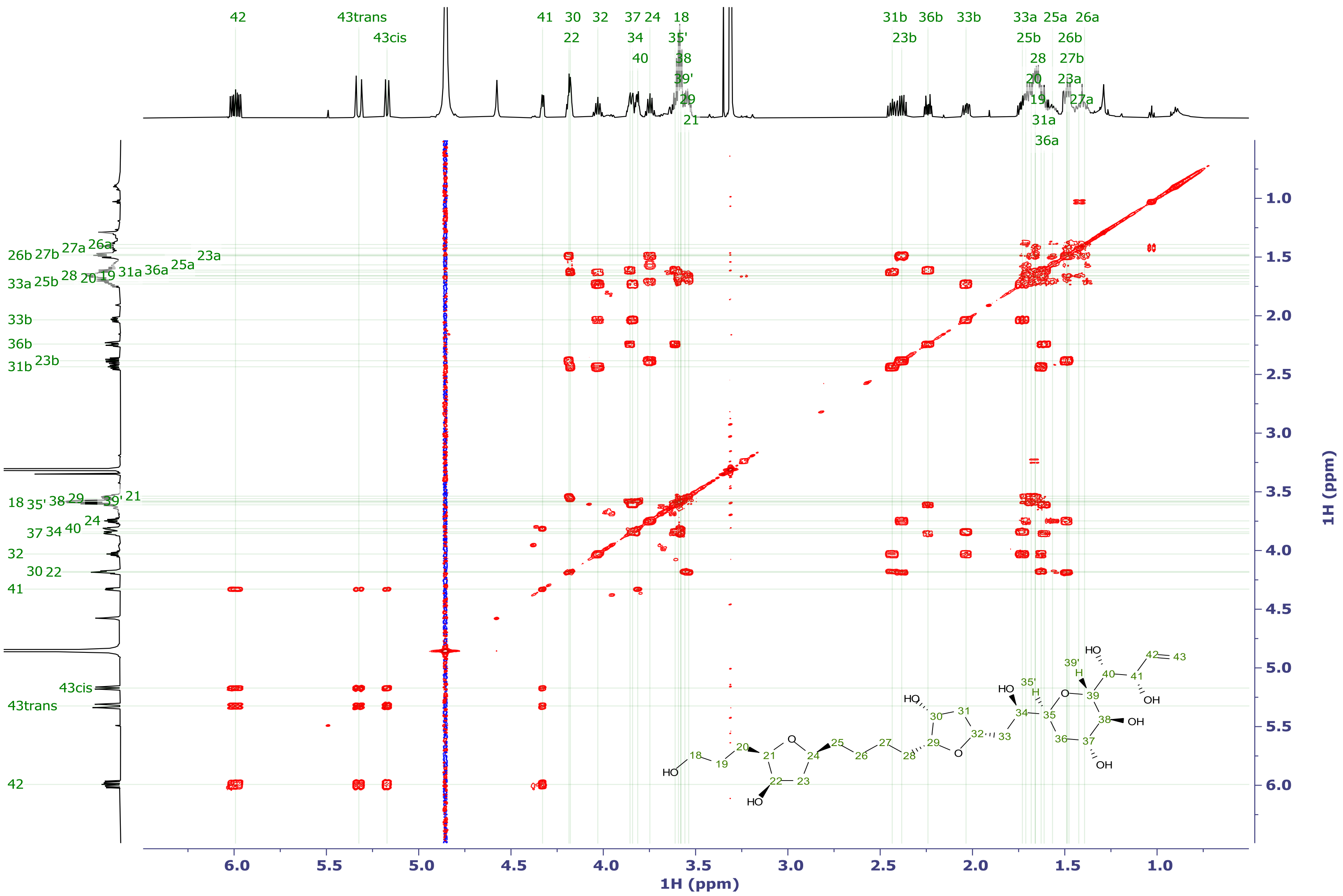

Compound 34*R*-62: HSQC NMR ([D4]-MeOH)

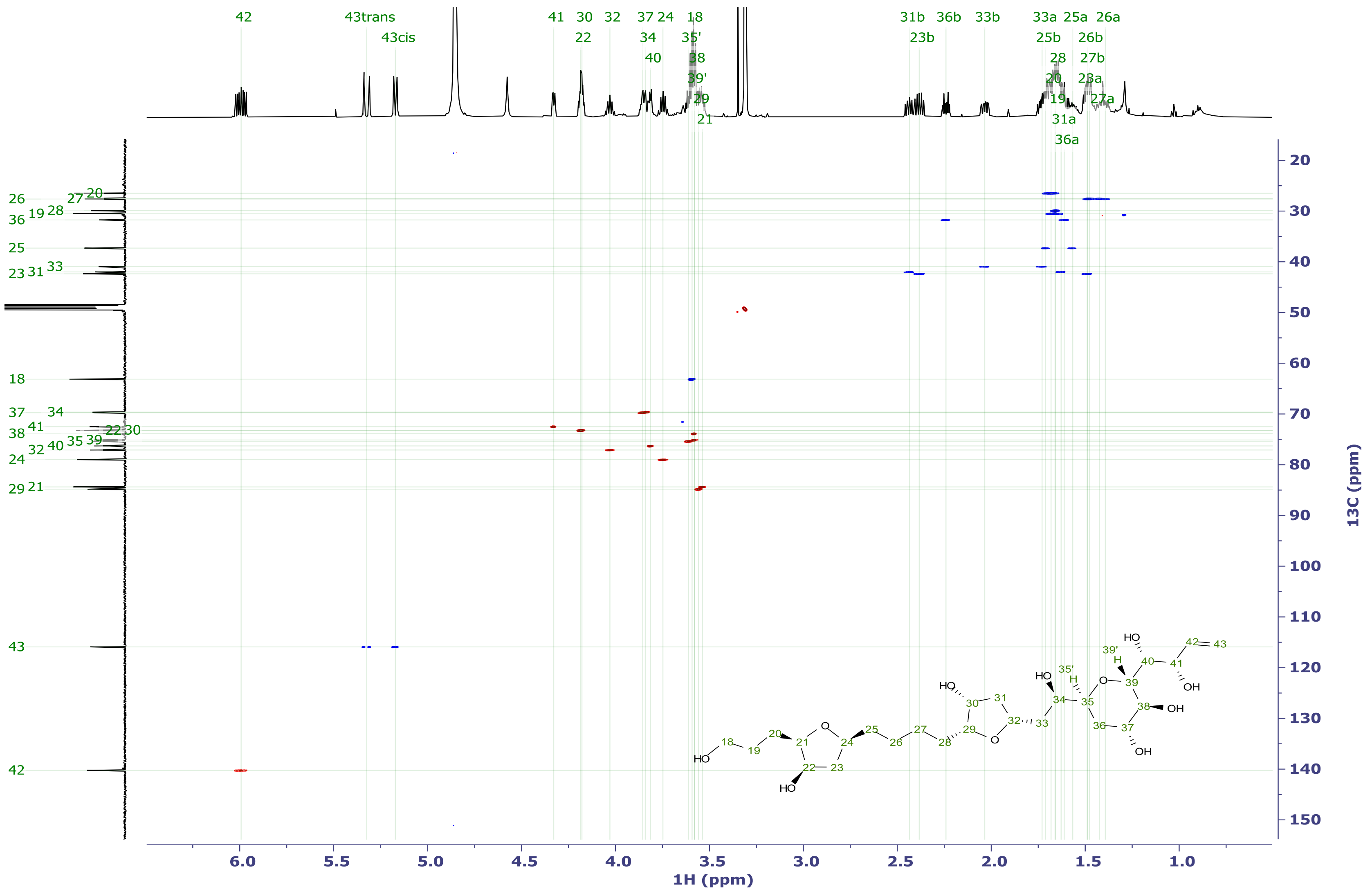

Compound 34*R*-62: HMBC NMR ([D$_4$]-MeOH)

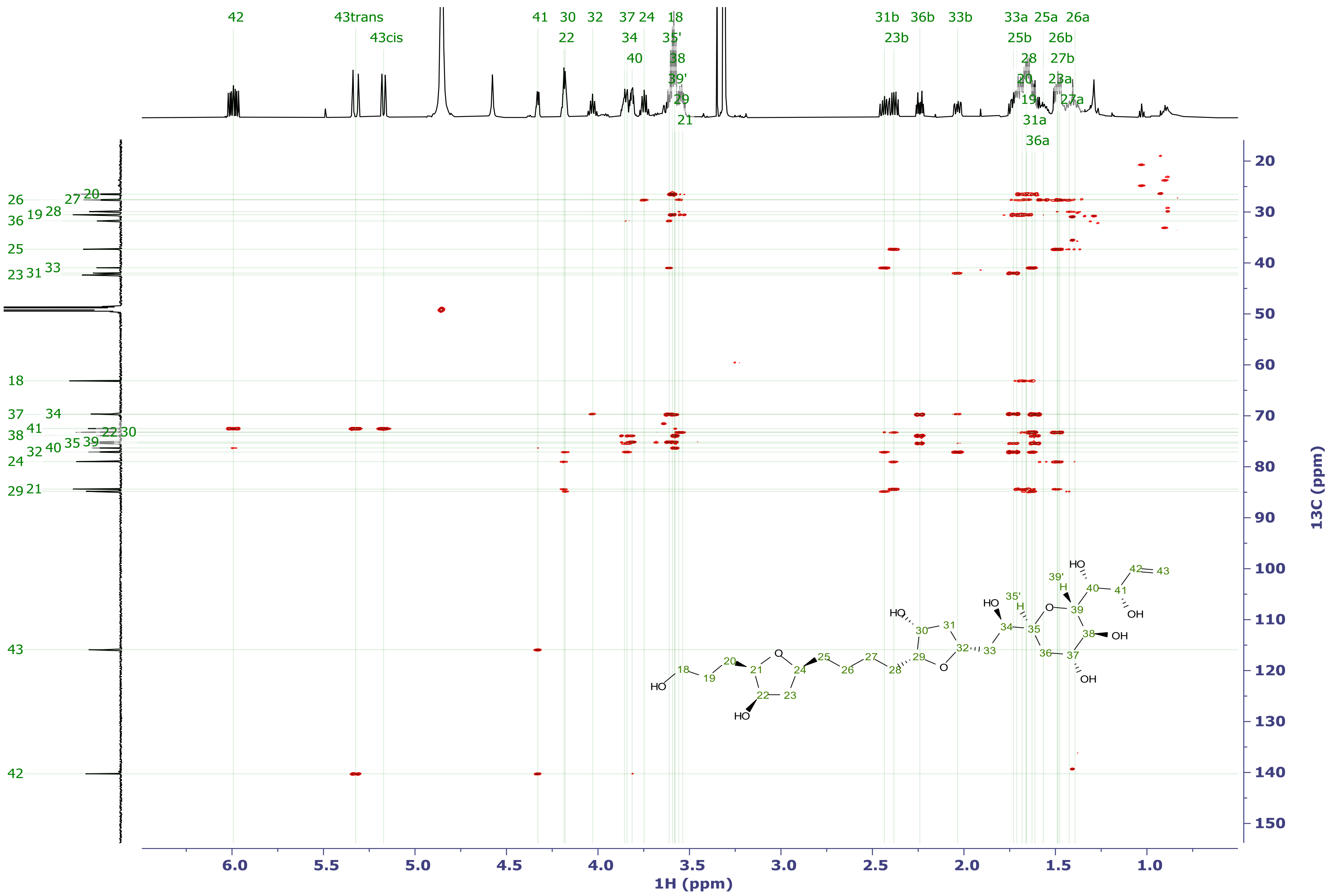

Compound 34*R*-62: NOESY ([D$_4$]-MeOH)

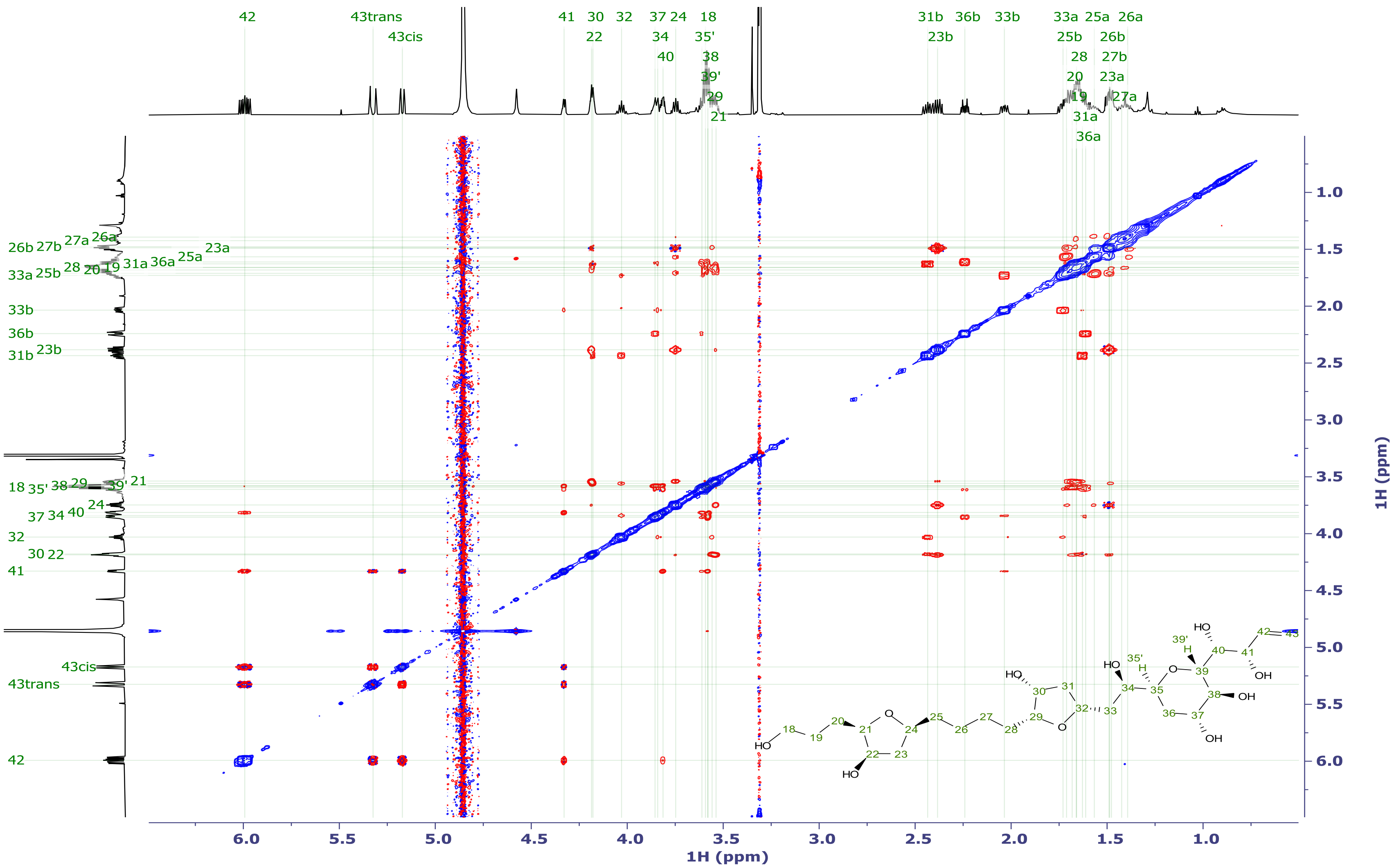

Compound 63: ^{1}H NMR (400 MHz, $CDCl_3$)

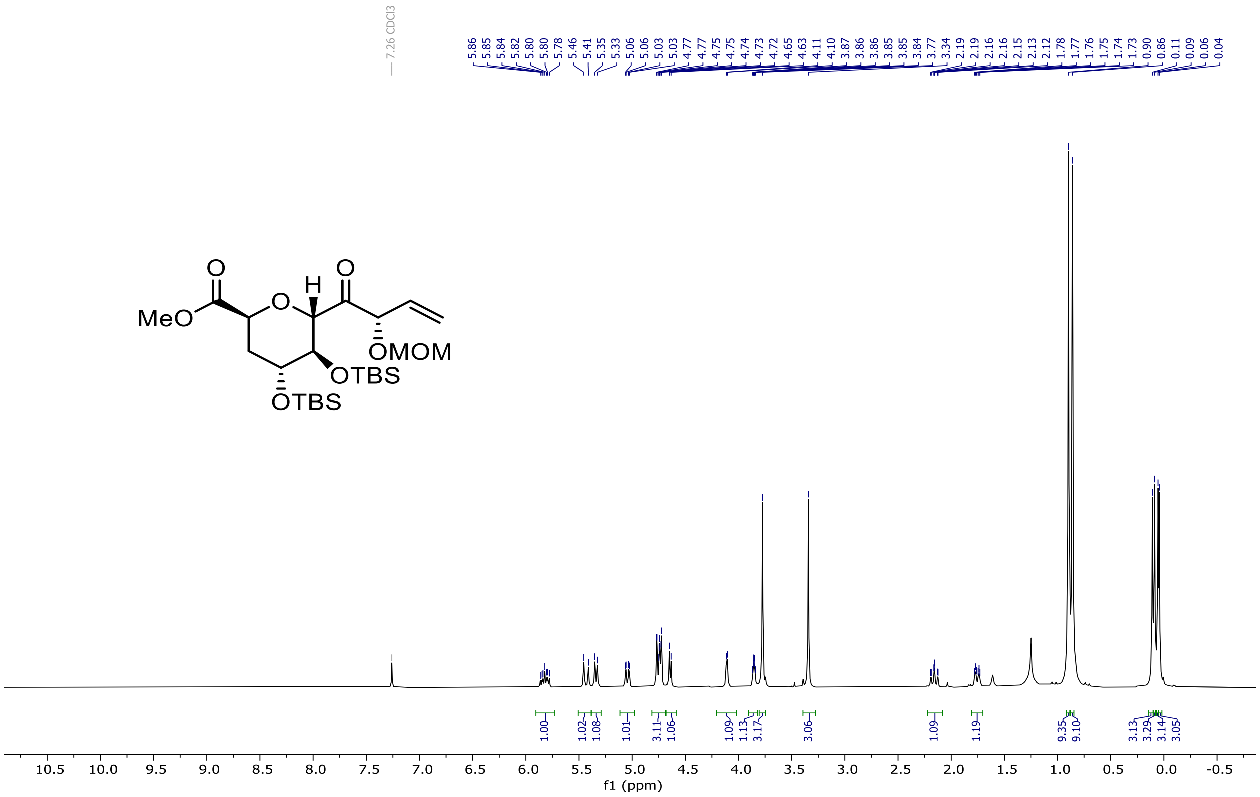

^{13}C NMR (101 MHz, $CDCl_3$)

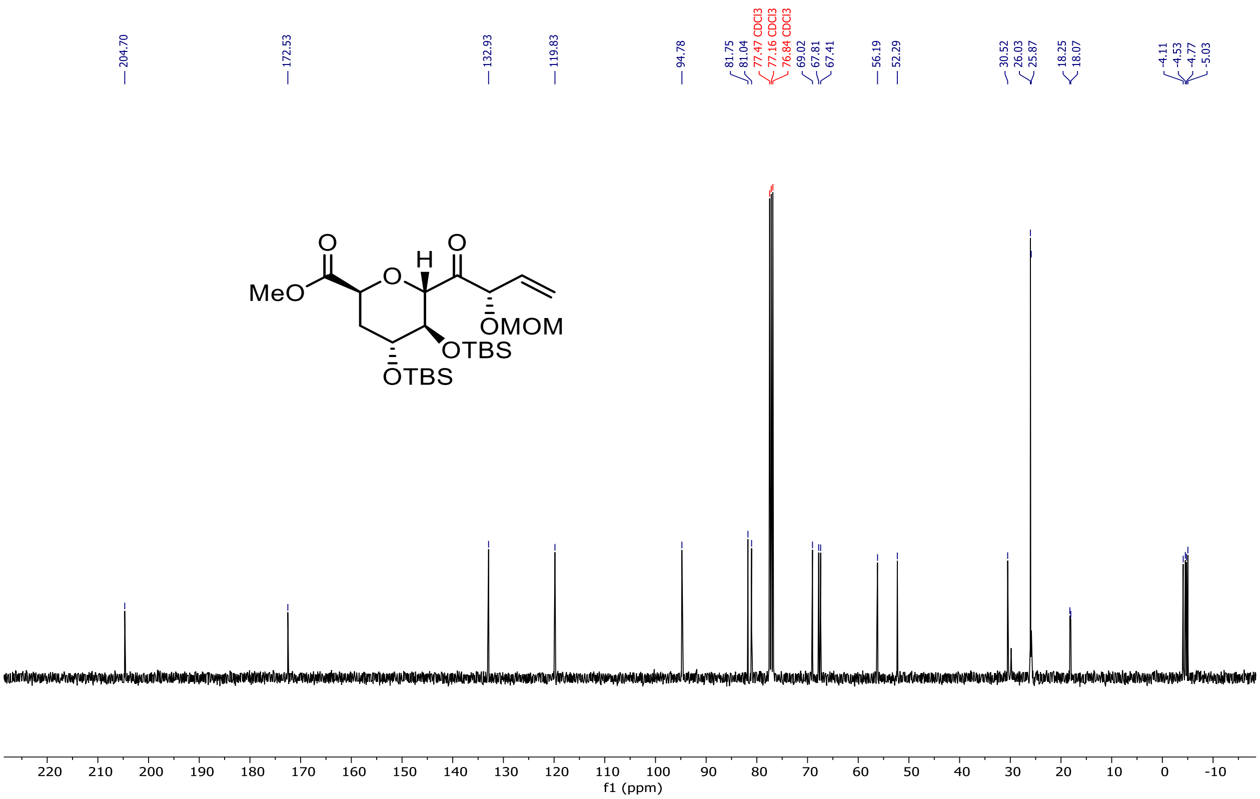

Compound 64: ^{1}H NMR (400 MHz, $CDCl_3$)

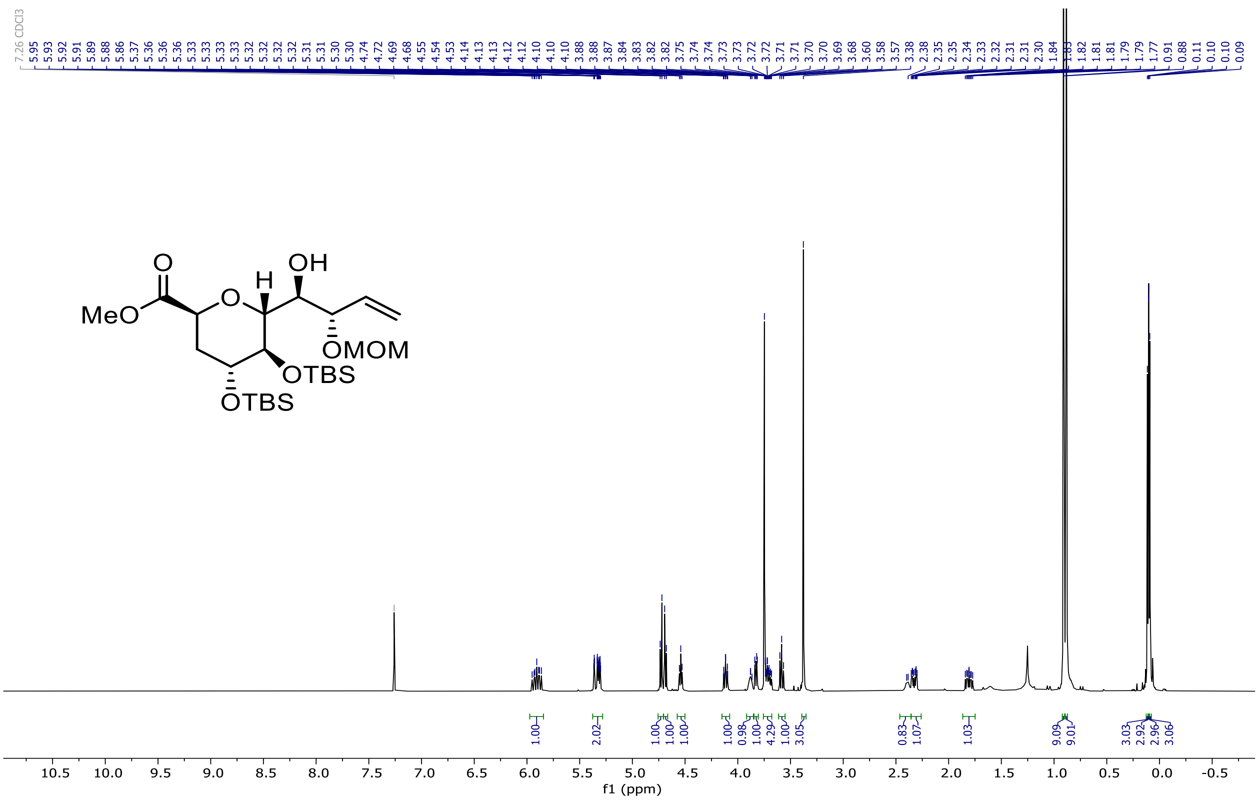

^{13}C NMR (101 MHz, $CDCl_3$)

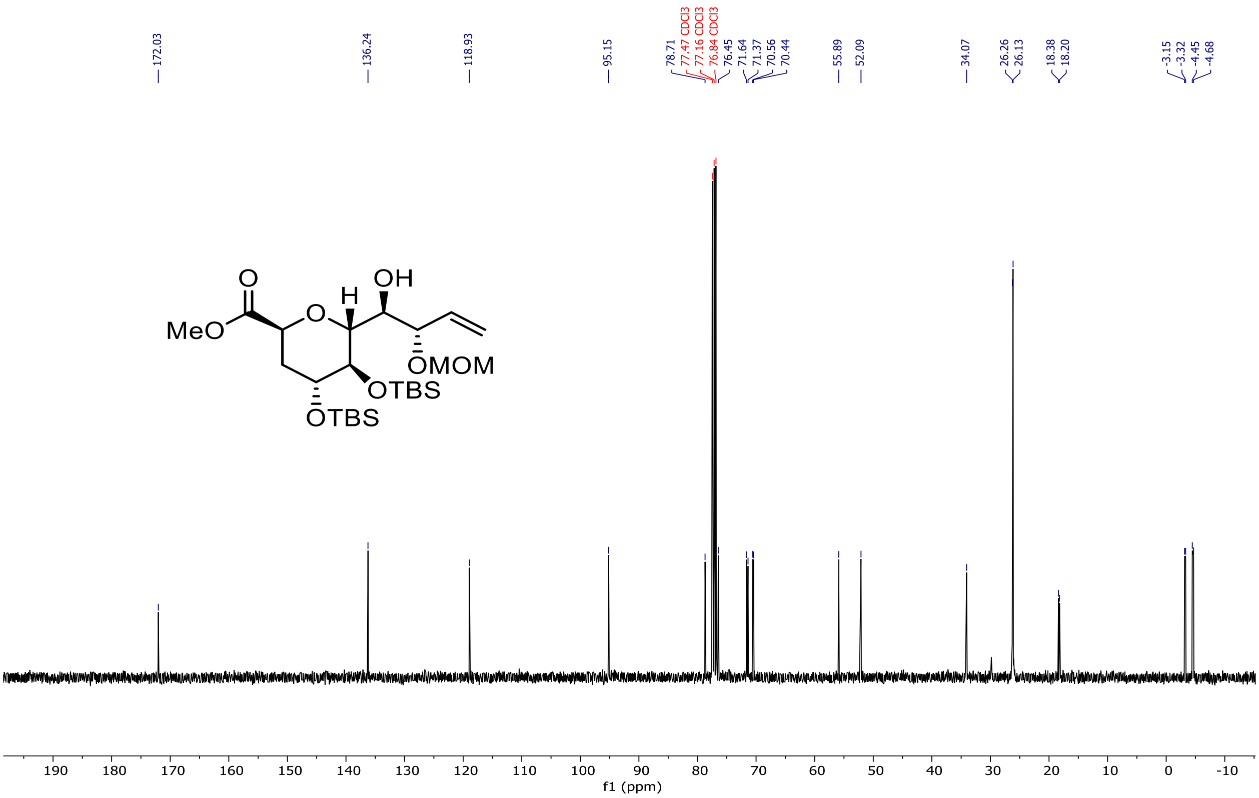

Compound S21: ^{1}H NMR (400 MHz, $CDCl_3$)

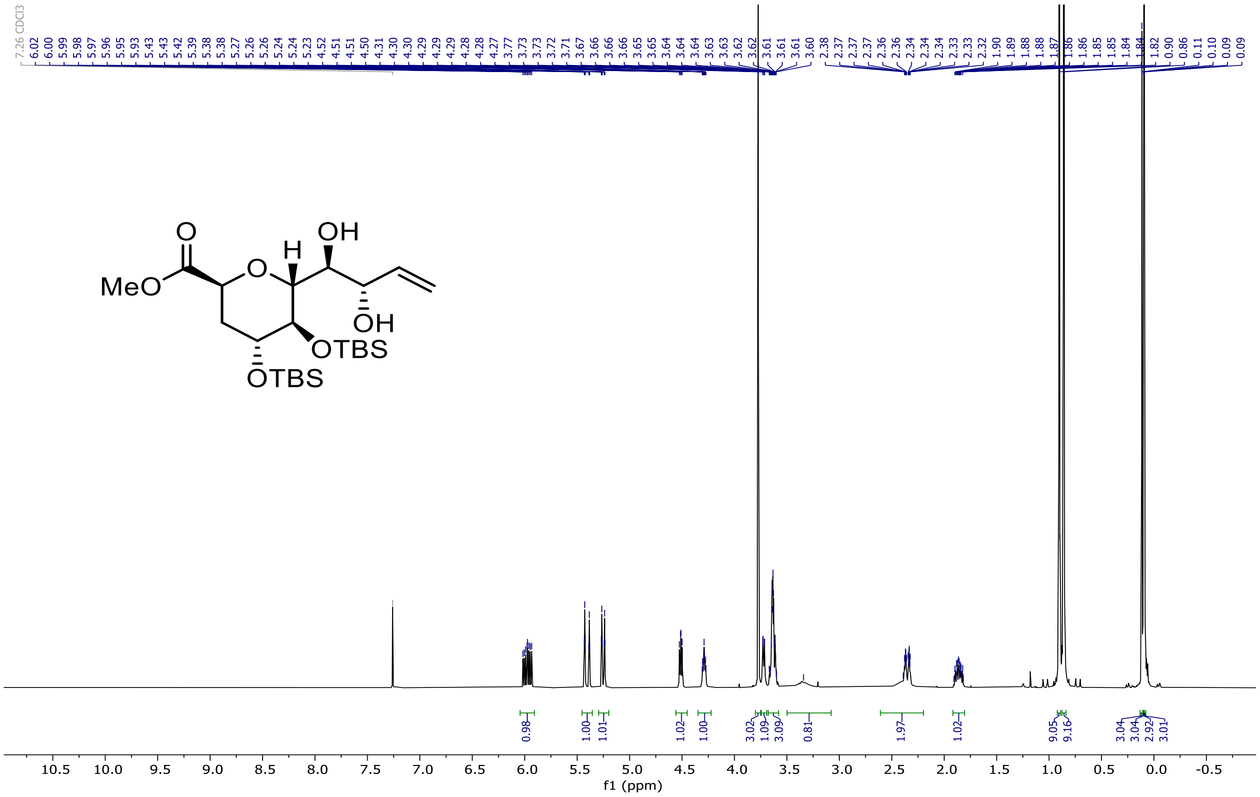

^{13}C NMR (101 MHz, $CDCl_3$)

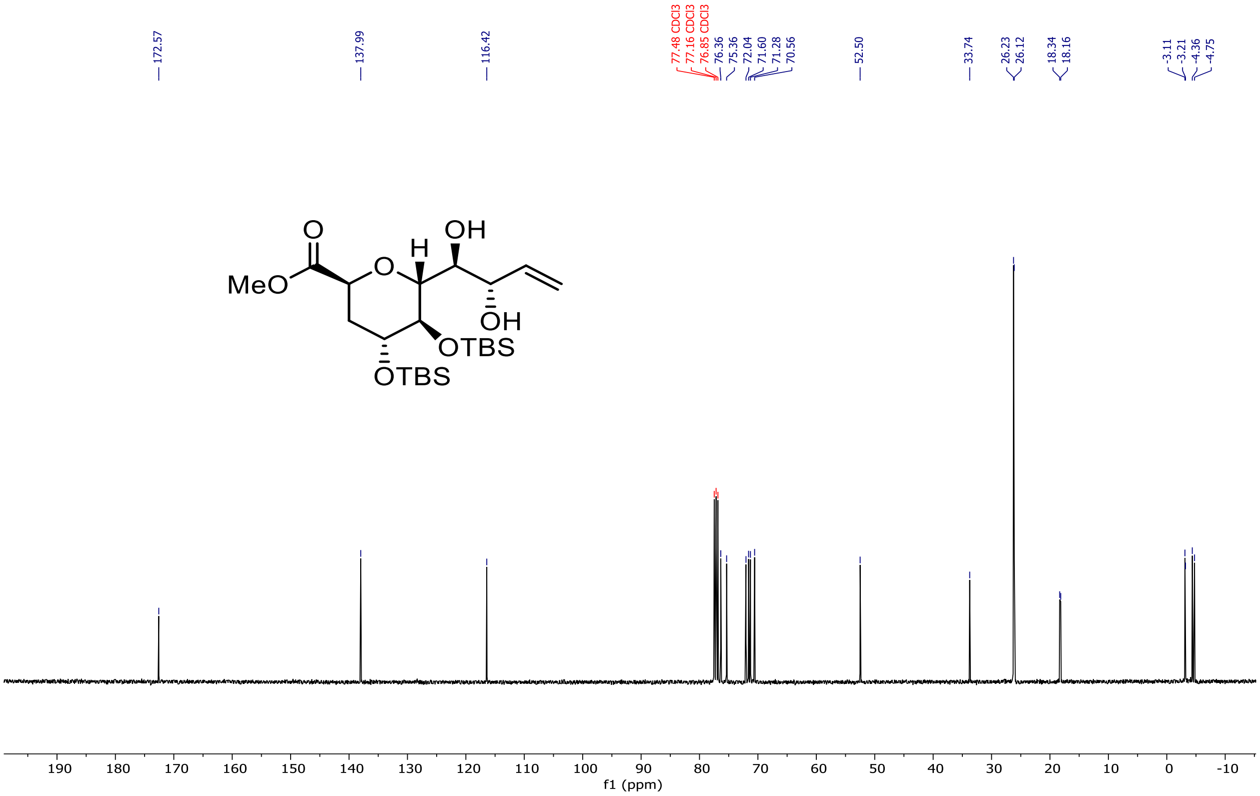

Compound 65: ^{1}H NMR (600 MHz, [D$_4$]-MeOH)

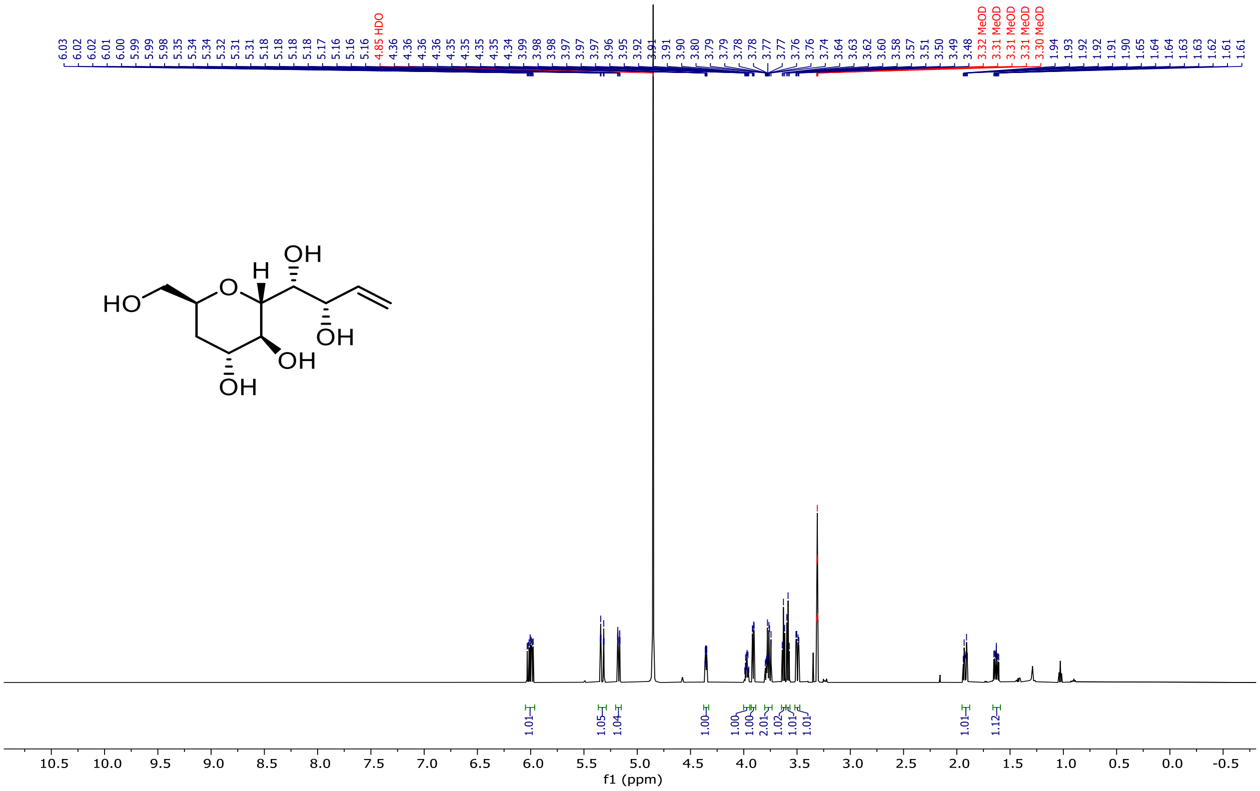

^{13}C NMR (151 MHz, [D$_4$]-MeOH)

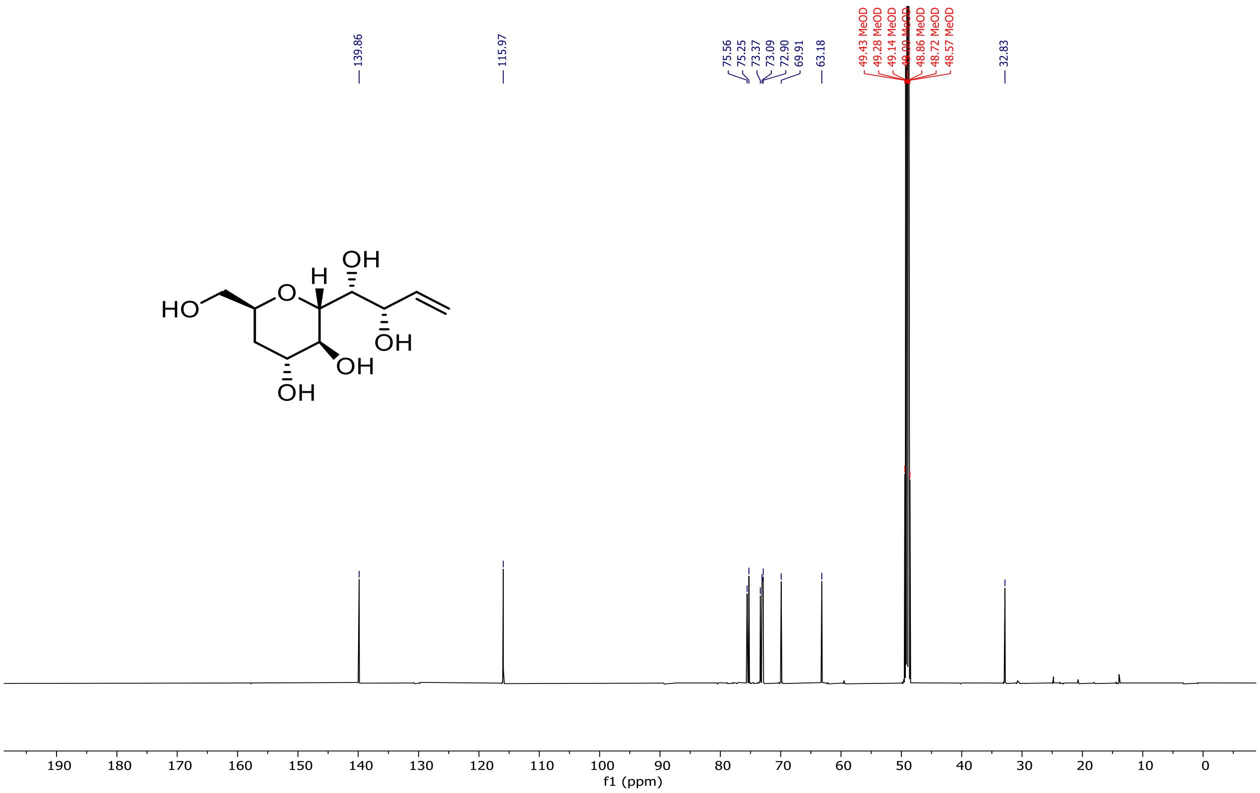

Compound 65: ^{1}H-^{1}H COSY ([D$_4$]-MeOH)

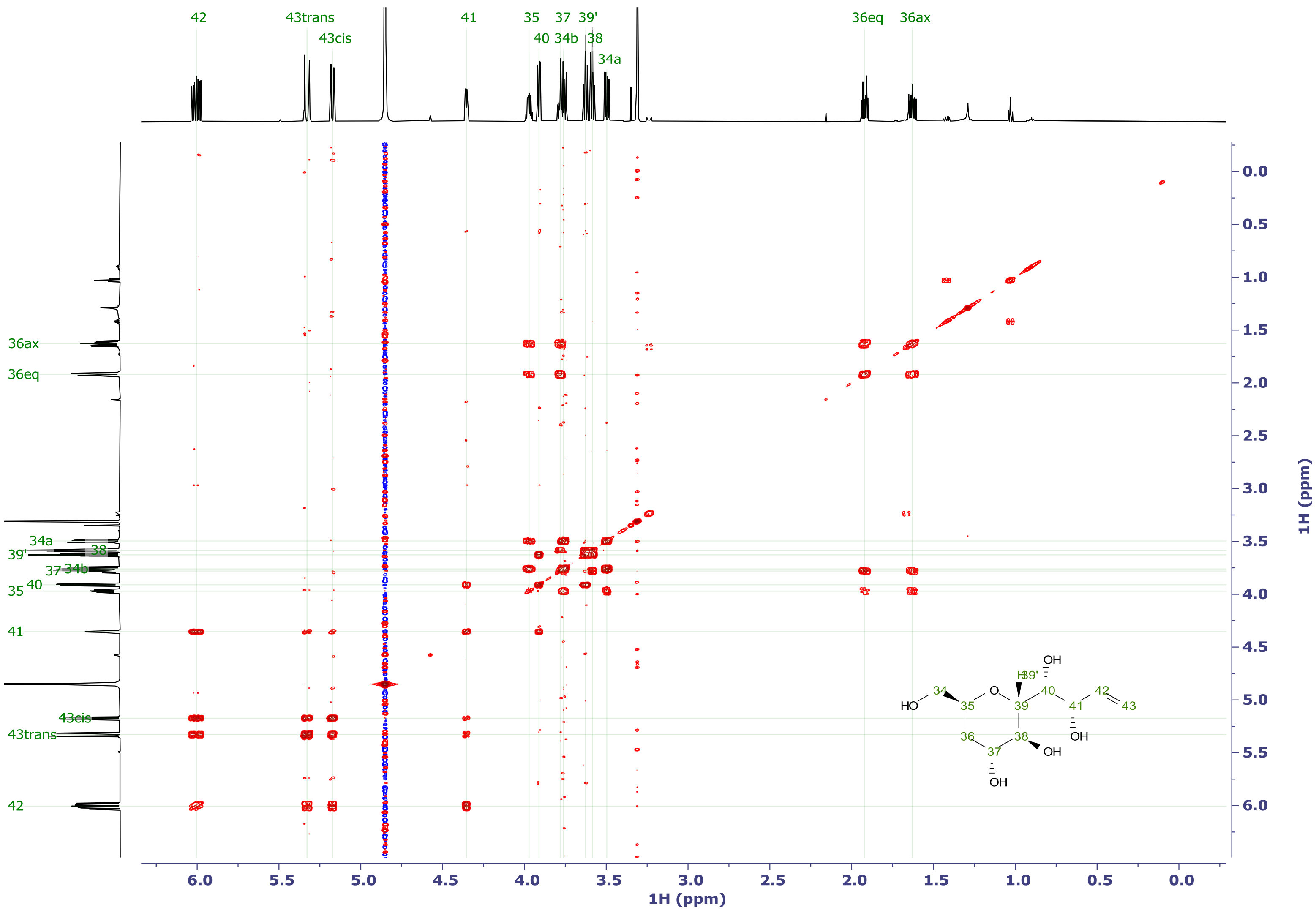

Compound 65: HSQC NMR ([D_4]-MeOH)

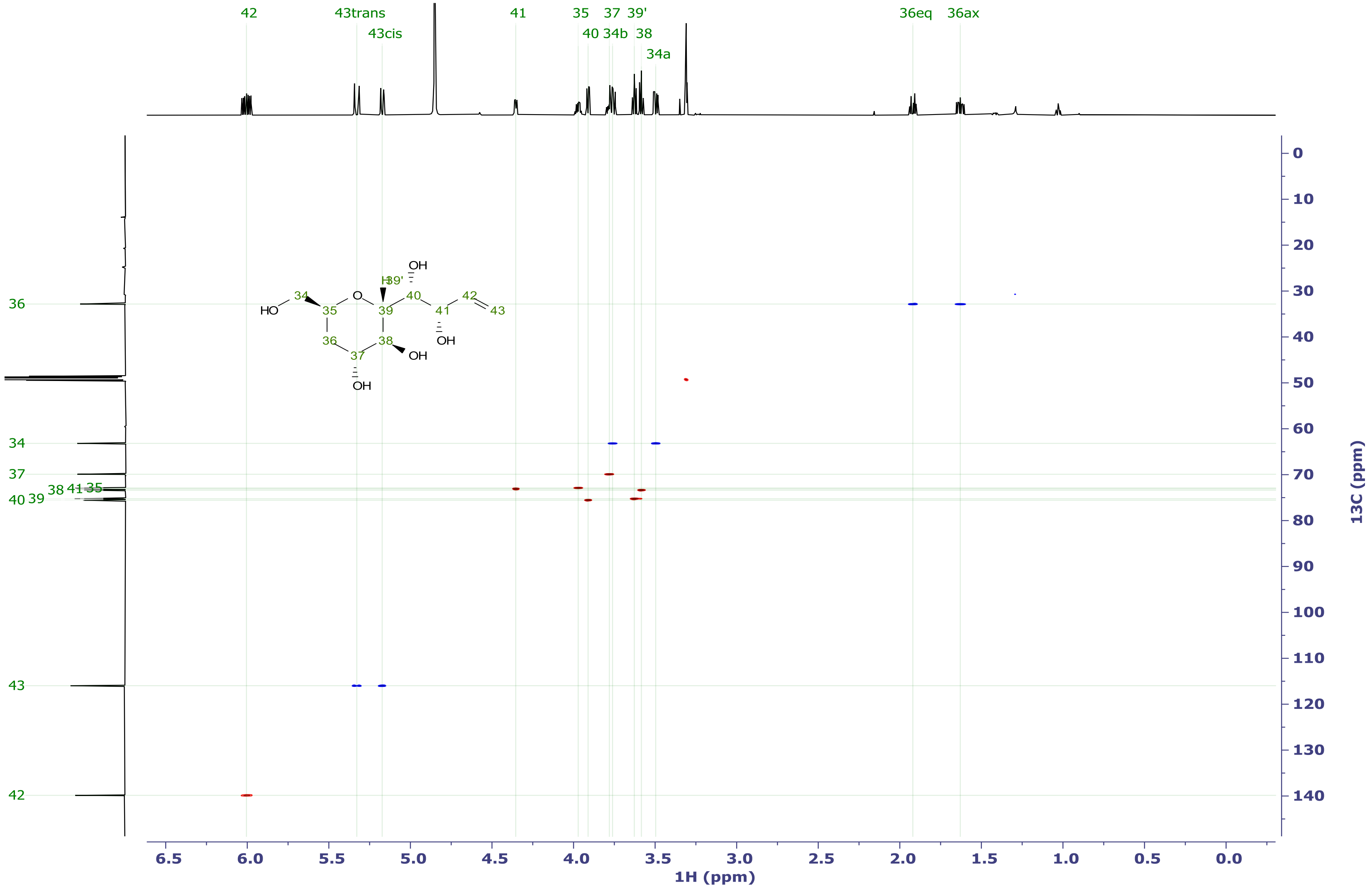

Compound 65: HMBC NMR ([D$_4$]-MeOH)

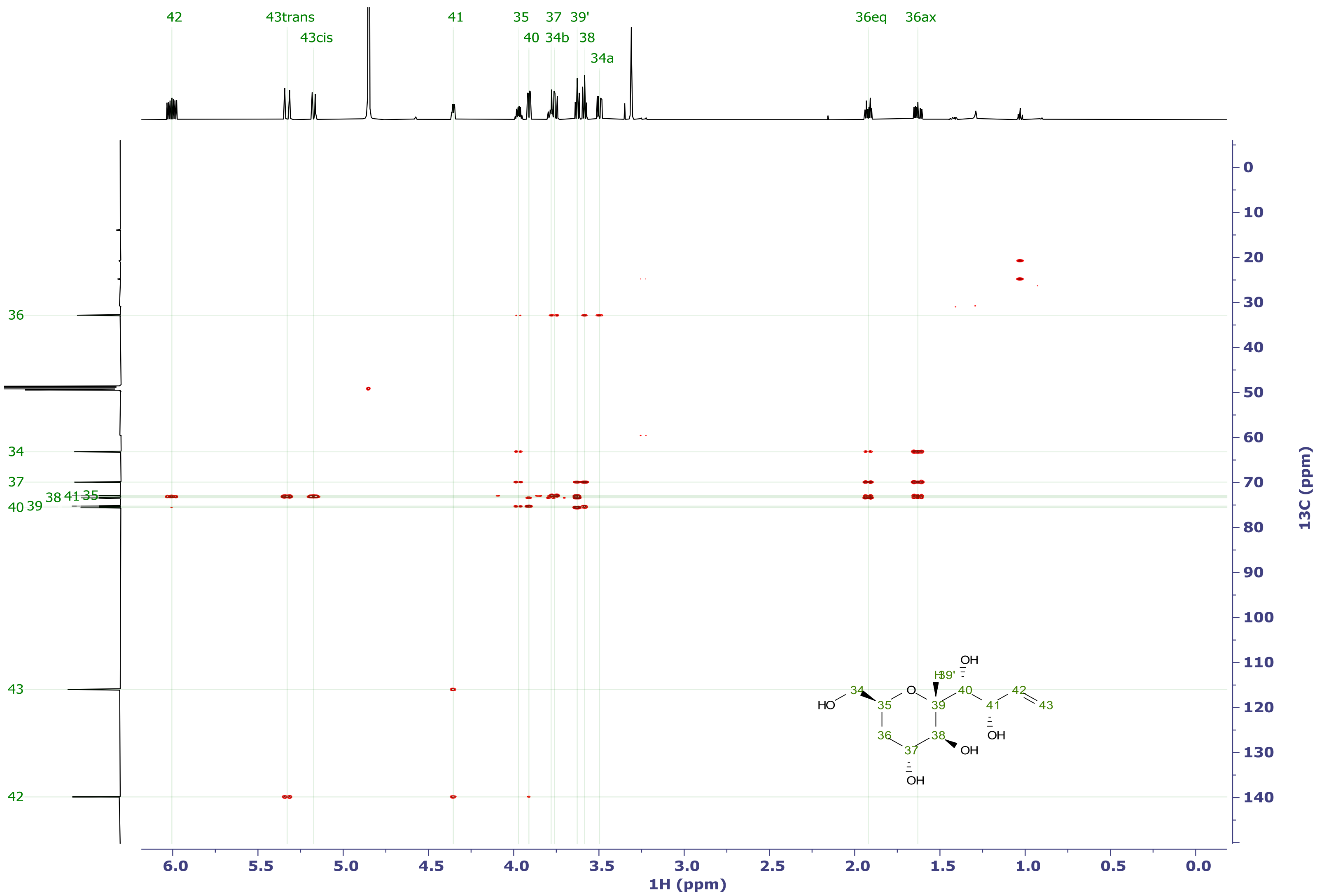

Compound 65: NOESY ([D$_4$]-MeOH)

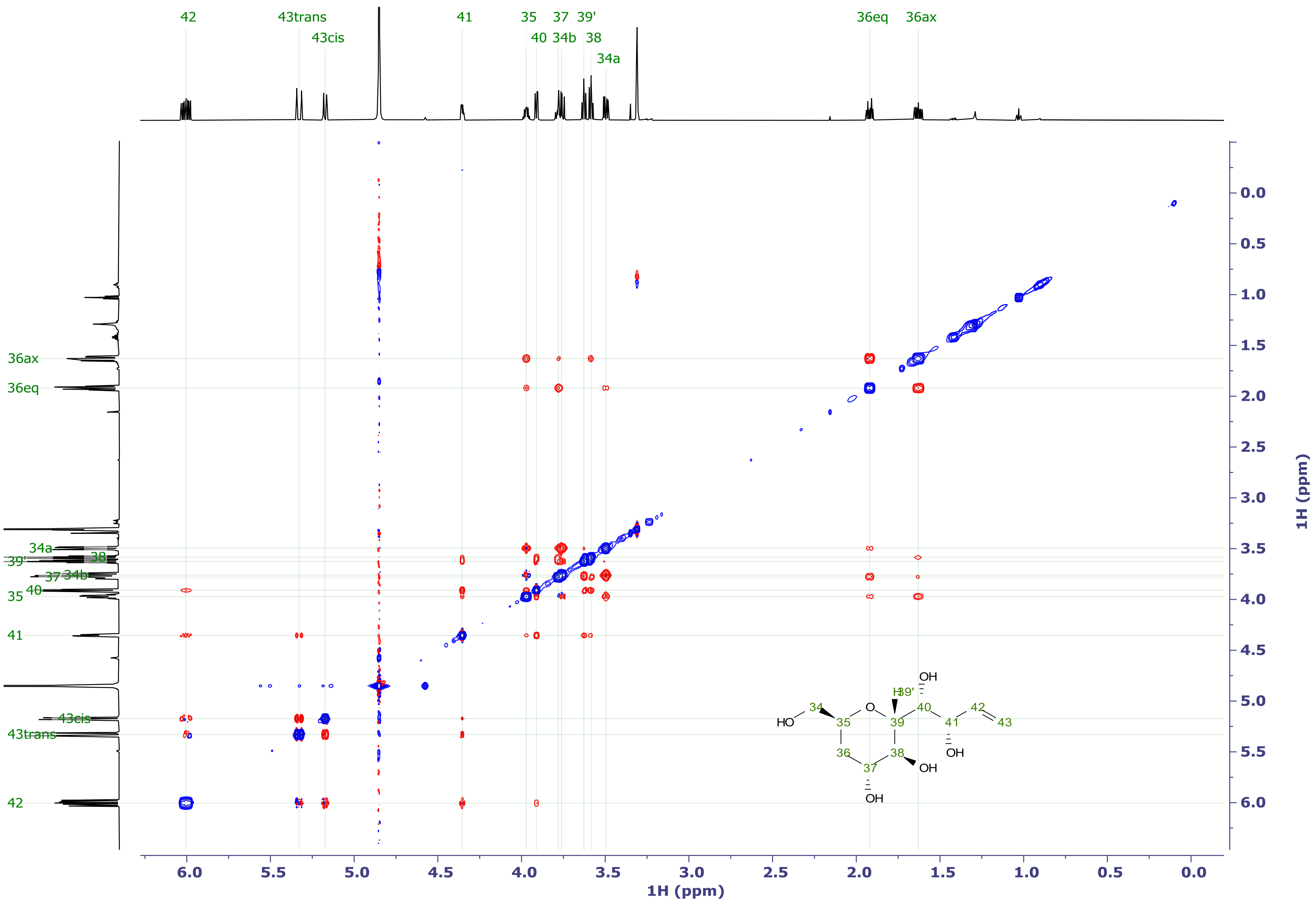

Compound 66: ^{1}H NMR (600 MHz, [D$_4$]-MeOH)

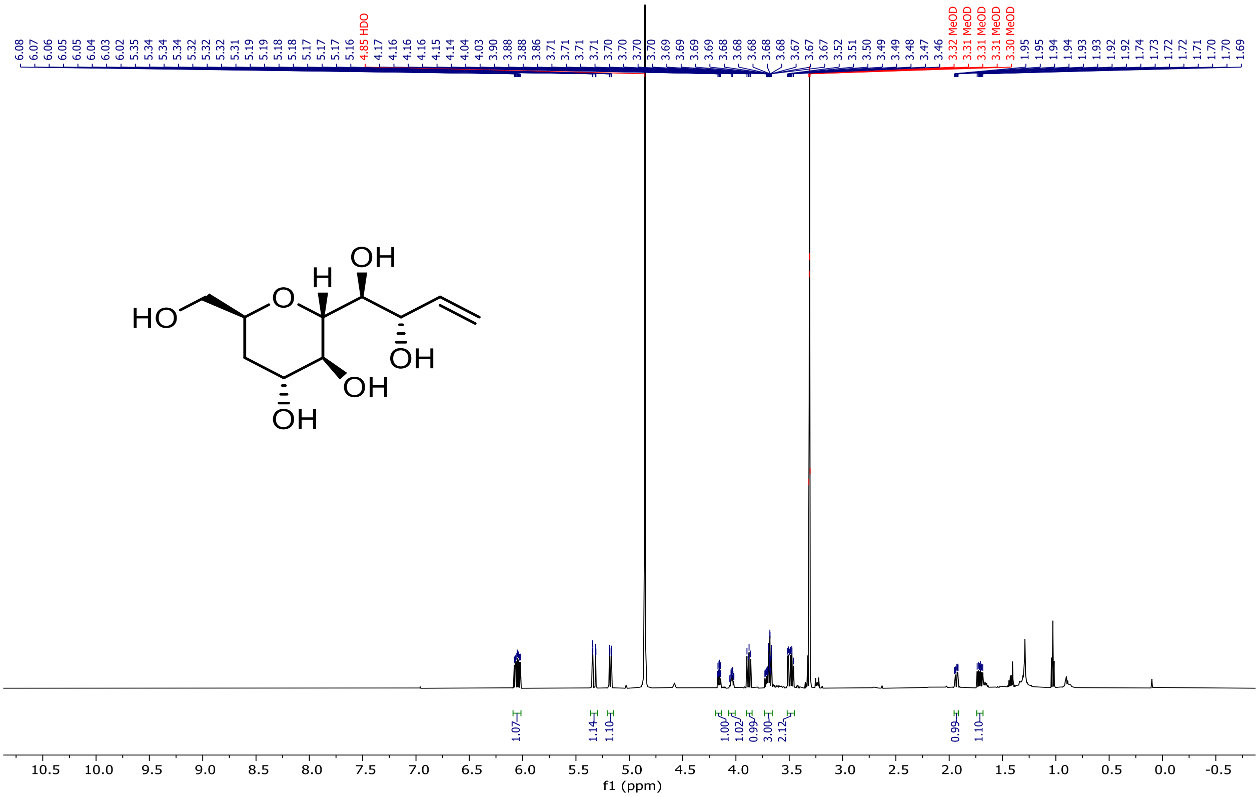

^{13}C NMR (151 MHz, [D$_4$]-MeOH)

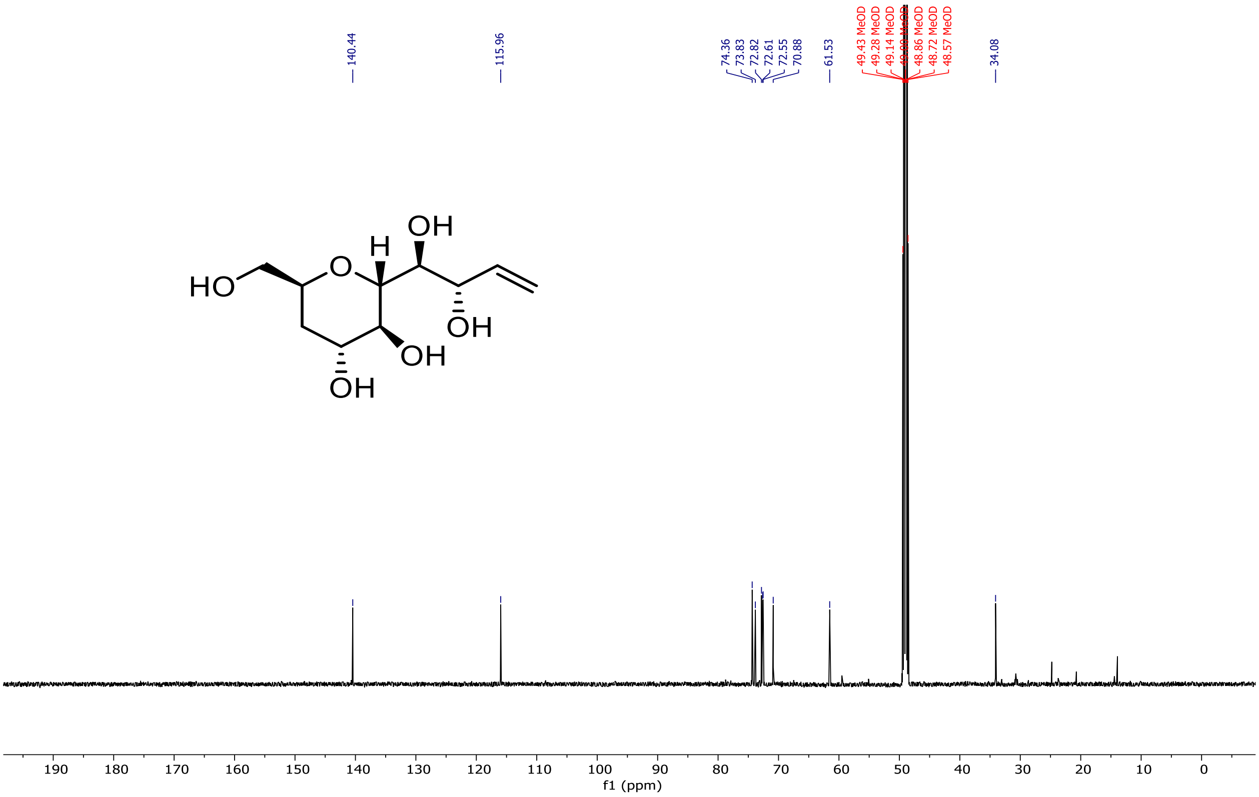

Compound 66: ^{1}H-^{1}H COSY ([D$_4$]-MeOH)

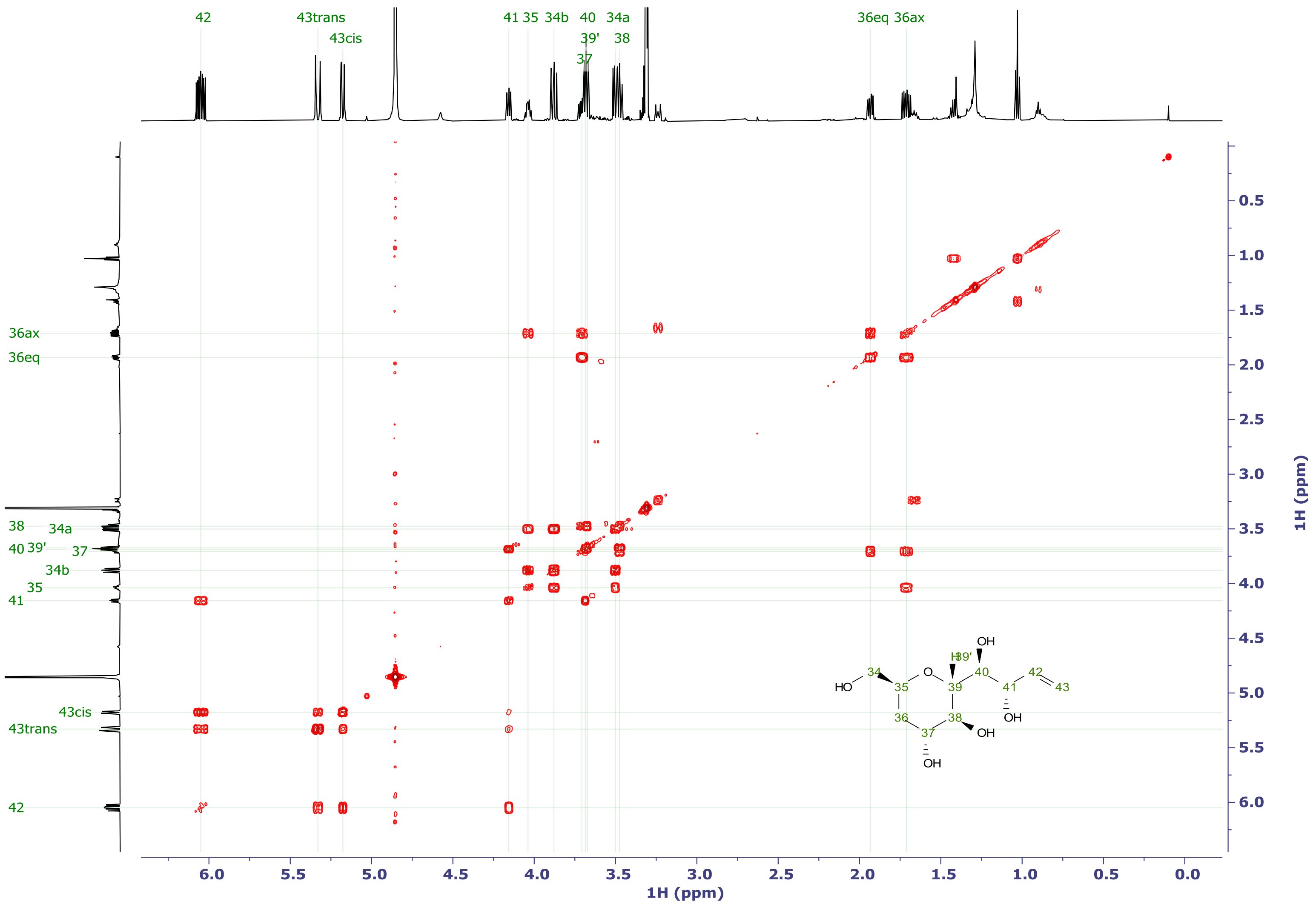

Compound 66: HSQC NMR ([D_4]-MeOH)

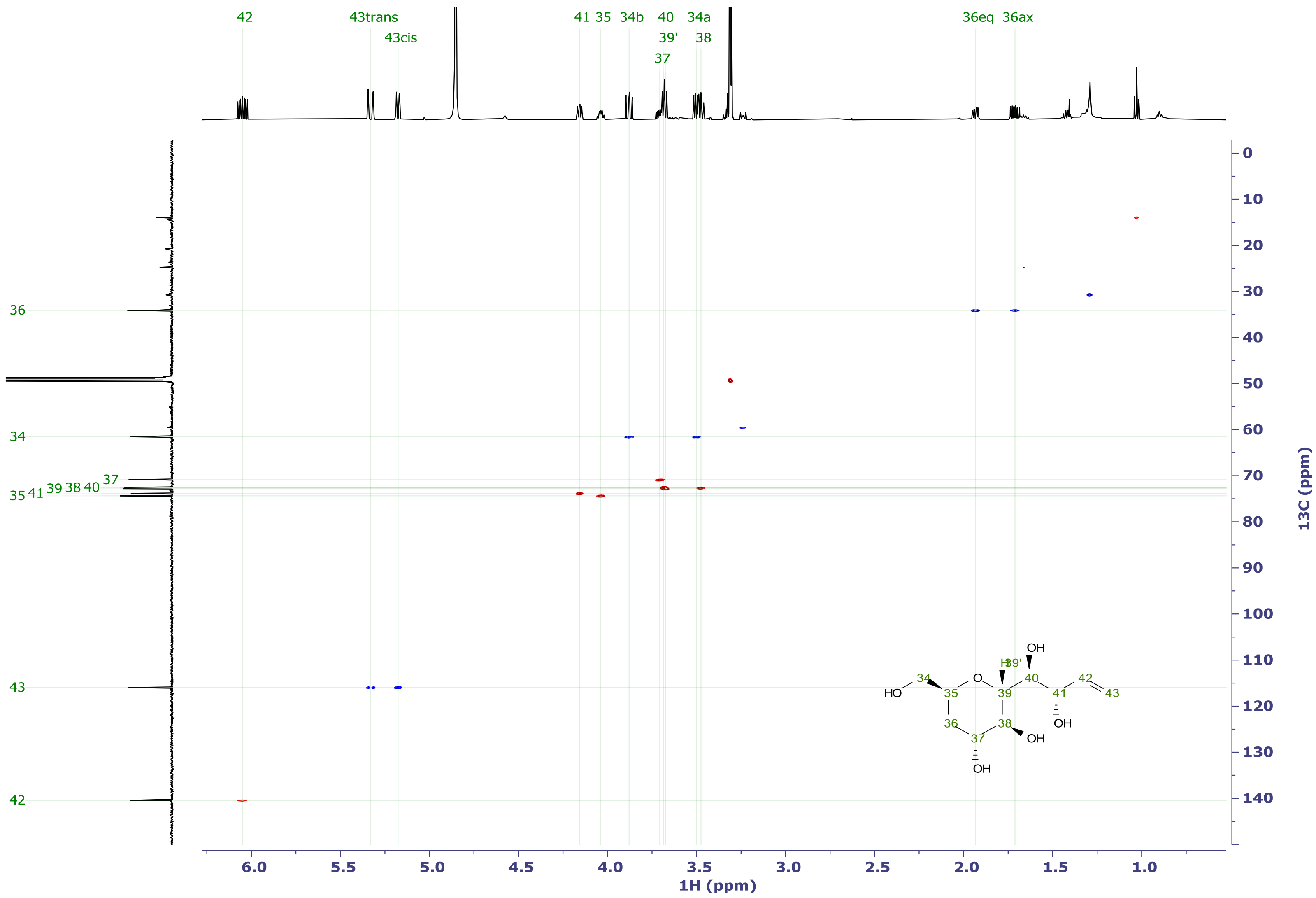

Compound 66: HMBC NMR ([D4]-MeOH)

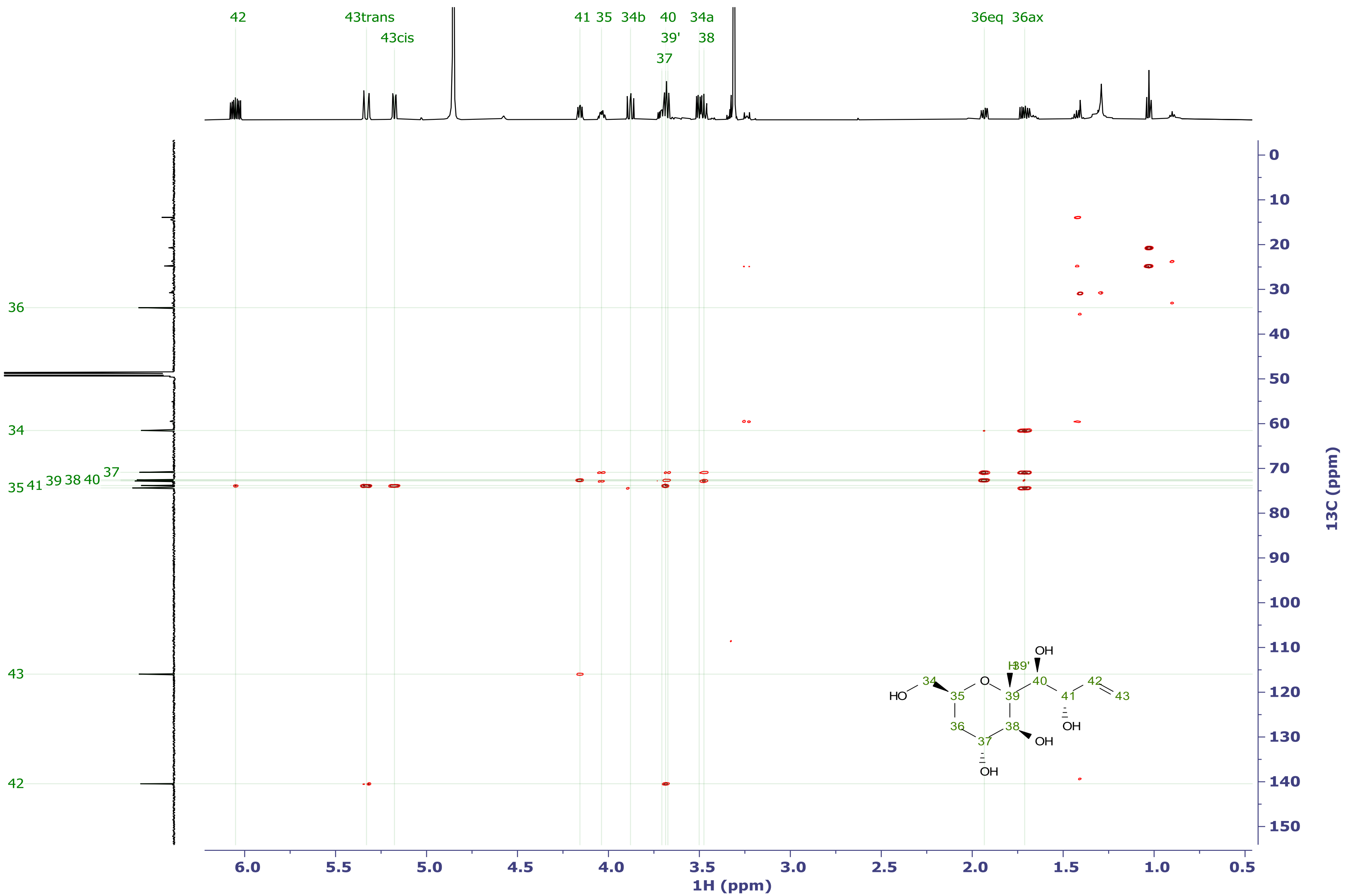

Compound 66: NOESY ([D$_4$]-MeOH)

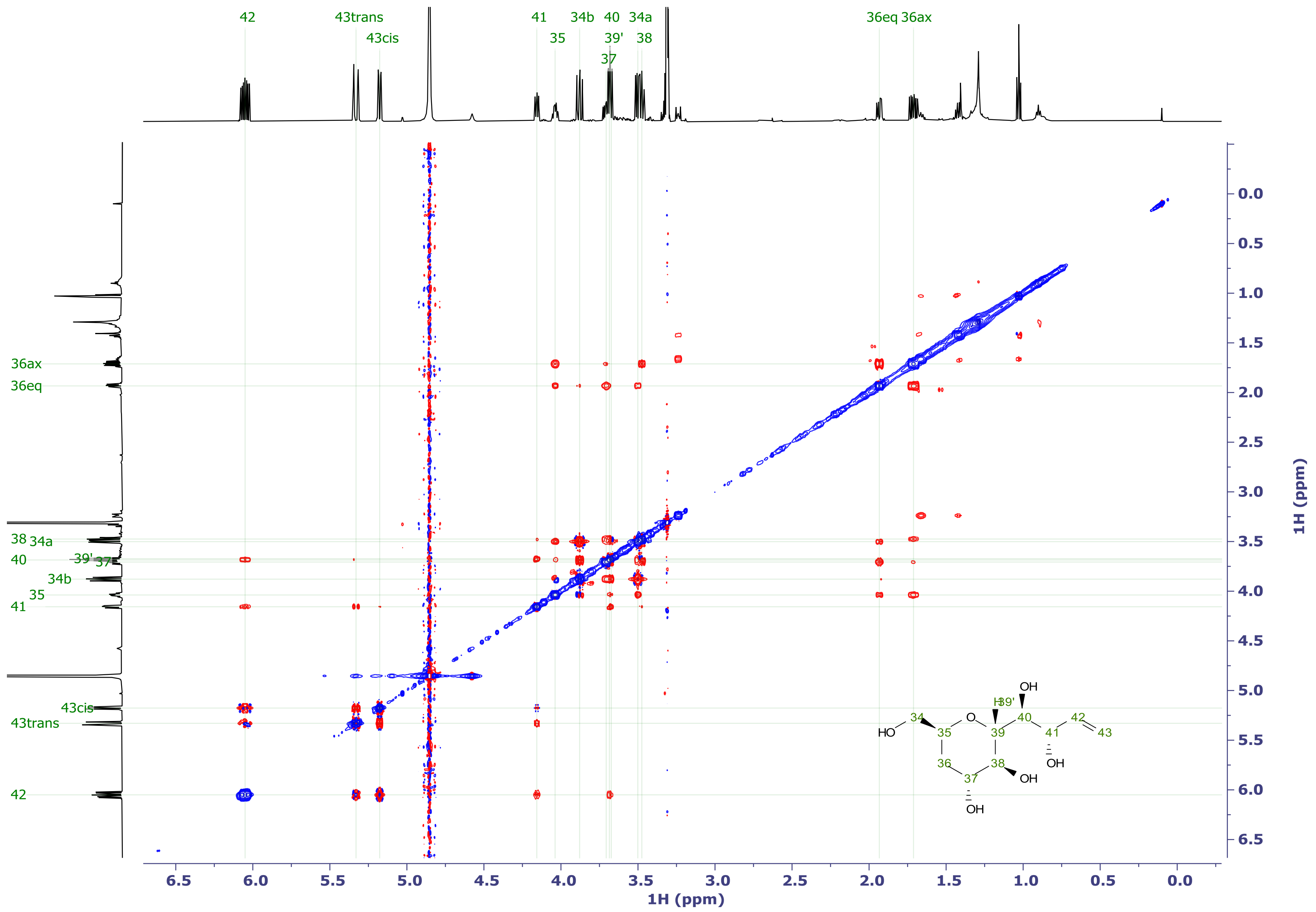

Compound S22: ^{1}H NMR (400 MHz, $CDCl_3$)

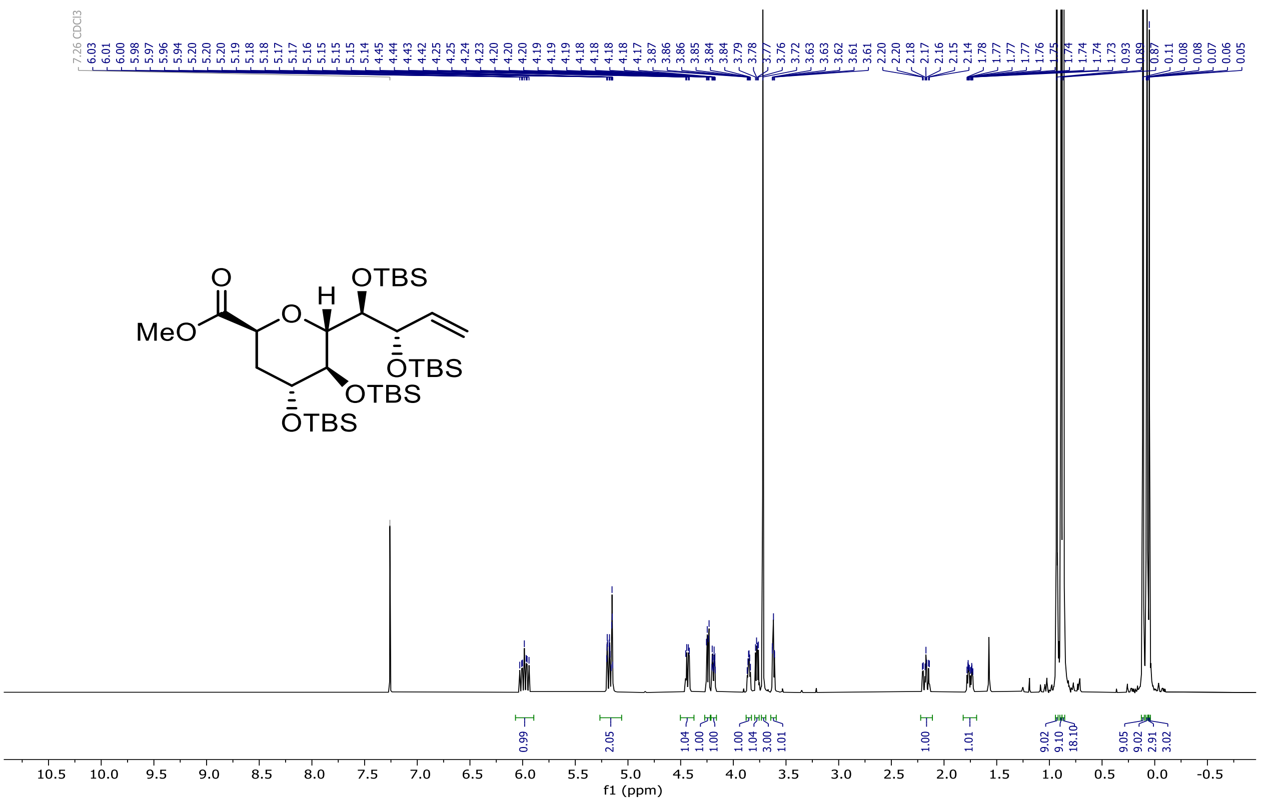

^{13}C NMR (101 MHz, $CDCl_3$)

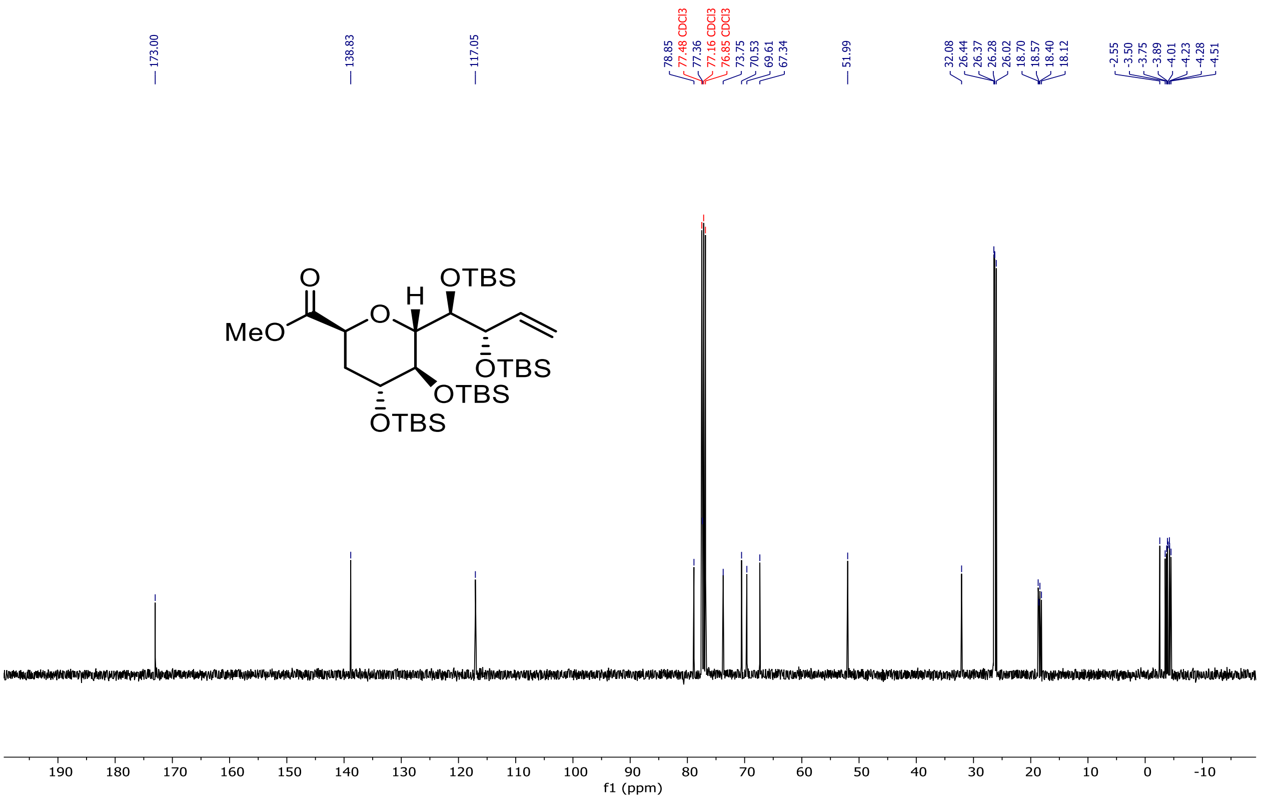

Compound 68: ^{1}H NMR (400 MHz, $CDCl_3$)

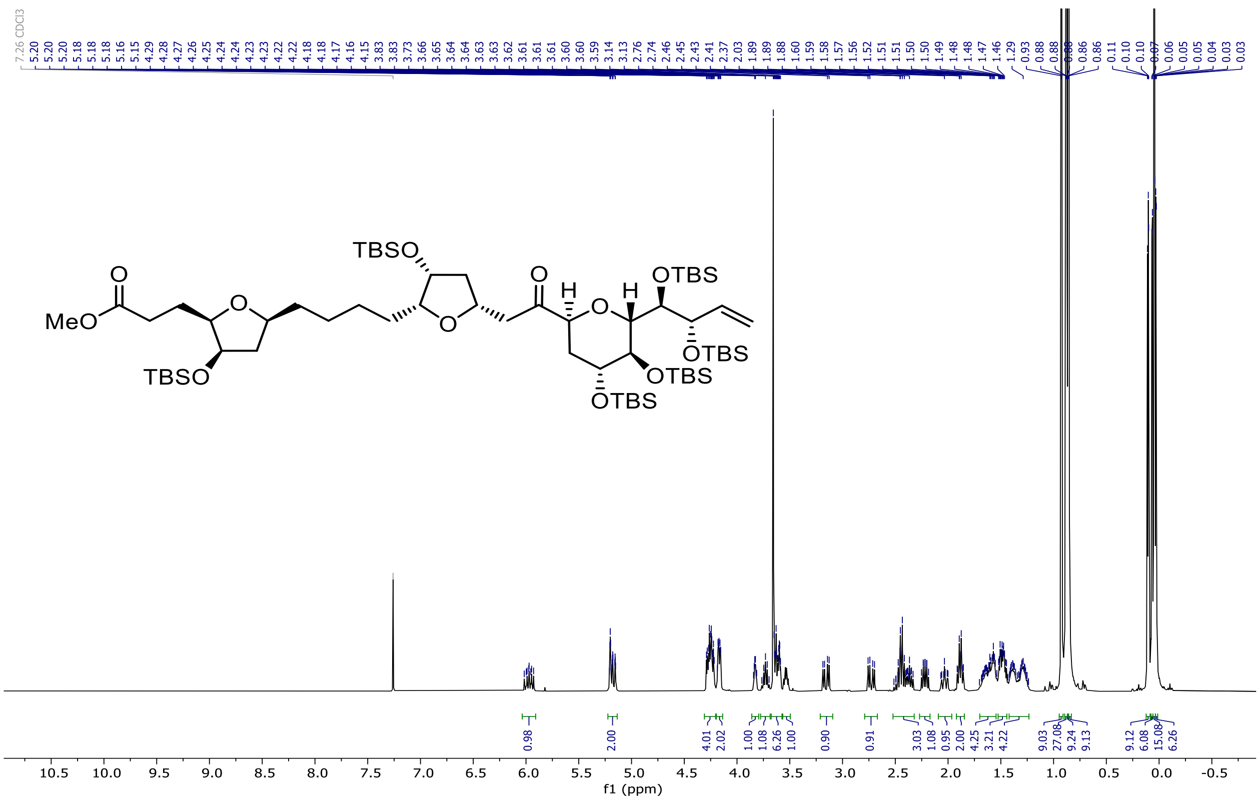

^{13}C NMR (101 MHz, $CDCl_3$)

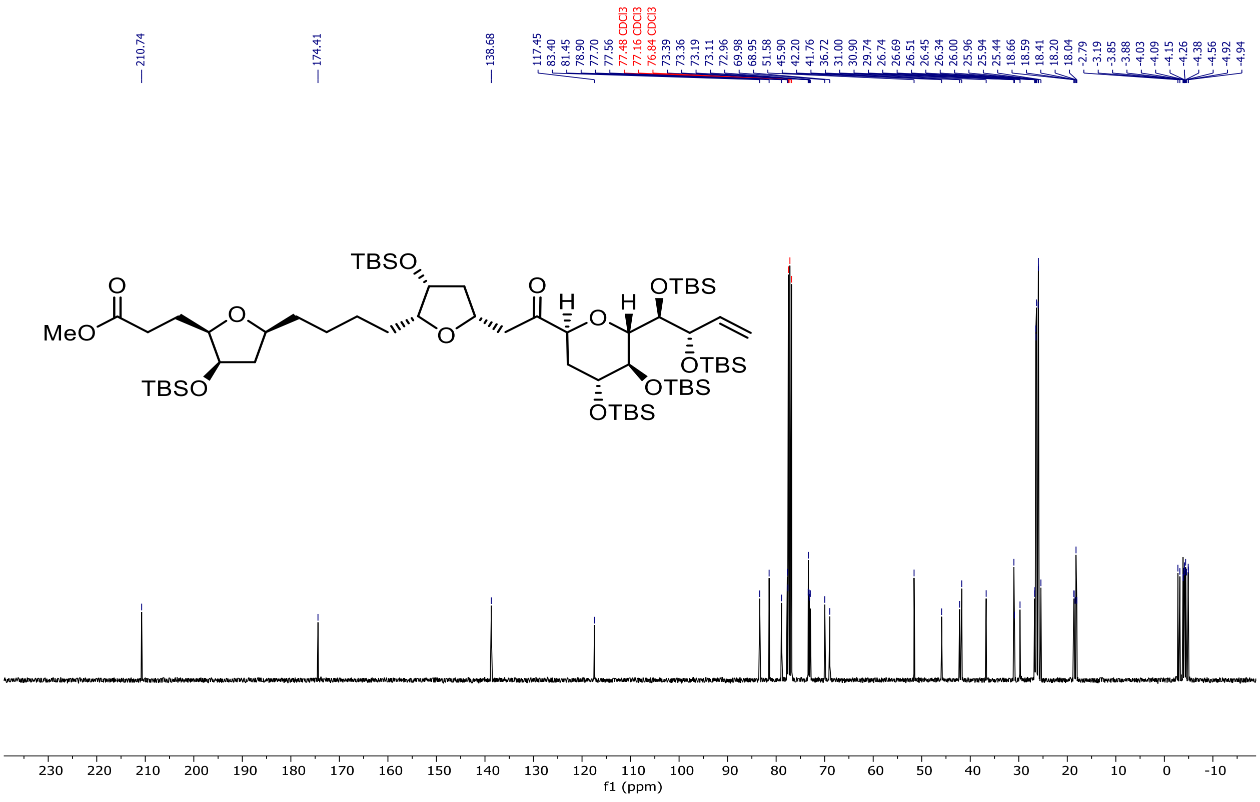

Compound 69: ^{1}H NMR (400 MHz, $CDCl_3$)

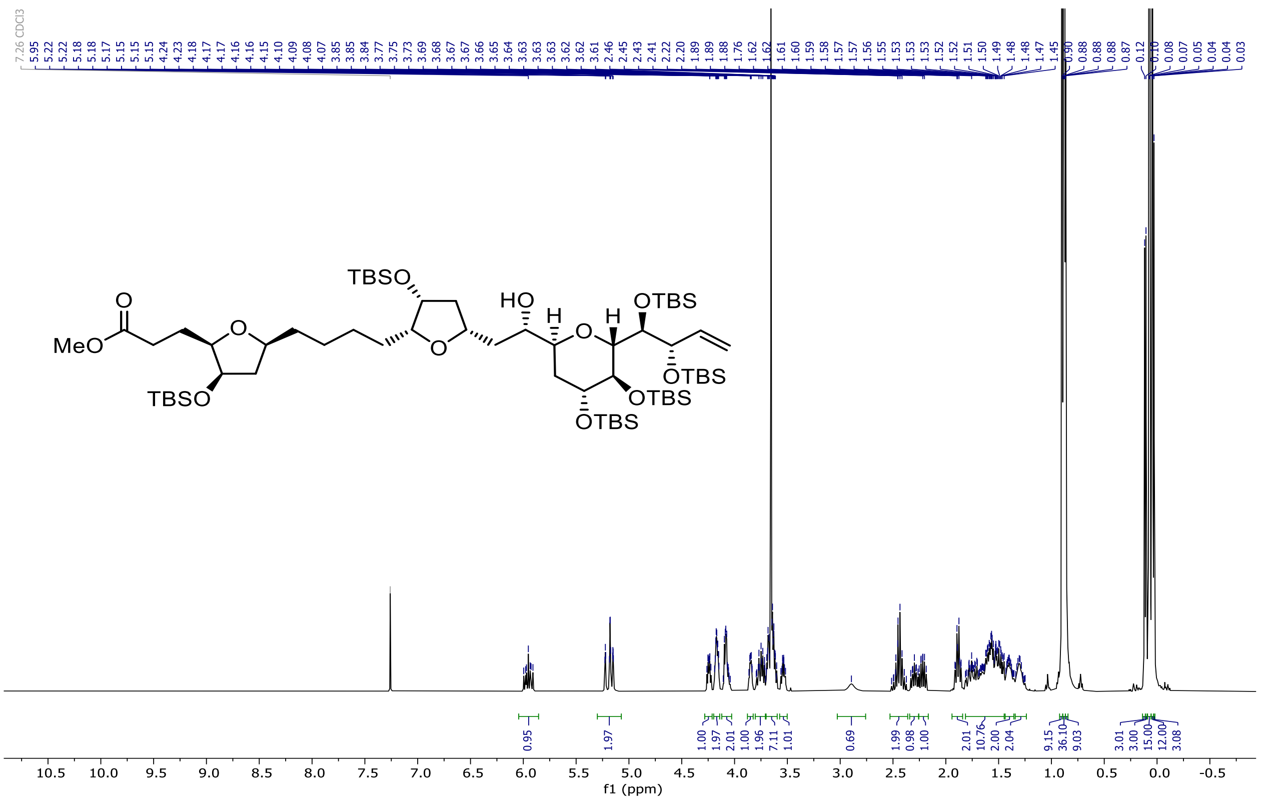

^{13}C NMR (101 MHz, $CDCl_3$)

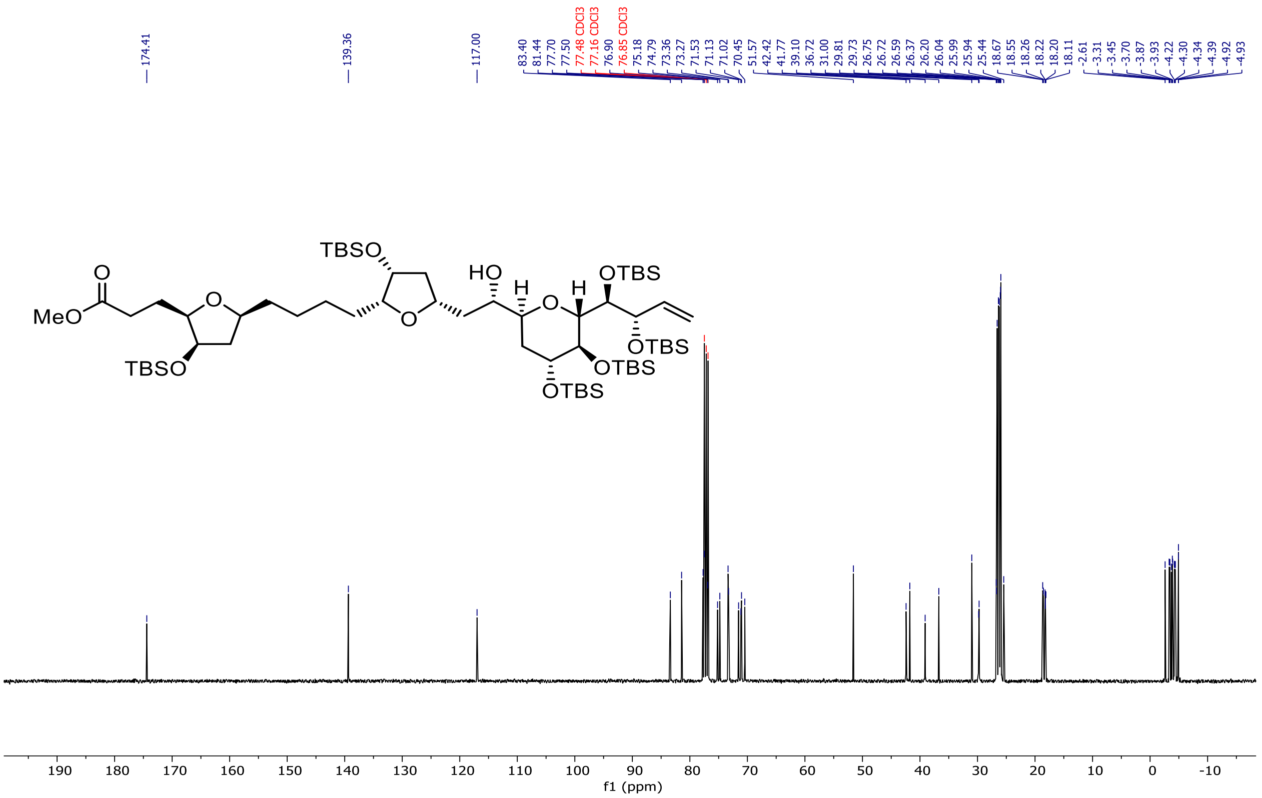

Compound 70: ^{1}H NMR (600 MHz, [D$_4$]-MeOH)

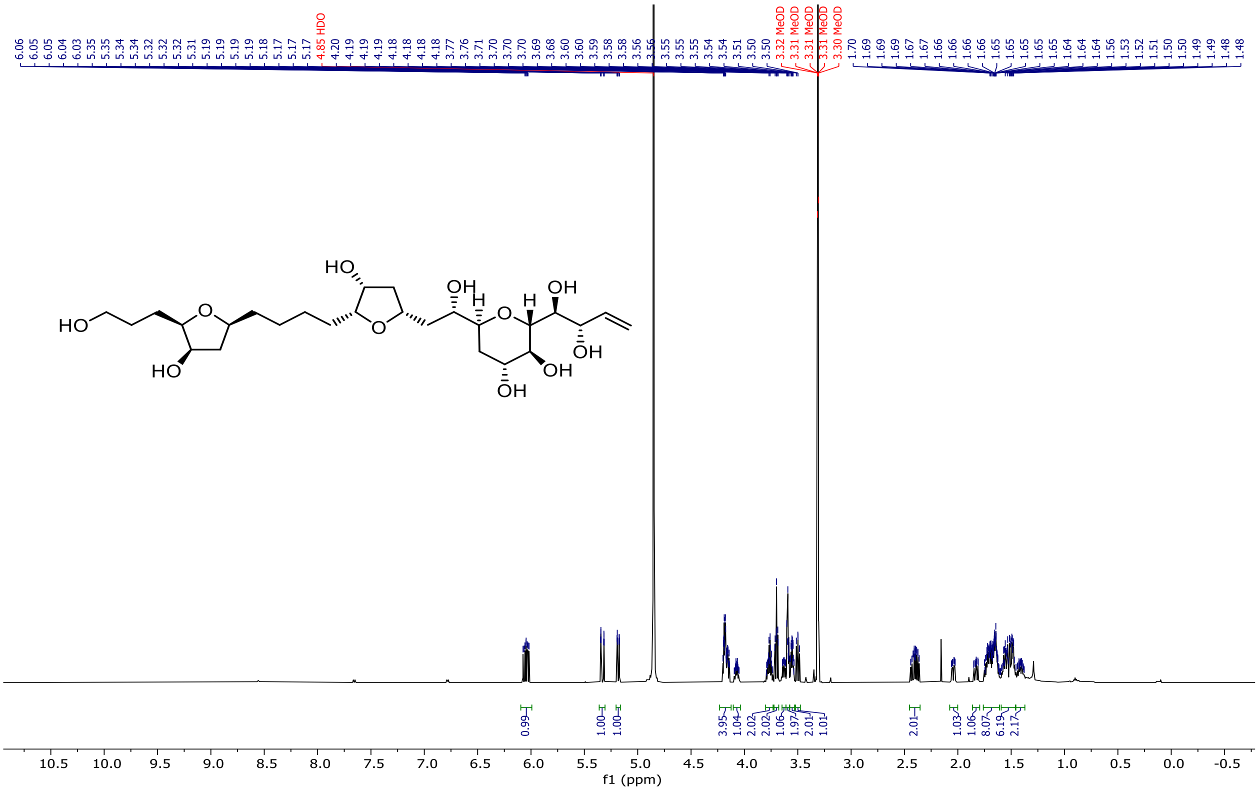

^{13}C NMR (151 MHz, [D$_4$]-MeOH)

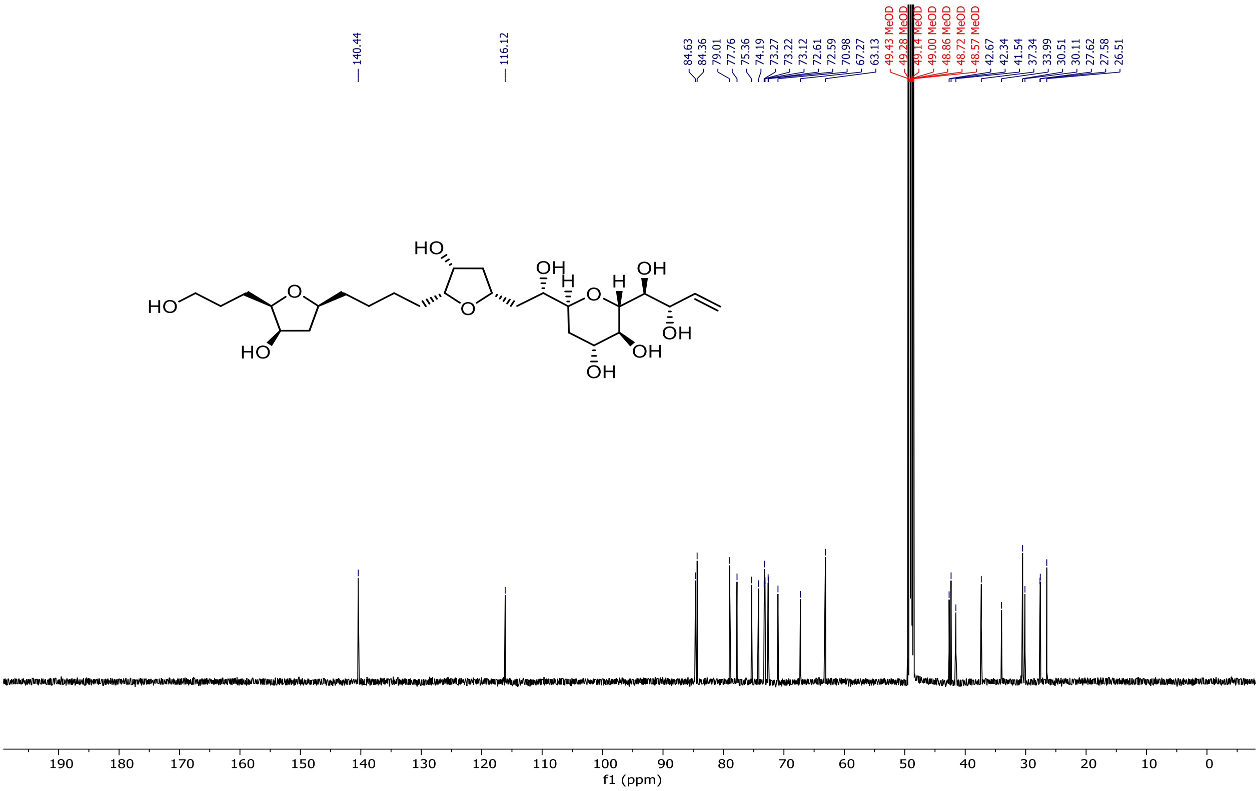

Compound 70: ^1H-^1H COSY ([D$_4$]-MeOH)

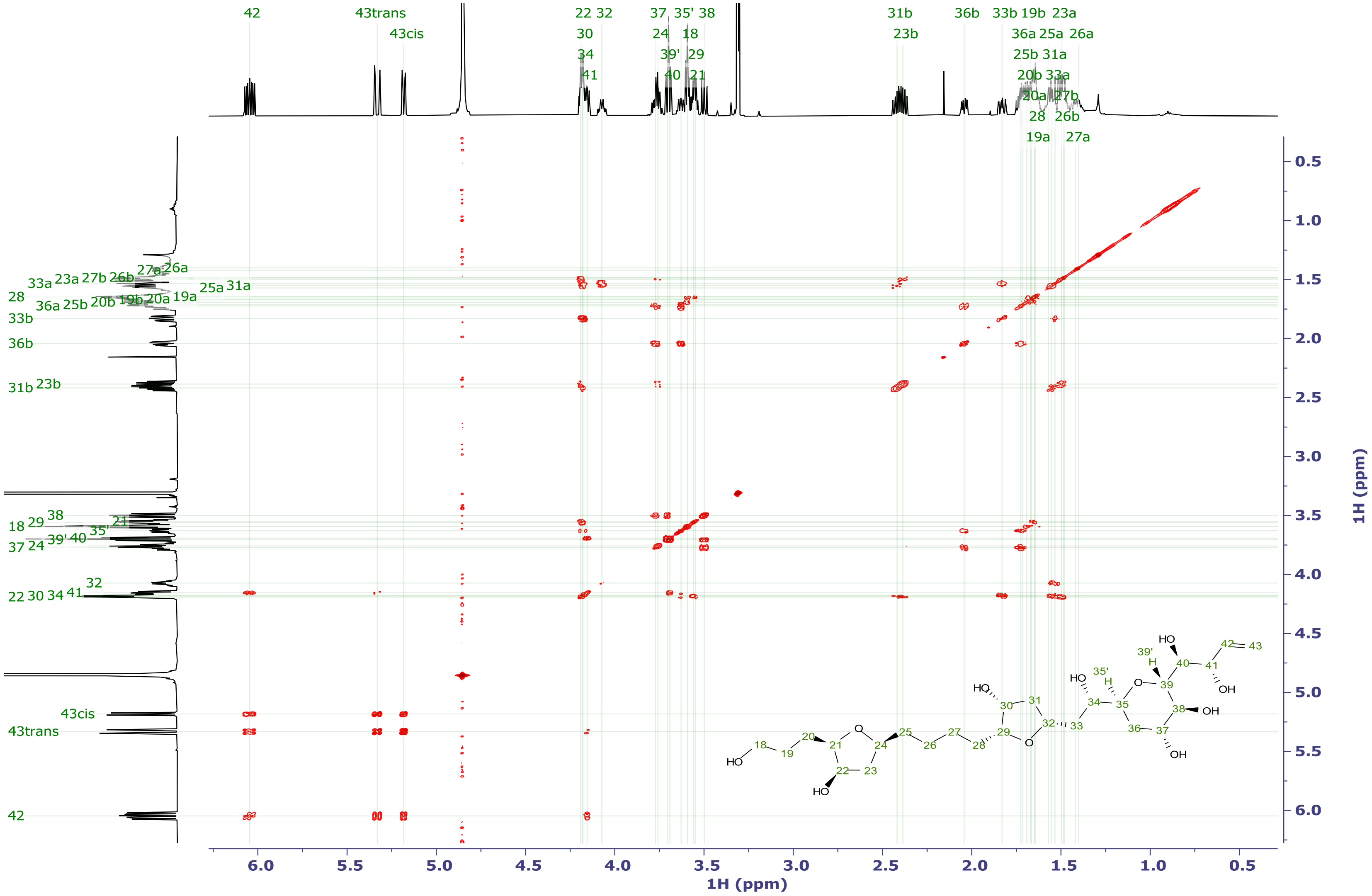

Compound 70: HSQC NMR ([D$_4$]-MeOH)

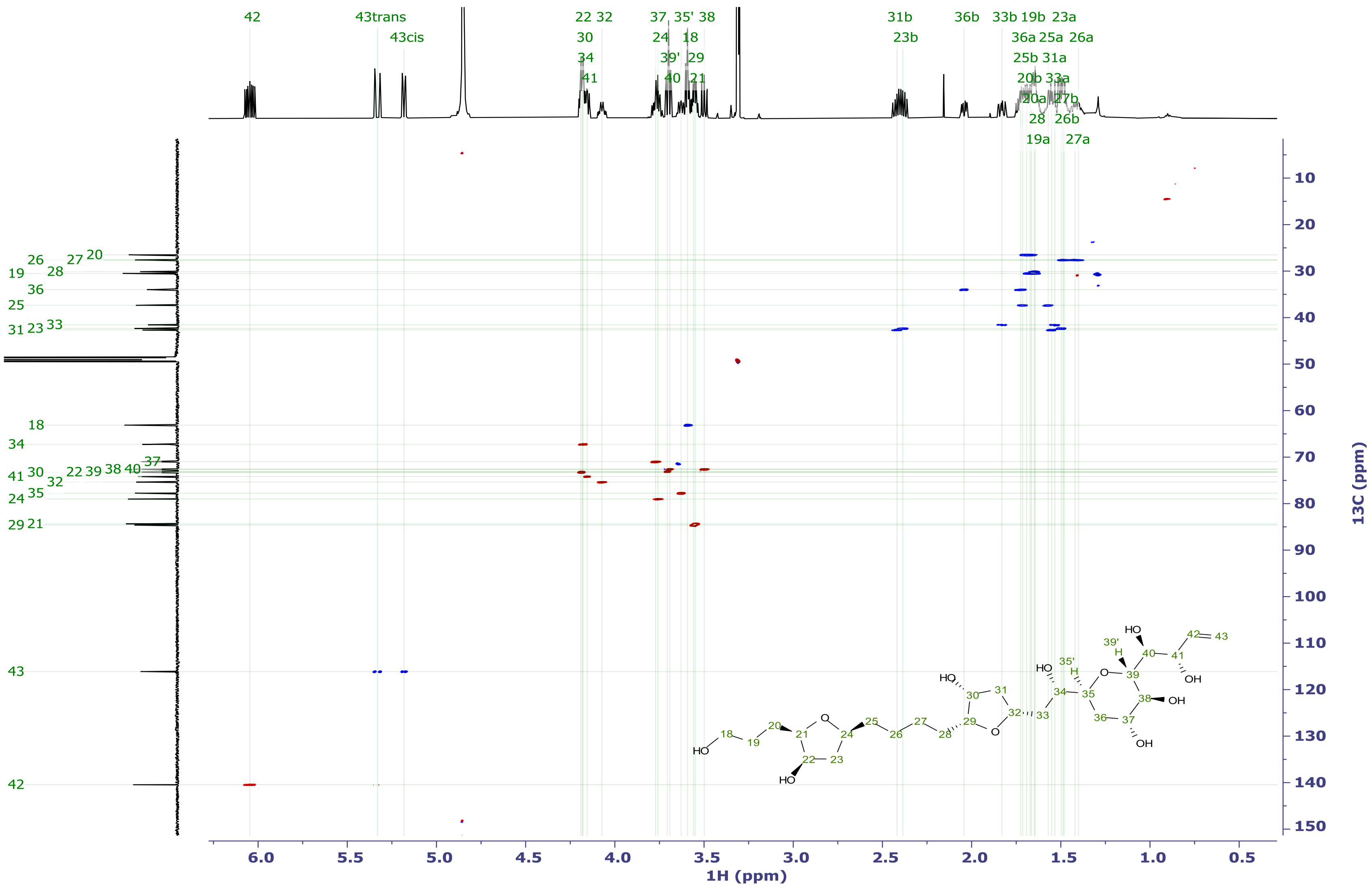

Compound 70: HMBC NMR ([D$_4$]-MeOH)

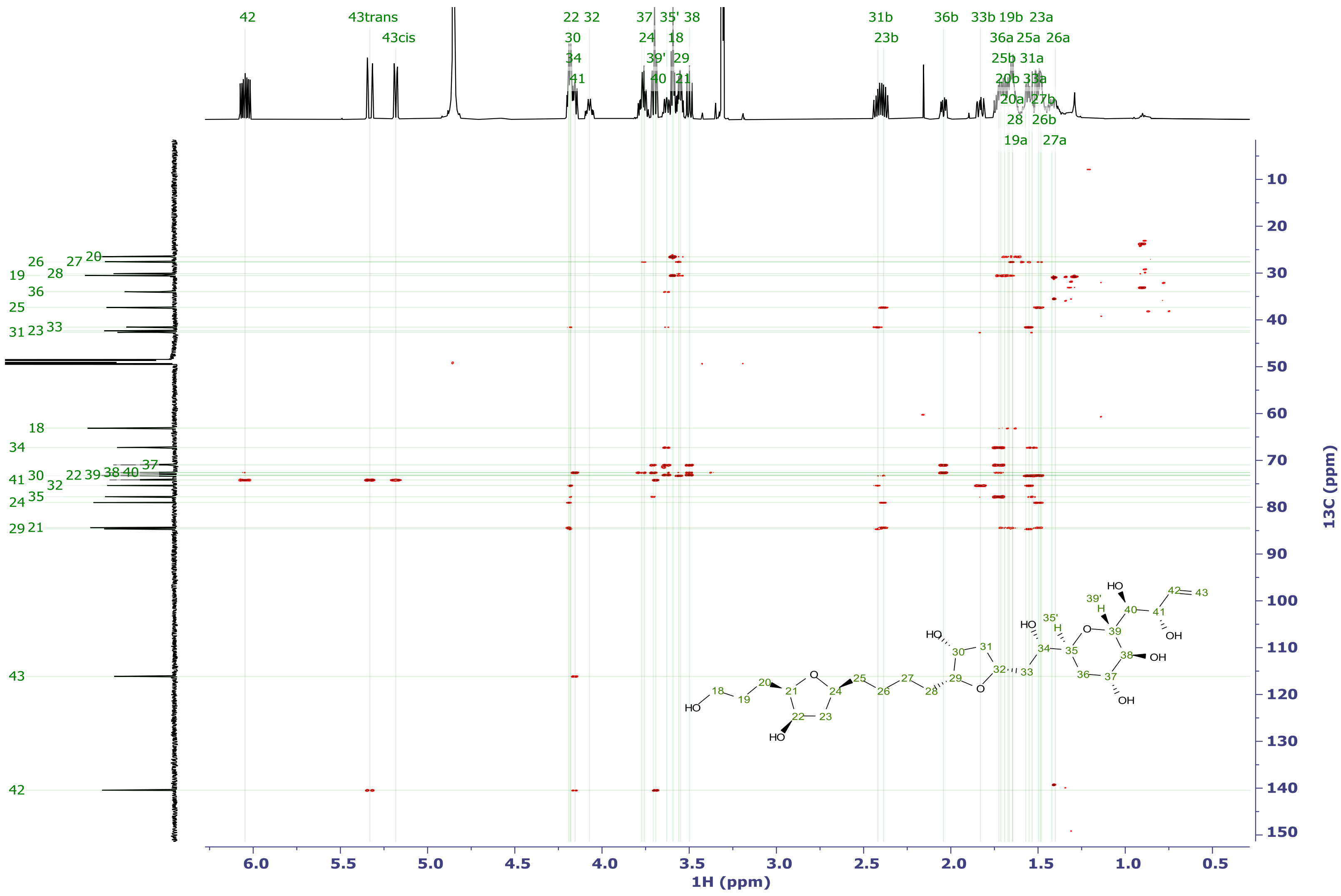

Compound 70: NOESY ([D$_4$]-MeOH)

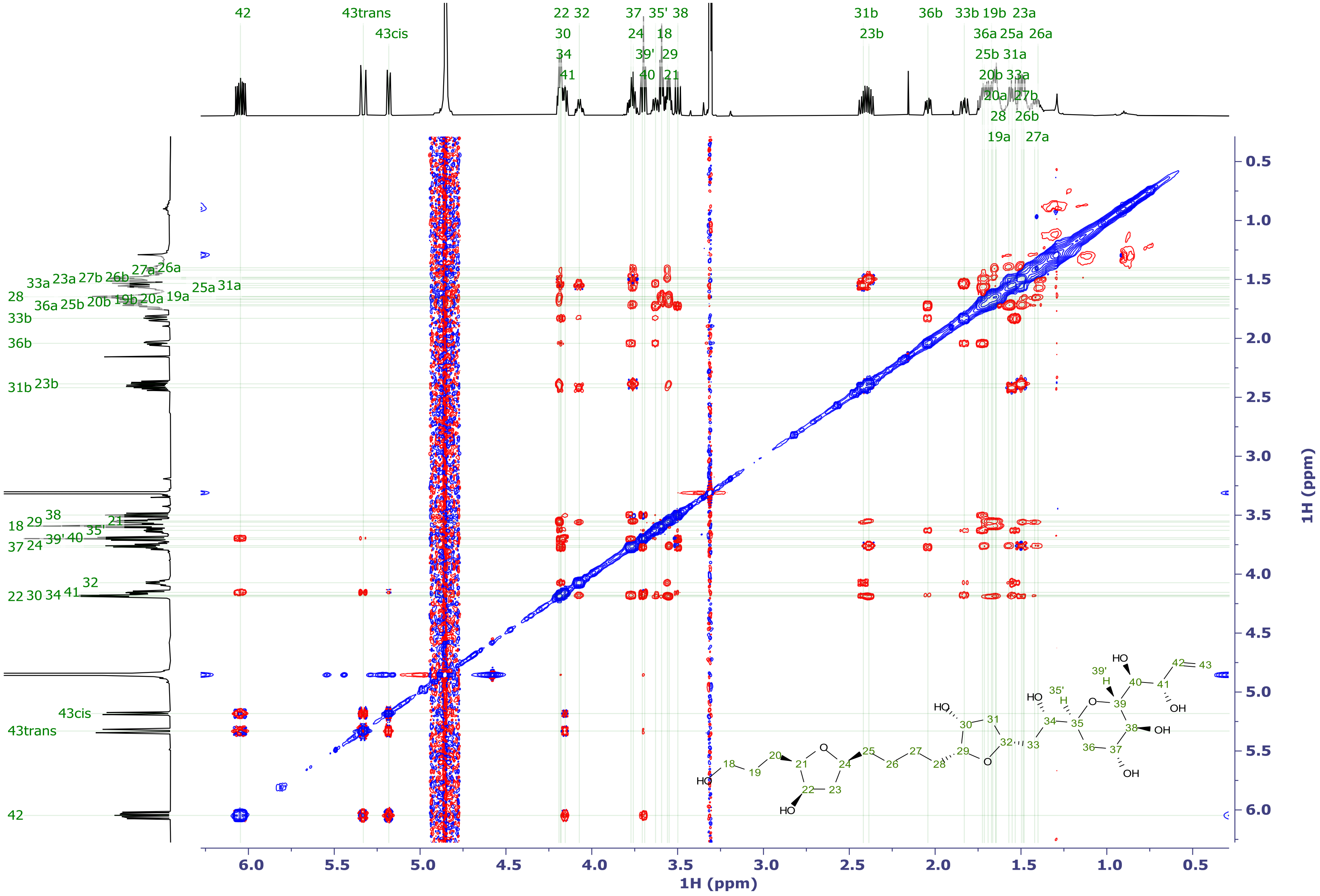

References

1. Das, B.; Mahender, G.; Sunil Kumar, V.; Chowdhury, N., Chemoselective deprotection of trityl ethers using silica-supported sodium hydrogen sulfate. *Tetrahedron Lett.* **2004,** *45* (36), 6709-6711.
2. Glaus, F.; Altmann, K.-H., Total Synthesis of the Bacterial RNA Polymerase Inhibitor Ripostatin B. *Angew. Chem. Int. Ed.* **2012,** *51* (14), 3405-3409.
3. Gampe, C. M.; Carreira, E. M., Cyclohexyne Cycloinsertion in the Divergent Synthesis of Guanacastepenes. *Chemistry – A European Journal* **2012,** *18* (49), 15761-15771.
4. Hoye, T. R.; Jeffrey, C. S.; Shao, F., Mosher ester analysis for the determination of absolute configuration of stereogenic (chiral) carbinol carbons. *Nat. Protocols* **2007,** *2* (10), 2451-2458.
5. Jiang, Z.-P.; Sun, S.-H.; Yu, Y.; Mándi, A.; Luo, J.-Y.; Yang, M.-H.; Kurtán, T.; Chen, W.-H.; Shen, L.; Wu, J., Discovery of benthol A and its challenging stereochemical assignment: opening up a new window for skeletal diversity of super-carbon-chain compounds. *Chem. Sci.* **2021,** *12* (30), 10197-10206.
6. Yin, N.; Wang, G.; Qian, M.; Negishi, E., Stereoselective Synthesis of the Side Chains of Mycolactones A and B Featuring Stepwise Double Substitutions of 1,1-Dibromo-1-alkenes. *Angew. Chem. Int. Ed.* **2006,** *45* (18), 2916-2920.
